# Integration of fluorescence in situ hybridization and chromosome-length genome assemblies revealed synteny map for guinea pig, naked mole-rat, and human

**DOI:** 10.1038/s41598-023-46595-x

**Published:** 2023-11-29

**Authors:** Svetlana A. Romanenko, Sergei F. Kliver, Natalia A. Serdyukova, Polina L. Perelman, Vladimir A. Trifonov, Andrei Seluanov, Vera Gorbunova, Jorge Azpurua, Jorge C. Pereira, Malcolm A. Ferguson-Smith, Alexander S. Graphodatsky

**Affiliations:** 1 Institute of Molecular and Cellular Biology, Russian Academy of Sciences, Siberian Branch, Novosibirsk, Russia; 2 Center for Evolutionary Hologenomics, The Globe Institute, The University of Copenhagen, Copenhagen, Denmark; 3 Novosibirsk State University, Novosibirsk, Russia; 4 Department of Biology, University of Rochester, Rochester, NY USA; 5 Department of Biochemistry and Molecular Medicine, The George Washington University, Washington, DC USA; 6 Animal and Veterinary Research Centre, University of Trás-os-Montes and Alto Douro, Vila Real, Portugal; 7 Cambridge Resource Centre for Comparative Genomics, Department of Veterinary Medicine, University of Cambridge, Cambridge, UK

**Keywords:** Cytogenetics, Comparative genomics, Genome evolution

## Abstract

Descriptions of karyotypes of many animal species are currently available. In addition, there has been a significant increase in the number of sequenced genomes and an ever-improving quality of genome assembly. To close the gap between genomic and cytogenetic data we applied fluorescent in situ hybridization (FISH) and Hi-C technology to make the first full chromosome-level genome comparison of the guinea pig (*Cavia porcellus*), naked mole-rat (*Heterocephalus glaber*), and human. Comparative chromosome maps obtained by FISH with chromosome-specific probes link genomic scaffolds to individual chromosomes and orient them relative to centromeres and heterochromatic blocks. Hi-C assembly made it possible to close all gaps on the comparative maps and to reveal additional rearrangements that distinguish the karyotypes of the three species. As a result, we integrated the bioinformatic and cytogenetic data and adjusted the previous comparative maps and genome assemblies of the guinea pig, naked mole-rat, and human. Syntenic associations in the two hystricomorphs indicate features of their putative ancestral karyotype. We postulate that the two approaches applied in this study complement one another and provide complete information about the organization of these genomes at the chromosome level.

## Introduction

Evolutionary chromosome rearrangements revealed by comparative analysis of different species reflect the history of speciation^1^ and make it possible to determine phylogenetic relationships^2^, to reconstruct ancestral karyotypes^3,4^, and to discern mechanisms of genome evolution and function^5,6^. Prior to the advent of DNA sequencing, the chromosome arrangement of the genome of an organism was observed using cytogenetic methods, predominantly by comparing differentially stained chromosomes and fluorescence in situ hybridization (FISH)^3,7^. To date, the karyotypes of more than 2000 mammalian species (about one-third of all modern mammals^8^), representing all existing clades, have been characterized in this way^9^.

Currently, the study of vertebrate genome evolution is being revolutionized by advances in DNA sequencing technology that allow researchers to create near-complete and error-free de novo genome assemblies^10,11^. By themselves, sequence data are of limited use for studying fundamental and applied biological questions, as they do not provide insight into how the genome is organized into chromosomes^12^. Chromosome-level assemblies are emerging as the gold standard for de novo whole genome sequencing^13–16^.

For vertebrates, the availability of high-quality chromosome-level reference genomes has led to significant outcomes in functional, comparative, and population genomics, and in conservation biology^15,17–21^. The main approach to study the organization of the genome in this kind of research is high-throughput chromosome conformation capture (Hi-C) by combining proximity ligation sequencing^22,23^ with massive parallel whole genome sequencing^24,25^. The average resolution of the method is several thousand base pairs, which is tens of times greater than the capabilities of microscopy.

New approaches do not always provide a direct link to information on the evolution of the vertebrate genome obtained using cytogenetic approaches^11^. Proper organization of sequenced fragments, such as contigs and scaffolds, in relation to each other, as well as the positioning of superscaffolds into structured chromosomes, is often a challenging step in the construction of a reliable genome reference^26,27^. Sequencing and positioning of repeat-containing sequences and genomes with segmental duplications is a daunting task^28,29^.

In recent years, the need for close integration of the latest advances in cytogenetics, genome sequencing, epigenomics, and cell biology has been actively emphasized^12,30^. In addition to increasing the quality assurance of bioinformatic genome assembly, the need to validate assemblies with different approaches, all of which are mainly based on FISH, has been noted^11,27,31–34^. The integration may be much more successful in answering fundamental biological questions than genomic or cytogenetic approaches alone^12^.

Many organisms important for biomedical research have incomplete genome assemblies, or the quality of the assemblies has not been verified by molecular cytogenetic methods.

The naked mole-rat (HGL, *Heterocephalus glaber*) is the longest-lived subterranean rodent endemic to northeast Africa^35^. High tolerance to hypoxia, hypercapnia, and soil-based toxins, along with strict resistance to neoplasia and experimental tumorigenesis, make naked mole-rats an ideal model for research on cancer, longevity, and disease resistance^36–43^. The *H. glaber* genome was sequenced, but all assemblies were relatively fragmented and contained about 15% of unfilled gaps^41,44–46^. Recently the high-quality genome assembly to superscaffolds or pseudo-chromosomes has been obtained^43^; however, the superscaffolds were not correlated with the physical map of *H. glaber* chromosomes.

The guinea pig (CPOR, *Cavia porcellus*) belongs to the same order Hystricomorpha as naked mole-rat (divergence of 39.5 mya). It is an important model organism used in the study of vaccines, the research and diagnosis of infectious diseases and such human diseases as diabetes, asthma and others^47–51^. Guinea pig meat is an important source of high-quality animal protein in South America, which is of great economic significance^52^. All these features make it extremely important to study the guinea pig genome deeply. After sequencing in 2008, the assembly of the guinea pig genome has been improved several times but it continues to consist of large unplaced scaffolds^26,53,54^. A chromosome-level assembly was never completed. Several years ago, a comparative chromosome map of guinea pig and human was obtained which, for the first time, made it possible to link the complete genomes of these species^55^.

The present work aimed to use *H. glaber, C. porcellus,* and human probes to link Hi-C scaffolds obtained for *H. glaber, C. porcellus* to their chromosomal maps and thereby obtain chromosomal-level assemblies for both rodent species. Linkage to a physical map of chromosomes provides information about the orientation of the sequences and reflects the location of centromeres and heterochromatic blocks in the genome. The resulting high-quality reference genome should prove useful as a template for studying other species and provide a solid basis for comparative and evolutionary genomics.

## Materials and methods

### Cells

The research was completed using equipment and materials from the Core Facilities Centre, “Cryobank of cell cultures”, at the Institute of Molecular and Cellular Biology Siberian Branch of Russian Academy of Sciences (Novosibirsk, Russia). The study involved work exclusively with cell cultures. Animals or any parts of animals were not used.

The *C. porcellus* (male) primary fibroblast cell lines were derived from a skin biopsy and provided by the National Cancer Institute (NCI), USA. The establishment of the *C. porcellus* (male) primary fibroblast cell lines used in the work was described earlier^55^. For this work, *C. porcellus* cells were cultured in alpha MEM supplemented with 15% of FBS (Gibco), 10^5^ U/L penicillin, and 100 mg/L streptomycin, and 2.5 mg/L amphotericin B at 37 °C in the presence of 5% CO_2_.

The immortalized *Heterocephalus glaber* fibroblasts, NSF8, were obtained from skin in the Department of Biology, University of Rochester, USA, and provided for joint research. The establishment of the cell line was described earlier^56^. *H. glaber* cells were cultured in alpha MEM supplemented with 15% of FBS (Gibco), 10% AmnioMAX II Complete Medium (Gibco), 5 ng/mL bFGF, 10^5^ U/L penicillin, and 100 mg/L streptomycin, and 2.5 mg/L amphotericin B at 32 °C in the presence of 5% CO_2_.

The MRC-5 is a commercially available diploid cell line established from human male embryonic lung fibroblasts. The cell line was obtained from the “State Scientific Center for Virology and Biotechnology “Vector”, Koltsovo, Novosibirsk region, Russia”, and was cultured under the same conditions as *C. porcellus* cells.

### Chromosome preparation and chromosome staining

Chromosomal suspensions from cell cultures, metaphase preparations, and GTG-, CBG-, and CDAG-banding were made as previously described^55,57–61^.

### Generation of painting probes for *C*. *porcellus*, *H*. *glaber*, and human

The set of human (2n = 46) chromosome-specific painting probes was generated in the Cambridge Resource Centre for Comparative Genomics (UK) and provided for collaborative research use^62^. The set of *C. porcellus* (2n = 64) chromosome-specific probes was generated at the National Cancer Institute (USA) as described previously^55^. Chromosome-specific painting probes of *H. glaber* (2n = 60) were generated in the Cambridge Resource Centre for Comparative Genomics (UK) from the cell line NSF8^56^ as described previously^63^.

### Telomeric and ribosomal DNA probes

The telomeric DNA probe was generated by PCR with oligonucleotides (TTAGGG)_5_ and (CCCTAA)_5_^64^. Clones of human ribosomal DNA (rDNA) containing a partial 18S ribosomal gene, the full 5.8S gene, a part of the 28S gene, and two internal transcribed spacers were obtained as described elsewhere^65^. Labeling was performed using PCR by incorporation of biotin-dUTP and digoxigenin-dUTP (Sigma).

### Fluorescence in situ hybridization (FISH)

We used sequential GTG-banding^59^ and FISH for precise chromosome identification^66^. VideoTesT-FISH and VideoTesT-Karyo (aMicro, Saint-Petersburg, Russia) digital imaging systems were used in this study. Hybridization signals were assigned to specific chromosome regions defined by GTG-banding patterns.

### Genome assemblies

Chromosome-length genome assemblies (HetGla_female_1.0_HiC and Cavpor3.0_HiC) of naked mole-rat (*Heterocephalus glaber*)^25,45^ and domestic guinea pig (*Cavia porcellus*)^25,53^ were downloaded from the DNA Zoo Consortium website (https://www.dnazoo.org/assemblies/Cavia_porcellus; https://www.dnazoo.org/assemblies/Heterocephalus_glaber). For human, we used reference genome assembly version GRCh38.p13.

### Repeat masking and whole genome alignments

Tandem and interspersed repeats in genome assemblies of three species were detected using three tools: RepeatMasker v4^67^, TRF^68^, and Windowmasker^69^. Human and Rodentia repeat libraries from RepBase^70,71^ were used for the RepeatMasker run. Bedtools^72^ package was used to mask detected repeats in the assemblies. Multiple whole genome alignment of three masked genomes was performed using Progressive Cactus^73,74^.

### Synteny blocks and visualization

Raw synteny blocks were extracted from the multiple whole genome alignment using halSynteny v2.2^75^ with options --minBlockSize 50000 --maxAnchorDistance 50000. The procedure is pairwise and requires setting one of the genomes in alignment as a reference and the second as a query. By this method, we generated synteny blocks for all possible combinations of target and query species and got six psl files. Next, raw blocks were filtered in three stages to get a set of synteny blocks comparable to the FISH data. First, shorter blocks completely embedded in the longer ones were filtered out. Second, we removed “short” (L < 1 Mbp) blocks located further than 3 Mbp from other blocks corresponding to the same pair of target and query chromosomes. This was done to remove short isolated blocks and, at the same time, keep short but clustered ones (Supplementary Tables S1–S3). Third, we merged adjacent (distance < 1 Mbp) blocks corresponding to the same chromosomes (Supplementary Tables S4–S6). Finally, only blocks longer than 1 Mbp were retained.

Filtration and visualization of synteny blocks were performed by draw_synteny.py script from the MACE package (https://github.com/mahajrod/MACE).

## Results

### *Heterocephalus glaber* karyotype and flow karyotype description

**Figure 1:**
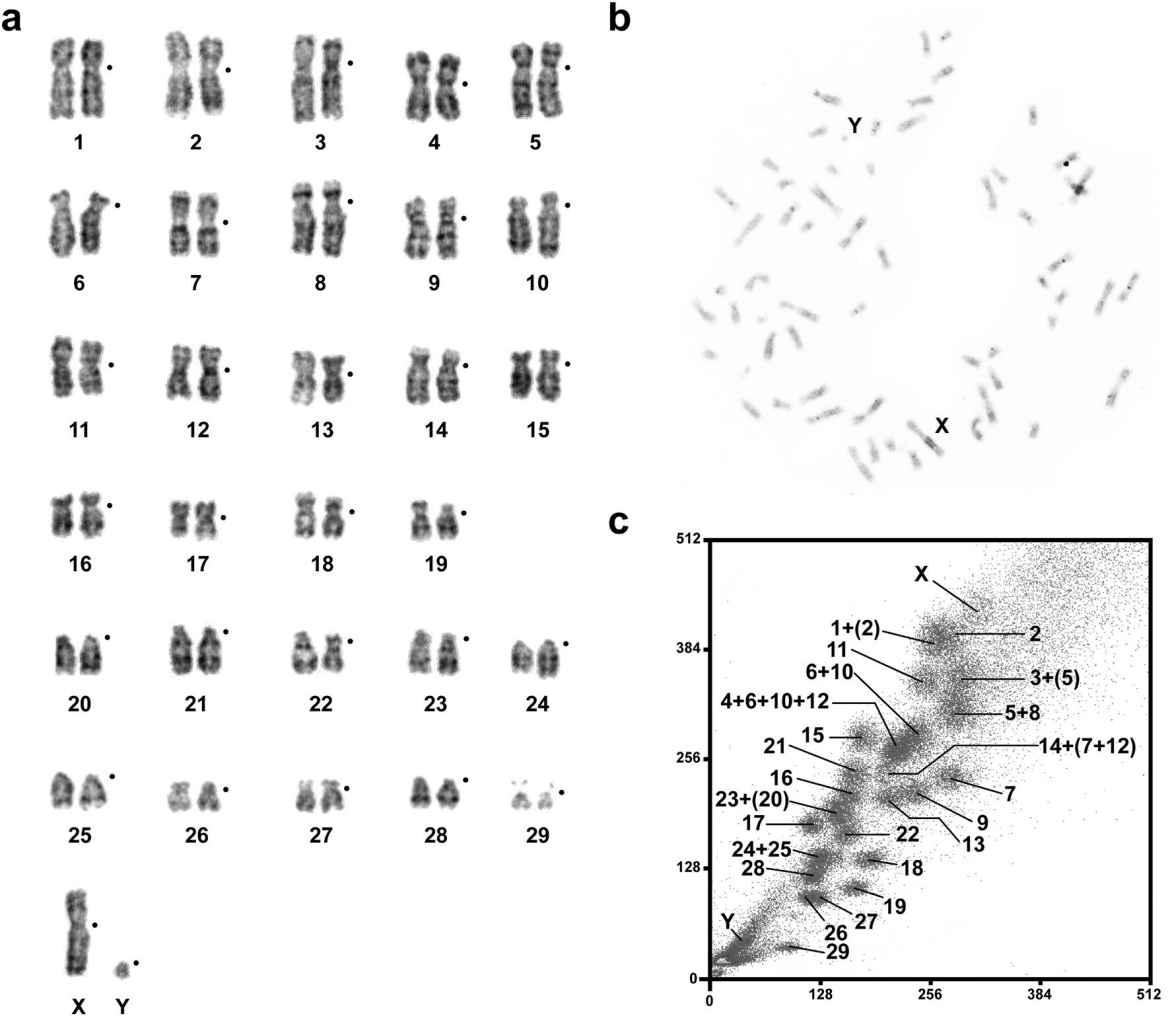
A male of *Heterocephalus glaber* wits 2n = 60. (**a**) GTG-banded karyotype, black dots mark centromere positions. (**b**) CBG-banded metaphase plate. (**c**) Flow karyotype showing the assignment of flow peaks to specific chromosomes. The weaker chromosome-specific signals revealed during FISH are indicated in brackets.

The *H. glaber* cell line, NSF8, was karyotyped at different passages (up to 39) and demonstrated a stable karyotype with 2n = 60. The X is a large submetacentric chromosome and the Y is the smallest acrocentric chromosome in the complement (Fig. 1a). The 18S/28S-rDNA probe gave the only signal in the p-arms of chromosome HGL 29 (Supplementary Fig. S1).

CBG-banding revealed tiny heterochromatic blocks in pericentromeric regions of a few chromosomes. Most chromosomal arms had uneven staining. The X chromosome carried a block of gray heterochromatin in the q-arm. The Y chromosome did not show a typical mammalian C-positive staining (Fig. 1b). Blocks of GC-rich heterochromatin were detected in the short arms of chromosome 29 by CDAG (causing the formation of satellites) indicating the localization of ribosomal gene clusters (Supplementary Fig. S2). The telomeric repeat (TTTAGG)_n_ probe marked the distal parts of all chromosomal arms and did not reveal any interstitial signals (Supplementary Fig. S1).

The *H. glaber* karyotype resolved into 26 flow peaks (Fig. 1c). Chromosome paints were generated from each of the 26 chromosomal pools and assigned by FISH to GTG-banded *H. glaber* chromosomes. Some of the pools contain a mixture of DNA from several chromosomes, which demonstrate different intensities during FISH (Fig. 1c).

### Comparative chromosome painting

**Figure 2:**
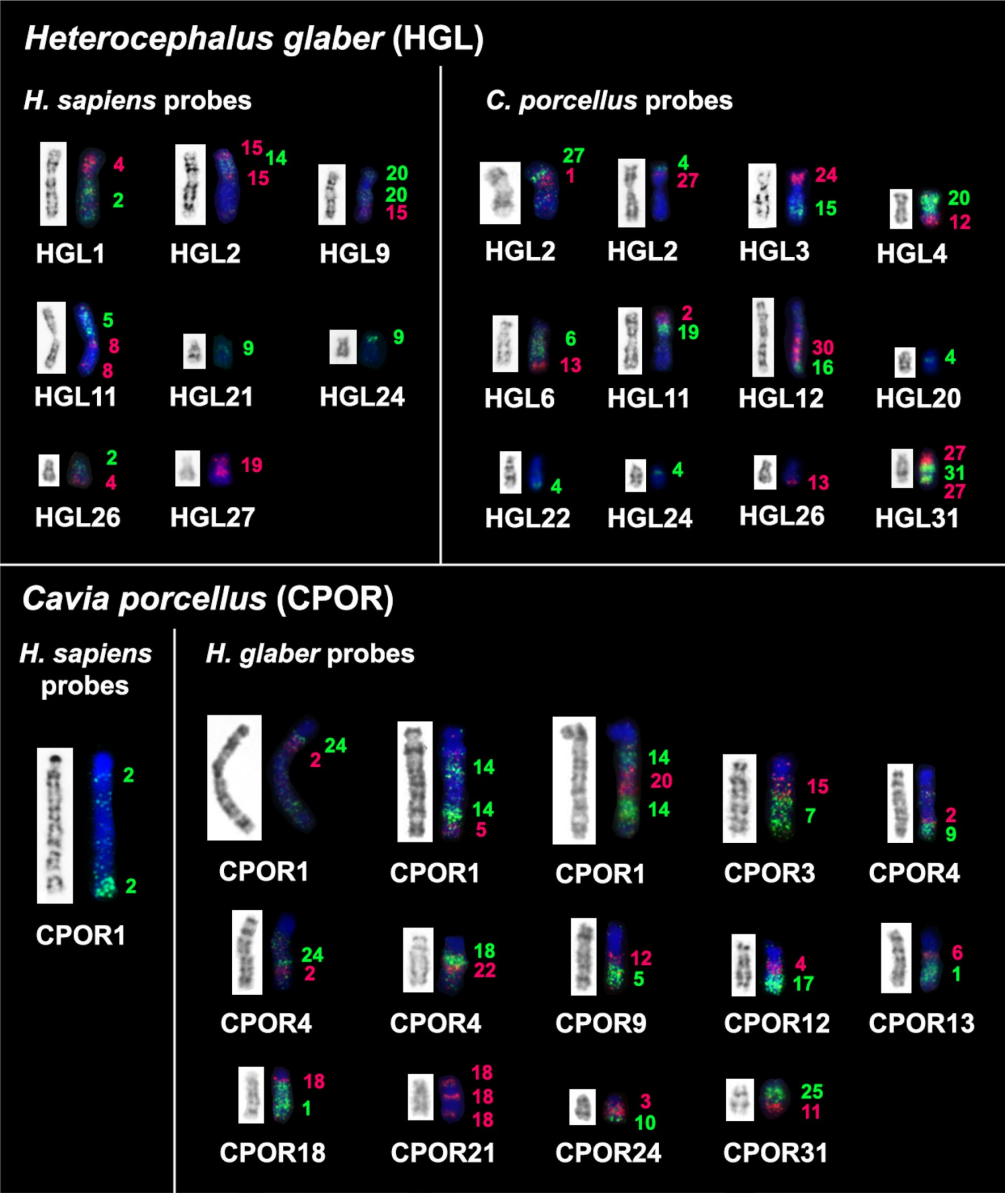
FISH of chromosome-specific probes on metaphase chromosomes of *Heterocephalus glaber* (HGL) and *Cavia porcellus* (CPOR). The upper part of the figure shows the localization of human and guinea pig probes onto naked mole-rat chromosomes. The bottom part of the figure shows the localization of human and naked mole-rat probes onto guinea pig chromosomes. The chromosome-specific probe number, colored red or green corresponding to the fluorochrome signal, is shown on the right of the chromosome.

Bidirectional comparative chromosome painting for *C. porcellus* and *Homo sapiens* was made earlier^55^. Here we localized *H. glaber* painting-probes to *C. porcellus* chromosomes and vice versa. The set of *H. sapiens* probes was localized to *H. glaber* also. The quality of probe hybridization varied greatly. Only a few *H. glaber* probes were successfully localized on human chromosomes. Examples of fluorescence in situ hybridization are shown in Fig. 2.

### Hi-C data

The final Hi-C data are presented in the form of tables reflecting the homology of the chromosomes of each species and the chromosomes of two other species, indicating the genomic coordinates and sizes of each region (Supplementary Tables S4–S6).

**Figure 3:**
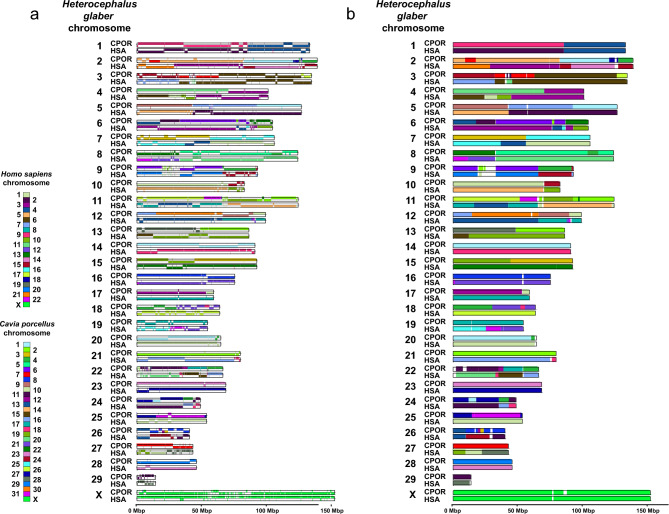
Syntenic blocks identified in the naked mole-rat genome in chromosome-level assemblies’ comparison with the guinea pig (CPOR) and human (HSA). Each chromosome is divided into an upper and lower track. If the orientation of the blocks is the same in the reference genome and the query genome, then the block is drawn on the upper track; if not, then on the lower one. Minimal length of the syntenic block: (**a**) 1 Mbp threshold. (**b**) 5 Mbp threshold.

We identified syntenic blocks shared among three studied species through pairwise alignments and visualized the syntenic homologies (Fig. 3, Supplementary Figs. S3–S5). Next, we cross-referenced the chromosome painting and Hi-C-derived synteny data across three species, carefully checking all individual homologies. Some homologies were not initially revealed by comparative chromosome painting and were subsequently verified by FISH, while other smaller homologies were beyond the resolution of available painting probe sets. The homologies not previously detected by FISH corresponded mostly to 1–3 Mbp fragments. The 1–3 Mbp could be a limit of chromosome painting resolution for heterological FISH of chromosome-specific probes.

**Figure 4:**
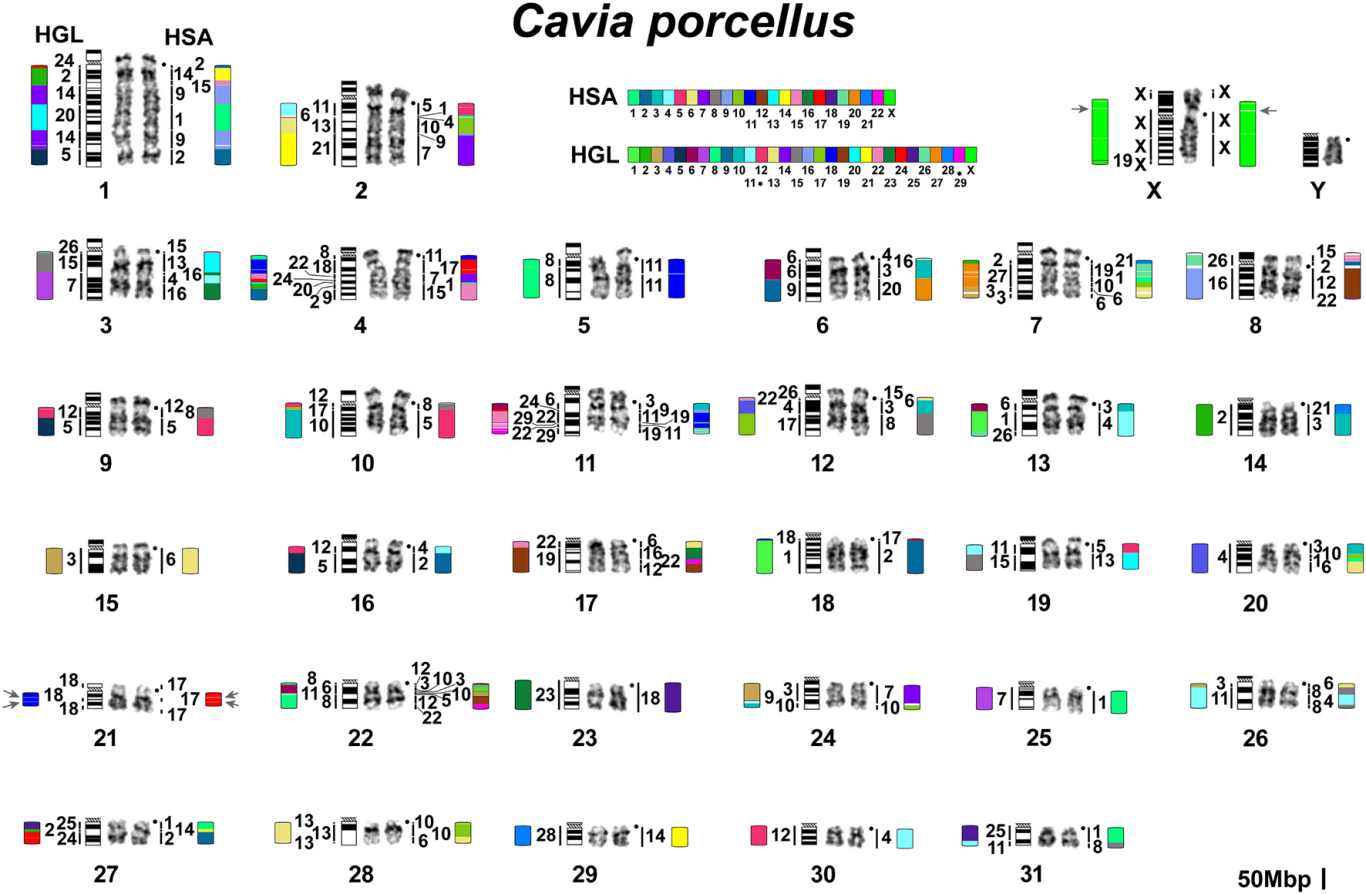
Idiogram and karyotype of the guinea pig (CPOR) with homologies to naked mole-rat (HGL) and human (HSA) chromosomes revealed by comparative analysis of chromosome painting data and chromosome-level assemblies. The idiogram and karyotype of the guinea pig correspond to those previously published^55^ with one correction: idiograms and pairs of chromosomes 27 and 29 were exchanged. Color-schemes visualize Hi-C chromosome-level assembly data with 5 Mbp threshold. Gray arrows mark heterochromatin regions not assembled by Hi-C. Black dots mark centromere positions. The scale bar is 50 Mb and refers to chromosome assembly colored blocks. The idiogram does not show inversions within syntenic blocks.

**Figure 5:**
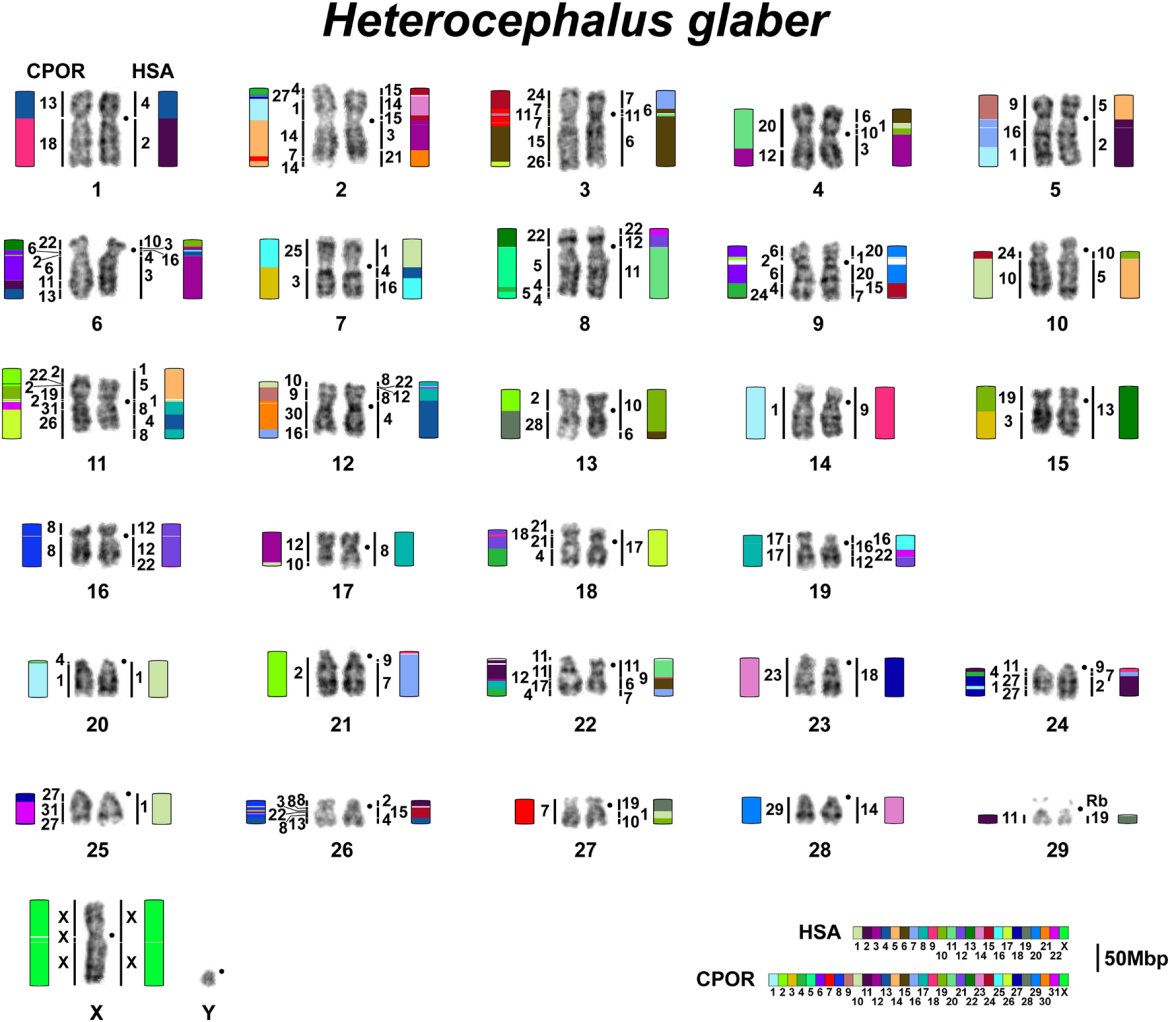
Naked mole-rat (HGL) karyotype with homologies to guinea pig (CPOR) and human (HSA) revealed by painting (vertical chromosome homology lines) and chromosome-level assemblies (visualized by color-schemes with 5 Mbp threshold). Black dots mark centromere positions. Scale bar 50 Mbp and refers to chromosome assembly colored blocks. The idiogram does not show inversions within syntenic blocks.

**Figure 6:**
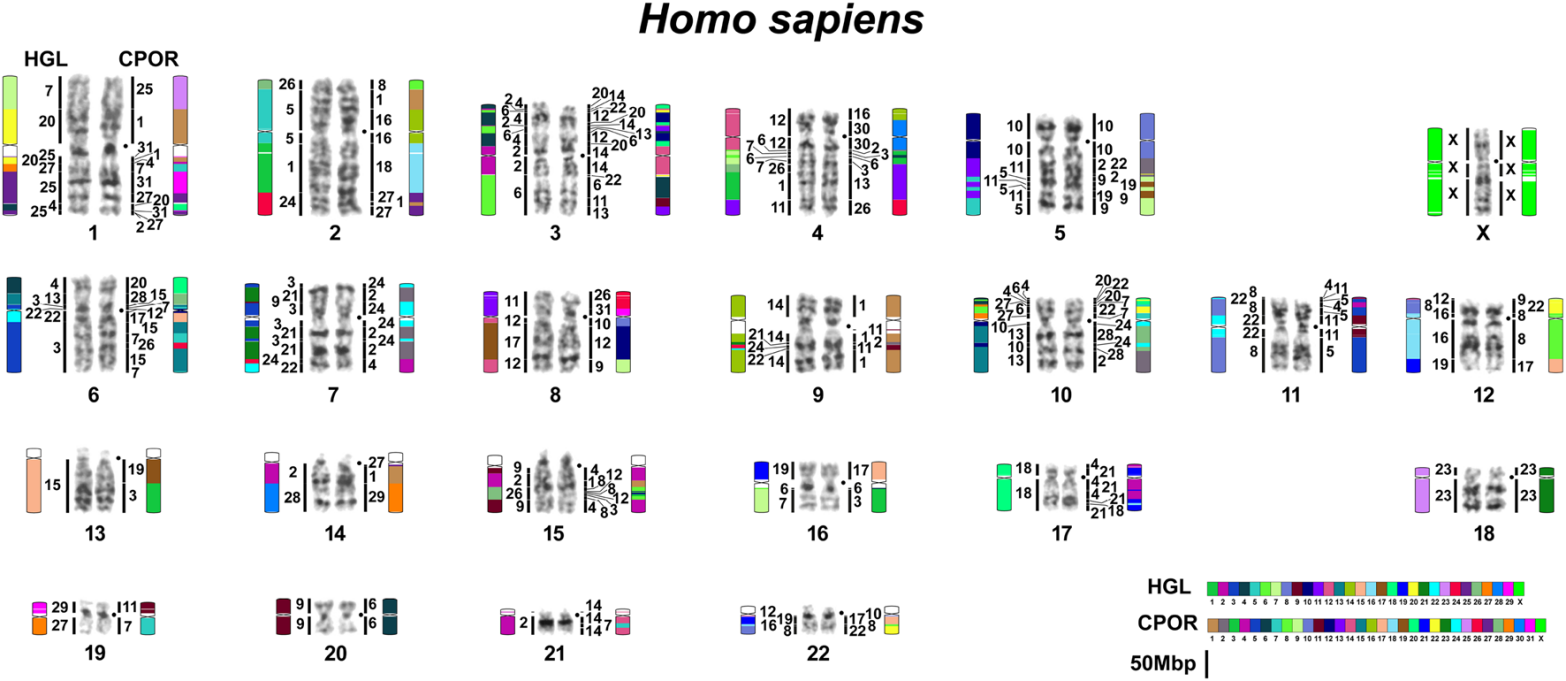
Human karyotype with updated homologies to guinea pig (CPOR) and naked mole-rat (HGL) revealed by painting and chromosome-level assemblies. Color-schemes visualize Hi-C chromosome-level assembly data with 5 Mbp threshold. Black dots mark centromere positions. Scale bar 50 Mbp and refers to chromosome assembly colored blocks. The idiogram does not show inversions within syntenic blocks.

By conducting repeated comparisons of syntenic blocks and painting data, we set the size of the Hi-C-derived syntenic blocks to be over 1 Mbp. This 1 Mbp threshold helped to filter out noise from small rearrangements that may be attributed to either the assembly artifacts or be the result of matching repeat sequences or alignment artifacts (Fig. 3a). 1 Mbp cutoff of syntenic blocks provides a satisfactory match of chromosome painting data and comparative Hi-C-derived synteny data. It is possible that smaller rearrangements (between syntenies less than 1 Mbp): fission, fusion, and inversion events may have occurred in evolution and will require higher-quality genomes constructed with long reads to be confirmed and distinguished from assembly artifacts. High-quality human assembly allowed us to visualize the centromeres on the synteny comparisons (Supplementary Fig. S5). The syntenic blocks and exact genome coordinates were extracted and presented in a table form (see Supplementary Tables S4–S6). Further, we merged smaller syntenies into larger blocks (at least 5 Mbp) for visualization alongside the painting data. By raising the threshold to 5 Mbp, we further minimize noise from small inversions frequently occurring within large syntenic blocks (Fig. 3b). We utilized the Hi-C chromosome-level assembly-based comparative synteny data to juxtapose and orient the Hi-C-derived syntenic segments exceeding 5Mbp along the chromosomes in the painting-mapped karyotypes (Figs. 4, 5, 6).

### The number of autosomal syntenic fragments

The number of autosomal syntenic fragments corresponds to a number of syntenic blocks revealed by chromosome-specific probes from another species. In whole-genome comparisons, this number of syntenies will depend on the threshold set for the syntenic block. In the Supplementary Table S7 we summarized the data about the number of autosomal syntenic fragments in *C. porcellus* and *H. glaber* genomes revealed by different approaches (Supplementary Table S7). The increased number of autosomal syntenic fragments at 1 Mbp cutoff may be related to the assembly artifacts and we anticipate that this number may decrease significantly with improved future assemblies.

## Discussion

### *H*.* glaber* karyotype

All previously published data showed the stability of diploid chromosome numbers (2n = 60) in the naked mole-rat karyotype. At the same time, the value of the fundamental number (number of chromosome arms, NF) varies greatly: NF = 120^76^, NF = 82^77^, and NF = 100^63^. According to the latest data, the naked mole-rat karyotype consists of 19 biarmed and 10 autosomal pairs represented by acrocentrics and medium and small subtelocentrics, NF = 100^78^, consistent with the data obtained here (Fig. 1a). The differences in reported NF counts can be attributed to whether the p-arms of acrocentric (subtelocentric) chromosomes were or were not taken into account, rather than to the karyotype variability.

Small variations in the length of the short chromosomal arms (e.g. pairs No. 19, 22, 26, and 27) that we observed (Fig. 1a) may be caused by different amounts of repeated sequences. Even though CBG-banding indicates a low accumulation of constitutive heterochromatin in the pericentromeric regions of chromosomes, uneven staining intensity of chromosome arms, and the presence of clearly visible gray blocks may indicate saturation of these areas of the naked mole-rat genome with repeated sequences (Fig. 1b). Further detailed analysis of the structure and distribution of repeated sequences in the naked mole-rat genome would be of interest.

Comparison of GTG-banded chromosomes showed some differences between the karyotype presented here and the karyotype presented in Deuve et al.^63^. Since there is no other data on the differential staining of the naked mole-rat karyotype, we can only assume that this represents population-specific difference or differences between cell lines that cannot be detected using conventional chromosome staining. It is also possible that the karyotypic variations led to differences between the flow karyotypes in our Fig. 1c and in previously published work^63^.

### Comparative chromosome-level genome analysis. Combination of FISH and Hi-C data

During the primary molecular cytogenetic comparison of *H. glaber, C. porcellus,* and human karyotypes, comparative cross-species chromosome maps showed gaps. Also, some chromosomes in mixed painting probes present in the libraries of *H. glaber* (Fig. 1c) and *C. porcellus*^55^ could not be assigned unambiguously, presumably due to homologue heteromorphisms present in different flow peaks. Also, there was unexplained difficulty in localizing *H. glaber* chromosome-specific probes to human chromosomes by in situ hybridization. All these limitations of heterologous chromosome painting on phylogenetically distant species led us here to rely on the chromosome-level assembly data to obtain complete comparative maps. At the same time, the orientation of scaffolds relative to the centromere could only be performed by linking bioinformatic data with cytogenetic data; this also applies to establishing the length of the gaps for heterochromatin regions. However, comparison of bioinformatic and cytogenetic data allowed us to link all chromosome-scaffolds to individual cytogenetically characterized chromosomes (Supplementary Tables S4–S6). The result of this work was the construction of complete comparative chromosome maps of two hystricomorph species and human (Figs. 4, 5, 6).

The approach of complementing chromosome painting and chromosome-level assemblies improved the genome assemblies of guinea pig and naked mole-rat and yielded a complete comparative cross-species chromosome map with a human genome.

The analysis of chromosome-level assemblies allowed the identification of additional fine rearrangements in a previously published guinea pig and human comparative map^55^. The refinement is mainly related to the detection of additional fragments, the size of which is below the resolution level of chromosome painting (Figs. 4, 6). However, in some cases, such as the localization of HSA 2 in the centromeric region of CPOR 1, we were able to confirm bioinformatic data using FISH (Fig. 2).

Bioinformatic analysis has limitations when assembling regions rich in repeated sequences. At the same time, such areas are clearly visible in cytogenetic analysis. The naked mole-rat genome does not contain large blocks of repeats that would make assembly difficult. On the contrary, the guinea pig genome is characterized by large heterochromatic blocks that led to the “collapsing” of individual scaffolds during bioinformatic analysis of the genome of this species. The most striking examples are chromosomes CPOR 21 and CPOR X, on which homologous blocks of human and naked mole-rat chromosomes are separated by large blocks of heterochromatin according to CBG-banding and FISH data (Figs. 1, 2, 4).

The number of autosomal conserved segments identified in the genomes of various species, during a comparative analysis of their chromosome sets, indirectly indicates the evolutionary conservation of a whole chromosomal complement of a particular species. This value varies significantly for species from different branches of the phylogenetic tree. Among rodents, comparisons with the human genome have been made for a small number of species, showing higher rearrangement rates in myomorphs and hystricomorphs compared to other rodents^55,79^. Of course, due to different resolutions, the method of comparative chromosome painting reveals fewer conserved segments than, for example, comparisons of complete genome sequences. For example, in guinea pig, 100 autosomal syntenic segments were identified by human genomic sequences with a 5 Mbp cutoff and 536 with a 1 Mbp cutoff. The number of inversions per individual chromosome (the chromosome length and the number of syntenic blocks within it may be taken into account) is another relative measure of the level of evolutionary rearrangements activity (in guinea pig the most rearranged chromosome is CPOR 4) (Supplementary Figs. S3–S6). Here the data analysis revealed a significant number of conserved segments in the genomes of both rodent species relative to human, but the naked mole-rat karyotype is characterized by the conservation of larger fragments homologous to human chromosomes (Supplementary Table S7).

### Analysis of ancestral syntenies in *H*. *glaber* and *C*. *porcellus* karyotypes

Questions about the structure of the ancestral karyotype for a taxon of interest have been discussed since the dawn of cytogenetics. Understanding chromosome structure provides opportunities for studying the evolution of the genome at many different levels. The development of comparative methods of genome analysis leads to the reevaluation of previously described ancestral karyotypes. The combination of comparative chromosome painting and Hi-C genome assemblies provides more complete and accurate information since it accounts for differences in resolution of the methods and bridges limitations of each method^12^.

Here we analyzed the syntenic associations of human chromosomes that have been shown in earlier work to reveal putative ancestral eutherian karyotypes and consider the presence of such syntenies in the genomes of two hystricomorphs. The presence of any synteny in the genomes of both *H. glaber* and *C. porcellus* may indicate the putative ancestral status of these syntenies for the hystricomorphs. The ancestral eutherian syntenies (HSA 3/21, 4/8, 12/22, 14/15)^80^ were found in karyotypes of both *H. glaber* and *C. porcellus* (Figs. 4, 5, Supplementary Fig. S6). In both species, an inversion occurred, which transformed the ancestral eutherian synteny HSA 4/8 to the synteny HSA 8/4/8 in CPOR 26 and HGL 11 (Figs. 4, 5). Perhaps this inversion should be considered ancestral to the hystricomorphs, but a larger number of species of the suborder is still needed to confirm this. The synteny HSA 4/8 found in HGL 12 represents a derived state.

We found three and four syntenies HSA 12/22 in the karyotypes of *C. porcellus* and *H. glaber*, respectively (Figs. 4, 5, Supplementary Fig. S6). A comparison of human genomic coordinates showed that the synteny on CPOR 8 corresponds to the synteny on HGL 16, the synteny on CPOR 17 corresponds to the synteny on HGL 19, and the synteny on CPOR 22 corresponds to the synteny on HGL 8 (Supplementary Table S6). The synteny HSA 12/22 on HGL 12 is specific for naked mole-rat. In the genome of *C. porcellus* the syntenies HSA 14/15 are formed by smaller fragments of the human chromosomes than in the genome of *H. glaber*, which also had an inversion HSA 15/14/15 (Figs. 4, 5, Supplementary Fig. S3, Supplementary Table S6).

Two more ancestral eutherian syntenic associations (HSA 7/16 and 16/19) perhaps were lost during the evolution of Hystricomorpha, as they were not found either in the present work or in previous studies on these rodents^43,55^.

The human synteny HSA 9/11 defined as specific for Rodentia and Glires (the group combining Rodentia and Lagomorpha)^81^ was not previously detected in the *C. porcellus* karyotype by FISH^55^, or in the *H. glaber* genome assembly^43^. Current data show that HSA 9/11 is present in the genomes of both species (CPOR 11 and HGL 22) (Figs. 4, 5, Supplementary Fig. S6).

The ancestral status for this group also involves HSA 1/10, which was previously found in *H. glaber* karyotype^43^ and in the *C. porcellus* genome here (Fig. 4). In total, two HSA 1/10 syntenies were identified in the *C. porcellus* genome, with the ancestral one corresponding to that found on chromosome CPOR 20 and homologous to HSA 1/10 on HGL 4 (Figs. 4, 5, Supplementary Fig. S6, Supplementary Table S6).

The synteny HSA 3/19 considered as putative ancestral for Rodentia and some rodent suborders^55,81,82^ was not confirmed earlier in the *H. glaber* genome^43^, nor in *H. glaber* and *C. porcellus* in this work. It is possible that HSA 3/19 was lost solely in the hystricomorph phylogenetic lineage since it is found in representatives of other rodent suborders^81,83,84^.

From two syntenies apomorphic to rodents^81^, HSA 8/12 was revealed in both hystricomorphs, but HSA 15/20 is characteristic for *H. glaber* genome only (Figs. 4, 5). The HSA 8/12 is formed by a larger HSA 12 on HGL 12, than on CPOR 9 (Supplementary Table S6). It is noted that HSA 8/12 was not identified in previous work by Zhou et al.^43^. It is emphasized that only some parts of the data on the analysis of human chromosome associations in the *H. glaber* karyotype published earlier^43^ find confirmation in our work. Some differences can be explained by different thresholds set for conserved blocks in these studies, but some contradictions could be caused by misorientation of superscaffolds and the formation of hybrid superscaffolds in the assembly presented in Zhou et al.^43^ (Supplementary Table S8). Both studies show that an inversion took place in HGL 2 that led to the appearance of the HSA 15/14/15/3/21 association (Figs. 2, 5, Supplementary Table S8). Chromosome HGL 3 is homologous to not only HSA 6, but HSA 7/6/11/6 (Fig. 5, Supplementary Table S8). Chromosome HGL 9 is homologous to HSA 20/1/20/15/20, not just the HSA 15/20. Homology to HSA 1/5/1/8/4/8 is characteristic of HGL 11 (Figs. 2, 5). A similar association was previously identified, but did not include fragments of human chromosomes 1^43^ (see Supplementary Table S7 and comments there). Our data also clearly show that HSA 17 is homologous to a single HGL 18 chromosome and HGL 27 is homologous to HSA 19/1/10 (Fig. 5). We did not reveal a synteny HSA 10/1/19/17 as described previously^43^ (Supplementary Table S8 and comments there).

In addition to the above associations, we find other common associations in the genomes of *C. porcellus* and *H. glaber*: the ancestral eutherian synteny HSA 1/10 is included in the bigger synteny HSA 6/1/10/3 on HGL 4 and CPOR 20. In this case, the size of HSA 3 and HSA 6 is somewhat different, although both segments contain homologous fragments in both species (Figs. 4, 5, Supplementary Table S6). The ancestral eutherian synteny HSA 12/22 forms an association with HSA 16 on CPOR 17 and HGL 19. Fragment size of HSA 16 is similar in both species. Moreover, both *C. porcellus* and *H. glaber* have HSA 4/16 (CPOR 3, HGL 7) and HSA 6/10 (CPOR 18, HGL 13) associations in their genomes (Figs. 4, 5, Supplementary Table S6). It can be assumed that all these associations are ancestral to Hystricomorpha.

So we can assume that the synteny HSA 1/10 (possibly as part of HSA 6/1/10/3), 3/21, 4/16, 6/10, 8/4/8, 8/12, 9/11, 12/22 (three, one in the HSA 16/22/12), and 14/15 could be ancestral for hystricomorphs ancestral karyotype, but a detailed comparison of the genomes of other members of the order with the human is necessary to confirm or refute this assumption.

## Conclusion

At first, the possibility of fast and inexpensive genome sequencing led to the emergence of many assemblies that were not verified by other methods. Their number continues to grow to this day. Even these data have already made it possible to use model organisms more effectively, including the guinea pig and the naked mole-rat, for a clearer understanding of mole-rat evolutionary history and for suggesting molecular pathways that may explain the extraordinarily longevity and unique health traits of this species^41,45,85–87^. Later, comparison of sequence data with cytogenetic data reveals the imperfection of exclusively bioinformatic approaches in chromosome-level assembly^12^. Currently, the chromosome-level genome assembly is the gold standard that all international consortiums for genomic projects strive for^13^. Our data once again point to the extreme importance of verifying bioinformatic data by other methods. Obtaining chromosome-level assemblies for the naked mole-rat and guinea pig, building complete comparative chromosome maps for both species and humans, opens new prospects for using these species as models for studying animal and human disease, as well as for studying genomic evolution and comparative genomics.

### Supplementary Information

Supplementary Information.

## Data Availability

The datasets analysed during the current study are available in the DNA Zoo Consortium website, https://www.dnazoo.org/assemblies/Cavia_porcellus and https://www.dnazoo.org/assemblies/Heterocephalus_glaber. For human, we used reference genome assembly version GRCh38.p13 available in the National Center for Biotechnology Information repository, https://www.ncbi.nlm.nih.gov/assembly/GCF_000001405.39/.

## References

[1] King M (1993). Species Evolution: The Role of Chromosome Change.

[2] Robinson TJ, Ruiz-Herrera A, Avise JC (2008). Hemiplasy and homoplasy in the karyotypic phylogenies of mammals. Proc. Natl. Acad. Sci..

[3] Graphodatsky AS, Trifonov VA, Stanyon R (2011). The genome diversity and karyotype evolution of mammals. Mol. Cytogenet..

[4] Deakin JE, Ezaz T (2014). Tracing the evolution of amniote chromosomes. Chromosoma.

[5] Bernardi G (2015). Chromosome architecture and genome organization. PLoS One.

[6] Mayrose I, Lysak MA (2021). The evolution of chromosome numbers: Mechanistic models and experimental approaches. Genome Biol. Evol..

[7] Peichel CL (2017). Chromosome evolution: Molecular mechanisms and evolutionary consequences. J. Hered..

[8] Burgin CJ, Colella JP, Kahn PL, Upham NS (2018). How many species of mammals are there?. J. Mammal..

[9] Graphodatsky AS (2020). Atlas of Mammalian Chromosomes.

[10] Damas J, Corbo M, Lewin HA (2021). Vertebrate chromosome evolution. Annu. Rev. Anim. Biosci..

[11] Iannucci A (2021). Bridging the gap between vertebrate cytogenetics and genomics with single-chromosome sequencing (ChromSeq). Genes.

[12] Deakin JE (2019). Chromosomics: Bridging the gap between genomes and chromosomes. Genes.

[13] A reference standard for genome biology. *Nat. Biotechnol.* **36**, 1121–1121 (2018). 10.1038/nbt.4318 30520871

[14] Kim J (2013). Reference-assisted chromosome assembly. Proc. Natl. Acad. Sci..

[15] Rhie A (2021). Towards complete and error-free genome assemblies of all vertebrate species. Nature.

[16] Nurk S (2022). The complete sequence of a human genome. Science.

[17] Garner BA (2016). Genomics in conservation: Case studies and bridging the gap between data and application. Trends Ecol. Evol..

[18] Worley KC (2017). A golden goat genome. Nat. Genet..

[19] Supple MA, Shapiro B (2018). Conservation of biodiversity in the genomics era. Genome Biol..

[20] Smith JJ (2018). The sea lamprey germline genome provides insights into programmed genome rearrangement and vertebrate evolution. Nat. Genet..

[21] Marlétaz F (2018). Amphioxus functional genomics and the origins of vertebrate gene regulation. Nature.

[22] Lieberman-Aiden E (2009). Comprehensive mapping of long-range interactions reveals folding principles of the human genome. Science.

[23] van Berkum NL (2010). Hi-C: A method to study the three-dimensional architecture of genomes. J. Vis. Exp..

[24] Burton JN (2013). Chromosome-scale scaffolding of de novo genome assemblies based on chromatin interactions. Nat. Biotechnol..

[25] Dudchenko O (2017). De novo assembly of the *Aedes aegypti* genome using Hi-C yields chromosome-length scaffolds. Science.

[26] Hills M (2021). Construction of whole genomes from scaffolds using single cell strand-seq data. Int. J. Mol. Sci..

[27] Poisson W (2023). Chromosome-level assembly of the Rangifer tarandus genome and validation of cervid and bovid evolution insights. BMC Genom..

[28] Zimin AV (2012). Mis-assembled “segmental duplications” in two versions of the *Bos*
*taurus* genome. PLoS One.

[29] Luo J (2021). A comprehensive review of scaffolding methods in genome assembly. Brief. Bioinform..

[30] Graphodatsky AS (2007). Comparative chromosomics. Mol. Biol..

[31] Lewin HA, Graves JAM, Ryder OA, Graphodatsky AS, O’Brien SJ (2019). Precision nomenclature for the new genomics. GigaScience.

[32] O’Connor RE (2018). Chromosome-level assembly reveals extensive rearrangement in saker falcon and budgerigar, but not ostrich, genomes. Genome Biol..

[33] Shearer LA (2014). Fluorescence in situ hybridization and optical mapping to correct scaffold arrangement in the tomato genome. G3: Genes Genomes Genet..

[34] Chamala S (2013). Assembly and validation of the genome of the nonmodel basal angiosperm *Amborella*. Science.

[35] Sherman PW (1991). The Biology of the Naked Mole-Rat.

[36] Buffenstein R (2008). Negligible senescence in the longest living rodent, the naked mole-rat: Insights from a successfully aging species. J. Comp. Physiol. B.

[37] Tian X (2013). High-molecular-mass hyaluronan mediates the cancer resistance of the naked mole rat. Nature.

[38] Seluanov A (2009). Hypersensitivity to contact inhibition provides a clue to cancer resistance of naked mole-rat. Proc. Natl. Acad. Sci..

[39] Liang S, Mele J, Wu Y, Buffenstein R, Hornsby PJ (2010). Resistance to experimental tumorigenesis in cells of a long-lived mammal, the naked mole-rat (*Heterocephalus*
*glaber*). Aging Cell.

[40] Edrey YH, Hanes M, Pinto M, Mele J, Buffenstein R (2011). Successful aging and sustained good health in the naked mole rat: A long-lived mammalian model for biogerontology and biomedical research. ILAR J..

[41] Lewis KN (2016). Unraveling the message: Insights into comparative genomics of the naked mole-rat. Mamm. Genome.

[42] Holtze S (2021). Alternative animal models of aging research. Front. Mol. Biosci..

[43] Zhou X (2020). Beaver and naked mole rat genomes reveal common paths to longevity. Cell Rep..

[44] Kim EB (2011). Genome sequencing reveals insights into physiology and longevity of the naked mole rat. Nature.

[45] Keane M (2014). The Naked Mole Rat Genome Resource: Facilitating analyses of cancer and longevity-related adaptations. Bioinformatics.

[46] Fang X (2014). Adaptations to a subterranean environment and longevity revealed by the analysis of mole rat genomes. Cell Rep..

[47] Padilla-Carlin DJ, McMurray DN, Hickey AJ (2008). The guinea pig as a model of infectious diseases. Comp. Med..

[48] Hook LM, Friedman HM, Awasthi S (2021). Guinea pig and mouse models for genital herpes infection. Curr. Protoc..

[49] Adner M (2020). Back to the future: Re-establishing guinea pig in vivo asthma models. Clin. Sci..

[50] Baeten LA (2018). Standardized guinea pig model for Q fever vaccine reactogenicity. PLoS One.

[51] Podell BK (2017). A model of type 2 diabetes in the guinea pig using sequential diet-induced glucose intolerance and streptozotocin treatment. Dis. Models Mech..

[52] Sánchez-Macías D, Barba-Maggi L, Morales-delaNuez A, Palmay-Paredes J (2018). Guinea pig for meat production: A systematic review of factors affecting the production, carcass and meat quality. Meat Sci..

[53] Lindblad-Toh K (2011). A high-resolution map of human evolutionary constraint using 29 mammals. Nature.

[54] Weyrich A (2014). Whole genome sequencing and methylome analysis of the wild guinea pig. BMC Genom..

[55] Romanenko SA (2015). A first generation comparative chromosome map between guinea pig (*Cavia*
*porcellus*) and humans. PLoS One.

[56] Tian X (2015). *INK4* locus of the tumor-resistant rodent, the naked mole rat, expresses a functional p15/p16 hybrid isoform. Proc. Natl. Acad. Sci..

[57] Graphodatsky AS, Radjabli SI (1988). Chromosomes of Agricultural and Laboratory Mammals.

[58] Stanyon R, Galleni L (1991). A rapid fibroblast culture technique for high resolution karyotypes. Boll. Zool..

[59] Seabright M (1971). A rapid banding technique for human chromosomes. Lancet.

[60] Sumner AT (1972). A simple technique for demonstrating centromeric heterochromatin. Exp. Cell Res..

[61] Lemskaya NA (2018). A combined banding method that allows the reliable identification of chromosomes as well as differentiation of AT- and GC-rich heterochromatin. Chromosome Res..

[62] Ferguson-Smith MA (1997). Genetic analysis by chromosome sorting and painting: Phylogenetic and diagnostic applications. Eur. J. Hum. Genet. EJHG.

[63] Deuve JL (2006). Complex evolution of X and Y autosomal translocations in the giant mole-rat, *Cryptomys*
*mechowi* (Bathyergidae). Chromosome Res..

[64] Ijdo JW, Wells RA, Baldini A, Reeders ST (1991). Improved telomere detection using a telomere repeat probe (TTAGGG)n generated by PCR. Nucleic Acids Res..

[65] Maden BEH (1987). Clones of human ribosomal DNA containing the complete 18 S-rRNA and 28 S-rRNA genes. Characterization, a detailed map of the human ribosomal transcription unit and diversity among clones. Biochem. J..

[66] Yang F (1999). A complete comparative chromosome map for the dog, red fox, and human and its integration with canine genetic maps. Genomics.

[67] Smit, A. F. A., Hubley, R. & Green, P. RepeatMasker Open-4.0. (2013-2015). http://www.repeatmasker.org.

[68] Benson G (1999). Tandem repeats finder: A program to analyze DNA sequences. Nucleic Acids Res..

[69] Morgulis A, Gertz EM, Schäffer AA, Agarwala R (2006). WindowMasker: Window-based masker for sequenced genomes. Bioinformatics.

[70] Bao W, Kojima KK, Kohany O (2015). Repbase Update, a database of repetitive elements in eukaryotic genomes. Mobile DNA.

[71] Jurka J (2000). Repbase Update: A database and an electronic journal of repetitive elements. Trends Genet..

[72] Quinlan AR, Hall IM (2010). BEDTools: A flexible suite of utilities for comparing genomic features. Bioinformatics.

[73] Armstrong J (2020). Progressive Cactus is a multiple-genome aligner for the thousand-genome era. Nature.

[74] Paten B (2011). Cactus: Algorithms for genome multiple sequence alignment. Genome Res..

[75] Krasheninnikova K (2020). halSynteny: A fast, easy-to-use conserved synteny block construction method for multiple whole-genome alignments. GigaScience.

[76] George W (1979). Conservatism in the karyotypes of two African mole rats (Rodentia, Bathyergidae). Zeitschrift für Säugetierkunde.

[77] Capanna E, Merani MS (1980). Karyotypes of Somalian rodent populations. Monit. Zool. Ital. Suppl..

[78] Zemlemerova ED, Kostin DS, Lavrenchenko LA (2020). Chromosomal monomorphism in the naked mole-rat *Heterocephalus*
*glaber* (Rodentia: Heterocephalidae). Russ. J. Genet..

[79] Romanenko SA, Volobouev V (2012). Non-Sciuromorph rodent karyotypes in evolution. Cytogenet. Genome Res..

[80] Ferguson-Smith MA, Trifonov V (2007). Mammalian karyotype evolution. Nat. Rev. Genet..

[81] Graphodatsky AS (2008). Tracking genome organization in rodents by Zoo-FISH. Chromosome Res..

[82] Romanenko SA, Perelman PL, Trifonov VA, Graphodatsky AS (2012). Chromosomal evolution in Rodentia. Heredity.

[83] Froenicke L (2006). Are molecular cytogenetics and bioinformatics suggesting diverging models of ancestral mammalian genomes?. Genome Res..

[84] Murphy WJ (2005). Evolution: Dynamics of mammalian chromosome evolution inferred from multispecies comparative maps. Science.

[85] Boshra H, Zelek WM, Hughes TR, Rodriguez de Cordoba S, Morgan BP (2018). Absence of CD59 in guinea pigs: Analysis of the *Cavia*
*porcellus* genome suggests the evolution of a CD59 pseudogene. J. Immunol..

[86] Wu H (2018). Scleral hypoxia is a target for myopia control. Proc. Natl. Acad. Sci. USA.

[87] Delsuc F, Tilak MK (2015). Naked but not hairless: The pitfalls of analyses of molecular adaptation based on few genome sequence comparisons. Genome Biol. Evol..

