# Supplementary material for: Integration of fluorescence in situ hybridization and chromosome-length genome assemblies revealed synteny map for guinea pig, naked mole-rat, and human
Source: Sci Rep. 2023 Nov 29;13:21055. doi: 10.1038/s41598-023-46595-x (PMC10687270; doi:10.1038/s41598-023-46595-x)
Supplement: Supplementary file 1 — Supplementary Information. [file 41598_2023_46595_MOESM1_ESM.pdf]

Integration of fluorescence *in situ* hybridization and chromosome-length genome assemblies revealed synteny map for guinea pig, naked mole-rat, and human

Svetlana A. Romanenko[1*], Sergei F. Kliver[2], Natalia A. Serdyukova[1], Polina L. Perelman[1], Vladimir A. Trifonov[1,3], Andrei Seluanov[4], Vera Gorbunova[4], Jorge Azpurua[5], Jorge C. Pereira[6,7], Malcolm A. Ferguson-Smith[7], Alexander S. Graphodatsky[1]

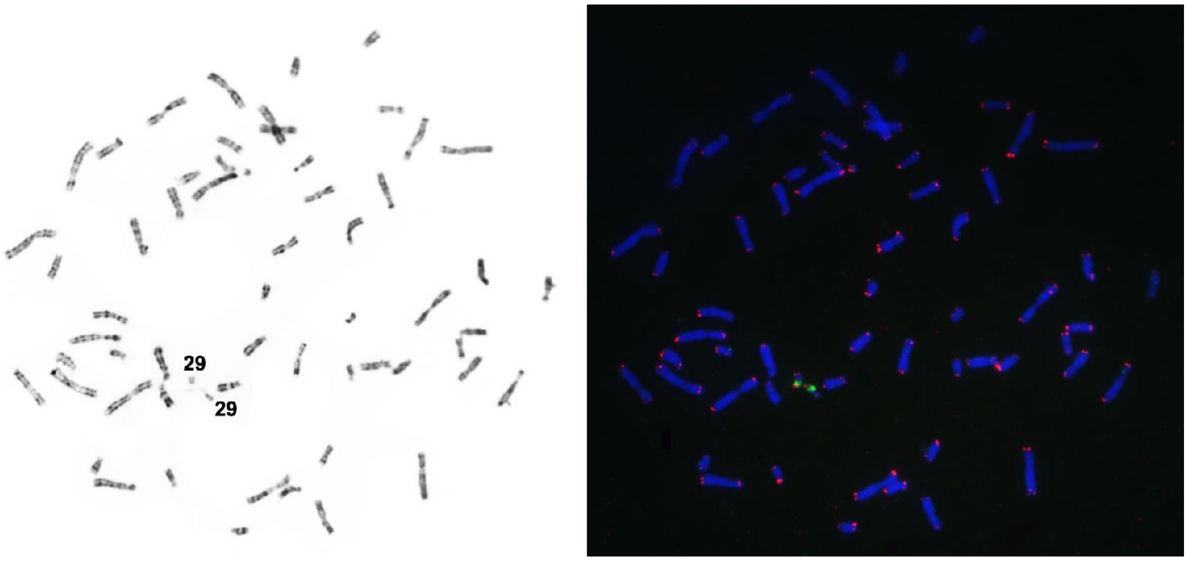

Supplementary Figure S1. Localization of 5.8S/18S/28S-rDNA (green) and telomeric repeat $(TTAGGG)_n$ (red) on *Heterocephalus glaber* GTG-banded metaphase chromosomes.

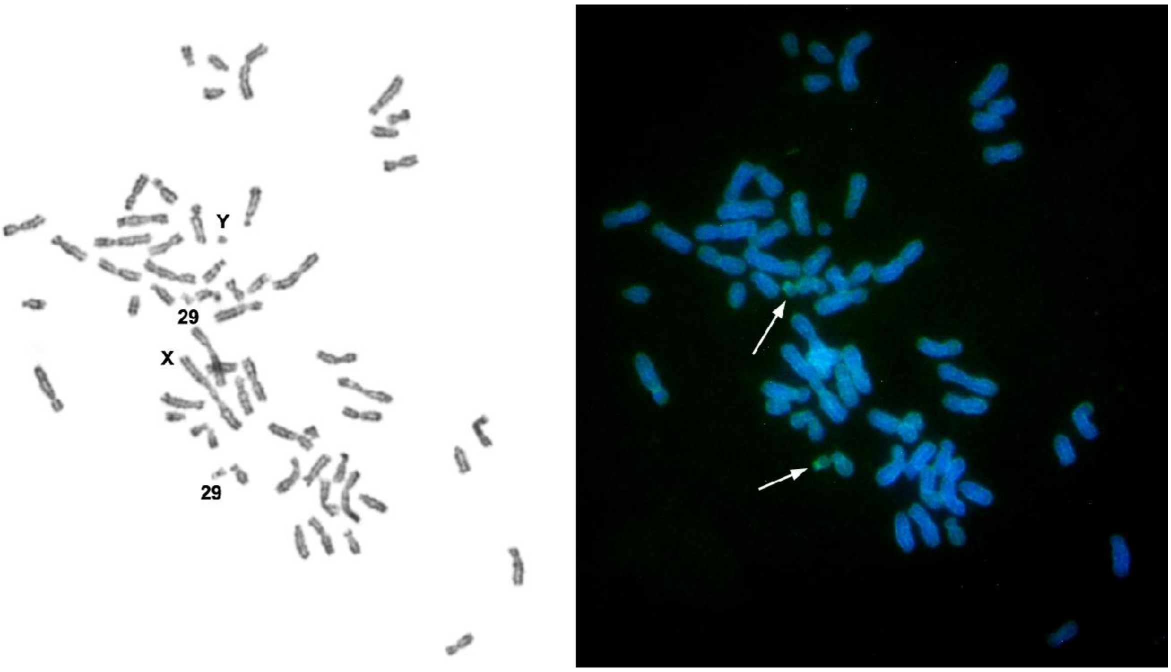

Supplementary Figure S2. CDAG-banding of *Heterocephalus glaber* GTG-banded metaphase chromosomes. White arrows mark the chromosome 29 carrying blocks of GC-rich heterochromatin (green).

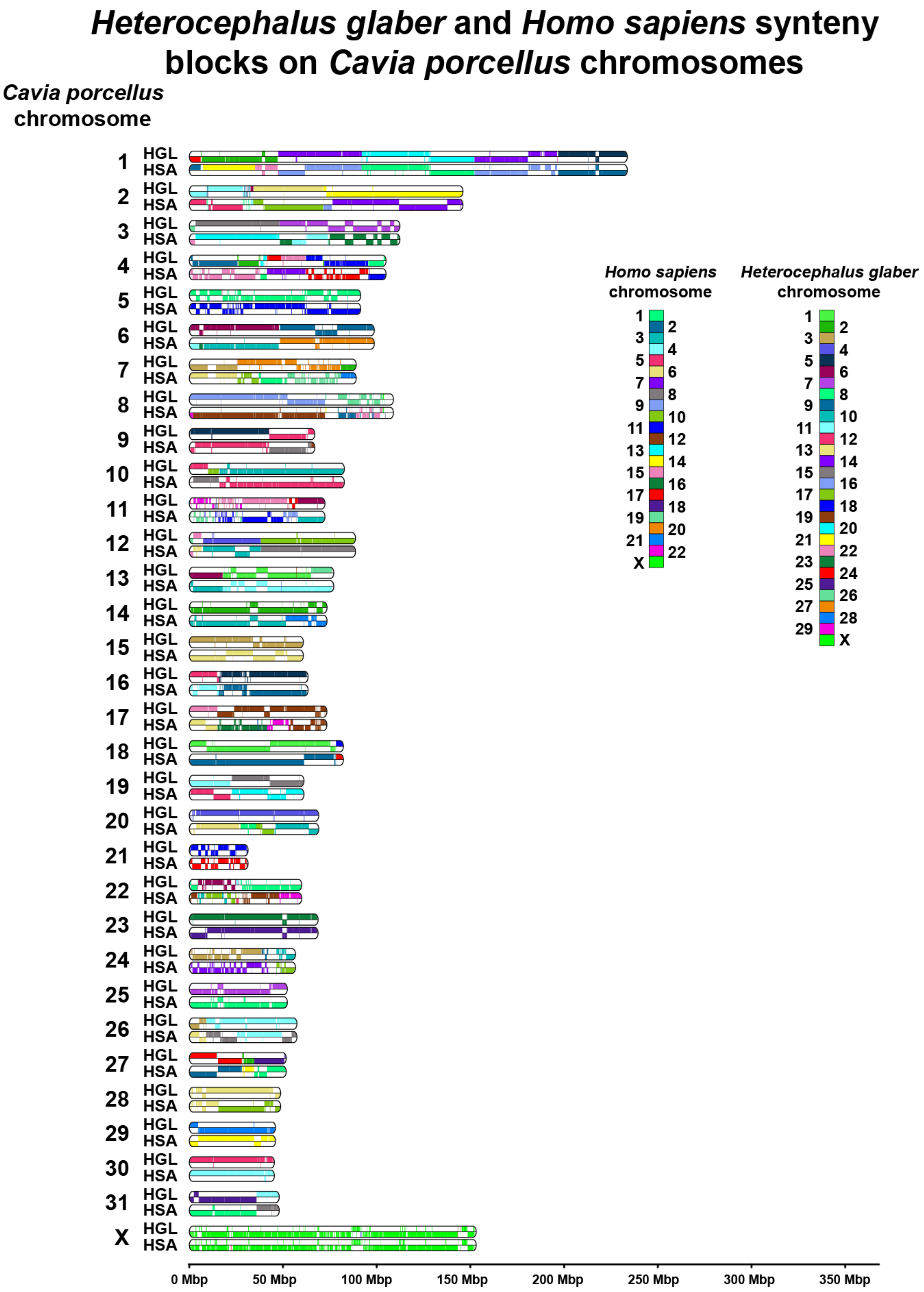

Supplementary Figure S3. Syntenic blocks identified in the guinea pig genome in chromosome pairwise comparison with the naked mole-rat (HGL) and human (HSA). Each chromosome is divided into an upper and lower track. If the orientation of the blocks is the same in the reference genome and the query genome, then the block is drawn on the upper track; if not, then on the lower one.

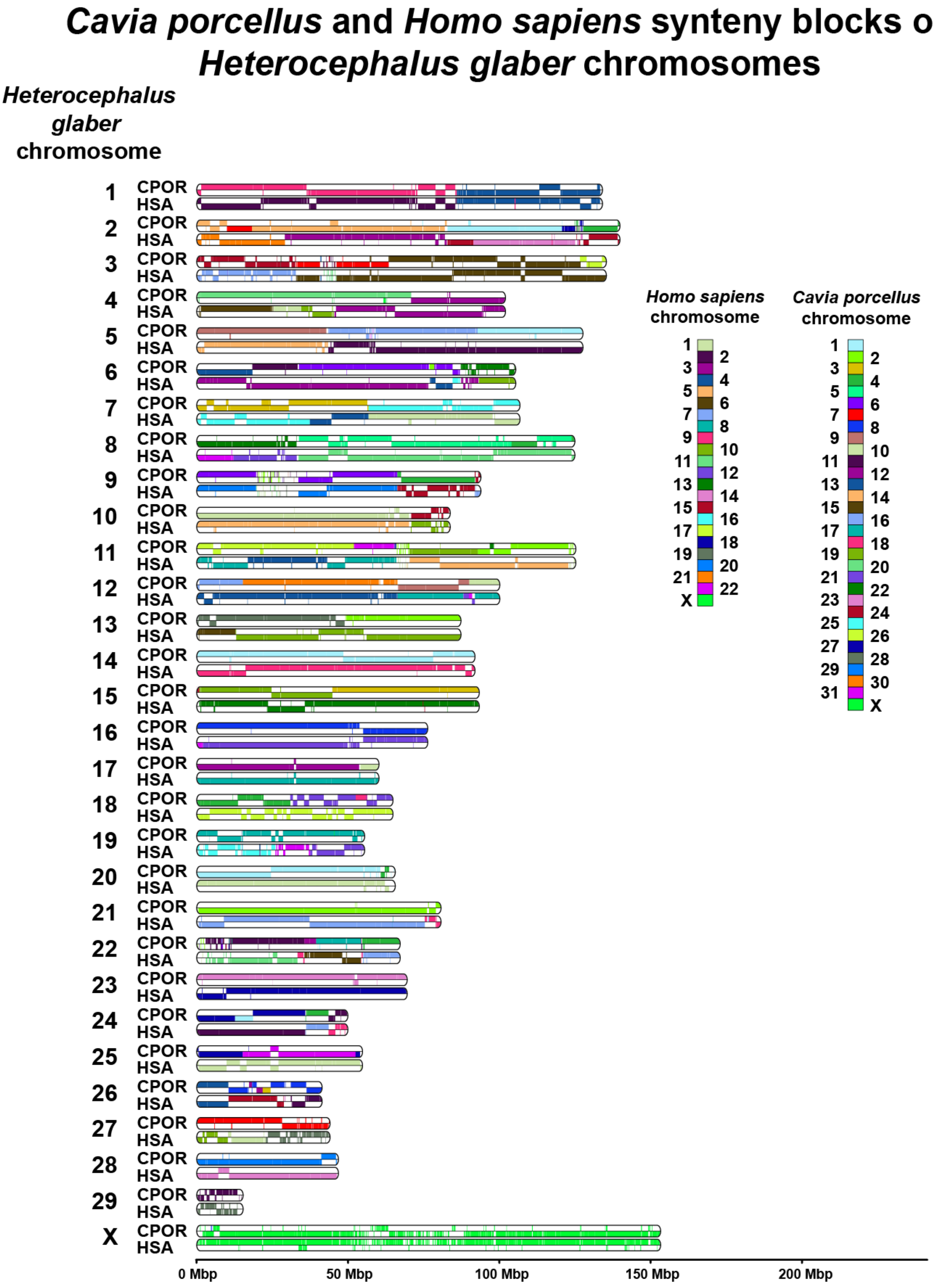

Supplementary Figure S4. Syntenic blocks identified in the naked mole-rat genome in chromosome pairwise comparison with the guinea pig (CPOR) and human (HSA). Each chromosome is divided into an upper and lower track. If the orientation of the blocks is the same in the reference genome and the query genome, then the block is drawn on the upper track; if not, then on the lower one.

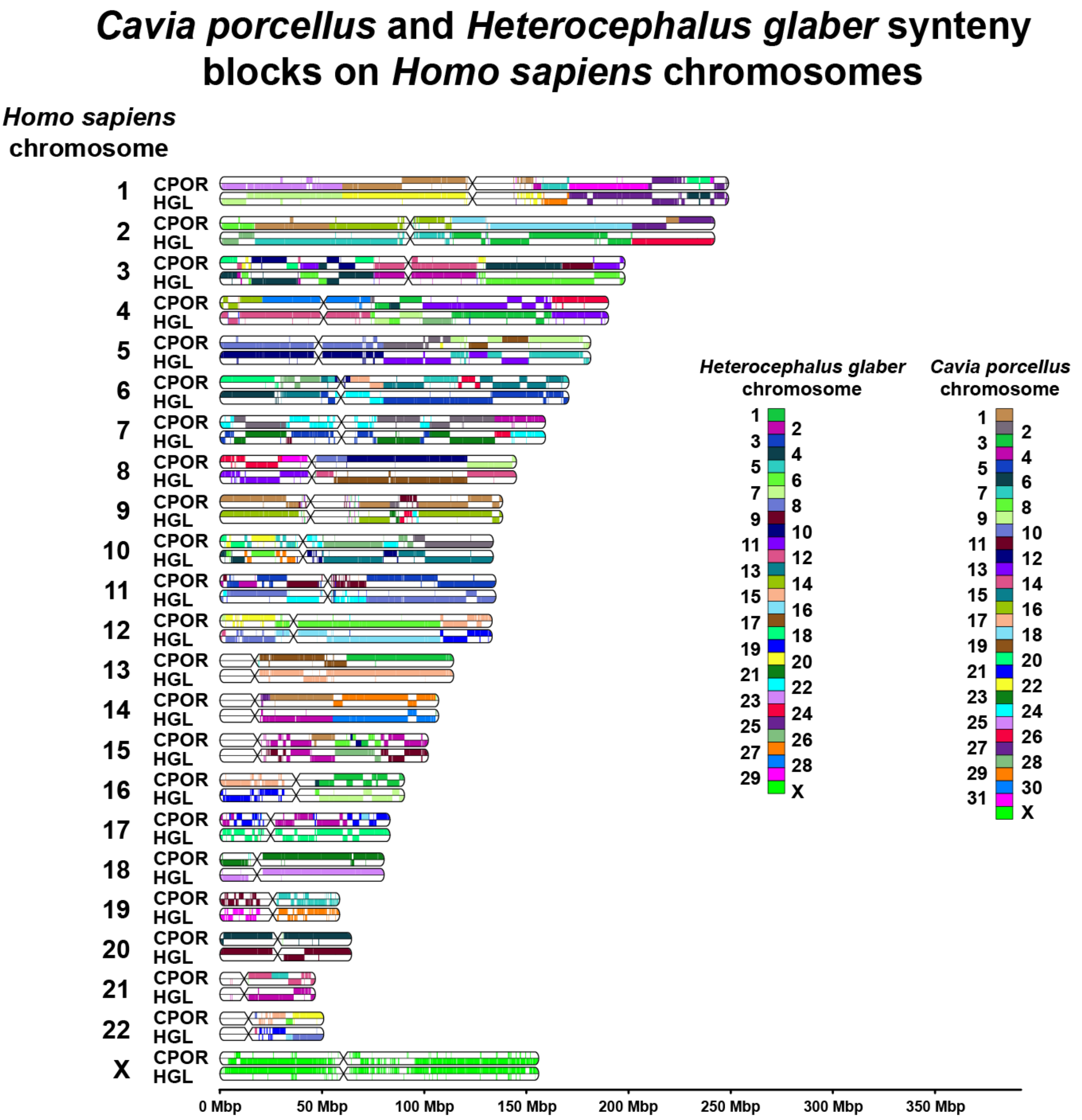

Supplementary Figure S5. Syntenic blocks identified in human genome in chromosome pairwise comparison with the guinea pig (CPOR) and naked mole-rat (HGL). Each chromosome is divided into an upper and lower track. If the orientation of the blocks is the same in the reference genome and the query genome, then the block is drawn on the upper track; if not, then on the lower one.

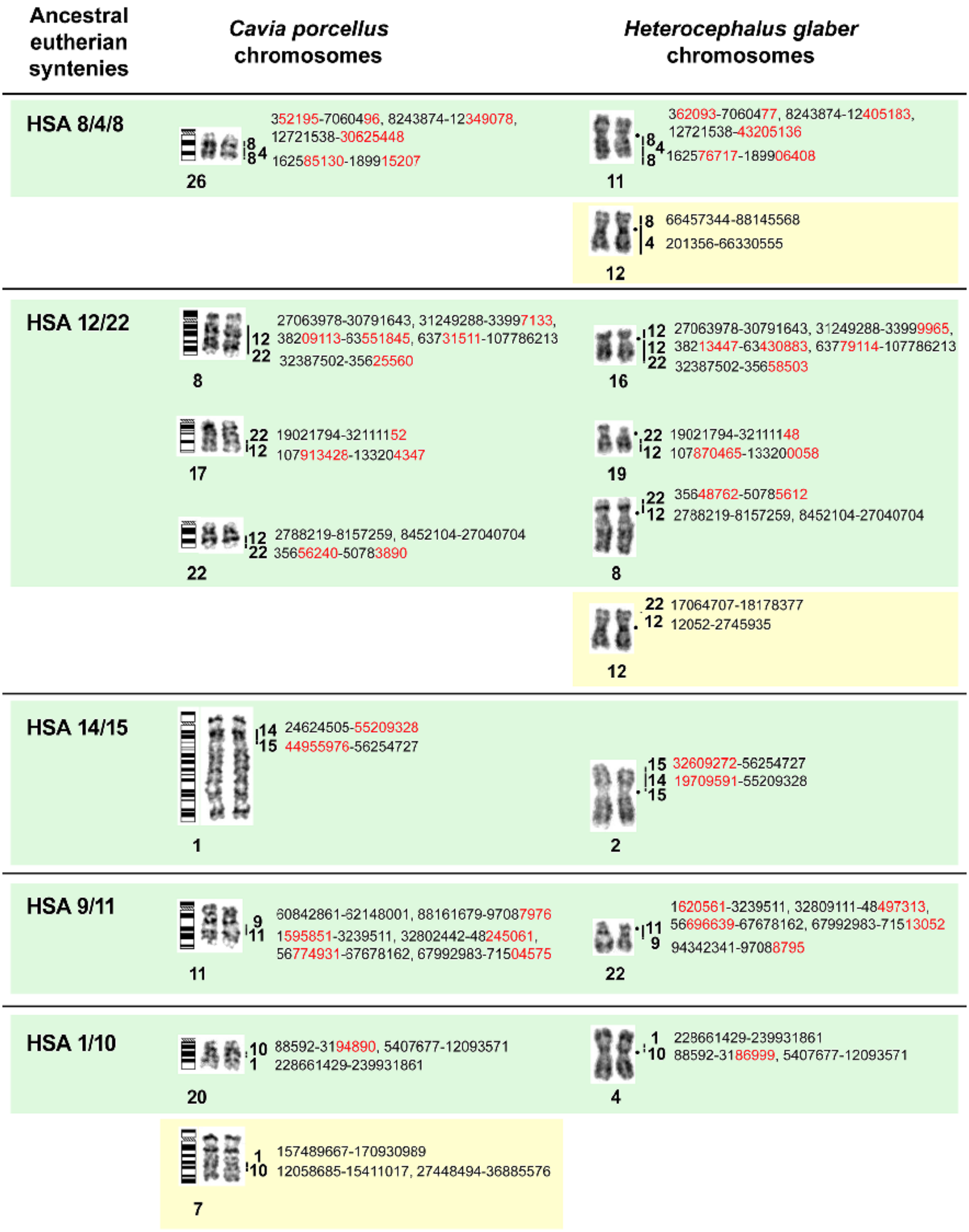

Supplementary Figure S6. Ancestral eutherian associations in *Cavia porcellus* and *Heterocephalus glaber* genomes. Coordinates in the human genome (HSA) are indicated for each segment on the right. Differences in the coordinates of the corresponding segments between the species are marked in red. Green blocks mark ancestral associations, yellow blocks highlight similar associations on other chromosomes that are not ancestral.

Supplementary Table S1 (part 1). Syntenic blocks with a minimum cut-off length of 50 kilobases in the reference guinea pig genome.

scaffold	query	start	end	query_start	query_end	strand	color	target_len	query_len	dist_upstream	dist_downstream	dist_end_start	dist_end_end	embedded
**CPOR 1**	HSA 2	36451	6006258	218435809	224710822	+	#036A9C	5969807	6275013		4488468			
	HSA 14	7349946	9836406	24608432	27002809	+	#FFFF00	2486460	2394377		7832			
	HSA 14	9844238	9931644	27003427	27094932	-	#FFFF00	87406	91505	7832	10298	7832	95238	false
	HSA 14	9941942	16097042	27100273	33858751	+	#FFFF00	6155100	6758478	10298	65981	10298	6165398	false
	HSA 14	16163023	20195893	33859567	38186011	+	#FFFF00	4032870	4326444	65981	103040	65981	4098851	false
	HSA 14	20298933	21420616	38187489	39577803	+	#FFFF00	1121683	1390314	103040	56212	103040	1224723	false
	HSA 14	21476828	24087839	39577803	42619694	+	#FFFF00	2611011	3041891	56212	71239	56212	2667223	false
	HSA 14	24159078	27018675	42622219	45710666	+	#FFFF00	2859597	3088447	71239	44079	71239	2930836	false
	HSA 14	27062754	35112351	45705141	55209328	+	#FFFF00	8049597	9504187	44079	176887018	44079	8093676	false
	HSA 15	35144936	35696068	44955976	45808774	+	#EC83BA	551132	852798	14856232	0	14856232	15407364	false
	HSA 15	35696068	35874939	45891632	46150678	+	#EC83BA	178871	259046	0	527	0	178871	false
	HSA 15	35875466	36380751	46209433	46934118	+	#EC83BA	505285	724685	527	183	527	505812	false
	HSA 15	36380934	36510902	46997888	47180292	+	#EC83BA	129968	182404	183	1661	183	130151	false
	HSA 15	36512563	36812949	45691614	46158290	+	#EC83BA	300386	466676	1661	-7497	1661	302047	false
	HSA 15	36805452	37350165	46165771	46953424	+	#EC83BA	544713	787653	-7497	52300	-7497	537216	false
	HSA 15	37402465	37708706	45703554	46158290	+	#EC83BA	306241	454736	52300	-7090	52300	358541	false
	HSA 15	37701616	38480730	46165771	47180292	+	#EC83BA	779114	1014521	-7090	1655	-7090	772024	false
	HSA 15	38482385	38558997	45691614	45793702	+	#EC83BA	76612	102088	1655	1155	1655	78267	false
	HSA 15	38560152	38701381	45862087	46077362	+	#EC83BA	141229	215275	1155	19376	1155	142384	false
	HSA 15	38720757	39296057	46165771	46935562	-	#EC83BA	575300	769791	19376	-5475	19376	594676	false
	HSA 15	39290582	39585557	45691615	46155443	-	#EC83BA	294975	463828	-5475	1778	-5475	289500	false
	HSA 15	39587335	40414635	46209433	47180292	-	#EC83BA	827300	970859	1778	31086	1778	829078	false
	HSA 15	40445721	44463307	46942900	51933059	+	#EC83BA	4017586	4990159	31086	-45006	31086	4048672	false
	HSA 15	44418301	46246095	51949331	54713540	+	#EC83BA	1827794	2764209	-45006	615	-45006	1782788	false
	HSA 15	46246710	46437393	54775085	55104102	+	#EC83BA	190683	329017	615	3824	615	191298	false
	HSA 15	46441217	47077202	55165096	56254727	+	#EC83BA	635985	1089631	3824		3824	639809	false
	HSA 5	47394896	47465207	181188122	181251982	-	#F33071	70311	63860		81259			
	HSA 5	47546466	47657439	180777946	180909452	-	#F33071	110973	131506	81259		81259	192232	false
	HSA 9	47695388	56949970	72870779	83195588	-	#819EF7	9254582	10324809	37103979	3137	37103979	46358561	false
	HSA 9	56953107	57246160	72884195	73103937	-	#819EF7	293053	219742	3137	-91524	3137	296190	false

	HSA 9	57154636	61341799	68622384	72955690	-	#819EF7	4187163	4333306	-91524	26184	-91524	4095639	false
	HSA 9	61367983	61699559	68234836	68566842	-	#819EF7	331576	332006	26184	-116876	26184	357760	false
	HSA 9	61582683	62503021	69631	1132296	+	#819EF7	920338	1062665	-116876	80212	-116876	803462	false
	HSA 9	62583233	66520713	1159197	5197933	+	#819EF7	3937480	4038736	80212	-23624	80212	4017692	false
	HSA 9	66497089	66620317	5222030	5326908	+	#819EF7	123228	104878	-23624	-116835	-23624	99604	false
	HSA 9	66503482	67461608	5308310	6286637	+	#819EF7	958126	978327	-116835	52264	-116835	841291	false
	HSA 9	67513872	67865120	6290985	6667938	+	#819EF7	351248	376953	52264	72473	52264	403512	false
	HSA 9	67937593	72693230	6739382	12052627	+	#819EF7	4755637	5313245	72473	52567	72473	4828110	false
	HSA 9	72745797	75449146	12052884	14966243	+	#819EF7	2703349	2913359	52567	11889	52567	2755916	false
	HSA 9	75461035	81408920	15078928	21102375	+	#819EF7	5947885	6023447	11889	363388	11889	5959774	false
	HSA 9	81772308	81825800	21266941	21351893	-	#819EF7	53492	84952	363388	-53492	363388	416880	false
	HSA 9	81772308	81831826	21139544	21241285	-	#819EF7	59518	101741	-53492	-59518	-53492	6026	false
	HSA 9	81772308	81832566	21175954	21229209	-	#819EF7	60258	53255	-59518	-16720	-59518	740	false
	HSA 9	81815846	90298596	21402129	30538920	+	#819EF7	8482750	9136791	-16720	49998	-16720	8466030	false
	HSA 9	90348594	91980048	30604415	32383067	+	#819EF7	1631454	1778652	49998	60270851	49998	1681452	false
	HSA 1	92030842	92201508	143799053	143909665	+	#00FF7F	170666	110612		-170663			
	HSA 1	92030845	93692526	149707679	151387494	+	#00FF7F	1661681	1679815	-170663	171195	-170663	1491018	false
	HSA 1	93863721	94781398	151390784	152247036	+	#00FF7F	917677	856252	171195	-15793	171195	1088872	false
	HSA 1	94765605	95221506	152298054	152650366	+	#00FF7F	455901	352312	-15793	-100238	-15793	440108	false
	HSA 1	95121268	95264338	152600659	152668104	-	#00FF7F	143070	67445	-100238	-143070	-100238	42832	false
	HSA 1	95121268	95372756	152624768	152679898	-	#00FF7F	251488	55130	-143070	-125254	-143070	108418	false
	HSA 1	95247502	95830301	152600659	153034950	+	#00FF7F	582799	434291	-125254	131952	-125254	457545	false
	HSA 1	95962253	96246709	153073904	153414439	+	#00FF7F	284456	340535	131952	54370	131952	416408	false
	HSA 1	96301079	96527017	88992599	89191959	+	#00FF7F	225938	199360	54370	-183727	54370	280308	false
	HSA 1	96343290	96563310	89126477	89300734	+	#00FF7F	220020	174257	-183727	-79273	-183727	36293	false
	HSA 1	96484037	96564346	89114347	89191959	+	#00FF7F	80309	77612	-79273	-49131	-79273	1036	false
	HSA 1	96515215	96631909	89331624	89385365	-	#00FF7F	116694	53741	-49131	129960	-49131	67563	false
	HSA 1	96761869	100428664	89406534	93382723	+	#00FF7F	3666795	3976189	129960	14370	129960	3796755	false
	HSA 1	100443034	109886034	93434310	103585609	+	#00FF7F	9443000	10151299	14370	-106029	14370	9457370	false
	HSA 1	109780005	109901107	103609598	103696436	+	#00FF7F	121102	86838	-106029	-121102	-106029	15073	false
	HSA 1	109780005	109901116	103616655	103710143	-	#00FF7F	121111	93488	-121102	70770	-121102	9	false
	HSA 1	109971886	113971566	103749671	108212397	+	#00FF7F	3999680	4462726	70770	915	70770	4070450	false

HSA 1	113972481	114042507	108272328	108340816	-	#00FF7F	70026	68488	915	23356	915	70941	false
HSA 1	114065863	116533460	108511309	110964572	+	#00FF7F	2467597	2453263	23356	312596	23356	2490953	false
HSA 1	116846056	118476358	111113501	112818630	+	#00FF7F	1630302	1705129	312596	-7603	312596	1942898	false
HSA 1	118468755	118534450	112867564	112929863	+	#00FF7F	65695	62299	-7603	7702	-7603	58092	false
HSA 1	118542152	118632418	112795518	112853597	+	#00FF7F	90266	58079	7702	-81616	7702	97968	false
HSA 1	118550802	125013361	112868493	119396311	+	#00FF7F	6462559	6527818	-81616	164666	-81616	6380943	false
HSA 1	125178027	125435180	119397632	119555128	+	#00FF7F	257153	157496	164666	-257153	164666	421819	false
HSA 1	125178027	125709186	119528201	119830094	+	#00FF7F	531159	301893	-257153	744	-257153	274006	false
HSA 1	125709930	126147333	119889435	120319916	+	#00FF7F	437403	430481	744	-349495	744	438147	false
HSA 1	125797838	125965663	146149170	146328881	+	#00FF7F	167825	179711	-349495	-167825	-349495	-181670	true
HSA 1	125797838	126147333	149197758	149471527	-	#00FF7F	349495	273769	-167825	-349495	-167825	181670	false
HSA 1	125797838	126242482	148600112	149098639	+	#00FF7F	444644	498527	-349495	-7276	-349495	95149	false
HSA 1	126235206	126590990	147118631	147446111	+	#00FF7F	355784	327480	-7276	88702	-7276	348508	false
HSA 1	126679692	127328295	147484001	148009863	+	#00FF7F	648603	525862	88702	-64492	88702	737305	false
HSA 1	127263803	127722493	145600531	146050941	+	#00FF7F	458690	450410	-64492	195588	-64492	394198	false
HSA 1	127918081	127988252	120965791	121098587	+	#00FF7F	70171	132796	195588	72898	195588	265759	false
HSA 1	128061150	128158909	120966158	121098587	-	#00FF7F	97759	132429	72898	99184	72898	170657	false
HSA 1	128258093	128357264	121018908	121098587	-	#00FF7F	99171	79679	99184	49817	99184	198355	false
HSA 1	128407081	128468714	120965791	121061215	+	#00FF7F	61633	95424	49817	4565	49817	111450	false
HSA 1	128473279	128551631	121018908	121098587	-	#00FF7F	78352	79679	4565	85802	4565	82917	false
HSA 1	128637433	128714512	143802256	143886008	+	#00FF7F	77079	83752	85802	46223	85802	162881	false
HSA 1	128760735	134310165	83287049	89059762	-	#00FF7F	5549430	5772713	46223	17917	46223	5595653	false
HSA 1	134328082	144991921	70819287	83210092	-	#00FF7F	10663839	12390805	17917	42557	17917	10681756	false
HSA 1	145034478	146131977	69316795	70762714	-	#00FF7F	1097499	1445919	42557	31760	42557	1140056	false
HSA 1	146163737	147069433	68013451	69264177	-	#00FF7F	905696	1250726	31760	9202	31760	937456	false
HSA 1	147078635	147175343	67672388	67884683	-	#00FF7F	96708	212295	9202	31366	9202	105910	false
HSA 1	147206709	148760299	65254038	67460916	-	#00FF7F	1553590	2206878	31366	36639	31366	1584956	false
HSA 1	148796938	152094638	59944564	65110082	-	#00FF7F	3297700	5165518	36639		36639	3334339	false
HSA 9	152250899	152660957	121016249	121447760	-	#819EF7	410058	431511	60270851	-25229	60270851	60680909	false
HSA 9	152635728	157591982	116037810	121097997	-	#819EF7	4956254	5060187	-25229	1	-25229	4931025	false
HSA 9	157591983	157646615	115976689	116037751	+	#819EF7	54632	61062	1	0	1	54633	false
HSA 9	157646615	160304160	113097314	115976644	-	#819EF7	2657545	2879330	0	24568	0	2657545	false

	HSA 9	160328728	160400329	112938835	113049970	+	#819EF7	71601	111135	24568	17217	24568	96169	false
	HSA 9	160417546	160521229	112938835	113115345	-	#819EF7	103683	176510	17217	190671	17217	120900	false
	HSA 9	160711900	160811449	112957037	113014338	+	#819EF7	99549	57301	190671	71763	190671	290220	false
	HSA 9	160883212	171186395	101362351	112936934	-	#819EF7	10303183	11574583	71763	32341	71763	10374946	false
	HSA 9	171218736	174841140	97275303	101383202	-	#819EF7	3622404	4107899	32341	0	32341	3654745	false
	HSA 9	174841140	175763553	37510708	38472134	-	#819EF7	922413	961426	0	119271	0	922413	false
	HSA 9	175882824	177142140	36169316	37503920	-	#819EF7	1259316	1334604	119271	54047	119271	1378587	false
	HSA 9	177196187	177347851	36006895	36173464	-	#819EF7	151664	166569	54047	56477	54047	205711	false
	HSA 9	177404328	178467788	34839118	36005743	-	#819EF7	1063460	1166625	56477	-130960	56477	1119937	false
	HSA 9	178336828	178478800	34720732	34811260	-	#819EF7	141972	90528	-130960	-41632	-130960	11012	false
	HSA 9	178437168	179406188	33813329	34839833	-	#819EF7	969020	1026504	-41632	1918	-41632	927388	false
	HSA 9	179408106	180593228	32341721	33474466	-	#819EF7	1185122	1132745	1918	469503	1918	1187040	false
	HSA 9	181062731	182054627	121439241	122454578	+	#819EF7	991896	1015337	469503	228705	469503	1461399	false
	HSA 9	182283332	182632311	122472925	122764184	+	#819EF7	348979	291259	228705	64921	228705	577684	false
	HSA 9	182697232	187216994	122776507	128154332	+	#819EF7	4519762	5377825	64921	2520350	64921	4584683	false
	HSA 9	189737344	192940213	128156107	133174549	+	#819EF7	3202869	5018442	2520350	165508	2520350	5723219	false
	HSA 9	193105721	194471092	133331305	135530996	-	#819EF7	1365371	2199691	165508	43476	165508	1530879	false
	HSA 9	194514568	195211388	135539549	136724262	-	#819EF7	696820	1184713	43476	6869	43476	740296	false
	HSA 9	195218257	196226901	136738777	138124581	+	#819EF7	1008644	1385804	6869		6869	1015513	false
	HSA 2	196814789	212888505	36125704	53634302	-	#036A9C	16073716	17508598	135103820	6283	135103820	151177536	false
	HSA 2	212894788	212970202	36024863	36125651	+	#036A9C	75414	100788	6283	17415	6283	81697	false
	HSA 2	212987617	215196035	33630706	36022787	-	#036A9C	2208418	2392081	17415	0	17415	2225833	false
	HSA 2	215196035	216755282	33675403	35484090	-	#036A9C	1559247	1808687	0	9399	0	1559247	false
	HSA 2	216764681	217303912	33698434	34251598	+	#036A9C	539231	553164	9399	31188	9399	548630	false
	HSA 2	217335100	218020905	34326461	35197136	+	#036A9C	685805	870675	31188	69144	31188	716993	false
	HSA 2	218090049	218499885	35246097	35723480	+	#036A9C	409836	477383	69144	92906	69144	478980	false
	HSA 2	218592791	220608334	31661914	33682916	-	#036A9C	2015543	2021002	92906	1099	92906	2108449	false
	HSA 2	220609433	233513972	17215029	31586961	-	#036A9C	12904539	14371932	1099		1099	12905638	false
**CPOR 10**	HSA 8	2106505	11065470	47257053	57010486	+	#80797D	8958965	9753433		254			
	HSA 8	11065724	11141375	57017073	57081916	-	#80797D	75651	64843	254	90158	254	75905	false
	HSA 8	11231533	12013201	57449186	58286520	+	#80797D	781668	837334	90158	159005	90158	871826	false
	HSA 8	12172206	15713048	58289625	61790204	+	#80797D	3540842	3500579	159005		159005	3699847	false

	HSA 5	16063387	16198781	79998086	80084130	-	#F33071	135394	86044	14202441	67208	14202441	14337835	false
	HSA 5	16265989	17173804	79143530	79998086	-	#F33071	907815	854556	67208	71177	67208	975023	false
	HSA 5	17244981	18011603	78354627	79132326	-	#F33071	766622	777699	71177	168818	71177	837799	false
	HSA 5	18180421	19491953	77020380	78346120	-	#F33071	1311532	1325740	168818	89660	168818	1480350	false
	HSA 5	19581613	19933831	75016530	75279458	+	#F33071	352218	262928	89660	50492	89660	441878	false
	HSA 5	19984323	21514135	75314758	76976918	+	#F33071	1529812	1662160	50492	74712	50492	1580304	false
	HSA 5	21588847	24990419	71450514	74929418	-	#F33071	3401572	3478904	74712	1	74712	3476284	false
	HSA 5	24990420	25062830	70025200	70128504	+	#F33071	72410	103304	1	-72410	1	72411	false
	HSA 5	24990420	25096741	70900618	71067130	+	#F33071	106321	166512	-72410	60138	-72410	33911	false
	HSA 5	25156879	25479170	69217296	69557336	-	#F33071	322291	340040	60138	303550	60138	382429	false
	HSA 5	25782720	29046445	65717938	69207167	-	#F33071	3263725	3489229	303550	70266	303550	3567275	false
	HSA 5	29116711	30380166	64401352	65748787	-	#F33071	1263455	1347435	70266	177936	70266	1333721	false
	HSA 5	30558102	34004172	60784368	64383466	-	#F33071	3446070	3599098	177936	121873	177936	3624006	false
	HSA 5	34126045	38904800	55723888	60964859	-	#F33071	4778755	5240971	121873	80524	121873	4900628	false
	HSA 5	38985324	39046175	70970168	71067130	+	#F33071	60851	96962	80524	110312	80524	141375	false
	HSA 5	39156487	43961323	50538233	55718204	-	#F33071	4804836	5179971	110312	34014	110312	4915148	false
	HSA 5	43995337	44255408	50275558	50485276	-	#F33071	260071	209718	34014	49894	34014	294085	false
	HSA 5	44305302	46634539	43103659	45911317	-	#F33071	2329237	2807658	49894	149498	49894	2379131	false
	HSA 5	46784037	47493889	42041360	42891536	-	#F33071	709852	850176	149498	63121	149498	859350	false
	HSA 5	47557010	52951177	36036230	42037338	-	#F33071	5394167	6001108	63121	-30524	63121	5457288	false
	HSA 5	52920653	54295973	34408241	35954803	-	#F33071	1375320	1546562	-30524	363	-30524	1344796	false
	HSA 5	54296336	58264371	29456054	34060253	-	#F33071	3968035	4604199	363	87	363	3968398	false
	HSA 5	58264458	64191970	22207223	29401685	-	#F33071	5927512	7194462	87	6681	87	5927599	false
	HSA 5	64198651	64699667	21545103	22142396	-	#F33071	501016	597293	6681	17898	6681	507697	false
	HSA 5	64717565	65076099	20923801	21439349	-	#F33071	358534	515548	17898	-324413	17898	376432	false
	HSA 6	64751686	64844631	26905056	27002576	+	#EBE47D	92945	97520		839267			
	HSA 5	64751686	64852261	69789977	69894041	-	#F33071	100575	104064	-324413	-100575	-324413	-223838	true
	HSA 5	64751686	64856365	69659162	69773097	+	#F33071	104679	113935	-100575	303092	-100575	4104	false
	HSA 5	65159457	65267777	34068734	34173691	+	#F33071	108320	104957	303092	-108320	303092	411412	false
	HSA 5	65159457	65372625	20590675	20793187	-	#F33071	213168	202512	-108320	1035	-108320	104848	false
	HSA 5	65373660	65484297	21652544	21789576	+	#F33071	110637	137032	1035	49868	1035	111672	false
	HSA 5	65534165	65624258	21545103	21652543	-	#F33071	90093	107440	49868	14927	49868	139961	false

	HSA 5	65639185	66018366	20923799	21453104	-	#F33071	379181	529305	14927	-334468	14927	394108	false
	HSA 6	65683898	65783581	26905056	27002576	+	#EBE47D	99683	97520	839267		839267	938950	false
	HSA 5	65683898	65791206	69789977	69894041	-	#F33071	107308	104064	-334468	-107308	-334468	-227160	true
	HSA 5	65683898	65795416	69659162	69773097	+	#F33071	111518	113935	-107308	293999	-107308	4210	false
	HSA 5	66089415	66189101	34068734	34173691	+	#F33071	99686	104957	293999	-99686	293999	393685	false
	HSA 5	66089415	67414133	19151074	20793187	-	#F33071	1324718	1642113	-99686	38583	-99686	1225032	false
	HSA 5	67452716	68780999	17628533	19099192	-	#F33071	1328283	1470659	38583	-5633	38583	1366866	false
	HSA 5	68775366	72819730	12813672	17529960	-	#F33071	4044364	4716288	-5633	65531	-5633	4038731	false
	HSA 5	72885261	76950057	8086848	12813648	-	#F33071	4064796	4726800	65531	-743734	65531	4130327	false
	HSA 5	76206323	76390865	7925550	8082584	+	#F33071	184542	157034	-743734	9587	-743734	-559192	true
	HSA 5	76400452	76485424	8090516	8154310	-	#F33071	84972	63794	9587	6082	9587	94559	false
	HSA 5	76491506	76608227	8177586	8291854	+	#F33071	116721	114268	6082	79274	6082	122803	false
	HSA 5	76687501	76756457	9097972	9190932	-	#F33071	68956	92960	79274	12001	79274	148230	false
	HSA 5	76768458	76913194	8495536	8642512	+	#F33071	144736	146976	12001	27928	12001	156737	false
	HSA 5	76941122	77012807	8108560	8159754	+	#F33071	71685	51194	27928	-17233	27928	99613	false
	HSA 5	76995574	77108215	8772245	8961835	+	#F33071	112641	189590	-17233	-95352	-17233	95408	false
	HSA 5	77012863	78249396	6567005	8081296	-	#F33071	1236533	1514291	-95352	665	-95352	1141181	false
	HSA 5	78250061	81612000	1650259	6509306	-	#F33071	3361939	4859047	665	598	665	3362604	false
	HSA 5	81612598	82004353	770004	1551887	-	#F33071	391755	781883	598	-65277	598	392353	false
	HSA 5	81939076	82402609	189445	769994	-	#F33071	463533	580549	-65277		-65277	398256	false
**CPOR 11**	HSA 19	406163	678514	9378027	9665130	+	#64E09A	272351	287103		-155885			
	HSA 19	522629	677616	9414919	9537503	+	#64E09A	154987	122584	-155885	-94111	-155885	-898	true
	HSA 19	583505	677740	9611412	9662287	+	#64E09A	94235	50875	-94111	1623956	-94111	124	false
	HSA 19	2301696	2399420	9445455	9585290	-	#64E09A	97724	139835	1623956	6260	1623956	1721680	false
	HSA 19	2405680	3363562	9804712	11460744	+	#64E09A	957882	1656032	6260	73436	6260	964142	false
	HSA 19	3436998	3492678	11468863	11561477	+	#64E09A	55680	92614	73436	190271	73436	129116	false
	HSA 19	3682949	3872051	6585536	6929063	-	#64E09A	189102	343527	190271	159081	190271	379373	false
	HSA 19	4031132	5424715	3956663	6566184	-	#64E09A	1393583	2609521	159081	-8308	159081	1552664	false
	HSA 19	5416407	5587404	2416235	2814772	+	#64E09A	170997	398537	-8308	8989	-8308	162689	false
	HSA 19	5596393	5657261	3271020	3454947	+	#64E09A	60868	183927	8989	0	8989	69857	false
	HSA 19	5657261	5777759	2975099	3208580	-	#64E09A	120498	233481	0	40848	0	120498	false
	HSA 19	5818607	6029486	3502539	3956686	+	#64E09A	210879	454147	40848	854	40848	251727	false

	HSA 19	6030340	7172107	474906	2436928	-	#64E09A	1141767	1962022	854	130352	854	1142621	false
	HSA 19	7302459	7397815	15614439	15721910	-	#64E09A	95356	107471	130352	-95349	130352	225708	false
	HSA 19	7302466	7408925	15844400	15927481	+	#64E09A	106459	83081	-95349	-102635	-95349	11110	false
	HSA 19	7306290	7869002	14931265	15617777	-	#64E09A	562712	686512	-102635	920571	-102635	460077	false
	HSA 11	8212936	8275246	48430046	48497818	+	#0000FF	62310	67772		83139			
	HSA 11	8358385	8423085	55710850	55800201	-	#0000FF	64700	89351	83139	189587	83139	147839	false
	HSA 11	8612672	8699459	55646394	55726873	+	#0000FF	86787	80479	189587	5189073	189587	276374	false
	HSA 19	8789573	8969774	14246583	14573526	-	#64E09A	180201	326943	920571	153	920571	1100772	false
	HSA 19	8969927	9254847	13669915	14189078	-	#64E09A	284920	519163	153	1775	153	285073	false
	HSA 19	9256622	9779914	12645065	13601276	-	#64E09A	523292	956211	1775	909886	1775	525067	false
	HSA 19	10689800	10889816	7075077	7355103	+	#64E09A	200016	280026	909886	47234	909886	1109902	false
	HSA 19	10937050	11361788	7338540	7770810	+	#64E09A	424738	432270	47234	770546	47234	471972	false
	HSA 19	12132334	12211143	8997662	9079923	-	#64E09A	78809	82261	770546	3170	770546	849355	false
	HSA 19	12214313	12334071	8807449	8941662	-	#64E09A	119758	134213	3170	255583	3170	122928	false
	HSA 19	12589654	13225261	7763099	8733250	-	#64E09A	635607	970151	255583	11862864	255583	891190	false
	HSA 11	13888532	14003336	55342545	55473678	+	#0000FF	114804	131133	5189073	67411	5189073	5303877	false
	HSA 11	14070747	14145457	48409349	48522918	-	#0000FF	74710	113569	67411	-74710	67411	142121	false
	HSA 11	14070747	14179624	49902128	50060108	+	#0000FF	108877	157980	-74710	188888	-74710	34167	false
	HSA 11	14368512	14433812	55646655	55726031	+	#0000FF	65300	79376	188888	454203	188888	254188	false
	HSA 11	14888015	14964307	54662949	54722255	+	#0000FF	76292	59306	454203	470986	454203	530495	false
	HSA 11	15435293	15503740	56024845	56123397	-	#0000FF	68447	98552	470986	251738	470986	539433	false
	HSA 11	15755478	15871056	63158412	63274439	-	#0000FF	115578	116027	251738	-115386	251738	367316	false
	HSA 11	15755670	15946557	63366299	63517749	+	#0000FF	190887	151450	-115386	11841	-115386	75501	false
	HSA 11	15958398	16081233	62852786	63025523	-	#0000FF	122835	172737	11841	73245	11841	134676	false
	HSA 11	16154478	16425791	62337476	62839931	-	#0000FF	271313	502455	73245	210528	73245	344558	false
	HSA 11	16636319	16724768	62201975	62265723	-	#0000FF	88449	63748	210528	470870	210528	298977	false
	HSA 11	17195638	17659683	61258570	62050038	+	#0000FF	464045	791468	470870	70948	470870	934915	false
	HSA 11	17730631	17919619	60763301	61251224	-	#0000FF	188988	487923	70948	21122	70948	259936	false
	HSA 11	17940741	18232934	60482692	60709601	-	#0000FF	292193	226909	21122	-49019	21122	313315	false
	HSA 11	18183915	18394792	60340085	60513477	-	#0000FF	210877	173392	-49019	-19042	-49019	161858	false
	HSA 11	18375750	18427589	60250706	60303420	+	#0000FF	51839	52714	-19042	104721	-19042	32797	false
	HSA 11	18532310	18584080	56344148	56412002	-	#0000FF	51770	67854	104721	113172	104721	156491	false

	HSA 11	18697252	18754663	56160655	56253989	-	#0000FF	57411	93334	113172	31361	113172	170583	false
	HSA 11	18786024	18840883	60279822	60345052	+	#0000FF	54859	65230	31361	155023	31361	86220	false
	HSA 11	18995906	19055184	60271868	60383230	+	#0000FF	59278	111362	155023	-58368	155023	214301	false
	HSA 11	18996816	19275348	59968157	60245111	-	#0000FF	278532	276954	-58368	130676	-58368	220164	false
	HSA 11	19406024	19471715	59820897	59875156	+	#0000FF	65691	54259	130676	1221	130676	196367	false
	HSA 11	19472936	19755572	59497260	59853080	-	#0000FF	282636	355820	1221	765	1221	283857	false
	HSA 11	19756337	19835951	58343936	58404488	+	#0000FF	79614	60552	765	46390	765	80379	false
	HSA 11	19882341	19937235	58112364	58179945	-	#0000FF	54894	67581	46390	-38719	46390	101284	false
	HSA 11	19898516	20020384	58310110	58399553	+	#0000FF	121868	89443	-38719	164456	-38719	83149	false
	HSA 11	20184840	20246227	59475376	59541044	-	#0000FF	61387	65668	164456	136565	164456	225843	false
	HSA 11	20382792	20815187	59085702	59463801	-	#0000FF	432395	378099	136565	9338	136565	568960	false
	HSA 11	20824525	21348589	58401431	58771648	-	#0000FF	524064	370217	9338	136211	9338	533402	false
	HSA 11	21484800	21862667	58109628	58404488	-	#0000FF	377867	294860	136211	90599	136211	514078	false
	HSA 11	21953266	22444349	57297501	58038221	-	#0000FF	491083	740720	90599	29058	90599	581682	false
	HSA 11	22473407	22929906	56840058	57241109	-	#0000FF	456499	401051	29058	47555	29058	485557	false
	HSA 11	22977461	23092834	56375619	56546289	+	#0000FF	115373	170670	47555	12508	47555	162928	false
	HSA 11	23105342	23370260	56596890	56893191	+	#0000FF	264918	296301	12508	18317	12508	277426	false
	HSA 11	23388577	23539760	56815194	57019034	+	#0000FF	151183	203840	18317	38324	18317	169500	false
	HSA 11	23578084	23645738	56136991	56188788	+	#0000FF	67654	51797	38324	119885	38324	105978	false
	HSA 11	23765623	23891672	56797333	56937909	-	#0000FF	126049	140576	119885	362	119885	245934	false
	HSA 11	23892034	23949902	56596890	56663006	-	#0000FF	57868	66116	362	683031	362	58230	false
	HSA 11	24632933	24684321	49980768	50057839	+	#0000FF	51388	77071	683031	3660851	683031	734419	false
	HSA 19	25088125	25373851	16183533	16893903	+	#64E09A	285726	710370	11862864	954	11862864	12148590	false
	HSA 19	25374805	25677619	17048448	17734567	+	#64E09A	302814	686119	954	465087	954	303768	false
	HSA 19	26142706	26196587	16059663	16173864	-	#64E09A	53881	114201	465087	929845	465087	518968	false
	HSA 19	27126432	28028633	17747343	19551453	-	#64E09A	902201	1804110	929845		929845	1832046	false
	HSA 11	28345172	28403993	54587618	54665230	-	#0000FF	58821	77612	3660851	-58821	3660851	3719672	false
	HSA 11	28345172	28438126	48372023	48494354	-	#0000FF	92954	122331	-58821	-89671	-58821	34133	false
	HSA 11	28348455	28454716	49915350	50055260	+	#0000FF	106261	139910	-89671	111898	-89671	16590	false
	HSA 11	28566614	41296660	33176533	48245061	-	#0000FF	12730046	15068528	111898	67387	111898	12841944	false
	HSA 11	41364047	41689197	32802442	33172332	-	#0000FF	325150	369890	67387	20776	67387	392537	false
	HSA 11	41709973	42409164	1595851	2224680	+	#0000FF	699191	628829	20776	51628	20776	719967	false

	HSA 11	42460792	43223705	2255380	3077562	+	#0000FF	762913	822182	51628	255446	51628	814541	false
	HSA 11	43479151	43633272	3067787	3239511	+	#0000FF	154121	171724	255446	72441	255446	409567	false
	HSA 11	43705713	44105169	71055662	71504575	-	#0000FF	399456	448913	72441	3585	72441	471897	false
	HSA 11	44108754	44590653	70271598	70955499	-	#0000FF	481899	683901	3585	92363	3585	485484	false
	HSA 11	44683016	46566870	67992983	70387428	-	#0000FF	1883854	2394445	92363	18644	92363	1976217	false
	HSA 11	46585514	50098413	63549724	67678162	-	#0000FF	3512899	4128438	18644		18644	3531543	false
	HSA 9	50128455	51761268	94557424	96302402	+	#819EF7	1632813	1744978		162199			
	HSA 9	51923467	52161450	96313832	96670546	-	#819EF7	237983	356714	162199	68580	162199	400182	false
	HSA 9	52230030	52292027	97032447	97087976	-	#819EF7	61997	55529	68580	65656	68580	130577	false
	HSA 9	52357683	52412030	96759773	96852215	-	#819EF7	54347	92442	65656	64316	65656	120003	false
	HSA 9	52476346	52556986	94342334	94461857	-	#819EF7	80640	119523	64316	105740	64316	144956	false
	HSA 9	52662726	52719946	60842861	60926114	-	#819EF7	57220	83253	105740	-57220	105740	162960	false
	HSA 9	52662726	52900052	41806605	42195350	-	#819EF7	237326	388745	-57220	-237326	-57220	180106	false
	HSA 9	52662726	53008079	66980839	67573761	+	#819EF7	345353	592922	-237326	-345353	-237326	108027	false
	HSA 9	52662726	53171308	38648544	39367397	-	#819EF7	508582	718853	-345353	-244134	-345353	163229	false
	HSA 9	52927174	53008079	62014950	62148001	-	#819EF7	80905	133051	-244134	268505	-244134	-163229	true
	HSA 9	53276584	53770154	92207771	92775086	+	#819EF7	493570	567315	268505	14810	268505	762075	false
	HSA 9	53784964	54062072	92944590	93321320	+	#819EF7	277108	376730	14810	64761	14810	291918	false
	HSA 9	54126833	54536288	93423222	94011011	+	#819EF7	409455	587789	64761	51263	64761	474216	false
	HSA 9	54587551	54698862	94064484	94214148	-	#819EF7	111311	149664	51263	21104	51263	162574	false
	HSA 9	54719966	54776351	94013298	94068858	+	#819EF7	56385	55560	21104	88334	21104	77489	false
	HSA 9	54864685	56370556	88161679	90185649	+	#819EF7	1505871	2023970	88334	8251	88334	1594205	false
	HSA 9	56378807	57192412	90237790	91464196	+	#819EF7	813605	1226406	8251	11140	8251	821856	false
	HSA 9	57203552	57291557	91474518	91547027	-	#819EF7	88005	72509	11140	83	11140	99145	false
	HSA 9	57291640	57845428	91554410	92122135	+	#819EF7	553788	567725	83		83	553871	false
	HSA 3	57927822	68280999	171543521	182728884	-	#02BCB7	10353177	11185363		163470			
	HSA 3	68444469	72247757	167638488	171499576	-	#02BCB7	3803288	3861088	163470		163470	3966758	false
**CPOR 12**	HSA 15	9489	2017955	66572451	69292682	-	#EC83BA	2008466	2720231		4370709			
	HSA 6	2121928	3168694	56355395	57436697	+	#EBE47D	1046766	1081302		77463			
	HSA 6	3246157	3405127	57531473	57663378	+	#EBE47D	158970	131905	77463	69628	77463	236433	false
	HSA 6	3474755	3710853	60613814	60793721	+	#EBE47D	236098	179907	69628	-116054	69628	305726	false
	HSA 6	3594799	3711108	57967053	58042502	+	#EBE47D	116309	75449	-116054	31081	-116054	255	false

	HSA 6	3742189	3816877	60808322	60861935	-	#EBE47D	74688	53613	31081	243273	31081	105769	false
	HSA 6	4060150	4180937	61134738	61267388	-	#EBE47D	120787	132650	243273	0	243273	364060	false
	HSA 6	4180937	5490317	61537971	62834249	+	#EBE47D	1309380	1296278	0	109	0	1309380	false
	HSA 6	5490426	5575887	62887610	62984060	+	#EBE47D	85461	96450	109	69937	109	85570	false
	HSA 6	5645824	6382783	63000307	63857271	+	#EBE47D	736959	856964	69937		69937	806896	false
	HSA 3	7379674	11483834	15596832	19765134	+	#02BCB7	4104160	4168302	3974547	131869	3974547	8078707	false
	HSA 3	11615703	12196429	19817223	20348862	+	#02BCB7	580726	531639	131869	9	131869	712595	false
	HSA 3	12196438	12472327	20348863	20645711	-	#02BCB7	275889	296848	9	6	9	275898	false
	HSA 3	12472333	24392446	20645711	32650039	+	#02BCB7	11920113	12004328	6	56	6	11920119	false
	HSA 3	24392502	28152993	62187084	66141471	-	#02BCB7	3760491	3954387	56	213	56	3760547	false
	HSA 3	28153206	31248371	59117889	62331123	-	#02BCB7	3095165	3213234	213	77854	213	3095378	false
	HSA 3	31326225	32038442	58205577	59064453	-	#02BCB7	712217	858876	77854	15152	77854	790071	false
	HSA 3	32053594	32250812	15402566	15611793	+	#02BCB7	197218	209227	15152	49238	15152	212370	false
	HSA 3	32300050	32460447	15200927	15357432	-	#02BCB7	160397	156505	49238	946	49238	209635	false
	HSA 3	32461393	38114432	52315137	58228598	+	#02BCB7	5653039	5913461	946		946	5653985	false
	HSA 8	38253342	48057762	62115160	72199638	+	#80797D	9804420	10084478		-155537			
	HSA 8	47902225	47977691	72199652	72286323	+	#80797D	75466	86671	-155537	59504	-155537	-80071	true
	HSA 8	48037195	56358646	72309264	81636756	+	#80797D	8321451	9327492	59504	-68001	59504	8380955	false
	HSA 8	56290645	59175095	81640463	85062424	+	#80797D	2884450	3421961	-68001	18541	-68001	2816449	false
	HSA 8	59193636	59287883	85017463	85076912	+	#80797D	94247	59449	18541	336299	18541	112788	false
	HSA 8	59624182	60091089	85086558	85577901	+	#80797D	466907	491343	336299	1698	336299	803206	false
	HSA 8	60092787	60149111	85583452	85636994	-	#80797D	56324	53542	1698	0	1698	58022	false
	HSA 8	60149111	70750575	85829883	98121265	+	#80797D	10601464	12291382	0	73603	0	10601464	false
	HSA 8	70824178	73451067	98122625	101317806	+	#80797D	2626889	3195181	73603	14341	73603	2700492	false
	HSA 8	73465408	77312531	108909606	113470570	+	#80797D	3847123	4560964	14341	8330	14341	3861464	false
	HSA 8	77320861	82565021	113526062	120051454	+	#80797D	5244160	6525392	8330	642	8330	5252490	false
	HSA 8	82565663	83211612	120122308	121014928	+	#80797D	645949	892620	642	1934	642	646591	false
	HSA 8	83213546	84031735	101318561	102440209	+	#80797D	818189	1121648	1934	2360	1934	820123	false
	HSA 8	84034095	84105131	102528245	102661410	+	#80797D	71036	133165	2360	1517	2360	73396	false
	HSA 8	84106648	87220528	102744224	107563690	+	#80797D	3113880	4819466	1517	465	1517	3115397	false
	HSA 8	87220993	88235300	107616083	108899524	+	#80797D	1014307	1283441	465		465	1014772	false
**CPOR 13**	HSA 3	8586	354324	182729166	183121751	+	#02BCB7	345738	392585		97			

HSA 3	354421	629946	197667520	198044771	-	#02BCB7	275525	377251	97	11742	97	275622	false
HSA 3	641688	804056	195699516	195923257	+	#02BCB7	162368	223741	11742	440	11742	174110	false
HSA 3	804496	2014351	196049253	197610193	+	#02BCB7	1209855	1560940	440	418	440	1210295	false
HSA 3	2014769	2780594	194541999	195600691	-	#02BCB7	765825	1058692	418	-33738	418	766243	false
HSA 3	2746856	9280934	186779178	194498238	-	#02BCB7	6534078	7719060	-33738	-49259	-33738	6500340	false
HSA 3	9231675	11263245	184496606	186778734	-	#02BCB7	2031570	2282128	-49259	52407	-49259	1982311	false
HSA 3	11315652	12479221	183121751	184468598	-	#02BCB7	1163569	1346847	52407	1984	52407	1215976	false
HSA 3	12481205	12565901	125528295	125629441	-	#02BCB7	84696	101146	1984	44262	1984	86680	false
HSA 3	12610163	13600168	46810873	48199660	-	#02BCB7	990005	1388787	44262	-29450	44262	1034267	false
HSA 3	13570718	17212674	40533501	46755913	-	#02BCB7	3641956	6222412	-29450	5208	-29450	3612506	false
HSA 3	17217882	17377281	40130901	40463187	-	#02BCB7	159399	332286	5208	2163	5208	164607	false
HSA 3	17379444	17742331	39383014	40054743	-	#02BCB7	362887	671729	2163	197735	2163	365050	false
HSA 3	17940066	17993936	65536293	65594044	-	#02BCB7	53870	57751	197735		197735	251605	false
HSA 4	18039503	20151052	159456529	162184448	-	#7FFFFF	2111549	2727919		5042			
HSA 4	20156094	20279184	161957269	162117705	-	#7FFFFF	123090	160436	5042	12479	5042	128132	false
HSA 4	20291663	22116104	159456529	161903596	-	#7FFFFF	1824441	2447067	12479	10770	12479	1836920	false
HSA 4	22126874	24027214	159502717	162117332	+	#7FFFFF	1900340	2614615	10770	4502	10770	1911110	false
HSA 4	24031716	24769795	159456528	160431100	+	#7FFFFF	738079	974572	4502	2128	4502	742581	false
HSA 4	24771923	24972627	160524530	160801468	+	#7FFFFF	200704	276938	2128	62870	2128	202832	false
HSA 4	25035497	25145609	161462263	161651326	+	#7FFFFF	110112	189063	62870	70769	62870	172982	false
HSA 4	25216378	25295776	159318860	159374774	+	#7FFFFF	79398	55914	70769	-26804	70769	150167	false
HSA 4	25268972	25383402	159313606	159374774	-	#7FFFFF	114430	61168	-26804	28310	-26804	87626	false
HSA 4	25411712	25538177	159313606	159502677	-	#7FFFFF	126465	189071	28310	29305	28310	154775	false
HSA 4	25567482	26168557	158670007	159502610	-	#7FFFFF	601075	832603	29305	54283	29305	630380	false
HSA 4	26222840	29454795	154778904	158672588	+	#7FFFFF	3231955	3893684	54283	11109	54283	3286238	false
HSA 4	29465904	29531195	154550904	154622851	+	#7FFFFF	65291	71947	11109	46	11109	76400	false
HSA 4	29531241	30307573	153717734	154550904	-	#7FFFFF	776332	833170	46	37509	46	776378	false
HSA 4	30345082	30408517	140440632	140541723	-	#7FFFFF	63435	101091	37509	5919	37509	100944	false
HSA 4	30414436	31423917	152439302	153716604	-	#7FFFFF	1009481	1277302	5919	-19589	5919	1015400	false
HSA 4	31404328	35738125	147695323	152422610	-	#7FFFFF	4333797	4727287	-19589	44368	-19589	4314208	false
HSA 4	35782493	38277727	140619607	143555023	+	#7FFFFF	2495234	2935416	44368	20891	44368	2539602	false
HSA 4	38298618	38393562	154659484	154762685	+	#7FFFFF	94944	103201	20891	21470	20891	115835	false

	HSA 4	38415032	38592056	143574850	143908830	+	#7FFFFF	177024	333980	21470	-68441	21470	198494	false
	HSA 4	38523615	38592149	143944783	144022694	+	#7FFFFF	68534	77911	-68441	-68534	-68441	93	false
	HSA 4	38523615	41634098	144054727	147695241	+	#7FFFFF	3110483	3640514	-68534	0	-68534	3041949	false
	HSA 4	41634098	59431073	119971136	140617918	-	#7FFFFF	17796975	20646782	0	-24107	0	17796975	false
	HSA 9	48952506	49057668	66275736	66390515	+	#819EF7	105162	114779		-105162			
	HSA 9	48952506	49079110	63557154	63698186	-	#819EF7	126604	141032	-105162	-126604	-105162	21442	false
	HSA 9	48952506	49131043	39879026	40100914	-	#819EF7	178537	221888	-126604	-178537	-126604	51933	false
	HSA 9	48952506	49149332	41364597	41643581	+	#819EF7	196826	278984	-178537	-60714	-178537	18289	false
	HSA 9	49088618	49149332	67727112	67831306	-	#819EF7	60714	104194	-60714		-60714	0	true
	HSA 4	59406966	59516393	119800687	119967039	-	#7FFFFF	109427	166352	-24107	88625	-24107	85320	false
	HSA 4	59605018	59898810	119471338	119800687	-	#7FFFFF	293792	329349	88625	1820	88625	382417	false
	HSA 4	59900630	60507433	118673214	119331286	-	#7FFFFF	606803	658072	1820	347	1820	608623	false
	HSA 4	60507780	62256325	116423421	118417944	-	#7FFFFF	1748545	1994523	347	4135	347	1748892	false
	HSA 4	62260460	62455478	116192442	116423421	+	#7FFFFF	195018	230979	4135	4293	4135	199153	false
	HSA 4	62459771	64844556	113445704	116192442	-	#7FFFFF	2384785	2746738	4293	212586	4293	2389078	false
	HSA 4	65057142	65924801	112223284	113405763	-	#7FFFFF	867659	1182479	212586	280	212586	1080245	false
	HSA 4	65925081	65993663	103249314	103330997	+	#7FFFFF	68582	81683	280	23072	280	68862	false
	HSA 4	66016735	66096869	103101215	103249278	+	#7FFFFF	80134	148063	23072	3261	23072	103206	false
	HSA 4	66100130	76050087	99336631	112222270	-	#7FFFFF	9949957	12885639	3261		3261	9953218	false
	HSA 11	76695984	76804629	55563118	55673257	+	#0000FF	108645	110139		133237			
	HSA 11	76937866	77082057	56666793	56824200	-	#0000FF	144191	157407	133237		133237	277428	false
**CPOR 14**	HSA 3	111898	770499	10025460	10727172	-	#02BCB7	658601	701712		-32297			
	HSA 3	738202	1029924	9649228	9999251	-	#02BCB7	291722	350023	-32297	73550	-32297	259425	false
	HSA 3	1103474	2197843	8427346	9568141	-	#02BCB7	1094369	1140795	73550	282842	73550	1167919	false
	HSA 3	2480685	2698291	38122246	38267285	-	#02BCB7	217606	145039	282842	-270	282842	500448	false
	HSA 3	2698021	3696854	38325030	39381900	+	#02BCB7	998833	1056870	-270	11557	-270	998563	false
	HSA 3	3708411	6837243	122321357	125524976	-	#02BCB7	3128832	3203619	11557	31116	11557	3140389	false
	HSA 3	6868359	6934598	122675500	122760117	+	#02BCB7	66239	84617	31116	18806	31116	97355	false
	HSA 3	6953404	7009135	95442233	95501428	+	#02BCB7	55731	59195	18806	42469	18806	74537	false
	HSA 3	7051604	7166252	98060454	98170674	-	#02BCB7	114648	110220	42469	94978	42469	157117	false
	HSA 3	7261230	8558179	121008019	122342901	-	#02BCB7	1296949	1334882	94978	13297	94978	1391927	false
	HSA 3	8571476	16564899	112829837	120957664	-	#02BCB7	7993423	8127827	13297	-88440	13297	8006720	false

	HSA 3	16476459	17126241	112323245	112841068	-	#02BCB7	649782	517823	-88440	15231	-88440	561342	false
	HSA 3	17141472	20820782	108353195	112139397	-	#02BCB7	3679310	3786202	15231	43654	15231	3694541	false
	HSA 3	20864436	24933571	104098249	108524763	-	#02BCB7	4069135	4426514	43654	136	43654	4112789	false
	HSA 3	24933707	25017214	103995048	104096678	+	#02BCB7	83507	101630	136	538	136	83643	false
	HSA 3	25017752	28459253	100482102	103984870	-	#02BCB7	3441501	3502768	538	51611	538	3442039	false
	HSA 3	28510864	28569263	100507235	100560270	-	#02BCB7	58399	53035	51611	55665	51611	110010	false
	HSA 3	28624928	30872753	98333295	100484405	-	#02BCB7	2247825	2151110	55665	182774	55665	2303490	false
	HSA 3	31055527	31166569	95405001	95526685	-	#02BCB7	111042	121684	182774	16177	182774	293816	false
	HSA 3	31182746	32304380	96665156	98039919	-	#02BCB7	1121634	1374763	16177	762	16177	1137811	false
	HSA 3	32305142	32976212	95901339	96803782	+	#02BCB7	671070	902443	762	44786	762	671832	false
	HSA 3	33020998	34192369	94032263	95352807	+	#02BCB7	1171371	1320544	44786	288	44786	1216157	false
	HSA 3	34192657	35126990	95405001	96640693	+	#02BCB7	934333	1235692	288	86080	288	934621	false
	HSA 3	35213070	36291553	94008411	95233972	+	#02BCB7	1078483	1225561	86080	66122	86080	1164563	false
	HSA 3	36357675	36679546	95435076	95901339	+	#02BCB7	321871	466263	66122	1	66122	387993	false
	HSA 3	36679547	37450763	95526206	96660756	-	#02BCB7	771216	1134550	1	36936	1	771217	false
	HSA 3	37487699	38793475	93808606	95340565	-	#02BCB7	1305776	1531959	36936	-92372	36936	1342712	false
	HSA 3	38701103	49315552	78073973	90261169	-	#02BCB7	10614449	12187196	-92372	805	-92372	10522077	false
	HSA 3	49316357	49393733	77997418	78073973	+	#02BCB7	77376	76555	805	17338	805	78181	false
	HSA 3	49411071	49552446	77845851	77969493	+	#02BCB7	141375	123642	17338	60	17338	158713	false
	HSA 3	49552506	51454185	75810436	77841562	-	#02BCB7	1901679	2031126	60		60	1901739	false
	HSA 21	51404034	51454185	9249514	9300256	+	#007FFF	50151	50742		15734			
	HSA 21	51469919	54137694	14105860	17273027	+	#007FFF	2667775	3167167	15734	22069	15734	2683509	false
	HSA 21	54159763	59499167	17324338	23665031	+	#007FFF	5339404	6340693	22069	64125	22069	5361473	false
	HSA 21	59563292	60095848	23665351	24513475	+	#007FFF	532556	848124	64125	459	64125	596681	false
	HSA 21	60096307	60212995	24582672	24730300	+	#007FFF	116688	147628	459	4635	459	117147	false
	HSA 21	60217630	60957929	24275719	25172621	+	#007FFF	740299	896902	4635	626	4635	744934	false
	HSA 21	60958555	61032968	25296358	25391714	+	#007FFF	74413	95356	626	0	626	75039	false
	HSA 21	61032968	61125538	21809268	21932233	+	#007FFF	92570	122965	0	70	0	92570	false
	HSA 21	61125608	61200176	19032793	19122970	-	#007FFF	74568	90177	70	59362	70	74638	false
	HSA 21	61259538	61386556	22405888	22608637	+	#007FFF	127018	202749	59362	48	59362	186380	false
	HSA 21	61386604	61689875	22674992	23060161	+	#007FFF	303271	385169	48	23	48	303319	false
	HSA 21	61689898	62294632	23163976	23943543	+	#007FFF	604734	779567	23	-113420	23	604757	false

	HSA 21	62181212	62287766	25117163	25228781	+	#007FFF	106554	111618	-113420	18834	-113420	-6866	true
	HSA 21	62306600	63416879	23997598	25391714	+	#007FFF	1110279	1394116	18834	-112558	18834	1129113	false
	HSA 21	63304321	63412620	23958270	24092804	+	#007FFF	108299	134534	-112558	30510	-112558	-4259	true
	HSA 21	63443130	65150654	44499204	46666244	-	#007FFF	1707524	2167040	30510	0	30510	1738034	false
	HSA 21	65150654	65616524	43715410	44469116	+	#007FFF	465870	753706	0	16041	0	465870	false
	HSA 21	65632565	66595228	39836973	41283948	+	#007FFF	962663	1446975	16041	2209	16041	978704	false
	HSA 21	66597437	66653658	41291409	41409178	-	#007FFF	56221	117769	2209	128513	2209	58430	false
	HSA 21	66782171	67434492	41719444	42789353	+	#007FFF	652321	1069909	128513	4179	128513	780834	false
	HSA 21	67438671	67612967	42848016	43131622	+	#007FFF	174296	283606	4179	36814	4179	178475	false
	HSA 21	67649781	67716172	43272556	43376608	+	#007FFF	66391	104052	36814	1960	36814	103205	false
	HSA 21	67718132	67811698	5966517	6076907	-	#007FFF	93566	110390	1960	-93566	1960	95526	false
	HSA 21	67718132	67902555	43461357	43694566	+	#007FFF	184423	233209	-93566	26399	-93566	90857	false
	HSA 21	67928954	70902264	36112765	39805344	-	#007FFF	2973310	3692579	26399	126898	26399	2999709	false
	HSA 21	71029162	73461731	33473440	36074079	-	#007FFF	2432569	2600639	126898		126898	2559467	false
**CPOR 15**	HSA 6	400660	688827	99708003	99864893	-	#EBE47D	288167	156890		64695			
	HSA 6	753522	5563053	95074884	99685500	-	#EBE47D	4809531	4610616	64695	48	64695	4874226	false
	HSA 6	5563101	10474513	89881650	95020635	-	#EBE47D	4911412	5138985	48	74987	48	4911460	false
	HSA 6	10549500	13594252	86728083	89821309	-	#EBE47D	3044752	3093226	74987	3796	74987	3119739	false
	HSA 6	13598048	13696764	86364351	86445708	+	#EBE47D	98716	81357	3796	3952	3796	102512	false
	HSA 6	13700716	13840906	86525525	86728083	+	#EBE47D	140190	202558	3952	14092	3952	144142	false
	HSA 6	13854998	14047837	86117378	86364133	-	#EBE47D	192839	246755	14092	117432	14092	206931	false
	HSA 6	14165269	14287678	85886997	85968412	-	#EBE47D	122409	81415	117432	15	117432	239841	false
	HSA 6	14287693	15444943	84641557	85757319	-	#EBE47D	1157250	1115762	15	33730	15	1157265	false
	HSA 6	15478673	19469629	80101386	84591076	-	#EBE47D	3990956	4489690	33730	49503	33730	4024686	false
	HSA 6	19519132	19686506	49596597	49790895	+	#EBE47D	167374	194298	49503	11388	49503	216877	false
	HSA 6	19697894	19762174	49725385	49790895	+	#EBE47D	64280	65510	11388	31239	11388	75668	false
	HSA 6	19793413	22698720	49709048	52603202	+	#EBE47D	2905307	2894154	31239	107473	31239	2936546	false
	HSA 6	22806193	22920876	52603202	52688278	+	#EBE47D	114683	85076	107473	42661	107473	222156	false
	HSA 6	22963537	23074773	52796857	52932556	+	#EBE47D	111236	135699	42661	89989	42661	153897	false
	HSA 6	23164762	23253463	52816042	52928678	+	#EBE47D	88701	112636	89989	-88701	89989	178690	false
	HSA 6	23164762	23254633	52882852	52933893	+	#EBE47D	89871	51041	-88701	-78667	-88701	1170	false
	HSA 6	23175966	23271552	52812258	52932556	-	#EBE47D	95586	120298	-78667	30343	-78667	16919	false

	HSA 6	23301895	23352751	52837771	52910338	+	#EBE47D	50856	72567	30343	28653	30343	81199	false
	HSA 6	23381404	24295738	159682124	160524411	+	#EBE47D	914334	842287	28653	-43897	28653	942987	false
	HSA 6	24251841	24320126	160699909	160758468	-	#EBE47D	68285	58559	-43897	-24365	-43897	24388	false
	HSA 6	24295761	29976963	160914525	167247669	+	#EBE47D	5681202	6333144	-24365	-114862	-24365	5656837	false
	HSA 6	29862101	29980523	167250971	167393888	-	#EBE47D	118422	142917	-114862	4749	-114862	3560	false
	HSA 6	29985272	30134180	167398858	167588915	+	#EBE47D	148908	190057	4749	248	4749	153657	false
	HSA 6	30134428	30478803	167642037	168060722	+	#EBE47D	344375	418685	248	1581	248	344623	false
	HSA 6	30480384	33442320	146587847	149922269	+	#EBE47D	2961936	3334422	1581	-44404	1581	2963517	false
	HSA 6	33397916	33502677	150015714	150096305	+	#EBE47D	104761	80591	-44404	-90107	-44404	60357	false
	HSA 6	33412570	33910418	149999880	150398427	+	#EBE47D	497848	398547	-90107	91581	-90107	407741	false
	HSA 6	34001999	37454539	133551016	137648909	-	#EBE47D	3452540	4097893	91581	35795	91581	3544121	false
	HSA 6	37490334	45784425	150405175	159656885	-	#EBE47D	8294091	9251710	35795	82831	35795	8329886	false
	HSA 6	45867256	46112325	127269648	127549096	+	#EBE47D	245069	279448	82831	-46840	82831	327900	false
	HSA 6	46065485	49983071	127549873	131915202	+	#EBE47D	3917586	4365329	-46840	61031	-46840	3870746	false
	HSA 6	50044102	50653979	131915224	132562858	+	#EBE47D	609877	647634	61031	324425	61031	670908	false
	HSA 6	50978404	51107320	127269648	127442040	-	#EBE47D	128916	172392	324425	232456	324425	453341	false
	HSA 6	51339776	52305740	132555405	133550122	+	#EBE47D	965964	994717	232456	3920	232456	1198420	false
	HSA 6	52309660	60319301	137660081	146859923	-	#EBE47D	8009641	9199842	3920	75179	3920	8013561	false
	HSA 6	60394480	60470173	123990726	124069152	+	#EBE47D	75693	78426	75179		75179	150872	false
**CPOR 16**	HSA 4	63	819585	1654869	2841422	+	#7FFFFF	819522	1186553		272860			
	HSA 4	1092445	1708551	2841739	3863252	+	#7FFFFF	616106	1021513	272860	549	272860	888966	false
	HSA 4	1709100	1898853	8590125	8913117	-	#7FFFFF	189753	322992	549	360	549	190302	false
	HSA 4	1899213	4487190	4197110	8478611	-	#7FFFFF	2587977	4281501	360	2186	360	2588337	false
	HSA 4	4489376	4721253	9763159	10130232	+	#7FFFFF	231877	367073	2186	569530	2186	234063	false
	HSA 4	5290783	10019244	10399232	15665498	+	#7FFFFF	4728461	5266266	569530	132801	569530	5297991	false
	HSA 4	10152045	10270577	15680330	15851096	+	#7FFFFF	118532	170766	132801	93	132801	251333	false
	HSA 4	10270670	14836922	15712485	20745871	+	#7FFFFF	4566252	5033386	93	1000	93	4566345	false
	HSA 4	14837922	15633177	498744	1659398	-	#7FFFFF	795255	1160654	1000		1000	796255	false
	HSA 2	15879417	15978543	95641982	95743177	-	#036A9C	99126	101195	5761927	-99126	5761927	5861053	false
	HSA 2	15879417	16382052	112906040	113384792	-	#036A9C	502635	478752	-99126	-23620	-99126	403509	false
	HSA 2	16358432	16482824	112906040	113033149	+	#036A9C	124392	127109	-23620	3297	-23620	100772	false
	HSA 2	16486121	16566913	112783396	112916797	+	#036A9C	80792	133401	3297	-678	3297	84089	false

	HSA 2	16566235	16929604	112439173	112907165	-	#036A9C	363369	467992	-678	5466	-678	362691	false
	HSA 2	16935070	17177090	112062584	112345869	-	#036A9C	242020	283285	5466	99261	5466	247486	false
	HSA 2	17276351	17757032	111457135	112037683	-	#036A9C	480681	580548	99261	-69157	99261	579942	false
	HSA 2	17687875	17757032	87431447	87493090	+	#036A9C	69157	61643	-69157	19239	-69157	0	true
	HSA 2	17776271	17907497	87551649	87695749	+	#036A9C	131226	144100	19239	-131226	19239	150465	false
	HSA 2	17776271	18431281	110636062	111399171	-	#036A9C	655010	763109	-131226	49296	-131226	523784	false
	HSA 2	18480577	18580690	110064741	110235529	-	#036A9C	100113	170788	49296	-18716	49296	149409	false
	HSA 2	18561974	18864991	94979408	95414295	+	#036A9C	303017	434887	-18716	1226	-18716	284301	false
	HSA 2	18866217	19139468	96024396	96375948	+	#036A9C	273251	351552	1226	1378	1226	274477	false
	HSA 2	19140846	19500011	96473238	96979218	+	#036A9C	359165	505980	1378	10462	1378	360543	false
	HSA 2	19510473	20673278	97643499	99154642	+	#036A9C	1162805	1511143	10462	62228	10462	1173267	false
	HSA 2	20735506	22281566	99154681	101195745	+	#036A9C	1546060	2041064	62228	110581	62228	1608288	false
	HSA 2	22392147	23074575	101250572	102281034	+	#036A9C	682428	1030462	110581	2420	110581	793009	false
	HSA 2	23076995	23231909	102222992	102473346	-	#036A9C	154914	250354	2420	53078	2420	157334	false
	HSA 2	23284987	25991341	102222992	105964771	+	#036A9C	2706354	3741779	53078	3672	53078	2759432	false
	HSA 2	25995013	26079607	106015073	106203482	+	#036A9C	84594	188409	3672	40396	3672	88266	false
	HSA 2	26120003	26861944	106531163	107825036	+	#036A9C	741941	1293873	40396	904	40396	782337	false
	HSA 2	26862848	27043198	107924082	108181013	+	#036A9C	180350	256931	904	13524	904	181254	false
	HSA 2	27056722	27258851	108254021	108321975	+	#036A9C	202129	67954	13524	-5312	13524	215653	false
	HSA 2	27253539	28091975	108383386	109725990	+	#036A9C	838436	1342604	-5312	11002	-5312	833124	false
	HSA 2	28102977	28267513	90010600	90097308	+	#036A9C	164536	86708	11002	-86529	11002	175538	false
	HSA 2	28180984	28267381	88950762	89043462	-	#036A9C	86397	92700	-86529	-86397	-86529	-132	true
	HSA 2	28180984	28267513	88968302	89049569	-	#036A9C	86529	81267	-86397	81393	-86397	132	false
	HSA 2	28348906	28404699	88958553	89011637	-	#036A9C	55793	53084	81393	178788	81393	137186	false
	HSA 2	28583487	28641231	88950730	89011637	-	#036A9C	57744	60907	178788	137897	178788	236532	false
	HSA 2	28779128	28836639	90023826	90097308	+	#036A9C	57511	73482	137897	-26971	137897	195408	false
	HSA 2	28809668	28866723	88958553	89011637	-	#036A9C	57055	53084	-26971	88760	-26971	30084	false
	HSA 2	28955483	29014833	88947053	89009858	-	#036A9C	59350	62805	88760	-59350	88760	148110	false
	HSA 2	28955483	29017814	89011620	89117218	-	#036A9C	62331	105598	-59350	-62331	-59350	2981	false
	HSA 2	28955483	29017818	88968772	89045866	-	#036A9C	62335	77094	-62331	-62335	-62331	4	false
	HSA 2	28955483	29017959	90011331	90097308	+	#036A9C	62476	85977	-62335	67731	-62335	141	false
	HSA 2	29085690	29165192	88945761	89011637	-	#036A9C	79502	65876	67731	-79490	67731	147233	false

	HSA 2	29085702	29169717	88953570	89043462	-	#036A9C	84015	89892	-79490	-84015	-79490	4525	false
	HSA 2	29085702	29218931	88958553	89049569	-	#036A9C	133229	91016	-84015	-76543	-84015	49214	false
	HSA 2	29142388	29218931	89936775	90026381	+	#036A9C	76543	89606	-76543	54349	-76543	0	true
	HSA 2	29273280	29487091	90019986	90072464	+	#036A9C	213811	52478	54349	-213811	54349	268160	false
	HSA 2	29273280	29713909	88978276	89161951	-	#036A9C	440629	183675	-213811	-437422	-213811	226818	false
	HSA 2	29276487	29717569	90047485	90179948	+	#036A9C	441082	132463	-437422	-288702	-437422	3660	false
	HSA 2	29428867	29582190	90100861	90160706	+	#036A9C	153323	59845	-288702	-153323	-288702	-135379	true
	HSA 2	29428867	29582191	90154697	90210903	+	#036A9C	153324	56206	-153323	-153324	-153323	1	false
	HSA 2	29428867	29620430	88992092	89045866	-	#036A9C	191563	53774	-153324	-191563	-153324	38239	false
	HSA 2	29428867	29643338	88958553	89040100	-	#036A9C	214471	81547	-191563	-214454	-191563	22908	false
	HSA 2	29428884	29627344	88995382	89045859	-	#036A9C	198460	50477	-214454	-134147	-214454	-15994	true
	HSA 2	29493197	29667403	90004613	90079857	+	#036A9C	174206	75244	-134147	-170971	-134147	40059	false
	HSA 2	29496432	29620430	89952657	90011525	+	#036A9C	123998	58868	-170971	-118652	-170971	-46973	true
	HSA 2	29501778	29667403	90099915	90157567	+	#036A9C	165625	57652	-118652	-165625	-118652	46973	false
	HSA 2	29501778	29713909	90153750	90235559	+	#036A9C	212131	81809	-165625	-110246	-165625	46506	false
	HSA 2	29603663	29717779	89847437	89950580	+	#036A9C	114116	103143	-110246	-42	-110246	3870	false
	HSA 2	29717737	29829457	89274046	89327042	-	#036A9C	111720	52996	-42	-46051	-42	111678	false
	HSA 2	29783406	30053105	89077433	89157006	-	#036A9C	269699	79573	-46051	-269326	-46051	223648	false
	HSA 2	29783779	30164099	90025731	90102128	+	#036A9C	380320	76397	-269326	-297529	-269326	110994	false
	HSA 2	29866570	30108496	88897277	89011637	-	#036A9C	241926	114360	-297529	-241914	-297529	-55603	true
	HSA 2	29866582	30064923	88971558	89049569	-	#036A9C	198341	78011	-241914	-187604	-241914	-43573	true
	HSA 2	29877319	30064729	90010476	90074342	+	#036A9C	187410	63866	-187604	-186949	-187604	-194	true
	HSA 2	29877780	30136505	90099739	90154901	+	#036A9C	258725	55162	-186949	-258423	-186949	71776	false
	HSA 2	29878082	30164095	90159757	90211916	+	#036A9C	286013	52159	-258423	-286013	-258423	27590	false
	HSA 2	29878082	30164099	90082712	90191135	+	#036A9C	286017	108423	-286013	-278154	-286013	4	false
	HSA 2	29885945	30164099	89988597	90084676	+	#036A9C	278154	96079	-278154	-222332	-278154	0	true
	HSA 2	29941767	30032654	90100416	90151508	+	#036A9C	90887	51092	-222332	-90887	-222332	-131445	true
	HSA 2	29941767	30136466	90160013	90210816	+	#036A9C	194699	50803	-90887	-133981	-90887	103812	false
	HSA 2	30002485	30064927	89847571	89910100	+	#036A9C	62442	62529	-133981	-61709	-133981	-71539	true
	HSA 2	30003218	30164099	89955547	90011664	+	#036A9C	160881	56117	-61709	-76165	-61709	99172	false
	HSA 2	30087934	30140620	90010639	90102244	-	#036A9C	52686	91605	-76165	-50836	-76165	-23479	true
	HSA 2	30089784	30141179	89048009	89103783	+	#036A9C	51395	55774	-50836	47174	-50836	559	false

	HSA 2	30188353	30328555	89912457	90011565	+	#036A9C	140202	99108	47174	-140202	47174	187376	false
	HSA 2	30188353	30332978	89170388	89301247	-	#036A9C	144625	130859	-140202	-80874	-140202	4423	false
	HSA 2	30252104	30332966	90152894	90210855	+	#036A9C	80862	57961	-80874	-80783	-80874	-12	true
	HSA 2	30252183	30332974	89099489	89171404	-	#036A9C	80791	71915	-80783	-73844	-80783	8	false
	HSA 2	30259130	30332978	90115093	90191035	+	#036A9C	73848	75942	-73844	-72172	-73844	4	false
	HSA 2	30260806	30332975	90113091	90210903	+	#036A9C	72169	97812	-72172	12433	-72172	-3	true
	HSA 2	30345408	30521582	89100446	89197070	-	#036A9C	176174	96624	12433	-172795	12433	188607	false
	HSA 2	30348787	30508530	97331227	97386240	+	#036A9C	159743	55013	-172795	-124853	-172795	-13052	true
	HSA 2	30383677	30521582	88947888	89042234	-	#036A9C	137905	94346	-124853	-133278	-124853	13052	false
	HSA 2	30388304	30581393	90026933	90121845	+	#036A9C	193089	94912	-133278	-170693	-133278	59811	false
	HSA 2	30410700	30581393	89959151	90052640	+	#036A9C	170693	93489	-170693	-147838	-170693	0	true
	HSA 2	30433555	30555297	88966451	89027518	+	#036A9C	121742	61067	-147838	-121742	-147838	-26096	true
	HSA 2	30433555	30555302	89046179	89142931	+	#036A9C	121747	96752	-121742	580990	-121742	5	false
	HSA 2	31136292	31187689	88947192	89011623	+	#036A9C	51397	64431	580990	976534	580990	632387	false
	HSA 2	32164223	32647107	88097893	88755713	-	#036A9C	482884	657820	976534	88795	976534	1459418	false
	HSA 2	32735902	32823129	87997696	88127486	-	#036A9C	87227	129790	88795	-1358	88795	176022	false
	HSA 2	32821771	39319608	79102644	86861180	-	#036A9C	6497837	7758536	-1358	-18553	-1358	6496479	false
	HSA 2	39301055	44132278	73700155	79088459	-	#036A9C	4831223	5388304	-18553	45350	-18553	4812670	false
	HSA 2	44177628	44268060	73670641	73747048	-	#036A9C	90432	76407	45350	-86043	45350	135782	false
	HSA 2	44182017	46410062	71178575	73644030	-	#036A9C	2228045	2465455	-86043	-6142	-86043	2142002	false
	HSA 2	46403920	54598733	62099555	71091031	-	#036A9C	8194813	8991476	-6142	80833	-6142	8188671	false
	HSA 2	54679566	59081738	57135308	61923744	-	#036A9C	4402172	4788436	80833	880	80833	4483005	false
	HSA 2	59082618	59143953	57042652	57125923	+	#036A9C	61335	83271	880	6720	880	62215	false
	HSA 2	59150673	59575081	56575300	57035651	-	#036A9C	424408	460351	6720	116	6720	431128	false
	HSA 2	59575197	59720748	56417216	56573542	+	#036A9C	145551	156326	116	1397	116	145667	false
	HSA 2	59722145	62564633	53634559	56413562	-	#036A9C	2842488	2779003	1397		1397	2843885	false
**CPOR 17**	HSA 6	797	160525	66930244	67198768	+	#EBE47D	159728	268524		4431			
	HSA 6	164956	3620936	63872374	67843072	+	#EBE47D	3455980	3970698	4431	37071	4431	3460411	false
	HSA 6	3658007	8490047	67930656	73231973	+	#EBE47D	4832040	5301317	37071	25442	37071	4869111	false
	HSA 6	8515489	8653259	75146681	75293147	+	#EBE47D	137770	146466	25442	1006	25442	163212	false
	HSA 6	8654265	9930649	78833631	80148526	-	#EBE47D	1276384	1314895	1006	7002	1006	1277390	false
	HSA 6	9937651	14958805	73392037	78728131	-	#EBE47D	5021154	5336094	7002		7002	5028156	false

HSA 16	15146175	16156088	30348340	31531343	-	#0A803B	1009913	1183003		6082			
HSA 16	16162170	16649238	29643419	30205408	+	#0A803B	487068	561989	6082	-43776	6082	493150	false
HSA 16	16605462	16723988	28477173	28614895	-	#0A803B	118526	137722	-43776	56022	-43776	74750	false
HSA 16	16780010	16931599	28819510	29032722	+	#0A803B	151589	213212	56022	73547	56022	207611	false
HSA 16	17005146	20115042	25087329	28323949	-	#0A803B	3109896	3236620	73547	-58250	73547	3183443	false
HSA 16	20056792	22482386	22561812	25060377	-	#0A803B	2425594	2498565	-58250	-45761	-58250	2367344	false
HSA 16	22436625	22647951	21558877	21796206	-	#0A803B	211326	237329	-45761	41	-45761	165565	false
HSA 16	22647992	23097802	21937162	22419424	-	#0A803B	449810	482262	41	79528	41	449851	false
HSA 16	23177330	23732823	20709425	21340339	-	#0A803B	555493	630914	79528	82357	79528	635021	false
HSA 16	23815180	23938040	14954804	15100600	-	#0A803B	122860	145796	82357	29	82357	205217	false
HSA 16	23938069	24637042	15395211	16308689	+	#0A803B	698973	913478	29	-61688	29	699002	false
HSA 16	24575354	24639675	18484014	18558886	-	#0A803B	64321	74872	-61688	7747	-61688	2633	false
HSA 16	24647422	24736356	15234128	15345692	-	#0A803B	88934	111564	7747	-82479	7747	96681	false
HSA 16	24653877	24811967	16416549	16570806	+	#0A803B	158090	154257	-82479	57954	-82479	75611	false
HSA 16	24869921	26110821	16763298	18078901	-	#0A803B	1240900	1315603	57954	199246	57954	1298854	false
HSA 16	26310067	26377198	18782975	18865664	+	#0A803B	67131	82689	199246	149220	199246	266377	false
HSA 16	26526418	26862965	18982364	19381928	-	#0A803B	336547	399564	149220	16359	149220	485767	false
HSA 16	26879324	27830675	19385119	20445089	+	#0A803B	951351	1059970	16359	137671	16359	967710	false
HSA 16	27968346	28050532	20616258	20705082	-	#0A803B	82186	88824	137671	9144	137671	219857	false
HSA 16	28059676	28278233	14411883	14733677	-	#0A803B	218557	321794	9144	61774	9144	227701	false
HSA 16	28340007	31896658	10385614	14362060	-	#0A803B	3556651	3976446	61774	153776	61774	3618425	false
HSA 16	32050434	38568089	2895922	10277096	-	#0A803B	6517655	7381174	153776	23872	153776	6671431	false
HSA 11	36437925	36514257	3525406	3592334	+	#0000FF	76332	66928		21147			
HSA 12	36437925	36514257	8334179	8407894	+	#8D3B0C	76332	73715		21147			
HSA 3	36437925	36514257	75516752	75591357	+	#02BCB7	76332	74605		21147			
HSA 4	36437925	36514257	4052841	4118987	+	#7FFFFF	76332	66146		21147			
HSA 7	36437925	36514257	97879403	97939692	+	#7F00FF	76332	60289		21599			
HSA 8	36437925	36514257	7164570	7239333	+	#80797D	76332	74763		21147			
HSA 11	36535404	36597606	71700981	71804772	+	#0000FF	62202	103791	21147	-62202	21147	83349	false
HSA 12	36535404	36598244	8226566	8329904	-	#8D3B0C	62840	103338	21147	16776136	21147	83987	false
HSA 4	36535404	36598244	9062723	9171443	+	#7FFFFF	62840	108720	21147	-62840	21147	83987	false
HSA 8	36535404	36598244	12419822	12538488	-	#80797D	62840	118666	21147	-62840	21147	83987	false

HSA 3	36535404	36600430	130091238	130189100	-	#02BCB7	65026	97862	21147	-65026	21147	86173	false
HSA 4	36535404	36600430	9594834	9710831	+	#7FFFFF	65026	115997	-62840	-65026	-62840	2186	false
HSA 8	36535404	36600430	8127194	8241422	+	#80797D	65026	114228	-62840		-62840	2186	false
HSA 3	36535404	36600486	75414969	75512459	-	#02BCB7	65082	97490	-65026	-65082	-65026	56	false
HSA 4	36535404	36600549	3935273	4049608	-	#7FFFFF	65145	114335	-65026		-65026	119	false
HSA 11	36535404	36600602	3401523	3521111	-	#0000FF	65198	119588	-62202		-62202	2996	false
HSA 3	36535404	36600602	125826951	125934781	+	#02BCB7	65198	107830	-65082		-65082	116	false
HSA 7	36535856	36600602	6923581	6999914	-	#7F00FF	64746	76333	21599		21599	86345	false
HSA 16	38591961	38784598	2734074	2871351	+	#0A803B	192637	137277	23872	119055	23872	216509	false
HSA 16	38903653	41444288	28451	2543394	-	#0A803B	2540635	2514943	119055		119055	2659690	false
HSA 22	41559149	41933505	21544531	21985513	-	#F400E1	374356	440982		191407			
HSA 22	42124912	43223087	19021794	20323822	-	#F400E1	1098175	1302028	191407	3011	191407	1289582	false
HSA 22	43226098	43458494	20392941	20626226	+	#F400E1	232396	233285	3011	3174	3011	235407	false
HSA 22	43461668	43846927	20700317	21081007	-	#F400E1	385259	380690	3174	9059	3174	388433	false
HSA 22	43855986	44091701	25454840	25730990	-	#F400E1	235715	276150	9059	-69387	9059	244774	false
HSA 22	44022314	44423786	24735286	25276737	-	#F400E1	401472	541451	-69387	59724	-69387	332085	false
HSA 22	44483510	47808192	25733255	29398529	+	#F400E1	3324682	3665274	59724	-38148	59724	3384406	false
HSA 22	47770044	50255051	29416456	32009282	+	#F400E1	2485007	2592826	-38148	62291	-38148	2446859	false
HSA 22	50317342	50387512	32034111	32111152	+	#F400E1	70170	77041	62291	975966	62291	132461	false
HSA 22	51363478	51450568	22475586	22610793	+	#F400E1	87090	135207	975966	52163	975966	1063056	false
HSA 22	51502731	51749006	22768885	22882111	+	#F400E1	246275	113226	52163	-24246	52163	298438	false
HSA 22	51724760	51933182	22767159	22882111	+	#F400E1	208422	114952	-24246	-94964	-24246	184176	false
HSA 22	51838218	52022249	22768885	22880990	+	#F400E1	184031	112105	-94964	-54947	-94964	89067	false
HSA 22	51967302	52035932	22601502	22702155	+	#F400E1	68630	100653	-54947	-13640	-54947	13683	false
HSA 22	52022292	52080720	22712684	22772025	+	#F400E1	58428	59341	-13640	-3558	-13640	44788	false
HSA 22	52077162	52562317	22877531	23347362	+	#F400E1	485155	469831	-3558	-33242	-3558	481597	false
HSA 22	52529075	52929708	24266471	24636386	+	#F400E1	400633	369915	-33242	-7956	-33242	367391	false
HSA 22	52921752	53327959	23737441	24250892	-	#F400E1	406207	513451	-7956	2026876	-7956	398251	false
HSA 12	53374380	54004629	109195494	110053321	-	#8D3B0C	630249	857827	16776136	15031	16776136	17406385	false
HSA 12	54019660	55190923	107913428	109270715	+	#8D3B0C	1171263	1357287	15031	218314	15031	1186294	false
HSA 22	55354835	55440855	24715762	24791836	+	#F400E1	86020	76074	2026876		2026876	2112896	false
HSA 12	55409237	61052760	114165994	121065576	-	#8D3B0C	5643523	6899582	218314	723580	218314	5861837	false

	HSA 12	61776340	63154977	112113330	114103569	-	#8D3B0C	1378637	1990239	723580	60831	723580	2102217	false
	HSA 12	63215808	64689882	110059691	112113298	-	#8D3B0C	1474074	2053607	60831	-25152	60831	1534905	false
	HSA 12	64664730	66701801	121104364	124605403	+	#8D3B0C	2037071	3501039	-25152	40243	-25152	2011919	false
	HSA 12	66742044	67248731	124758167	125684078	+	#8D3B0C	506687	925911	40243	49991	40243	546930	false
	HSA 12	67298722	67708308	127346260	128024927	-	#8D3B0C	409586	678667	49991	182	49991	459577	false
	HSA 12	67708490	67797988	126348628	126422386	+	#8D3B0C	89498	73758	182	-89498	182	89680	false
	HSA 12	67708490	67889883	126997015	127290068	-	#8D3B0C	181393	293053	-89498	2302	-89498	91895	false
	HSA 12	67892185	67956043	126882325	126978165	+	#8D3B0C	63858	95840	2302	1024	2302	66160	false
	HSA 12	67957067	68028917	126756738	126880569	-	#8D3B0C	71850	123831	1024	427	1024	72874	false
	HSA 12	68029344	68215354	127522447	127771832	+	#8D3B0C	186010	249385	427	2486	427	186437	false
	HSA 12	68217840	68360395	126224401	126431062	-	#8D3B0C	142555	206661	2486	58685	2486	145041	false
	HSA 12	68419080	68615469	125661013	126083725	-	#8D3B0C	196389	422712	58685	103011	58685	255074	false
	HSA 12	68718480	69028446	127346260	127925602	-	#8D3B0C	309966	579342	103011	182	103011	412977	false
	HSA 12	69028628	69115672	126348628	126422386	+	#8D3B0C	87044	73758	182	-87044	182	87226	false
	HSA 12	69028628	69204327	126997015	127290068	-	#8D3B0C	175699	293053	-87044	2302	-87044	88655	false
	HSA 12	69206629	69266545	126882325	126978165	+	#8D3B0C	59916	95840	2302	1309	2302	62218	false
	HSA 12	69267854	69487313	126424952	126880569	-	#8D3B0C	219459	455617	1309	3189	1309	220768	false
	HSA 12	69490502	69555000	126460031	126565080	-	#8D3B0C	64498	105049	3189	20575	3189	67687	false
	HSA 12	69575575	69990596	125693248	126395917	-	#8D3B0C	415021	702669	20575	5	20575	435596	false
	HSA 12	69990601	70153598	125734482	126093931	+	#8D3B0C	162997	359449	5	1158	5	163002	false
	HSA 12	70154756	70588416	126176601	126880569	+	#8D3B0C	433660	703968	1158	1056	1158	434818	false
	HSA 12	70589472	70655432	126883138	126978165	-	#8D3B0C	65960	95027	1056	4893	1056	67016	false
	HSA 12	70660325	70832031	127002770	127290068	+	#8D3B0C	171706	287298	4893	182	4893	176599	false
	HSA 12	70832213	70884440	127346260	127442795	+	#8D3B0C	52227	96535	182	416	182	52409	false
	HSA 12	70884856	70993930	127813079	128024927	+	#8D3B0C	109074	211848	416	16059	416	109490	false
	HSA 12	71009989	71302234	128093689	128746456	+	#8D3B0C	292245	652767	16059	12741	16059	308304	false
	HSA 12	71314975	71788287	128798391	129764639	+	#8D3B0C	473312	966248	12741	391	12741	486053	false
	HSA 12	71788678	71857674	129841237	129994389	+	#8D3B0C	68996	153152	391	17	391	69387	false
	HSA 12	71857691	72111691	130045076	130672866	+	#8D3B0C	254000	627790	17	7535	17	254017	false
	HSA 12	72119226	72270156	130790640	131139486	+	#8D3B0C	150930	348846	7535	59099	7535	158465	false
	HSA 12	72329255	72424912	131390616	131636297	+	#8D3B0C	95657	245681	59099	411	59099	154756	false
	HSA 12	72425323	73322090	131708851	133204347	+	#8D3B0C	896767	1495496	411		411	897178	false

**CPOR 18**	HSA 2	259315	415633	201621849	201783809	-	#036A9C	156318	161960		3959			
	HSA 2	419592	5722217	194168712	201481682	-	#036A9C	5302625	7312970	3959	148	3959	5306584	false
	HSA 2	5722365	12515209	185861171	194116180	-	#036A9C	6792844	8255009	148	-19102	148	6792992	false
	HSA 2	12496107	14084055	183784212	185835812	-	#036A9C	1587948	2051600	-19102	79	-19102	1568846	false
	HSA 2	14084134	14290440	183546997	183771413	+	#036A9C	206306	224416	79	3487	79	206385	false
	HSA 2	14293927	19055075	178108481	183544663	-	#036A9C	4761148	5436182	3487	37390	3487	4764635	false
	HSA 2	19092465	61176548	132366508	178164184	-	#036A9C	42084083	45797676	37390	30343	37390	42121473	false
	HSA 2	61206891	66858353	113672181	119406730	+	#036A9C	5651462	5734549	30343	280461	30343	5681805	false
	HSA 2	67138814	74553928	119407194	127196526	+	#036A9C	7415114	7789332	280461	27746	280461	7695575	false
	HSA 2	74581674	76828085	127252616	129483268	+	#036A9C	2246411	2230652	27746	76040	27746	2274157	false
	HSA 2	76904125	77271187	129674971	129920729	+	#036A9C	367062	245758	76040	88651	76040	443102	false
	HSA 2	77359838	77836041	130720203	131157934	-	#036A9C	476203	437731	88651	191187	88651	564854	false
	HSA 2	78027228	78100483	130513889	130566559	+	#036A9C	73255	52670	191187	888	191187	264442	false
	HSA 2	78101371	78198288	130336097	130387596	-	#036A9C	96917	51499	888		888	97805	false
	HSA 17	78351017	81963280	69240889	73209171	+	#FF0000	3612263	3968282	11312482		11312482	14924745	false
**CPOR 19**	HSA 5	677957	3008535	138188172	140728751	+	#F33071	2330578	2540579		96403			
	HSA 5	3104938	3552964	140763782	141195045	+	#F33071	448026	431263	96403	-32326	96403	544429	false
	HSA 5	3520638	7852499	141190487	145713965	+	#F33071	4331861	4523478	-32326	60279	-32326	4299535	false
	HSA 5	7912778	11138954	145731223	149106581	+	#F33071	3226176	3375358	60279	-53853	60279	3286455	false
	HSA 5	11085101	12655975	149084276	150809278	+	#F33071	1570874	1725002	-53853	196914	-53853	1517021	false
	HSA 5	12852889	12924115	150910424	150986473	+	#F33071	71226	76049	196914	140	196914	268140	false
	HSA 5	12924255	21077608	122720666	131070339	-	#F33071	8153353	8349673	140	53325	140	8153493	false
	HSA 5	21130933	21918254	121849424	122720613	-	#F33071	787321	871189	53325		53325	840646	false
	HSA 13	22670890	23103105	24611094	24925161	+	#00FFFF	432215	314067		6826			
	HSA 13	23109931	24577741	19633117	21187939	+	#00FFFF	1467810	1554822	6826	7139	6826	1474636	false
	HSA 13	24584880	25954432	21372560	22911586	+	#00FFFF	1369552	1539026	7139	18371	7139	1376691	false
	HSA 13	25972803	26699353	23006197	23895791	+	#00FFFF	726550	889594	18371	0	18371	744921	false
	HSA 13	26699353	27032516	23957611	24317249	-	#00FFFF	333163	359638	0	-33256	0	333163	false
	HSA 13	26999260	27968472	25055557	26098100	+	#00FFFF	969212	1042543	-33256	24419	-33256	935956	false
	HSA 13	27992891	39543343	26178210	38330384	+	#00FFFF	11550452	12152174	24419	61973	24419	11574871	false
	HSA 13	39605316	40108138	38330384	38889063	+	#00FFFF	502822	558679	61973	0	61973	564795	false
	HSA 13	40108138	41834605	38953825	40871558	+	#00FFFF	1726467	1917733	0	-113629	0	1726467	false

	HSA 13	41720976	41912215	52476596	52637533	-	#00FFFF	191239	160937	-113629	-191239	-113629	77610	false
	HSA 13	41720976	42009617	52175550	52476575	+	#00FFFF	288641	301025	-191239	17968	-191239	97402	false
	HSA 13	42027585	42872963	51165936	52160021	-	#00FFFF	845378	994085	17968	81521	17968	863346	false
	HSA 13	42954484	43097018	61839788	61958664	-	#00FFFF	142534	118876	81521	97199	81521	224055	false
	HSA 13	43194217	48681968	55711202	61839788	-	#00FFFF	5487751	6128586	97199	356	97199	5584950	false
	HSA 13	48682324	48895144	55495802	55709941	+	#00FFFF	212820	214139	356	0	356	213176	false
	HSA 13	48895144	49552048	54752832	55495495	-	#00FFFF	656904	742663	0	21260	0	656904	false
	HSA 13	49573308	51566118	52651976	54686818	-	#00FFFF	1992810	2034842	21260	-14053	21260	2014070	false
	HSA 13	51552065	51688297	40907108	41088894	+	#00FFFF	136232	181786	-14053	66276	-14053	122179	false
	HSA 13	51754573	51810644	41186040	41264708	+	#00FFFF	56071	78668	66276	20116	66276	122347	false
	HSA 13	51830760	55600097	41315601	45376501	+	#00FFFF	3769337	4060900	20116	21	20116	3789453	false
	HSA 13	55600118	55670584	45384805	45456302	-	#00FFFF	70466	71497	21	477	21	70487	false
	HSA 13	55671061	56500893	45464383	46402820	+	#00FFFF	829832	938437	477	218	477	830309	false
	HSA 13	56501111	61080017	46468944	51136864	+	#00FFFF	4578906	4667920	218		218	4579124	false
**CPOR 2**	HSA 5	157227	382040	102087351	102389145	+	#F33071	224813	301794		522			
	HSA 5	382562	5446250	102130738	107849966	+	#F33071	5063688	5719228	522	934	522	5064210	false
	HSA 5	5447184	6987765	109144667	110898309	+	#F33071	1540581	1753642	934	53426	934	1541515	false
	HSA 5	7041191	8870339	110917756	112940839	+	#F33071	1829148	2023083	53426	8	53426	1882574	false
	HSA 5	8870347	9141098	137889288	138188172	+	#F33071	270751	298884	8	1128084	8	270759	false
	HSA 1	9342588	9553006	247370354	247494520	+	#00FF7F	210418	124166		-34640			
	HSA 1	9518366	9595029	247495713	247620680	+	#00FF7F	76663	124967	-34640	245076	-34640	42023	false
	HSA 1	9840105	9912384	247647828	247742255	-	#00FF7F	72279	94427	245076	24228	245076	317355	false
	HSA 1	9936612	10006163	247923353	248025250	-	#00FF7F	69551	101897	24228	18646603	24228	93779	false
	HSA 5	10269182	11076621	101068609	102055469	-	#F33071	807439	986860	1128084	44037	1128084	1935523	false
	HSA 5	11120658	11485168	100534236	100996474	-	#F33071	364510	462238	44037	64608	44037	408547	false
	HSA 5	11549776	11620018	100401986	100468206	-	#F33071	70242	66220	64608	95670	64608	134850	false
	HSA 5	11715688	12429616	99582583	100374684	+	#F33071	713928	792101	95670	-70202	95670	809598	false
	HSA 5	12359414	12599420	99237625	99467738	-	#F33071	240006	230113	-70202	55220	-70202	169804	false
	HSA 5	12654640	19647769	91253258	99236845	-	#F33071	6993129	7983587	55220	0	55220	7048349	false
	HSA 5	19647769	28424237	80116042	91193899	-	#F33071	8776468	11077857	0		0	8776468	false
	HSA 1	28652766	28741563	248689020	248779635	+	#00FF7F	88797	90615	18646603	2172	18646603	18735400	false
	HSA 1	28743735	29003940	248701129	248918969	+	#00FF7F	260205	217840	2172	369342	2172	262377	false

HSA 1	29373282	29450388	248398563	248459786	+	#00FF7F	77106	61223	369342	185340	369342	446448	false
HSA 1	29635728	29732159	247647828	247742255	+	#00FF7F	96431	94427	185340	695675	185340	281771	false
HSA 1	30427834	30521828	247915959	248048153	+	#00FF7F	93994	132194	695675	95555	695675	789669	false
HSA 1	30617383	30675271	247964201	248048153	-	#00FF7F	57888	83952	95555	722696	95555	153443	false
HSA 1	31397967	31578150	247883067	248061956	+	#00FF7F	180183	178889	722696	88828	722696	902879	false
HSA 1	31666978	31761232	247922814	248100738	-	#00FF7F	94254	177924	88828	280131	88828	183082	false
HSA 1	32041363	32097859	247919898	248048153	+	#00FF7F	56496	128255	280131	20409	280131	336627	false
HSA 1	32118268	32201114	248179503	248278861	-	#00FF7F	82846	99358	20409	24309	20409	103255	false
HSA 1	32225423	32294701	247919898	248047983	-	#00FF7F	69278	128085	24309		24309	93587	false
HSA 4	32767307	33005050	73671859	73926154	+	#7FFFFF	237743	254295		44132			
HSA 4	33049182	34122097	74157989	75567434	+	#7FFFFF	1072915	1409445	44132		44132	1117047	false
HSA 10	34241568	34293098	94752636	94854626	+	#82C90F	51530	101990		-51530			
HSA 10	34241568	34359340	94921778	95011018	+	#82C90F	117772	89240	-51530	-113667	-51530	66242	false
HSA 10	34245673	34370748	94680127	94852724	+	#82C90F	125075	172597	-113667	54415	-113667	11408	false
HSA 10	34425163	34529141	95234926	95342175	+	#82C90F	103978	107249	54415	42469	54415	158393	false
HSA 10	34571610	35972802	95311393	96790965	+	#82C90F	1401192	1479572	42469	61036	42469	1443661	false
HSA 10	36033838	39423303	96792477	100371138	+	#82C90F	3389465	3578661	61036	123540	61036	3450501	false
HSA 10	39546843	39640968	133507296	133561533	-	#82C90F	94125	54237	123540	502550	123540	217665	false
HSA 10	40143518	71347568	100308646	133423664	-	#82C90F	31204050	33115018	502550	-20977848	502550	31706600	false
HSA 10	50369720	50442266	122879182	122955632	+	#82C90F	72546	76450	-20977848	13019110	-20977848	-20905302	true
HSA 9	71654297	71865989	87488206	87719406	-	#819EF7	211692	231200		433			
HSA 9	71866422	71945913	87416183	87488185	+	#819EF7	79491	72002	433	0	433	79924	false
HSA 9	71945913	73140010	86204961	87409495	-	#819EF7	1194097	1204534	0	29529	0	1194097	false
HSA 9	73169539	73359348	85857441	86102895	-	#819EF7	189809	245454	29529	1861	29529	219338	false
HSA 9	73361209	76065515	83210354	85748005	-	#819EF7	2704306	2537651	1861		1861	2706167	false
HSA 7	76437412	76585157	87503157	87615866	+	#7F00FF	147745	112709	67121314	16656	67121314	67269059	false
HSA 7	76601813	79239582	87503815	90106200	+	#7F00FF	2637769	2602385	16656	91891	16656	2654425	false
HSA 7	79331473	84430031	90162634	95734353	+	#7F00FF	5098558	5571719	91891	68422	91891	5190449	false
HSA 7	84498453	86579440	95756775	97873787	+	#7F00FF	2080987	2117012	68422	91163	68422	2149409	false
HSA 8	86448284	86558073	46696874	46770344	-	#80797D	109789	73470		-95206			
HSA 8	86462867	86585684	46544751	46623790	+	#80797D	122817	79039	-95206		-95206	27611	false
HSA 7	86670603	91646774	7021513	12506036	+	#7F00FF	4976171	5484523	91163	17	91163	5067334	false

	HSA 7	91646791	97569998	112497276	118786956	+	#7F00FF	5923207	6289680	17	93950	17	5923224	false
	HSA 7	97663948	97992493	118845813	119245267	+	#7F00FF	328545	399454	93950	1564	93950	422495	false
	HSA 7	97994057	98080941	119245287	119343181	-	#7F00FF	86884	97894	1564	2352	1564	88448	false
	HSA 7	98083293	106444882	119343197	128460230	+	#7F00FF	8361589	9117033	2352	21654	2352	8363941	false
	HSA 7	106466536	111827622	128663903	134631312	+	#7F00FF	5361086	5967409	21654	109101	21654	5382740	false
	HSA 7	111936723	114369556	29744111	32480205	-	#7F00FF	2432833	2736094	109101	-6724	109101	2541934	false
	HSA 7	114362832	120714822	22541386	29648663	-	#7F00FF	6351990	7107277	-6724	0	-6724	6345266	false
	HSA 7	120714822	129379159	12520581	22489340	-	#7F00FF	8664337	9968759	0	78	0	8664337	false
	HSA 7	129379237	137379989	103289099	112497210	-	#7F00FF	8000752	9208111	78	33671	78	8000830	false
	HSA 7	137413660	137755151	102691867	103150919	-	#7F00FF	341491	459052	33671	-103762	33671	375162	false
	HSA 7	137651389	144822872	77069779	86044390	+	#7F00FF	7171483	8974611	-103762	184	-103762	7067721	false
	HSA 7	144823056	145021299	86045335	86372744	-	#7F00FF	198243	327409	184	160	184	198427	false
	HSA 7	145021459	145753126	86374119	87445826	+	#7F00FF	731667	1071707	160		160	731827	false
**CPOR 20**	HSA 6	256885	356314	22357433	22448101	-	#EBE47D	99429	90668		69693			
	HSA 6	426007	532107	22270411	22358578	-	#EBE47D	106100	88167	69693	254785	69693	175793	false
	HSA 6	786892	1021724	22284524	22409968	-	#EBE47D	234832	125444	254785	-36235	254785	489617	false
	HSA 6	985489	1090294	22271682	22409968	-	#EBE47D	104805	138286	-36235	1067655	-36235	68570	false
	HSA 6	2157949	2257480	28430729	28593341	-	#EBE47D	99531	162612	1067655	20335	1067655	1167186	false
	HSA 6	2277815	2434504	28068075	28366930	-	#EBE47D	156689	298855	20335	5528	20335	177024	false
	HSA 6	2440032	2567218	27837861	28005009	-	#EBE47D	127186	167148	5528	-35637	5528	132714	false
	HSA 6	2531581	2586655	27783618	27839142	-	#EBE47D	55074	55524	-35637	34368	-35637	19437	false
	HSA 6	2621023	2735931	27420127	27620480	-	#EBE47D	114908	200353	34368	63688	34368	149276	false
	HSA 6	2799619	2868632	27023078	27167005	-	#EBE47D	69013	143927	63688	880157	63688	132701	false
	HSA 6	3748789	5912724	181356	2935662	+	#EBE47D	2163935	2754306	880157	211923	880157	3044092	false
	HSA 6	6124647	8003262	2871576	5016062	+	#EBE47D	1878615	2144486	211923	25874	211923	2090538	false
	HSA 6	8029136	8647269	5067037	5805913	+	#EBE47D	618133	738876	25874	32027	25874	644007	false
	HSA 6	8679296	26211870	5857107	26000382	+	#EBE47D	17532574	20143275	32027	87218	32027	17564601	false
	HSA 6	26299088	26535434	25956299	26200061	+	#EBE47D	236346	243762	87218	-63846	87218	323564	false
	HSA 6	26471588	26599024	26199516	26436739	+	#EBE47D	127436	237223	-63846	470220	-63846	63590	false
	HSA 6	27069244	27262690	26382248	26664028	+	#EBE47D	193446	281780	470220		470220	663666	false
	HSA 1	27470819	27646102	228661429	229163594	+	#00FF7F	175283	502165		101			
	HSA 1	27646203	31238646	229216393	234490100	+	#00FF7F	3592443	5273707	101	12	101	3592544	false

	HSA 1	31238658	31695527	234491513	235094180	-	#00FF7F	456869	602667	12	1312	12	456881	false
	HSA 1	31696839	35694920	235099440	239931861	+	#00FF7F	3998081	4832421	1312		1312	3999393	false
	HSA 10	35729009	37937599	88592	2260811	+	#82C90F	2208590	2172219		10750			
	HSA 10	37948349	38109655	2262432	2409997	-	#82C90F	161306	147565	10750	3129	10750	172056	false
	HSA 10	38112784	38907718	2411635	3194890	+	#82C90F	794934	783255	3129	30936	3129	798063	false
	HSA 10	38938654	45256559	5407677	12093571	-	#82C90F	6317905	6685894	30936		30936	6348841	false
	HSA 3	45624174	46073019	126002140	126547448	-	#02BCB7	448845	545308		92			
	HSA 3	46073111	54709391	66213651	75243253	+	#02BCB7	8636280	9029602	92	-31087	92	8636372	false
	HSA 3	54678304	60033084	12066	5160572	+	#02BCB7	5354780	5148506	-31087	106791	-31087	5323693	false
	HSA 3	60139875	61540048	5106937	6326628	+	#02BCB7	1400173	1219691	106791	88740	106791	1506964	false
	HSA 3	61628788	63763684	6378865	8442826	+	#02BCB7	2134896	2063961	88740	27027	88740	2223636	false
	HSA 3	63790711	68960963	32684946	38138616	-	#02BCB7	5170252	5453670	27027		27027	5197279	false
**CPOR 21**	HSA 17	375322	881789	19768463	20319402	+	#FF0000	506467	550939		-97127			
	HSA 17	784662	1548981	15826087	16654105	+	#FF0000	764319	828018	-97127	20543	-97127	667192	false
	HSA 17	1569524	6198791	10672341	15708963	-	#FF0000	4629267	5036622	20543	20639	20543	4649810	false
	HSA 17	6219430	6986384	9557364	10455934	+	#FF0000	766954	898570	20639	-32872	20639	787593	false
	HSA 17	6953512	7236023	10455371	10733562	+	#FF0000	282511	278191	-32872	2498	-32872	249639	false
	HSA 17	7238521	8445739	8194861	9631383	+	#FF0000	1207218	1436522	2498	42227	2498	1209716	false
	HSA 17	8487966	9733726	6871735	8194777	-	#FF0000	1245760	1323042	42227	3217	42227	1287987	false
	HSA 17	9736943	10162506	4631636	5115377	+	#FF0000	425563	483741	3217	4418	3217	428780	false
	HSA 17	10166924	10540324	5179568	5510359	+	#FF0000	373400	330791	4418	1060686	4418	377818	false
	HSA 17	11601010	11933348	75205155	75579105	-	#FF0000	332338	373950	1060686	177325	1060686	1393024	false
	HSA 17	12110673	13022666	75579141	76594775	-	#FF0000	911993	1015634	177325	44724	177325	1089318	false
	HSA 17	13067390	13138699	46246317	46343415	+	#FF0000	71309	97098	44724	24783	44724	116033	false
	HSA 17	13163482	13248726	75164125	75217534	+	#FF0000	85244	53409	24783	83276	24783	110027	false
	HSA 17	13332002	15148939	73192959	75209608	-	#FF0000	1816937	2016649	83276	73365	83276	1900213	false
	HSA 17	15222304	15336781	73069629	73209171	-	#FF0000	114477	139542	73365	115634	73365	187842	false
	HSA 17	15452415	18139478	76622847	80120974	+	#FF0000	2687063	3498127	115634	66661	115634	2802697	false
	HSA 17	18206139	19549857	80121187	81735207	+	#FF0000	1343718	1614020	66661	16	66661	1410379	false
	HSA 17	19549873	20097668	81800892	82354930	+	#FF0000	547795	554038	16	63215	16	547811	false
	HSA 17	20160883	20889258	82355184	83231424	+	#FF0000	728375	876240	63215	730082	63215	791590	false
	HSA 11	21180875	21236579	56971719	57035309	-	#0000FF	55704	63590	10542529	14482	10542529	10598233	false

	HSA 11	21251061	21324773	56748219	56824200	-	#0000FF	73712	75981	14482	33385	14482	88194	false
	HSA 11	21358158	21418037	56717452	56772582	-	#0000FF	59879	55130	33385		33385	93264	false
	HSA 17	21619340	21941799	21099081	21466836	-	#FF0000	322459	367755	730082	47853	730082	1052541	false
	HSA 17	21989652	22078289	20967434	21046646	-	#FF0000	88637	79212	47853	-68086	47853	136490	false
	HSA 17	22010203	23331288	16919177	18364610	+	#FF0000	1321085	1445433	-68086	18099	-68086	1252999	false
	HSA 17	23349387	23716824	46607044	47003557	-	#FF0000	367437	396513	18099	17879	18099	385536	false
	HSA 17	23734703	24270464	45632770	46241243	+	#FF0000	535761	608473	17879	-48065	17879	553640	false
	HSA 17	24222399	24485602	47090397	47335354	+	#FF0000	263203	244957	-48065	122901	-48065	215138	false
	HSA 17	24608503	24659422	72972437	73034645	+	#FF0000	50919	62208	122901	132539	122901	173820	false
	HSA 17	24791961	27102203	62366674	64758892	+	#FF0000	2310242	2392218	132539	-111803	132539	2442781	false
	HSA 17	26990400	29819576	64969422	68113639	-	#FF0000	2829176	3144217	-111803	-18029	-111803	2717373	false
	HSA 17	29801547	30508655	68231636	69146693	+	#FF0000	707108	915057	-18029	139473	-18029	689079	false
	HSA 17	30648128	31068370	19194147	19755354	-	#FF0000	420242	561207	139473	-10982	139473	559715	false
	HSA 17	31057388	31242615	18856647	19072871	-	#FF0000	185227	216224	-10982		-10982	174245	false
**CPOR 22**	HSA 12	8126	908746	6281416	7221824	-	#8D3B0C	900620	940408		53824			
	HSA 12	962570	4213966	2844576	6278419	+	#8D3B0C	3251396	3433843	53824	370083	53824	3305220	false
	HSA 10	4376907	4436292	18536588	18664600	-	#82C90F	59385	128012		21867			
	HSA 10	4458159	4511182	20833833	20891417	-	#82C90F	53023	57584	21867	3798659	21867	74890	false
	HSA 12	4584049	4655538	2788219	2844599	-	#8D3B0C	71489	56380	370083	23583478	370083	441572	false
	HSA 3	4810356	5035136	12537148	12842398	+	#02BCB7	224780	305250		-5838			
	HSA 3	5029298	5456788	129395050	129994482	+	#02BCB7	427490	599432	-5838	577	-5838	421652	false
	HSA 3	5457365	6068790	14109499	15102688	-	#02BCB7	611425	993189	577	32807	577	612002	false
	HSA 3	6101597	7914244	126567248	128816833	+	#02BCB7	1812647	2249585	32807	3942998	32807	1845454	false
	HSA 10	8309841	8421172	27127696	27223115	+	#82C90F	111331	95419	3798659	588958	3798659	3909990	false
	HSA 10	9010130	9399818	15411024	15871694	+	#82C90F	389688	460670	588958	84973	588958	978646	false
	HSA 10	9484791	11797896	15892798	18663949	+	#82C90F	2313105	2771151	84973	306007	84973	2398078	false
	HSA 10	12103903	12200135	21276401	21443230	-	#82C90F	96232	166829	306007	48656	306007	402239	false
	HSA 10	12248791	17396251	18664765	24560351	+	#82C90F	5147460	5895586	48656	82848	48656	5196116	false
	HSA 10	17479099	17533468	15868621	15934347	+	#82C90F	54369	65726	82848	2688778	82848	137217	false
	HSA 3	17566294	18295166	12897726	13897637	+	#02BCB7	728872	999911	5551155	404385	5551155	6280027	false
	HSA 3	18699551	19228541	11929980	12533168	-	#02BCB7	528990	603188	404385	277	404385	933375	false
	HSA 3	19228818	20017799	10753986	11855486	-	#02BCB7	788981	1101500	277		277	789258	false

	HSA 10	20222246	21994500	24574069	26622661	+	#82C90F	1772254	2048592	2688778	-61145	2688778	4461032	false
	HSA 10	21933355	22177271	26633506	26872931	+	#82C90F	243916	239425	-61145	8779	-61145	182771	false
	HSA 10	22186050	22282258	27148213	27242291	-	#82C90F	96208	94078	8779	221968	8779	104987	false
	HSA 10	22504226	24437084	3213662	4815017	-	#82C90F	1932858	1601355	221968	103289	221968	2154826	false
	HSA 10	24540373	24627071	4815017	4893552	+	#82C90F	86698	78535	103289	102024	103289	189987	false
	HSA 10	24729095	24838971	4922181	5007637	+	#82C90F	109876	85456	102024	-60018	102024	211900	false
	HSA 10	24778953	24911313	5096796	5234018	+	#82C90F	132360	137222	-60018	-102972	-60018	72342	false
	HSA 10	24808341	24913243	4995322	5051978	-	#82C90F	104902	56656	-102972	1421888	-102972	1930	false
	HSA 5	25153681	25391765	107846821	108125720	-	#F33071	238084	278899		424			
	HSA 5	25392189	26335048	108125774	109228131	-	#F33071	942859	1102357	424		424	943283	false
	HSA 10	26335131	26440086	5299719	5382295	+	#82C90F	104955	82576	1421888		1421888	1526843	false
	HSA 12	28239016	28313549	7377198	7602483	+	#8D3B0C	74533	225285	23583478	254458	23583478	23658011	false
	HSA 12	28568007	28657546	8452104	8543743	-	#8D3B0C	89539	91639	254458	310524	254458	343997	false
	HSA 12	28968070	29044435	31124719	31211945	-	#8D3B0C	76365	87226	310524	48040	310524	386889	false
	HSA 12	29092475	29192433	9052994	9113797	+	#8D3B0C	99958	60803	48040	24551	48040	147998	false
	HSA 12	29216984	29380303	9024508	9134785	-	#8D3B0C	163319	110277	24551	62782	24551	187870	false
	HSA 12	29443085	29799507	8598055	9020280	-	#8D3B0C	356422	422225	62782	62993	62782	419204	false
	HSA 12	29862500	29957755	7648306	7744892	+	#8D3B0C	95255	96586	62993	108039	62993	158248	false
	HSA 12	30065794	30124491	8599732	8701109	-	#8D3B0C	58697	101377	108039	2501	108039	166736	false
	HSA 12	30126992	30196938	7883026	7933597	-	#8D3B0C	69946	50571	2501	16269	2501	72447	false
	HSA 12	30213207	30272352	8598055	8700584	+	#8D3B0C	59145	102529	16269	160486	16269	75414	false
	HSA 12	30432838	30491752	7649305	7744892	-	#8D3B0C	58914	95587	160486	39219	160486	219400	false
	HSA 12	30530971	30756593	7919856	8142244	+	#8D3B0C	225622	222388	39219	53610	39219	264841	false
	HSA 12	30810203	31376251	10495163	10852034	-	#8D3B0C	566048	356871	53610	220325	53610	619658	false
	HSA 12	31596576	31647054	10446346	10510559	-	#8D3B0C	50478	64213	220325	65788	220325	270803	false
	HSA 12	31712842	31901969	10495163	10596865	-	#8D3B0C	189127	101702	65788	53480	65788	254915	false
	HSA 12	31955449	32042463	10370743	10498493	-	#8D3B0C	87014	127750	53480	81367	53480	140494	false
	HSA 12	32123830	32464960	9934875	10315710	-	#8D3B0C	341130	380835	81367	487649	81367	422497	false
	HSA 12	32952609	33033876	10937136	11003047	+	#8D3B0C	81267	65911	487649	-10653	487649	568916	false
	HSA 12	33023223	33075608	11069358	11195940	+	#8D3B0C	52385	126582	-10653	283954	-10653	41732	false
	HSA 12	33359562	33414586	11264907	11349392	+	#8D3B0C	55024	84485	283954	-55024	283954	338978	false
	HSA 12	33359562	36107717	11350806	14213249	+	#8D3B0C	2748155	2862443	-55024	82305	-55024	2693131	false

	HSA 12	36190022	39357353	14237648	17764355	+	#8D3B0C	3167331	3526707	82305	49206	82305	3249636	false
	HSA 12	39406559	40963150	17861561	19790841	+	#8D3B0C	1556591	1929280	49206	15163	49206	1605797	false
	HSA 12	40978313	41091900	19790841	19898974	-	#8D3B0C	113587	108133	15163	11864	15163	128750	false
	HSA 12	41103764	42022164	19898979	20918563	+	#8D3B0C	918400	1019584	11864	-49666	11864	930264	false
	HSA 12	41972498	42029970	20958905	21106474	+	#8D3B0C	57472	147569	-49666	1909	-49666	7806	false
	HSA 12	42031879	42326500	21014046	21430338	-	#8D3B0C	294621	416292	1909	-179792	1909	296530	false
	HSA 12	42146708	42338964	20808944	21120620	-	#8D3B0C	192256	311676	-179792	-66751	-179792	12464	false
	HSA 12	42272213	42338964	21129731	21255700	-	#8D3B0C	66751	125969	-66751	0	-66751	0	true
	HSA 12	42338964	42439843	21248089	21306802	-	#8D3B0C	100879	58713	0	-50124	0	100879	false
	HSA 12	42389719	42555366	20879739	21090912	-	#8D3B0C	165647	211173	-50124	-163984	-50124	115523	false
	HSA 12	42391382	42555366	21049006	21239884	-	#8D3B0C	163984	190878	-163984	-47129	-163984	0	true
	HSA 12	42508237	42713063	21122014	21255700	-	#8D3B0C	204826	133686	-47129	-129801	-47129	157697	false
	HSA 12	42583262	42713063	20801307	20918477	-	#8D3B0C	129801	117170	-129801	9671	-129801	0	true
	HSA 12	42722734	44663963	21434629	23636528	+	#8D3B0C	1941229	2201899	9671	138252	9671	1950900	false
	HSA 12	44802215	47753260	23640620	27040704	+	#8D3B0C	2951045	3400084	138252		138252	3089297	false
	HSA 22	47792101	48211389	35656223	36130755	+	#F400E1	419288	474532		146679			
	HSA 22	48358068	48431580	36138812	36191110	-	#F400E1	73512	52298	146679	-73512	146679	220191	false
	HSA 22	48358068	48486175	36138808	36227448	-	#F400E1	128107	88640	-73512	-93811	-73512	54595	false
	HSA 22	48392364	48570317	36189595	36286952	-	#F400E1	177953	97357	-93811	234511	-93811	84142	false
	HSA 22	48804828	50626403	36256297	38600987	+	#F400E1	1821575	2344690	234511	88189	234511	2056086	false
	HSA 22	50714592	50952766	38639837	38942282	+	#F400E1	238174	302445	88189	30775	88189	326363	false
	HSA 22	50983541	53349301	39099384	42095238	+	#F400E1	2365760	2995854	30775	678231	30775	2396535	false
	HSA 22	54027532	54499331	42128633	42521931	+	#F400E1	471799	393298	678231	-21435	678231	1150030	false
	HSA 22	54477896	57337032	42548434	47363028	+	#F400E1	2859136	4814594	-21435	1460	-21435	2837701	false
	HSA 22	57338492	57434042	47363833	47479851	-	#F400E1	95550	116018	1460	447	1460	97010	false
	HSA 22	57434489	59845734	47488614	50783890	+	#F400E1	2411245	3295276	447		447	2411692	false
**CPOR 23**	HSA 18	241827	413243	13564957	13834038	-	#4E1A96	171416	269081		10125			
	HSA 18	423368	777293	12784673	13374070	-	#4E1A96	353925	589397	10125	1601	10125	364050	false
	HSA 18	778894	1011721	12247127	12727399	-	#4E1A96	232827	480272	1601	47385	1601	234428	false
	HSA 18	1059106	1199509	11678683	11911428	-	#4E1A96	140403	232745	47385	63	47385	187788	false
	HSA 18	1199572	1353074	11237419	11562202	-	#4E1A96	153502	324783	63	-40592	63	153565	false
	HSA 18	1312482	1689407	10663472	11235152	-	#4E1A96	376925	571680	-40592	378	-40592	336333	false

	HSA 18	1689785	8535568	1364762	10555928	-	#4E1A96	6845783	9191166	378	3351	378	6846161	false
	HSA 18	8538919	8724465	1158957	1360777	+	#4E1A96	185546	201820	3351	0	3351	188897	false
	HSA 18	8724465	9595017	112548	1147557	-	#4E1A96	870552	1035009	0	-12550	0	870552	false
	HSA 18	9582467	16859023	20947738	29579589	+	#4E1A96	7276556	8631851	-12550	55240	-12550	7264006	false
	HSA 18	16914263	17203913	29597053	29931017	+	#4E1A96	289650	333964	55240	6593	55240	344890	false
	HSA 18	17210506	17343395	29933687	30070950	-	#4E1A96	132889	137263	6593	6467	6593	139482	false
	HSA 18	17349862	18786827	30085757	31742879	+	#4E1A96	1436965	1657122	6467	95220	6467	1443432	false
	HSA 18	18882047	18950054	31693041	31785860	-	#4E1A96	68007	92819	95220	38128	95220	163227	false
	HSA 18	18988182	24428581	31796054	38137873	+	#4E1A96	5440399	6341819	38128	51229	38128	5478527	false
	HSA 18	24479810	30261927	38178057	44327037	+	#4E1A96	5782117	6148980	51229	4654	51229	5833346	false
	HSA 18	30266581	32621834	44381397	46959525	+	#4E1A96	2355253	2578128	4654	7901	4654	2359907	false
	HSA 18	32629735	34830365	47036742	49494341	+	#4E1A96	2200630	2457599	7901	73987	7901	2208531	false
	HSA 18	34904352	49589676	49510760	64082580	+	#4E1A96	14685324	14571820	73987	259	73987	14759311	false
	HSA 18	49589935	49660937	63970378	64084877	-	#4E1A96	71002	114499	259	18162	259	71261	false
	HSA 18	49679099	50499899	64916385	65651963	-	#4E1A96	820800	735578	18162	58303	18162	838962	false
	HSA 18	50558202	50871475	64521267	64870084	-	#4E1A96	313273	348817	58303	20006	58303	371576	false
	HSA 18	50891481	51768839	64304748	65260504	-	#4E1A96	877358	955756	20006	30307	20006	897364	false
	HSA 18	51799146	52036767	64114547	64338997	-	#4E1A96	237621	224450	30307	1674	30307	267928	false
	HSA 18	52038441	52414177	64084230	64479976	+	#4E1A96	375736	395746	1674	7095	1674	377410	false
	HSA 18	52421272	55222958	65363542	68075566	+	#4E1A96	2801686	2712024	7095	60020	7095	2808781	false
	HSA 18	55282978	58635496	68099193	71233344	+	#4E1A96	3352518	3134151	60020	16228	60020	3412538	false
	HSA 18	58651724	58734530	71234463	71292438	-	#4E1A96	82806	57975	16228	517	16228	99034	false
	HSA 18	58735047	64735606	71296780	77171495	+	#4E1A96	6000559	5874715	517	98868	517	6001076	false
	HSA 18	64834474	65177381	77228246	77535643	+	#4E1A96	342907	307397	98868	133643	98868	441775	false
	HSA 18	65311024	67939885	77537064	80047540	+	#4E1A96	2628861	2510476	133643	71701	133643	2762504	false
	HSA 18	68011586	68194102	80064936	80254828	+	#4E1A96	182516	189892	71701		71701	254217	false
**CPOR 24**	HSA 7	32230	300276	74444683	74852673	+	#7F00FF	268046	407990		-99013			
	HSA 7	201263	388388	74923630	75219536	-	#7F00FF	187125	295906	-99013	35309	-99013	88112	false
	HSA 7	423697	485685	67339922	67406208	+	#7F00FF	61988	66286	35309	153268	35309	97297	false
	HSA 7	638953	691328	48929451	49034667	-	#7F00FF	52375	105216	153268	119812	153268	205643	false
	HSA 7	811140	1147714	68489691	68927487	+	#7F00FF	336574	437796	119812	33207	119812	456386	false
	HSA 7	1180921	1250833	48418207	48522406	+	#7F00FF	69912	104199	33207	30716	33207	103119	false

	HSA 7	1281549	1373468	6108995	6171712	+	#7F00FF	91919	62717	30716	79901	30716	122635	false
	HSA 7	1453369	1543850	6108995	6181186	+	#7F00FF	90481	72191	79901	187817	79901	170382	false
	HSA 7	1731667	2058755	47645642	48244277	-	#7F00FF	327088	598635	187817	0	187817	514905	false
	HSA 7	2058755	3256932	45858243	47590041	-	#7F00FF	1198177	1731798	0	1	0	1198177	false
	HSA 7	3256933	3522828	45358149	45724561	-	#7F00FF	265895	366412	1	587	1	265896	false
	HSA 7	3523415	4305197	44043900	45282048	-	#7F00FF	781782	1238148	587	23	587	782369	false
	HSA 7	4305220	4394343	43866311	43960172	-	#7F00FF	89123	93861	23	66993	23	89146	false
	HSA 7	4461336	4630103	55795285	56114670	+	#7F00FF	168767	319385	66993	-63325	66993	235760	false
	HSA 7	4566778	4747323	65726831	65976373	+	#7F00FF	180545	249542	-63325	43653	-63325	117220	false
	HSA 7	4790976	4983795	66137929	66363875	+	#7F00FF	192819	225946	43653	41050	43653	236472	false
	HSA 7	5024845	5096606	66740247	66813390	+	#7F00FF	71761	73143	41050	13360	41050	112811	false
	HSA 7	5109966	5229601	66918434	67088460	+	#7F00FF	119635	170026	13360	90852	13360	132995	false
	HSA 7	5320453	9432522	67419641	72605335	-	#7F00FF	4112069	5185694	90852	117340	90852	4202921	false
	HSA 7	9549862	9621336	48418207	48522406	-	#7F00FF	71474	104199	117340	40944	117340	188814	false
	HSA 7	9662280	9719983	48881591	49027808	+	#7F00FF	57703	146217	40944	233288	40944	98647	false
	HSA 7	9953271	10817980	53991977	55314860	-	#7F00FF	864709	1322883	233288	7943	233288	1097997	false
	HSA 7	10825923	11209307	52584578	53064506	+	#7F00FF	383384	479928	7943	44150	7943	391327	false
	HSA 7	11253457	11563544	53872536	54341947	-	#7F00FF	310087	469411	44150	1856	44150	354237	false
	HSA 7	11565400	11721027	53189424	53382814	+	#7F00FF	155627	193390	1856	139815	1856	157483	false
	HSA 7	11860842	12272587	51611150	52143786	-	#7F00FF	411745	532636	139815	2211	139815	551560	false
	HSA 7	12274798	12812812	52565555	53183412	+	#7F00FF	538014	617857	2211	1404	2211	540225	false
	HSA 7	12814216	12868955	53755677	53861296	-	#7F00FF	54739	105619	1404	38362	1404	56143	false
	HSA 7	12907317	13244749	53189424	53677142	-	#7F00FF	337432	487718	38362	1856	38362	375794	false
	HSA 7	13246605	13340779	53872536	53991977	+	#7F00FF	94174	119441	1856	4492	1856	96030	false
	HSA 7	13345271	14122808	51449148	52582804	-	#7F00FF	777537	1133656	4492	20207	4492	782029	false
	HSA 7	14143015	16330459	48156789	51358487	-	#7F00FF	2187444	3201698	20207	8637	20207	2207651	false
	HSA 7	16339096	17010997	48704480	49600039	-	#7F00FF	671901	895559	8637	200225	8637	680538	false
	HSA 7	17211222	17431445	55327346	55586310	+	#7F00FF	220223	258964	200225	110493	200225	420448	false
	HSA 7	17541938	18318237	73286443	74440615	-	#7F00FF	776299	1154172	110493	35781	110493	886792	false
	HSA 7	18354018	18773058	75530458	76070349	+	#7F00FF	419040	539891	35781	0	35781	454821	false
	HSA 7	18773058	19015357	76178017	76516704	+	#7F00FF	242299	338687	0	23675	0	242299	false
	HSA 7	19039032	19165274	102387694	102526158	-	#7F00FF	126242	138464	23675	1268	23675	149917	false

	HSA 7	19166542	20732337	100338188	102326821	-	#7F00FF	1565795	1988633	1268	-37326	1268	1567063	false
	HSA 7	20695011	20785481	100138575	100221979	-	#7F00FF	90470	83404	-37326	219918	-37326	53144	false
	HSA 7	21005399	21306081	99927389	100140566	-	#7F00FF	300682	213177	219918	343535	219918	520600	false
	HSA 7	21649616	21828966	99723050	99870427	+	#7F00FF	179350	147377	343535	-122923	343535	522885	false
	HSA 7	21706043	21838542	99700835	99867990	-	#7F00FF	132499	167155	-122923	31210	-122923	9576	false
	HSA 7	21869752	21928798	99643365	99700852	+	#7F00FF	59046	57487	31210	55962	31210	90256	false
	HSA 7	21984760	22047381	99643365	99700968	-	#7F00FF	62621	57603	55962	-62621	55962	118583	false
	HSA 7	21984760	22087064	99680687	99737902	-	#7F00FF	102304	57215	-62621	-23431	-62621	39683	false
	HSA 7	22063633	22185492	99737490	99792765	+	#7F00FF	121859	55275	-23431	-121303	-23431	98428	false
	HSA 7	22064189	22200746	99700835	99800656	+	#7F00FF	136557	99821	-121303	-93782	-121303	15254	false
	HSA 7	22106964	22159365	99643365	99701098	+	#7F00FF	52401	57733	-93782	52543	-93782	-41381	true
	HSA 7	22211908	23772106	97974383	99702818	-	#7F00FF	1560198	1728435	52543	90938	52543	1612741	false
	HSA 7	23863044	24033900	5986263	6202448	+	#7F00FF	170856	216185	90938	96761	90938	261794	false
	HSA 7	24130661	24717455	6097183	6730960	+	#7F00FF	586794	633777	96761	12895	96761	683555	false
	HSA 7	24730350	24789391	6110980	6167006	-	#7F00FF	59041	56026	12895	73079	12895	71936	false
	HSA 7	24862470	25029271	2354077	2531271	-	#7F00FF	166801	177194	73079	89997	73079	239880	false
	HSA 7	25119268	25169877	6110793	6195656	-	#7F00FF	50609	84863	89997	97122	89997	140606	false
	HSA 7	25266999	25347218	2380071	2454608	+	#7F00FF	80219	74537	97122	209555	97122	177341	false
	HSA 7	25556773	27315942	2672800	4964859	-	#7F00FF	1759169	2292059	209555	-7013	209555	1968724	false
	HSA 7	27308929	27428447	2531271	2671725	+	#7F00FF	119518	140454	-7013	136505	-7013	112505	false
	HSA 7	27564952	27624328	4878943	4956361	+	#7F00FF	59376	77418	136505	12904	136505	195881	false
	HSA 7	27637232	27696058	6110980	6190056	-	#7F00FF	58826	79076	12904	26968	12904	71730	false
	HSA 7	27723026	28216819	5181242	5788368	+	#7F00FF	493793	607126	26968	96120	26968	520761	false
	HSA 7	28312939	28468156	5030316	5161137	-	#7F00FF	155217	130821	96120	126178	96120	251337	false
	HSA 7	28594334	29505894	1135229	2358128	-	#7F00FF	911560	1222899	126178	241998	126178	1037738	false
	HSA 7	29747892	30505989	31731	1141300	-	#7F00FF	758097	1109569	241998	35070	241998	1000095	false
	HSA 7	30541059	30592596	35224324	35299877	-	#7F00FF	51537	75553	35070	43	35070	86607	false
	HSA 7	30592639	31319026	35063866	35910181	+	#7F00FF	726387	846315	43	-700372	43	726430	false
	HSA 12	30612097	30749135	63557835	63725474	+	#8D3B0C	137038	167639		2739333			
	HSA 7	30618654	30710246	103182246	103288790	+	#7F00FF	91592	106544	-700372	587067	-700372	-608780	true
	HSA 7	31297313	33484609	35945323	38366616	+	#7F00FF	2187296	2421293	587067	3029	587067	2774363	false
	HSA 7	33487638	33550207	35051265	35185595	+	#7F00FF	62569	134330	3029	-60311	3029	65598	false

	HSA 12	33488468	33550207	63557835	63667932	+	#8D3B0C	61739	110097	2739333		2739333	2801072	false
	HSA 7	33489896	33551928	29651251	29742500	-	#7F00FF	62032	91249	-60311	473	-60311	1721	false
	HSA 7	33552401	34877080	38259713	39755333	+	#7F00FF	1324679	1495620	473	555	473	1325152	false
	HSA 7	34877635	38446644	39883585	43808010	+	#7F00FF	3569009	3924425	555	164312	555	3569564	false
	HSA 7	38610956	39459024	33128419	34154824	-	#7F00FF	848068	1026405	164312	188825	164312	1012380	false
	HSA 7	39647849	39841693	32867550	33110202	-	#7F00FF	193844	242652	188825	40	188825	382669	false
	HSA 7	39841733	39941927	32475945	32597071	-	#7F00FF	100194	121126	40	504996	40	100234	false
	HSA 7	40446923	41148796	34126674	34950485	+	#7F00FF	701873	823811	504996	46869	504996	1206869	false
	HSA 7	41195665	41254148	35007658	35076956	+	#7F00FF	58483	69298	46869	2070	46869	105352	false
	HSA 7	41256218	42029816	33128419	34154824	-	#7F00FF	773598	1026405	2070		2070	775668	false
	HSA 2	46464944	46529951	97509057	97585893	-	#036A9C	65007	76836		-65006			
	HSA 2	46464945	46541230	94801576	94854439	-	#036A9C	76285	52863	-65006	2400909	-65006	11279	false
	HSA 13	46466090	46541231	18821921	18874747	-	#00FFFF	75141	52826		-59850			
	HSA 10	46467129	46552384	37130714	37246935	+	#82C90F	85255	116221		19863			
	HSA 13	46481381	46552214	19265186	19339989	-	#00FFFF	70833	74803	-59850		-59850	10983	false
	HSA 10	46572247	46623603	37946834	38057630	-	#82C90F	51356	110796	19863	-50546	19863	71219	false
	HSA 10	46573057	46710583	37755779	37903698	-	#82C90F	137526	147919	-50546	1109	-50546	86980	false
	HSA 10	46711692	47078475	42784023	43462053	+	#82C90F	366783	678030	1109	9231	1109	367892	false
	HSA 10	47087706	47968481	43555587	45035319	+	#82C90F	880775	1479732	9231	323844	9231	890006	false
	HSA 10	48292325	48342648	45255232	45309346	-	#82C90F	50323	54114	323844	-21549	323844	374167	false
	HSA 10	48321099	48382593	45255231	45313638	+	#82C90F	61494	58407	-21549	287	-21549	39945	false
	HSA 10	48382880	48635153	45373845	45723178	+	#82C90F	252273	349333	287	-39582	287	252560	false
	HSA 10	48595571	48649017	45723861	45800807	-	#82C90F	53446	76946	-39582	-53446	-39582	13864	false
	HSA 10	48595571	48654311	50021997	50141381	-	#82C90F	58740	119384	-53446	14201	-53446	5294	false
	HSA 10	48668512	48718556	46035400	46087845	-	#82C90F	50044	52445	14201	223587	14201	64245	false
	HSA 2	48942139	49037952	207710668	207760821	-	#036A9C	95813	50153	2400909		2400909	2496722	false
	HSA 10	48942143	49037864	42403746	42455418	+	#82C90F	95721	51672	223587	375629	223587	319308	false
	HSA 10	49413493	49544071	45876825	46035758	-	#82C90F	130578	158933	375629	-100678	375629	506207	false
	HSA 10	49443393	50773785	48063576	49969746	-	#82C90F	1330392	1906170	-100678	-80993	-100678	1229714	false
	HSA 10	50692792	51125810	46877084	47454439	+	#82C90F	433018	577355	-80993	-73964	-80993	352025	false
	HSA 10	51051846	51176569	46232593	46375529	-	#82C90F	124723	142936	-73964	-124720	-73964	50759	false
	HSA 10	51051849	51265823	46232504	46453372	+	#82C90F	213974	220868	-124720	12050	-124720	89254	false

	HSA 10	51277873	51408979	46515015	46688793	+	#82C90F	131106	173778	12050	-95631	12050	143156	false
	HSA 10	51313348	51704950	86606666	87216735	-	#82C90F	391602	610069	-95631	2201	-95631	295971	false
	HSA 10	51707151	56542663	80258809	86529279	-	#82C90F	4835512	6270470	2201		2201	4837713	false
**CPOR 25**	HSA 1	524448	5260769	54903663	59881074	-	#00FF7F	4736321	4977411		65197			
	HSA 1	5325966	7082372	52925836	54891463	-	#00FF7F	1756406	1965627	65197	144763	65197	1821603	false
	HSA 1	7227135	10082974	49961816	52922694	-	#00FF7F	2855839	2960878	144763	35	144763	3000602	false
	HSA 1	10083009	11599256	48302172	49910560	-	#00FF7F	1516247	1608388	35	5406	35	1516282	false
	HSA 1	11604662	11783413	48205853	48307271	+	#00FF7F	178751	101418	5406	200122	5406	184157	false
	HSA 1	11983535	12924528	47173299	48112525	-	#00FF7F	940993	939226	200122	-21722	200122	1141115	false
	HSA 1	12902806	12962057	46843304	46900501	+	#00FF7F	59251	57197	-21722	113327	-21722	37529	false
	HSA 1	13075384	13166814	46929169	46996410	+	#00FF7F	91430	67241	113327	56014	113327	204757	false
	HSA 1	13222828	13292850	46926938	46996410	-	#00FF7F	70022	69472	56014	143227	56014	126036	false
	HSA 1	13436077	13491388	46926938	46996410	-	#00FF7F	55311	69472	143227	-843	143227	198538	false
	HSA 1	13490545	15082206	45226794	46824921	-	#00FF7F	1591661	1598127	-843	52653	-843	1590818	false
	HSA 1	15134859	15780675	42348740	43123531	+	#00FF7F	645816	774791	52653	289162	52653	698469	false
	HSA 1	16069837	16519037	43123633	43665323	+	#00FF7F	449200	541690	289162	29295	289162	738362	false
	HSA 1	16548332	16616423	43760754	43825376	-	#00FF7F	68091	64622	29295	367	29295	97386	false
	HSA 1	16616790	17015710	43781652	44132331	+	#00FF7F	398920	350679	367	76469	367	399287	false
	HSA 1	17092179	18008085	44181665	45121125	+	#00FF7F	915906	939460	76469	56077	76469	992375	false
	HSA 1	18064162	18117857	45169582	45226794	+	#00FF7F	53695	57212	56077	199531	56077	109772	false
	HSA 1	18317388	18443448	42192812	42348728	-	#00FF7F	126060	155916	199531	7974	199531	325591	false
	HSA 1	18451422	18546009	43657303	43764113	-	#00FF7F	94587	106810	7974	164287	7974	102561	false
	HSA 1	18710296	20218858	40541141	42192710	-	#00FF7F	1508562	1651569	164287	75728	164287	1672849	false
	HSA 1	20294586	22619262	37782933	40518181	-	#00FF7F	2324676	2735248	75728	-1496462	75728	2400404	false
	HSA 1	21122800	21211685	39734674	39797934	+	#00FF7F	88885	63260	-1496462	1418389	-1496462	-1407577	true
	HSA 1	22630074	22697333	43657303	43722629	-	#00FF7F	67259	65326	1418389	8266	1418389	1485648	false
	HSA 1	22705599	24945683	35169780	37771319	-	#00FF7F	2240084	2601539	8266	73880	8266	2248350	false
	HSA 1	25019563	33358487	25332583	35165572	-	#00FF7F	8338924	9832989	73880	-4308560	73880	8412804	false
	HSA 1	29049927	29404795	29642666	29960252	+	#00FF7F	354868	317586	-4308560	-184897	-4308560	-3953692	true
	HSA 1	29219898	29434767	30465095	30695474	-	#00FF7F	214869	230379	-184897	649	-184897	29972	false
	HSA 1	29435416	29512965	29672169	29745014	-	#00FF7F	77549	72845	649	-26555	649	78198	false
	HSA 1	29486410	29542503	30089132	30190141	+	#00FF7F	56093	101009	-26555	-56093	-26555	29538	false

	HSA 1	29486410	29583639	30487853	30639618	+	#00FF7F	97229	151765	-56093	-17002	-56093	41136	false
	HSA 1	29566637	29666319	29453730	29586183	-	#00FF7F	99682	132453	-17002	14413	-17002	82680	false
	HSA 1	29680732	29850073	30477206	30708342	+	#00FF7F	169341	231136	14413	-85649	14413	183754	false
	HSA 1	29764424	29973712	30199507	30452033	+	#00FF7F	209288	252526	-85649	-95332	-85649	123639	false
	HSA 1	29878380	29962824	29970674	30048348	-	#00FF7F	84444	77674	-95332	3389207	-95332	-10888	true
	HSA 1	33352031	36328782	21486598	25248074	-	#00FF7F	2976751	3761476	3389207	1366	3389207	6365958	false
	HSA 1	36330148	37449445	20055431	21387522	-	#00FF7F	1119297	1332091	1366	509964	1366	1120663	false
	HSA 1	37959409	40325752	16911951	20009083	-	#00FF7F	2366343	3097132	509964	645	509964	2876307	false
	HSA 1	40326397	42646505	13458482	16466235	-	#00FF7F	2320108	3007753	645	915690	645	2320753	false
	HSA 1	43562195	43681448	12658615	12762286	-	#00FF7F	119253	103671	915690	522189	915690	1034943	false
	HSA 1	44203637	44478791	12634873	12686202	-	#00FF7F	275154	51329	522189	168372	522189	797343	false
	HSA 1	44647163	47121552	8942009	12632099	-	#00FF7F	2474389	3690090	168372	7957	168372	2642761	false
	HSA 1	47129509	51172161	2780551	8881496	-	#00FF7F	4042652	6100945	7957	222	7957	4050609	false
	HSA 1	51172383	51774622	1699688	2647825	-	#00FF7F	602239	948137	222	-41214	222	602461	false
	HSA 1	51733408	52276457	903444	1683185	-	#00FF7F	543049	779741	-41214		-41214	501835	false
**CPOR 26**	HSA 6	229598	2568124	118313867	121619508	+	#EBE47D	2338526	3305641		32980			
	HSA 6	2601104	5248612	121676471	124873081	+	#EBE47D	2647508	3196610	32980	24853	32980	2680488	false
	HSA 6	5273465	7353818	124873084	127403458	-	#EBE47D	2080353	2530374	24853	41879	24853	2105206	false
	HSA 6	7395697	7513617	137648995	137849897	+	#EBE47D	117920	200902	41879	137511	41879	159799	false
	HSA 6	7651128	8969958	116673663	118345336	-	#EBE47D	1318830	1671673	137511	4532	137511	1456341	false
	HSA 6	8974490	9037991	116614588	116673663	+	#EBE47D	63501	59075	4532		4532	68033	false
	HSA 8	9071624	9647195	352195	1189282	+	#80797D	575571	837087		24677			
	HSA 8	9671872	10166913	1241630	2241498	+	#80797D	495041	999868	24677	109681	24677	519718	false
	HSA 8	10276594	12571276	2501939	5502550	+	#80797D	2294682	3000611	109681	276	109681	2404363	false
	HSA 8	12571552	12894034	5509747	5893311	-	#80797D	322482	383564	276	0	276	322758	false
	HSA 8	12894034	13694933	5893541	6878939	+	#80797D	800899	985398	0	65772	0	800899	false
	HSA 8	13760705	13887063	6868684	7024517	+	#80797D	126358	155833	65772	15464	65772	192130	false
	HSA 8	13902527	14676401	28386367	29276267	+	#80797D	773874	889900	15464	3556	15464	789338	false
	HSA 8	14679957	16328838	9828096	12102835	+	#80797D	1648881	2274739	3556	143579	3556	1652437	false
	HSA 8	16472417	16581159	7415858	7493036	-	#80797D	108742	77178	143579	-108742	143579	252321	false
	HSA 8	16472417	16616506	7818521	7930817	+	#80797D	144089	112296	-108742	48586	-108742	35347	false
	HSA 8	16665092	16753341	6960295	7024429	-	#80797D	88249	64134	48586	217333	48586	136835	false

	HSA 8	16970674	17656069	27526796	28417797	-	#80797D	685395	891001	217333	533	217333	902728	false
	HSA 8	17656602	17715912	39742779	39838258	+	#80797D	59310	95479	533	4518	533	59843	false
	HSA 8	17720430	21344210	23245178	27548288	-	#80797D	3623780	4303110	4518	70486	4518	3628298	false
	HSA 8	21414696	25500862	18334654	23046467	-	#80797D	4086166	4711813	70486	-122380	70486	4156652	false
	HSA 8	25378482	25530991	18133687	18226318	-	#80797D	152509	92631	-122380	24012799	-122380	30129	false
	HSA 4	25544846	27110385	162585130	164496606	+	#7FFFFF	1565539	1911476		503			
	HSA 4	27110888	27229935	164496606	164632174	-	#7FFFFF	119047	135568	503	126	503	119550	false
	HSA 4	27230061	27493063	164636228	164925857	+	#7FFFFF	263002	289629	126	-35116	126	263128	false
	HSA 4	27457947	29862903	165031305	167995182	+	#7FFFFF	2404956	2963877	-35116	-8902	-35116	2369840	false
	HSA 4	29854001	30204004	167522945	167992807	-	#7FFFFF	350003	469862	-8902	34006	-8902	341101	false
	HSA 4	30238010	30557291	167028601	167467868	-	#7FFFFF	319281	439267	34006	34869	34006	353287	false
	HSA 4	30592160	30674736	167995189	168100014	-	#7FFFFF	82576	104825	34869	116939	34869	117445	false
	HSA 4	30791675	30991144	168092501	168326546	+	#7FFFFF	199469	234045	116939	-117609	116939	316408	false
	HSA 4	30873535	32338774	168357859	170162539	+	#7FFFFF	1465239	1804680	-117609	20031	-117609	1347630	false
	HSA 4	32358805	36404037	170061972	174996264	+	#7FFFFF	4045232	4934292	20031	1845	20031	4065263	false
	HSA 4	36405882	36502998	175144322	175326176	+	#7FFFFF	97116	181854	1845	9729	1845	98961	false
	HSA 4	36512727	36607403	175385902	175542805	+	#7FFFFF	94676	156903	9729	54149	9729	104405	false
	HSA 4	36661552	38938550	175536259	178200566	+	#7FFFFF	2276998	2664307	54149	0	54149	2331147	false
	HSA 4	38938550	39093897	178206938	178375665	-	#7FFFFF	155347	168727	0	1863	0	155347	false
	HSA 4	39095760	49154690	178387950	189633532	+	#7FFFFF	10058930	11245582	1863	47787	1863	10060793	false
	HSA 4	49202477	49428813	189701133	189915207	+	#7FFFFF	226336	214074	47787		47787	274123	false
	HSA 8	49543790	54636822	12721538	18103888	-	#80797D	5093032	5382350	24012799	9042	24012799	29105831	false
	HSA 8	54645864	56047302	8243874	9787481	+	#80797D	1401438	1543607	9042	898	9042	1410480	false
	HSA 8	56048200	57427492	29326445	30625448	+	#80797D	1379292	1299003	898		898	1380190	false
**CPOR 27**	HSA 2	576	342126	218066625	218475084	-	#036A9C	341550	408459		-37590			
	HSA 2	304536	8602314	208598017	218058387	-	#036A9C	8297778	9460370	-37590	13449	-37590	8260188	false
	HSA 2	8615763	14633358	201778160	208499027	-	#036A9C	6017595	6720867	13449	677455	13449	6031044	false
	HSA 2	15310813	17870854	224746425	227648644	+	#036A9C	2560041	2902219	677455	-33602	677455	3237496	false
	HSA 2	17837252	20133588	227650354	230400016	+	#036A9C	2296336	2749662	-33602	-116511	-33602	2262734	false
	HSA 2	20017077	20761376	230369742	231536383	+	#036A9C	744299	1166641	-116511	25	-116511	627788	false
	HSA 2	20761401	21310811	231592498	232353496	+	#036A9C	549410	760998	25	-68019	25	549435	false
	HSA 2	21242792	27137331	232432735	240646317	+	#036A9C	5894539	8213582	-68019	1476	-68019	5826520	false

	HSA 2	27138807	27863192	240711434	241810090	+	#036A9C	724385	1098656	1476	130987	1476	725861	false
	HSA 2	27994179	28080366	241791840	241892126	+	#036A9C	86187	100286	130987		130987	217174	false
	HSA 14	28438372	28581470	20186768	20248826	+	#FFFF00	143098	62058		60038			
	HSA 14	28641508	28821913	20224727	20421800	-	#FFFF00	180405	197073	60038	9073	60038	240443	false
	HSA 14	28830986	29153566	20357843	20668724	+	#FFFF00	322580	310881	9073	181032	9073	331653	false
	HSA 14	29334598	29439311	20763593	20876119	-	#FFFF00	104713	112526	181032	131203	181032	285745	false
	HSA 14	29570514	30770910	20971411	21968679	+	#FFFF00	1200396	997268	131203	-367741	131203	1331599	false
	HSA 14	30403169	30612014	21866123	21924061	+	#FFFF00	208845	57938	-367741	-133977	-367741	-158896	true
	HSA 14	30478037	30771102	21700928	21844292	+	#FFFF00	293065	143364	-133977	-58093	-133977	159088	false
	HSA 14	30713009	30799631	22011737	22224989	+	#FFFF00	86622	213252	-58093	51822	-58093	28529	false
	HSA 14	30851453	30972511	22007487	22224989	+	#FFFF00	121058	217502	51822	-50306	51822	172880	false
	HSA 14	30922205	31124978	21829853	21968679	+	#FFFF00	202773	138826	-50306	-150037	-50306	152467	false
	HSA 14	30974941	31125189	21786884	21892088	+	#FFFF00	150248	105204	-150037	-88574	-150037	211	false
	HSA 14	31036615	31167539	22082800	22224989	+	#FFFF00	130924	142189	-88574	2617	-88574	42350	false
	HSA 14	31170156	31257435	21784546	21879039	+	#FFFF00	87279	94493	2617	-80000	2617	89896	false
	HSA 14	31177435	31271731	21911102	22003752	+	#FFFF00	94296	92650	-80000	-34273	-80000	14296	false
	HSA 14	31237458	31395002	22004927	22222779	+	#FFFF00	157544	217852	-34273	23345	-34273	123271	false
	HSA 14	31418347	31479506	21911102	22003752	+	#FFFF00	61159	92650	23345	63839	23345	84504	false
	HSA 14	31543345	31633957	21746434	21842674	+	#FFFF00	90612	96240	63839	-82728	63839	154451	false
	HSA 14	31551229	31661770	22038394	22133956	+	#FFFF00	110541	95562	-82728	31653	-82728	27813	false
	HSA 14	31693423	31828134	21748548	21847378	+	#FFFF00	134711	98830	31653	-76901	31653	166364	false
	HSA 14	31751233	31828134	21824034	21895187	+	#FFFF00	76901	71153	-76901	-71181	-76901	0	true
	HSA 14	31756953	32480483	21881404	22707227	+	#FFFF00	723530	825823	-71181	50358	-71181	652349	false
	HSA 14	32530841	33537367	22741515	23789898	+	#FFFF00	1006526	1048383	50358	163642	50358	1056884	false
	HSA 14	33701009	34467368	23899039	24636404	+	#FFFF00	766359	737365	163642		163642	930001	false
	HSA 1	34722178	36453560	209684464	211398034	-	#00FF7F	1731382	1713570		3123			
	HSA 1	36456683	36824731	241459285	241891115	+	#00FF7F	368048	431830	3123	845	3123	371171	false
	HSA 1	36825576	37302869	226907546	227463930	+	#00FF7F	477293	556384	845	2665	845	478138	false
	HSA 1	37305534	40575811	243113687	246962717	-	#00FF7F	3270277	3849030	2665	27989	2665	3272942	false
	HSA 1	40603800	41424640	241995645	243024826	-	#00FF7F	820840	1029181	27989	39332	27989	848829	false
	HSA 1	41463972	49577327	211512057	222447648	+	#00FF7F	8113355	10935591	39332	4576	39332	8152687	false
	HSA 1	49581903	50149778	222521051	223558898	+	#00FF7F	567875	1037847	4576	78	4576	572451	false

	HSA 1	50149856	50259589	223608972	223847471	+	#00FF7F	109733	238499	78	12074	78	109811	false
	HSA 1	50271663	50363707	224042288	224193005	+	#00FF7F	92044	150717	12074	80155	12074	104118	false
	HSA 1	50443862	50726178	224227122	224810562	+	#00FF7F	282316	583440	80155	26135	80155	362471	false
	HSA 1	50752313	51422768	224958368	225881306	+	#00FF7F	670455	922938	26135		26135	696590	false
**CPOR 28**	HSA 6	95584	235088	29548395	29683416	+	#EBE47D	139504	135021		12995			
	HSA 6	248083	488392	30049457	30380673	+	#EBE47D	240309	331216	12995	15470	12995	253304	false
	HSA 6	503862	580348	29956425	30009748	-	#EBE47D	76486	53323	15470	85805	15470	91956	false
	HSA 6	666153	723609	29887719	29959800	+	#EBE47D	57456	72081	85805	14400	85805	143261	false
	HSA 6	738009	1008112	30442316	30863034	-	#EBE47D	270103	420718	14400	159136	14400	284503	false
	HSA 6	1167248	1240615	30864059	30988418	+	#EBE47D	73367	124359	159136	-9762	159136	232503	false
	HSA 6	1230853	1310409	31034666	31181375	+	#EBE47D	79556	146709	-9762	10537	-9762	69794	false
	HSA 6	1320946	1395872	29956392	30009834	-	#EBE47D	74926	53442	10537	124363	10537	85463	false
	HSA 6	1520235	2104364	31523008	32230431	+	#EBE47D	584129	707423	124363	377106	124363	708492	false
	HSA 6	2481470	2579547	32290447	32373819	+	#EBE47D	98077	83372	377106	368802	377106	475183	false
	HSA 6	2948349	3025172	32461878	32533968	-	#EBE47D	76823	72090	368802	589642	368802	445625	false
	HSA 6	3614814	4339294	32380853	33088502	+	#EBE47D	724480	707649	589642	-375295	589642	1314122	false
	HSA 6	3963999	4033417	32630057	32701712	+	#EBE47D	69418	71655	-375295	352527	-375295	-305877	true
	HSA 6	4385944	4470095	33024727	33130192	-	#EBE47D	84151	105465	352527	99492	352527	436678	false
	HSA 6	4569587	5271538	33146900	34155072	+	#EBE47D	701951	1008172	99492	9638	99492	801443	false
	HSA 6	5281176	6926335	34225075	36549744	+	#EBE47D	1645159	2324669	9638	19758	9638	1654797	false
	HSA 6	6946093	8916029	36554428	39335376	-	#EBE47D	1969936	2780948	19758	4993	19758	1989694	false
	HSA 6	8921022	10430246	39315755	41229142	+	#EBE47D	1509224	1913387	4993	-25303	4993	1514217	false
	HSA 6	10404943	15123698	41271333	48795326	+	#EBE47D	4718755	7523993	-25303	309	-25303	4693452	false
	HSA 6	15124007	15196135	48885271	48943882	+	#EBE47D	72128	58611	309		309	72437	false
	HSA 10	15331373	15464651	80046125	80211745	+	#82C90F	133278	165620		20318			
	HSA 10	15484969	20441014	73741151	79498116	-	#82C90F	4956045	5756965	20318	6668	20318	4976363	false
	HSA 10	20447682	35499388	56285094	73659578	-	#82C90F	15051706	17374484	6668	-1376488	6668	15058374	false
	HSA 10	34122900	34246720	52929954	53065520	+	#82C90F	123820	135566	-1376488	-41178	-1376488	-1252668	true
	HSA 10	34205542	34269415	56738532	56834601	-	#82C90F	63873	96069	-41178	831416	-41178	22695	false
	HSA 10	35100831	35155331	57872554	57924078	-	#82C90F	54500	51524	831416	383323	831416	885916	false
	HSA 10	35538654	39922311	50788610	56209738	-	#82C90F	4383657	5421128	383323	56625	383323	4766980	false
	HSA 10	39978936	40403866	50194392	50655359	+	#82C90F	424930	460967	56625	0	56625	481555	false

	HSA 10	40403866	40635908	79715216	79794251	-	#82C90F	232042	79035	0	-174550	0	232042	false
	HSA 10	40461358	42828498	87431950	89836583	+	#82C90F	2367140	2404633	-174550	52101	-174550	2192590	false
	HSA 10	42880599	42961145	89865907	89987733	+	#82C90F	80546	121826	52101	42233	52101	132647	false
	HSA 10	43003378	43197187	90097358	90259669	-	#82C90F	193809	162311	42233	31515	42233	236042	false
	HSA 10	43228702	44676762	90237424	91721828	+	#82C90F	1448060	1484404	31515	1141047	31515	1479575	false
	HSA 10	45817809	48398397	91723032	94612562	-	#82C90F	2580588	2889530	1141047		1141047	3721635	false
	HSA 7	48463414	48523970	48297709	48381817	-	#7F00FF	60556	84108		6646			
	HSA 7	48530616	48758319	48381817	48672779	+	#7F00FF	227703	290962	6646		6646	234349	false
**CPOR 29**	HSA 14	29255	177476	55269884	55442914	+	#FFFF00	148221	173030		19202			
	HSA 14	196678	349496	55551102	55688960	-	#FFFF00	152818	137858	19202	46775	19202	172020	false
	HSA 14	396271	4662376	55660405	59999237	-	#FFFF00	4266105	4338832	46775	429	46775	4312880	false
	HSA 14	4662805	4863204	59863771	60175904	+	#FFFF00	200399	312133	429	160217	429	200828	false
	HSA 14	5023421	8000858	60183509	63099043	+	#FFFF00	2977437	2915534	160217	99013	160217	3137654	false
	HSA 14	8099871	8806654	63099045	63803000	+	#FFFF00	706783	703955	99013	62136	99013	805796	false
	HSA 14	8868790	11517537	63838815	66692778	+	#FFFF00	2648747	2853963	62136	16168	62136	2710883	false
	HSA 14	11533705	14893030	66754262	70290855	+	#FFFF00	3359325	3536593	16168	-31217	16168	3375493	false
	HSA 14	14861813	14942235	70320088	70423657	+	#FFFF00	80422	103569	-31217	64282	-31217	49205	false
	HSA 14	15006517	20579227	70581171	76685048	+	#FFFF00	5572710	6103877	64282	59024	64282	5636992	false
	HSA 14	20638251	34571667	76693157	92039424	+	#FFFF00	13933416	15346267	59024	-129538	59024	13992440	false
	HSA 14	34442129	34548860	91967763	92017823	+	#FFFF00	106731	50060	-129538	29627	-129538	-22807	true
	HSA 14	34578487	36712463	91962729	94374248	-	#FFFF00	2133976	2411519	29627	51434	29627	2163603	false
	HSA 14	36763897	38068214	94574932	96267779	-	#FFFF00	1304317	1692847	51434	6570	51434	1355751	false
	HSA 14	38074784	38273787	94363465	94524701	-	#FFFF00	199003	161236	6570	-30811	6570	205573	false
	HSA 14	38242976	41768844	96266380	100642507	+	#FFFF00	3525868	4376127	-30811	475558	-30811	3495057	false
	HSA 14	42244402	42634519	100642946	101079411	+	#FFFF00	390117	436465	475558	337713	475558	865675	false
	HSA 14	42972232	44417142	101082411	103157355	+	#FFFF00	1444910	2074944	337713	25707	337713	1782623	false
	HSA 14	44442849	45181631	103323363	104449762	+	#FFFF00	738782	1126399	25707	36052	25707	764489	false
	HSA 14	45217683	45851339	104507045	105531522	+	#FFFF00	633656	1024477	36052		36052	669708	false
**CPOR 3**	HSA 15	33042	374103	63482849	63886531	-	#EC83BA	341061	403682		107795			
	HSA 15	481898	585662	72454458	72600528	-	#EC83BA	103764	146070	107795	151	107795	211559	false
	HSA 15	585813	3015569	69293230	72378933	-	#EC83BA	2429756	3085703	151	27899381	151	2429907	false
	HSA 13	3254123	3361699	61952113	62014436	+	#00FFFF	107576	62323		92495			

HSA 13	3454194	4659097	62030206	63375214	+	#00FFFF	1204903	1345008	92495	976	92495	1297398	false
HSA 13	4660073	16328883	63474772	76109609	+	#00FFFF	11668810	12634837	976	300	976	11669786	false
HSA 13	16329183	16433127	76128018	76266715	-	#00FFFF	103944	138697	300	1557	300	104244	false
HSA 13	16434684	25315682	76268748	86202257	+	#00FFFF	8880998	9933509	1557	1038	1557	8882555	false
HSA 13	25316720	33414228	86281130	95382590	+	#00FFFF	8097508	9101460	1038	120659	1038	8098546	false
HSA 13	33534887	46071594	95383210	110562440	+	#00FFFF	12536707	15179230	120659	13168	120659	12657366	false
HSA 13	46084762	46178205	110615258	110763766	+	#00FFFF	93443	148508	13168	1575	13168	106611	false
HSA 13	46179780	46241402	110877748	111009431	+	#00FFFF	61622	131683	1575	6073	1575	63197	false
HSA 13	46247475	46563295	111105861	111668893	+	#00FFFF	315820	563032	6073	101441	6073	321893	false
HSA 13	46664736	46895510	111843416	112192219	+	#00FFFF	230774	348803	101441	44423	101441	332215	false
HSA 13	46939933	47656000	112255413	113671678	+	#00FFFF	716067	1416265	44423	420	44423	760490	false
HSA 13	47656420	48011603	113723068	114328985	+	#00FFFF	355183	605917	420		420	355603	false
HSA 16	48349662	50472752	53427159	55718936	-	#0A803B	2123090	2291777		8157			
HSA 16	50480909	54860603	48639697	53328031	-	#0A803B	4379694	4688334	8157	19580934	8157	4387851	false
HSA 4	54886720	60273895	77420658	82923099	-	#7FFFFF	5387175	5502441		175447			
HSA 4	60449342	61847139	75897187	77293063	-	#7FFFFF	1397797	1395876	175447	45044	175447	1573244	false
HSA 4	61892183	61961213	88086829	88141255	-	#7FFFFF	69030	54426	45044	35684	45044	114074	false
HSA 4	61996897	62054947	88086827	88141924	+	#7FFFFF	58050	55097	35684	123329	35684	93734	false
HSA 4	62178276	62309891	88086827	88145604	-	#7FFFFF	131615	58777	123329	70364	123329	254944	false
HSA 4	62380255	62537716	88049190	88151288	-	#7FFFFF	157461	102098	70364	134968	70364	227825	false
HSA 4	62672684	62765450	88092209	88151283	+	#7FFFFF	92766	59074	134968	-47676	134968	227734	false
HSA 4	62717774	62813248	88037667	88145580	+	#7FFFFF	95474	107913	-47676	2271	-47676	47798	false
HSA 4	62815519	62895701	88037667	88145604	+	#7FFFFF	80182	107937	2271	18411	2271	82453	false
HSA 4	62914112	62969956	88056305	88148486	+	#7FFFFF	55844	92181	18411	50320	18411	74255	false
HSA 4	63020276	63091565	88094901	88145604	+	#7FFFFF	71289	50703	50320	2075	50320	121609	false
HSA 4	63093640	63242727	88037667	88141016	+	#7FFFFF	149087	103349	2075	28917	2075	151162	false
HSA 4	63271644	63370448	88091961	88169371	+	#7FFFFF	98804	77410	28917	64	28917	127721	false
HSA 4	63370512	63489123	88023735	88141016	+	#7FFFFF	118611	117281	64	70554	64	118675	false
HSA 4	63559677	73921257	88033743	98811188	+	#7FFFFF	10361580	10777445	70554	38474	70554	10432134	false
HSA 4	73959731	74301681	98833506	99144638	-	#7FFFFF	341950	311132	38474		38474	380424	false
HSA 16	74441537	74525210	55719208	55770255	-	#0A803B	83673	51047	19580934	-48031	19580934	19664607	false
HSA 16	74477179	74647490	55719208	55797503	-	#0A803B	170311	78295	-48031	-109049	-48031	122280	false

	HSA 16	74538441	74704593	55719208	55797503	-	#0A803B	166152	78295	-109049	-43897	-109049	57103	false
	HSA 16	74660696	74859171	55725693	55797503	-	#0A803B	198475	71810	-43897	-136531	-43897	154578	false
	HSA 16	74722640	74933106	55725693	55797504	-	#0A803B	210466	71811	-136531	-26096	-136531	73935	false
	HSA 16	74907010	82978357	55799504	64578961	+	#0A803B	8071347	8779457	-26096	114	-26096	8045251	false
	HSA 16	82978471	83588957	61930636	62638577	-	#0A803B	610486	707941	114	0	114	610600	false
	HSA 16	83588957	86308802	61930637	65014593	-	#0A803B	2719845	3083956	0	0	0	2719845	false
	HSA 16	86308802	87427642	63999438	65014594	-	#0A803B	1118840	1015156	0	1011	0	1118840	false
	HSA 16	87428653	94299623	62917701	69943578	+	#0A803B	6870970	7025877	1011	52	1011	6871981	false
	HSA 16	94299675	97604268	70896912	74328227	-	#0A803B	3304593	3431315	52	31717	52	3304645	false
	HSA 16	97635985	98119931	70250981	70819448	-	#0A803B	483946	568467	31717	11808	31717	515663	false
	HSA 16	98131739	98249301	70107143	70189637	-	#0A803B	117562	82494	11808	-49067	11808	129370	false
	HSA 16	98200234	98899116	74434592	75226343	+	#0A803B	698882	791751	-49067	36056	-49067	649815	false
	HSA 16	98935172	99583291	75201926	75884693	+	#0A803B	648119	682767	36056	64013	36056	684175	false
	HSA 16	99647304	100349082	75885159	76575402	+	#0A803B	701778	690243	64013	2616	64013	765791	false
	HSA 16	100351698	100933917	76546887	77194090	-	#0A803B	582219	647203	2616	1	2616	584835	false
	HSA 16	100933918	101100608	76977252	77194090	-	#0A803B	166690	216838	1	572	1	166691	false
	HSA 16	101101180	101373644	76547453	76868234	-	#0A803B	272464	320781	572	0	572	273036	false
	HSA 16	101373644	101952928	76546887	77192066	-	#0A803B	579284	645179	0	1	0	579284	false
	HSA 16	101952929	102539800	76550456	77194090	-	#0A803B	586871	643634	1	5585	1	586872	false
	HSA 16	102545385	102874414	76550575	76948069	+	#0A803B	329029	397494	5585	69725	5585	334614	false
	HSA 16	102944139	107078604	76972611	81964749	+	#0A803B	4134465	4992138	69725	114813	69725	4204190	false
	HSA 16	107193417	109249508	81880479	84330325	-	#0A803B	2056091	2449846	114813	4615	114813	2170904	false
	HSA 16	109254123	110987516	84354352	87770398	-	#0A803B	1733393	3416046	4615	34910	4615	1738008	false
	HSA 16	111022426	111264942	87770631	88357389	+	#0A803B	242516	586758	34910	17397	34910	277426	false
	HSA 16	111282339	111604422	88408712	88977072	+	#0A803B	322083	568360	17397	403	17397	339480	false
	HSA 16	111604825	111678752	89045912	89203087	+	#0A803B	73927	157175	403	14021	403	74330	false
	HSA 16	111692773	112335960	89259445	90051543	+	#0A803B	643187	792098	14021		14021	657208	false
**CPOR 30**	HSA 4	64	4307867	20748954	25580287	+	#7FFFFF	4307803	4831333		16038			
	HSA 4	4323905	12732083	25636167	35391791	+	#7FFFFF	8408178	9755624	16038	69379	16038	8424216	false
	HSA 4	12801462	16748472	35466605	40007948	+	#7FFFFF	3947010	4541343	69379	60262	69379	4016389	false
	HSA 4	16808734	19960963	40046439	43434335	+	#7FFFFF	3152229	3387896	60262	51907	60262	3212491	false
	HSA 4	20012870	20837078	43522974	44495991	+	#7FFFFF	824208	973017	51907	68740	51907	876115	false

	HSA 4	20905818	24914214	44560525	49083563	+	#7FFFFF	4008396	4523038	68740	9998	68740	4077136	false
	HSA 4	24924212	30981486	51814471	58297847	+	#7FFFFF	6057274	6483376	9998	35	9998	6067272	false
	HSA 4	30981521	35191485	58390607	63032849	+	#7FFFFF	4209964	4642242	35	1258	35	4209999	false
	HSA 4	35192743	36151272	63083567	64248679	+	#7FFFFF	958529	1165112	1258	76636	1258	959787	false
	HSA 4	36227908	38224770	64248679	66443696	+	#7FFFFF	1996862	2195017	76636	0	76636	2073498	false
	HSA 4	38224770	38283322	66443713	66517863	-	#7FFFFF	58552	74150	0	1593	0	58552	false
	HSA 4	38284915	39202511	66518975	67402987	+	#7FFFFF	917596	884012	1593	133	1593	919189	false
	HSA 4	39202644	39948332	67466944	68273573	+	#7FFFFF	745688	806629	133	7726	133	745821	false
	HSA 4	39956058	40084394	68310385	68503309	-	#7FFFFF	128336	192924	7726	241630	7726	136062	false
	HSA 4	40326024	40414788	69410444	69485023	-	#7FFFFF	88764	74579	241630	85407	241630	330394	false
	HSA 4	40500195	40673222	68831393	68907336	-	#7FFFFF	173027	75943	85407	-89194	85407	258434	false
	HSA 4	40584028	40705125	68507256	68596659	-	#7FFFFF	121097	89403	-89194	-121097	-89194	31903	false
	HSA 4	40584028	40815793	68517405	68719318	-	#7FFFFF	231765	201913	-121097	-223885	-121097	110668	false
	HSA 4	40591908	40758152	69027402	69141406	+	#7FFFFF	166244	114004	-223885	-75322	-223885	-57641	true
	HSA 4	40682830	40796225	69027402	69135515	+	#7FFFFF	113395	108113	-75322	-57690	-75322	38073	false
	HSA 4	40738535	40815784	68568254	68727649	-	#7FFFFF	77249	159395	-57690	-40282	-57690	19559	false
	HSA 4	40775502	40854241	69409066	69470888	-	#7FFFFF	78739	61822	-40282	-78413	-40282	38457	false
	HSA 4	40775828	40855304	69409066	69470888	+	#7FFFFF	79476	61822	-78413	159773	-78413	1063	false
	HSA 4	41015077	41097035	68624521	68727649	+	#7FFFFF	81958	103128	159773	-19590	159773	241731	false
	HSA 4	41077445	41142867	69346437	69443453	-	#7FFFFF	65422	97016	-19590	-57491	-19590	45832	false
	HSA 4	41085376	41142862	69457882	69518561	+	#7FFFFF	57486	60679	-57491	92049	-57491	-5	true
	HSA 4	41234911	41303720	69587726	69656240	+	#7FFFFF	68809	68514	92049	39363	92049	160858	false
	HSA 4	41343083	41460987	69587688	69657608	+	#7FFFFF	117904	69920	39363	67723	39363	157267	false
	HSA 4	41528710	41859208	69699153	70073572	+	#7FFFFF	330498	374419	67723	-21532	67723	398221	false
	HSA 4	41837676	42017499	70082624	70403122	+	#7FFFFF	179823	320498	-21532	0	-21532	158291	false
	HSA 4	42017499	43337117	70462457	71879149	+	#7FFFFF	1319618	1416692	0	567	0	1319618	false
	HSA 4	43337684	45052184	71939112	73650509	+	#7FFFFF	1714500	1711397	567		567	1715067	false
**CPOR 31**	HSA 1	7	9857103	174096347	184974136	-	#00FF7F	9857096	10877789		31190			
	HSA 1	9888293	12374839	171038789	174043149	-	#00FF7F	2486546	3004360	31190	28970	31190	2517736	false
	HSA 1	12403809	13485510	240013784	241450246	+	#00FF7F	1081701	1436462	28970	8634	28970	1110671	false
	HSA 1	13494144	15457550	207665756	209684464	-	#00FF7F	1963406	2018708	8634	-109549	8634	1972040	false
	HSA 1	15348001	16942696	205908526	207665664	-	#00FF7F	1594695	1757138	-109549	-328623	-109549	1485146	false

	HSA 1	16614073	16715721	143938351	144044467	-	#00FF7F	101648	106116	-328623	233578	-328623	-226975	true
	HSA 1	16949299	18672856	203932119	205851601	-	#00FF7F	1723557	1919482	233578	71057	233578	1957135	false
	HSA 1	18743913	20453423	201980238	203892016	-	#00FF7F	1709510	1911778	71057	70411	71057	1780567	false
	HSA 1	20523834	21734580	200622446	201975171	-	#00FF7F	1210746	1352725	70411	58225	70411	1281157	false
	HSA 1	21792805	25149766	197033033	200622446	-	#00FF7F	3356961	3589413	58225	72104	58225	3415186	false
	HSA 1	25221870	25286863	196769744	196832715	-	#00FF7F	64993	62971	72104	-64993	72104	137097	false
	HSA 1	25221870	27073041	194399446	196748034	-	#00FF7F	1851171	2348588	-64993	94	-64993	1786178	false
	HSA 1	27073135	27161317	194222570	194325039	+	#00FF7F	88182	102469	94	164477	94	88276	false
	HSA 1	27325794	35772809	184993235	194165612	-	#00FF7F	8447015	9172377	164477		164477	8611492	false
	HSA 8	35778170	35896369	30575610	30668862	+	#80797D	118199	93252		184693			
	HSA 8	36081062	43919796	30673574	39106687	+	#80797D	7838734	8433113	184693	64334	184693	8023427	false
	HSA 8	43984130	44081918	39530682	39587751	-	#80797D	97788	57069	64334	-67853	64334	162122	false
	HSA 8	44014065	44131826	39445958	39523831	+	#80797D	117761	77873	-67853	89398	-67853	49908	false
	HSA 8	44221224	44325008	39349236	39442620	+	#80797D	103784	93384	89398	463	89398	193182	false
	HSA 8	44325471	44500058	39110666	39284965	+	#80797D	174587	174299	463	195729	463	175050	false
	HSA 8	44695787	44811400	39579621	39730204	+	#80797D	115613	150583	195729	29472	195729	311342	false
	HSA 8	44840872	46407294	39889619	41452572	+	#80797D	1566422	1562953	29472	58332	29472	1595894	false
	HSA 8	46465626	47574554	41472112	42606951	+	#80797D	1108928	1134839	58332	50601	58332	1167260	false
	HSA 8	47625155	47800885	42616859	42802021	+	#80797D	175730	185162	50601		50601	226331	false
**CPOR 4**	HSA 15	274027	405734	101828368	101950223	+	#EC83BA	131707	121855		316016			
	HSA 15	721750	1488745	78853185	79907354	+	#EC83BA	766995	1054169	316016	-15496	316016	1083011	false
	HSA 15	1473249	1707309	79952019	80324135	+	#EC83BA	234060	372116	-15496	5892	-15496	218564	false
	HSA 15	1713201	1972353	22785177	23042641	-	#EC83BA	259152	257464	5892	131	5892	265044	false
	HSA 15	1972484	2088601	20430774	20507549	-	#EC83BA	116117	76775	131	-116117	131	116248	false
	HSA 15	1972484	2091024	22493501	22575114	+	#EC83BA	118540	81613	-116117	-118540	-116117	2423	false
	HSA 15	1972484	4457033	25264668	28323528	-	#EC83BA	2484549	3058860	-118540	-7847	-118540	2366009	false
	HSA 15	4449186	4724361	24847807	25167128	-	#EC83BA	275175	319321	-7847	562696	-7847	267328	false
	HSA 15	5287057	5350699	24388981	24442841	-	#EC83BA	63642	53860	562696	2842	562696	626338	false
	HSA 15	5353541	5578962	23552701	23875360	-	#EC83BA	225421	322659	2842	186269	2842	228263	false
	HSA 15	5765231	5843574	30277229	30376921	+	#EC83BA	78343	99692	186269	-78343	186269	264612	false
	HSA 15	5765231	6877345	30899423	32261428	-	#EC83BA	1112114	1362005	-78343	64343	-78343	1033771	false
	HSA 15	6941688	8080960	28861005	30076423	+	#EC83BA	1139272	1215418	64343	0	64343	1203615	false

HSA 15	8080960	9649804	99921358	101732197	-	#EC83BA	1568844	1810839	0	4527	0	1568844	false
HSA 15	9654331	10259904	99024233	99754865	-	#EC83BA	605573	730632	4527	11	4527	610100	false
HSA 15	10259915	10664788	98537145	99023290	+	#EC83BA	404873	486145	11	0	11	404884	false
HSA 15	10664788	15692502	92780704	98536051	-	#EC83BA	5027714	5755347	0	15955	0	5027714	false
HSA 15	15708457	17415655	91048557	92721951	-	#EC83BA	1707198	1673394	15955	-8656	15955	1723153	false
HSA 15	17406999	21752078	85286423	90273575	+	#EC83BA	4345079	4987152	-8656	854	-8656	4336423	false
HSA 15	21752932	22300897	90315186	91025213	-	#EC83BA	547965	710027	854	33	854	548819	false
HSA 15	22300930	22758920	84598234	85156765	+	#EC83BA	457990	558531	33	2371	33	458023	false
HSA 15	22761291	24144813	82536567	84150119	+	#EC83BA	1383522	1613552	2371	-101311	2371	1385893	false
HSA 15	24043502	25641192	80326875	82277023	-	#EC83BA	1597690	1950148	-101311	39506	-101311	1496379	false
HSA 15	25680698	26775911	43658491	44748401	-	#EC83BA	1095213	1089910	39506	-112202	39506	1134719	false
HSA 15	26663709	35193666	34640418	43657409	-	#EC83BA	8529957	9016991	-112202	53493	-112202	8417755	false
HSA 15	35247159	36961217	32609272	34374869	+	#EC83BA	1714058	1765597	53493	483450	53493	1767551	false
HSA 15	37444667	37596007	101842044	101950224	+	#EC83BA	151340	108180	483450	33697869	483450	634790	false
HSA 1	38004956	39329654	155683854	157192434	-	#00FF7F	1324698	1508580		-79707			
HSA 1	39249947	40089695	154662214	155681866	-	#00FF7F	839748	1019652	-79707	3877	-79707	760041	false
HSA 1	40093572	40231802	154560513	154661804	+	#00FF7F	138230	101291	3877	3774	3877	142107	false
HSA 1	40235576	41202299	153503215	154613939	-	#00FF7F	966723	1110724	3774		3774	970497	false
HSA 7	41492551	41585747	87500139	87617695	+	#7F00FF	93196	117556		49075			
HSA 7	41634822	41699149	134525671	134626158	+	#7F00FF	64327	100487	49075	4012	49075	113402	false
HSA 7	41703161	45039918	134509504	138044555	+	#7F00FF	3336757	3535051	4012	119219	4012	3340769	false
HSA 7	45159137	48534890	138065986	141842118	+	#7F00FF	3375753	3776132	119219	16180	119219	3494972	false
HSA 7	48551070	49836765	141924511	142864560	+	#7F00FF	1285695	940049	16180	-814972	16180	1301875	false
HSA 7	49021793	49465749	142419624	142581470	+	#7F00FF	443956	161846	-814972	-388157	-814972	-371016	true
HSA 7	49077592	49613204	142289633	142443157	+	#7F00FF	535612	153524	-388157	-422315	-388157	147455	false
HSA 7	49190889	49277923	142487008	142541340	+	#7F00FF	87034	54332	-422315	-87034	-422315	-335281	true
HSA 7	49190889	49613161	142348095	142443110	+	#7F00FF	422272	95015	-87034	-259283	-87034	335238	false
HSA 7	49353878	49580127	142479680	142547062	+	#7F00FF	226249	67382	-259283	256638	-259283	-33034	true
HSA 7	49836765	49986625	142825059	143015437	-	#7F00FF	149860	190378	256638	-10174	256638	406498	false
HSA 7	49976451	50069569	142834314	142966934	+	#7F00FF	93118	132620	-10174	9421	-10174	82944	false
HSA 7	50078990	50702247	142893103	143494158	+	#7F00FF	623257	601055	9421	17540	9421	632678	false
HSA 7	50719787	50845413	143470680	143569308	+	#7F00FF	125626	98628	17540	-47904	17540	143166	false

	HSA 7	50797509	50898170	143717551	143807701	-	#7F00FF	100661	90150	-47904	-3358	-47904	52757	false
	HSA 7	50894812	51215095	143818407	144158438	+	#7F00FF	320283	340031	-3358	213123	-3358	316925	false
	HSA 7	51428218	51564555	144186942	144256871	-	#7F00FF	136337	69929	213123	-120883	213123	349460	false
	HSA 7	51443672	55945803	144296188	149888344	+	#7F00FF	4502131	5592156	-120883	-251096	-120883	4381248	false
	HSA 7	55694707	55761512	149589146	149699315	+	#7F00FF	66805	110169	-251096	-55885	-251096	-184291	true
	HSA 7	55705627	55764064	149589146	149661624	+	#7F00FF	58437	72478	-55885	181739	-55885	2552	false
	HSA 7	55945803	57673966	150320762	152762347	+	#7F00FF	1728163	2441585	181739	47963	181739	1909902	false
	HSA 7	57721929	58386851	152850721	153705056	+	#7F00FF	664922	854335	47963	89063	47963	712885	false
	HSA 7	58475914	58638265	153820256	153957172	+	#7F00FF	162351	136916	89063	11068	89063	251414	false
	HSA 7	58649333	58706681	150004310	150068223	-	#7F00FF	57348	63913	11068	46341	11068	68416	false
	HSA 7	58753022	59735319	154167268	155782655	+	#7F00FF	982297	1615387	46341	108123	46341	1028638	false
	HSA 7	59843442	61777377	155795105	159175861	+	#7F00FF	1933935	3380756	108123	22765	108123	2042058	false
	HSA 7	61800142	61882269	159243579	159331441	+	#7F00FF	82127	87862	22765	33620591	22765	104892	false
	HSA 17	62285036	63489998	58549699	59967259	+	#FF0000	1204962	1417560		-41513			
	HSA 17	63448485	63541286	20711518	20830787	+	#FF0000	92801	119269	-41513	-22518	-41513	51288	false
	HSA 17	63518768	65225253	60138663	62228764	-	#FF0000	1706485	2090101	-22518	281	-22518	1683967	false
	HSA 17	65225534	66455148	36456995	37843052	+	#FF0000	1229614	1386057	281	225	281	1229895	false
	HSA 17	66455373	66544173	60033262	60109361	+	#FF0000	88800	76099	225	-30911	225	89025	false
	HSA 17	66513262	66584019	36186873	36245689	-	#FF0000	70757	58816	-30911	-70757	-30911	39846	false
	HSA 17	66513262	66587868	36077702	36158055	-	#FF0000	74606	80353	-70757	364559	-70757	3849	false
	HSA 17	66952427	67058856	35968832	36076332	-	#FF0000	106429	107500	364559	13073	364559	470988	false
	HSA 17	67071929	67356717	35530554	35901807	-	#FF0000	284788	371253	13073	-16527	13073	297861	false
	HSA 17	67340190	67432426	35350591	35444919	-	#FF0000	92236	94328	-16527	48232	-16527	75709	false
	HSA 17	67480658	70262177	32133854	35273799	-	#FF0000	2781519	3139945	48232	202	48232	2829751	false
	HSA 17	70262379	70498945	30731228	31005961	-	#FF0000	236566	274733	202	-49605	202	236768	false
	HSA 17	70449340	70960076	31355849	32001249	-	#FF0000	510736	645400	-49605	1097655	-49605	461131	false
	HSA 15	71293876	71347716	21604056	21685093	+	#EC83BA	53840	81037	33697869	382362	33697869	33751709	false
	HSA 15	71730078	71828211	101868072	101950224	-	#EC83BA	98133	82152	382362	-25617	382362	480495	false
	HSA 15	71802594	71888908	101806666	101876888	-	#EC83BA	86314	70222	-25617		-25617	60697	false
	HSA 17	72057731	73319050	5514149	6845307	+	#FF0000	1261319	1331158	1097655	-3701	1097655	2358974	false
	HSA 17	73315349	74400674	3397883	4628870	-	#FF0000	1085325	1230987	-3701	301051	-3701	1081624	false
	HSA 17	74701725	74904502	3279379	3438711	-	#FF0000	202777	159332	301051	14238	301051	503828	false

	HSA 17	74918740	75024101	3146875	3242603	+	#FF0000	105361	95728	14238	221400	14238	119599	false
	HSA 17	75245501	76747477	1337967	3039912	-	#FF0000	1501976	1701945	221400	19124	221400	1723376	false
	HSA 17	76766601	77760505	142967	1334471	+	#FF0000	993904	1191504	19124	-49894	19124	1013028	false
	HSA 17	77710611	77894631	30343335	30529492	-	#FF0000	184020	186157	-49894	118960	-49894	134126	false
	HSA 17	78013591	79844416	28312310	30343066	-	#FF0000	1830825	2030756	118960	0	118960	1949785	false
	HSA 17	79844416	80269824	27741366	28197156	-	#FF0000	425408	455790	0	359944	0	425408	false
	HSA 17	80629768	81047963	27197291	27650843	-	#FF0000	418195	453552	359944	-31085	359944	778139	false
	HSA 17	81016878	81316486	31050969	31385750	+	#FF0000	299608	334781	-31085	329646	-31085	268523	false
	HSA 17	81646132	83221027	56862062	58608223	-	#FF0000	1574895	1746161	329646	301539	329646	1904541	false
	HSA 17	83522566	88960868	50020433	56869725	-	#FF0000	5438302	6849292	301539	142083	301539	5739841	false
	HSA 17	89102951	89709651	49097493	49999457	-	#FF0000	606700	901964	142083	-34850	142083	748783	false
	HSA 17	89674801	90608116	47762191	49090113	-	#FF0000	933315	1327922	-34850	60385	-34850	898465	false
	HSA 17	90668501	90788378	47596280	47757543	-	#FF0000	119877	161263	60385	36199	60385	180262	false
	HSA 17	90824577	90880793	47480742	47542756	-	#FF0000	56216	62014	36199	-56216	36199	92415	false
	HSA 17	90824577	91285777	38245367	39001042	+	#FF0000	461200	755675	-56216	130	-56216	404984	false
	HSA 17	91285907	92449701	39061574	40720333	+	#FF0000	1163794	1658759	130	-38497	130	1163924	false
	HSA 17	92411204	92971039	40721380	41225806	+	#FF0000	559835	504426	-38497	-91402	-38497	521338	false
	HSA 17	92879637	93696126	41220389	42615093	+	#FF0000	816489	1394704	-91402	-23160	-91402	725087	false
	HSA 17	93672966	93857039	42612618	42924900	+	#FF0000	184073	312282	-23160	226308	-23160	160913	false
	HSA 17	94083347	94205969	42950541	43228883	+	#FF0000	122622	278342	226308	16397	226308	348930	false
	HSA 17	94222366	94758804	43477121	44564169	+	#FF0000	536438	1087048	16397	24763	16397	552835	false
	HSA 17	94783567	95382766	44646415	45499992	+	#FF0000	599199	853577	24763		24763	623962	false
	HSA 11	95610087	96341110	17314547	18118624	-	#0000FF	731023	804077		6578			
	HSA 11	96347688	96405543	17330062	17386638	+	#0000FF	57855	56576	6578	14802	6578	64433	false
	HSA 11	96420345	103686347	9277181	17368628	-	#0000FF	7266002	8091447	14802	101532	14802	7280804	false
	HSA 11	103787879	104025433	1176768	1577388	-	#0000FF	237554	400620	101532	-34864	101532	339086	false
	HSA 11	103990569	104764596	192132	1176931	-	#0000FF	774027	984799	-34864		-34864	739163	false
**CPOR 5**	HSA 11	49297	547692	18236654	18806863	+	#0000FF	498395	570209		2618			
	HSA 11	550310	747113	18948269	19029877	+	#0000FF	196803	81608	2618	-95514	2618	199421	false
	HSA 11	651599	747565	18801169	18957739	+	#0000FF	95966	156570	-95514	186872	-95514	452	false
	HSA 11	934437	2919845	18958157	21457782	+	#0000FF	1985408	2499625	186872	2	186872	2172280	false
	HSA 11	2919847	3526186	20615789	21326952	+	#0000FF	606339	711163	2	606	2	606341	false

	HSA 11	3526792	3984968	21603130	22219040	-	#0000FF	458176	615910	606	19823	606	458782	false
	HSA 11	4004791	4256803	20615789	20953814	-	#0000FF	252012	338025	19823	2	19823	271835	false
	HSA 11	4256805	4985324	20615789	21457782	-	#0000FF	728519	841993	2	2	2	728521	false
	HSA 11	4985326	5045678	21335861	21457782	-	#0000FF	60352	121921	2	91804	2	60354	false
	HSA 11	5137482	5542998	20615789	21212976	-	#0000FF	405516	597187	91804	27240	91804	497320	false
	HSA 11	5570238	5809223	20831365	21073499	+	#0000FF	238985	242134	27240	1	27240	266225	false
	HSA 11	5809224	6346486	21002777	21631665	+	#0000FF	537262	628888	1	4516	1	537263	false
	HSA 11	6351002	6445863	21228239	21334108	+	#0000FF	94861	105869	4516	17954	4516	99377	false
	HSA 11	6463817	8545387	21458368	24168381	+	#0000FF	2081570	2710013	17954	8116	17954	2099524	false
	HSA 11	8553503	8624930	24185919	24287639	-	#0000FF	71427	101720	8116	-61750	8116	79543	false
	HSA 11	8563180	9582810	24254314	25583867	+	#0000FF	1019630	1329553	-61750	928	-61750	957880	false
	HSA 11	9583738	10639693	24586920	25975120	-	#0000FF	1055955	1388200	928	311	928	1056883	false
	HSA 11	10640004	10757696	25975163	26142254	-	#0000FF	117692	167091	311	812	311	118003	false
	HSA 11	10758508	11196799	25584014	26148761	+	#0000FF	438291	564747	812	11732	812	439103	false
	HSA 11	11208531	17141136	24586920	32796688	+	#0000FF	5932605	8209768	11732	451032	11732	5944337	false
	HSA 11	17592168	17695103	48080701	48246590	-	#0000FF	102935	165889	451032	83133	451032	553967	false
	HSA 11	17778236	19220483	7727100	9275140	-	#0000FF	1442247	1548040	83133	200940	83133	1525380	false
	HSA 11	19421423	20250465	6852781	7728687	-	#0000FF	829042	875906	200940	13943	200940	1029982	false
	HSA 11	20264408	20474365	6832814	7001632	+	#0000FF	209957	168818	13943	228	13943	223900	false
	HSA 11	20474593	20648649	6790810	6853074	-	#0000FF	174056	62264	228	374535	228	174284	false
	HSA 11	21023184	21163483	6708914	6788537	+	#0000FF	140299	79623	374535	-88054	374535	514834	false
	HSA 11	21075429	21221138	6708628	6784400	-	#0000FF	145709	75772	-88054	78449	-88054	57655	false
	HSA 11	21299587	22000007	5996183	6747596	-	#0000FF	700420	751413	78449	-40420	78449	778869	false
	HSA 11	21959587	22153991	5859824	5992892	-	#0000FF	194404	133068	-40420	25810	-40420	153984	false
	HSA 11	22179801	22230319	5773794	5832561	+	#0000FF	50518	58767	25810	-23542	25810	76328	false
	HSA 11	22206777	22419449	5732023	5804082	-	#0000FF	212672	72059	-23542	-118526	-23542	189130	false
	HSA 11	22300923	22472554	5697081	5759282	-	#0000FF	171631	62201	-118526	278610	-118526	53105	false
	HSA 11	22751164	22808531	5847935	5902175	-	#0000FF	57367	54240	278610	205118	278610	335977	false
	HSA 11	23013649	23071963	5773874	5832561	+	#0000FF	58314	58687	205118	-19347	205118	263432	false
	HSA 11	23052616	23202148	5722680	5804082	-	#0000FF	149532	81402	-19347	64426	-19347	130185	false
	HSA 11	23266574	24108466	5118368	5713072	-	#0000FF	841892	594704	64426	-753451	64426	906318	false
	HSA 11	23355015	23536674	5632767	5710642	+	#0000FF	181659	77875	-753451	1010232	-753451	-571792	true

	HSA 11	24546906	24606987	4862977	4930001	+	#0000FF	60081	67024	1010232	104688	1010232	1070313	false
	HSA 11	24711675	24795705	4691756	4851463	-	#0000FF	84030	159707	104688	278237	104688	188718	false
	HSA 11	25073942	25205607	5058606	5156322	+	#0000FF	131665	97716	278237	106275	278237	409902	false
	HSA 11	25311882	25362556	5034658	5091530	+	#0000FF	50674	56872	106275	21241	106275	156949	false
	HSA 11	25383797	25472933	5118364	5174978	-	#0000FF	89136	56614	21241	193112	21241	110377	false
	HSA 11	25666045	25753847	5034658	5110272	-	#0000FF	87802	75614	193112	-33213	193112	280914	false
	HSA 11	25720634	25784067	5059875	5110272	+	#0000FF	63433	50397	-33213	178112	-33213	30220	false
	HSA 11	25962179	26017289	4972935	5064421	+	#0000FF	55110	91486	178112	2840	178112	233222	false
	HSA 11	26020129	26104701	4862967	4948171	-	#0000FF	84572	85204	2840	317156	2840	87412	false
	HSA 11	26421857	26576492	5118368	5174978	+	#0000FF	154635	56610	317156	464104	317156	471791	false
	HSA 11	27040596	27288832	4741766	4948171	+	#0000FF	248236	206405	464104	2211	464104	712340	false
	HSA 11	27291043	27362432	4981832	5064421	-	#0000FF	71389	82589	2211	-15553	2211	73600	false
	HSA 11	27346879	27416033	4967280	5064023	+	#0000FF	69154	96743	-15553	14046	-15553	53601	false
	HSA 11	27430079	27554418	4876583	4981378	-	#0000FF	124339	104795	14046	-14771	14046	138385	false
	HSA 11	27539647	27640416	4802631	4890739	+	#0000FF	100769	88108	-14771	1938	-14771	85998	false
	HSA 11	27642354	28198143	4372853	4818711	-	#0000FF	555789	445858	1938	64759	1938	557727	false
	HSA 11	28262902	28792730	3605106	4194491	-	#0000FF	529828	589385	64759	2	64759	594587	false
	HSA 11	28792732	32376665	71915623	75717280	+	#0000FF	3583933	3801657	2	209244	2	3583935	false
	HSA 11	32585909	33643567	75702531	76828869	+	#0000FF	1057658	1126338	209244	1399240	209244	1266902	false
	HSA 11	35042807	40394346	76829384	82715514	+	#0000FF	5351539	5886130	1399240	57261	1399240	6750779	false
	HSA 11	40451607	42929195	82718127	85470439	+	#0000FF	2477588	2752312	57261	35376	57261	2534849	false
	HSA 11	42964571	45893360	85523146	88856240	+	#0000FF	2928789	3333094	35376	65997	35376	2964165	false
	HSA 11	45959357	46135679	49492043	49660480	-	#0000FF	176322	168437	65997	-176322	65997	242319	false
	HSA 11	45959357	46288109	88958752	89324456	+	#0000FF	328752	365704	-176322	66566	-176322	152430	false
	HSA 11	46354675	46760380	89405820	89759861	+	#0000FF	405705	354041	66566	-37547	66566	472271	false
	HSA 2	46679023	46775798	95500001	95558810	+	#036A9C	96775	58809		-87357			
	HSA 2	46688441	46789761	95519587	95591869	-	#036A9C	101320	72282	-87357		-87357	13963	false
	HSA 11	46722833	46947141	90080105	90289732	+	#0000FF	224308	209627	-37547	11751	-37547	186761	false
	HSA 11	46958892	51208376	90347640	95059888	+	#0000FF	4249484	4712248	11751	354589	11751	4261235	false
	HSA 11	51562965	54458862	95013018	98408622	+	#0000FF	2895897	3395604	354589	1763	354589	3250486	false
	HSA 11	54460625	54619576	98408630	98683473	-	#0000FF	158951	274843	1763	578	1763	160714	false
	HSA 11	54620154	57252883	98683473	101859032	+	#0000FF	2632729	3175559	578	52713	578	2633307	false

	HSA 11	57305596	58214240	101859558	102760731	+	#0000FF	908644	901173	52713	-42024	52713	961357	false
	HSA 11	58172216	60235022	102768089	104980762	+	#0000FF	2062806	2212673	-42024	-88982	-42024	2020782	false
	HSA 11	60146040	60284251	104980766	105055024	+	#0000FF	138211	74258	-88982	-23632	-88982	49229	false
	HSA 11	60260619	60321072	105055559	105138733	-	#0000FF	60453	83174	-23632	-60453	-23632	36821	false
	HSA 11	60260619	60356777	104942782	105070526	-	#0000FF	96158	127744	-60453	11534	-60453	35705	false
	HSA 11	60368311	60561864	105135279	105298388	+	#0000FF	193553	163109	11534	50414	11534	205087	false
	HSA 11	60612278	61542438	105359230	106376898	+	#0000FF	930160	1017668	50414	27343	50414	980574	false
	HSA 11	61569781	62281512	106582263	107490197	-	#0000FF	711731	907934	27343	84462	27343	739074	false
	HSA 11	62365974	62610940	106376898	106581964	+	#0000FF	244966	205066	84462	308580	84462	329428	false
	HSA 11	62919520	71667529	125776134	134885434	-	#0000FF	8748009	9109300	308580	-51459	308580	9056589	false
	HSA 11	71616070	72882823	124568788	125767471	-	#0000FF	1266753	1198683	-51459	78534	-51459	1215294	false
	HSA 11	72961357	73327990	124308288	124571461	-	#0000FF	366633	263173	78534	115068	78534	445167	false
	HSA 11	73443058	73577807	124242848	124368050	-	#0000FF	134749	125202	115068	62174	115068	249817	false
	HSA 11	73639981	73703054	124322348	124404544	+	#0000FF	63073	82196	62174	-21644	62174	125247	false
	HSA 11	73681410	73926296	124173163	124368048	-	#0000FF	244886	194885	-21644	210273	-21644	223242	false
	HSA 11	74136569	74240333	124267051	124341803	-	#0000FF	103764	74752	210273	-16627	210273	314037	false
	HSA 11	74223706	74282556	124169931	124267930	-	#0000FF	58850	97999	-16627	245413	-16627	42223	false
	HSA 11	74527969	74786584	124050302	124218639	+	#0000FF	258615	168337	245413	-17672	245413	504028	false
	HSA 11	74768912	74847795	124237835	124315812	+	#0000FF	78883	77977	-17672	162180	-17672	61211	false
	HSA 11	75009975	75254174	124251303	124368050	+	#0000FF	244199	116747	162180	116962	162180	406379	false
	HSA 11	75371136	75616946	124173163	124368050	+	#0000FF	245810	194887	116962	60600	116962	362772	false
	HSA 11	75677546	76091179	123804848	124114498	-	#0000FF	413633	309650	60600	-9168	60600	474233	false
	HSA 11	76082011	84448366	114650025	123803402	-	#0000FF	8366355	9153377	-9168	9090	-9168	8357187	false
	HSA 11	84457456	84511998	114531151	114598421	-	#0000FF	54542	67270	9090	26433	9090	63632	false
	HSA 11	84538431	84628210	114479474	114533000	+	#0000FF	89779	53526	26433	58128	26433	116212	false
	HSA 11	84686338	91323700	107493033	114479473	-	#0000FF	6637362	6986440	58128		58128	6695490	false
**CPOR 6**	HSA 4	315557	641473	75573983	75904702	+	#7FFFFF	325916	330719		19104			
	HSA 4	660577	4849443	83518534	88078355	-	#7FFFFF	4188866	4559821	19104	62921	19104	4207970	false
	HSA 4	4912364	5297415	82923215	83485311	-	#7FFFFF	385051	562096	62921		62921	447972	false
	HSA 16	5302785	7089514	46581690	48615880	-	#0A803B	1786729	2034190					
	HSA 3	7620056	7991266	166795565	167172132	-	#02BCB7	371210	376567		18628			
	HSA 3	8009894	8082879	166608804	166732778	-	#02BCB7	72985	123974	18628	70954	18628	91613	false

	HSA 3	8153833	10768195	163798764	166530596	-	#02BCB7	2614362	2731832	70954	50760	70954	2685316	false
	HSA 3	10818955	14609876	159515486	163704520	-	#02BCB7	3790921	4189034	50760	3682	50760	3841681	false
	HSA 3	14613558	14703087	159417464	159515475	+	#02BCB7	89529	98011	3682	659	3682	93211	false
	HSA 3	14703746	21908943	151804437	159417464	-	#02BCB7	7205197	7613027	659	96012	659	7205856	false
	HSA 3	22004955	22070996	151776763	151842910	-	#02BCB7	66041	66147	96012	75647	96012	162053	false
	HSA 3	22146643	24067457	149809229	151833818	-	#02BCB7	1920814	2024589	75647	591196	75647	1996461	false
	HSA 3	24658653	28088046	146433236	149812192	-	#02BCB7	3429393	3378956	591196	-200576	591196	4020589	false
	HSA 3	27887470	28187536	146329904	146471190	-	#02BCB7	300066	141286	-200576	-47968	-200576	99490	false
	HSA 3	28139568	29193338	145280950	146284980	-	#02BCB7	1053770	1004030	-47968	21106	-47968	1005802	false
	HSA 3	29214444	29306951	145193110	145279010	+	#02BCB7	92507	85900	21106	2	21106	113613	false
	HSA 3	29306953	32096138	142296895	145193064	-	#02BCB7	2789185	2896169	2	23502	2	2789187	false
	HSA 3	32119640	35582357	138650455	142241255	-	#02BCB7	3462717	3590800	23502	69949	23502	3486219	false
	HSA 3	35652306	43562356	130340770	138637925	-	#02BCB7	7910050	8297155	69949	582	69949	7979999	false
	HSA 3	43562938	43689033	130212776	130286544	-	#02BCB7	126095	73768	582	133566	582	126677	false
	HSA 3	43822599	44269063	51879115	52313051	-	#02BCB7	446464	433936	133566	-35124	133566	580030	false
	HSA 3	44233939	47467152	48515353	51878847	-	#02BCB7	3233213	3363494	-35124	53526	-35124	3198089	false
	HSA 3	47520678	47631742	48348173	48509879	-	#02BCB7	111064	161706	53526		53526	164590	false
	HSA 20	48426408	51637051	1763854	5444701	+	#EF8701	3210643	3680847		8382			
	HSA 20	51645433	62821463	5498421	18223139	+	#EF8701	11176030	12724718	8382	-12239	8382	11184412	false
	HSA 20	62809224	66285039	18380772	23553614	+	#EF8701	3475815	5172842	-12239	17442	-12239	3463576	false
	HSA 20	66302481	66431637	23639708	23693554	+	#EF8701	129156	53846	17442	6577	17442	146598	false
	HSA 20	66438214	66758771	24044984	24823824	+	#EF8701	320557	778840	6577	6703	6577	327134	false
	HSA 20	66765474	67165169	24908293	25624405	+	#EF8701	399695	716112	6703	88901	6703	406398	false
	HSA 20	67254070	67676099	1561357	1711268	-	#EF8701	422029	149911	88901	-422025	88901	510930	false
	HSA 20	67254074	67704520	1582864	1711263	-	#EF8701	450446	128399	-422025	-450440	-422025	28421	false
	HSA 20	67254080	67598041	1561357	1660078	-	#EF8701	343961	98721	-450440	-330093	-450440	-106479	true
	HSA 20	67267948	67674530	1629348	1722167	-	#EF8701	406582	92819	-330093	-305304	-330093	76489	false
	HSA 20	67369226	67704520	1652808	1722167	-	#EF8701	335294	69359	-305304	-33059	-305304	29990	false
	HSA 20	67671461	68233462	940594	1521865	-	#EF8701	562001	581271	-33059	119260	-33059	528942	false
	HSA 20	68352722	68421222	883997	952386	-	#EF8701	68500	68389	119260	-7992	119260	187760	false
	HSA 20	68413230	68473462	829155	917794	+	#EF8701	60232	88639	-7992	61236	-7992	52240	false
	HSA 20	68534698	68687596	748400	962613	-	#EF8701	152898	214213	61236	138913	61236	214134	false

	HSA 20	68826509	69398163	85809	740907	-	#EF8701	571654	655098	138913	861	138913	710567	false
	HSA 20	69399024	69556327	31253962	31451060	+	#EF8701	157303	197098	861	57258	861	158164	false
	HSA 20	69613585	71012358	31460101	33183960	+	#EF8701	1398773	1723859	57258	206335	57258	1456031	false
	HSA 20	71218693	72187637	33206234	34199051	+	#EF8701	968944	992817	206335	25393	206335	1175279	false
	HSA 20	72213030	75996855	34169105	38440657	+	#EF8701	3783825	4271552	25393	38374	25393	3809218	false
	HSA 20	76035229	76231140	38420049	38676963	+	#EF8701	195911	256914	38374	85828	38374	234285	false
	HSA 20	76316968	76406265	38526462	38688536	-	#EF8701	89297	162074	85828	17665	85828	175125	false
	HSA 20	76423930	82483192	38620725	45177560	+	#EF8701	6059262	6556835	17665	-95835	17665	6076927	false
	HSA 20	82387357	82860690	45176108	45454606	+	#EF8701	473333	278498	-95835	299481	-95835	377498	false
	HSA 20	83160171	94403416	45455585	59521485	+	#EF8701	11243245	14065900	299481	-9096420	299481	11542726	false
	HSA 20	85306996	85468762	47789330	48077981	-	#EF8701	161766	288651	-9096420	9307828	-9096420	-8934654	true
	HSA 20	94776590	98614970	59545620	64276314	+	#EF8701	3838380	4730694	9307828		9307828	13146208	false
**CPOR 7**	HSA 6	348	3081940	99874430	102948672	+	#EBE47D	3081592	3074242		53856			
	HSA 6	3135796	3209435	102986164	103048815	-	#EBE47D	73639	62651	53856	233	53856	127495	false
	HSA 6	3209668	9025415	103094121	109545058	+	#EBE47D	5815747	6450937	233	90	233	5815980	false
	HSA 6	9025505	9739800	109653635	110424691	+	#EBE47D	714295	771056	90	4427057	90	714385	false
	HSA 6	14166857	14624783	110474457	110950072	+	#EBE47D	457926	475615	4427057	50398	4427057	4884983	false
	HSA 6	14675181	14754569	110954954	111030728	+	#EBE47D	79388	75774	50398	50527	50398	129786	false
	HSA 6	14805096	15073744	111057050	111294474	+	#EBE47D	268648	237424	50527	84703	50527	319175	false
	HSA 6	15158447	15948782	111258299	112112155	+	#EBE47D	790335	853856	84703	922	84703	875038	false
	HSA 6	15949704	16428000	112044092	112555039	+	#EBE47D	478296	510947	922	6840	922	479218	false
	HSA 6	16434840	16549939	112555210	112685325	-	#EBE47D	115099	130115	6840	0	6840	121939	false
	HSA 6	16549939	18555687	112687031	114839053	+	#EBE47D	2005748	2152022	0	108	0	2005748	false
	HSA 6	18555795	18634281	114841208	114955305	-	#EBE47D	78486	114097	108	5044	108	78594	false
	HSA 6	18639325	20269640	114960794	116593592	+	#EBE47D	1630315	1632798	5044	47080	5044	1635359	false
	HSA 6	20316720	22269423	168150961	170581093	+	#EBE47D	1952703	2430132	47080	14878	47080	1999783	false
	HSA 6	22284301	22399025	109690276	109827236	+	#EBE47D	114724	136960	14878	29023	14878	129602	false
	HSA 6	22428048	25493079	52945234	56352609	+	#EBE47D	3065031	3407375	29023		29023	3094054	false
	HSA 10	25747432	26138671	15027158	15411017	-	#82C90F	391239	383859		0			
	HSA 10	26138671	27593137	13314784	14973481	-	#82C90F	1454466	1658697	0	196775	0	1454466	false
	HSA 10	27789912	28424257	28644655	29400317	+	#82C90F	634345	755662	196775	-14194	196775	831120	false
	HSA 10	28410063	28537102	30409946	30597723	-	#82C90F	127039	187777	-14194	90412	-14194	112845	false

	HSA 10	28627514	29304875	29443875	30248826	-	#82C90F	677361	804951	90412	-41842	90412	767773	false
	HSA 10	29263033	30792989	30687086	32420888	+	#82C90F	1529956	1733802	-41842	82625	-41842	1488114	false
	HSA 10	30875614	31713561	12058685	13075460	+	#82C90F	837947	1016775	82625	55630	82625	920572	false
	HSA 10	31769191	31922764	13098082	13298547	+	#82C90F	153573	200465	55630	58806	55630	209203	false
	HSA 10	31981570	33122346	27448494	28639252	+	#82C90F	1140776	1190758	58806	22549	58806	1199582	false
	HSA 10	33144895	35333527	32814310	35222403	-	#82C90F	2188632	2408093	22549	105532	22549	2211181	false
	HSA 10	35439059	35543045	32445392	32586551	-	#82C90F	103986	141159	105532	21344	105532	209518	false
	HSA 10	35564389	37023384	35227153	36885576	-	#82C90F	1458995	1658423	21344		21344	1480339	false
	HSA 1	37081223	38159179	169918197	170930989	+	#00FF7F	1077956	1012792		53254			
	HSA 1	38212433	38384397	170072705	170352186	-	#00FF7F	171964	279481	53254	12996	53254	225218	false
	HSA 1	38397393	41358673	166808583	170012691	-	#00FF7F	2961280	3204108	12996	0	12996	2974276	false
	HSA 1	41358673	46128334	161591116	166705127	-	#00FF7F	4769661	5114011	0	-83182	0	4769661	false
	HSA 1	46045152	46136359	161503374	161611327	-	#00FF7F	91207	107953	-83182	30568	-83182	8025	false
	HSA 1	46166927	46616170	160944911	161429252	-	#00FF7F	449243	484341	30568	113417	30568	479811	false
	HSA 1	46729587	49033081	158880948	160888942	-	#00FF7F	2303494	2007994	113417	-309758	113417	2416911	false
	HSA 1	48723323	49648672	158352653	159001859	-	#00FF7F	925349	649206	-309758	-716348	-309758	615591	false
	HSA 1	48932324	49016067	159001859	159062877	-	#00FF7F	83743	61018	-716348	1786133	-716348	-632605	true
	HSA 1	50802200	50980578	158202190	158293677	-	#00FF7F	178378	91487	1786133	73969	1786133	1964511	false
	HSA 1	51054547	51704827	157489667	158186144	-	#00FF7F	650280	696477	73969		73969	724249	false
	HSA 19	51755272	52299725	28098099	28737000	-	#64E09A	544453	638901		123941			
	HSA 19	52423666	56052092	28818818	32933682	+	#64E09A	3628426	4114864	123941	2383	123941	3752367	false
	HSA 19	56054475	56107599	32816073	32871521	+	#64E09A	53124	55448	2383	14511	2383	55507	false
	HSA 19	56122110	56324951	32826823	33125842	+	#64E09A	202841	299019	14511	8324	14511	217352	false
	HSA 19	56333275	57006274	34913786	35645873	-	#64E09A	672999	732087	8324	49767	8324	681323	false
	HSA 19	57056041	57114946	34733311	34786315	-	#64E09A	58905	53004	49767	236912	49767	108672	false
	HSA 19	57351858	57528656	57940871	58029460	-	#64E09A	176798	88589	236912	189694	236912	413710	false
	HSA 19	57718350	57780350	57595053	57679095	-	#64E09A	62000	84042	189694	131857	189694	251694	false
	HSA 19	57912207	57995518	57398095	57564148	-	#64E09A	83311	166053	131857	-17213	131857	215168	false
	HSA 19	57978305	58137860	57230910	57379830	-	#64E09A	159555	148920	-17213	81804	-17213	142342	false
	HSA 19	58219664	58275580	56765768	56881101	-	#64E09A	55916	115333	81804	117766	81804	137720	false
	HSA 19	58393346	58455313	56507151	56581043	-	#64E09A	61967	73892	117766	4	117766	179733	false
	HSA 19	58455317	58516879	56379025	56483409	+	#64E09A	61562	104384	4	16984	4	61566	false

	HSA 19	58533863	58653161	56086963	56315735	-	#64E09A	119298	228772	16984	157582	16984	136282	false
	HSA 19	58810743	59301463	55042550	55696882	-	#64E09A	490720	654332	157582	501368	157582	648302	false
	HSA 19	59802831	60074572	54573323	54699303	-	#64E09A	271741	125980	501368	-268656	501368	773109	false
	HSA 19	59805916	59995811	54256669	54340539	+	#64E09A	189895	83870	-268656	-168639	-268656	-78761	true
	HSA 19	59827172	60165258	54573490	54713506	-	#64E09A	338086	140016	-168639	-121733	-168639	169447	false
	HSA 19	60043525	60194274	54599518	54713506	-	#64E09A	150749	113988	-121733	51167	-121733	29016	false
	HSA 19	60245441	60311306	54306989	54357410	+	#64E09A	65865	50421	51167	-65865	51167	117032	false
	HSA 19	60245441	60353439	54585913	54713506	-	#64E09A	107998	127593	-65865	110310	-65865	42133	false
	HSA 19	60463749	60625578	54252739	54340539	+	#64E09A	161829	87800	110310	151688	110310	272139	false
	HSA 19	60777266	60858964	54354071	54474628	-	#64E09A	81698	120557	151688	164778	151688	233386	false
	HSA 19	61023742	61130132	54250136	54357561	-	#64E09A	106390	107425	164778	-105042	164778	271168	false
	HSA 19	61025090	61130280	54601045	54668927	+	#64E09A	105190	67882	-105042	58613	-105042	148	false
	HSA 19	61188893	61273283	54215625	54301179	-	#64E09A	84390	85554	58613	-69911	58613	143003	false
	HSA 19	61203372	61327571	54242206	54340539	-	#64E09A	124199	98333	-69911	-124199	-69911	54288	false
	HSA 19	61203372	61451773	54588886	54713506	+	#64E09A	248401	124620	-124199	-55536	-124199	124202	false
	HSA 19	61396237	61469107	54242206	54309619	-	#64E09A	72870	67413	-55536	-72540	-55536	17334	false
	HSA 19	61396567	61568158	54231399	54335997	-	#64E09A	171591	104598	-72540	211006	-72540	99051	false
	HSA 19	61779164	62043783	53795816	54256588	-	#64E09A	264619	460772	211006	1195973	211006	475625	false
	HSA 19	63239756	63317872	53199777	53287112	-	#64E09A	78116	87335	1195973	172998	1195973	1274089	false
	HSA 19	63490870	63573173	53199777	53287112	-	#64E09A	82303	87335	172998	79598	172998	255301	false
	HSA 19	63652771	63722538	53199777	53287112	-	#64E09A	69767	87335	79598	-36175	79598	149365	false
	HSA 19	63686363	63825458	53203746	53305212	+	#64E09A	139095	101466	-36175	469802	-36175	102920	false
	HSA 19	64295260	64400712	52174794	52264395	+	#64E09A	105452	89601	469802	487041	469802	575254	false
	HSA 19	64887753	64968936	51884235	51996896	-	#64E09A	81183	112661	487041	120763	487041	568224	false
	HSA 19	65089699	65253070	51777816	51858509	-	#64E09A	163371	80693	120763	-156966	120763	284134	false
	HSA 19	65096104	65443788	51542220	51803376	-	#64E09A	347684	261156	-156966	13770	-156966	190718	false
	HSA 19	65457558	65634669	51214493	51427529	-	#64E09A	177111	213036	13770	-30493	13770	190881	false
	HSA 19	65604176	66499688	50143646	51179719	-	#64E09A	895512	1036073	-30493	-468234	-30493	865019	false
	HSA 19	66031454	66091180	50819076	50876985	+	#64E09A	59726	57909	-468234	409393	-468234	-408508	true
	HSA 19	66500573	67793473	48265680	50067931	-	#64E09A	1292900	1802251	409393	619437	409393	1702293	false
	HSA 19	68412910	68637004	47990809	48204432	-	#64E09A	224094	213623	619437	837360	619437	843531	false
	HSA 19	69474364	70621599	46263667	47886526	-	#64E09A	1147235	1622859	837360	105401	837360	1984595	false

	HSA 19	70727000	71348102	45185490	46029963	-	#64E09A	621102	844473	105401	160026	105401	726503	false
	HSA 19	71508128	71941339	44586544	45193511	-	#64E09A	433211	606967	160026	716	160026	593237	false
	HSA 19	71942055	72112963	44130763	44530128	-	#64E09A	170908	399365	716	15855	716	171624	false
	HSA 19	72128818	72671480	43258346	43881462	-	#64E09A	542662	623116	15855	-37393	15855	558517	false
	HSA 19	72634087	73644567	41755613	42662788	+	#64E09A	1010480	907175	-37393	-444997	-37393	973087	false
	HSA 19	73199570	73652275	41641130	41809113	+	#64E09A	452705	167983	-444997	-377384	-444997	7708	false
	HSA 19	73274891	73593145	41719373	41809113	-	#64E09A	318254	89740	-377384	-318094	-377384	-59130	true
	HSA 19	73275051	73599113	41757276	41809091	-	#64E09A	324062	51815	-318094	-314661	-318094	5968	false
	HSA 19	73284452	73407652	43019006	43078841	+	#64E09A	123200	59835	-314661	-123200	-314661	-191461	true
	HSA 19	73284452	73599985	42522160	42632260	+	#64E09A	315533	110100	-123200	-241101	-123200	192333	false
	HSA 19	73358884	73599986	42503986	42594051	+	#64E09A	241102	90065	-241101	-136878	-241101	1	false
	HSA 19	73463108	73644567	42588378	42663222	-	#64E09A	181459	74844	-136878	-179510	-136878	44581	false
	HSA 19	73465057	73602003	43199933	43333380	+	#64E09A	136946	133447	-179510	35012	-179510	-42564	true
	HSA 19	73637015	73721416	42504005	42594051	-	#64E09A	84401	90046	35012	-60425	35012	119413	false
	HSA 19	73660991	73772520	41685410	41809113	+	#64E09A	111529	123703	-60425	-72551	-60425	51104	false
	HSA 19	73699969	73779474	42504005	42594051	-	#64E09A	79505	90046	-72551	82650	-72551	6954	false
	HSA 19	73862124	73918170	41707589	41763108	+	#64E09A	56046	55519	82650	-56039	82650	138696	false
	HSA 19	73862131	73944669	41637080	41690117	-	#64E09A	82538	53037	-56039	631	-56039	26499	false
	HSA 19	73945300	74212895	41138174	41449754	-	#64E09A	267595	311580	631	15896	631	268226	false
	HSA 19	74228791	74568206	40920115	41018172	+	#64E09A	339415	98057	15896	-138756	15896	355311	false
	HSA 19	74429450	74512771	40944286	41001080	+	#64E09A	83321	56794	-138756	-19848	-138756	-55435	true
	HSA 19	74492923	74650089	40920115	40973424	+	#64E09A	157166	53309	-19848	-43492	-19848	137318	false
	HSA 19	74606597	74694279	41023053	41074142	-	#64E09A	87682	51089	-43492	393313	-43492	44190	false
	HSA 19	75087592	75786895	39793757	40812843	-	#64E09A	699303	1019086	393313	297743	393313	1092616	false
	HSA 19	76084638	77456760	37825668	39569689	-	#64E09A	1372122	1744021	297743	48103	297743	1669865	false
	HSA 19	77504863	77591601	37696336	37780764	+	#64E09A	86738	84428	48103	3340	48103	134841	false
	HSA 19	77594941	77691907	37593726	37692461	-	#64E09A	96966	98735	3340	70255	3340	100306	false
	HSA 19	77762162	77856234	37073387	37268119	+	#64E09A	94072	194732	70255	-2773	70255	164327	false
	HSA 19	77853461	78036931	37332710	37552292	+	#64E09A	183470	219582	-2773	56645	-2773	180697	false
	HSA 19	78093576	78186488	36887552	37005910	+	#64E09A	92912	118358	56645	20399	56645	149557	false
	HSA 19	78206887	78306587	36337722	36531492	+	#64E09A	99700	193770	20399	6818	20399	120099	false
	HSA 19	78313405	78398148	37825668	37918966	+	#64E09A	84743	93298	6818	3673	6818	91561	false

	HSA 19	78401821	78526648	36544326	36769453	+	#64E09A	124827	225127	3673	30	3673	128500	false
	HSA 19	78526678	79059721	35645967	36240348	-	#64E09A	533043	594381	30	43140	30	533073	false
	HSA 19	79102861	80370108	33183404	34506075	+	#64E09A	1267247	1322671	43140	170261	43140	1310387	false
	HSA 19	80540369	80606752	58117779	58223262	+	#64E09A	66383	105483	170261	33160	170261	236644	false
	HSA 19	80639912	80882754	58278779	58574293	+	#64E09A	242842	295514	33160		33160	276002	false
	HSA 21	80964311	84237062	25371677	28859123	+	#007FFF	3272751	3487446		51183			
	HSA 21	84288245	86091250	28923299	30640335	+	#007FFF	1803005	1717036	51183	158298	51183	1854188	false
	HSA 21	86249548	88046474	30636297	32535052	+	#007FFF	1796926	1898755	158298	65	158298	1955224	false
	HSA 21	88046539	88883894	32595786	33480516	+	#007FFF	837355	884730	65		65	837420	false
**CPOR 8**	HSA 12	43733	254108	27149891	27332558	-	#8D3B0C	210375	182667		1832395			
	HSA 22	305237	367062	35508442	35625560	-	#F400E1	61825	117118		4174			
	HSA 22	371236	1648808	33199515	35456085	-	#F400E1	1277572	2256570	4174	20	4174	1281746	false
	HSA 22	1648828	2085917	32387502	33140349	-	#F400E1	437089	752847	20		20	437109	false
	HSA 12	2086503	5960510	101887848	107786213	-	#8D3B0C	3874007	5898365	1832395	1801	1832395	5706402	false
	HSA 12	5962311	7112535	100200391	101832717	-	#8D3B0C	1150224	1632326	1801	1134	1801	1152025	false
	HSA 12	7113669	16979656	88773733	100148402	-	#8D3B0C	9865987	11374669	1134	4611	1134	9867121	false
	HSA 12	16984267	17039335	88713739	88773680	+	#8D3B0C	55068	59941	4611	685	4611	59679	false
	HSA 12	17040020	31687784	71744847	88697237	-	#8D3B0C	14647764	16952390	685	52360	685	14648449	false
	HSA 12	31740144	34038945	69340312	71719722	-	#8D3B0C	2298801	2379410	52360	87229	52360	2351161	false
	HSA 12	34126174	35171635	68258795	69349878	-	#8D3B0C	1045461	1091083	87229	90066	87229	1132690	false
	HSA 12	35261701	39551956	63731511	68258795	-	#8D3B0C	4290255	4527284	90066	-25871	90066	4380321	false
	HSA 12	39526085	40073491	63052971	63551845	-	#8D3B0C	547406	498874	-25871	55025	-25871	521535	false
	HSA 12	40128516	45005196	57689548	63022766	-	#8D3B0C	4876680	5333218	55025	20112	55025	4931705	false
	HSA 12	45025308	46020523	56773052	57637534	-	#8D3B0C	995215	864482	20112	311309	20112	1015327	false
	HSA 12	46331832	46696940	56377056	56815580	-	#8D3B0C	365108	438524	311309	-46395	311309	676417	false
	HSA 12	46650545	47361730	55667422	56365220	-	#8D3B0C	711185	697798	-46395	455013	-46395	664790	false
	HSA 12	47816743	47911268	55512429	55620509	-	#8D3B0C	94525	108080	455013	236183	455013	549538	false
	HSA 12	48147451	48207595	55283613	55370432	-	#8D3B0C	60144	86819	236183	114544	236183	296327	false
	HSA 12	48322139	48490550	55248064	55453539	+	#8D3B0C	168411	205475	114544	125712	114544	282955	false
	HSA 12	48616262	48716652	55364301	55453392	+	#8D3B0C	100390	89091	125712	508719	125712	226102	false
	HSA 12	49225371	49281651	55253134	55322140	-	#8D3B0C	56280	69006	508719	38395	508719	564999	false
	HSA 12	49320046	49391185	55238969	55322140	-	#8D3B0C	71139	83171	38395	57471	38395	109534	false

HSA 12	49448656	49661054	55108457	55224631	-	#8D3B0C	212398	116174	57471	63637	57471	269869	false
HSA 12	49724691	49917370	54949729	55096950	-	#8D3B0C	192679	147221	63637	8121	63637	256316	false
HSA 12	49925491	53516798	50952569	54768372	-	#8D3B0C	3591307	3815803	8121	1510	8121	3599428	false
HSA 12	53518308	53614426	50934467	51041576	-	#8D3B0C	96118	107109	1510	2689	1510	97628	false
HSA 12	53617115	55304350	49129749	51038041	-	#8D3B0C	1687235	1908292	2689	-28499	2689	1689924	false
HSA 12	55275851	55879444	48554386	49129749	-	#8D3B0C	603593	575363	-28499	184660	-28499	575094	false
HSA 12	56064104	56147892	48430899	48523350	-	#8D3B0C	83788	92451	184660	47913	184660	268448	false
HSA 12	56195805	57159769	47514544	48443087	-	#8D3B0C	963964	928543	47913	0	47913	1011877	false
HSA 12	57159769	57215491	47458153	47510641	+	#8D3B0C	55722	52488	0	-47259	0	55722	false
HSA 12	57168232	62179203	42265159	47460185	-	#8D3B0C	5010971	5195026	-47259	22315	-47259	4963712	false
HSA 12	62201518	66077581	38367791	42339259	-	#8D3B0C	3876063	3971468	22315	59348	22315	3898378	false
HSA 12	66136929	66265067	38209113	38291177	-	#8D3B0C	128138	82064	59348	-128138	59348	187486	false
HSA 12	66136929	68117680	32063836	33997133	-	#8D3B0C	1980751	1933297	-128138	71242	-128138	1852613	false
HSA 12	68188922	68256260	29720230	29801418	+	#8D3B0C	67338	81188	71242	66330	71242	138580	false
HSA 12	68322590	68401208	31955643	32055908	-	#8D3B0C	78618	100265	66330	299959	66330	144948	false
HSA 12	68701167	69137380	31249288	31839479	-	#8D3B0C	436213	590191	299959	2857	299959	736172	false
HSA 12	69140237	69322060	30616918	30791643	-	#8D3B0C	181823	174725	2857	63207	2857	184680	false
HSA 12	69385267	69514524	30380699	30532212	-	#8D3B0C	129257	151513	63207	38	63207	192464	false
HSA 12	69514562	72401448	27508855	30282681	-	#8D3B0C	2886886	2773826	38	-2552722	38	2886924	false
HSA 12	69848726	69965103	29507157	29612031	-	#8D3B0C	116377	104874	-2552722	71801	-2552722	-2436345	true
HSA 12	70036904	70150693	30190051	30282142	+	#8D3B0C	113789	92091	71801	-79582	71801	185590	false
HSA 12	70071111	70324637	29916678	30171176	-	#8D3B0C	253526	254498	-79582	1879439	-79582	173944	false
HSA 12	72204076	72296326	27063978	27148407	-	#8D3B0C	92250	84429	1879439		1879439	1971689	false
HSA 14	72636554	72749692	105574438	105649642	+	#FFFF00	113138	75204		94283			
HSA 14	72843975	73066777	105849995	105924710	+	#FFFF00	222802	74715	94283		94283	317085	false
HSA 2	79284937	79551842	37055	684748	+	#036A9C	266905	647693		506			
HSA 2	79552348	79662028	1038494	1548270	-	#036A9C	109680	509776	506	56831	506	110186	false
HSA 2	79718859	83718620	1581812	9090765	-	#036A9C	3999761	7508953	56831	9474	56831	4056592	false
HSA 2	83728094	84071971	9157614	9731412	+	#036A9C	343877	573798	9474	424147	9474	353351	false
HSA 2	84496118	84943310	16066193	16741051	-	#036A9C	447192	674858	424147	32221	424147	871339	false
HSA 2	84975531	85157727	15510792	15863551	-	#036A9C	182196	352759	32221	105558	32221	214417	false
HSA 15	85170486	85234493	65037952	65145938	+	#EC83BA	64007	107986		1483784			

HSA 2	85263285	88288224	11133044	15310187	-	#036A9C	3024939	4177143	105558	1308	105558	3130497	false
HSA 15	86718277	86775183	20363215	20428922	+	#EC83BA	56906	65707	1483784	2113934	1483784	1540690	false
HSA 2	88289532	88754683	9828094	11074848	-	#036A9C	465151	1246754	1308	2257139	1308	466459	false
HSA 15	88889117	90716551	56357668	58684334	-	#EC83BA	1827434	2326666	2113934	68880	2113934	3941368	false
HSA 15	90785431	90906168	64958289	65037359	+	#EC83BA	120737	79070	68880	511665	68880	189617	false
HSA 2	91011822	91210395	14831964	15239204	-	#036A9C	198573	407240	2257139	0	2257139	2455712	false
HSA 2	91210395	91389564	14494565	14688260	-	#036A9C	179169	193695	0	4614735	0	179169	false
HSA 15	91417833	91971949	74318820	75051177	+	#EC83BA	554116	732357	511665	36480	511665	1065781	false
HSA 15	92008429	92090918	75151679	75251551	+	#EC83BA	82489	99872	36480	-37786	36480	118969	false
HSA 15	92053132	92325796	75335956	75736397	+	#EC83BA	272664	400441	-37786	-33482	-37786	234878	false
HSA 15	92292314	93960809	75798079	77895321	-	#EC83BA	1668495	2097242	-33482	-8462	-33482	1635013	false
HSA 15	93952347	94482573	77967802	78713705	-	#EC83BA	530226	745903	-8462	-20335	-8462	521764	false
HSA 15	94462238	94517885	78758285	78828929	+	#EC83BA	55647	70644	-20335	255552	-20335	35312	false
HSA 15	94773437	95788055	62304737	63466363	-	#EC83BA	1014618	1161626	255552	4	255552	1270170	false
HSA 15	95788059	96004299	61945836	62233484	-	#EC83BA	216240	287648	4	246904	4	216244	false
HSA 2	96004299	96078691	9799803	9953115	-	#036A9C	74392	153312	4614735	76400	4614735	4689127	false
HSA 9	96155091	96229875	40225990	40280497	+	#819EF7	74784	54507		-74784			
HSA 18	96155091	96229903	14182732	14241276	+	#4E1A96	74812	58544		1660399			
HSA 2	96155091	96229903	94799874	94854571	-	#036A9C	74812	54697	76400	1276292	76400	151212	false
HSA 21	96155091	96229903	13918700	13976557	-	#007FFF	74812	57857		1660399			
HSA 9	96155091	96229903	42855276	42909868	+	#819EF7	74812	54592	-74784	-74812	-74784	28	false
HSA 9	96155091	96231340	64074475	64135048	+	#819EF7	76249	60573	-74812	-76249	-74812	1437	false
HSA 9	96155091	96232463	67862019	67919291	+	#819EF7	77372	57272	-76249	1657839	-76249	1123	false
HSA 13	96157980	96229903	18820217	18874747	-	#00FFFF	71923	54530		1660399			
HSA 10	96160894	96223159	27024033	27080089	-	#82C90F	62265	56056		1670536			
HSA 15	96251203	97251978	72723550	74051581	+	#EC83BA	1000775	1328031	246904	0	246904	1247679	false
HSA 15	97251978	97412952	74120388	74343079	+	#EC83BA	160974	222691	0	680510	0	160974	false
HSA 2	97506195	97660594	15282706	15559200	-	#036A9C	154399	276494	1276292	38	1276292	1430691	false
HSA 2	97660632	97871720	14186986	14402006	-	#036A9C	211088	215020	38	58776	38	211126	false
HSA 18	97890302	98002131	14184728	14238315	-	#4E1A96	111829	53587	1660399	-37145	1660399	1772228	false
HSA 9	97890302	98002131	64076478	64130533	-	#819EF7	111829	54055	1657839	3815075	1657839	1769668	false
HSA 13	97890302	98007559	18823183	18873442	+	#00FFFF	117257	50259	1660399	3812183	1660399	1777656	false

	HSA 21	97890302	98007559	13921666	13972747	+	#007FFF	117257	51081	1660399	3812183	1660399	1777656	false
	HSA 10	97893695	97990753	27024033	27089379	+	#82C90F	97058	65346	1670536	29473	1670536	1767594	false
	HSA 2	97930496	98002131	97520922	97584276	+	#036A9C	71635	63354	58776	303960	58776	130411	false
	HSA 18	97964986	98071953	14753346	14839896	-	#4E1A96	106967	86550	-37145	3747789	-37145	69822	false
	HSA 10	98020226	98071953	37130714	37189059	-	#82C90F	51727	58345	29473	3745474	29473	81200	false
	HSA 15	98093462	98221928	61914272	62073569	+	#EC83BA	128466	159297	680510	515815	680510	808976	false
	HSA 2	98306091	98459987	15282706	15559200	-	#036A9C	153896	276494	303960	38	303960	457856	false
	HSA 2	98460025	98610893	14257328	14402006	-	#036A9C	150868	144678	38	3208849	38	150906	false
	HSA 15	98737743	98941653	61724984	61965867	-	#EC83BA	203910	240883	515815	23311	515815	719725	false
	HSA 15	98964964	101761934	58683890	61981067	-	#EC83BA	2796970	3297177	23311	222816	23311	2820281	false
	HSA 2	101819742	101967759	94799874	94854571	+	#036A9C	148017	54697	3208849	2778885	3208849	3356866	false
	HSA 15	101984750	103118061	65147976	66572101	+	#EC83BA	1133311	1424125	222816	664048	222816	1356127	false
	HSA X	103366942	103616692	7040245	7353347	+	#00FF00	249750	313102	12869592	59358	12869592	13119342	false
	HSA X	103676050	103782101	7910287	7996791	+	#00FF00	106051	86504	59358		59358	165409	false
	HSA 15	103782109	103862045	63753166	63823906	-	#EC83BA	79936	70740	664048		664048	743984	false
	HSA 2	104746644	104824411	51003428	51070169	-	#036A9C	77767	66741	2778885		2778885	2856652	false
**CPOR 9**	HSA 5	668075	1376763	179678290	180669574	-	#F33071	708688	991284		5454			
	HSA 5	1382217	1966681	178859715	179627338	-	#F33071	584464	767623	5454	229905	5454	589918	false
	HSA 5	2196586	2580474	178064522	178634149	-	#F33071	383888	569627	229905	269335	229905	613793	false
	HSA 5	2849809	3129643	151000851	151354939	-	#F33071	279834	354088	269335	109	269335	549169	false
	HSA 5	3129752	6254649	131137458	134735661	+	#F33071	3124897	3598203	109	77611	109	3125006	false
	HSA 5	6332260	7281990	151395227	152456968	+	#F33071	949730	1061741	77611	4290	77611	1027341	false
	HSA 5	7286280	11531408	152388046	157089854	+	#F33071	4245128	4701808	4290	117326	4290	4249418	false
	HSA 5	11648734	11699887	156966512	157069837	+	#F33071	51153	103325	117326	122586	117326	168479	false
	HSA 5	11822473	11918255	156932455	157069300	-	#F33071	95782	136845	122586	171	122586	218368	false
	HSA 5	11918426	12040747	157003172	157104389	-	#F33071	122321	101217	171	39713	171	122492	false
	HSA 5	12080460	29706221	156932455	175958223	+	#F33071	17625761	19025768	39713	83785	39713	17665474	false
	HSA 5	29790006	31168400	176291834	177613302	+	#F33071	1378394	1321468	83785	75387	83785	1462179	false
	HSA 5	31243787	32201979	134713644	135730270	+	#F33071	958192	1016626	75387	133	75387	1033579	false
	HSA 5	32202112	34263280	135813256	137889254	+	#F33071	2061168	2075998	133	6	133	2061301	false
	HSA 5	34263286	39066354	112940839	117709773	+	#F33071	4803068	4768934	6	12175	6	4803074	false
	HSA 5	39078529	39292252	117723873	117957997	-	#F33071	213723	234124	12175	29	12175	225898	false

	HSA 5	39292281	41003651	117958518	119636394	+	#F33071	1711370	1677876	29	19596	29	1711399	false
	HSA 5	41023247	41224277	119649310	119864423	-	#F33071	201030	215113	19596	0	19596	220626	false
	HSA 5	41224277	41752972	119864990	120374635	-	#F33071	528695	509645	0	8896	0	528695	false
	HSA 5	41761868	42377458	120375083	120921505	+	#F33071	615590	546422	8896		8896	624486	false
	HSA 8	42789355	50818502	133995976	142843437	-	#80797D	8029147	8847461		56314			
	HSA 8	50874816	53040333	131461261	133930031	-	#80797D	2165517	2468770	56314	123	56314	2221831	false
	HSA 8	53040456	59761724	123822049	131368712	-	#80797D	6721268	7546663	123	172260	123	6721391	false
	HSA 8	59933984	61943433	121375490	123815951	-	#80797D	2009449	2440461	172260	52323	172260	2181709	false
	HSA 8	61995756	62334418	120986762	121365251	-	#80797D	338662	378489	52323	720709	52323	390985	false
	HSA 8	63055127	63187498	142908359	143045361	-	#80797D	132371	137002	720709	98967	720709	853080	false
	HSA 8	63286465	64016915	142986536	143886984	+	#80797D	730450	900448	98967	70495	98967	829417	false
	HSA 8	64087410	64870801	143914239	144756069	+	#80797D	783391	841830	70495	44362	70495	853886	false
	HSA 8	64915163	64971865	144770704	144845817	-	#80797D	56702	75113	44362	11982	44362	101064	false
	HSA 8	64983847	65051409	144874782	144953666	+	#80797D	67562	78884	11982		11982	79544	false
	HSA 12	65105595	66826945	952073	2745935	+	#8D3B0C	1721350	1793862					
**CPOR X**	HSA X	0	455033	155462362	155942085	-	#00FF00	455033	479723		2387			
	HSA X	457420	1191528	154656174	155383049	-	#00FF00	734108	726875	2387	202838	2387	736495	false
	HSA X	1394366	1660428	154219582	154553010	-	#00FF00	266062	333428	202838	-34950	202838	468900	false
	HSA X	1625478	2329554	153260134	154219078	-	#00FF00	704076	958944	-34950	394978	-34950	669126	false
	HSA X	2724532	2907762	152807869	153043973	-	#00FF00	183230	236104	394978	10437	394978	578208	false
	HSA X	2918199	3474360	152104222	152670628	-	#00FF00	556161	566406	10437	-44213	10437	566598	false
	HSA X	3430147	3668307	151926406	152129275	+	#00FF00	238160	202869	-44213	-33468	-44213	193947	false
	HSA X	3634839	4009305	151497741	151932937	-	#00FF00	374466	435196	-33468	84506	-33468	340998	false
	HSA X	4093811	4338297	151151587	151495336	-	#00FF00	244486	343749	84506	22070	84506	328992	false
	HSA X	4360367	5253184	149927950	151001748	-	#00FF00	892817	1073798	22070	-19697	22070	914887	false
	HSA X	5233487	5329616	149531376	149636879	+	#00FF00	96129	105503	-19697	1012257	-19697	76432	false
	HSA X	6341873	6468248	149638176	149704233	+	#00FF00	126375	66057	1012257	93593	1012257	1138632	false
	HSA X	6561841	6621334	149638176	149704233	+	#00FF00	59493	66057	93593	232575	93593	153086	false
	HSA X	6853909	8983753	147176614	149552179	-	#00FF00	2129844	2375565	232575	-10488	232575	2362419	false
	HSA X	8973265	9198883	146969015	147219203	+	#00FF00	225618	250188	-10488	46230	-10488	215130	false
	HSA X	9245113	9423581	146766199	146931830	+	#00FF00	178468	165631	46230	0	46230	224698	false
	HSA X	9423581	10739052	145361122	146764131	-	#00FF00	1315471	1403009	0	57152	0	1315471	false

	HSA X	10796204	10913144	145281212	145361122	-	#00FF00	116940	79910	57152	195373	57152	174092	false
	HSA X	11108517	11176942	143466153	143559002	+	#00FF00	68425	92849	195373	-27787	195373	263798	false
	HSA X	11149155	11450446	145002404	145279696	-	#00FF00	301291	277292	-27787	2887	-27787	273504	false
	HSA X	11453333	11547151	144861769	145002404	+	#00FF00	93818	140635	2887	691	2887	96705	false
	HSA X	11547842	11824961	144482948	144861727	-	#00FF00	277119	378779	691	1501	691	277810	false
	HSA X	11826462	13338927	142796550	144423729	-	#00FF00	1512465	1627179	1501	5581	1501	1513966	false
	HSA X	13344508	13405159	142705200	142787261	+	#00FF00	60651	82061	5581	3998	5581	66232	false
	HSA X	13409157	13897909	142162379	142697994	-	#00FF00	488752	535615	3998	-12731	3998	492750	false
	HSA X	13885178	14086627	141904591	142148667	+	#00FF00	201449	244076	-12731	1199	-12731	188718	false
	HSA X	14087826	14408163	141473986	141860993	-	#00FF00	320337	387007	1199	-75210	1199	321536	false
	HSA X	14332953	14663500	141035404	141472695	+	#00FF00	330547	437291	-75210	356	-75210	255337	false
	HSA X	14663856	14791246	140887782	140995024	-	#00FF00	127390	107242	356	59046	356	127746	false
	HSA X	14850292	19822646	135929543	140879318	-	#00FF00	4972354	4949775	59046	-37616	59046	5031400	false
	HSA X	19785030	19871009	135710332	135800573	+	#00FF00	85979	90241	-37616	-85979	-37616	48363	false
	HSA X	19785030	19881451	135681772	135788678	-	#00FF00	96421	106906	-85979	-93276	-85979	10442	false
	HSA X	19788175	19884409	135590063	135687485	+	#00FF00	96234	97422	-93276	-9779	-93276	2958	false
	HSA X	19874630	20033327	135420927	135583159	-	#00FF00	158697	162232	-9779	-18059	-9779	148918	false
	HSA X	20015268	20220374	135135747	135195662	-	#00FF00	205106	59915	-18059	-33444	-18059	187047	false
	HSA X	20186930	20283240	135334970	135449316	-	#00FF00	96310	114346	-33444	-16457	-33444	62866	false
	HSA X	20266783	20323531	135346980	135401766	-	#00FF00	56748	54786	-16457	37267	-16457	40291	false
	HSA X	20360798	20427605	135130403	135216343	-	#00FF00	66807	85940	37267	-66807	37267	104074	false
	HSA X	20360798	20453805	135179165	135295098	+	#00FF00	93007	115933	-66807	370918	-66807	26200	false
	HSA X	20824723	21040273	135005461	135105927	-	#00FF00	215550	100466	370918	-103376	370918	586468	false
	HSA X	20936897	21095006	135254022	135386109	-	#00FF00	158109	132087	-103376	-30177	-103376	54733	false
	HSA X	21064829	21127672	135346954	135400967	-	#00FF00	62843	54013	-30177	2159121	-30177	32666	false
	HSA 17	21968833	22038314	58579000	58634616	-	#FF0000	69481	55616		281415			
	HSA 17	22319729	22383198	58579000	58634616	-	#FF0000	63469	55616	281415		281415	344884	false
	HSA X	23286793	26384463	131803786	135001616	-	#00FF00	3097670	3197830	2159121	200620	2159121	5256791	false
	HSA X	26585083	26850185	131444520	131678741	-	#00FF00	265102	234221	200620	-85193	200620	465722	false
	HSA X	26764992	28502208	129897385	131478331	-	#00FF00	1737216	1580946	-85193	370611	-85193	1652023	false
	HSA X	28872819	30175806	128659521	129896798	-	#00FF00	1302987	1237277	370611	370544	370611	1673598	false
	HSA X	30546350	30984007	128254440	128656805	-	#00FF00	437657	402365	370544	63239	370544	808201	false

	HSA X	31047246	31172328	128125699	128213218	-	#00FF00	125082	87519	63239	-1696	63239	188321	false
	HSA X	31170632	31290248	127950816	128052587	-	#00FF00	119616	101771	-1696	56925	-1696	117920	false
	HSA X	31347173	31453648	127830230	127950808	-	#00FF00	106475	120578	56925	187732	56925	163400	false
	HSA X	31641380	31795702	127697394	127830330	+	#00FF00	154322	132936	187732	209459	187732	342054	false
	HSA X	32005161	32097177	127393721	127471782	+	#00FF00	92016	78061	209459	69901	209459	301475	false
	HSA X	32167078	32658785	126953081	127335159	-	#00FF00	491707	382078	69901	82010	69901	561608	false
	HSA X	32740795	35294125	124247808	126894449	-	#00FF00	2553330	2646641	82010	55084	82010	2635340	false
	HSA X	35349209	35543079	124188941	124247808	-	#00FF00	193870	58867	55084	0	55084	248954	false
	HSA X	35543079	38839999	120943263	124135424	-	#00FF00	3296920	3192161	0	-3231	0	3296920	false
	HSA X	38836768	39550463	120201582	120876436	-	#00FF00	713695	674854	-3231	271009	-3231	710464	false
	HSA X	39821472	40033604	119969704	120058164	-	#00FF00	212132	88460	271009	2771	271009	483141	false
	HSA X	40036375	42696717	117091997	119728432	-	#00FF00	2660342	2636435	2771	57842	2771	2663113	false
	HSA X	42754559	43024376	116825197	117069499	-	#00FF00	269817	244302	57842	187565	57842	327659	false
	HSA X	43211941	43614736	116220806	116658594	-	#00FF00	402795	437788	187565	66012	187565	590360	false
	HSA X	43680748	43796512	116135608	116212362	-	#00FF00	115764	76754	66012	308885	66012	181776	false
	HSA X	44105397	45616589	114332544	115727199	-	#00FF00	1511192	1394655	308885	15766	308885	1820077	false
	HSA X	45632355	46785711	113451720	114279885	-	#00FF00	1153356	828165	15766	69991	15766	1169122	false
	HSA X	46855702	47061181	113274738	113445342	-	#00FF00	205479	170604	69991	33218	69991	275470	false
	HSA X	47094399	47156624	113159617	113231101	+	#00FF00	62225	71484	33218	187764	33218	95443	false
	HSA X	47344388	50272562	110646173	113130467	-	#00FF00	2928174	2484294	187764	0	187764	3115938	false
	HSA X	50272562	50354363	110551831	110639969	+	#00FF00	81801	88138	0	0	0	81801	false
	HSA X	50354363	51931832	109221364	110546662	-	#00FF00	1577469	1325298	0	181143	0	1577469	false
	HSA X	52112975	52213424	108960179	109052423	-	#00FF00	100449	92244	181143	256169	181143	281592	false
	HSA X	52469593	54472729	107061750	108751471	-	#00FF00	2003136	1689721	256169	191501	256169	2259305	false
	HSA X	54664230	55159994	106591411	107019072	-	#00FF00	495764	427661	191501	106782	191501	687265	false
	HSA X	55266776	55322902	106437184	106511965	+	#00FF00	56126	74781	106782	26001	106782	162908	false
	HSA X	55348903	55529864	106459911	106591071	+	#00FF00	180961	131160	26001	92149	26001	206962	false
	HSA X	55622013	55686920	106368057	106479623	-	#00FF00	64907	111566	92149	63683	92149	157056	false
	HSA X	55750603	55857705	106216239	106350308	+	#00FF00	107102	134069	63683	-107102	63683	170785	false
	HSA X	55750603	55928020	106102600	106349889	-	#00FF00	177417	247289	-107102	-3590	-107102	70315	false
	HSA X	55924430	57980868	104005098	106044838	-	#00FF00	2056438	2039740	-3590	19292	-3590	2052848	false
	HSA X	58000160	58314082	103524798	103847035	-	#00FF00	313922	322237	19292	9393	19292	333214	false

	HSA X	58323475	58540762	103219168	103452590	-	#00FF00	217287	233422	9393	8018	9393	226680	false
	HSA X	58548780	58642305	102693410	102759409	-	#00FF00	93525	65999	8018	52783	8018	101543	false
	HSA X	58695088	58769220	102568077	102682056	-	#00FF00	74132	113979	52783	34181	52783	126915	false
	HSA X	58803401	58861697	102270349	102368216	+	#00FF00	58296	97867	34181	92322	34181	92477	false
	HSA X	58954019	59011398	102085739	102148840	-	#00FF00	57379	63101	92322	14	92322	149701	false
	HSA X	59011412	59075140	103092569	103159055	+	#00FF00	63728	66486	14	5574	14	63742	false
	HSA X	59080714	59511725	101595449	101994222	-	#00FF00	431011	398773	5574	52054	5574	436585	false
	HSA X	59563779	59721411	101343531	101563221	-	#00FF00	157632	219690	52054	15828	52054	209686	false
	HSA X	59737239	61348961	100002491	101293459	-	#00FF00	1611722	1290968	15828	66747	15828	1627550	false
	HSA X	61415708	61924404	99585943	99986126	-	#00FF00	508696	400183	66747	50442	66747	575443	false
	HSA X	61974846	62290725	99248965	99531181	-	#00FF00	315879	282216	50442	4493	50442	366321	false
	HSA X	62295218	62781925	98948248	99196196	-	#00FF00	486707	247948	4493	212249	4493	491200	false
	HSA X	62994174	63392851	98551328	98829222	-	#00FF00	398677	277894	212249	56178	212249	610926	false
	HSA X	63449029	64355894	97825128	98534243	-	#00FF00	906865	709115	56178	255757	56178	963043	false
	HSA X	64611651	65817965	96571565	97713976	-	#00FF00	1206314	1142411	255757	169218	255757	1462071	false
	HSA X	65987183	66129503	96431894	96503640	-	#00FF00	142320	71746	169218	82061	169218	311538	false
	HSA X	66211564	67045056	95951098	96428545	-	#00FF00	833492	477447	82061	136069	82061	915553	false
	HSA X	67181125	67663277	95619698	95914868	-	#00FF00	482152	295170	136069	4088	136069	618221	false
	HSA X	67667365	67826386	95223286	95422145	-	#00FF00	159021	198859	4088	633175	4088	163109	false
	HSA X	68459561	68726536	91708731	91844949	+	#00FF00	266975	136218	633175	206870	633175	900150	false
	HSA X	68933406	69144265	92144580	92267480	+	#00FF00	210859	122900	206870	385700	206870	417729	false
	HSA X	69529965	69723820	87513699	87670164	-	#00FF00	193855	156465	385700	1834	385700	579555	false
	HSA X	69725654	69861348	87301490	87432348	-	#00FF00	135694	130858	1834	216121	1834	137528	false
	HSA X	70077469	70257336	85859839	86057124	-	#00FF00	179867	197285	216121	161242	216121	395988	false
	HSA X	70418578	71113700	84997100	85549412	-	#00FF00	695122	552312	161242	111502	161242	856364	false
	HSA X	71225202	71461182	84269422	84526348	-	#00FF00	235980	256926	111502	41235	111502	347482	false
	HSA X	71502417	71762132	84035036	84190349	-	#00FF00	259715	155313	41235	394718	41235	300950	false
	HSA X	72156850	72412692	83415551	83576920	-	#00FF00	255842	161369	394718	918588	394718	650560	false
	HSA X	73331280	73399827	81194379	81298283	-	#00FF00	68547	103904	918588	252502	918588	987135	false
	HSA X	73652329	73883874	80650268	80811091	-	#00FF00	231545	160823	252502	196368	252502	484047	false
	HSA X	74080242	74152793	80257231	80346135	-	#00FF00	72551	88904	196368	57092	196368	268919	false
	HSA X	74209885	74392133	79139763	79272541	-	#00FF00	182248	132778	57092	61576	57092	239340	false

	HSA X	74453709	74556082	79069986	79137228	-	#00FF00	102373	67242	61576	413239	61576	163949	false
	HSA X	74969321	75070328	78268565	78334313	-	#00FF00	101007	65748	413239	32520	413239	514246	false
	HSA X	75102848	75622119	77774226	78150623	-	#00FF00	519271	376397	32520	15302	32520	551791	false
	HSA X	75637421	76008668	77419361	77723998	-	#00FF00	371247	304637	15302	167954	15302	386549	false
	HSA X	76176622	76271408	77341841	77419055	-	#00FF00	94786	77214	167954	223405	167954	262740	false
	HSA X	76494813	76667075	76854279	77098669	-	#00FF00	172262	244390	223405	1000236	223405	395667	false
	HSA X	77667311	78031015	75228490	75517138	-	#00FF00	363704	288648	1000236	193400	1000236	1363940	false
	HSA X	78224415	78854327	74728595	75161693	-	#00FF00	629912	433098	193400	217773	193400	823312	false
	HSA X	79072100	79145273	74570279	74632799	-	#00FF00	73173	62520	217773	189306	217773	290946	false
	HSA X	79334579	79421607	74410591	74468384	+	#00FF00	87028	57793	189306	276485	189306	276334	false
	HSA X	79698092	79842985	74233183	74313066	-	#00FF00	144893	79883	276485	164832	276485	421378	false
	HSA X	80007817	80080368	73940817	73997827	-	#00FF00	72551	57010	164832	246195	164832	237383	false
	HSA X	80326563	80393851	73726269	73792534	-	#00FF00	67288	66265	246195	818496	246195	313483	false
	HSA X	81212347	81750235	72294492	72810369	-	#00FF00	537888	515877	818496	56783	818496	1356384	false
	HSA X	81807018	82322069	71857676	72282034	-	#00FF00	515051	424358	56783	-23288	56783	571834	false
	HSA X	82298781	82960600	71037081	71661441	-	#00FF00	661819	624360	-23288	523198	-23288	638531	false
	HSA X	83483798	83657362	70801778	70956310	-	#00FF00	173564	154532	523198	212239	523198	696762	false
	HSA X	83869601	85314901	69488162	70563129	-	#00FF00	1445300	1074967	212239	55694	212239	1657539	false
	HSA X	85370595	86208413	68790547	69474125	-	#00FF00	837818	683578	55694	210442	55694	893512	false
	HSA X	86418855	86668146	63632161	63838642	+	#00FF00	249291	206481	210442	173964	210442	459733	false
	HSA X	86842110	86943123	64178588	64274194	-	#00FF00	101013	95606	173964	162608	173964	274977	false
	HSA X	87105731	87312227	64913939	65114429	+	#00FF00	206496	200490	162608	413353	162608	369104	false
	HSA X	87725580	88050081	65495242	65747493	+	#00FF00	324501	252251	413353	65128	413353	737854	false
	HSA X	88115209	88524167	65961805	66288762	+	#00FF00	408958	326957	65128	13036	65128	474086	false
	HSA X	88537203	88628302	66379478	66449636	+	#00FF00	91099	70158	13036	261833	13036	104135	false
	HSA X	88890135	89078101	66589898	66816140	+	#00FF00	187966	226242	261833	60305	261833	449799	false
	HSA X	89138406	89264363	66866738	66921495	+	#00FF00	125957	54757	60305	74385	60305	186262	false
	HSA X	89338748	89459537	67017410	67143607	+	#00FF00	120789	126197	74385	134264	74385	195174	false
	HSA X	89593801	89701067	67257697	67351785	+	#00FF00	107266	94088	134264	259014	134264	241530	false
	HSA X	89960081	90301968	67520132	67790186	+	#00FF00	341887	270054	259014	272786	259014	600901	false
	HSA X	90574754	91278735	67933506	68583798	+	#00FF00	703981	650292	272786	767349	272786	976767	false
	HSA X	92046084	92117598	57896856	57962355	+	#00FF00	71514	65499	767349	824809	767349	838863	false

	HSA X	92942407	93081792	56224209	56302272	-	#00FF00	139385	78063	824809	77545	824809	964194	false
	HSA X	93159337	93239264	55714126	55795399	-	#00FF00	79927	81273	77545	190727	77545	157472	false
	HSA X	93429991	93591962	54912945	55036803	-	#00FF00	161971	123858	190727	168274	190727	352698	false
	HSA X	93760236	93815099	54723470	54790593	+	#00FF00	54863	67123	168274	-17068	168274	223137	false
	HSA X	93798031	93919284	54773482	54844186	-	#00FF00	121253	70704	-17068	122113	-17068	104185	false
	HSA X	94041397	94221189	54439839	54563139	-	#00FF00	179792	123300	122113	126789	122113	301905	false
	HSA X	94347978	94800656	54092732	54403431	-	#00FF00	452678	310699	126789	80625	126789	579467	false
	HSA X	94881281	95089781	53929818	54073350	-	#00FF00	208500	143532	80625	452502	80625	289125	false
	HSA X	95542283	96366388	52914720	53716174	-	#00FF00	824105	801454	452502	-145313	452502	1276607	false
	HSA X	96221075	96366388	52887570	52941093	+	#00FF00	145313	53523	-145313	174221	-145313	0	true
	HSA X	96540609	96667105	52114331	52212257	-	#00FF00	126496	97926	174221	137240	174221	300717	false
	HSA X	96804345	97106503	51710342	51942284	-	#00FF00	302158	231942	137240	53584	137240	439398	false
	HSA X	97160087	97357685	51378463	51619037	-	#00FF00	197598	240574	53584	131933	53584	251182	false
	HSA X	97489618	97563554	50987338	51047161	-	#00FF00	73936	59823	131933	202083	131933	205869	false
	HSA X	97765637	98334512	50535627	50927717	-	#00FF00	568875	392090	202083	110702	202083	770958	false
	HSA X	98445214	98892645	50280289	50528151	-	#00FF00	447431	247862	110702	76006	110702	558133	false
	HSA X	98968651	99582204	49533316	50146582	-	#00FF00	613553	613266	76006	-2612	76006	689559	false
	HSA X	99579592	100388554	48391758	49346447	-	#00FF00	808962	954689	-2612	-1601	-2612	806350	false
	HSA X	100386953	100874313	47777365	48166579	-	#00FF00	487360	389214	-1601	13316	-1601	485759	false
	HSA X	100887629	101251819	47363797	47663945	-	#00FF00	364190	300148	13316	95582	13316	377506	false
	HSA X	101347401	102105001	46461941	47301499	-	#00FF00	757600	839558	95582	-66851	95582	853182	false
	HSA X	102038150	102110906	46432090	46547257	+	#00FF00	72756	115167	-66851	-37949	-66851	5905	false
	HSA X	102072957	103167190	45640515	46458957	-	#00FF00	1094233	818442	-37949	55248	-37949	1056284	false
	HSA X	103222438	103378649	45567250	45624303	-	#00FF00	156211	57053	55248	52297	55248	211459	false
	HSA X	103430946	103662806	45425158	45566813	-	#00FF00	231860	141655	52297	15916	52297	284157	false
	HSA X	103678722	111974210	37473537	45364699	-	#00FF00	8295488	7891162	15916	-111638	15916	8311404	false
	HSA X	111862572	111976192	36968330	37054804	+	#00FF00	113620	86474	-111638	190912	-111638	1982	false
	HSA X	112167104	112429816	37291193	37443025	+	#00FF00	262712	151832	190912	161	190912	453624	false
	HSA X	112429977	112625857	36877803	36966884	-	#00FF00	195880	89081	161	-192639	161	196041	false
	HSA X	112433218	112628510	34656044	34811712	+	#00FF00	195292	155668	-192639	28004	-192639	2653	false
	HSA X	112656514	112865510	36783269	36908657	-	#00FF00	208996	125388	28004	-118052	28004	237000	false
	HSA X	112747458	112862798	34891480	35026830	+	#00FF00	115340	135350	-118052	14035	-118052	-2712	true

	HSA X	112876833	112946535	36643257	36721544	-	#00FF00	69702	78287	14035	181	14035	83737	false
	HSA X	112946716	113007167	36520801	36582461	-	#00FF00	60451	61660	181	117585	181	60632	false
	HSA X	113124752	115411228	34561726	36517125	-	#00FF00	2286476	1955399	117585	-1992258	117585	2404061	false
	HSA X	113418970	113499203	35637986	35710449	+	#00FF00	80233	72463	-1992258	432969	-1992258	-1912025	true
	HSA X	113932172	114353854	36155660	36404656	+	#00FF00	421682	248996	432969	53345	432969	854651	false
	HSA X	114407199	114677234	36478201	36678475	+	#00FF00	270035	200274	53345	62635	53345	323380	false
	HSA X	114739869	114903176	36787245	36870486	+	#00FF00	163307	83241	62635	561301	62635	225942	false
	HSA X	115464477	115608844	34362160	34536520	-	#00FF00	144367	174360	561301	904962	561301	705668	false
	HSA X	116513806	116928915	34171790	34348331	-	#00FF00	415109	176541	904962	18886	904962	1320071	false
	HSA X	116947801	121163712	30218581	34116023	-	#00FF00	4215911	3897442	18886	152847	18886	4234797	false
	HSA X	121316559	123523856	28126465	30261979	-	#00FF00	2207297	2135514	152847	52006	152847	2360144	false
	HSA X	123575862	124461801	27330296	28124919	-	#00FF00	885939	794623	52006	-34928	52006	937945	false
	HSA X	124426873	125928140	26148831	27330296	-	#00FF00	1501267	1181465	-34928	-293540	-34928	1466339	false
	HSA X	125634600	125989183	26158539	26225698	-	#00FF00	354583	67159	-293540	7681	-293540	61043	false
	HSA X	125996864	126122317	26154670	26250533	+	#00FF00	125453	95863	7681	17653	7681	133134	false
	HSA X	126139970	126346205	26154670	26224561	+	#00FF00	206235	69891	17653	-105090	17653	223888	false
	HSA X	126241115	126353065	26148831	26210229	+	#00FF00	111950	61398	-105090	44729	-105090	6860	false
	HSA X	126397794	126551132	26154670	26218010	-	#00FF00	153338	63340	44729	-153338	44729	198067	false
	HSA X	126397794	129222472	23570046	26224561	-	#00FF00	2824678	2654515	-153338	126617	-153338	2671340	false
	HSA X	129349089	134376022	18379797	23569176	-	#00FF00	5026933	5189379	126617	50883	126617	5153550	false
	HSA X	134426905	136529392	16327827	18374505	-	#00FF00	2102487	2046678	50883	0	50883	2153370	false
	HSA X	136529392	136645452	16236858	16321408	+	#00FF00	116060	84550	0	-48095	0	116060	false
	HSA X	136597357	136917044	16006747	16264422	-	#00FF00	319687	257675	-48095	37	-48095	271592	false
	HSA X	136917081	137027223	15932415	16006522	+	#00FF00	110142	74107	37	0	37	110179	false
	HSA X	137027223	139802390	13397245	15932396	-	#00FF00	2775167	2535151	0	50834	0	2775167	false
	HSA X	139853224	140242217	12904581	13344820	-	#00FF00	388993	440239	50834	59589	50834	439827	false
	HSA X	140301806	143151368	9964485	12903481	-	#00FF00	2849562	2938996	59589	640534	59589	2909151	false
	HSA X	143791902	143849092	14137870	14214524	+	#00FF00	57190	76654	640534	949095	640534	697724	false
	HSA X	144798187	144888665	14137870	14233753	-	#00FF00	90478	95883	949095	588712	949095	1039573	false
	HSA X	145477377	145553111	8900602	8960151	+	#00FF00	75734	59549	588712	31905	588712	664446	false
	HSA X	145585016	146416169	9034860	9962184	+	#00FF00	831153	927324	31905	165796	31905	863058	false
	HSA X	146581965	146940549	7023616	7423998	+	#00FF00	358584	400382	165796	23834	165796	524380	false

	HSA X	146964383	147925241	7910287	8869597	+	#00FF00	960858	959310	23834	572934	23834	984692	false
	HSA X	148498175	148564948	7922098	7999565	+	#00FF00	66773	77467	572934	44432	572934	639707	false
	HSA X	148609380	151296005	5195840	8033013	-	#00FF00	2686625	2837173	44432	466881	44432	2731057	false
	HSA X	151762886	151908918	4857884	5143813	-	#00FF00	146032	285929	466881	5885	466881	612913	false
	HSA X	151914803	152016189	4565211	4806941	-	#00FF00	101386	241730	5885	17630	5885	107271	false
	HSA X	152033819	152088404	4099681	4396770	-	#00FF00	54585	297089	17630		17630	72215	false

Supplementary Table S1 (part 2). Syntenic blocks with a minimum cut-off length of 50 kilobases in the reference guinea pig genome.

scaffold	query	start	end	query_start	query_end	strand	color	target_len	query_len	dist_upstream	dist_downstream	dist_end_start	dist_end_end	embedded
CPOR 1	HGL 24	36451	6006258	12691544	18602371	-	#FF0000	5969807	5910827					
	HGL 2	6332332	6481187	120776365	120843099	+	#1CB707	148855	66734		-114895			
	HGL 2	6366292	6502477	120776399	120843031	+	#1CB707	136185	66632	-114895	76060	-114895	21290	false
	HGL 2	6578537	6791053	120776399	120843099	-	#1CB707	212516	66700	76060	30629	76060	288576	false
	HGL 2	6821682	7233143	120776365	120843099	-	#1CB707	411461	66734	30629	-135966	30629	442090	false
	HGL 2	7097177	7169186	120783892	120843099	-	#1CB707	72009	59207	-135966	129460	-135966	-63957	true
	HGL 2	7298646	18961865	108500685	120843099	-	#1CB707	11663219	12342414	129460	-2864733	129460	11792679	false
	HGL 2	16097132	16161283	107494421	107598667	+	#1CB707	64151	104246	-2864733	3256934	-2864733	-2800582	true
	HGL 2	19418217	20906280	106619284	108500330	-	#1CB707	1488063	1881046	3256934	13148	3256934	4744997	false
	HGL 2	20919428	21411860	106064733	106568913	-	#1CB707	492432	504180	13148	64966	13148	505580	false
	HGL 2	21476826	23808955	103444458	106064733	-	#1CB707	2332129	2620275	64966	85351	64966	2397095	false
	HGL 2	23894306	26811302	100239583	103443980	-	#1CB707	2916996	3204397	85351	52557	85351	3002347	false
	HGL 2	26863859	27019400	100051901	100210348	-	#1CB707	155541	158447	52557	41753	52557	208098	false
	HGL 2	27061153	35696068	90802379	100059089	-	#1CB707	8634915	9256710	41753	0	41753	8676668	false
	HGL 2	35696068	36512563	89696667	90743256	-	#1CB707	816495	1046589	0	0	0	816495	false
	HGL 2	36512563	37350295	89870225	90930725	-	#1CB707	837732	1060500	0	50823	0	837732	false
	HGL 2	37401118	38482385	89696667	90921750	-	#1CB707	1081267	1225083	50823	0	50823	1132090	false
	HGL 2	38482385	38556475	90822514	90930725	-	#1CB707	74090	108211	0	3677	0	74090	false
	HGL 2	38560152	38705514	90592201	90770004	-	#1CB707	145362	177803	3677	15243	3677	149039	false
	HGL 2	38720757	39585557	89886366	90930724	+	#1CB707	864800	1044358	15243	0	15243	880043	false
	HGL 2	39585557	40445719	89696667	90591611	+	#1CB707	860162	894944	0	2	0	860162	false
	HGL 2	40445721	47078620	82895219	89881596	-	#1CB707	6632899	6986377	2		2	6632901	false
	HGL 14	47553527	47648088	202252	305836	+	#7F00FF	94561	103584		86907			

	HGL 14	47734995	56969368	320393	10894764	+	#7F00FF	9234373	10574371	86907	-142894	86907	9321280	false
	HGL 14	56826474	56998141	11225761	11357444	-	#7F00FF	171667	131683	-142894	-32795	-142894	28773	false
	HGL 14	56965346	57245095	10658668	10845500	+	#7F00FF	279749	186832	-32795	-245415	-32795	246954	false
	HGL 14	56999680	57245095	11262241	11362958	-	#7F00FF	245415	100717	-245415	-90967	-245415	0	true
	HGL 14	57154128	57351260	10928366	11016747	-	#7F00FF	197132	88381	-90967	-197132	-90967	106165	false
	HGL 14	57154128	57352830	11247401	11336888	-	#7F00FF	198702	89487	-197132	-196433	-197132	1570	false
	HGL 14	57156397	57241761	10774332	10838237	+	#7F00FF	85364	63905	-196433	-85364	-196433	-111069	true
	HGL 14	57156397	57242442	11270965	11334343	-	#7F00FF	86045	63378	-85364	-85698	-85364	681	false
	HGL 14	57156744	57352485	11107303	11214278	+	#7F00FF	195741	106975	-85698	-151339	-85698	110043	false
	HGL 14	57201146	57352830	10772684	10864058	+	#7F00FF	151684	91374	-151339	-60551	-151339	345	false
	HGL 14	57292279	57848320	11383669	11989337	+	#7F00FF	556041	605668	-60551	3816	-60551	495490	false
	HGL 14	57852136	62503484	12074756	17158405	+	#7F00FF	4651348	5083649	3816	76544	3816	4655164	false
	HGL 14	62580028	67227847	17179877	22006840	+	#7F00FF	4647819	4826963	76544	96010	76544	4724363	false
	HGL 14	67323857	81178067	22012907	36471743	+	#7F00FF	13854210	14458836	96010	507175	96010	13950220	false
	HGL 14	81685242	81740476	36452051	36506185	+	#7F00FF	55234	54134	507175	107607	507175	562409	false
	HGL 14	81848083	84159884	36795971	39292936	+	#7F00FF	2311801	2496965	107607	59126	107607	2419408	false
	HGL 14	84219010	90871817	39345380	46959090	+	#7F00FF	6652807	7613710	59126	3571	59126	6711933	false
	HGL 14	90875388	91980048	47019509	48400794	+	#7F00FF	1104660	1381285	3571	60277890	3571	1108231	false
	HGL 20	91991552	92078659	57365092	57435174	-	#00FFFF	87107	70082		-48008			
	HGL 20	92030651	93004689	57365092	58280763	+	#00FFFF	974038	915671	-48008	50084	-48008	926030	false
	HGL 20	93054773	93738288	58312261	58914019	+	#00FFFF	683515	601758	50084	131017	50084	733599	false
	HGL 20	93869305	94330974	58952726	59365610	+	#00FFFF	461669	412884	131017	0	131017	592686	false
	HGL 20	94330974	94579501	59365611	59544019	-	#00FFFF	248527	178408	0	4	0	248527	false
	HGL 20	94579505	95090391	59544022	60005380	+	#00FFFF	510886	461358	4	30327	4	510890	false
	HGL 20	95120718	95190972	59990107	60043894	-	#00FFFF	70254	53787	30327	-29037	30327	100581	false
	HGL 20	95161935	95288431	59952338	60059327	-	#00FFFF	126496	106989	-29037	-58655	-29037	97459	false
	HGL 20	95229776	95835278	59952338	60420832	+	#00FFFF	605502	468494	-58655	53052	-58655	546847	false
	HGL 20	95888330	96247769	60415666	60680689	+	#00FFFF	359439	265023	53052	78280	53052	412491	false
	HGL 20	96326049	96604163	24492895	24716516	+	#00FFFF	278114	223621	78280	-3886	78280	356394	false
	HGL 20	96600277	96670773	24661846	24716516	+	#00FFFF	70496	54670	-3886	155525	-3886	66610	false
	HGL 20	96826298	100105143	24773469	28321157	+	#00FFFF	3278845	3547688	155525	35	155525	3434370	false
	HGL 20	100105178	104201189	28436580	33082079	+	#00FFFF	4096011	4645499	35	-24312	35	4096046	false

	HGL 20	104176877	109902923	33145287	39868019	+	#00FFFF	5726046	6722732	-24312	87602	-24312	5701734	false
	HGL 20	109990525	113971603	39891544	44206349	+	#00FFFF	3981078	4314805	87602	87892	87602	4068680	false
	HGL 20	114059495	115189776	44209288	45279077	+	#00FFFF	1130281	1069789	87892	-95813	87892	1218173	false
	HGL 20	115093963	116311134	45279281	46396870	+	#00FFFF	1217171	1117589	-95813	55062	-95813	1121358	false
	HGL 20	116366196	116534830	46479384	46612562	+	#00FFFF	168634	133178	55062	304142	55062	223696	false
	HGL 20	116838972	116953429	46764679	46892302	+	#00FFFF	114457	127623	304142	67736	304142	418599	false
	HGL 20	117021165	125013361	46899472	54903895	+	#00FFFF	7992196	8004423	67736	164666	67736	8059932	false
	HGL 20	125178027	125500071	54905327	55080681	+	#00FFFF	322044	175354	164666	2307	164666	486710	false
	HGL 20	125502378	125717329	55161587	55413805	-	#00FFFF	214951	252218	2307	7896	2307	217258	false
	HGL 20	125725225	127728414	55436927	57276848	+	#00FFFF	2003189	1839921	7896	0	7896	2011085	false
	HGL 20	127728414	127805360	57293373	57405283	-	#00FFFF	76946	111910	0	116892	0	76946	false
	HGL 20	127922252	127987613	57397941	57481221	+	#00FFFF	65361	83280	116892	74304	116892	182253	false
	HGL 20	128061917	128150505	57397941	57481221	-	#00FFFF	88588	83280	74304	-1869	74304	162892	false
	HGL 20	128148636	128252281	57365092	57442711	+	#00FFFF	103645	77619	-1869	6430	-1869	101776	false
	HGL 20	128258711	128360966	57399526	57481221	-	#00FFFF	102255	81695	6430	112947	6430	108685	false
	HGL 20	128473913	128632358	57365092	57481221	-	#00FFFF	158445	116129	112947	-5285	112947	271392	false
	HGL 20	128627073	128713858	57399526	57481219	+	#00FFFF	86785	81693	-5285	46816	-5285	81500	false
	HGL 20	128760674	131257612	21968915	24494969	-	#00FFFF	2496938	2526054	46816	-246655	46816	2543754	false
	HGL 20	131010957	152093744	14433	22056863	-	#00FFFF	21082787	22042430	-246655	28532844	-246655	20836132	false
	HGL 14	152257938	158774186	71502894	78088954	-	#7F00FF	6516248	6586060	60277890	5260	60277890	66794138	false
	HGL 14	158779446	160304157	69932015	71448777	-	#7F00FF	1524711	1516762	5260	24499	5260	1529971	false
	HGL 14	160328656	160442731	69862547	69963770	+	#7F00FF	114075	101223	24499	-25207	24499	138574	false
	HGL 14	160417524	160521301	69862547	69950915	-	#7F00FF	103777	88368	-25207	361910	-25207	78570	false
	HGL 14	160883211	168163784	61898424	69862525	-	#7F00FF	7280573	7964101	361910	-47547	361910	7642483	false
	HGL 14	168116237	168269127	61744875	61898374	-	#7F00FF	152890	153499	-47547	-23827	-47547	105343	false
	HGL 14	168245300	171138774	58587868	61741574	-	#7F00FF	2893474	3153706	-23827	-10044	-23827	2869647	false
	HGL 14	171128730	175763563	53586924	58501260	-	#7F00FF	4634833	4914336	-10044	119260	-10044	4624789	false
	HGL 14	175882823	176956741	52498773	53586015	-	#7F00FF	1073918	1087242	119260	7848	119260	1193178	false
	HGL 14	176964589	177133438	52030471	52211074	-	#7F00FF	168849	180603	7848	56303	7848	176697	false
	HGL 14	177189741	180593228	48340303	51978268	-	#7F00FF	3403487	3637965	56303	459206	56303	3459790	false
	HGL 20	180626588	180709782	65179004	65269524	+	#00FFFF	83194	90520	28532844	-83194	28532844	28616038	false
	HGL 20	180626588	180721942	64276114	64340779	-	#00FFFF	95354	64665	-83194		-83194	12160	false

	HGL 14	181052434	182067071	78126138	79079824	+	#7F00FF	1014637	953686	459206	67922	459206	1473843	false
	HGL 14	182134993	182255866	79130873	79252006	+	#7F00FF	120873	121133	67922	-20162	67922	188795	false
	HGL 14	182235704	187222289	79365220	84475555	+	#7F00FF	4986585	5110335	-20162	362153	-20162	4966423	false
	HGL 14	187584442	187894079	84513512	84725702	+	#7F00FF	309637	212190	362153	172501	362153	671790	false
	HGL 14	188066580	188126515	84613657	84725702	+	#7F00FF	59935	112045	172501	1040755	172501	232436	false
	HGL 14	189167270	189242701	84923649	84988345	+	#7F00FF	75431	64696	1040755	329215	1040755	1116186	false
	HGL 14	189571916	189632412	85208113	85269953	+	#7F00FF	60496	61840	329215	38028	329215	389711	false
	HGL 14	189670440	196230313	85208113	91881877	+	#7F00FF	6559873	6673764	38028	-3188005	38028	6597901	false
	HGL 14	193042308	193093801	88638563	88723035	+	#7F00FF	51493	84472	-3188005		-3188005	-3136512	true
	HGL 22	196293297	196443530	40603953	40768188	-	#EC83BA	150233	164235	15384490	18408	15384490	15534723	false
	HGL 22	196461938	196521429	40025328	40117074	-	#EC83BA	59491	91746	18408		18408	77899	false
	HGL 5	196814789	202583274	92830201	98918447	+	#093456	5768485	6088246		2285			
	HGL 5	202585559	212890697	98979415	109903081	+	#093456	10305138	10923666	2285	6038	2285	10307423	false
	HGL 5	212896735	212973647	109903239	109994552	-	#093456	76912	91313	6038	12072	6038	82950	false
	HGL 5	212985719	215196011	109997087	112343888	+	#093456	2210292	2346801	12072	2703	12072	2222364	false
	HGL 5	215198714	216755282	110550231	112294441	+	#093456	1556568	1744210	2703	8839	2703	1559271	false
	HGL 5	216764121	217305240	111647869	112273692	-	#093456	541119	625823	8839	29860	8839	549958	false
	HGL 5	217335100	218035633	110817611	111583988	-	#093456	700533	766377	29860	54416	29860	730393	false
	HGL 5	218090049	218500295	110290994	110796027	-	#093456	410246	505033	54416	9542	54416	464662	false
	HGL 5	218509837	218575590	110550231	110641165	-	#093456	65753	90934	9542	15449	9542	75295	false
	HGL 5	218591039	226882127	112286347	120503927	+	#093456	8291088	8217580	15449	10	15449	8306537	false
	HGL 5	226882137	233464054	120580240	127447574	+	#093456	6581917	6867334	10		10	6581927	false
**CPOR 10**	HGL 12	44221	1804106	90058565	91866560	+	#F33071	1759885	1807995		96116			
	HGL 12	1900222	1959124	91873114	91924950	+	#F33071	58902	51836	96116	99887	96116	155018	false
	HGL 12	2059011	7431761	91924950	97751209	+	#F33071	5372750	5826259	99887	78571	99887	5472637	false
	HGL 12	7510332	9838731	97774224	100074019	+	#F33071	2328399	2299795	78571		78571	2406970	false
	HGL 17	10076120	10540075	59665548	60190451	-	#82C90F	463955	524903		369			
	HGL 17	10540444	12013215	57876630	59599237	-	#82C90F	1472771	1722607	369	159043	369	1473140	false
	HGL 17	12172258	12542716	57369750	57876406	-	#82C90F	370458	506656	159043	16041	159043	529501	false
	HGL 17	12558757	15301159	54394340	57315986	-	#82C90F	2742402	2921646	16041	-34700	16041	2758443	false
	HGL 17	15266459	15642792	54092104	54393956	-	#82C90F	376333	301852	-34700		-34700	341633	false
	HGL 10	16063387	16201943	70009795	70097658	-	#02BCB7	138556	87863	5987719	58958	5987719	6126275	false

	HGL 10	16260901	17173804	69139436	70001299	-	#02BCB7	912903	861863	58958	70883	58958	971861	false
	HGL 10	17244687	18011603	68379591	69138559	-	#02BCB7	766916	758968	70883	168818	70883	837799	false
	HGL 10	18180421	19491953	67022458	68373641	-	#02BCB7	1311532	1351183	168818	129523	168818	1480350	false
	HGL 10	19621476	20156170	62698381	63282491	-	#02BCB7	534694	584110	129523	-19350	129523	664217	false
	HGL 10	20136820	20239725	63613538	63703123	+	#02BCB7	102905	89585	-19350	-29181	-19350	83555	false
	HGL 10	20210544	20380849	62515452	62693976	-	#02BCB7	170305	178524	-29181	-23886	-29181	141124	false
	HGL 10	20356963	21514135	65634921	66925729	+	#02BCB7	1157172	1290808	-23886	75261	-23886	1133286	false
	HGL 10	21589396	21657744	63350279	63414052	-	#02BCB7	68348	63773	75261	-8616	75261	143609	false
	HGL 10	21649128	22971534	63705902	65214477	-	#02BCB7	1322406	1508575	-8616	12040	-8616	1313790	false
	HGL 10	22983574	25521881	59930275	62365882	-	#02BCB7	2538307	2435607	12040	204080	12040	2550347	false
	HGL 10	25725961	25814812	59869124	59943471	-	#02BCB7	88851	74347	204080	814	204080	292931	false
	HGL 10	25815626	29046445	55671361	59712138	-	#02BCB7	3230819	4040777	814	70120	814	3231633	false
	HGL 10	29116565	30386413	54427976	55706819	-	#02BCB7	1269848	1278843	70120	52692	70120	1339968	false
	HGL 10	30439105	30506269	60231403	60335608	+	#02BCB7	67164	104205	52692	51976	52692	119856	false
	HGL 10	30558245	33997020	50681230	54427879	-	#02BCB7	3438775	3746649	51976	128947	51976	3490751	false
	HGL 10	34125967	38906119	45650563	50803483	-	#02BCB7	4780152	5152920	128947	64108	128947	4909099	false
	HGL 10	38970227	39056295	60231403	60335608	-	#02BCB7	86068	104205	64108	97683	64108	150176	false
	HGL 10	39153978	40195754	44503412	45631618	-	#02BCB7	1041776	1128206	97683	31854	97683	1139459	false
	HGL 10	40227608	46644371	37226929	44447715	-	#02BCB7	6416763	7220786	31854	115330	31854	6448617	false
	HGL 10	46759701	47493901	36321225	37137613	-	#02BCB7	734200	816388	115330	60318	115330	849530	false
	HGL 10	47554219	51164439	32268965	36320948	-	#02BCB7	3610220	4051983	60318	15	60318	3670538	false
	HGL 10	51164454	51255251	32177977	32264596	+	#02BCB7	90797	86619	15	0	15	90812	false
	HGL 10	51255251	52956444	30235318	32163890	-	#02BCB7	1701193	1928572	0	-22400	0	1701193	false
	HGL 10	52934044	54393082	28708168	30229136	-	#02BCB7	1459038	1520968	-22400	-14065	-22400	1436638	false
	HGL 10	54379017	65372588	16810398	28568033	-	#02BCB7	10993571	11757635	-14065	1072	-14065	10979506	false
	HGL 10	65373660	65478015	17618142	17740088	+	#02BCB7	104355	121946	1072	56150	1072	105427	false
	HGL 10	65534165	76302868	6337099	17618141	-	#02BCB7	10768703	11281042	56150	-50542	56150	10824853	false
	HGL 10	76252326	82407688	25004	6336979	-	#02BCB7	6155362	6311975	-50542		-50542	6104820	false
**CPOR 11**	HGL 29	114270	262605	2645258	2699178	+	#F400E1	148335	53920		121959			
	HGL 29	384564	710201	4186556	4503802	+	#F400E1	325637	317246	121959	608360	121959	447596	false
	HGL 29	1318561	1374626	14680321	14744336	-	#F400E1	56065	64015	608360	923135	608360	664425	false
	HGL 29	2297761	3363562	4520521	5731086	+	#F400E1	1065801	1210565	923135	73436	923135	1988936	false

	HGL 29	3436998	3653587	5736409	5938749	+	#F400E1	216589	202340	73436	29163	73436	290025	false
	HGL 29	3682750	3878203	6202648	6468205	-	#F400E1	195453	265557	29163	108900	29163	224616	false
	HGL 29	3987103	4174249	5985922	6163474	-	#F400E1	187146	177552	108900	119	108900	296046	false
	HGL 29	4174368	4465771	6515405	6841032	+	#F400E1	291403	325627	119	-19472	119	291522	false
	HGL 29	4446299	5595974	6840775	8380753	+	#F400E1	1149675	1539978	-19472	419	-19472	1130203	false
	HGL 29	5596393	5657261	8396121	8491140	-	#F400E1	60868	95019	419	92	419	61287	false
	HGL 29	5657353	7245250	8513732	10634129	+	#F400E1	1587897	2120397	92	29128	92	1587989	false
	HGL 29	7274378	7389349	10799128	10871866	+	#F400E1	114971	72738	29128	-30491	29128	144099	false
	HGL 29	7358858	7884794	10965464	11666539	+	#F400E1	525936	701075	-30491	879529	-30491	495445	false
	HGL 9	8004985	8065664	32994022	33058223	+	#036A9C	60679	64201	6630359	598690	6630359	6691038	false
	HGL 22	8197816	8267746	11132335	11340020	-	#EC83BA	69930	207685		207730			
	HGL 3	8214511	8267746	44457520	44512605	+	#C3A652	53235	55085		207730			
	HGL 3	8475476	8532284	42773618	42827756	+	#C3A652	56808	54138	207730	79991	207730	264538	false
	HGL 22	8475476	8604048	11189626	11260033	+	#EC83BA	128572	70407	207730	8227	207730	336302	false
	HGL 22	8612275	8664354	10796244	10858149	+	#EC83BA	52079	61905	8227	0	8227	60306	false
	HGL 3	8612275	8664354	41888314	41946551	-	#C3A652	52079	58237	79991	6787151	79991	132070	false
	HGL 22	8664354	8741926	10519075	10662421	-	#EC83BA	77572	143346	0	5152842	0	77572	false
	HGL 9	8664354	8741926	24309157	24424668	+	#036A9C	77572	115511	598690	4823684	598690	676262	false
	HGL 29	8764323	9801549	11934301	13420173	+	#F400E1	1037226	1485872	879529	885768	879529	1916755	false
	HGL 29	10687317	10936944	2811096	3103528	+	#F400E1	249627	292432	885768	8584	885768	1135395	false
	HGL 29	10945528	11311619	3020885	3298766	+	#F400E1	366091	277881	8584	114082	8584	374675	false
	HGL 29	11425701	11499558	2745434	2798969	+	#F400E1	73857	53535	114082	616851	114082	187939	false
	HGL 29	12116409	12429429	3916547	4181084	-	#F400E1	313020	264537	616851	38837	616851	929871	false
	HGL 29	12468266	13219576	3300578	3928688	-	#F400E1	751310	628110	38837	346034	38837	790147	false
	HGL 29	13565610	13643723	14102771	14153762	-	#F400E1	78113	50991	346034	11444355	346034	424147	false
	HGL 9	13565610	13643769	28777596	28832882	-	#036A9C	78159	55286	4823684	256466	4823684	4901843	false
	HGL 22	13894768	13953587	10931084	11014882	-	#EC83BA	58819	83798	5152842	163612	5152842	5211661	false
	HGL 9	13900235	13954391	20205657	20289357	-	#036A9C	54156	83700	256466	452441	256466	310622	false
	HGL 22	14117199	14229478	11208209	11337672	+	#EC83BA	112279	129463	163612	25900	163612	275891	false
	HGL 22	14255378	14333673	11233171	11286999	+	#EC83BA	78295	53828	25900	73159	25900	104195	false
	HGL 22	14406832	14486606	10519075	10650523	-	#EC83BA	79774	131448	73159	103484	73159	152933	false
	HGL 9	14406832	14486606	24331716	24424668	+	#036A9C	79774	92952	452441	67938	452441	532215	false

	HGL 9	14554544	14686234	24324644	24463836	-	#036A9C	131690	139192	67938	222844	67938	199628	false
	HGL 22	14590090	14683646	10520145	10657350	+	#EC83BA	93556	137205	103484	215882	103484	197040	false
	HGL 22	14899528	14953601	10971527	11051660	-	#EC83BA	54073	80133	215882	480824	215882	269955	false
	HGL 9	14909078	14961849	20211111	20289357	-	#036A9C	52771	78246	222844	9564177	222844	275615	false
	HGL 22	15434425	15730299	9514235	9807192	-	#EC83BA	295874	292957	480824	166812	480824	776698	false
	HGL 3	15451505	15630367	43665149	43911121	+	#C3A652	178862	245972	6787151	-170904	6787151	6966013	false
	HGL 3	15459463	15730299	45432693	45698227	-	#C3A652	270836	265534	-170904	7247162	-170904	99932	false
	HGL 22	15897111	16082197	2975071	3134812	+	#EC83BA	185086	159741	166812	71878	166812	351898	false
	HGL 22	16154075	16425846	3135357	3418118	+	#EC83BA	271771	282761	71878	752174	71878	343649	false
	HGL 22	17178020	18159100	3497424	4351430	+	#EC83BA	981080	854006	752174	79320	752174	1733254	false
	HGL 22	18238420	18382435	4335918	4411309	+	#EC83BA	144015	75391	79320	30507	79320	223335	false
	HGL 22	18412942	18490234	4420047	4477434	-	#EC83BA	77292	57387	30507	-24132	30507	107799	false
	HGL 22	18466102	18530661	4789134	4866275	+	#EC83BA	64559	77141	-24132	1687	-24132	40427	false
	HGL 22	18532348	18583844	8591205	8684242	+	#EC83BA	51496	93037	1687	29959	1687	53183	false
	HGL 22	18613803	18678327	4631840	4697603	-	#EC83BA	64524	65763	29959	-6069	29959	94483	false
	HGL 22	18672258	18773105	4426561	4631840	+	#EC83BA	100847	205279	-6069	-9657	-6069	94778	false
	HGL 22	18763448	18902023	4990677	5104261	+	#EC83BA	138575	113584	-9657	71861	-9657	128918	false
	HGL 22	18973884	19321166	5059516	5407661	+	#EC83BA	347282	348145	71861	151770	71861	419143	false
	HGL 22	19472936	19755774	5446661	5666496	+	#EC83BA	282838	219835	151770	129139	151770	434608	false
	HGL 22	19884913	20015269	6948778	7045438	-	#EC83BA	130356	96660	129139	354132	129139	259495	false
	HGL 22	20369401	21319135	5701191	6449837	+	#EC83BA	949734	748646	354132	-4445	354132	1303866	false
	HGL 22	21314690	21407630	6746665	6813646	-	#EC83BA	92940	66981	-4445	-92924	-4445	88495	false
	HGL 22	21314706	21408849	6583633	6680886	-	#EC83BA	94143	97253	-92924	-75889	-92924	1219	false
	HGL 22	21332960	21467931	6364704	6527336	-	#EC83BA	134971	162632	-75889	-57964	-75889	59082	false
	HGL 22	21409967	21633040	6803846	6899349	+	#EC83BA	223073	95503	-57964	-156280	-57964	165109	false
	HGL 22	21476760	21871118	6921214	7239330	+	#EC83BA	394358	318116	-156280	50786	-156280	238078	false
	HGL 22	21921904	22931292	7223220	8127070	+	#EC83BA	1009388	903850	50786	46169	50786	1060174	false
	HGL 3	22977461	23099431	44949487	45039275	+	#C3A652	121970	89788	7247162	447164	7247162	7369132	false
	HGL 22	22977461	23137247	8362195	8604242	-	#EC83BA	159786	242047	46169	46696	46169	205955	false
	HGL 22	23183943	23370260	8091613	8192545	-	#EC83BA	186317	100932	46696	17046	46696	233013	false
	HGL 22	23387306	23546476	7965952	8136558	-	#EC83BA	159170	170606	17046	119	17046	176216	false
	HGL 3	23546595	23661328	44796693	44895904	-	#C3A652	114733	99211	447164	864698	447164	561897	false

	HGL 22	23546595	23672977	8678576	8855946	+	#EC83BA	126382	177370	119	90482	119	126501	false
	HGL 22	23763459	23890666	8046612	8155638	+	#EC83BA	127207	109026	90482	29243	90482	217689	false
	HGL 22	23919909	23976719	8362195	8467208	+	#EC83BA	56810	105013	29243	552375	29243	86053	false
	HGL 9	24526026	24617343	24145836	24300055	-	#036A9C	91317	154219	9564177	-61201	9564177	9655494	false
	HGL 3	24526026	24631947	41911768	42047876	-	#C3A652	105921	136108	864698	3519010	864698	970619	false
	HGL 22	24529094	24584741	10671324	10725982	+	#EC83BA	55647	54658	552375	48317	552375	608022	false
	HGL 9	24556142	24631947	20037811	20133642	+	#036A9C	75805	95831	-61201	31659080	-61201	14604	false
	HGL 22	24633058	24684321	11283528	11342091	+	#EC83BA	51263	58563	48317	7070	48317	99580	false
	HGL 22	24691391	24749981	11157055	11337314	-	#EC83BA	58590	180259	7070	13054	7070	65660	false
	HGL 22	24763035	24820776	8601631	8743189	+	#EC83BA	57741	141558	13054	3336745	13054	70795	false
	HGL 29	25088078	25682261	395485	1077275	-	#F400E1	594183	681790	11444355	215275	11444355	12038538	false
	HGL 29	25897536	25961296	324920	378909	-	#F400E1	63760	53989	215275	176768	215275	279035	false
	HGL 29	26138064	26212604	1091918	1169802	+	#F400E1	74540	77884	176768	871311	176768	251308	false
	HGL 29	27083915	28041137	1307797	2384959	-	#F400E1	957222	1077162	871311		871311	1828533	false
	HGL 22	28157521	28214433	10671324	10725973	+	#EC83BA	56912	54649	3336745	2457	3336745	3393657	false
	HGL 22	28216890	28269347	10807922	10859268	+	#EC83BA	52457	51346	2457	96332	2457	54914	false
	HGL 22	28365679	28529825	11164229	11403031	+	#EC83BA	164146	238802	96332	80349	96332	260478	false
	HGL 22	28610174	37429095	11459656	20287544	+	#EC83BA	8818921	8827888	80349	624	80349	8899270	false
	HGL 22	37429719	37484546	20293478	20358316	-	#EC83BA	54827	64838	624	48713	624	55451	false
	HGL 22	37533259	40742004	20424644	23918501	+	#EC83BA	3208745	3493857	48713	10637	48713	3257458	false
	HGL 22	40752641	40856771	23926021	24029014	-	#EC83BA	104130	102993	10637	-48243	10637	114767	false
	HGL 22	40808528	41712554	24041846	24979579	+	#EC83BA	904026	937733	-48243	41350	-48243	855783	false
	HGL 22	41753904	43246193	25079995	26449177	+	#EC83BA	1492289	1369182	41350	267509	41350	1533639	false
	HGL 22	43513702	44591305	26477998	27630047	+	#EC83BA	1077603	1152049	267509	87863	267509	1345112	false
	HGL 22	44679168	48750376	27508023	31800173	+	#EC83BA	4071208	4292150	87863	7914	87863	4159071	false
	HGL 22	48758290	50058044	31870883	33249489	+	#EC83BA	1299754	1378606	7914	53160	7914	1307668	false
	HGL 22	50111204	51833106	33295304	35216687	+	#EC83BA	1721902	1921383	53160	90271	53160	1775062	false
	HGL 22	51923377	52160032	35235562	35600079	+	#EC83BA	236655	364517	90271	62509	90271	326926	false
	HGL 22	52222541	52305010	54256683	54370929	+	#EC83BA	82469	114246	62509	52670	62509	144978	false
	HGL 22	52357680	52576619	54430353	54672085	-	#EC83BA	218939	241732	52670		52670	271609	false
	HGL 24	53086454	53202447	47564994	47652328	-	#FF0000	115993	87334		86947			
	HGL 24	53289394	54724625	45811003	47546816	+	#FF0000	1435231	1735813	86947	167739	86947	1522178	false

	HGL 24	54892364	54990442	45583197	45705859	-	#FF0000	98078	122662	167739	13739	167739	265817	false
	HGL 24	55004181	55157149	45334909	45527048	-	#FF0000	152968	192139	13739	2768	13739	166707	false
	HGL 24	55159917	55888892	44195363	45276126	-	#FF0000	728975	1080763	2768	20000	2768	731743	false
	HGL 24	55908892	56362533	43585302	44144085	-	#FF0000	453641	558783	20000	40498	20000	473641	false
	HGL 24	56403031	57845444	47675815	49654116	+	#FF0000	1442413	1978301	40498		40498	1482911	false
	HGL 6	57927822	68288498	18375945	29298700	+	#980157	10360676	10922755		1			
	HGL 6	68288499	68430248	29268101	29346013	-	#980157	141749	77912	1	14221	1	141750	false
	HGL 6	68444469	72247757	29346017	33304229	+	#980157	3803288	3958212	14221		14221	3817509	false
**CPOR 12**	HGL 26	9279	2019349	19789350	21824042	-	#64E09A	2010070	2034692		4381541			
	HGL 22	2221341	4053191	35627141	37329254	+	#EC83BA	1831850	1702113		64354			
	HGL 22	4117545	6391459	37392922	39487995	+	#EC83BA	2273914	2095073	64354		64354	2338268	false
	HGL 4	7377223	11559706	84229620	88486472	-	#5654E8	4182483	4256852		57058			
	HGL 4	11616764	12196430	83712694	84227996	-	#5654E8	579666	515302	57058	7	57058	636724	false
	HGL 4	12196437	12472327	83443676	83712694	+	#5654E8	275890	269018	7	6	7	275897	false
	HGL 4	12472333	12947118	82968114	83443676	-	#5654E8	474785	475562	6	6390	6	474791	false
	HGL 4	12953508	13102427	82829256	82949946	+	#5654E8	148919	120690	6390	0	6390	155309	false
	HGL 4	13102427	24390191	70863440	82815515	-	#5654E8	11287764	11952075	0	77391	0	11287764	false
	HGL 4	24467582	26619652	99860421	102034657	-	#5654E8	2152070	2174236	77391	18027	77391	2229461	false
	HGL 4	26637679	28028100	98424767	99807948	-	#5654E8	1390421	1383181	18027	123134	18027	1408448	false
	HGL 4	28151234	28241729	98356623	98420297	-	#5654E8	90495	63674	123134	-88523	123134	213629	false
	HGL 4	28153206	28244506	98422304	98530822	-	#5654E8	91300	108518	-88523	69594	-88523	2777	false
	HGL 4	28314100	38089967	88527092	98356253	-	#5654E8	9775867	9829161	69594		69594	9845461	false
	HGL 17	38252720	57171474	32816135	53624308	-	#82C90F	18918754	20808173		655			
	HGL 17	57172129	57786437	32113908	32812456	+	#82C90F	614308	698548	655	0	655	614963	false
	HGL 17	57786437	59180041	30578476	32107750	-	#82C90F	1393604	1529274	0	482003	0	1393604	false
	HGL 17	59662044	65538031	23970973	30576371	-	#82C90F	5875987	6605398	482003	364948	482003	6357990	false
	HGL 17	65902979	70750575	18702739	23970570	-	#82C90F	4847596	5267831	364948	73636	364948	5212544	false
	HGL 17	70824211	77478949	11583481	18701491	-	#82C90F	6654738	7118010	73636	94	73636	6728374	false
	HGL 17	77479043	77559345	11500284	11583481	+	#82C90F	80302	83197	94	37	94	80396	false
	HGL 17	77559382	88218628	86334	11497807	-	#82C90F	10659246	11411473	37		37	10659283	false
	HGL 2	88496189	88569438	126288542	126409867	-	#1CB707	73249	121325		12810			
	HGL 2	88582248	88655768	125508473	125619240	-	#1CB707	73520	110767	12810		12810	86330	false

**CPOR 13**	HGL 6	8821	2000619	16417824	18375906	-	#980157	1991798	1958082		14150			
	HGL 6	2014769	14975432	3021566	16350474	-	#980157	12960663	13328908	14150	32107	14150	12974813	false
	HGL 6	15007539	17742328	53270	2962397	-	#980157	2734789	2909127	32107		32107	2766896	false
	HGL 1	18086471	18864725	132338689	133260711	-	#49F643	778254	922022		107			
	HGL 1	18864832	18932678	132275051	132338258	+	#49F643	67846	63207	107	334	107	67953	false
	HGL 1	18933012	20154393	130960858	132269036	-	#49F643	1221381	1308178	334	1670	334	1221715	false
	HGL 1	20156063	20279173	133090043	133250660	-	#49F643	123110	160617	1670	12490	1670	124780	false
	HGL 1	20291663	20883696	132340362	133037842	-	#49F643	592033	697480	12490	286	12490	604523	false
	HGL 1	20883982	20970181	132270095	132338258	+	#49F643	86199	68163	286	372	286	86485	false
	HGL 1	20970553	22122694	130958666	132269036	-	#49F643	1152141	1310370	372	4136	372	1152513	false
	HGL 1	22126830	23241904	131008503	132269036	+	#49F643	1115074	1260533	4136	382	4136	1119210	false
	HGL 1	23242286	23306506	132269287	132338258	-	#49F643	64220	68971	382	633	382	64602	false
	HGL 1	23307139	24027349	132338689	133250095	+	#49F643	720210	911406	633	658	633	720843	false
	HGL 1	24028007	24768505	130960858	131768072	+	#49F643	740498	807214	658	3402	658	741156	false
	HGL 1	24771907	24973478	131846516	132090147	+	#49F643	201571	243631	3402	62017	3402	204973	false
	HGL 1	25035495	25145609	132646116	132822571	+	#49F643	110114	176455	62017	173217	62017	172131	false
	HGL 1	25318826	25383402	130874274	130933790	-	#49F643	64576	59516	173217	28017	173217	237793	false
	HGL 1	25411419	25538177	130874274	131008503	-	#49F643	126758	134229	28017	29276	28017	154775	false
	HGL 1	25567453	30307573	125800363	131008503	-	#49F643	4740120	5208140	29276	106863	29276	4769396	false
	HGL 1	30414436	35073481	120804925	125798074	-	#49F643	4659045	4993149	106863	131895	106863	4765908	false
	HGL 1	35205376	35732049	120221149	120804518	-	#49F643	526673	583369	131895	37374	131895	658568	false
	HGL 1	35769423	38257792	113489996	116268649	+	#49F643	2488369	2778653	37374	40125	37374	2525743	false
	HGL 1	38297917	41548665	116328166	119888047	+	#49F643	3250748	3559881	40125	12860	40125	3290873	false
	HGL 1	41561525	41826617	113212489	113470073	+	#49F643	265092	257584	12860	14118	12860	277952	false
	HGL 1	41840735	41950787	98494479	98585883	+	#49F643	110052	91404	14118	-89299	14118	124170	false
	HGL 1	41861488	54541639	98760098	113211712	-	#49F643	12680151	14451614	-89299	4418	-89299	12590852	false
	HGL 1	54546057	59370030	92680375	98469302	-	#49F643	4823973	5788927	4418	-21782	4418	4828391	false
	HGL 3	57024573	57178521	40987942	41154479	+	#C3A652	153948	166537		260766			
	HGL 19	57024766	57112769	14842042	14920546	+	#8D3B0C	88003	78504		5179			
	HGL 19	57117948	57176031	14932034	14991819	-	#8D3B0C	58083	59785	5179		5179	63262	false
	HGL 3	57439287	57497595	40933260	40987942	+	#C3A652	58308	54682	260766		260766	319074	false
	HGL 1	59348248	59517129	92395143	92644066	-	#49F643	168881	248923	-21782	87493	-21782	147099	false

	HGL 1	59604622	59896001	92030558	92394743	-	#49F643	291379	364185	87493	97718	87493	378872	false
	HGL 1	59993719	62256325	89263181	92030450	-	#49F643	2262606	2767269	97718	1107	97718	2360324	false
	HGL 1	62257432	62455478	89028464	89256094	+	#49F643	198046	227630	1107	27	1107	199153	false
	HGL 1	62455505	63256408	87939793	89028452	-	#49F643	800903	1088659	27	659	27	800930	false
	HGL 1	63257067	63307823	87873341	87927678	+	#49F643	50756	54337	659	1742	659	51415	false
	HGL 1	63309565	63846408	87146832	87825625	-	#49F643	536843	678793	1742	254	1742	538585	false
	HGL 1	63846662	64478220	86410747	87089998	-	#49F643	631558	679251	254	4505	254	631812	false
	HGL 1	64482725	64813499	86000603	86351507	-	#49F643	330774	350904	4505	105240	4505	335279	false
	HGL 1	64918739	64969609	133285239	133337723	-	#49F643	50870	52484	105240		105240	156110	false
	HGL 26	65085019	65924859	16515	935755	+	#64E09A	839840	919240		92331			
	HGL 26	66017190	66098634	7563917	7645263	-	#64E09A	81444	81346	92331	4	92331	173775	false
	HGL 26	66098638	68830010	935755	3521132	+	#64E09A	2731372	2585377	4	405	4	2731376	false
	HGL 26	68830415	76037444	3576335	10289923	+	#64E09A	7207029	6713588	405	12246	405	7207434	false
	HGL 26	76049690	76100318	10348540	10427360	+	#64E09A	50628	78820	12246		12246	62874	false
	HGL 22	76195243	76321015	41890288	42038443	+	#EC83BA	125772	148155		610219			
	HGL 22	76931234	77056823	8130532	8215634	+	#EC83BA	125589	85102	610219		610219	735808	false
**CPOR 14**	HGL 2	111724	668899	82036557	82581128	+	#1CB707	557175	544571		5443			
	HGL 2	674342	2180896	80304338	81912042	-	#1CB707	1506554	1607704	5443	307367	5443	1511997	false
	HGL 2	2488263	6832682	75448378	80061088	-	#1CB707	4344419	4612710	307367	34037	307367	4651786	false
	HGL 2	6866719	6934598	75750824	75833927	+	#1CB707	67879	83103	34037	19154	34037	101916	false
	HGL 2	6953752	7048681	45443799	45593278	+	#1CB707	94929	149479	19154	41119	19154	114083	false
	HGL 2	7089800	7153872	48068217	48120891	-	#1CB707	64072	52674	41119	103331	41119	105191	false
	HGL 2	7257203	7883235	74841434	75465397	-	#1CB707	626032	623963	103331	13966	103331	729363	false
	HGL 2	7897201	11281830	70855999	74548275	-	#1CB707	3384629	3692276	13966	0	13966	3398595	false
	HGL 2	11281830	11399963	70681515	70830308	+	#1CB707	118133	148793	0	15	0	118133	false
	HGL 2	11399978	17125799	64723239	70674580	-	#1CB707	5725821	5951341	15	15672	15	5725836	false
	HGL 2	17141471	19617361	61465211	64553216	-	#1CB707	2475890	3088005	15672	85347	15672	2491562	false
	HGL 2	19702708	28459335	51339705	61419026	-	#1CB707	8756627	10079321	85347	-7594899	85347	8841974	false
	HGL 2	20864436	21059912	60144978	60379638	-	#1CB707	195476	234660	-7594899	7399532	-7594899	-7399423	true
	HGL 2	28459444	28569265	51367267	51482299	-	#1CB707	109821	115032	7399532	55658	7399532	7509353	false
	HGL 2	28624923	30583746	48815721	51200521	-	#1CB707	1958823	2384800	55658	-91848	55658	2014481	false
	HGL 2	30491898	30611961	48748286	48823074	-	#1CB707	120063	74788	-91848	-101843	-91848	28215	false

	HGL 2	30510118	30583746	48872920	48926731	+	#1CB707	73628	53811	-101843	-12565	-101843	-28215	true
	HGL 2	30571181	30702460	48664810	48748259	-	#1CB707	131279	83449	-12565	-5097	-12565	118714	false
	HGL 2	30697363	30872753	48224706	48356802	-	#1CB707	175390	132096	-5097	0	-5097	170293	false
	HGL 2	30872753	30941000	45322561	45422699	+	#1CB707	68247	100138	0	22292	0	68247	false
	HGL 2	30963292	31018185	45360825	45452416	-	#1CB707	54893	91591	22292	28551	22292	77185	false
	HGL 2	31046736	31166569	45349545	45546025	-	#1CB707	119833	196480	28551	16177	28551	148384	false
	HGL 2	31182746	32305137	46472563	47812087	-	#1CB707	1122391	1339524	16177	5	16177	1138568	false
	HGL 2	32305142	33019823	45869148	46666329	+	#1CB707	714681	797181	5	1175	5	714686	false
	HGL 2	33020998	35212467	44055829	46669664	+	#1CB707	2191469	2613835	1175	416	1175	2192644	false
	HGL 2	35212883	36291552	44037727	45226500	+	#1CB707	1078669	1188773	416	1691	416	1079085	false
	HGL 2	36293243	36679546	45306452	45869148	+	#1CB707	386303	562696	1691	0	1691	387994	false
	HGL 2	36679546	37450647	45530581	46472562	-	#1CB707	771101	941981	0	1990	0	771101	false
	HGL 2	37452637	49307973	31392798	45445325	-	#1CB707	11855336	14052527	1990	7575	1990	11857326	false
	HGL 2	49315548	49393734	31293572	31392798	+	#1CB707	78186	99226	7575	8	7575	85761	false
	HGL 2	49393742	55384922	24502572	31293456	-	#1CB707	5991180	6790884	8	0	8	5991188	false
	HGL 2	55384922	55618366	24202014	24502512	+	#1CB707	233444	300498	0	69	0	233444	false
	HGL 2	55618435	59499138	19858828	24202014	-	#1CB707	3880703	4343186	69	105	69	3880772	false
	HGL 2	59499243	59562638	45342592	45444850	+	#1CB707	63395	102258	105	643	105	63500	false
	HGL 2	59563281	60095848	19135998	19858828	-	#1CB707	532567	722830	643	469	643	533210	false
	HGL 2	60096317	60212999	18935952	19061951	-	#1CB707	116682	125999	469	2947	469	117151	false
	HGL 2	60215946	60958482	18497486	19346957	-	#1CB707	742536	849471	2947	111	2947	745483	false
	HGL 2	60958593	61032968	18266192	18360589	-	#1CB707	74375	94397	111	0	111	74486	false
	HGL 2	61032968	61125608	21467232	21611310	-	#1CB707	92640	144078	0	172	0	92640	false
	HGL 2	61125780	61200158	24105535	24164884	+	#1CB707	74378	59349	172	58288	172	74550	false
	HGL 2	61258446	61379359	20889685	21047990	-	#1CB707	120913	158305	58288	7245	58288	179201	false
	HGL 2	61386604	61689875	20354436	20794960	-	#1CB707	303271	440524	7245	1335	7245	310516	false
	HGL 2	61691210	63416879	18266192	20279501	-	#1CB707	1725669	2013309	1335	26140	1335	1727004	false
	HGL 2	63443019	66656323	15181	3102151	+	#1CB707	3213304	3086970	26140	2203	26140	3239444	false
	HGL 2	66658526	66712183	3058243	3150846	-	#1CB707	53657	92603	2203	2176	2203	55860	false
	HGL 2	66714359	67916841	3108245	4369803	+	#1CB707	1202482	1261558	2176	11533	2176	1204658	false
	HGL 2	67928374	70921822	4379904	7448505	-	#1CB707	2993448	3068601	11533	-4598	11533	3004981	false
	HGL 2	70917224	71043397	7475039	7532858	-	#1CB707	126173	57819	-4598	-29992	-4598	121575	false

	HGL 2	71013405	73461729	7562439	10025026	+	#1CB707	2448324	2462587	-29992		-29992	2418332	false
**CPOR 15**	HGL 3	400660	693221	63398607	63623816	+	#C3A652	292561	225209		57367			
	HGL 3	750588	4370044	63631567	67358570	+	#C3A652	3619456	3727003	57367	17682	57367	3676823	false
	HGL 3	4387726	13598050	67418525	77640006	+	#C3A652	9210324	10221481	17682	10307	17682	9228006	false
	HGL 3	13608357	13779948	77780854	77989015	-	#C3A652	171591	208161	10307	2941	10307	181898	false
	HGL 3	13782889	13841200	77640626	77710805	-	#C3A652	58311	70179	2941	13001	2941	61252	false
	HGL 3	13854201	14047837	78004848	78233017	+	#C3A652	193636	228169	13001	213944	13001	206637	false
	HGL 3	14261781	19684656	78497853	84610209	+	#C3A652	5422875	6112356	213944	-70323	213944	5636819	false
	HGL 3	19614333	19803737	84627290	84802726	-	#C3A652	189404	175436	-70323	-106030	-70323	119081	false
	HGL 3	19697707	19864521	84639343	84729245	-	#C3A652	166814	89902	-106030	-49261	-106030	60784	false
	HGL 3	19815260	22694902	84809378	87741556	+	#C3A652	2879642	2932178	-49261	138350	-49261	2830381	false
	HGL 3	22833252	23051403	87754750	87945647	+	#C3A652	218151	190897	138350	54770	138350	356501	false
	HGL 3	23106173	23194518	87853308	87920297	+	#C3A652	88345	66989	54770	128484	54770	143115	false
	HGL 3	23323002	26278981	87918509	91231184	+	#C3A652	2955979	3312675	128484	222194	128484	3084463	false
	HGL 3	26501175	33381766	91231194	98890985	+	#C3A652	6880591	7659791	222194	241762	222194	7102785	false
	HGL 3	33623528	33913516	99010784	99294533	+	#C3A652	289988	283749	241762	88086	241762	531750	false
	HGL 3	34001602	37459235	108602936	112354637	-	#C3A652	3457633	3751701	88086	10742	88086	3545719	false
	HGL 3	37469977	38795624	107074459	108426329	+	#C3A652	1325647	1351870	10742	4895	10742	1336389	false
	HGL 3	38800519	40953857	104733037	107024935	-	#C3A652	2153338	2291898	4895	40996	4895	2158233	false
	HGL 3	40994853	45784425	99304578	104655135	-	#C3A652	4789572	5350557	40996	82703	40996	4830568	false
	HGL 3	45867128	46113115	126488080	126799605	-	#C3A652	245987	311525	82703	-39388	82703	328690	false
	HGL 3	46073727	49618141	122919278	126479596	-	#C3A652	3544414	3560318	-39388	93	-39388	3505026	false
	HGL 3	49618234	49980292	122486550	122865166	-	#C3A652	362058	378616	93	60609	93	362151	false
	HGL 3	50040901	50654251	121870445	122485594	-	#C3A652	613350	615149	60609	-48627	60609	673959	false
	HGL 3	50605624	50833030	121760082	121902250	+	#C3A652	227406	142168	-48627	-127516	-48627	178779	false
	HGL 3	50705514	50847816	57452503	57532762	+	#C3A652	142302	80259	-127516	130588	-127516	14786	false
	HGL 3	50978404	51107453	126650253	126799605	+	#C3A652	129049	149352	130588	100585	130588	259637	false
	HGL 3	51208038	51325176	121802824	121904214	-	#C3A652	117138	101390	100585	-940	100585	217723	false
	HGL 3	51324236	51425086	57452249	57537237	+	#C3A652	100850	84988	-940	-39106	-940	99910	false
	HGL 3	51385980	60319526	112359335	121761131	-	#C3A652	8933546	9401796	-39106	-8714655	-39106	8894440	false
	HGL 3	51604871	51655925	57185400	57236391	+	#C3A652	51054	50991	-8714655	310554	-8714655	-8663601	true
	HGL 3	51966479	52116547	57297520	57451635	+	#C3A652	150068	154115	310554	190996	310554	460622	false

	HGL 3	52307543	52445826	95618506	95798812	-	#C3A652	138283	180306	190996	7942648	190996	329279	false
**CPOR 16**	HGL 12	0	819589	897624	1715254	+	#F33071	819589	817630		264477			
	HGL 12	1084066	4969938	1715306	5655738	+	#F33071	3885872	3940432	264477	-81425	264477	4150349	false
	HGL 12	4888513	4944551	5574663	5640866	+	#F33071	56038	66203	-81425	71458	-81425	-25387	true
	HGL 12	5016009	5180557	5581795	5655738	+	#F33071	164548	73943	71458	-112016	71458	236006	false
	HGL 12	5068541	5222354	5649308	5741966	+	#F33071	153813	92658	-112016	-100936	-112016	41797	false
	HGL 12	5121418	5205229	5581803	5637478	+	#F33071	83811	55675	-100936	-31863	-100936	-17125	true
	HGL 12	5173366	5286147	5578667	5649367	+	#F33071	112781	70700	-31863	-36486	-31863	80918	false
	HGL 12	5249661	6974389	5708718	7418128	+	#F33071	1724728	1709410	-36486	0	-36486	1688242	false
	HGL 12	6974389	7055684	7472823	7558375	-	#F33071	81295	85552	0	12165	0	81295	false
	HGL 12	7067849	10019263	7589421	10470074	+	#F33071	2951414	2880653	12165	129643	12165	2963579	false
	HGL 12	10148906	10270572	10479012	10619847	+	#F33071	121666	140835	129643	89	129643	251309	false
	HGL 12	10270661	14836922	10506845	15272556	+	#F33071	4566261	4765711	89	1000	89	4566350	false
	HGL 12	14837922	15717188	31887	902098	-	#F33071	879266	870211	1000		1000	880266	false
	HGL 5	15879413	16381924	56418433	56927790	-	#093456	502511	509357	5761923	-23364	5761923	6264434	false
	HGL 5	16358560	16482824	56418433	56575347	+	#093456	124264	156914	-23364	18966	-23364	100900	false
	HGL 5	16501790	16566913	56331455	56429859	+	#093456	65123	98404	18966	23610	18966	84089	false
	HGL 5	16590523	16919291	55999671	56339424	-	#093456	328768	339753	23610	25825	23610	352378	false
	HGL 5	16945116	17177797	43484719	43756470	+	#093456	232681	271751	25825	98553	25825	258506	false
	HGL 5	17276350	20673278	43791542	47618543	+	#093456	3396928	3827001	98553	62227	98553	3495481	false
	HGL 5	20735505	22286915	47618575	49408760	+	#093456	1551410	1790185	62227	105214	62227	1613637	false
	HGL 5	22392129	23069087	49419785	50279310	+	#093456	676958	859525	105214	7908	105214	782172	false
	HGL 5	23076995	23228214	50255253	50435562	-	#093456	151219	180309	7908	60521	7908	159127	false
	HGL 5	23288735	27050105	50255253	54999490	+	#093456	3761370	4744237	60521	12010	60521	3821891	false
	HGL 5	27062115	27172404	55050628	55117723	+	#093456	110289	67095	12010	20834	12010	122299	false
	HGL 5	27193238	28091983	55050628	55965592	+	#093456	898745	914964	20834	848060	20834	919579	false
	HGL 5	28940043	29114737	57145438	57266212	+	#093456	174694	120774	848060	125658	848060	1022754	false
	HGL 5	29240395	29343060	57170447	57270194	+	#093456	102665	99747	125658	149803	125658	228323	false
	HGL 5	29492863	29720825	57501188	57604967	+	#093456	227962	103779	149803	-163511	149803	377765	false
	HGL 5	29557314	29620973	57889352	57950118	+	#093456	63659	60766	-163511	-17901	-163511	-99852	true
	HGL 5	29603072	29657395	57111052	57165290	+	#093456	54323	54238	-17901	-37059	-17901	36422	false
	HGL 5	29620336	29720825	58303090	58362360	-	#093456	100489	59270	-37059	-40638	-37059	63430	false

	HGL 5	29680187	29785120	57145429	57270194	+	#093456	104933	124765	-40638	-63928	-40638	64295	false
	HGL 5	29721192	29905549	58316987	58406082	-	#093456	184357	89095	-63928	-59838	-63928	120429	false
	HGL 5	29845711	29905549	57145429	57196784	+	#093456	59838	51355	-59838	-59838	-59838	0	true
	HGL 5	29845711	30106722	57906550	58029916	+	#093456	261011	123366	-59838	-185784	-59838	201173	false
	HGL 5	29920938	30033126	57893158	57948049	+	#093456	112188	54891	-185784	-17528	-185784	-73596	true
	HGL 5	30015598	30096302	57426531	57483709	+	#093456	80704	57178	-17528	93740	-17528	63176	false
	HGL 5	30190042	30243275	58896488	58958887	+	#093456	53233	62399	93740	9225	93740	146973	false
	HGL 5	30252500	30336432	58560742	58678895	+	#093456	83932	118153	9225	178187	9225	93157	false
	HGL 5	30514619	30643625	58964365	59031640	+	#093456	129006	67275	178187	189130	178187	307193	false
	HGL 5	30832755	31170389	58896488	59031640	-	#093456	337634	135152	189130	-71738	189130	526764	false
	HGL 5	31098651	31193235	58774014	58887872	-	#093456	94584	113858	-71738	820922	-71738	22846	false
	HGL 5	32014157	37661983	59113047	65456817	+	#093456	5647826	6343770	820922	-5002214	820922	6468748	false
	HGL 5	32659769	32752031	59799690	59864902	-	#093456	92262	65212	-5002214	4946159	-5002214	-4909952	true
	HGL 5	37698190	45540831	65508220	74721307	+	#093456	7842641	9213087	4946159	42890	4946159	12788800	false
	HGL 5	45583721	52380258	74773390	82247296	+	#093456	6796537	7473906	42890	48672	42890	6839427	false
	HGL 5	52428930	55484301	82332498	85481808	+	#093456	3055371	3149310	48672	0	48672	3104043	false
	HGL 5	55484301	59079113	85557781	89252965	+	#093456	3594812	3695184	0	1893	0	3594812	false
	HGL 5	59081006	59145532	89253188	89314666	-	#093456	64526	61478	1893	0	1893	66419	false
	HGL 5	59145532	62008067	89314686	92125297	+	#093456	2862535	2810611	0	-4238	0	2862535	false
	HGL 5	62003829	62564633	92283808	92829925	+	#093456	560804	546117	-4238		-4238	556566	false
**CPOR 17**	HGL 22	1053	161031	42199555	42402273	+	#EC83BA	159978	202718		3916			
	HGL 22	164947	5362390	39501248	44799704	+	#EC83BA	5197443	5298456	3916	78637	3916	5201359	false
	HGL 22	5441027	9940015	44800166	49321418	+	#EC83BA	4498988	4521252	78637	224561	78637	4577625	false
	HGL 22	10164576	10299042	49406109	49548304	-	#EC83BA	134466	142195	224561	29	224561	359027	false
	HGL 22	10299071	15002797	49548304	54239412	+	#EC83BA	4703726	4691108	29		29	4703755	false
	HGL 19	15004171	23127174	7366248	14661624	-	#8D3B0C	8123003	7295376		66995			
	HGL 19	23194169	23789122	6835980	7362897	-	#8D3B0C	594953	526917	66995	13036	66995	661948	false
	HGL 19	23802158	23973634	15042150	15218713	-	#8D3B0C	171476	176563	13036	1716	13036	184512	false
	HGL 19	23975350	27311994	62344	3190487	+	#8D3B0C	3336644	3128143	1716	50	1716	3338360	false
	HGL 19	27312044	27835230	3251983	3757666	+	#8D3B0C	523186	505683	50	92170	50	523236	false
	HGL 19	27927400	28278570	3732550	4108458	+	#8D3B0C	351170	375908	92170	34223	92170	443340	false
	HGL 19	28312793	28362996	4108133	4158633	-	#8D3B0C	50203	50500	34223	910	34223	84426	false

	HGL 19	28363906	31052020	4160163	6751973	+	#8D3B0C	2688114	2591810	910	79432	910	2689024	false
	HGL 19	31131452	32014858	15218723	16138096	+	#8D3B0C	883406	919373	79432	56790	79432	962838	false
	HGL 19	32071648	36834376	16209313	21157891	+	#8D3B0C	4762728	4948578	56790	14872	56790	4819518	false
	HGL 19	36849248	39190170	21252901	23751948	+	#8D3B0C	2340922	2499047	14872	-4378	14872	2355794	false
	HGL 19	39185792	39919216	23821055	24528935	+	#8D3B0C	733424	707880	-4378	28	-4378	729046	false
	HGL 19	39919244	41456448	24685125	25964117	-	#8D3B0C	1537204	1278992	28	99024	28	1537232	false
	HGL 19	41555472	42981555	27022280	28507930	-	#8D3B0C	1426083	1485650	99024	27398	99024	1525107	false
	HGL 19	43008953	43854739	25987350	27020828	+	#8D3B0C	845786	1033478	27398	-11498	27398	873184	false
	HGL 19	43843241	50445312	28553024	35621899	+	#8D3B0C	6602071	7068875	-11498	41311	-11498	6590573	false
	HGL 19	50486623	50601416	35682154	35778200	+	#8D3B0C	114793	96046	41311	762010	41311	156104	false
	HGL 19	51363426	51438814	35837686	35980845	+	#8D3B0C	75388	143159	762010	-1178	762010	837398	false
	HGL 19	51437636	51557479	36131935	36209794	+	#8D3B0C	119843	77859	-1178	292367	-1178	118665	false
	HGL 19	51849846	61136163	36166132	45666435	+	#8D3B0C	9286317	9500303	292367	31404	292367	9578684	false
	HGL 19	61167567	61265104	45583974	45673694	+	#8D3B0C	97537	89720	31404	29707	31404	128941	false
	HGL 19	61294811	61523248	45506065	45712172	+	#8D3B0C	228437	206107	29707	-76255	29707	258144	false
	HGL 19	61446993	61541354	45577737	45673592	+	#8D3B0C	94361	95855	-76255	50456	-76255	18106	false
	HGL 19	61591810	61722993	45577737	45704720	+	#8D3B0C	131183	126983	50456	-49061	50456	181639	false
	HGL 19	61673932	61808945	45629493	45714278	+	#8D3B0C	135013	84785	-49061	-128438	-49061	85952	false
	HGL 19	61680507	63155124	45615842	47112257	+	#8D3B0C	1474617	1496415	-128438	60684	-128438	1346179	false
	HGL 19	63215808	67248731	47112274	51517366	+	#8D3B0C	4032923	4405092	60684	21787	60684	4093607	false
	HGL 19	67270518	68028917	52172127	53021586	-	#8D3B0C	758399	849459	21787	427	21787	780186	false
	HGL 19	68029344	68215354	52596081	52778375	+	#8D3B0C	186010	182294	427	11741	427	186437	false
	HGL 19	68227095	68360519	51873195	52011843	-	#8D3B0C	133424	138648	11741	58594	11741	145165	false
	HGL 19	68419113	68615475	51501177	51775180	-	#8D3B0C	196362	274003	58594	4015	58594	254956	false
	HGL 19	68619490	69201821	52298938	53021586	-	#8D3B0C	582331	722648	4015	9757	4015	586346	false
	HGL 19	69211578	69265539	52244217	52298938	+	#8D3B0C	53961	54721	9757	-12848	9757	63718	false
	HGL 19	69252691	69478240	52018352	52287380	-	#8D3B0C	225549	269028	-12848	12026	-12848	212701	false
	HGL 19	69490266	69988455	51523169	52079772	-	#8D3B0C	498189	556603	12026	2155	12026	510215	false
	HGL 19	69990610	70152791	51543522	51777910	+	#8D3B0C	162181	234388	2155	1456	2155	164336	false
	HGL 19	70154247	70884440	51840733	52559623	+	#8D3B0C	730193	718890	1456	1648	1456	731649	false
	HGL 19	70886088	72291345	52804651	54480564	+	#8D3B0C	1405257	1675913	1648	-41454	1648	1406905	false
	HGL 19	72249891	72397591	54404991	54590296	-	#8D3B0C	147700	185305	-41454	-64622	-41454	106246	false

	HGL 19	72332969	73314007	54533440	55477892	+	#8D3B0C	981038	944452	-64622		-64622	916416	false
**CPOR 18**	HGL 1	259140	631372	85505408	85929393	-	#49F643	372232	423985		300			
	HGL 1	631672	3678736	82172485	85391168	+	#49F643	3047064	3218683	300	0	300	3047364	false
	HGL 1	3678736	9077174	73193421	78851406	+	#49F643	5398438	5657985	0	205134	0	5398438	false
	HGL 1	9282308	11949196	79266005	82140754	-	#49F643	2666888	2874749	205134	-109431	205134	2872022	false
	HGL 1	11839765	11983002	72717257	72866398	-	#49F643	143237	149141	-109431	-23421	-109431	33806	false
	HGL 1	11959581	12027730	79100632	79184584	-	#49F643	68149	83952	-23421	28550	-23421	44728	false
	HGL 1	12056280	12279825	72454516	72716777	-	#49F643	223545	262261	28550	43859	28550	252095	false
	HGL 1	12323684	12486230	72256108	72449341	+	#49F643	162546	193233	43859	9632	43859	206405	false
	HGL 1	12495862	12574430	85396773	85465688	-	#49F643	78568	68915	9632	-1636	9632	88200	false
	HGL 1	12572794	13521307	71089308	72182652	-	#49F643	948513	1093344	-1636	1337	-1636	946877	false
	HGL 1	13522644	13638965	70963554	71086675	+	#49F643	116321	123121	1337	7054	1337	117658	false
	HGL 1	13646019	14956402	69511794	70926647	-	#49F643	1310383	1414853	7054	0	7054	1317437	false
	HGL 1	14956402	19091197	65057740	69455050	-	#49F643	4134795	4397310	0	4431	0	4134795	false
	HGL 1	19095628	30216092	52915879	64948813	-	#49F643	11120464	12032934	4431	-49325	4431	11124895	false
	HGL 1	30166767	41857571	39546336	52846979	-	#49F643	11690804	13300643	-49325	3094	-49325	11641479	false
	HGL 1	41860665	43127206	45687	1411972	-	#49F643	1266541	1366285	3094	4302	3094	1269635	false
	HGL 1	43131508	46911288	1479835	5781366	+	#49F643	3779780	4301531	4302	173130	4302	3784082	false
	HGL 1	47084418	58693814	5782613	18627582	+	#49F643	11609396	12844969	173130	79463	173130	11782526	false
	HGL 1	58773277	61756571	18672407	21821634	+	#49F643	2983294	3149227	79463	1475	79463	3062757	false
	HGL 1	61758046	61891215	21828071	21996674	-	#49F643	133169	168603	1475	11678	1475	134644	false
	HGL 1	61902893	66858664	22002650	27145174	+	#49F643	4955771	5142524	11678	279716	11678	4967449	false
	HGL 1	67138380	74300305	27145268	35295546	+	#49F643	7161925	8150278	279716	34672	279716	7441641	false
	HGL 1	74334977	75237317	35365547	36277564	+	#49F643	902340	912017	34672	38940	34672	937012	false
	HGL 1	75276257	76831860	37738982	39512902	-	#49F643	1555603	1773920	38940	44334	38940	1594543	false
	HGL 1	76876194	77275011	37163395	37555386	-	#49F643	398817	391991	44334	81902	44334	443151	false
	HGL 1	77356913	77660949	36897544	37152419	-	#49F643	304036	254875	81902	1562	81902	385938	false
	HGL 1	77662511	77896964	36600215	36825960	-	#49F643	234453	225745	1562	1620	1562	236015	false
	HGL 1	77898584	77971027	36465690	36549510	-	#49F643	72443	83820	1620	130344	1620	74063	false
	HGL 1	78101371	78198288	36390633	36462984	-	#49F643	96917	72351	130344		130344	227261	false
	HGL 18	78351016	82009440	52555940	56312545	+	#0000FF	3658424	3756605	11274134		11274134	14932558	false
**CPOR 19**	HGL 11	93448	676885	124602111	125233235	+	#7FFFFF	583437	631124		993			

	HGL 11	677878	3004221	90524652	92965901	-	#7FFFFF	2326343	2441249	993	100717	993	2327336	false
	HGL 11	3104938	12731446	80355309	90456343	-	#7FFFFF	9626508	10101034	100717	32589	100717	9727225	false
	HGL 11	12764035	12840994	80355309	80448098	+	#7FFFFF	76959	92789	32589	114772	32589	109548	false
	HGL 11	12955766	21070945	71118419	80253982	-	#7FFFFF	8115179	9135563	114772	57055	114772	8229951	false
	HGL 11	21128000	21787837	70375290	71112500	-	#7FFFFF	659837	737210	57055	2526	57055	716892	false
	HGL 11	21790363	21843561	69466095	69560041	+	#7FFFFF	53198	93946	2526		2526	55724	false
	HGL 15	22666971	23103099	895150	1303932	+	#80797D	436128	408782		6827			
	HGL 15	23109926	33897029	1435670	14018073	+	#80797D	10787103	12582403	6827	29156	6827	10793930	false
	HGL 15	33926185	39543343	14069268	20760641	+	#80797D	5617158	6691373	29156	61973	29156	5646314	false
	HGL 15	39605316	40386575	20760679	21611070	+	#80797D	781259	850391	61973	47	61973	843232	false
	HGL 15	40386622	42872963	21673126	24681829	+	#80797D	2486341	3008703	47	81505	47	2486388	false
	HGL 15	42954468	43097018	44700933	44844868	-	#80797D	142550	143935	81505	97199	81505	224055	false
	HGL 15	43194217	48395051	39145064	44700933	-	#80797D	5200834	5555869	97199	27293	97199	5298033	false
	HGL 15	48422344	58565098	27799517	39093691	-	#80797D	10142754	11294174	27293	4653	27293	10170047	false
	HGL 15	58569751	58692137	27607265	27790240	+	#80797D	122386	182975	4653	6	4653	127039	false
	HGL 15	58692143	61080017	24701723	27601516	-	#80797D	2387874	2899793	6		6	2387880	false
**CPOR 2**	HGL 11	157227	382040	103511297	103723969	-	#7FFFFF	224813	212672		734			
	HGL 11	382774	5446250	98200986	103692749	-	#7FFFFF	5063476	5491763	734	449	734	5064210	false
	HGL 11	5446699	6984295	95249259	96936093	-	#7FFFFF	1537596	1686834	449	53853	449	1538045	false
	HGL 11	7038148	9141189	92965901	95244533	-	#7FFFFF	2103041	2278632	53853	200851	53853	2156894	false
	HGL 11	9342040	9465414	124522512	124601580	-	#7FFFFF	123374	79068	200851	-67875	200851	324225	false
	HGL 9	9397539	9468457	24793872	24862832	-	#036A9C	70918	68960		-70685			
	HGL 11	9397539	9504655	124245912	124326602	+	#7FFFFF	107116	80690	-67875	-96239	-67875	39241	false
	HGL 9	9397772	9472639	20712901	20806083	-	#036A9C	74867	93182	-70685	-20442	-70685	4182	false
	HGL 22	9399536	9465431	2613308	2671362	-	#EC83BA	65895	58054		294506			
	HGL 11	9408416	9534495	68815299	68907908	-	#7FFFFF	126079	92609	-96239	-67296	-96239	29840	false
	HGL 9	9452197	9534495	20596610	20675356	-	#036A9C	82298	78746	-20442	85201	-20442	61856	false
	HGL 11	9467199	9526010	66451886	66567430	+	#7FFFFF	58811	115544	-67296	71622	-67296	-8485	true
	HGL 11	9597632	9708042	122965014	123059220	-	#7FFFFF	110410	94206	71622	39346	71622	182032	false
	HGL 9	9619696	9697887	21504164	21600366	+	#036A9C	78191	96202	85201	57832	85201	163392	false
	HGL 11	9747388	9877042	70023413	70100634	+	#7FFFFF	129654	77221	39346	392140	39346	169000	false
	HGL 9	9755719	9859408	23903199	23977791	-	#036A9C	103689	74592	57832	-103689	57832	161521	false

	HGL 9	9755719	9877589	22009576	22097805	-	#036A9C	121870	88229	-103689	-121870	-103689	18181	false
	HGL 9	9755719	9877729	23944222	24000542	-	#036A9C	122010	56320	-121870	-122010	-121870	140	false
	HGL 9	9755719	9883984	25085106	25195090	+	#036A9C	128265	109984	-122010	-124159	-122010	6255	false
	HGL 9	9759825	9912476	25146811	25299519	+	#036A9C	152651	152708	-124159	19222663	-124159	28492	false
	HGL 22	9759937	9912476	2213740	2360401	-	#EC83BA	152539	146661	294506	19359204	294506	447045	false
	HGL 11	10269182	11070674	103757897	104535409	+	#7FFFFF	801492	777512	392140	52468	392140	1193632	false
	HGL 11	11123142	12507568	104577276	106173753	+	#7FFFFF	1384426	1596477	52468	51119	52468	1436894	false
	HGL 11	12558687	28486386	106228323	122808356	+	#7FFFFF	15927699	16580033	51119	165955	51119	15978818	false
	HGL 11	28652341	28741314	123295521	123406195	-	#7FFFFF	88973	110674	165955	5994	165955	254928	false
	HGL 11	28747308	29011108	123159811	123371531	-	#7FFFFF	263800	211720	5994	128608	5994	269794	false
	HGL 9	29135139	29222362	23711618	23787383	-	#036A9C	87223	75765	19222663	235179	19222663	19309886	false
	HGL 11	29139716	29203892	123558019	123613277	-	#7FFFFF	64176	55258	128608	53276	128608	192784	false
	HGL 11	29257168	29314448	68420448	68503737	+	#7FFFFF	57280	83289	53276	143093	53276	110556	false
	HGL 22	29271680	29379843	2454786	2532457	-	#EC83BA	108163	77671	19359204	164989	19359204	19467367	false
	HGL 11	29457541	29516434	67338625	67418193	-	#7FFFFF	58893	79568	143093	28398	143093	201986	false
	HGL 9	29457541	29521906	20452633	20568406	+	#036A9C	64365	115773	235179	3689	235179	299544	false
	HGL 9	29525595	29640420	23393185	23473178	+	#036A9C	114825	79993	3689	-104355	3689	118514	false
	HGL 9	29536065	29640468	33174006	33234091	-	#036A9C	104403	60085	-104355	-32490	-104355	48	false
	HGL 11	29544832	29693780	123969688	124030020	+	#7FFFFF	148948	60332	28398	-112330	28398	177346	false
	HGL 22	29544832	29768740	2169577	2311747	+	#EC83BA	223908	142170	164989	60812	164989	388897	false
	HGL 11	29581450	29662335	67386901	67446720	+	#7FFFFF	80885	59819	-112330	28008	-112330	-31445	true
	HGL 9	29607978	29662335	22103601	22169512	-	#036A9C	54357	65911	-32490	-413	-32490	21867	false
	HGL 9	29661922	29771238	21885719	21944567	+	#036A9C	109316	58848	-413	-103348	-413	108903	false
	HGL 9	29667890	29760072	21899929	21974500	-	#036A9C	92182	74571	-103348	75728	-103348	-11166	true
	HGL 11	29690343	29757466	70044139	70100634	-	#7FFFFF	67123	56495	28008	338614	28008	95131	false
	HGL 22	29829552	29883519	2262584	2360401	-	#EC83BA	53967	97817	60812	567903	60812	114779	false
	HGL 9	29835800	29887758	23903199	23991762	+	#036A9C	51958	88563	75728	617060	75728	127686	false
	HGL 11	30096080	30250387	69939983	70026044	-	#7FFFFF	154307	86061	338614	-54641	338614	492921	false
	HGL 11	30195746	30250387	67027071	67081004	+	#7FFFFF	54641	53933	-54641	164784	-54641	0	true
	HGL 11	30415171	30484384	69034787	69111131	+	#7FFFFF	69213	76344	164784	-33691	164784	233997	false
	HGL 11	30450693	30537757	66173563	66255615	-	#7FFFFF	87064	82052	-33691	463727	-33691	53373	false
	HGL 22	30451422	30545079	1233369	1297870	+	#EC83BA	93657	64501	567903	211254	567903	661560	false

	HGL 9	30504818	30571773	27096482	27153529	-	#036A9C	66955	57047	617060	82987	617060	684015	false
	HGL 9	30654760	30759556	24527022	24646875	-	#036A9C	104796	119853	82987	-4099	82987	187783	false
	HGL 9	30755457	30813547	27414145	27476588	-	#036A9C	58090	62443	-4099	423313	-4099	53991	false
	HGL 22	30756333	30812905	319974	373682	-	#EC83BA	56572	53708	211254	428211	211254	267826	false
	HGL 11	31001484	31118279	67027071	67230510	-	#7FFFFF	116795	203439	463727	-94202	463727	580522	false
	HGL 11	31024077	31190922	69846611	70071339	+	#7FFFFF	166845	224728	-94202	-71727	-94202	72643	false
	HGL 11	31119195	31193959	123413674	123507476	+	#7FFFFF	74764	93802	-71727	30156	-71727	3037	false
	HGL 11	31224115	31286711	70023414	70087489	+	#7FFFFF	62596	64075	30156	392260	30156	92752	false
	HGL 9	31236860	31308288	23903199	23991762	-	#036A9C	71428	88563	423313	48158	423313	494741	false
	HGL 22	31241116	31309382	2262584	2344268	+	#EC83BA	68266	81684	428211	-57091	428211	496477	false
	HGL 22	31252291	31309424	2293603	2360401	-	#EC83BA	57133	66798	-57091	327842	-57091	42	false
	HGL 9	31356446	31465707	24556127	24666303	+	#036A9C	109261	110176	48158	113426	48158	157419	false
	HGL 9	31579133	31656406	24561333	24662580	+	#036A9C	77273	101247	113426	309611	113426	190699	false
	HGL 22	31637266	31704555	1227291	1304989	+	#EC83BA	67289	77698	327842	319814	327842	395131	false
	HGL 11	31678971	31758091	67901509	67969578	+	#7FFFFF	79120	68069	392260	280761	392260	471380	false
	HGL 9	31966017	32042607	24571393	24662119	+	#036A9C	76590	90726	309611	82361	309611	386201	false
	HGL 22	32024369	32141770	1227295	1304989	+	#EC83BA	117401	77694	319814	-22916	319814	437215	false
	HGL 11	32038852	32114500	66168824	66239659	-	#7FFFFF	75648	70835	280761	-70528	280761	356409	false
	HGL 11	32043972	32199915	68586695	68705841	+	#7FFFFF	155943	119146	-70528	-75619	-70528	85415	false
	HGL 22	32118854	32200387	1615372	1673106	+	#EC83BA	81533	57734	-22916	10074	-22916	58617	false
	HGL 11	32124296	32210115	67102804	67301024	-	#7FFFFF	85819	198220	-75619	-31469	-75619	10200	false
	HGL 9	32124968	32186351	27162226	27225115	+	#036A9C	61383	62889	82361	48620	82361	143744	false
	HGL 11	32178646	32296526	66124660	66240131	+	#7FFFFF	117880	115471	-31469	-10014	-31469	86411	false
	HGL 22	32210461	32299739	1226999	1293122	-	#EC83BA	89278	66123	10074	100945	10074	99352	false
	HGL 9	32234971	32291007	27019834	27087685	-	#036A9C	56036	67851	48620	2338	48620	104656	false
	HGL 11	32286512	32370723	69059925	69111009	-	#7FFFFF	84211	51084	-10014	176686	-10014	74197	false
	HGL 9	32293345	32403818	24527022	24662118	-	#036A9C	110473	135096	2338	-4001	2338	112811	false
	HGL 9	32399817	32453790	27413205	27476588	-	#036A9C	53973	63383	-4001	-53591	-4001	49972	false
	HGL 9	32400199	32457829	22424199	22501430	+	#036A9C	57630	77231	-53591		-53591	4039	false
	HGL 22	32400684	32452121	319974	373682	-	#EC83BA	51437	53708	100945		100945	152382	false
	HGL 11	32547409	32626072	69940466	70020425	-	#7FFFFF	78663	79959	176686		176686	255349	false
	HGL 6	32680360	34122398	76936750	78593218	+	#980157	1442038	1656468					

	HGL 13	34235967	34293120	48998029	49049628	+	#EBE47D	57153	51599		129111			
	HGL 13	34422231	34517608	49353088	49446722	+	#EBE47D	95377	93634	129111	54002	129111	224488	false
	HGL 13	34571610	35094329	49519302	50078899	+	#EBE47D	522719	559597	54002	9713	54002	576721	false
	HGL 13	35104042	36373193	50154091	51538333	+	#EBE47D	1269151	1384242	9713	3	9713	1278864	false
	HGL 13	36373196	38228804	51747519	53756929	+	#EBE47D	1855608	2009410	3	6638	3	1855611	false
	HGL 13	38235442	38323199	53810754	53881539	+	#EBE47D	87757	70785	6638	62637	6638	94395	false
	HGL 13	38385836	38799010	53883201	54381365	+	#EBE47D	413174	498164	62637	60805	62637	475811	false
	HGL 13	38859815	39423303	54462535	55053259	+	#EBE47D	563488	590724	60805	54000	60805	624293	false
	HGL 13	39477303	39664425	55053262	55307417	+	#EBE47D	187122	254155	54000	116045	54000	241122	false
	HGL 13	39780470	39836563	55703292	55768555	+	#EBE47D	56093	65263	116045	-31598	116045	172138	false
	HGL 13	39804965	43393725	55768559	59496814	+	#EBE47D	3588760	3728255	-31598	1459	-31598	3557162	false
	HGL 13	43395184	50475008	59546964	66986476	+	#EBE47D	7079824	7439512	1459	11996	1459	7081283	false
	HGL 13	50487004	50700333	51561776	51747260	-	#EBE47D	213329	185484	11996	-58691	11996	225325	false
	HGL 13	50641642	52506423	66986881	68850524	+	#EBE47D	1864781	1863643	-58691	4292	-58691	1806090	false
	HGL 13	52510715	53007238	68918105	69336112	+	#EBE47D	496523	418007	4292	453	4292	500815	false
	HGL 13	53007691	53324703	69395028	69678433	+	#EBE47D	317012	283405	453	72972	453	317465	false
	HGL 13	53397675	71353990	69681071	87311750	+	#EBE47D	17956315	17630679	72972		72972	18029287	false
	HGL 21	71654297	73123673	78999673	80564350	+	#FFFF00	1469376	1564677		50195			
	HGL 21	73173868	74866429	77194733	78975180	-	#FFFF00	1692561	1780447	50195	61010	50195	1742756	false
	HGL 21	74927439	75411087	76760503	77190070	-	#FFFF00	483648	429567	61010	21220	61010	544658	false
	HGL 21	75432307	76070451	75368535	76072257	-	#FFFF00	638144	703722	21220	334032	21220	659364	false
	HGL 21	76404483	76542256	76459011	76642786	+	#FFFF00	137773	183775	334032	-78463	334032	471805	false
	HGL 21	76463793	76585792	75182325	75313765	-	#FFFF00	121999	131440	-78463	14336	-78463	43536	false
	HGL 21	76600128	82532622	68989443	75322653	-	#FFFF00	5932494	6333210	14336	60352	14336	5946830	false
	HGL 21	82592974	84430031	66958331	68985347	-	#FFFF00	1837057	2027016	60352	68415	60352	1897409	false
	HGL 21	84498446	86592171	64742761	66938796	-	#FFFF00	2093725	2196035	68415	75437	68415	2162140	false
	HGL 21	86667608	89951413	61119650	64742718	-	#FFFF00	3283805	3623068	75437	1234	75437	3359242	false
	HGL 21	89952647	97569396	52873986	61069490	-	#FFFF00	7616749	8195504	1234	922	1234	7617983	false
	HGL 21	97570318	97643095	52794714	52872757	+	#FFFF00	72777	78043	922	0	922	73699	false
	HGL 21	97643095	97916449	52526116	52792527	-	#FFFF00	273354	266411	0	22668	0	273354	false
	HGL 21	97939117	97992836	52407830	52463018	-	#FFFF00	53719	55188	22668	1221	22668	76387	false
	HGL 21	97994057	98080650	52337650	52405755	+	#FFFF00	86593	68105	1221	5038	1221	87814	false

	HGL 21	98085688	113470442	35489972	52331615	-	#FFFF00	15384754	16841643	5038	-652	5038	15389792	false
	HGL 21	113469790	145753126	690587	35412293	-	#FFFF00	32283336	34721706	-652	3341	-652	32282684	false
	HGL 21	145756467	145850575	469778	570800	-	#FFFF00	94108	101022	3341		3341	97449	false
**CPOR 20**	HGL 4	250588	340777	478306	534182	-	#5654E8	90189	55876		4500			
	HGL 4	345277	398986	360945	412620	+	#5654E8	53709	51675	4500	495111	4500	58209	false
	HGL 4	894097	1002514	305751	360832	-	#5654E8	108417	55081	495111	474618	495111	603528	false
	HGL 4	1477132	1563360	106400	238005	+	#5654E8	86228	131605	474618	69608	474618	560846	false
	HGL 4	1632968	1706193	20904345	20975788	+	#5654E8	73225	71443	69608	-71303	69608	142833	false
	HGL 4	1634890	1721997	360945	412620	-	#5654E8	87107	51675	-71303	298531	-71303	15804	false
	HGL 4	2020528	2281889	560368	867502	+	#5654E8	261361	307134	298531	-4770	298531	559892	false
	HGL 4	2277119	2351818	819910	952293	-	#5654E8	74699	132383	-4770	9219	-4770	69929	false
	HGL 4	2361037	2419337	958586	1018759	+	#5654E8	58300	60173	9219	110228	9219	67519	false
	HGL 4	2529565	2837756	1018820	1363217	+	#5654E8	308191	344397	110228	905125	110228	418419	false
	HGL 4	3742881	5934364	1373685	3582787	+	#5654E8	2191483	2209102	905125	180605	905125	3096608	false
	HGL 4	6114969	26273133	3548215	24525755	+	#5654E8	20158164	20977540	180605	-40898	180605	20338769	false
	HGL 4	26232235	26300578	24524056	24606524	-	#5654E8	68343	82468	-40898	-67770	-40898	27445	false
	HGL 4	26232808	26658865	24525461	24917607	+	#5654E8	426057	392146	-67770	351716	-67770	358287	false
	HGL 4	27010581	27072921	24908918	24968963	+	#5654E8	62340	60045	351716	403	351716	414056	false
	HGL 4	27073324	44803084	25114270	44714024	+	#5654E8	17729760	19599754	403	18	403	17730163	false
	HGL 4	44803102	44980901	44768540	44959540	-	#5654E8	177799	191000	18	8866	18	177817	false
	HGL 4	44989767	45341506	44961383	45373385	+	#5654E8	351739	412002	8866	118618	8866	360605	false
	HGL 4	45460124	45513608	45411548	45482204	+	#5654E8	53484	70656	118618	-48843	118618	172102	false
	HGL 4	45464765	45564231	45410506	45484342	-	#5654E8	99466	73836	-48843	59943	-48843	50623	false
	HGL 4	45624174	58609093	45526180	59663446	+	#5654E8	12984919	14137266	59943	3705	59943	13044862	false
	HGL 4	58612798	59977223	59720937	61096182	+	#5654E8	1364425	1375245	3705	-65962	3705	1368130	false
	HGL 4	59911261	61053147	61137544	62352894	+	#5654E8	1141886	1215350	-65962	-989920	-65962	1075924	false
	HGL 4	60063227	60192413	61033626	61107245	+	#5654E8	129186	73619	-989920	877817	-989920	-860734	true
	HGL 4	61070230	61244611	62480804	62693960	-	#5654E8	174381	213156	877817	0	877817	1052198	false
	HGL 4	61244611	61321917	62370495	62470071	+	#5654E8	77306	99576	0	34082	0	77306	false
	HGL 4	61355999	61549160	62698125	62956996	+	#5654E8	193161	258871	34082	0	34082	227243	false
	HGL 4	61549160	61628790	62965389	63021150	-	#5654E8	79630	55761	0	0	0	79630	false
	HGL 4	61628790	64556561	63035170	66239990	+	#5654E8	2927771	3204820	0	139973	0	2927771	false

	HGL 4	64696534	68959858	66240048	70846679	+	#5654E8	4263324	4606631	139973		139973	4403297	false
**CPOR 21**	HGL 18	309071	587642	46357169	46615687	-	#0000FF	278571	258518		-465			
	HGL 18	587177	4783262	37073272	41942454	+	#0000FF	4196085	4869182	-465	4522	-465	4195620	false
	HGL 18	4787784	6198791	35473488	37070381	-	#0000FF	1411007	1596893	4522	20918	4522	1415529	false
	HGL 18	6219709	7236023	34422804	35536784	+	#0000FF	1016314	1113980	20918	3050	20918	1037232	false
	HGL 18	7239073	8445669	33209680	34480440	+	#0000FF	1206596	1270760	3050	42345	3050	1209646	false
	HGL 18	8488014	9736131	31853024	33209282	-	#0000FF	1248117	1356258	42345	2681	42345	1290462	false
	HGL 18	9738812	10544768	30948727	31757369	+	#0000FF	805956	808642	2681	1047585	2681	808637	false
	HGL 22	10582813	10638346	8244145	8339056	+	#EC83BA	55533	94911		140665			
	HGL 22	10779011	10845014	8633876	8687009	+	#EC83BA	66003	53133	140665	10403402	140665	206668	false
	HGL 18	11592353	11936039	58063362	58419887	-	#0000FF	343686	356525	1047585	172716	1047585	1391271	false
	HGL 18	12108755	13027337	58527500	59496569	-	#0000FF	918582	969069	172716	147043	172716	1091298	false
	HGL 18	13174380	13248120	58027644	58083910	+	#0000FF	73740	56266	147043	82883	147043	220783	false
	HGL 18	13331003	13855351	57561462	58076143	-	#0000FF	524348	514681	82883	72199	82883	607231	false
	HGL 18	13927550	15336780	56169334	57538147	-	#0000FF	1409230	1368813	72199	111643	72199	1481429	false
	HGL 18	15448423	18139735	59516446	62280051	+	#0000FF	2691312	2763605	111643	66401	111643	2802955	false
	HGL 18	18206136	18301127	62280051	62364231	+	#0000FF	94991	84180	66401	26862	66401	161392	false
	HGL 18	18327989	20101749	62493224	64180580	+	#0000FF	1773760	1687356	26862	55403	26862	1800622	false
	HGL 18	20157152	20890131	64180609	64828114	+	#0000FF	732979	647505	55403	729209	55403	788382	false
	HGL 22	21248416	21324507	8129636	8181156	+	#EC83BA	76091	51520	10403402	33644	10403402	10479493	false
	HGL 22	21358151	21418773	8161637	8238617	+	#EC83BA	60622	76980	33644		33644	94266	false
	HGL 18	21619340	21945499	44997248	45329786	-	#0000FF	326159	332538	729209	39750	729209	1055368	false
	HGL 18	21985249	24480414	42038553	44716691	-	#0000FF	2495165	2678138	39750	128089	39750	2534915	false
	HGL 18	24608503	24659410	56066725	56131798	+	#0000FF	50907	65073	128089	18993	128089	178996	false
	HGL 18	24678403	24754197	56239813	56312545	+	#0000FF	75794	72732	18993	36843	18993	94787	false
	HGL 18	24791040	24900605	46189975	46333714	-	#0000FF	109565	143739	36843	-9462	36843	146408	false
	HGL 18	24891143	30508655	46616617	52519128	+	#0000FF	5617512	5902511	-9462	136033	-9462	5608050	false
	HGL 18	30644688	31242615	45450859	46148468	-	#0000FF	597927	697609	136033		136033	733960	false
**CPOR 22**	HGL 8	56954	909205	29342912	30068981	+	#00FF7F	852251	726069		63171			
	HGL 8	972376	1858032	32179487	32958140	-	#00FF7F	885656	778653	63171	55632	63171	948827	false
	HGL 8	1913664	4214281	30068987	32170530	-	#00FF7F	2300617	2101543	55632	373876	55632	2356249	false
	HGL 6	4370078	4440720	95856213	95994097	-	#980157	70642	137884		17438			

	HGL 6	4458158	4511182	97975624	98027174	-	#980157	53024	51550	17438	187631	17438	70462	false
	HGL 8	4588157	4657851	32960567	33035695	+	#00FF7F	69694	75128	373876	15499259	373876	443570	false
	HGL 6	4698813	4964490	87282749	87539703	+	#980157	265677	256954	187631	0	187631	453308	false
	HGL 6	4964490	6104822	87609390	88797449	+	#980157	1140332	1188059	0	78583	0	1140332	false
	HGL 6	6183405	6863375	88853262	89593589	+	#980157	679970	740327	78583	107960	78583	758553	false
	HGL 6	6971335	7356584	89664001	90053070	-	#980157	385249	389069	107960	6059	107960	493209	false
	HGL 6	7362643	7818934	90066654	90546222	+	#980157	456291	479568	6059	0	6059	462350	false
	HGL 6	7818934	7908214	90556096	90665896	-	#980157	89280	109800	0	401627	0	89280	false
	HGL 6	8309841	8421187	103134355	103224621	-	#980157	111346	90266	401627	73604	401627	512973	false
	HGL 6	8494791	9399818	93125128	93649799	+	#980157	905027	524671	73604	-774523	73604	978631	false
	HGL 6	8625295	8981375	93125128	93199604	+	#980157	356080	74476	-774523	-300363	-774523	-418443	true
	HGL 6	8681012	8984179	93022741	93073369	-	#980157	303167	50628	-300363	500612	-300363	2804	false
	HGL 6	9484791	11798581	93668413	95990731	+	#980157	2313790	2322318	500612	45024	500612	2814402	false
	HGL 6	11843605	11981309	90670591	90794167	+	#980157	137704	123576	45024	121596	45024	182728	false
	HGL 6	12102905	12200135	98366507	98418216	-	#980157	97230	51709	121596	41407	121596	218826	false
	HGL 6	12241542	17403874	95993183	101089990	+	#980157	5162332	5096807	41407	181583	41407	5203739	false
	HGL 6	17585457	18307956	92289560	93005856	+	#980157	722499	716296	181583	84965	181583	904082	false
	HGL 6	18392921	18454171	93012685	93070739	-	#980157	61250	58054	84965	62037	84965	146215	false
	HGL 6	18516208	18640383	93138982	93203713	+	#980157	124175	64731	62037	-56011	62037	186212	false
	HGL 6	18584372	18638986	93067817	93145138	-	#980157	54614	77321	-56011	56515	-56011	-1397	true
	HGL 6	18695501	19237578	91752075	92268788	-	#980157	542077	516713	56515	558	56515	598592	false
	HGL 6	19238136	20017854	90827682	91675542	-	#980157	779718	847860	558	204385	558	780276	false
	HGL 6	20222239	22281942	101090320	103210437	+	#980157	2059703	2120117	204385	222284	204385	2264088	false
	HGL 6	22504226	24440822	103308926	104929248	-	#980157	1936596	1620322	222284	99551	222284	2158880	false
	HGL 6	24540373	24796914	104930142	105129292	+	#980157	256541	199150	99551	230551	99551	356092	false
	HGL 6	25027465	25120606	105154424	105228490	+	#980157	93141	74066	230551	1225203	230551	323692	false
	HGL 11	25153681	25391765	97941386	98202672	+	#7FFFFF	238084	261286		421			
	HGL 11	25392186	26335048	96841577	97941331	+	#7FFFFF	942862	1099754	421		421	943283	false
	HGL 6	26345809	26440086	105231000	105291485	+	#980157	94277	60485	1225203		1225203	1319480	false
	HGL 8	27445936	27624094	29208854	29333729	+	#00FF7F	178158	124875	7226574	-27256	7226574	7404732	false
	HGL 8	27596838	27678930	29234844	29315614	+	#00FF7F	82092	80770	-27256	-12936	-27256	54836	false
	HGL 8	27665994	27865349	29212144	29333724	+	#00FF7F	199355	121580	-12936	-58598	-12936	186419	false

	HGL 8	27806751	27911067	29211931	29315614	+	#00FF7F	104316	103683	-58598	41164	-58598	45718	false
	HGL 8	27952231	28036029	29210582	29306596	+	#00FF7F	83798	96014	41164	190289	41164	124962	false
	HGL 8	28226318	28366871	29031373	29186502	-	#00FF7F	140553	155129	190289	101117	190289	330842	false
	HGL 8	28467988	28846759	28781069	29054715	-	#00FF7F	378771	273646	101117	74101	101117	479888	false
	HGL 8	28920860	29380303	28464887	28758909	-	#00FF7F	459443	294022	74101	62683	74101	533544	false
	HGL 8	29442986	29797385	28220109	28464356	-	#00FF7F	354399	244247	62683	55734	62683	417082	false
	HGL 8	29853119	30046703	28074857	28218092	-	#00FF7F	193584	143235	55734	19091	55734	249318	false
	HGL 8	30065794	30125594	28218258	28306427	-	#00FF7F	59800	88169	19091	89729	19091	78891	false
	HGL 8	30215323	30272471	28220109	28306014	+	#00FF7F	57148	85905	89729	16445	89729	146877	false
	HGL 8	30288916	30496157	28067454	28218092	+	#00FF7F	207241	150638	16445	29772	16445	223686	false
	HGL 8	30525929	30754056	27871215	28094106	-	#00FF7F	228127	222891	29772	116444	29772	257899	false
	HGL 8	30870500	31345924	27169720	27517582	-	#00FF7F	475424	347862	116444	61856	116444	591868	false
	HGL 8	31407780	31604509	27107680	27229749	+	#00FF7F	196729	122069	61856	108333	61856	258585	false
	HGL 8	31712842	32029561	27104234	27269766	-	#00FF7F	316719	165532	108333	31837	108333	425052	false
	HGL 8	32061398	32534264	26749234	27040517	-	#00FF7F	472866	291283	31837	851	31837	504703	false
	HGL 8	32535115	32601929	26653372	26722530	+	#00FF7F	66814	69158	851	-6667	851	67665	false
	HGL 8	32595262	32665879	26605348	26656123	-	#00FF7F	70617	50775	-6667	288561	-6667	63950	false
	HGL 8	32954440	33076790	26368118	26432122	-	#00FF7F	122350	64004	288561	161686	288561	410911	false
	HGL 8	33238476	33338708	26483451	26556401	+	#00FF7F	100232	72950	161686	24300	161686	261918	false
	HGL 8	33363008	36122066	23782689	26358442	-	#00FF7F	2759058	2575753	24300	65774	24300	2783358	false
	HGL 8	36187840	40963979	18735637	23753970	-	#00FF7F	4776139	5018333	65774	11809	65774	4841913	false
	HGL 8	40975788	41090358	18611421	18716779	+	#00FF7F	114570	105358	11809	16351	11809	126379	false
	HGL 8	41106709	42274749	17347277	18606766	-	#00FF7F	1168040	1259489	16351	4961	16351	1184391	false
	HGL 8	42279710	42338964	17347472	17475477	-	#00FF7F	59254	128005	4961	0	4961	64215	false
	HGL 8	42338964	42509617	17347502	17535086	-	#00FF7F	170653	187584	0	48863	0	170653	false
	HGL 8	42558480	42713091	17345518	17517083	-	#00FF7F	154611	171565	48863	9632	48863	203474	false
	HGL 8	42722723	44663963	15282449	17244682	-	#00FF7F	1941240	1962233	9632	138252	9632	1950872	false
	HGL 8	44802215	48277873	11681325	15279874	-	#00FF7F	3475658	3598549	138252	114491	138252	3613910	false
	HGL 8	48392364	48564060	11408426	11464738	+	#00FF7F	171696	56312	114491	276147	114491	286187	false
	HGL 8	48840207	53346445	6646059	11482057	-	#00FF7F	4506238	4835998	276147	337811	276147	4782385	false
	HGL 8	53684256	53807460	6565062	6644298	+	#00FF7F	123204	79236	337811	1383	337811	461015	false
	HGL 8	53808843	53943904	6565062	6621752	-	#00FF7F	135061	56690	1383	210658	1383	136444	false

	HGL 8	54154562	59846039	10448	6595891	-	#00FF7F	5691477	6585443	210658		210658	5902135	false
**CPOR 23**	HGL 23	141132	414265	19468	265333	+	#0A803B	273133	245865		9103			
	HGL 23	423368	1793268	383507	1794291	+	#0A803B	1369900	1410784	9103	-4509	9103	1379003	false
	HGL 23	1788759	1867776	1781028	1905714	-	#0A803B	79017	124686	-4509	14599	-4509	74508	false
	HGL 23	1882375	16859081	1908479	17433939	+	#0A803B	14976706	15525460	14599	55163	14599	14991305	false
	HGL 23	16914244	18750814	17433939	19347185	+	#0A803B	1836570	1913246	55163	237326	55163	1891733	false
	HGL 23	18988140	21200050	19385716	21711100	+	#0A803B	2211910	2325384	237326	32434	237326	2449236	false
	HGL 23	21232484	24428580	21763180	25142155	+	#0A803B	3196096	3378975	32434	0	32434	3228530	false
	HGL 23	24428580	24479816	25142155	25197990	-	#0A803B	51236	55835	0	12	0	51236	false
	HGL 23	24479828	41317040	25198004	43050052	+	#0A803B	16837212	17852048	12	-10102	12	16837224	false
	HGL 23	41306938	45546971	43050195	47238072	+	#0A803B	4240033	4187877	-10102	4341	-10102	4229931	false
	HGL 23	45551312	49589676	47291186	51723647	+	#0A803B	4038364	4432461	4341	259	4341	4042705	false
	HGL 23	49589935	49678494	51598450	51725736	-	#0A803B	88559	127286	259	605	259	88818	false
	HGL 23	49679099	50499899	52602330	53415983	-	#0A803B	820800	813653	605	58303	605	821405	false
	HGL 23	50558202	50871475	52171408	52564328	-	#0A803B	313273	392920	58303	20006	58303	371576	false
	HGL 23	50891481	51768839	51972500	52964792	-	#0A803B	877358	992292	20006	30307	20006	897364	false
	HGL 23	51799146	52029156	51762075	52006024	-	#0A803B	230010	243949	30307	9783	30307	260317	false
	HGL 23	52038939	52421388	51725736	52141491	+	#0A803B	382449	415755	9783	5551	9783	392232	false
	HGL 23	52426939	56175058	53068313	57103094	+	#0A803B	3748119	4034781	5551	57260	5551	3753670	false
	HGL 23	56232318	58635496	57103094	59620344	+	#0A803B	2403178	2517250	57260	5389	57260	2460438	false
	HGL 23	58640885	58734530	59621457	59704325	-	#0A803B	93645	82868	5389	481	5389	99034	false
	HGL 23	58735011	64735842	59704325	66129806	+	#0A803B	6000831	6425481	481	101758	481	6001312	false
	HGL 23	64837600	65177397	66202433	66569371	+	#0A803B	339797	366938	101758	133627	101758	441555	false
	HGL 23	65311024	67944660	66572940	69225831	+	#0A803B	2633636	2652891	133627	19793	133627	2767263	false
	HGL 23	67964453	68194136	69275856	69467224	+	#0A803B	229683	191368	19793		19793	249476	false
**CPOR 24**	HGL 3	32107	480548	19415001	19839328	+	#C3A652	448441	424327		156880			
	HGL 3	637428	692479	28135102	28208405	-	#C3A652	55051	73303	156880	118551	156880	211931	false
	HGL 3	811030	1147714	20731602	21082924	+	#C3A652	336684	351322	118551	32842	118551	455235	false
	HGL 3	1180556	1250828	27785181	27872408	+	#C3A652	70272	87227	32842	200288	32842	103114	false
	HGL 3	1451116	1543839	1838830	1898802	+	#C3A652	92723	59972	200288	188495	200288	293011	false
	HGL 3	1732334	2769386	26611957	27651562	-	#C3A652	1037052	1039605	188495	0	188495	1225547	false
	HGL 3	2769386	2860521	26543053	26611954	+	#C3A652	91135	68901	0	7	0	91135	false

	HGL 3	2860528	9432522	19849652	26543051	-	#C3A652	6571994	6693399	7	117345	7	6572001	false
	HGL 3	9549867	9621699	27785181	27872408	-	#C3A652	71832	87227	117345	40537	117345	189177	false
	HGL 3	9662236	9719978	28121566	28204429	+	#C3A652	57742	82863	40537	233293	40537	98279	false
	HGL 3	9953271	10825708	31625486	32537606	-	#C3A652	872437	912120	233293	100	233293	1105730	false
	HGL 3	10825808	11208010	30641360	31000428	+	#C3A652	382202	359068	100	45447	100	382302	false
	HGL 3	11253457	11720990	31368995	31887710	-	#C3A652	467533	518715	45447	137729	45447	512980	false
	HGL 3	11858719	12274375	29915028	30312034	-	#C3A652	415656	397006	137729	1093	137729	553385	false
	HGL 3	12275468	13344658	30625271	31625475	+	#C3A652	1069190	1000204	1093	452	1093	1070283	false
	HGL 3	13345110	16330459	27577211	30641352	-	#C3A652	2985349	3064141	452	18974	452	2985801	false
	HGL 3	16349433	17011003	28004209	28665757	-	#C3A652	661570	661548	18974	203823	18974	680544	false
	HGL 3	17214826	17432823	32542975	32702238	+	#C3A652	217997	159263	203823	109115	203823	421820	false
	HGL 3	17541938	20996831	16207509	19402663	-	#C3A652	3454893	3195154	109115	48679	109115	3564008	false
	HGL 3	21045510	21309914	15972735	16248787	-	#C3A652	264404	276052	48679	148742	48679	313083	false
	HGL 3	21458656	21534628	15846935	15921999	+	#C3A652	75972	75064	148742	745239	148742	224714	false
	HGL 3	22279867	23459535	1211	1233567	+	#C3A652	1179668	1232356	745239	1549	745239	1924907	false
	HGL 3	23461084	24033900	1357306	1914804	+	#C3A652	572816	557498	1549	83253	1549	574365	false
	HGL 3	24117153	24724347	1837557	2479590	+	#C3A652	607194	642033	83253	126884	83253	690447	false
	HGL 3	24851231	25057302	2458496	2696053	-	#C3A652	206071	237557	126884	61058	126884	332955	false
	HGL 3	25118360	25190551	1837464	1908307	-	#C3A652	72191	70843	61058	76448	61058	133249	false
	HGL 3	25266999	25403899	2510734	2695920	+	#C3A652	136900	185186	76448	145406	76448	213348	false
	HGL 3	25549305	27443112	2663479	4703209	-	#C3A652	1893807	2039730	145406	0	145406	2039213	false
	HGL 3	27443112	27518624	2458496	2580905	-	#C3A652	75512	122409	0	46328	0	75512	false
	HGL 3	27564952	27626192	4627142	4691243	+	#C3A652	61240	64101	46328	11003	46328	107568	false
	HGL 3	27637195	27700466	1841653	1905996	-	#C3A652	63271	64343	11003	22559	11003	74274	false
	HGL 3	27723025	28230428	4723389	5225091	+	#C3A652	507403	501702	22559	77539	22559	529962	false
	HGL 3	28307967	28469938	5239395	5415647	+	#C3A652	161971	176252	77539	99129	77539	239510	false
	HGL 3	28569067	33444704	5405785	10121499	+	#C3A652	4875637	4715714	99129	-2833315	99129	4974766	false
	HGL 3	30611389	30696810	10244215	10310958	+	#C3A652	85421	66743	-2833315	2600912	-2833315	-2747894	true
	HGL 3	33297722	33443509	10052157	10121439	+	#C3A652	145787	69282	2600912	-138648	2600912	2746699	false
	HGL 3	33304861	33600140	10097063	10369058	+	#C3A652	295279	271995	-138648	-43604	-138648	156631	false
	HGL 3	33556536	38447014	10444159	15611287	+	#C3A652	4890478	5167128	-43604		-43604	4846874	false
	HGL 15	38611199	39231720	5423	792238	+	#80797D	620521	786815		2024565			

	HGL 9	38905373	39125319	93007432	93176203	+	#036A9C	219946	168771		-53643			
	HGL 9	39071676	39206115	93224843	93353905	+	#036A9C	134439	129062	-53643	-37075	-53643	80796	false
	HGL 9	39169040	39312188	93360313	93529498	+	#036A9C	143148	169185	-37075	-79263	-37075	106073	false
	HGL 9	39232925	39459193	93533918	93742403	+	#036A9C	226268	208485	-79263	201118	-79263	147005	false
	HGL 9	39660311	39956466	91905920	92124237	+	#036A9C	296155	218317	201118	494021	201118	497273	false
	HGL 9	40450487	41260883	92261883	93006516	-	#036A9C	810396	744633	494021	348886	494021	1304417	false
	HGL 15	41256285	41962640	5423	846984	+	#80797D	706355	841561	2024565		2024565	2730920	false
	HGL 9	41609769	41824801	93007432	93176203	+	#036A9C	215032	168771	348886	-48405	348886	563918	false
	HGL 9	41776396	41892538	93233910	93353905	+	#036A9C	116142	119995	-48405	-46973	-48405	67737	false
	HGL 9	41845565	41955234	93360313	93529319	+	#036A9C	109669	169006	-46973	-33851	-46973	62696	false
	HGL 9	41921383	42030438	93533742	93742470	+	#036A9C	109055	208728	-33851		-33851	75204	false
	HGL 10	46502443	46554066	62368365	62430020	+	#02BCB7	51623	61655		5392			
	HGL 10	46559458	47894043	81415576	83124233	+	#02BCB7	1334585	1708657	5392	-298827	5392	1339977	false
	HGL 10	47595216	47896883	80436195	80799358	+	#02BCB7	301667	363163	-298827	32907	-298827	2840	false
	HGL 10	47929790	48131414	81278851	81400605	-	#02BCB7	201624	121754	32907	176380	32907	234531	false
	HGL 10	48307794	48530845	81074854	81284235	-	#02BCB7	223051	209381	176380	17830	176380	399431	false
	HGL 10	48548675	48742436	79627437	79809832	+	#02BCB7	193761	182395	17830	-16534	17830	211591	false
	HGL 10	48725902	48783117	79857017	79920106	-	#02BCB7	57215	63089	-16534	330485	-16534	40681	false
	HGL 10	49113602	49213020	79943220	80025308	-	#02BCB7	99418	82088	330485	-42807	330485	429903	false
	HGL 10	49170213	49302853	79943214	80000964	-	#02BCB7	132640	57750	-42807	-63003	-42807	89833	false
	HGL 10	49239850	49341454	79945586	80001107	+	#02BCB7	101604	55521	-63003	-56116	-63003	38601	false
	HGL 10	49285338	49391332	79903094	80042589	-	#02BCB7	105994	139495	-56116	-69093	-56116	49878	false
	HGL 10	49322239	49405762	80021077	80085679	-	#02BCB7	83523	64602	-69093	7927	-69093	14430	false
	HGL 10	49413689	50541219	77445161	78944438	+	#02BCB7	1127530	1499277	7927	266610	7927	1135457	false
	HGL 10	50807829	51018740	78948410	79219022	+	#02BCB7	210911	270612	266610	174045	266610	477521	false
	HGL 10	51192785	51306397	83124707	83284483	+	#02BCB7	113612	159776	174045	2724	174045	287657	false
	HGL 10	51309121	51614637	79220335	79623006	+	#02BCB7	305516	402671	2724	11622	2724	308240	false
	HGL 10	51626259	53249618	75399259	77443781	-	#02BCB7	1623359	2044522	11622	31417	11622	1634981	false
	HGL 10	53281035	56608146	70905498	75339012	-	#02BCB7	3327111	4433514	31417		31417	3358528	false
**CPOR 25**	HGL 7	524448	5267659	93231101	97668547	-	#B542E4	4743211	4437446		58307			
	HGL 7	5325966	7085700	91528515	93211161	-	#B542E4	1759734	1682646	58307	141435	58307	1818041	false
	HGL 7	7227135	11601136	87191325	91504078	-	#B542E4	4374001	4312753	141435	6965	141435	4515436	false

	HGL 7	11608101	11783434	87125904	87226888	+	#B542E4	175333	100984	6965	197300	6965	182298	false
	HGL 7	11980734	12967627	86082710	87066802	-	#B542E4	986893	984092	197300	313422	197300	1184193	false
	HGL 7	13281049	13333854	85842740	85901545	-	#B542E4	52805	58805	313422	145250	313422	366227	false
	HGL 7	13479104	15082206	84365316	85901545	-	#B542E4	1603102	1536229	145250	52653	145250	1748352	false
	HGL 7	15134859	15916029	81358786	82141039	+	#B542E4	781170	782253	52653	10288	52653	833823	false
	HGL 7	15926317	16519371	82060570	82655070	+	#B542E4	593054	594500	10288	28875	10288	603342	false
	HGL 7	16548246	16616433	82737162	82813955	-	#B542E4	68187	76793	28875	363	28875	97062	false
	HGL 7	16616796	17015943	82760426	83090321	+	#B542E4	399147	329895	363	80741	363	399510	false
	HGL 7	17096684	18117857	83295890	84364726	+	#B542E4	1021173	1068836	80741	199531	80741	1101914	false
	HGL 7	18317388	18444037	81242960	81358774	-	#B542E4	126649	115814	199531	7385	199531	326180	false
	HGL 7	18451422	18544940	82647153	82738711	-	#B542E4	93518	91558	7385	164735	7385	100903	false
	HGL 7	18709675	20227691	79719816	81243637	-	#B542E4	1518016	1523821	164735	67035	164735	1682751	false
	HGL 7	20294726	22619957	77252723	79713231	-	#B542E4	2325231	2460508	67035	8550	67035	2392266	false
	HGL 7	22628507	22695834	82647153	82710097	-	#B542E4	67327	62944	8550	7037	8550	75877	false
	HGL 7	22702871	24947132	74970567	77252718	-	#B542E4	2244261	2282151	7037	72393	7037	2251298	false
	HGL 7	25019525	37624104	61799396	74970514	-	#B542E4	12604579	13171118	72393	56735	72393	12676972	false
	HGL 7	37680839	37735332	61818238	61894402	+	#B542E4	54493	76164	56735	223470	56735	111228	false
	HGL 7	37958802	42647389	56820696	61825209	-	#B542E4	4688587	5004513	223470	51155	223470	4912057	false
	HGL 7	42698544	42939344	98149828	98290999	+	#B542E4	240800	141171	51155	-51225	51155	291955	false
	HGL 7	42888119	43001205	98083167	98136652	-	#B542E4	113086	53485	-51225	-62616	-51225	61861	false
	HGL 7	42938589	43082263	98234856	98290999	+	#B542E4	143674	56143	-62616	-107991	-62616	81058	false
	HGL 7	42974272	43181164	98149122	98290999	+	#B542E4	206892	141877	-107991	-200276	-107991	98901	false
	HGL 7	42980888	43075973	98250380	98361970	+	#B542E4	95085	111590	-200276	32780	-200276	-105191	true
	HGL 7	43108753	43260958	98176683	98290999	+	#B542E4	152205	114316	32780	8606	32780	184985	false
	HGL 7	43269564	43393113	98090115	98146440	-	#B542E4	123549	56325	8606	-58258	8606	132155	false
	HGL 7	43334855	43469617	98170897	98255887	+	#B542E4	134762	84990	-58258	-17475	-58258	76504	false
	HGL 7	43452142	43517120	98258560	98361970	+	#B542E4	64978	103410	-17475	-59513	-17475	47503	false
	HGL 7	43457607	43542225	98149122	98264246	+	#B542E4	84618	115124	-59513	3297	-59513	25105	false
	HGL 7	43545522	43673093	98522115	98599450	+	#B542E4	127571	77335	3297	481297	3297	130868	false
	HGL 7	44154390	44227591	98640592	98710245	+	#B542E4	73201	69653	481297	245716	481297	554498	false
	HGL 7	44473307	52303456	98689490	106804146	+	#B542E4	7830149	8114656	245716		245716	8075865	false
**CPOR 26**	HGL 11	85341	142436	67027071	67081004	-	#7FFFFF	57095	53933		-57095			

	HGL 11	85341	145914	69939983	70006993	+	#7FFFFF	60573	67010	-57095	8951872	-57095	3478	false
	HGL 3	229598	5248612	128880347	133922172	-	#C3A652	5019014	5041825		24853			
	HGL 3	5273465	7342453	126666438	128803191	+	#C3A652	2068988	2136753	24853	53235	24853	2093841	false
	HGL 3	7395688	7513617	112354647	112518407	+	#C3A652	117929	163760	53235	37255	53235	171164	false
	HGL 3	7550872	7644681	28492340	28552262	-	#C3A652	93809	59922	37255	5565	37255	131064	false
	HGL 3	7650246	8969992	133904861	135225928	+	#C3A652	1319746	1321067	5565	4487	5565	1325311	false
	HGL 3	8974479	9037161	135225949	135286603	-	#C3A652	62682	60654	4487		4487	67169	false
	HGL 11	9097786	13700386	113062	4709298	+	#7FFFFF	4602600	4596236	8951872	56772	8951872	13554472	false
	HGL 11	13757158	13874456	4704778	4910951	+	#7FFFFF	117298	206173	56772	-56817	56772	174070	false
	HGL 11	13817639	13877386	4774121	4824653	-	#7FFFFF	59747	50532	-56817	18083	-56817	2930	false
	HGL 11	13895469	16276733	5580377	8004012	-	#7FFFFF	2381264	2423635	18083	86588	18083	2399347	false
	HGL 11	16363321	16414213	5463923	5567410	-	#7FFFFF	50892	103487	86588	-23635	86588	137480	false
	HGL 11	16390578	16455276	5289221	5346950	+	#7FFFFF	64698	57729	-23635	-64698	-23635	41063	false
	HGL 11	16390578	16455281	5331109	5410240	+	#7FFFFF	64703	79131	-64698	-64703	-64698	5	false
	HGL 11	16390578	16458949	5107474	5202620	+	#7FFFFF	68371	95146	-64703	-68371	-64703	3668	false
	HGL 11	16390578	16466837	5389063	5462871	-	#7FFFFF	76259	73808	-68371	-76259	-68371	7888	false
	HGL 11	16390578	16501722	5238223	5390489	+	#7FFFFF	111144	152266	-76259	-27895	-76259	34885	false
	HGL 11	16473827	16632091	4933693	5062418	+	#7FFFFF	158264	128725	-27895	323380	-27895	130369	false
	HGL 11	16955471	17716308	7966814	8744479	+	#7FFFFF	760837	777665	323380	4122	323380	1084217	false
	HGL 11	17720430	29863038	8807631	21734739	+	#7FFFFF	12142608	12927108	4122	1190	4122	12146730	false
	HGL 11	29864228	30557291	20987645	21716841	-	#7FFFFF	693063	729196	1190	2793	1190	694253	false
	HGL 11	30560084	30672694	21734739	21844332	-	#7FFFFF	112610	109593	2793	118981	2793	115403	false
	HGL 11	30791675	30864706	21798566	21871691	+	#7FFFFF	73031	73125	118981	6140	118981	192012	false
	HGL 11	30870846	30945930	21873350	21999629	-	#7FFFFF	75084	126279	6140	-75084	6140	81224	false
	HGL 11	30870846	32356430	22057371	23784367	+	#7FFFFF	1485584	1726996	-75084	2375	-75084	1410500	false
	HGL 11	32358805	33018217	23652808	24529434	+	#7FFFFF	659412	876626	2375	3748	2375	661787	false
	HGL 11	33021965	36607403	24588724	28784696	+	#7FFFFF	3585438	4195972	3748	-30576	3748	3589186	false
	HGL 11	36576827	46235564	28669481	39430728	+	#7FFFFF	9658737	10761247	-30576	1808	-30576	9628161	false
	HGL 11	46237372	46766594	39433148	40009599	-	#7FFFFF	529222	576451	1808	0	1808	531030	false
	HGL 11	46766594	47972662	40019795	41389969	+	#7FFFFF	1206068	1370174	0	28092	0	1206068	false
	HGL 11	48000754	48081622	41390019	41492302	-	#7FFFFF	80868	102283	28092	41	28092	108960	false
	HGL 11	48081663	48752957	41493427	42276589	+	#7FFFFF	671294	783162	41	53774	41	671335	false

	HGL 11	48806731	50944723	42335950	44717191	+	#7FFFFF	2137992	2381241	53774	-23959	53774	2191766	false
	HGL 11	50920764	57427499	44745120	52031369	+	#7FFFFF	6506735	7286249	-23959		-23959	6482776	false
**CPOR 27**	HGL 24	382	14633358	18570028	35824115	+	#FF0000	14632976	17254087		677455			
	HGL 11	14709620	14898827	65401558	65610705	+	#7FFFFF	189207	209147		0			
	HGL 11	14898827	15007259	65693911	65869210	+	#7FFFFF	108432	175299	0		0	108432	false
	HGL 24	15310813	22088013	5927226	12669605	-	#FF0000	6777200	6742379	677455	-7683	677455	7454655	false
	HGL 24	22080330	22208897	5835525	5912662	-	#FF0000	128567	77137	-7683	-27557	-7683	120884	false
	HGL 24	22181340	27870197	93879	5758426	-	#FF0000	5688857	5664547	-27557	123373	-27557	5661300	false
	HGL 24	27993570	28068761	41738	108926	-	#FF0000	75191	67188	123373		123373	198564	false
	HGL 2	28278626	28384824	126656406	126744917	+	#1CB707	106198	88511		-6298			
	HGL 2	28378526	28566149	126860693	126995564	+	#1CB707	187623	134871	-6298	63432	-6298	181325	false
	HGL 2	28629581	28824425	126958263	127172856	-	#1CB707	194844	214593	63432	4230	63432	258276	false
	HGL 2	28828655	29154904	127093667	127388167	+	#1CB707	326249	294500	4230	175773	4230	330479	false
	HGL 2	29330677	29473646	127433034	127517867	-	#1CB707	142969	84833	175773	48589	175773	318742	false
	HGL 2	29522235	30456231	124175959	125016997	-	#1CB707	933996	841038	48589	157000	48589	982585	false
	HGL 2	30613231	30700098	124115699	124204714	-	#1CB707	86867	89015	157000	-28341	157000	243867	false
	HGL 2	30671757	30774212	123967397	124102558	-	#1CB707	102455	135161	-28341	-45202	-28341	74114	false
	HGL 2	30729010	30899324	123709713	123911854	-	#1CB707	170314	202141	-45202	1459	-45202	125112	false
	HGL 2	30900783	30952048	123967178	124023527	-	#1CB707	51265	56349	1459	571	1459	52724	false
	HGL 2	30952619	31045031	123695413	123879749	-	#1CB707	92412	184336	571	-66467	571	92983	false
	HGL 2	30978564	31046225	123633424	123695413	-	#1CB707	67661	61989	-66467	23637	-66467	1194	false
	HGL 2	31069862	31127269	123967178	124041023	-	#1CB707	57407	73845	23637	4226	23637	81044	false
	HGL 2	31131495	31306859	123719299	123885162	-	#1CB707	175364	165863	4226	-111614	4226	179590	false
	HGL 2	31195245	31320304	123514447	123664993	-	#1CB707	125059	150546	-111614	-47230	-111614	13445	false
	HGL 2	31273074	31683393	123633424	123911854	-	#1CB707	410319	278430	-47230	-205085	-47230	363089	false
	HGL 2	31478308	31574957	123900531	123953329	-	#1CB707	96649	52798	-205085	-59714	-205085	-108436	true
	HGL 2	31515243	31761527	123640435	123833437	-	#1CB707	246284	193002	-59714	-76126	-59714	186570	false
	HGL 2	31685401	31809933	123658522	123727424	-	#1CB707	124532	68902	-76126	-89714	-76126	48406	false
	HGL 2	31720219	32508497	122738971	123640765	-	#1CB707	788278	901794	-89714	-694417	-89714	698564	false
	HGL 2	31814080	31865033	123697473	123760691	-	#1CB707	50953	63218	-694417	671807	-694417	-643464	true
	HGL 2	32536840	33520022	121613247	122680125	-	#1CB707	983182	1066878	671807	177080	671807	1654989	false
	HGL 2	33697102	33959040	121189174	121447553	-	#1CB707	261938	258379	177080	-47636	177080	439018	false

	HGL 2	33911404	34285065	120866572	121189103	-	#1CB707	373661	322531	-47636	-24912	-47636	326025	false
	HGL 2	34260153	34432881	120776365	120865714	-	#1CB707	172728	89349	-24912	30205	-24912	147816	false
	HGL 2	34463086	34605879	120776365	120843099	-	#1CB707	142793	66734	30205		30205	172998	false
	HGL 25	34695399	36454227	52487474	53948068	-	#4E1A96	1758828	1460594		861			
	HGL 25	36455088	44476275	6703284	15214610	-	#4E1A96	8021187	8511326	861	57311	861	8022048	false
	HGL 25	44533586	50363811	809303	6702690	-	#4E1A96	5830225	5893387	57311	58651	57311	5887536	false
	HGL 25	50422462	50621171	600417	803474	-	#4E1A96	198709	203057	58651	10164	58651	257360	false
	HGL 25	50631335	50726438	39669	145348	+	#4E1A96	95103	105679	10164	279379	10164	105267	false
	HGL 25	51005817	51431783	147920	593161	+	#4E1A96	425966	445241	279379		279379	705345	false
**CPOR 28**	HGL 13	150643	469540	8512	303495	+	#EBE47D	318897	294983		268369			
	HGL 13	737909	952112	303497	572706	-	#EBE47D	214203	269209	268369	215097	268369	482572	false
	HGL 13	1167209	1319331	574414	731725	+	#EBE47D	152122	157311	215097	81914	215097	367219	false
	HGL 13	1401245	1497167	18707963	18760741	-	#EBE47D	95922	52778	81914	-21631	81914	177836	false
	HGL 13	1475536	2120833	794361	1385290	+	#EBE47D	645297	590929	-21631	1525390	-21631	623666	false
	HGL 13	3646223	3974987	1385294	1616745	+	#EBE47D	328764	231451	1525390	-7962	1525390	1854154	false
	HGL 13	3967025	4342741	1526635	1818267	+	#EBE47D	375716	291632	-7962	43203	-7962	367754	false
	HGL 13	4385944	4470016	1764492	1815670	-	#EBE47D	84072	51178	43203	85204	43203	127275	false
	HGL 13	4555220	4712154	1818426	1973713	+	#EBE47D	156934	155287	85204	49108	85204	242138	false
	HGL 13	4761262	6962492	2034686	4281936	+	#EBE47D	2201230	2247250	49108	-10179	49108	2250338	false
	HGL 13	6952313	8930506	4270932	6464019	-	#EBE47D	1978193	2193087	-10179	-10136	-10179	1968014	false
	HGL 13	8920370	15216443	6437338	12833059	+	#EBE47D	6296073	6395721	-10136	82106	-10136	6285937	false
	HGL 13	15298549	24328028	12856720	22119098	+	#EBE47D	9029479	9262378	82106	1271	82106	9111585	false
	HGL 13	24329299	25183348	22241292	23174742	+	#EBE47D	854049	933450	1271	18506	1271	855320	false
	HGL 13	25201854	25409577	23233333	23466180	+	#EBE47D	207723	232847	18506	54304	18506	226229	false
	HGL 13	25463881	35393204	23491234	34711934	+	#EBE47D	9929323	11220700	54304	0	54304	9983627	false
	HGL 13	35393204	35668319	34810040	35178899	+	#EBE47D	275115	368859	0	-14686	0	275115	false
	HGL 13	35653633	42811108	35200289	43603576	+	#EBE47D	7157475	8403287	-14686	69137	-14686	7142789	false
	HGL 13	42880245	42957600	43775425	43927188	+	#EBE47D	77355	151763	69137	46423	69137	146492	false
	HGL 13	43004023	43225802	44004611	44250191	-	#EBE47D	221779	245580	46423	2895	46423	268202	false
	HGL 13	43228697	44676762	44236861	45888570	+	#EBE47D	1448065	1651709	2895	1132116	2895	1450960	false
	HGL 13	45808878	48398397	45890413	48833388	-	#EBE47D	2589519	2942975	1132116		1132116	3721635	false
	HGL 3	48463414	48524434	27681127	27753039	-	#C3A652	61020	71912		6182			

	HGL 3	48530616	48758319	27753039	27985173	+	#C3A652	227703	232134	6182		6182	233885	false
**CPOR 29**	HGL 28	29255	178576	46056363	46252118	-	#007FFF	149321	195755		18102			
	HGL 28	196678	375777	45826371	45980772	+	#007FFF	179099	154401	18102	20494	18102	197201	false
	HGL 28	396271	4662376	41186321	45852605	+	#007FFF	4266105	4666284	20494	429	20494	4286599	false
	HGL 28	4662805	4842162	41068578	41281098	-	#007FFF	179357	212520	429	98215	429	179786	false
	HGL 28	4940377	5212997	40704907	40979152	-	#007FFF	272620	274245	98215	55155	98215	370835	false
	HGL 28	5268152	8019718	37942434	40704620	-	#007FFF	2751566	2762186	55155	79767	55155	2806721	false
	HGL 28	8099485	20579906	25112285	37941916	-	#007FFF	12480421	12829631	79767	58711	79767	12560188	false
	HGL 28	20638617	34378049	10702544	25091243	-	#007FFF	13739432	14388699	58711	-60149	58711	13798143	false
	HGL 28	34317900	34572481	8598348	8682560	+	#007FFF	254581	84212	-60149	-145822	-60149	194432	false
	HGL 28	34426659	34567838	8549951	8612949	+	#007FFF	141179	62998	-145822	-125722	-145822	-4643	true
	HGL 28	34442116	41772607	3542945	10749253	-	#007FFF	7330491	7206308	-125722	418291	-125722	7204769	false
	HGL 28	42190898	42634533	3034658	3454301	-	#007FFF	443635	419643	418291	338769	418291	861926	false
	HGL 28	42973302	45857281	16484	2991045	-	#007FFF	2883979	2974561	338769		338769	3222748	false
**CPOR 3**	HGL 26	37652	374103	16906098	17251911	-	#64E09A	336451	345813		107785			
	HGL 26	481888	3015569	21824917	24383970	-	#64E09A	2533681	2559053	107785		107785	2641466	false
	HGL 15	3251537	3361699	44827341	44892661	+	#80797D	110162	65320		92495			
	HGL 15	3454194	4658243	44905726	46321914	+	#80797D	1204049	1416188	92495	48857	92495	1296544	false
	HGL 15	4707100	33412199	46444698	77939228	+	#80797D	28705099	31494530	48857	122155	48857	28753956	false
	HGL 15	33534354	35675591	77946000	80164261	+	#80797D	2141237	2218261	122155	33723	122155	2263392	false
	HGL 15	35709314	38086393	80260497	82792342	+	#80797D	2377079	2531845	33723	298	33723	2410802	false
	HGL 15	38086691	38164515	82792342	82852107	-	#80797D	77824	59765	298	682	298	78122	false
	HGL 15	38165197	48015453	82852107	93261865	+	#80797D	9850256	10409758	682		682	9850938	false
	HGL 7	48218368	60266580	30442803	43017579	+	#B542E4	12048212	12574776		171773			
	HGL 7	60438353	61827707	43073744	44474356	+	#B542E4	1389354	1400612	171773	64476	171773	1561127	false
	HGL 7	61892183	61950241	44777967	44831307	-	#B542E4	58058	53340	64476	478840	64476	122534	false
	HGL 7	62429081	62580846	44742073	44831936	-	#B542E4	151765	89863	478840	385619	478840	630605	false
	HGL 7	62966465	63081285	44583853	44637301	+	#B542E4	114820	53448	385619	-80292	385619	500439	false
	HGL 7	63000993	63086887	44681830	44750670	+	#B542E4	85894	68840	-80292	-23504	-80292	5602	false
	HGL 7	63063383	63263523	44778078	44945946	+	#B542E4	200140	167868	-23504	8121	-23504	176636	false
	HGL 7	63271644	73037175	44821123	55355362	+	#B542E4	9765531	10534239	8121	-9199478	8121	9773652	false
	HGL 7	63837697	63905264	45423481	45494384	+	#B542E4	67567	70903	-9199478	9186945	-9199478	-9131911	true

	HGL 7	73092209	73922764	55428719	56358806	+	#B542E4	830555	930087	9186945	76419	9186945	10017500	false
	HGL 7	73999183	74312644	56379753	56749025	-	#B542E4	313461	369272	76419	130447	76419	389880	false
	HGL 7	74443091	74613978	30233216	30354962	-	#B542E4	170887	121746	130447	-142686	130447	301334	false
	HGL 7	74471292	74615247	30263247	30399458	-	#B542E4	143955	136211	-142686	-89987	-142686	1269	false
	HGL 7	74525260	74679448	30263247	30399461	-	#B542E4	154188	136214	-89987	-89788	-89987	64201	false
	HGL 7	74589660	74855942	30254142	30391452	-	#B542E4	266282	137310	-89788	-151298	-89788	176494	false
	HGL 7	74704644	74845631	30327742	30399461	-	#B542E4	140987	71719	-151298	61381	-151298	-10311	true
	HGL 7	74907012	80823651	24147966	30245060	-	#B542E4	5916639	6097094	61381	52508	61381	5978020	false
	HGL 7	80876159	81596501	23395302	24109839	-	#B542E4	720342	714537	52508	31825	52508	772850	false
	HGL 7	81628326	82978078	21877542	23340633	-	#B542E4	1349752	1463091	31825	328	31825	1381577	false
	HGL 7	82978406	83588957	23842526	24458776	+	#B542E4	610551	616250	328	0	328	610879	false
	HGL 7	83588957	85331023	21445974	23340633	+	#B542E4	1742066	1894659	0	-26868	0	1742066	false
	HGL 7	85304155	85684683	23340778	23788051	+	#B542E4	380528	447273	-26868	410	-26868	353660	false
	HGL 7	85685093	86308802	23848056	24458775	+	#B542E4	623709	610719	410	0	410	624119	false
	HGL 7	86308802	87423546	21445973	22483516	+	#B542E4	1114744	1037543	0	4151	0	1114744	false
	HGL 7	87427697	87638724	23340773	23539408	-	#B542E4	211027	198635	4151	-26265	4151	215178	false
	HGL 7	87612459	91376033	19461521	23340633	-	#B542E4	3763574	3879112	-26265	-124929	-26265	3737309	false
	HGL 7	91251104	99587245	11136603	19494716	-	#B542E4	8336141	8358113	-124929	60059	-124929	8211212	false
	HGL 7	99647304	100349082	10363937	11134220	-	#B542E4	701778	770283	60059	2616	60059	761837	false
	HGL 7	100351698	100933917	9740670	10390467	+	#B542E4	582219	649797	2616	1	2616	584835	false
	HGL 7	100933918	101100604	9740670	10001099	+	#B542E4	166686	260429	1	413	1	166687	false
	HGL 7	101101017	101373644	10118091	10389922	+	#B542E4	272627	271831	413	0	413	273040	false
	HGL 7	101373644	101952928	9742826	10390467	+	#B542E4	579284	647641	0	1	0	579284	false
	HGL 7	101952929	102538937	9740670	10364834	+	#B542E4	586008	624164	1	0	1	586009	false
	HGL 7	102538937	102874414	10025867	10387625	-	#B542E4	335477	361758	0	69725	0	335477	false
	HGL 7	102944139	107196308	5626311	10005725	-	#B542E4	4252169	4379414	69725	-100707	69725	4321894	false
	HGL 7	107095601	109267726	3283358	5636514	+	#B542E4	2172125	2353156	-100707	-16842	-100707	2071418	false
	HGL 7	109250884	111928801	335797	3299754	-	#B542E4	2677917	2963957	-16842	50374	-16842	2661075	false
	HGL 7	111979175	112335634	21547	335793	-	#B542E4	356459	314246	50374		50374	406833	false
**CPOR 30**	HGL 12	100	1450001	15278582	16749708	+	#F33071	1449901	1471126		42559			
	HGL 12	1492560	10608802	16847120	26595937	+	#F33071	9116242	9748817	42559	2371	42559	9158801	false
	HGL 12	10611173	12605727	26646280	28850078	+	#F33071	1994554	2203798	2371	35196	2371	1996925	false

	HGL 12	12640923	12778920	28910782	29050367	+	#F33071	137997	139585	35196	420	35196	173193	false
	HGL 12	12779340	12970834	29058277	29298537	-	#F33071	191494	240260	420	619	420	191914	false
	HGL 12	12971453	14648279	29303050	31197078	+	#F33071	1676826	1894028	619	-61681	619	1677445	false
	HGL 12	14586598	17816804	31243811	34557779	+	#F33071	3230206	3313968	-61681	0	-61681	3168525	false
	HGL 12	17816804	30981486	34614415	49354179	+	#F33071	13164682	14739764	0	33	0	13164682	false
	HGL 12	30981519	36151272	49429626	55369163	+	#F33071	5169753	5939537	33	76636	33	5169786	false
	HGL 12	36227908	37449653	55369171	56752876	+	#F33071	1221745	1383705	76636	43776	76636	1298381	false
	HGL 12	37493429	37998602	56822966	57480958	+	#F33071	505173	657992	43776	3331	43776	548949	false
	HGL 12	38001933	38100758	57475846	57577886	-	#F33071	98825	102040	3331	47	3331	102156	false
	HGL 12	38100805	40088749	57580539	60088063	+	#F33071	1987944	2507524	47	12841	47	1987991	false
	HGL 12	40101590	40188659	60153839	60228138	+	#F33071	87069	74299	12841	624730	12841	99910	false
	HGL 12	40813389	40887081	60563535	60624440	+	#F33071	73692	60905	624730	347945	624730	698422	false
	HGL 12	41235026	41303720	61687432	61738295	+	#F33071	68694	50863	347945	38531	347945	416639	false
	HGL 12	41342251	41498266	61685406	61773704	+	#F33071	156015	88298	38531	27128	38531	194546	false
	HGL 12	41525394	41834994	61896415	62275084	+	#F33071	309600	378669	27128	-8663	27128	336728	false
	HGL 12	41826331	42004561	62350180	62614907	+	#F33071	178230	264727	-8663	-13092	-8663	169567	false
	HGL 12	41991469	43041290	62620710	63877833	+	#F33071	1049821	1257123	-13092	10361	-13092	1036729	false
	HGL 12	43051651	44283631	63932898	65370559	+	#F33071	1231980	1437661	10361	77633	10361	1242341	false
	HGL 12	44361264	44644930	65579647	65856189	+	#F33071	283666	276542	77633	69265	77633	361299	false
	HGL 12	44714195	45014456	65973252	66302879	+	#F33071	300261	329627	69265		69265	369526	false
**CPOR 31**	HGL 25	7	313317	29386077	29773827	-	#4E1A96	313310	387750		-29379			
	HGL 25	283938	2330578	27034630	29382359	-	#4E1A96	2046640	2347729	-29379	108350	-29379	2017261	false
	HGL 25	2438928	2987577	24332529	24907046	+	#4E1A96	548649	574517	108350	6065	108350	656999	false
	HGL 25	2993642	4895725	24958342	27034228	+	#4E1A96	1902083	2075886	6065	74049	6065	1908148	false
	HGL 25	4969774	5142274	24086026	24329734	-	#4E1A96	172500	243708	74049	71130	74049	246549	false
	HGL 25	5213404	11841431	17131960	24081696	-	#4E1A96	6628027	6949736	71130	2228	71130	6699157	false
	HGL 25	11843659	13485511	15216091	17051645	-	#4E1A96	1641852	1835554	2228	8605	2228	1644080	false
	HGL 25	13494116	18673864	47782110	52487474	-	#4E1A96	5179748	4705364	8605	69185	8605	5188353	false
	HGL 25	18743049	27267423	39076511	47739859	-	#4E1A96	8524374	8663348	69185	55825	69185	8593559	false
	HGL 25	27323248	35772809	29797033	39020799	-	#4E1A96	8449561	9223766	55825		55825	8505386	false
	HGL 11	35778650	35896363	51985295	52151031	+	#7FFFFF	117713	165736		177023			
	HGL 11	36073386	41261458	52167759	58076259	+	#7FFFFF	5188072	5908500	177023	0	177023	5365095	false

	HGL 11	41261458	41346824	58087022	58176101	-	#7FFFFF	85366	89079	0	3	0	85366	false
	HGL 11	41346827	43919796	58188358	61199428	+	#7FFFFF	2572969	3011070	3	92989	3	2572972	false
	HGL 11	44012785	44500058	61265234	61784789	+	#7FFFFF	487273	519555	92989	220879	92989	580262	false
	HGL 11	44720937	47078846	61848221	64528523	+	#7FFFFF	2357909	2680302	220879	60769	220879	2578788	false
	HGL 11	47139615	47570975	64556945	65038287	+	#7FFFFF	431360	481342	60769	52395	60769	492129	false
	HGL 11	47623370	47813161	65064796	65289233	+	#7FFFFF	189791	224437	52395		52395	242186	false
**CPOR 4**	HGL 9	724175	1088968	66342245	66732423	+	#036A9C	364793	390178		27127			
	HGL 9	1116095	1702777	66787247	67475606	+	#036A9C	586682	688359	27127	11188	27127	613809	false
	HGL 9	1713965	4708664	88770134	91888718	-	#036A9C	2994699	3118584	11188	460006	11188	3005887	false
	HGL 9	5168670	25641953	67589539	88736298	-	#036A9C	20473283	21146759	460006	23265934	460006	20933289	false
	HGL 2	25691209	26115504	139294369	139847614	+	#1CB707	424295	553245	25282535	70761	25282535	25706830	false
	HGL 2	26186265	36981094	127745768	139047718	-	#1CB707	10794829	11301950	70761	-1302	70761	10865590	false
	HGL 2	36979792	37090048	124916784	125001930	+	#1CB707	110256	85146	-1302	365008	-1302	108954	false
	HGL 2	37455056	37660633	125428860	125695513	+	#1CB707	205577	266653	365008	638	365008	570585	false
	HGL 2	37661271	37742024	125757935	125826481	+	#1CB707	80753	68546	638	-15892	638	81391	false
	HGL 2	37726132	37796299	125863310	125968010	+	#1CB707	70167	104700	-15892	33460680	-15892	54275	false
	HGL 20	38010038	39515458	60811726	62126870	-	#00FFFF	1505420	1315144		0			
	HGL 20	39515458	40090108	62136048	62640601	+	#00FFFF	574650	504553	0	2037	0	574650	false
	HGL 20	40092145	40227416	62630238	62728992	-	#00FFFF	135271	98754	2037	8160	2037	137308	false
	HGL 20	40235576	41204935	62682758	63510366	+	#00FFFF	969359	827608	8160		8160	977519	false
	HGL 24	41491042	41595709	35939705	36080260	-	#FF0000	104667	140555		52303			
	HGL 24	41648012	42135275	36170855	36627140	+	#FF0000	487263	456285	52303	90968	52303	539566	false
	HGL 24	42226243	48797428	36634733	43166937	+	#FF0000	6571185	6532204	90968	-143893	90968	6662153	false
	HGL 24	48653535	48991320	43171859	43480707	+	#FF0000	337785	308848	-143893		-143893	193892	false
	HGL 22	49093723	50390747	54693558	55560912	+	#EC83BA	1297024	867354		-521021			
	HGL 22	49869726	49935888	55226010	55310233	-	#EC83BA	66162	84223	-521021	54537	-521021	-454859	true
	HGL 22	49990425	50072123	55082487	55161765	-	#EC83BA	81698	79278	54537	6867	54537	136235	false
	HGL 22	50078990	50181822	55045088	55148355	-	#EC83BA	102832	103267	6867	134731	6867	109699	false
	HGL 22	50316553	50702266	55615990	55986441	+	#EC83BA	385713	370451	134731	46779	134731	520444	false
	HGL 22	50749045	51229435	55976203	56332259	+	#EC83BA	480390	356056	46779	271558	46779	527169	false
	HGL 22	51500993	55403389	56334593	60552044	+	#EC83BA	3902396	4217451	271558	14672	271558	4173954	false
	HGL 22	55418061	55689766	60623276	60891998	+	#EC83BA	271705	268722	14672	72760	14672	286377	false

	HGL 22	55762526	59735858	60919142	65018153	+	#EC83BA	3973332	4099011	72760	102369	72760	4046092	false
	HGL 18	59736023	59791576	13376879	13473015	-	#0000FF	55553	96136		2492399			
	HGL 22	59838227	61820492	65022023	67196853	+	#EC83BA	1982265	2174830	102369		102369	2084634	false
	HGL 18	62283975	65255043	13555221	16484382	+	#0000FF	2971068	2929161	2492399	0	2492399	5463467	false
	HGL 18	65255043	66586907	16553037	17898398	+	#0000FF	1331864	1345361	0	214769	0	1331864	false
	HGL 18	66801676	67355229	17951438	18356352	+	#0000FF	553553	404914	214769	131090	214769	768322	false
	HGL 18	67486319	70387507	18356352	21316843	+	#0000FF	2901188	2960491	131090	2509	131090	3032278	false
	HGL 18	70390016	70960129	21442027	22038365	+	#0000FF	570113	596338	2509	1124639	2509	572622	false
	HGL 2	71256979	71362134	126325044	126465327	-	#1CB707	105155	140283	33460680	91956	33460680	33565835	false
	HGL 2	71454090	71554902	125939635	126000281	-	#1CB707	100812	60646	91956	826	91956	192768	false
	HGL 2	71555728	71859946	125435918	125791488	-	#1CB707	304218	355570	826	11280	826	305044	false
	HGL 2	71871226	71929529	125101888	125166750	-	#1CB707	58303	64862	11280		11280	69583	false
	HGL 18	72084768	74487481	28433487	30934925	-	#0000FF	2402713	2501438	1124639	253623	1124639	3527352	false
	HGL 18	74741104	75049285	28061384	28346268	-	#0000FF	308181	284884	253623	3450	253623	561804	false
	HGL 18	75052735	75112675	13599268	13694545	+	#0000FF	59940	95277	3450	132765	3450	63390	false
	HGL 18	75245440	77893322	25263616	28059551	-	#0000FF	2647882	2795935	132765	120269	132765	2780647	false
	HGL 18	78013591	80272190	22919069	25260454	-	#0000FF	2258599	2341385	120269	85241	120269	2378868	false
	HGL 18	80357431	80486806	22882832	22934751	-	#0000FF	129375	51919	85241	155695	85241	214616	false
	HGL 18	80642501	81007594	22451418	22810987	-	#0000FF	365093	359569	155695	0	155695	520788	false
	HGL 18	81007594	81080132	22316549	22390278	-	#0000FF	72538	73729	0	7859	0	72538	false
	HGL 18	81087991	81315374	22011070	22259147	-	#0000FF	227383	248077	7859	323208	7859	235242	false
	HGL 18	81638582	82090589	13005865	13426211	-	#0000FF	452007	420346	323208	76234	323208	775215	false
	HGL 18	82166823	83216287	11931631	12963527	-	#0000FF	1049464	1031896	76234	162948	76234	1125698	false
	HGL 18	83379235	83467540	11931631	11988008	+	#0000FF	88305	56377	162948	0	162948	251253	false
	HGL 18	83467540	88960868	6035370	11955044	-	#0000FF	5493328	5919674	0	129242	0	5493328	false
	HGL 18	89090110	90608767	4459064	6035123	-	#0000FF	1518657	1576059	129242	59734	129242	1647899	false
	HGL 18	90668501	92771598	2319405	4457571	-	#0000FF	2103097	2138166	59734	82023	59734	2162831	false
	HGL 18	92853621	93857558	1322642	2313477	-	#0000FF	1003937	990835	82023	222796	82023	1085960	false
	HGL 18	94080354	95350692	33805	1300820	-	#0000FF	1270338	1267015	222796		222796	1493134	false
	HGL 8	95587122	96341110	111607147	112413322	-	#00FF7F	753988	806175		79235			
	HGL 8	96420345	103695223	104078146	111634913	-	#00FF7F	7274878	7556767	79235	64549	79235	7354113	false
	HGL 8	103759772	104768829	123843312	124925466	+	#00FF7F	1009057	1082154	64549		64549	1073606	false

**CPOR 5**	HGL 8	49797	751757	112414290	113086009	+	#00FF7F	701960	671719		182589			
	HGL 8	934346	2467263	113175289	114799613	+	#00FF7F	1532917	1624324	182589	22756	182589	1715506	false
	HGL 8	2490019	2914354	114859660	115293260	+	#00FF7F	424335	433600	22756	3313	22756	447091	false
	HGL 8	2917667	3162965	114538774	114808750	+	#00FF7F	245298	269976	3313	16624	3313	248611	false
	HGL 8	3179589	3526792	114859660	115195772	+	#00FF7F	347203	336112	16624	0	16624	363827	false
	HGL 8	3526792	3984968	115427253	115918166	-	#00FF7F	458176	490913	0	57545	0	458176	false
	HGL 8	4042513	4259005	114538774	114808750	-	#00FF7F	216492	269976	57545	2522	57545	274037	false
	HGL 8	4261527	4730820	114859660	115293489	-	#00FF7F	469293	433829	2522	18341	2522	471815	false
	HGL 8	4749161	4987532	114538774	114801228	-	#00FF7F	238371	262454	18341	3699	18341	256712	false
	HGL 8	4991231	5046535	115201462	115293260	-	#00FF7F	55304	91798	3699	90947	3699	59003	false
	HGL 8	5137482	5336235	114859660	115105005	-	#00FF7F	198753	245345	90947	15948	90947	289700	false
	HGL 8	5352183	5545194	114538774	114808750	-	#00FF7F	193011	269976	15948	25044	15948	208959	false
	HGL 8	5570238	5645977	114717726	114808196	+	#00FF7F	75739	90470	25044	15402	25044	100783	false
	HGL 8	5661379	5809223	114859660	114980237	+	#00FF7F	147844	120577	15402	19335	15402	163246	false
	HGL 8	5828558	6347908	114931047	115455141	+	#00FF7F	519350	524094	19335	3094	19335	538685	false
	HGL 8	6351002	6445626	115107867	115199260	+	#00FF7F	94624	91393	3094	18191	3094	97718	false
	HGL 8	6463817	8545387	115298676	117391960	+	#00FF7F	2081570	2093284	18191	8045	18191	2099761	false
	HGL 8	8553432	8627443	117396359	117466322	-	#00FF7F	74011	69963	8045	-62864	8045	82056	false
	HGL 8	8564579	9582810	117446085	118412511	+	#00FF7F	1018231	966426	-62864	0	-62864	955367	false
	HGL 8	9582810	10638946	117687381	118737617	-	#00FF7F	1056136	1050236	0	846	0	1056136	false
	HGL 8	10639792	10758508	118738216	118859033	-	#00FF7F	118716	120817	846	0	846	119562	false
	HGL 8	10758508	11208531	118412691	118861674	+	#00FF7F	450023	448983	0	741	0	450023	false
	HGL 8	11209272	17157488	117687381	123857878	+	#00FF7F	5948216	6170497	741	619708	741	5948957	false
	HGL 22	17385069	17466597	10742301	10812490	+	#EC83BA	81528	70189		158646			
	HGL 22	17625243	17695103	11536359	11609544	+	#EC83BA	69860	73185	158646		158646	228506	false
	HGL 8	17777196	18973732	102833614	104078146	-	#00FF7F	1196536	1244532	619708	7171	619708	1816244	false
	HGL 8	18980903	20248798	101608310	102768252	-	#00FF7F	1267895	1159942	7171	-1050468	7171	1275066	false
	HGL 8	19198330	19457859	102517011	102586828	-	#00FF7F	259529	69817	-1050468	795576	-1050468	-790939	true
	HGL 8	20253435	20474365	101574082	101741461	+	#00FF7F	220930	167379	795576	0	795576	1016506	false
	HGL 8	20474365	20719573	101441775	101638528	-	#00FF7F	245208	196753	0	274960	0	245208	false
	HGL 8	20994533	21163437	101260936	101405259	+	#00FF7F	168904	144323	274960	2779	274960	443864	false
	HGL 8	21166216	22421147	100174175	101311748	-	#00FF7F	1254931	1137573	2779	57037	2779	1257710	false

	HGL 8	22478184	22643968	100089665	100228920	-	#00FF7F	165784	139255	57037	165452	57037	222821	false
	HGL 8	22809420	22877560	100163054	100269375	-	#00FF7F	68140	106321	165452	53800	165452	233592	false
	HGL 8	22931360	22998566	100090726	100155678	-	#00FF7F	67206	64952	53800	10138	53800	121006	false
	HGL 8	23008704	23355509	100075148	100364698	-	#00FF7F	346805	289550	10138	195351	10138	356943	false
	HGL 8	23550860	24225272	99372479	100008151	-	#00FF7F	674412	635672	195351	78228	195351	869763	false
	HGL 8	24303500	24367192	99261626	99314413	-	#00FF7F	63692	52787	78228	40015	78228	141920	false
	HGL 8	24407207	24486619	99315131	99475572	+	#00FF7F	79412	160441	40015	73060	40015	119427	false
	HGL 8	24559679	24655067	99078989	99257418	+	#00FF7F	95388	178429	73060	57038	73060	168448	false
	HGL 8	24712105	24796373	98934064	99053210	-	#00FF7F	84268	119146	57038	100434	57038	141306	false
	HGL 8	24896807	24996684	99427438	99536192	-	#00FF7F	99877	108754	100434	17452	100434	200311	false
	HGL 8	25014136	25085939	99261354	99329364	-	#00FF7F	71803	68010	17452	142709	17452	89255	false
	HGL 8	25228648	25304021	99262620	99337937	-	#00FF7F	75373	75317	142709	217213	142709	218082	false
	HGL 8	25521234	25739187	99261354	99488956	-	#00FF7F	217953	227602	217213	22526	217213	435166	false
	HGL 8	25761713	25821889	99262620	99324707	+	#00FF7F	60176	62087	22526	136454	22526	82702	false
	HGL 8	25958343	26010526	99173938	99257418	+	#00FF7F	52183	83480	136454	9894	136454	188637	false
	HGL 8	26020420	26092819	99075405	99145235	-	#00FF7F	72399	69830	9894	28114	9894	82293	false
	HGL 8	26120933	26191683	99427438	99537983	-	#00FF7F	70750	110545	28114	46037	28114	98864	false
	HGL 8	26237720	26586454	99261626	99623506	+	#00FF7F	348734	361880	46037	32173	46037	394771	false
	HGL 8	26618627	26906718	99320511	99623506	+	#00FF7F	288091	302995	32173	5697	32173	320264	false
	HGL 8	26912415	27012774	99288014	99351680	+	#00FF7F	100359	63666	5697	12255	5697	106056	false
	HGL 8	27025029	27286421	98890742	99169346	+	#00FF7F	261392	278604	12255	5486	12255	273647	false
	HGL 8	27291907	27344346	99173938	99257418	-	#00FF7F	52439	83480	5486	3969	5486	57925	false
	HGL 8	27348315	27412823	99185615	99257418	+	#00FF7F	64508	71803	3969	117331	3969	68477	false
	HGL 8	27530154	27640416	99016263	99087524	+	#00FF7F	110262	71261	117331	1938	117331	227593	false
	HGL 8	27642354	28156391	98577767	99017325	-	#00FF7F	514037	439558	1938	106511	1938	515975	false
	HGL 8	28262902	29362086	97501242	98575047	-	#00FF7F	1099184	1073805	106511	8445	106511	1205695	false
	HGL 8	29370531	32373416	94423564	97403939	-	#00FF7F	3002885	2980375	8445	212492	8445	3011330	false
	HGL 8	32585908	33709229	93271593	94429804	-	#00FF7F	1123321	1158211	212492	-42711	212492	1335813	false
	HGL 8	33666518	33741428	93178923	93242629	-	#00FF7F	74910	63706	-42711	116947	-42711	32199	false
	HGL 8	33858375	33952032	93073345	93139934	-	#00FF7F	93657	66589	116947	451592	116947	210604	false
	HGL 8	34403624	34559334	93177751	93319018	+	#00FF7F	155710	141267	451592	251526	451592	607302	false
	HGL 8	34810860	34867439	93138242	93209149	+	#00FF7F	56579	70907	251526	122467	251526	308105	false

	HGL 8	34989906	35155117	92953477	93121220	-	#00FF7F	165211	167743	122467	-28888	122467	287678	false
	HGL 8	35126229	40263619	87456788	92945719	-	#00FF7F	5137390	5488931	-28888	109	-28888	5108502	false
	HGL 8	40263728	40394428	87208501	87350334	-	#00FF7F	130700	141833	109	54610	109	130809	false
	HGL 8	40449038	42905529	84471147	87208501	-	#00FF7F	2456491	2737354	54610	54760	54610	2511101	false
	HGL 8	42960289	46722066	80423297	84446214	-	#00FF7F	3761777	4022917	54760	39084	54760	3816537	false
	HGL 8	46761150	51205999	75662706	80368720	-	#00FF7F	4444849	4706014	39084	365291	39084	4483933	false
	HGL 8	51571290	54458947	72186164	75625157	-	#00FF7F	2887657	3438993	365291	0	365291	3252948	false
	HGL 8	54458947	54619881	71974559	72186163	+	#00FF7F	160934	211604	0	0	0	160934	false
	HGL 8	54619881	60235932	65819362	71974544	-	#00FF7F	5616051	6155182	0	25969	0	5616051	false
	HGL 8	60261901	60357659	65778292	65901960	+	#00FF7F	95758	123668	25969	195	25969	121727	false
	HGL 8	60357854	61542438	64549197	65769798	-	#00FF7F	1184584	1220601	195	27343	195	1184779	false
	HGL 8	61569781	62281512	63424175	64348172	+	#00FF7F	711731	923997	27343	84462	27343	739074	false
	HGL 8	62365974	62610940	64348428	64549197	-	#00FF7F	244966	200769	84462	308578	84462	329428	false
	HGL 8	62919518	69764854	33798859	41453642	+	#00FF7F	6845336	7654783	308578	35922	308578	7153914	false
	HGL 8	69800776	71586203	41509026	43449392	+	#00FF7F	1785427	1940366	35922	266070	35922	1821349	false
	HGL 8	71852273	72885145	48775014	49867478	-	#00FF7F	1032872	1092464	266070	109481	266070	1298942	false
	HGL 8	72994626	73262949	48267552	48594642	-	#00FF7F	268323	327090	109481	23510	109481	377804	false
	HGL 8	73286459	73357700	47942912	48017571	-	#00FF7F	71241	74659	23510	80640	23510	94751	false
	HGL 8	73438340	73577203	47864608	48017566	-	#00FF7F	138863	152958	80640	-70803	80640	219503	false
	HGL 8	73506400	73626173	47529921	47640581	-	#00FF7F	119773	110660	-70803	12433	-70803	48970	false
	HGL 8	73638606	73735581	48040015	48098159	+	#00FF7F	96975	58144	12433	-95497	12433	109408	false
	HGL 8	73640084	73734283	48204791	48280509	+	#00FF7F	94199	75718	-95497	11883	-95497	-1298	true
	HGL 8	73746166	73856685	47783808	47914900	-	#00FF7F	110519	131092	11883	-37753	11883	122402	false
	HGL 8	73818932	73925788	47476454	47530420	-	#00FF7F	106856	53966	-37753	-106853	-37753	69103	false
	HGL 8	73818935	73927243	47344500	47416581	-	#00FF7F	108308	72081	-106853	195736	-106853	1455	false
	HGL 8	74122979	74220426	47937423	48017566	-	#00FF7F	97447	80143	195736	6073	195736	293183	false
	HGL 8	74226499	74281147	47476454	47613547	-	#00FF7F	54648	137093	6073	32286	6073	60721	false
	HGL 8	74313433	74392101	48231068	48317459	+	#00FF7F	78668	86391	32286	23885	32286	110954	false
	HGL 8	74415986	74566199	47064757	47194762	+	#00FF7F	150213	130005	23885	12063	23885	174098	false
	HGL 8	74578262	74740463	47245111	47441129	+	#00FF7F	162201	196018	12063	-97011	12063	174264	false
	HGL 8	74643452	74928162	47452701	47686410	+	#00FF7F	284710	233709	-97011	-160484	-97011	187699	false
	HGL 8	74767678	74984951	47865596	48017571	+	#00FF7F	217273	151975	-160484	-104949	-160484	56789	false

	HGL 8	74880002	74980789	48104161	48163826	+	#00FF7F	100787	59665	-104949	18764	-104949	-4162	true
	HGL 8	74999553	75093297	47547559	47640581	+	#00FF7F	93744	93022	18764	-75743	18764	112508	false
	HGL 8	75017554	75245038	47864703	48047427	+	#00FF7F	227484	182724	-75743	-132093	-75743	151741	false
	HGL 8	75112945	75281187	48137815	48206970	+	#00FF7F	168242	69155	-132093	-146067	-132093	36149	false
	HGL 8	75135120	75246152	48246224	48361848	-	#00FF7F	111032	115624	-146067	-54971	-146067	-35035	true
	HGL 8	75191181	75281187	47983152	48043176	+	#00FF7F	90006	60024	-54971	58958	-54971	35035	false
	HGL 8	75340145	75512766	47588084	47863810	+	#00FF7F	172621	275726	58958	-148543	58958	231579	false
	HGL 8	75364223	75512608	47471570	47640581	+	#00FF7F	148385	169011	-148543	-59291	-148543	-158	true
	HGL 8	75453317	75601962	47865595	48047427	+	#00FF7F	148645	181832	-59291	75586	-59291	89354	false
	HGL 8	75677548	75744443	47194762	47297901	-	#00FF7F	66895	103139	75586	-14815	75586	142481	false
	HGL 8	75729628	79006181	43482882	47194762	-	#00FF7F	3276553	3711880	-14815	61153	-14815	3261738	false
	HGL 8	79067334	84542700	49871700	55702290	+	#00FF7F	5475366	5830590	61153	-4269	61153	5536519	false
	HGL 8	84538431	84670173	55617549	55756560	-	#00FF7F	131742	139011	-4269	16165	-4269	127473	false
	HGL 8	84686338	91323700	55756644	63421931	+	#00FF7F	6637362	7665287	16165		16165	6653527	false
**CPOR 6**	HGL 6	315556	568704	78599078	78865801	+	#980157	253148	266723		5486			
	HGL 6	574190	658571	78929479	79054510	-	#980157	84381	125031	5486	1743	5486	89867	false
	HGL 6	660314	3199632	79138949	82080936	+	#980157	2539318	2941987	1743	11473	1743	2541061	false
	HGL 6	3211105	3380093	82135055	82323026	+	#980157	168988	187971	11473	1830	11473	180461	false
	HGL 6	3381923	5296173	82381579	84540727	+	#980157	1914250	2159148	1830	6598	1830	1916080	false
	HGL 6	5302771	5613944	86549171	86929475	-	#980157	311173	380304	6598	0	6598	317771	false
	HGL 6	5613944	6848787	84897943	86378941	-	#980157	1234843	1480998	0	-11331	0	1234843	false
	HGL 6	6837456	7089514	84592846	84872806	-	#980157	252058	279960	-11331	493056	-11331	240727	false
	HGL 22	7171399	7319456	40025486	40195854	-	#EC83BA	148057	170368		4689			
	HGL 22	7324145	7500538	8229595	8467208	-	#EC83BA	176393	237613	4689	-32034	4689	181082	false
	HGL 22	7468504	7549091	8154237	8215634	-	#EC83BA	80587	61397	-32034	40606694	-32034	48553	false
	HGL 6	7582570	11399342	33780949	37784365	+	#980157	3816772	4003416	493056	51748	493056	4309828	false
	HGL 6	11451090	14609913	37843606	41598797	+	#980157	3158823	3755191	51748	2905	51748	3210571	false
	HGL 6	14612818	14703087	41598866	41706270	-	#980157	90269	107404	2905	0	2905	93174	false
	HGL 6	14703087	21916583	41708451	49442433	+	#980157	7213496	7733982	0	3063	0	7213496	false
	HGL 6	21919646	21999032	49433558	49527579	-	#980157	79386	94021	3063	22999	3063	82449	false
	HGL 6	22022031	22085787	49426131	49512983	+	#980157	63756	86852	22999	60156	22999	86755	false
	HGL 6	22145943	22265396	49513213	49606243	+	#980157	119453	93030	60156	-22680	60156	179609	false

	HGL 6	22242716	24068584	49688298	51736434	+	#980157	1825868	2048136	-22680	583861	-22680	1803188	false
	HGL 6	24652445	28151950	51730754	55704986	+	#980157	3499505	3974232	583861	-200005	583861	4083366	false
	HGL 6	27951945	28007675	55506327	55560386	+	#980157	55730	54059	-200005	132341	-200005	-144275	true
	HGL 6	28140016	41627552	55728025	70509260	+	#980157	13487536	14781235	132341	58019	132341	13619877	false
	HGL 6	41685571	44407721	70560483	73282211	+	#980157	2722150	2721728	58019	-36464	58019	2780169	false
	HGL 6	44371257	45271456	73283488	74236542	+	#980157	900199	953054	-36464	196724	-36464	863735	false
	HGL 6	45468180	47101818	74364710	75933585	+	#980157	1633638	1568875	196724	82554	196724	1830362	false
	HGL 6	47184372	47693513	76061080	76587950	+	#980157	509141	526870	82554	856	82554	591695	false
	HGL 6	47694369	47763402	76669804	76732894	+	#980157	69033	63090	856		856	69889	false
	HGL 9	48423401	66284026	44947974	65060142	+	#036A9C	17860625	20112168		5788			
	HGL 9	66289814	66422093	65166233	65226884	+	#036A9C	132279	60651	5788	13576	5788	138067	false
	HGL 9	66435669	67175838	65353034	66266865	+	#036A9C	740169	913831	13576	80132	13576	753745	false
	HGL 9	67255970	67312097	44751711	44805253	-	#036A9C	56127	53542	80132	46535	80132	136259	false
	HGL 9	67358632	67425922	44751715	44805253	-	#036A9C	67290	53538	46535	27278	46535	113825	false
	HGL 9	67453200	68233462	44145755	44807760	-	#036A9C	780262	662005	27278	6226	27278	807540	false
	HGL 9	68239688	68291424	44061648	44135561	+	#036A9C	51736	73913	6226	57117	6226	57962	false
	HGL 9	68348541	68421691	43941960	44025230	-	#036A9C	73150	83270	57117	47689	57117	130267	false
	HGL 9	68469380	70979858	41378280	44029154	-	#036A9C	2510478	2650874	47689	-2292216	47689	2558167	false
	HGL 9	68687642	68738353	44061648	44138803	+	#036A9C	50711	77155	-2292216	2273374	-2292216	-2241505	true
	HGL 9	71011727	71064280	41378280	41429075	+	#036A9C	52553	50795	2273374	154413	2273374	2325927	false
	HGL 9	71218693	72165261	40388951	41337550	-	#036A9C	946568	948599	154413	122303	154413	1100981	false
	HGL 9	72287564	76231140	36258020	40388951	-	#036A9C	3943576	4130931	122303	85828	122303	4065879	false
	HGL 9	76316968	76398448	36248081	36309577	+	#036A9C	81480	61496	85828	25482	85828	167308	false
	HGL 9	76423930	78910965	33605216	36297624	-	#036A9C	2487035	2692408	25482	1897	25482	2512517	false
	HGL 9	78912862	83316954	23557	4130175	+	#036A9C	4404092	4106618	1897	-233690	1897	4405989	false
	HGL 9	83083264	83155054	3936618	3986881	+	#036A9C	71790	50263	-233690	215174	-233690	-161900	true
	HGL 9	83370228	94489667	4132043	15565291	+	#036A9C	11119439	11433248	215174	-9167439	215174	11334613	false
	HGL 9	85322228	85447560	5842969	6015916	-	#036A9C	125332	172947	-9167439	6425656	-9167439	-9042107	true
	HGL 9	91873216	91960313	65066373	65157349	+	#036A9C	87097	90976	6425656	2796455	6425656	6512753	false
	HGL 9	94756768	98618828	15576005	19578468	+	#036A9C	3862060	4002463	2796455		2796455	6658515	false
**CPOR 7**	HGL 3	0	5380924	57639608	63384581	-	#C3A652	5380924	5744973		-498690			
	HGL 3	4882234	4937682	32820649	32893550	-	#C3A652	55448	72901	-498690	443242	-498690	-443242	true

	HGL 3	5380924	9730460	52225264	57174241	-	#C3A652	4349536	4948977	443242	4476153	443242	4792778	false
	HGL 3	14206613	14483478	51923719	52198514	-	#C3A652	276865	274795	4476153	66482	4476153	4753018	false
	HGL 3	14549960	14624783	51829382	51922716	-	#C3A652	74823	93334	66482	50439	66482	141305	false
	HGL 3	14675222	15008383	51454414	51826214	-	#C3A652	333161	371800	50439	7949	50439	383600	false
	HGL 3	15016332	15879700	39619210	40690468	-	#C3A652	863368	1071258	7949	49315	7949	871317	false
	HGL 3	15929015	16036366	51078589	51277924	-	#C3A652	107351	199335	49315	61317	49315	156666	false
	HGL 3	16097683	18555708	48295387	51078215	-	#C3A652	2458025	2782828	61317	69	61317	2519342	false
	HGL 3	18555777	18635068	48197249	48295155	+	#C3A652	79291	97906	69	36513	69	79360	false
	HGL 3	18671581	20277431	46191054	48108167	-	#C3A652	1605850	1917113	36513	17520	36513	1642363	false
	HGL 3	20294951	22269423	33237041	35363757	-	#C3A652	1974472	2126716	17520	14907	17520	1991992	false
	HGL 3	22284330	25480651	35500685	39590291	-	#C3A652	3196321	4089606	14907	13249	14907	3211228	false
	HGL 22	25493900	25591703	9622788	9678568	-	#EC83BA	97803	55780		-70823			
	HGL 3	25493900	25594887	42894005	42970033	-	#C3A652	100987	76028	13249	-100987	13249	114236	false
	HGL 3	25493900	25595723	45553926	45622311	-	#C3A652	101823	68385	-100987		-100987	836	false
	HGL 22	25520880	25594887	8988064	9048507	+	#EC83BA	74007	60443	-70823		-70823	3184	false
	HGL 27	25747432	30799102	184520	4798293	+	#EF8701	5051670	4613773		76491			
	HGL 27	30875593	31927645	4811458	5813629	+	#EF8701	1052052	1002171	76491	-20235	76491	1128543	false
	HGL 27	31907410	32014112	5821980	5900284	-	#EF8701	106702	78304	-20235	2124	-20235	86467	false
	HGL 27	32016236	35215163	5905677	8815443	+	#EF8701	3198927	2909766	2124	353958	2124	3201051	false
	HGL 27	35569121	44940792	8816638	18169718	+	#EF8701	9371671	9353080	353958	-7635494	353958	9725629	false
	HGL 27	37305298	37549748	11462190	11581910	-	#EF8701	244450	119720	-7635494	741418	-7635494	-7391044	true
	HGL 27	38291166	38460217	10381434	10713978	-	#EF8701	169051	332544	741418	6516432	741418	910469	false
	HGL 27	44976649	46637069	18220117	19931241	+	#EF8701	1660420	1711124	6516432	221933	6516432	8176852	false
	HGL 27	46859002	48931656	19907490	21810093	+	#EF8701	2072654	1902603	221933	95548	221933	2294587	false
	HGL 27	49027204	49256425	21831850	22063104	+	#EF8701	229221	231254	95548	2347	95548	324769	false
	HGL 27	49258772	49454735	22099383	22242678	-	#EF8701	195963	143295	2347	1633016	2347	198310	false
	HGL 27	51087751	51427842	22290677	22613369	+	#EF8701	340091	322692	1633016	86369	1633016	1973107	false
	HGL 27	51514211	55849227	22620994	27091854	+	#EF8701	4335016	4470860	86369	989	86369	4421385	false
	HGL 27	55850216	56120514	27154652	27373694	+	#EF8701	270298	219042	989	-59109	989	271287	false
	HGL 27	56061405	57115135	27247932	28174853	+	#EF8701	1053730	926921	-59109	21492	-59109	994621	false
	HGL 27	57136627	57201376	43587035	43640200	+	#EF8701	64749	53165	21492	35456	21492	86241	false
	HGL 27	57236832	57319446	43585853	43639108	-	#EF8701	82614	53255	35456	-82614	35456	118070	false

	HGL 27	57236832	57332536	43587035	43637642	+	#EF8701	95704	50607	-82614	72616	-82614	13090	false
	HGL 27	57405152	57531690	43513663	43639108	-	#EF8701	126538	125445	72616	184772	72616	199154	false
	HGL 27	57716462	57780350	43311134	43373371	-	#EF8701	63888	62237	184772	65940	184772	248660	false
	HGL 27	57846290	57901131	43202757	43271856	-	#EF8701	54841	69099	65940	41596	65940	120781	false
	HGL 27	57942727	59470273	41851682	43148577	-	#EF8701	1527546	1296895	41596	1294988	41596	1569142	false
	HGL 27	60765261	60858224	41721981	41798239	-	#EF8701	92963	76258	1294988	955157	1294988	1387951	false
	HGL 27	61813381	62164263	41355740	41678968	-	#EF8701	350882	323228	955157	1075662	955157	1306039	false
	HGL 27	63239925	63332076	41221984	41309201	+	#EF8701	92151	87217	1075662	158963	1075662	1167813	false
	HGL 27	63491039	63580668	41221984	41298986	+	#EF8701	89629	77002	158963	-49892	158963	248592	false
	HGL 27	63530776	63602157	41176353	41228200	+	#EF8701	71381	51847	-49892	472772	-49892	21489	false
	HGL 27	64074929	64294779	41093007	41227122	-	#EF8701	219850	134115	472772	-170834	472772	692622	false
	HGL 27	64123945	64379242	41009447	41143256	-	#EF8701	255297	133809	-170834	503382	-170834	84463	false
	HGL 27	64882624	67853283	38539558	40994426	-	#EF8701	2970659	2454868	503382	553204	503382	3474041	false
	HGL 27	68406487	68687097	38186813	38419893	-	#EF8701	280610	233080	553204	182818	553204	833814	false
	HGL 27	68869915	68940135	38027271	38121100	+	#EF8701	70220	93829	182818	126801	182818	253038	false
	HGL 27	69066936	69141998	38092885	38159026	+	#EF8701	75062	66141	126801	-31982	126801	201863	false
	HGL 27	69110016	69181760	38104152	38167170	-	#EF8701	71744	63018	-31982	167019	-31982	39762	false
	HGL 27	69348779	69424054	38104133	38157061	-	#EF8701	75275	52928	167019	109904	167019	242294	false
	HGL 27	69533958	70624577	36877340	37980602	-	#EF8701	1090619	1103262	109904	83755	109904	1200523	false
	HGL 27	70708332	71145257	36410256	36864244	-	#EF8701	436925	453988	83755	-26699	83755	520680	false
	HGL 27	71118558	71409924	35992656	36365261	-	#EF8701	291366	372605	-26699	-58953	-26699	264667	false
	HGL 27	71350971	71409924	36057435	36124872	-	#EF8701	58953	67437	-58953	-47730	-58953	0	true
	HGL 27	71362194	71462914	35941527	36010693	-	#EF8701	100720	69166	-47730	-100720	-47730	52990	false
	HGL 27	71362194	71536865	35941527	36096381	+	#EF8701	174671	154854	-100720	-67122	-100720	73951	false
	HGL 27	71469743	71530681	35941273	36012422	+	#EF8701	60938	71149	-67122	-60938	-67122	-6184	true
	HGL 27	71469743	73349326	34268848	36010947	-	#EF8701	1879583	1742099	-60938	578104	-60938	1818645	false
	HGL 27	73927430	74212623	34002082	34262691	-	#EF8701	285193	260609	578104	-7127	578104	863297	false
	HGL 27	74205496	74289516	33725712	33835688	+	#EF8701	84020	109976	-7127	-50146	-7127	76893	false
	HGL 27	74239370	74352544	33629782	33722842	-	#EF8701	113174	93060	-50146	33689	-50146	63028	false
	HGL 27	74386233	74469442	33633103	33730086	-	#EF8701	83209	96983	33689	22279	33689	116898	false
	HGL 27	74491721	74569982	33620836	33730086	-	#EF8701	78261	109250	22279	41643	22279	100540	false
	HGL 27	74611625	74711145	33267694	33354559	-	#EF8701	99520	86865	41643	183364	41643	141163	false

	HGL 27	74894509	74962899	33209153	33263396	+	#EF8701	68390	54243	183364	107455	183364	251754	false
	HGL 27	75070354	75791490	32440788	33165245	-	#EF8701	721136	724457	107455	294473	107455	828591	false
	HGL 27	76085963	78037212	30457450	32398899	-	#EF8701	1951249	1941449	294473	56218	294473	2245722	false
	HGL 27	78093430	80375222	28249870	30457381	-	#EF8701	2281792	2207511	56218	163595	56218	2338010	false
	HGL 27	80538817	80882754	43692047	44034782	+	#EF8701	343937	342735	163595		163595	507532	false
	HGL 2	80964311	84195301	14926614	18278125	-	#1CB707	3230990	3351511		54256			
	HGL 2	84249557	86250966	12731448	14838676	-	#1CB707	2001409	2107228	54256	-50179	54256	2055665	false
	HGL 2	86200787	88894802	10009588	12713879	-	#1CB707	2694015	2704291	-50179		-50179	2643836	false
**CPOR 8**	HGL 16	43733	3930497	44814	3968930	+	#819EF7	3886764	3924116		-31353			
	HGL 16	3899144	3950789	3992700	4052582	-	#819EF7	51645	59882	-31353	-51152	-31353	20292	false
	HGL 16	3899637	16979230	3985189	17957406	+	#819EF7	13079593	13972217	-51152	441	-51152	13028441	false
	HGL 16	16979671	17037831	17957406	18017606	-	#819EF7	58160	60200	441	1497	441	58601	false
	HGL 16	17039328	31700914	18017608	34034919	+	#819EF7	14661586	16017311	1497	33821	1497	14663083	false
	HGL 16	31734735	34038945	34085251	36587490	+	#819EF7	2304210	2502239	33821	144643	33821	2338031	false
	HGL 16	34183588	35032695	36565014	37438808	+	#819EF7	849107	873794	144643	229006	144643	993750	false
	HGL 16	35261701	39485159	37480969	41972264	+	#819EF7	4223458	4491295	229006	124465	229006	4452464	false
	HGL 16	39609624	46117487	42002076	48632527	+	#819EF7	6507863	6630451	124465	-10510	124465	6632328	false
	HGL 16	46106977	46191915	48563799	48632527	+	#819EF7	84938	68728	-10510	-3844	-10510	74428	false
	HGL 16	46188071	46355369	48563799	48655714	+	#819EF7	167298	91915	-3844	77	-3844	163454	false
	HGL 16	46355446	47505727	48723834	49900141	+	#819EF7	1150281	1176307	77	380886	77	1150358	false
	HGL 16	47886613	47989049	50149682	50273268	+	#819EF7	102436	123586	380886	156467	380886	483322	false
	HGL 16	48145516	48217057	50486378	50590295	-	#819EF7	71541	103917	156467	100935	156467	228008	false
	HGL 16	48317992	48486256	50443634	50662416	+	#819EF7	168264	218782	100935	126729	100935	269199	false
	HGL 16	48612985	48813197	50563212	50775724	+	#819EF7	200212	212512	126729	-3560	126729	326941	false
	HGL 16	48809637	48869668	50819459	50902590	+	#819EF7	60031	83131	-3560	79835	-3560	56471	false
	HGL 16	48949503	49004068	50658131	50775720	+	#819EF7	54565	117589	79835	14630	79835	134400	false
	HGL 16	49018698	49133390	50706841	50775724	+	#819EF7	114692	68883	14630	-81580	14630	129322	false
	HGL 16	49051810	49204888	50762065	50902590	+	#819EF7	153078	140525	-81580	-29579	-81580	71498	false
	HGL 16	49175309	49233588	50658131	50775574	+	#819EF7	58279	117443	-29579	35020	-29579	28700	false
	HGL 16	49268608	49401432	50683826	50836579	+	#819EF7	132824	152753	35020	34586	35020	167844	false
	HGL 16	49436018	49663756	50902654	51150645	+	#819EF7	227738	247991	34586	62021	34586	262324	false
	HGL 16	49725777	49917370	51156071	51337056	+	#819EF7	191593	180985	62021	0	62021	253614	false

	HGL 16	49917370	50013979	51355992	51445753	-	#819EF7	96609	89761	0	-2062	0	96609	false
	HGL 16	50011917	51373717	51501349	52816411	+	#819EF7	1361800	1315062	-2062	0	-2062	1359738	false
	HGL 16	51373717	52341800	52892570	53766260	+	#819EF7	968083	873690	0	972	0	968083	false
	HGL 16	52342772	55936508	72721197	76344281	-	#819EF7	3593736	3623084	972	124802	972	3594708	false
	HGL 16	56061310	56228235	72548419	72679027	-	#819EF7	166925	130608	124802	50261	124802	291727	false
	HGL 16	56278496	56377434	72376393	72489175	-	#819EF7	98938	112782	50261	56095	50261	149199	false
	HGL 16	56433529	62199118	65950252	72360810	-	#819EF7	5765589	6410558	56095	3444	56095	5821684	false
	HGL 16	62202562	63980558	63825469	65873168	-	#819EF7	1777996	2047699	3444	72808	3444	1781440	false
	HGL 16	64053366	64199556	63634015	63802716	-	#819EF7	146190	168701	72808	-46469	72808	218998	false
	HGL 16	64153087	66282575	61508028	63633415	-	#819EF7	2129488	2125387	-46469	51950	-46469	2083019	false
	HGL 16	66334525	68118020	59646103	61507228	-	#819EF7	1783495	1861125	51950	69169	51950	1835445	false
	HGL 16	68187189	68256639	57732650	57818194	+	#819EF7	69450	85544	69169	66746	69169	138619	false
	HGL 16	68323385	68440353	59461129	59640054	-	#819EF7	116968	178925	66746	60646	66746	183714	false
	HGL 16	68500999	68559855	59382025	59441539	-	#819EF7	58856	59514	60646	77565	60646	119502	false
	HGL 16	68637420	69311051	58557952	59380415	-	#819EF7	673631	822463	77565	74216	77565	751196	false
	HGL 16	69385267	70341630	57538820	58501970	-	#819EF7	956363	963150	74216	20794	74216	1030579	false
	HGL 16	70362424	72370111	55047831	57473317	-	#819EF7	2007687	2425486	20794		20794	2028481	false
	HGL 26	72613137	72725615	28916114	29029077	+	#64E09A	112478	112963		-87865			
	HGL 26	72637750	72750151	29029212	29142163	+	#64E09A	112401	112951	-87865	92363	-87865	24536	false
	HGL 26	72842514	73107415	29242289	29341316	+	#64E09A	264901	99027	92363	6176822	92363	357264	false
	HGL 26	79284237	84091742	31127765	36156500	+	#64E09A	4807505	5028735	6176822	404224	6176822	10984327	false
	HGL 26	84495966	85157726	40451987	41216681	-	#64E09A	661760	764694	404224	12761	404224	1065984	false
	HGL 26	85170487	85234486	18474766	18576331	+	#64E09A	63999	101565	12761	28799	12761	76760	false
	HGL 26	85263285	88750037	36302346	40327303	-	#64E09A	3486752	4024957	28799	134121	28799	3515551	false
	HGL 26	88884158	90726212	10537485	12708642	-	#64E09A	1842054	2171157	134121	5172	134121	1976175	false
	HGL 26	90731384	90907911	18313478	18465323	+	#64E09A	176527	151845	5172	103912	5172	181699	false
	HGL 26	91011823	91210395	39935776	40280783	-	#64E09A	198572	345007	103912	0	103912	302484	false
	HGL 26	91210395	91389564	39579422	39770489	-	#64E09A	179169	191067	0	28269	0	179169	false
	HGL 26	91417833	94512262	25633694	28782003	+	#64E09A	3094429	3148309	28269	261110	28269	3122698	false
	HGL 26	94773372	96004299	15601268	16891783	-	#64E09A	1230927	1290515	261110	246815	261110	1492037	false
	HGL 26	96251114	97412952	24431464	25650632	+	#64E09A	1161838	1219168	246815	93164	246815	1408653	false
	HGL 26	97506116	97660623	40307707	40498061	-	#64E09A	154507	190354	93164	13	93164	247671	false

	HGL 26	97660636	97871720	39275178	39519247	-	#64E09A	211084	244069	13	222256	13	211097	false
	HGL 26	98093976	98221928	15574807	15699489	+	#64E09A	127952	124682	222256	84074	222256	350208	false
	HGL 26	98306002	98460016	40307707	40498071	-	#64E09A	154014	190364	84074	13	84074	238088	false
	HGL 26	98460029	98611087	39361542	39519247	-	#64E09A	151058	157705	13	126441	13	151071	false
	HGL 26	98737528	98941653	15387927	15619404	-	#64E09A	204125	231477	126441	23311	126441	330566	false
	HGL 26	98964964	101761777	12704077	15632893	-	#64E09A	2796813	2928816	23311	222973	23311	2820124	false
	HGL 26	101984750	103118083	18581176	19789350	+	#64E09A	1133333	1208174	222973	140399	222973	1356306	false
	HGL 26	103258482	103349682	18198410	18313478	-	#64E09A	91200	115068	140399	432419	140399	231599	false
	HGL X	103363013	103616692	4730108	4978092	+	#00FF00	253679	247984		39651			
	HGL X	103656343	103782101	5492989	5579667	+	#00FF00	125758	86678	39651		39651	165409	false
	HGL 26	103782101	103862044	17179286	17239062	-	#64E09A	79943	59776	432419		432419	512362	false
**CPOR 9**	HGL 5	622879	2741319	160511	2224222	+	#093456	2118440	2063711		-54198			
	HGL 5	2687121	11530941	2153851	11118028	+	#093456	8843820	8964177	-54198	-4243519	-54198	8789622	false
	HGL 5	7287422	7344072	6759604	6834143	+	#093456	56650	74539	-4243519	4229870	-4243519	-4186869	true
	HGL 5	11573942	11689255	11082095	11168620	+	#093456	115313	86525	4229870	-3478	4229870	4345183	false
	HGL 5	11685777	11751445	11070019	11178331	+	#093456	65668	108312	-3478	72198	-3478	62190	false
	HGL 5	11823643	11885718	11070019	11178331	-	#093456	62075	108312	72198	-32615	72198	134273	false
	HGL 5	11853103	11952055	10933477	11067377	-	#093456	98952	133900	-32615	-22163	-32615	66337	false
	HGL 5	11929892	12004187	11113422	11178300	+	#093456	74295	64878	-22163	-68738	-22163	52132	false
	HGL 5	11935449	12069841	11113422	11187196	-	#093456	134392	73774	-68738	-22281	-68738	65654	false
	HGL 5	12047560	25218138	10933477	24752161	+	#093456	13170578	13818684	-22281	-13073713	-22281	13148297	false
	HGL 5	12144425	12198959	10933477	11067377	-	#093456	54534	133900	-13073713	12935873	-13073713	-13019179	true
	HGL 5	25134832	37045753	24726825	37218802	+	#093456	11910921	12491977	12935873	43710	12935873	24846794	false
	HGL 5	37089463	40244965	37270568	40714713	+	#093456	3155502	3444145	43710	57827	43710	3199212	false
	HGL 5	40302792	42377458	40715896	42805753	+	#093456	2074666	2089857	57827		57827	2132493	false
	HGL 6	42429597	42518050	105172084	105228490	+	#980157	88453	56406		94232			
	HGL 6	42612282	42698551	105231000	105293070	+	#980157	86269	62070	94232		94232	180501	false
	HGL 12	42789355	53040333	75617118	86345360	-	#F33071	10250978	10728242		123			
	HGL 12	53040456	59761724	68938538	75516904	-	#F33071	6721268	6578366	123	172207	123	6721391	false
	HGL 12	59933931	61923719	66835456	68928283	-	#F33071	1989788	2092827	172207	70022	172207	2161995	false
	HGL 12	61993741	62153494	66635438	66812314	-	#F33071	159753	176876	70022	94668	70022	229775	false
	HGL 12	62248162	62321552	66457375	66509189	-	#F33071	73390	51814	94668	715327	94668	168058	false

	HGL 22	62640356	62703868	8577451	8632275	+	#EC83BA	63512	54824	62271981	208670	62271981	62335493	false
	HGL 22	62912538	62965803	8774823	8834459	-	#EC83BA	53265	59636	208670		208670	261935	false
	HGL 12	63036879	63190830	86437443	86562016	-	#F33071	153951	124573	715327	95635	715327	869278	false
	HGL 12	63286465	66943108	86513564	90068718	+	#F33071	3656643	3555154	95635		95635	3752278	false
**CPOR X**	HGL X	0	644339	152732841	153250973	-	#00FF00	644339	518132		90260			
	HGL X	734599	1184137	152297033	152730871	-	#00FF00	449538	433838	90260	188601	90260	539798	false
	HGL X	1372738	1555415	152124620	152294811	-	#00FF00	182677	170191	188601	42210	188601	371278	false
	HGL X	1597625	2310649	151264145	152068050	-	#00FF00	713024	803905	42210	473251	42210	755234	false
	HGL X	2783900	3456359	150505822	151259266	-	#00FF00	672459	753444	473251	11255	473251	1145710	false
	HGL X	3467614	3645942	150370470	150513617	+	#00FF00	178328	143147	11255	3120	11255	189583	false
	HGL X	3649062	4032751	149885857	150354094	-	#00FF00	383689	468237	3120	-60666	3120	386809	false
	HGL X	3972085	4113201	149770374	149885834	-	#00FF00	141116	115460	-60666	1120321	-60666	80450	false
	HGL X	5233522	5446176	149182967	149384869	+	#00FF00	212654	201902	1120321	783560	1120321	1332975	false
	HGL X	6229736	6397404	149595372	149695659	+	#00FF00	167668	100287	783560	75223	783560	951228	false
	HGL X	6472627	6581845	149895143	149967193	-	#00FF00	109218	72050	75223	141255	75223	184441	false
	HGL X	6723100	6841114	149576723	149715024	+	#00FF00	118014	138301	141255	44790	141255	259269	false
	HGL X	6885904	8857218	147114354	149183498	-	#00FF00	1971314	2069144	44790	116047	44790	2016104	false
	HGL X	8973265	9186865	146862284	147074944	+	#00FF00	213600	212660	116047	7583	116047	329647	false
	HGL X	9194448	11010451	145069938	146865966	-	#00FF00	1816003	1796028	7583	97737	7583	1823586	false
	HGL X	11108188	11176942	143445926	143504154	+	#00FF00	68754	58228	97737	9202	97737	166491	false
	HGL X	11186144	20230645	135555325	145042120	-	#00FF00	9044501	9486795	9202	-213478	9202	9053703	false
	HGL X	20017167	20580105	135285471	135612809	+	#00FF00	562938	327338	-213478	37807	-213478	349460	false
	HGL X	20617912	20839271	135573672	135637580	+	#00FF00	221359	63908	37807	-192157	37807	259166	false
	HGL X	20647114	21131313	135147801	135350583	-	#00FF00	484199	202782	-192157	72754	-192157	292042	false
	HGL X	21204067	21319139	135042024	135138136	-	#00FF00	115072	96112	72754	1040614	72754	187826	false
	HGL X	22359753	22427714	135021375	135076750	-	#00FF00	67961	55375	1040614	491663	1040614	1108575	false
	HGL X	22919377	22987402	134970920	135051560	+	#00FF00	68025	80640	491663	272783	491663	559688	false
	HGL X	23260185	26514501	131528206	134957891	-	#00FF00	3254316	3429685	272783	72364	272783	3527099	false
	HGL X	26586865	28538329	129333473	131474207	-	#00FF00	1951464	2140734	72364	266105	72364	2023828	false
	HGL X	28804434	30205599	127939044	129347261	-	#00FF00	1401165	1408217	266105	336552	266105	1667270	false
	HGL X	30542151	31053887	127415076	127926243	-	#00FF00	511736	511167	336552	71517	336552	848288	false
	HGL X	31125404	31195065	127362919	127412930	-	#00FF00	69661	50011	71517	3433	71517	141178	false

	HGL X	31198498	31293552	127174840	127298318	-	#00FF00	95054	123478	3433	53196	3433	98487	false
	HGL X	31346748	31453544	127030615	127174840	-	#00FF00	106796	144225	53196	18	53196	159992	false
	HGL X	31453562	31795702	126784841	127030615	+	#00FF00	342140	245774	18	109574	18	342158	false
	HGL X	31905276	32100677	126549142	126740731	-	#00FF00	195401	191589	109574	78576	109574	304975	false
	HGL X	32179253	35493921	123057694	126544566	-	#00FF00	3314668	3486872	78576	57145	78576	3393244	false
	HGL X	35551066	38188171	120229159	123047524	-	#00FF00	2637105	2818365	57145	84	57145	2694250	false
	HGL X	38188255	38241808	120327594	120384752	+	#00FF00	53553	57158	84	0	84	53637	false
	HGL X	38241808	39675151	118629520	120225609	-	#00FF00	1433343	1596089	0	361114	0	1433343	false
	HGL X	40036265	42696718	115587775	118433288	-	#00FF00	2660453	2845513	361114	57222	361114	3021567	false
	HGL X	42753940	43413702	114776095	115524278	-	#00FF00	659762	748183	57222	511	57222	716984	false
	HGL X	43414213	43614969	114489535	114673329	-	#00FF00	200756	183794	511	350328	511	201267	false
	HGL X	43965297	44021389	114145856	114201168	-	#00FF00	56092	55312	350328	83928	350328	406420	false
	HGL X	44105317	45096353	113160998	114060557	-	#00FF00	991036	899559	83928	2154	83928	1074964	false
	HGL X	45098507	45584598	112542098	113103336	-	#00FF00	486091	561238	2154	2349	2154	488245	false
	HGL X	45586947	46786384	111239930	112491134	-	#00FF00	1199437	1251204	2349	67821	2349	1201786	false
	HGL X	46854205	46965830	111077404	111230898	-	#00FF00	111625	153494	67821	114170	67821	179446	false
	HGL X	47080000	47230637	110820319	111023840	+	#00FF00	150637	203521	114170	113750	114170	264807	false
	HGL X	47344387	49790430	107979912	110765674	-	#00FF00	2446043	2785762	113750	61701	113750	2559793	false
	HGL X	49852131	49944261	107883329	107945218	-	#00FF00	92130	61889	61701	25471	61701	153831	false
	HGL X	49969732	50433297	107360720	107829466	-	#00FF00	463565	468746	25471	38912	25471	489036	false
	HGL X	50472209	51662721	105961583	107293084	-	#00FF00	1190512	1331501	38912	122773	38912	1229424	false
	HGL X	51785494	52123595	105662110	105891929	-	#00FF00	338101	229819	122773	0	122773	460874	false
	HGL X	52123595	52277755	105501943	105576767	-	#00FF00	154160	74824	0	28880	0	154160	false
	HGL X	52306635	52364185	105343743	105408214	-	#00FF00	57550	64471	28880	57437	28880	86430	false
	HGL X	52421622	52752521	104883460	105247517	-	#00FF00	330899	364057	57437	857	57437	388336	false
	HGL X	52753378	53309867	104205011	104832954	-	#00FF00	556489	627943	857	52073	857	557346	false
	HGL X	53361940	54027665	103519187	104197944	-	#00FF00	665725	678757	52073	58082	52073	717798	false
	HGL X	54085747	54474244	103107369	103502805	-	#00FF00	388497	395436	58082	178878	58082	446579	false
	HGL X	54653122	55156319	102491033	102997118	-	#00FF00	503197	506085	178878	86525	178878	682075	false
	HGL X	55242844	55488163	102356950	102458632	+	#00FF00	245319	101682	86525	119103	86525	331844	false
	HGL X	55607266	55701724	102287364	102381077	-	#00FF00	94458	93713	119103	96945	119103	213561	false
	HGL X	55798669	55974443	102022123	102209947	-	#00FF00	175774	187824	96945	395	96945	272719	false

	HGL X	55974838	57989521	99536586	101970731	-	#00FF00	2014683	2434145	395	7709	395	2015078	false
	HGL X	57997230	58540762	98868986	99472617	-	#00FF00	543532	603631	7709	48897	7709	551241	false
	HGL X	58589659	58666956	98474424	98536547	+	#00FF00	77297	62123	48897	27819	48897	126194	false
	HGL X	58694775	58762676	98319057	98391576	+	#00FF00	67901	72519	27819	192529	27819	95720	false
	HGL X	58955205	59397968	97570208	98107683	-	#00FF00	442763	537475	192529	-3727	192529	635292	false
	HGL X	59394241	60124501	96569652	97363280	-	#00FF00	730260	793628	-3727	48867	-3727	726533	false
	HGL X	60173368	60650867	96097686	96584856	-	#00FF00	477499	487170	48867	21698	48867	526366	false
	HGL X	60672565	61011563	95671049	96040031	-	#00FF00	338998	368982	21698	13400	21698	360696	false
	HGL X	61024963	61139844	95568012	95665238	+	#00FF00	114881	97226	13400	12	13400	128281	false
	HGL X	61139856	61349551	95291142	95544974	-	#00FF00	209695	253832	12	51086	12	209707	false
	HGL X	61400637	61749658	94849063	95281223	-	#00FF00	349021	432160	51086	7	51086	400107	false
	HGL X	61749665	61835609	94783528	94841904	+	#00FF00	85944	58376	7	249	7	85951	false
	HGL X	61835858	62678379	93891467	94772180	-	#00FF00	842521	880713	249	111349	249	842770	false
	HGL X	62789728	62994174	93688344	93815282	-	#00FF00	204446	126938	111349	621	111349	315795	false
	HGL X	62994795	63474783	93118216	93638265	-	#00FF00	479988	520049	621	12736	621	480609	false
	HGL X	63487519	64050837	92388831	93037578	-	#00FF00	563318	648747	12736	140	12736	576054	false
	HGL X	64050977	64529767	91884844	92326197	-	#00FF00	478790	441353	140	82266	140	478930	false
	HGL X	64612033	65817990	90477864	91841480	-	#00FF00	1205957	1363616	82266	162738	82266	1288223	false
	HGL X	65980728	66129503	90173675	90315083	-	#00FF00	148775	141408	162738	152086	162738	311513	false
	HGL X	66281589	66731551	89687052	90058368	-	#00FF00	449962	371316	152086	25002	152086	602048	false
	HGL X	66756553	67048151	89455693	89602702	-	#00FF00	291598	147009	25002	32270	25002	316600	false
	HGL X	67080421	67172545	89361976	89443674	+	#00FF00	92124	81698	32270	7672	32270	124394	false
	HGL X	67180217	67666369	88900150	89361976	-	#00FF00	486152	461826	7672	124	7672	493824	false
	HGL X	67666493	67882344	88307189	88610837	-	#00FF00	215851	303648	124	577160	124	215975	false
	HGL X	68459504	68839069	84376806	84712463	+	#00FF00	379565	335657	577160	151426	577160	956725	false
	HGL X	68990495	69144575	84938259	85109480	+	#00FF00	154080	171221	151426	12412	151426	305506	false
	HGL X	69156987	69250622	85259031	85329524	+	#00FF00	93635	70493	12412	38284	12412	106047	false
	HGL X	69288906	69347988	85504693	85598447	+	#00FF00	59082	93754	38284	181977	38284	97366	false
	HGL X	69529965	69724685	83236496	83384139	-	#00FF00	194720	147643	181977	969	181977	376697	false
	HGL X	69725654	69861349	83015646	83162671	-	#00FF00	135695	147025	969	32916	969	136664	false
	HGL X	69894265	70074235	82340958	82407018	-	#00FF00	179970	66060	32916	3234	32916	212886	false
	HGL X	70077469	70168236	81492014	81627225	-	#00FF00	90767	135211	3234	1152	3234	94001	false

	HGL X	70169388	70259947	81346165	81414997	-	#00FF00	90559	68832	1152	123342	1152	91711	false
	HGL X	70383289	71155031	80235260	81162900	-	#00FF00	771742	927640	123342	70143	123342	895084	false
	HGL X	71225174	71461182	79700105	80073627	-	#00FF00	236008	373522	70143	29428	70143	306151	false
	HGL X	71490610	71763287	79369050	79590017	-	#00FF00	272677	220967	29428	262617	29428	302105	false
	HGL X	72025904	72392638	78719819	79059637	-	#00FF00	366734	339818	262617	19992	262617	629351	false
	HGL X	72412630	72497152	78498632	78609910	-	#00FF00	84522	111278	19992	339951	19992	104514	false
	HGL X	72837103	72991492	77958170	78070843	-	#00FF00	154389	112673	339951	263672	339951	494340	false
	HGL X	73255164	73399827	76739021	76902657	-	#00FF00	144663	163636	263672	252347	263672	408335	false
	HGL X	73652174	73884494	76141288	76438865	-	#00FF00	232320	297577	252347	141583	252347	484667	false
	HGL X	74026077	74152071	75892692	75989847	-	#00FF00	125994	97155	141583	57793	141583	267577	false
	HGL X	74209864	74391177	74819470	74967207	-	#00FF00	181313	147737	57793	62476	57793	239106	false
	HGL X	74453653	74556082	74678895	74794237	-	#00FF00	102429	115342	62476	432422	62476	164905	false
	HGL X	74988504	75153921	74001503	74130598	-	#00FF00	165417	129095	432422	99800	432422	597839	false
	HGL X	75253721	76008164	73103668	73885763	-	#00FF00	754443	782095	99800	319839	99800	854243	false
	HGL X	76328003	76388711	72687478	72745968	-	#00FF00	60708	58490	319839	106102	319839	380547	false
	HGL X	76494813	76708827	72498506	72663330	-	#00FF00	214014	164824	106102	796761	106102	320116	false
	HGL X	77505588	77612919	71486401	71551865	-	#00FF00	107331	65464	796761	54357	796761	904092	false
	HGL X	77667276	77790041	71280101	71421404	-	#00FF00	122765	141303	54357	424001	54357	177122	false
	HGL X	78214042	78641101	70707813	71107463	-	#00FF00	427059	399650	424001	73766	424001	851060	false
	HGL X	78714867	78854819	70589817	70688512	-	#00FF00	139952	98695	73766	217281	73766	213718	false
	HGL X	79072100	79206558	70310080	70449129	-	#00FF00	134458	139049	217281	106213	217281	351739	false
	HGL X	79312771	79640134	70011182	70267260	-	#00FF00	327363	256078	106213	33961	106213	433576	false
	HGL X	79674095	79848773	69549707	69757993	-	#00FF00	174678	208286	33961	159003	33961	208639	false
	HGL X	80007776	80081686	69284706	69354784	-	#00FF00	73910	70078	159003	519976	159003	232913	false
	HGL X	80601662	80725645	68714805	68854716	-	#00FF00	123983	139911	519976	192320	519976	643959	false
	HGL X	80917965	81116942	68281291	68485616	-	#00FF00	198977	204325	192320	138296	192320	391297	false
	HGL X	81255238	82964641	66388697	68128468	-	#00FF00	1709403	1739771	138296	520692	138296	1847699	false
	HGL X	83485333	83656702	65960563	66175385	-	#00FF00	171369	214822	520692	212899	520692	692061	false
	HGL X	83869601	84347529	65101110	65597370	-	#00FF00	477928	496260	212899	53668	212899	690827	false
	HGL X	84401197	86204875	63597894	65040805	-	#00FF00	1803678	1442911	53668	211980	53668	1857346	false
	HGL X	86416855	86655616	57952715	58267528	+	#00FF00	238761	314813	211980	201112	211980	450741	false
	HGL X	86856728	86957500	58473408	58587234	-	#00FF00	100772	113826	201112	148231	201112	301884	false

	HGL X	87105731	87173543	58828324	58913013	+	#00FF00	67812	84689	148231	32214	148231	216043	false
	HGL X	87205757	87353344	58976076	59123523	+	#00FF00	147587	147447	32214	191196	32214	179801	false
	HGL X	87544540	88085487	59383450	59783395	+	#00FF00	540947	399945	191196	58372	191196	732143	false
	HGL X	88143859	88522086	59880936	60245791	+	#00FF00	378227	364855	58372	13594	58372	436599	false
	HGL X	88535680	88631654	60332648	60408084	+	#00FF00	95974	75436	13594	193245	13594	109568	false
	HGL X	88824899	89078673	60475386	60759653	+	#00FF00	253774	284267	193245	70720	193245	447019	false
	HGL X	89149393	89265713	60819102	60936926	-	#00FF00	116320	117824	70720	67626	70720	187040	false
	HGL X	89333339	89391820	60975662	61074573	+	#00FF00	58481	98911	67626	22030	67626	126107	false
	HGL X	89413850	90475102	61138386	62242272	+	#00FF00	1061252	1103886	22030	71918	22030	1083282	false
	HGL X	90547020	90650795	62436134	62548185	+	#00FF00	103775	112051	71918	46309	71918	175693	false
	HGL X	90697104	91225543	62624137	63258693	+	#00FF00	528439	634556	46309	800580	46309	574748	false
	HGL 4	91406589	91703752	61894825	62352894	-	#5654E8	297163	458069		0			
	HGL 4	91703752	91781893	61687713	61808224	-	#5654E8	78141	120511	0		0	78141	false
	HGL X	92026123	92143631	57476253	57604862	+	#00FF00	117508	128609	800580	329081	800580	918088	false
	HGL X	92472712	92580677	56683757	56759197	-	#00FF00	107965	75440	329081	102023	329081	437046	false
	HGL X	92682700	92740862	56323114	56383846	-	#00FF00	58162	60732	102023	197674	102023	160185	false
	HGL X	92938536	93082381	55890576	56022938	-	#00FF00	143845	132362	197674	94245	197674	341519	false
	HGL X	93176626	93238117	55506769	55579162	-	#00FF00	61491	72393	94245	192564	94245	155736	false
	HGL X	93430681	93605962	55049813	55235972	-	#00FF00	175281	186159	192564	140444	192564	367845	false
	HGL X	93746406	93919268	54782122	54903334	-	#00FF00	172862	121212	140444	120337	140444	313306	false
	HGL X	94039605	94220167	54399120	54553415	-	#00FF00	180562	154295	120337	126126	120337	300899	false
	HGL X	94346293	94802974	53838434	54248481	-	#00FF00	456681	410047	126126	30	126126	582807	false
	HGL X	94803004	94869089	53787243	53838434	+	#00FF00	66085	51191	30	1941	30	66115	false
	HGL X	94871030	95089781	53492989	53792801	-	#00FF00	218751	299812	1941	449858	1941	220692	false
	HGL X	95539639	96350960	52267294	53053010	-	#00FF00	811321	785716	449858	-118221	449858	1261179	false
	HGL X	96232739	96472229	52192512	52350577	+	#00FF00	239490	158065	-118221	68380	-118221	121269	false
	HGL X	96540609	96641172	51985816	52041722	+	#00FF00	100563	55906	68380	163165	68380	168943	false
	HGL X	96804337	97012636	51612015	51868283	-	#00FF00	208299	256268	163165	142709	163165	371464	false
	HGL X	97155345	97240138	51445852	51530452	-	#00FF00	84793	84600	142709	82586	142709	227502	false
	HGL X	97322724	97593884	50812358	51311212	-	#00FF00	271160	498854	82586	88072	82586	353746	false
	HGL X	97681956	98332034	50130082	50791087	-	#00FF00	650078	661005	88072	62210	88072	738150	false
	HGL X	98394244	98963957	49620115	50094421	-	#00FF00	569713	474306	62210	175	62210	631923	false

	HGL X	98964132	99311830	49113684	49544534	-	#00FF00	347698	430850	175	60451	175	347873	false
	HGL X	99372281	100506889	48036573	49085865	-	#00FF00	1134608	1049292	60451	2676	60451	1195059	false
	HGL X	100509565	100602901	47854562	47928176	-	#00FF00	93336	73614	2676	55125	2676	96012	false
	HGL X	100658026	101081517	47437122	47822862	-	#00FF00	423491	385740	55125	261009	55125	478616	false
	HGL X	101342526	103144139	45094947	47080829	-	#00FF00	1801613	1985882	261009	293703	261009	2062622	false
	HGL X	103437842	103531936	44861952	44949641	-	#00FF00	94094	87689	293703	63708	293703	387797	false
	HGL X	103595644	103666664	44742750	44798257	-	#00FF00	71020	55507	63708	109674	63708	134728	false
	HGL X	103776338	105369951	42940783	44599689	-	#00FF00	1593613	1658906	109674	17389	109674	1703287	false
	HGL X	105387340	111979066	36296260	42883494	-	#00FF00	6591726	6587234	17389	178615	17389	6609115	false
	HGL X	112157681	113700066	34778194	36214378	-	#00FF00	1542385	1436184	178615	-1067959	178615	1721000	false
	HGL X	112632107	112739489	33475332	33536587	+	#00FF00	107382	61255	-1067959	41437	-1067959	-960577	true
	HGL X	112780926	112851951	33647281	33764448	+	#00FF00	71025	117167	41437	856381	41437	112462	false
	HGL X	113708332	113845480	34636841	34767345	+	#00FF00	137148	130504	856381	18484	856381	993529	false
	HGL X	113863964	115413253	33206007	34621409	-	#00FF00	1549289	1415402	18484	-417988	18484	1567773	false
	HGL X	114995265	115116639	35862933	35969919	+	#00FF00	121374	106986	-417988	391243	-417988	-296614	true
	HGL X	115507882	115636348	32970170	33127173	-	#00FF00	128466	157003	391243	57189	391243	519709	false
	HGL X	115693537	115772523	33028377	33078394	+	#00FF00	78986	50017	57189	741283	57189	136175	false
	HGL X	116513806	117396088	32264640	32970166	-	#00FF00	882282	705526	741283	50570	741283	1623565	false
	HGL X	117446658	121193461	28565822	32264105	-	#00FF00	3746803	3698283	50570	-94691	50570	3797373	false
	HGL X	121098770	121321054	28497955	28611762	+	#00FF00	222284	113807	-94691	-77207	-94691	127593	false
	HGL X	121243847	125944427	24458394	28588050	-	#00FF00	4700580	4129656	-77207	-164363	-77207	4623373	false
	HGL X	125780064	125988924	24458675	24534047	-	#00FF00	208860	75372	-164363	18935	-164363	44497	false
	HGL X	126007859	126191999	24458394	24539973	+	#00FF00	184140	81579	18935	-44162	18935	203075	false
	HGL X	126147837	126393067	24458394	24533399	+	#00FF00	245230	75005	-44162	46148	-44162	201068	false
	HGL X	126439215	129225028	21896766	24533908	-	#00FF00	2785813	2637142	46148	160543	46148	2831961	false
	HGL X	129385571	129455155	21754038	21873501	+	#00FF00	69584	119463	160543	1395	160543	230127	false
	HGL X	129456550	129532428	21651645	21742165	+	#00FF00	75878	90520	1395	1	1395	77273	false
	HGL X	129532429	137962004	12902989	21643626	-	#00FF00	8429575	8740637	1	67525	1	8429576	false
	HGL X	138029529	143181214	7628789	12880758	-	#00FF00	5151685	5251969	67525	1616986	67525	5219210	false
	HGL 19	143212856	143323710	45507164	45676503	-	#8D3B0C	110854	169339		101350			
	HGL 19	143425060	143551270	45506208	45703558	+	#8D3B0C	126210	197350	101350	802650	101350	227560	false
	HGL 19	144353920	144431980	45506208	45608669	+	#8D3B0C	78060	102461	802650		802650	880710	false

	HGL X	144798200	144879707	12021084	12106068	-	#00FF00	81507	84984	1616986	496885	1616986	1698493	false
	HGL X	145376592	146435248	6560741	7524595	+	#00FF00	1058656	963854	496885	146077	496885	1555541	false
	HGL X	146581325	146940549	4718255	5034294	+	#00FF00	359224	316039	146077	0	146077	505301	false
	HGL X	146940549	147925241	5493381	6430354	+	#00FF00	984692	936973	0	575569	0	984692	false
	HGL X	148500810	148564948	5524117	5583358	+	#00FF00	64138	59241	575569	44112	575569	639707	false
	HGL X	148609060	151385977	3023128	5620862	-	#00FF00	2776917	2597734	44112	-54925	44112	2821029	false
	HGL X	151331052	151415038	2718267	2804414	-	#00FF00	83986	86147	-54925	-71688	-54925	29061	false
	HGL X	151343350	151728633	2810675	2994445	-	#00FF00	385283	183770	-71688	-321359	-71688	313595	false
	HGL X	151407274	151640335	2832223	2917302	+	#00FF00	233061	85079	-321359	8914	-321359	-88298	true
	HGL X	151649249	151752966	2719414	2780278	+	#00FF00	103717	60864	8914	10336	8914	112631	false
	HGL X	151763302	152030698	2254065	2697006	-	#00FF00	267396	442941	10336	1111	10336	277732	false
	HGL X	152031809	152089444	1996660	2179912	-	#00FF00	57635	183252	1111	133996	1111	58746	false
	HGL X	152223440	152291774	1125418	1236271	+	#00FF00	68334	110853	133996		133996	202330	false

Scaffold – scaffold in reference genome; start – the start of the syntenic block in the reference genome; end – the end of the syntenic block in the reference genome; query – scaffold in query genome; query_start – the start of the syntenic block in the query genome; query_end – end of the syntenic block in the query genome; strand – strand ("+" if the block orientation is the same in the reference genome and the query genomes, "-" if the block orientation is not the same in the reference genome and the query genomes); color – the color code of the chromosome corresponding to the block in the query genome in the Supplementary Figure S3; target_len – synthetic block length in the reference genome; query_len – synthetic block length in the query genome. The color of the cells in the "query" and "color" columns corresponds to the color code of the chromosomes in the query genome. The color of the target_len and query_len cells corresponds to the length of the synthetic blocks: green – longer than 5 megabases, blue – between 5 and 1 megabases, orange – shorter than 1 megabases. CPOR – *Cavia porcellus*, HGL – *Heterocephalus glaber*, HSA – *Homo sapiens.*

Supplementary Table S2 (part 1). Syntenic blocks with a minimum cut-off length of 50 kilobases in the reference naked mole-rat genome.

scaffold	query	start	end	query_start	query_end	strand	color	target_len	query_len	dist_upstream	dist_downstream	dist_end_start	dist_end_end	embedded
HGL 1	HSA 2	45687	1412081	151339692	152717753	+	#4C0851	1366394	1378061		67754			
	HSA 2	1479835	5781366	147349458	151335751	-	#4C0851	4301531	3986293	67754	485	67754	4369285	false
	HSA 2	5781851	21131304	132366508	147180561	-	#4C0851	15349453	14814053	485	26876	485	15349938	false
	HSA 2	21158180	21255460	113672181	113782199	+	#4C0851	97280	110018	26876	52936	26876	124156	false
	HSA 2	21308396	21821634	113782730	114185212	+	#4C0851	513238	402482	52936	6463	52936	566174	false
	HSA 2	21828097	21996491	114185212	114331835	-	#4C0851	168394	146623	6463	7035	6463	174857	false
	HSA 2	22003526	23576759	114331835	115959391	+	#4C0851	1573233	1627556	7035	9987	7035	1580268	false

	HSA 2	23586746	34728706	116016985	126494621	+	#4C0851	11141960	10477636	9987	51668	9987	11151947	false
	HSA 2	34780374	35295546	126502404	126939634	+	#4C0851	515172	437230	51668	70001	51668	566840	false
	HSA 2	35365547	35642323	126950328	127191818	+	#4C0851	276776	241490	70001	10276	70001	346777	false
	HSA 2	35652599	36272839	127252616	127896208	+	#4C0851	620240	643592	10276	115835	10276	630516	false
	HSA 2	36388674	36469687	130335187	130393705	+	#4C0851	81013	58518	115835	190599	115835	196848	false
	HSA 2	36660286	37149606	130720203	131157934	+	#4C0851	489320	437731	190599	17670	190599	679919	false
	HSA 2	37167276	37525295	129672093	129920729	-	#4C0851	358019	248636	17670	60131	17670	375689	false
	HSA 2	37585426	39509930	127908935	129657995	-	#4C0851	1924504	1749060	60131	32791	60131	1984635	false
	HSA 2	39542721	52853539	152717754	165432539	+	#4C0851	13310818	12714785	32791	59549	32791	13343609	false
	HSA 2	52913088	56649688	165433090	169333324	+	#4C0851	3736600	3900234	59549	-35251	59549	3796149	false
	HSA 2	56614437	64964956	169333228	178162136	+	#4C0851	8350519	8828908	-35251	65882	-35251	8315268	false
	HSA 2	65030838	65109111	177954386	178012848	-	#4C0851	78273	58462	65882	2808	65882	144155	false
	HSA 2	65111919	69454370	178108210	182707284	+	#4C0851	4342451	4599074	2808	61274	2808	4345259	false
	HSA 2	69515644	70255325	182707304	183544663	+	#4C0851	739681	837359	61274	3689	61274	800955	false
	HSA 2	70259014	70486712	183546997	183771413	-	#4C0851	227698	224416	3689	101	3689	231387	false
	HSA 2	70486813	70928421	183784212	184303290	+	#4C0851	441608	519078	101	0	101	441709	false
	HSA 2	70928421	71086674	184315932	184464341	-	#4C0851	158253	148409	0	2634	0	158253	false
	HSA 2	71089308	72182652	184469415	185739743	+	#4C0851	1093344	1270328	2634	73945	2634	1095978	false
	HSA 2	72256597	72449341	185906456	186118940	-	#4C0851	192744	212484	73945	5175	73945	266689	false
	HSA 2	72454516	72716669	186167031	186395602	+	#4C0851	262153	228571	5175	10185	5175	267328	false
	HSA 2	72726854	72866703	186469460	186606117	+	#4C0851	139849	136657	10185	326726	10185	150034	false
	HSA 2	73193429	75398116	194168712	196828428	-	#4C0851	2204687	2659716	326726	158	326726	2531413	false
	HSA 2	75398274	78851422	189931046	194116180	-	#4C0851	3453148	4185134	158	441	158	3453306	false
	HSA 2	78851863	79184583	186179144	186479346	+	#4C0851	332720	300202	441	860	441	333161	false
	HSA 2	79185443	79251128	186552800	186606707	+	#4C0851	65685	53907	860	14877	860	66545	false
	HSA 2	79266005	82140754	186482555	189694630	+	#4C0851	2874749	3212075	14877	30699	14877	2889626	false
	HSA 2	82171453	85389892	196832386	201222745	-	#4C0851	3218439	4390359	30699	-2838682	30699	3249138	false
	HSA 2	82551210	82659454	200704697	200755829	-	#4C0851	108244	51132	-2838682	2845714	-2838682	-2730438	true
	HSA 2	85505168	85748687	201223017	201481682	+	#4C0851	243519	258665	2845714	10415	2845714	3089233	false
	HSA 2	85759102	85931005	201621849	201785979	+	#4C0851	171903	164130	10415		10415	182318	false
	HSA 4	86000603	86351247	113477832	113830399	+	#11559D	350644	352567		5146			
	HSA 4	86356393	86410100	113780811	113834740	-	#11559D	53707	53929	5146	647	5146	58853	false

	HSA 4	86410747	87091761	113834747	114513553	+	#11559D	681014	678806	647	56663	647	681661	false
	HSA 4	87148424	87832201	114531026	115154917	+	#11559D	683777	623891	56663	41140	56663	740440	false
	HSA 4	87873341	87933137	115154231	115215012	-	#11559D	59796	60781	41140	6656	41140	100936	false
	HSA 4	87939793	91499383	115215687	118418020	+	#11559D	3559590	3202333	6656	1086	6656	3566246	false
	HSA 4	91500469	92030450	118673214	119239527	+	#11559D	529981	566313	1086	108	1086	531067	false
	HSA 4	92030558	92573512	119474046	119967039	+	#11559D	542954	492993	108	-36261	108	543062	false
	HSA 4	92537251	92644066	119971136	120081476	+	#11559D	106815	110340	-36261	36525	-36261	70554	false
	HSA 4	92680591	98466147	120058817	125290328	+	#11559D	5785556	5231511	36525	28529	36525	5822081	false
	HSA 4	98494676	98585883	140236775	140347254	-	#11559D	91207	110479	28529	173647	28529	119736	false
	HSA 4	98759530	102592307	125302632	129045507	+	#11559D	3832777	3742875	173647	24574	173647	4006424	false
	HSA 4	102616881	113212490	129106788	140330595	+	#11559D	10595609	11223807	24574	66	24574	10620183	false
	HSA 9	105046776	105135539	67727112	67831306	+	#FA2D80	88763	104194		-88763			
	HSA 9	105046776	105298202	41364597	41643581	-	#FA2D80	251426	278984	-88763	-220777	-88763	162663	false
	HSA 9	105077425	105298202	39879026	40100914	+	#FA2D80	220777	221888	-220777		-220777	0	true
	HSA 4	113212556	113282225	147616609	147688494	+	#11559D	69669	71885	66	13	66	69735	false
	HSA 4	113282238	113469179	140367675	140612801	-	#11559D	186941	245126	13	41445	13	186954	false
	HSA 4	113510624	116290657	140619607	143524710	+	#11559D	2780033	2905103	41445	38193	41445	2821478	false
	HSA 4	116328850	116445880	154659484	154765434	+	#11559D	117030	105950	38193	22064	38193	155223	false
	HSA 4	116467944	116562230	143575078	143726270	+	#11559D	94286	151192	22064	2304	22064	116350	false
	HSA 4	116564534	116634761	143783234	143908830	+	#11559D	70227	125596	2304	-57411	2304	72531	false
	HSA 4	116577350	116634862	143944783	144022694	+	#11559D	57512	77911	-57411	-57512	-57411	101	false
	HSA 4	116577350	119888047	144054727	147591526	+	#11559D	3310697	3536799	-57512	62736	-57512	3253185	false
	HSA 4	119950783	120008242	147494973	147547690	+	#11559D	57459	52717	62736	-6936	62736	120195	false
	HSA 4	120001306	120118043	147899780	148021883	-	#11559D	116737	122103	-6936	103202	-6936	109801	false
	HSA 4	120221245	120804518	147713719	148345072	+	#11559D	583273	631353	103202	416	103202	686475	false
	HSA 4	120804934	124736475	148485084	152422610	+	#11559D	3931541	3937526	416	-33927	416	3931957	false
	HSA 4	124702548	126623353	152438637	154550904	+	#11559D	1920805	2112267	-33927	47	-33927	1886878	false
	HSA 4	126623400	126687002	154550904	154622851	-	#11559D	63602	71947	47	48838	47	63649	false
	HSA 4	126735840	126889743	158477655	158667516	-	#11559D	153903	189861	48838	23285	48838	202741	false
	HSA 4	126913028	130294597	154733359	158425988	-	#11559D	3381569	3692629	23285	61503	23285	3404854	false
	HSA 4	130356100	132265343	158669779	160965552	+	#11559D	1909243	2295773	61503	3944	61503	1970746	false
	HSA 4	132269287	132325575	160981914	161085747	-	#11559D	56288	103833	3944	13164	3944	60232	false

	HSA 4	132338739	133260703	161089263	162125322	+	#11559D	921964	1036059	13164		13164	935128	false
**HGL 10**	HSA 5	17737	431877	167066	769994	+	#FDB568	414140	602928		-62933			
	HSA 5	368944	779800	770004	1551887	+	#FDB568	410856	781883	-62933	3051	-62933	347923	false
	HSA 5	782851	4259354	1650259	6509306	+	#FDB568	3476503	4859047	3051	6644	3051	3479554	false
	HSA 5	4265998	9845683	6567005	12813648	+	#FDB568	5579685	6246643	6644	-4205148	6644	5586329	false
	HSA 5	5640535	5697968	8100022	8159754	-	#FDB568	57433	59732	-4205148	25766	-4205148	-4147715	true
	HSA 5	5723734	5848145	8495536	8642512	-	#FDB568	124411	146976	25766	229430	25766	150177	false
	HSA 5	6077575	6155245	8177586	8248135	-	#FDB568	77670	70549	229430	6731	229430	307100	false
	HSA 5	6161976	6231093	8090243	8154310	+	#FDB568	69117	64067	6731	9225	6731	75848	false
	HSA 5	6240318	6441588	7925742	8082584	-	#FDB568	201270	156842	9225	44874	9225	210495	false
	HSA 5	6486462	6569724	8355553	8411632	+	#FDB568	83262	56079	44874	3356908	44874	128136	false
	HSA 5	9926632	14180904	12813672	17529960	+	#FDB568	4254272	4716288	3356908	-878	3356908	7611180	false
	HSA 5	14180026	15519160	17649435	19099192	+	#FDB568	1339134	1449757	-878	38862	-878	1338256	false
	HSA 5	15558022	16977635	19151074	20793179	+	#FDB568	1419613	1642105	38862	77119	38862	1458475	false
	HSA 5	17054754	17498464	20923799	21452985	+	#FDB568	443710	529186	77119	17158	77119	520829	false
	HSA 5	17515622	18071731	21543957	22142396	+	#FDB568	556109	598439	17158	60850	17158	573267	false
	HSA 5	18132581	24284606	22207251	29401685	+	#FDB568	6152025	7194434	60850	86	60850	6212875	false
	HSA 5	24284692	28565372	29456054	33988791	+	#FDB568	4280680	4532737	86	139369	86	4280766	false
	HSA 5	28704741	28788230	33968672	34060253	+	#FDB568	83489	91581	139369	217	139369	222858	false
	HSA 5	28788447	30292623	34408241	35954803	+	#FDB568	1504176	1546562	217	-54157	217	1504393	false
	HSA 5	30238466	32216881	36035152	38032150	+	#FDB568	1978415	1996998	-54157	-38898	-54157	1924258	false
	HSA 5	32177983	32264596	37968421	38048058	-	#FDB568	86613	79637	-38898	4369	-38898	47715	false
	HSA 5	32268965	37108347	38048078	42890698	+	#FDB568	4839382	4842620	4369	123996	4369	4843751	false
	HSA 5	37232343	39862860	43103691	45911317	+	#FDB568	2630517	2807626	123996	89194	123996	2754513	false
	HSA 5	39952054	40187981	50275558	50485276	+	#FDB568	235927	209718	89194	32070	89194	325121	false
	HSA 5	40220051	44444436	50538057	54611691	+	#FDB568	4224385	4073634	32070	58976	32070	4256455	false
	HSA 5	44503412	54439914	54643289	64404782	+	#FDB568	9936502	9761493	58976	10117	58976	9995478	false
	HSA 5	54450031	59712138	64455394	69136586	+	#FDB568	5262107	4681192	10117	157566	10117	5272224	false
	HSA 5	59869704	60306662	69149758	69623795	+	#FDB568	436958	474037	157566	-89466	157566	594524	false
	HSA 5	60217196	60360418	70900618	71084787	-	#FDB568	143222	184169	-89466	-64604	-89466	53756	false
	HSA 5	60295814	60360418	70025200	70128504	-	#FDB568	64604	103304	-64604	1	-64604	0	true
	HSA 5	60360419	62367080	71450514	73481897	+	#FDB568	2006661	2031383	1	148375	1	2006662	false

	HSA 5	62515455	62693961	75541020	75707455	-	#FDB568	178506	166435	148375	1789	148375	326881	false
	HSA 5	62695750	63281328	75058334	75482080	-	#FDB568	585578	423746	1789	68960	1789	587367	false
	HSA 5	63350288	63544617	74878047	75058314	+	#FDB568	194329	180267	68960	69094	68960	263289	false
	HSA 5	63613711	63703123	75457293	75551919	+	#FDB568	89412	94626	69094	3825	69094	158506	false
	HSA 5	63706948	65214477	73493979	74888042	+	#FDB568	1507529	1394063	3825	420781	3825	1511354	false
	HSA 5	65635258	70100096	75675195	80086509	+	#FDB568	4464838	4411314	420781	13201210	420781	4885619	false
	HSA 10	70777945	70969668	79919792	80053805	-	#7AB506	191723	134013	8349749	21077	8349749	8541472	false
	HSA 10	70990745	75340940	80258809	84556897	+	#7AB506	4350195	4298088	21077	58319	21077	4371272	false
	HSA 10	75399259	77339738	84590257	86529279	+	#7AB506	1940479	1939022	58319	2201	58319	1998798	false
	HSA 10	77341939	77443781	86606666	86730153	+	#7AB506	101842	123487	2201	1380	2201	104043	false
	HSA 10	77445161	77596040	45876825	46035729	-	#7AB506	150879	158904	1380	-115480	1380	152259	false
	HSA 10	77480560	78944438	48346034	49969746	-	#7AB506	1463878	1623712	-115480	3972	-115480	1348398	false
	HSA 10	78948410	79219022	47037272	47323862	+	#7AB506	270612	286590	3972	1313	3972	274584	false
	HSA 10	79220335	79316747	46580339	46665611	+	#7AB506	96412	85272	1313	-92979	1313	97725	false
	HSA 10	79223768	79622802	86740743	87216735	-	#7AB506	399034	475992	-92979	5838	-92979	306055	false
	HSA 10	79628640	79711544	45612390	45723249	+	#7AB506	82904	110859	5838	-56551	5838	88742	false
	HSA 10	79654993	79733298	45723861	45795688	-	#7AB506	78305	71827	-56551	-78305	-56551	21754	false
	HSA 10	79654993	79738448	50021997	50136256	-	#7AB506	83455	114259	-78305	705761	-78305	5150	false
	HSA 10	80444209	80799397	44461940	44938689	+	#7AB506	355188	476749	705761	93847	705761	1060949	false
	HSA 10	80893244	80973802	50021997	50136266	+	#7AB506	80558	114269	93847	-50647	93847	174405	false
	HSA 10	80923155	81020820	45617885	45720202	-	#7AB506	97665	102317	-50647	54050	-50647	47018	false
	HSA 10	81074870	81233239	45373845	45601010	-	#7AB506	158369	227165	54050	187818	54050	212419	false
	HSA 10	81421057	81483773	37946834	38057630	-	#7AB506	62716	110796	187818	-59275	187818	250534	false
	HSA 10	81424498	81568124	37755779	37903698	-	#7AB506	143626	147919	-59275	7195	-59275	84351	false
	HSA 10	81575319	82040893	42784023	43462053	+	#7AB506	465574	678030	7195	7339	7195	472769	false
	HSA 10	82048232	83124281	43555587	44938667	+	#7AB506	1076049	1383080	7339	426	7339	1083388	false
	HSA 10	83124707	83208221	46388480	46453372	+	#7AB506	83514	64892	426		426	83940	false
	HSA 5	83301306	83444765	181123909	181251982	-	#FDB568	143459	128073	13201210	107036	13201210	13344669	false
	HSA 5	83551801	83648508	181001172	181093451	-	#FDB568	96707	92279	107036		107036	203743	false
**HGL 11**	HSA 8	109262	692096	362093	1189282	+	#02B4A9	582834	827189		20769			
	HSA 8	712865	1225069	1241630	2244724	+	#02B4A9	512204	1003094	20769	104955	20769	532973	false
	HSA 8	1330024	1423210	2501939	2583288	+	#02B4A9	93186	81349	104955	50159	104955	198141	false

	HSA 8	1473369	3647755	2602371	5502550	+	#02B4A9	2174386	2900179	50159	281	50159	2224545	false
	HSA 8	3648036	3954561	5512565	5893311	-	#02B4A9	306525	380746	281	0	281	306806	false
	HSA 8	3954561	4766461	5893543	6928843	+	#02B4A9	811900	1035300	0	166822	0	811900	false
	HSA 8	4933283	5014983	7415969	7491065	-	#02B4A9	81700	75096	166822	-81700	166822	248522	false
	HSA 8	4933283	5046038	7820492	7930817	+	#02B4A9	112755	110325	-81700	424761	-81700	31055	false
	HSA 8	5470799	5567869	7308039	7367127	-	#02B4A9	97070	59088	424761	23971	424761	521831	false
	HSA 8	5591840	7237889	9828096	11992454	-	#02B4A9	1646049	2164358	23971	6778	23971	1670020	false
	HSA 8	7244667	8668242	27526796	29276267	-	#02B4A9	1423575	1749471	6778	3471	6778	1430353	false
	HSA 8	8671713	8744032	39742779	39838258	+	#02B4A9	72319	95479	3471	63599	3471	75790	false
	HSA 8	8807631	12599780	23176487	27548288	-	#02B4A9	3792149	4371801	63599	-23056	63599	3855748	false
	HSA 8	12576724	16875710	18334654	23046467	-	#02B4A9	4298986	4711813	-23056	26286179	-23056	4275930	false
	HSA 4	16920006	18712573	162576717	164496606	+	#11559D	1792567	1919889		499			
	HSA 4	18713072	18852948	164496606	164632174	-	#11559D	139876	135568	499	115	499	140375	false
	HSA 4	18853063	19155360	164636228	164925857	+	#11559D	302297	289629	115	-33038	115	302412	false
	HSA 4	19122322	21864725	165031305	168188286	+	#11559D	2742403	3156981	-33038	154	-33038	2709365	false
	HSA 4	21864879	21996883	168357859	168475757	-	#11559D	132004	117898	154	-125190	154	132158	false
	HSA 4	21871693	21999736	168214899	168320960	-	#11559D	128043	106061	-125190	57617	-125190	2853	false
	HSA 4	22057353	22194517	168214919	168326546	+	#11559D	137164	111627	57617	-132675	57617	194781	false
	HSA 4	22061842	24531641	168357859	170921325	+	#11559D	2469799	2563466	-132675	57083	-132675	2337124	false
	HSA 4	24588724	28579881	170937385	174996264	+	#11559D	3991157	4058879	57083	1236	57083	4048240	false
	HSA 4	28581117	31307562	175144322	178198251	+	#11559D	2726445	3053929	1236	0	1236	2727681	false
	HSA 4	31307562	31487600	178206895	178375245	-	#11559D	180038	168350	0	0	0	180038	false
	HSA 4	31487600	39433211	178386705	186583016	+	#11559D	7945611	8196311	0	70	0	7945611	false
	HSA 4	39433281	39990996	186583650	187090425	-	#11559D	557715	506775	70	24950	70	557785	false
	HSA 4	40015946	41388577	187094193	188340037	+	#11559D	1372631	1245844	24950	1442	24950	1397581	false
	HSA 4	41390019	41492302	188343561	188423087	-	#11559D	102283	79526	1442	1125	1442	103725	false
	HSA 4	41493427	42276936	188423128	189181117	+	#11559D	783509	757989	1125	59014	1125	784634	false
	HSA 4	42335950	42727420	189228226	189633532	+	#11559D	391470	405306	59014	63814	59014	450484	false
	HSA 4	42791234	43066400	189701133	189906408	+	#11559D	275166	205275	63814		63814	338980	false
	HSA 8	43161889	44717191	16735249	18103888	-	#02B4A9	1555302	1368639	26286179	14509	26286179	27841481	false
	HSA 8	44731700	49018661	12721538	16754449	-	#02B4A9	4286961	4032911	14509	9089	14509	4301470	false
	HSA 8	49027750	49543471	8243874	8743188	+	#02B4A9	515721	499314	9089	64068	9089	524810	false

	HSA 8	49607539	50604613	8748670	9787481	+	#02B4A9	997074	1038811	64068	860	64068	1061142	false
	HSA 8	50605473	58076233	29326445	36173972	+	#02B4A9	7470760	6847527	860	10855	860	7471620	false
	HSA 8	58087088	58188359	36173978	36273413	-	#02B4A9	101271	99435	10855	0	10855	112126	false
	HSA 8	58188359	60035409	36273782	37979306	+	#02B4A9	1847050	1705524	0	72812	0	1847050	false
	HSA 8	60108221	61227319	37991752	39163352	+	#02B4A9	1119098	1171600	72812	37915	72812	1191910	false
	HSA 8	61265234	61406884	39530682	39585745	-	#02B4A9	141650	55063	37915	-140475	37915	179565	false
	HSA 8	61266409	61460754	39445958	39523831	+	#02B4A9	194345	77873	-140475	20435	-140475	53870	false
	HSA 8	61481189	61635400	39311297	39442620	+	#02B4A9	154211	131323	20435	498	20435	174646	false
	HSA 8	61635898	61789256	39110666	39287648	+	#02B4A9	153358	176982	498	58965	498	153856	false
	HSA 8	61848221	61924028	39622437	39730224	+	#02B4A9	75807	107787	58965	39465	58965	134772	false
	HSA 8	61963493	63782503	39889619	41452572	+	#02B4A9	1819010	1562953	39465	84816	39465	1858475	false
	HSA 8	63867319	65289944	41470632	42807225	+	#02B4A9	1422625	1336593	84816	113584	84816	1507441	false
	HSA 8	65403528	65610706	42833826	43030662	+	#02B4A9	207178	196836	113584	84580	113584	320762	false
	HSA 8	65695286	65899910	43056241	43205136	+	#02B4A9	204624	148895	84580		84580	289204	false
	HSA 1	66482208	66568781	247468434	247537099	+	#CBE5A7	86573	68665		217836			
	HSA 1	66786617	66838810	248172774	248278869	+	#CBE5A7	52193	106095	217836	229130	217836	270029	false
	HSA 1	67067940	67209600	248300313	248353326	-	#CBE5A7	141660	53013	229130	-5054	229130	370790	false
	HSA 1	67204546	67263153	248195548	248279176	+	#CBE5A7	58607	83628	-5054	656257	-5054	53553	false
	HSA 1	67919410	67990997	247933146	248004400	-	#CBE5A7	71587	71254	656257	880	656257	727844	false
	HSA 1	67991877	68050915	248172774	248275087	-	#CBE5A7	59038	102313	880	774742	880	59918	false
	HSA 1	68825657	68903291	247470404	247537099	-	#CBE5A7	77634	66695	774742	159665	774742	852376	false
	HSA 1	69062956	69115829	247883067	248048153	+	#CBE5A7	52873	165086	159665	606879	159665	212538	false
	HSA 5	69466600	69560580	121927050	122040999	-	#FDB568	93980	113949		812191			
	HSA 1	69722708	69821963	248195548	248279052	-	#CBE5A7	99255	83504	606879	53346298	606879	706134	false
	HSA 5	70372771	80253982	122055226	131038347	+	#FDB568	9881211	8983121	812191	56644	812191	10693402	false
	HSA 5	80310626	80382250	150893109	150986473	-	#FDB568	71624	93364	56644	65400	56644	128268	false
	HSA 5	80447650	82064161	149084276	150809278	-	#FDB568	1616511	1725002	65400	-71018	65400	1681911	false
	HSA 5	81993143	90065346	141199734	149106581	-	#FDB568	8072203	7906847	-71018	-20964	-71018	8001185	false
	HSA 5	90044382	93235431	137889288	141195045	-	#FDB568	3191049	3305757	-20964	9	-20964	3170085	false
	HSA 5	93235440	105017458	100534236	112940839	-	#FDB568	11782018	12406603	9	52472	9	11782027	false
	HSA 5	105069930	105141907	100401986	100468206	-	#FDB568	71977	66220	52472	108224	52472	124449	false
	HSA 5	105250131	106067655	99582583	100374684	+	#FDB568	817524	792101	108224	-87069	108224	925748	false

	HSA 5	105980586	106173753	99312103	99467738	-	#FDB568	193167	155635	-87069	163234	-87069	106098	false
	HSA 5	106336987	113672175	91253258	99236845	-	#FDB568	7335188	7983587	163234	0	163234	7498422	false
	HSA 5	113672175	122742221	80116042	91193899	-	#FDB568	9070046	11077857	0		0	9070046	false
	HSA 1	123168261	123405821	248689020	248918969	-	#CBE5A7	237560	229949	53346298	298690	53346298	53583858	false
	HSA 1	123704511	123758324	247940036	248004400	-	#CBE5A7	53813	64364	298690	743378	298690	352503	false
	HSA 1	124501702	124622386	247296485	247479264	-	#CBE5A7	120684	182779	743378	57165	743378	864062	false
	HSA 1	124679551	125182313	227731967	228520281	+	#CBE5A7	502762	788314	57165		57165	559927	false
**HGL 12**	HSA 4	201356	2392872	554457	3863252	+	#11559D	2191516	3308795		652			
	HSA 4	2393524	2598892	8590125	8913117	-	#11559D	205368	322992	652	313	652	206020	false
	HSA 4	2599205	5276121	4197110	8478611	-	#11559D	2676916	4281501	313	1581	313	2677229	false
	HSA 4	5277702	5693335	9745116	10310482	+	#11559D	415633	565366	1581	60235	1581	417214	false
	HSA 4	5753570	7418128	10399232	12352377	+	#11559D	1664558	1953145	60235	54695	60235	1724793	false
	HSA 4	7472823	7558375	12352377	12447922	-	#11559D	85552	95545	54695	35089	54695	140247	false
	HSA 4	7593464	16749705	12467477	22320411	+	#11559D	9156241	9852934	35089	26866	35089	9191330	false
	HSA 4	16776571	16840277	22320411	22372093	-	#11559D	63706	51682	26866	7293	26866	90572	false
	HSA 4	16847570	19759635	22372429	25580287	+	#11559D	2912065	3207858	7293	21908	7293	2919358	false
	HSA 4	19781543	29008250	25636167	35391791	+	#11559D	9226707	9755624	21908	42113	21908	9248615	false
	HSA 4	29050363	29262601	35462771	35683735	-	#11559D	212238	220964	42113	43879	42113	254351	false
	HSA 4	29306480	33445343	35684346	40005993	+	#11559D	4138863	4321647	43879	52165	43879	4182742	false
	HSA 4	33497508	34556387	40046439	41247237	+	#11559D	1058879	1200798	52165	58097	52165	1111044	false
	HSA 4	34614484	36940702	41247237	43434335	+	#11559D	2326218	2187098	58097	938	58097	2384315	false
	HSA 4	36941640	36999529	43438047	43522057	-	#11559D	57889	84010	938	1979	938	58827	false
	HSA 4	37001508	37930142	43522974	44495991	+	#11559D	928634	973017	1979	63394	1979	930613	false
	HSA 4	37993536	42507411	44560525	49083563	+	#11559D	4513875	4523038	63394	5032	63394	4577269	false
	HSA 4	42512443	47874571	51814471	56882945	+	#11559D	5362128	5068474	5032	64417	5032	5367160	false
	HSA 4	47938988	54193063	56904324	63032849	+	#11559D	6254075	6128525	64417	1302	64417	6318492	false
	HSA 4	54194365	55129728	63083567	64014179	+	#11559D	935363	930612	1302	54382	1302	936665	false
	HSA 4	55184110	56752728	64034928	65688042	+	#11559D	1568618	1653114	54382	67052	54382	1623000	false
	HSA 4	56819780	57477164	65717182	66221312	+	#11559D	657384	504130	67052	1206	67052	724436	false
	HSA 4	57478370	57576659	66224128	66288276	-	#11559D	98289	64148	1206	548	1206	99495	false
	HSA 4	57577207	57739997	66288332	66443696	+	#11559D	162790	155364	548	0	548	163338	false
	HSA 4	57739997	57811265	66444248	66517863	-	#11559D	71268	73615	0	3837	0	71268	false

	HSA 4	57815102	58894387	66518975	67401298	+	#11559D	1079285	882323	3837	23680	3837	1083122	false
	HSA 4	58918067	59855779	67463775	68273573	+	#11559D	937712	809798	23680	9579	23680	961392	false
	HSA 4	59865358	60083696	68310385	68503309	-	#11559D	218338	192924	9579	918830	9579	227917	false
	HSA 4	61002526	61067999	69020134	69072136	+	#11559D	65473	52002	918830	64937	918830	984303	false
	HSA 4	61132936	61220099	69020134	69072136	+	#11559D	87163	52002	64937	467366	64937	152100	false
	HSA 4	61687465	61759887	69587788	69727584	+	#11559D	72422	139796	467366	91488	467366	539788	false
	HSA 4	61851375	62466331	69682018	70073572	+	#11559D	614956	391554	91488	-199029	91488	706444	false
	HSA 4	62267302	62619598	69996754	70374253	+	#11559D	352296	377499	-199029	50000	-199029	153267	false
	HSA 4	62669598	64301252	70471158	71879370	+	#11559D	1631654	1408212	50000	39711	50000	1681654	false
	HSA 4	64340963	66078249	71942117	73456941	+	#11559D	1737286	1514824	39711	53408	39711	1776997	false
	HSA 4	66131657	66330555	73479938	73660001	+	#11559D	198898	180063	53408		53408	252306	false
	HSA 8	66457344	77961097	121015853	133931054	+	#02B4A9	11503753	12915201		66836			
	HSA 8	78027933	87998367	133995976	144756069	+	#02B4A9	9970434	10760093	66836	84993	66836	10037270	false
	HSA 8	88083360	88145568	144874782	144952043	+	#02B4A9	62208	77261	84993	3807565	84993	147201	false
	HSA 12	88187549	89966389	952073	2745935	+	#7444DF	1778840	1793862		1024619			
	HSA 22	89967493	90990455	17064707	18178377	+	#DB00F9	1022962	1113670					
	HSA 12	90991008	91883611	12052	951892	-	#7444DF	892603	939840	1024619		1024619	1917222	false
	HSA 8	91953133	92905580	47257053	48205803	+	#02B4A9	952447	948750	3807565	13798	3807565	4760012	false
	HSA 8	92919378	97753444	48257808	53128662	+	#02B4A9	4834066	4870854	13798	11076	13798	4847864	false
	HSA 8	97764520	97993413	53186473	53436639	+	#02B4A9	228893	250166	11076	8771	11076	239969	false
	HSA 8	98002184	100074019	53489172	55551938	+	#02B4A9	2071835	2062766	8771		8771	2080606	false
**HGL 13**	HSA 6	10928	87941	29569284	29683416	+	#564208	77013	114132		18370			
	HSA 6	106311	272387	30045089	30265604	+	#564208	166076	220515	18370	60324	18370	184446	false
	HSA 6	332711	653796	30542281	30977925	+	#564208	321085	435644	60324	-9	60324	381409	false
	HSA 6	653787	722475	31034657	31181375	+	#564208	68688	146718	-9	13890	-9	68679	false
	HSA 6	736365	807539	31401084	31473165	+	#564208	71174	72081	13890	-71174	13890	85064	false
	HSA 6	736365	809804	29906527	29976269	-	#564208	73439	69742	-71174	-73439	-71174	2265	false
	HSA 6	736365	1374398	31495295	32230431	+	#564208	638033	735136	-73439	10896	-73439	564594	false
	HSA 6	1385294	1475888	32412271	32475083	+	#564208	90594	62812	10896	-19237	10896	101490	false
	HSA 6	1456651	1859181	32551354	33107107	+	#564208	402530	555753	-19237	-63817	-19237	383293	false
	HSA 6	1795364	1973713	33091420	33325085	+	#564208	178349	233665	-63817	73684	-63817	114532	false
	HSA 6	2047397	2557366	33383547	34155072	+	#564208	509969	771525	73684	7506	73684	583653	false

	HSA 6	2564872	7876535	34225075	41218101	+	#564208	5311663	6993026	7506	7238	7506	5319169	false
	HSA 6	7883773	12764208	41268187	48795326	+	#564208	4880435	7527139	7238	312	7238	4887673	false
	HSA 6	12764520	12826731	48885271	49049403	+	#564208	62211	164132	312		312	62523	false
	HSA 10	12897815	13019129	80028180	80211643	+	#7AB506	121314	183463		16999			
	HSA 10	13036128	18215055	73741151	79498116	-	#7AB506	5178927	5756965	16999	508	16999	5195926	false
	HSA 10	18215563	22126081	69024130	73659578	-	#7AB506	3910518	4635448	508	115042	508	3911026	false
	HSA 10	22241123	23175327	67879550	69018130	-	#7AB506	934204	1138580	115042	58427	115042	1049246	false
	HSA 10	23233754	23465974	67605407	67861570	-	#7AB506	232220	256163	58427	20886	58427	290647	false
	HSA 10	23486860	34709495	56451383	67552200	-	#7AB506	11222635	11100817	20886	-1652890	20886	11243521	false
	HSA 10	33056605	33175779	52929954	53048749	+	#7AB506	119174	118795	-1652890	414467	-1652890	-1533716	true
	HSA 10	33590246	33686419	56318504	56401695	-	#7AB506	96173	83191	414467	439035	414467	510640	false
	HSA 10	34125454	34252455	58010003	58166342	-	#7AB506	127001	156339	439035	67082	439035	566036	false
	HSA 10	34319537	34393988	57872554	57924078	-	#7AB506	74451	51524	67082	416079	67082	141533	false
	HSA 10	34810067	40236394	50788610	56450375	-	#7AB506	5426327	5661765	416079	-5161841	416079	5842406	false
	HSA 10	35074553	35147504	53475010	53538264	+	#7AB506	72951	63254	-5161841	2548107	-5161841	-5088890	true
	HSA 10	37695611	37777818	56267026	56351510	+	#7AB506	82207	84484	2548107	2508905	2548107	2630314	false
	HSA 10	40286723	40877045	50194148	50655359	+	#7AB506	590322	461211	2508905	0	2508905	3099227	false
	HSA 10	40877045	40996759	79715216	79794251	-	#7AB506	119714	79035	0	-106899	0	119714	false
	HSA 10	40889860	48821154	87430176	94612562	+	#7AB506	7931294	7182386	-106899	61208	-106899	7824395	false
	HSA 10	48882362	49013772	95006794	95065945	-	#7AB506	131410	59151	61208	-128886	61208	192618	false
	HSA 10	48884886	49049576	94780722	94990748	+	#7AB506	164690	210026	-128886	175720	-128886	35804	false
	HSA 10	49225296	49282545	94680883	94735254	+	#7AB506	57249	54371	175720	-57249	175720	232969	false
	HSA 10	49225296	49294266	95036551	95116937	-	#7AB506	68970	80386	-57249	61725	-57249	11721	false
	HSA 10	49355991	49445998	95234926	95338333	+	#7AB506	90007	103407	61725	73304	61725	151732	false
	HSA 10	49519302	50092660	95311393	95880521	+	#7AB506	573358	569128	73304	76858	73304	646662	false
	HSA 10	50169518	51538790	95905861	97206311	+	#7AB506	1369272	1300450	76858	23609	76858	1446130	false
	HSA 10	51562399	51747260	122639877	122831390	+	#7AB506	184861	191513	23609	31	23609	208470	false
	HSA 10	51747291	54381302	97206327	99771386	+	#7AB506	2634011	2565059	31	84733	31	2634042	false
	HSA 10	54466035	55085553	99782317	100365152	+	#7AB506	619518	582835	84733	78655	84733	704251	false
	HSA 10	55164208	55250211	133507296	133561533	-	#7AB506	86003	54237	78655	917305	78655	164658	false
	HSA 10	56167516	59496470	130348611	133423664	-	#7AB506	3328954	3075053	917305	51215	917305	4246259	false
	HSA 10	59547685	66986559	122844714	130348606	-	#7AB506	7438874	7503892	51215	42379	51215	7490089	false

	HSA 10	67028938	69336112	120291122	122574276	-	#7AB506	2307174	2283154	42379	58917	42379	2349553	false
	HSA 10	69395029	87301593	100296384	120290701	-	#7AB506	17906564	19994317	58917		58917	17965481	false
**HGL 14**	HSA 9	58790	158665	64073559	64127045	+	#FA2D80	99875	53486		161728			
	HSA 9	320393	10864113	72870779	83146021	-	#FA2D80	10543720	10275242	161728	64253	161728	10705448	false
	HSA 9	10928366	11028580	72872903	72986171	+	#FA2D80	100214	113268	64253	16512	64253	164467	false
	HSA 9	11045092	11214278	72871635	73017243	-	#FA2D80	169186	145608	16512	33071	16512	185698	false
	HSA 9	11247349	11344546	72870779	73013603	+	#FA2D80	97197	142824	33071	36907	33071	130268	false
	HSA 9	11381453	11989396	72320617	72903763	-	#FA2D80	607943	583146	36907	86806	36907	644850	false
	HSA 9	12076202	16284605	68234836	72317356	-	#FA2D80	4208403	4082520	86806	-145648	86806	4295209	false
	HSA 9	16138957	21224290	69631	5189600	+	#FA2D80	5085333	5119969	-145648	-29924	-145648	4939685	false
	HSA 9	21194366	21290849	5222030	5326908	+	#FA2D80	96483	104878	-29924	-89645	-29924	66559	false
	HSA 9	21201204	22006840	5308310	6070021	+	#FA2D80	805636	761711	-89645	6229	-89645	715991	false
	HSA 9	22013069	22541518	6127454	6666841	+	#FA2D80	528449	539387	6229	55843	6229	534678	false
	HSA 9	22597361	27408250	6711788	11747676	+	#FA2D80	4810889	5035888	55843	30399	55843	4866732	false
	HSA 9	27438649	30531481	11806591	14966243	+	#FA2D80	3092832	3159652	30399	16810	30399	3123231	false
	HSA 9	30548291	34463718	15075170	19133542	+	#FA2D80	3915427	4058372	16810	24220	16810	3932237	false
	HSA 9	34487938	36484171	19184087	21146281	+	#FA2D80	1996233	1962194	24220	311800	24220	2020453	false
	HSA 9	36795971	39292936	21478864	23939633	+	#FA2D80	2496965	2460769	311800	52444	311800	2808765	false
	HSA 9	39345380	45502561	24009159	29858746	+	#FA2D80	6157181	5849587	52444	54325	52444	6209625	false
	HSA 9	45556886	45714788	29858840	29958038	+	#FA2D80	157902	99198	54325	57514	54325	212227	false
	HSA 9	45772302	46308503	29994720	30538920	+	#FA2D80	536201	544200	57514	76017	57514	593715	false
	HSA 9	46384520	49566797	30604415	33474445	+	#FA2D80	3182277	2870030	76017	2062	76017	3258294	false
	HSA 9	49568859	50644723	33813329	34839833	+	#FA2D80	1075864	1026504	2062	-47290	2062	1077926	false
	HSA 9	50597433	50740785	34714485	34811260	+	#FA2D80	143352	96775	-47290	-116682	-47290	96062	false
	HSA 9	50624103	51968609	34840328	36173464	+	#FA2D80	1344506	1333136	-116682	64224	-116682	1227824	false
	HSA 9	52032833	52211056	36171551	36334261	+	#FA2D80	178223	162710	64224	119933	64224	242447	false
	HSA 9	52330989	52490914	92208490	92349178	+	#FA2D80	159925	140688	119933	7859	119933	279858	false
	HSA 9	52498773	54522307	36334747	38472134	+	#FA2D80	2023534	2137387	7859	0	7859	2031393	false
	HSA 9	54522307	58501252	97275303	101388124	+	#FA2D80	3978945	4112821	0	48665	0	3978945	false
	HSA 9	58549917	72699954	101335891	115976644	+	#FA2D80	14150037	14640753	48665	0	48665	14198702	false
	HSA 9	72699954	72766682	115976689	116037751	-	#FA2D80	66728	61062	0	1	0	66728	false
	HSA 9	72766683	77718624	116037810	121097938	+	#FA2D80	4951941	5060128	1	46	1	4951942	false

	HSA 9	77718670	78088869	121013340	121421489	+	#FA2D80	370199	408149	46	57003	46	370245	false
	HSA 9	78145872	79067914	121439045	122454578	+	#FA2D80	922042	1015533	57003	389331	57003	979045	false
	HSA 9	79457245	84471448	122472925	128154332	+	#FA2D80	5014203	5681407	389331	808336	389331	5403534	false
	HSA 9	85279784	88637611	128156257	133173334	+	#FA2D80	3357827	5017077	808336	171861	808336	4166163	false
	HSA 9	88809472	90156911	133331305	135530996	-	#FA2D80	1347439	2199691	171861	31077	171861	1519300	false
	HSA 9	90187988	90875328	135539549	136724262	-	#FA2D80	687340	1184713	31077	6605	31077	718417	false
	HSA 9	90881933	91878552	136738777	138124581	+	#FA2D80	996619	1385804	6605		6605	1003224	false
**HGL 15**	HSA 13	901231	1306553	24611945	24931784	+	#007F00	405322	319839		-36214			
	HSA 13	1270339	1416584	24420184	24525072	-	#007F00	146245	104888	-36214	312	-36214	110031	false
	HSA 13	1416896	3063281	19605048	21187939	+	#007F00	1646385	1582891	312	21711	312	1646697	false
	HSA 13	3084992	4121544	21372560	22297559	+	#007F00	1036552	924999	21711	34813	21711	1058263	false
	HSA 13	4156357	4721169	22365870	22911586	+	#007F00	564812	545716	34813	36223	34813	599625	false
	HSA 13	4757392	5603715	22998336	23895278	+	#007F00	846323	896942	36223	0	36223	882546	false
	HSA 13	5603715	6031289	23957832	24316955	-	#007F00	427574	359123	0	-76035	0	427574	false
	HSA 13	5955254	7063969	25055557	26096752	+	#007F00	1108715	1041195	-76035	11907	-76035	1032680	false
	HSA 13	7075876	21375558	26178210	38889063	+	#007F00	14299682	12710853	11907	0	11907	14311589	false
	HSA 13	21375558	21609904	38953825	39259361	+	#007F00	234346	305536	0	64349	0	234346	false
	HSA 13	21674253	23355007	39259664	40871557	+	#007F00	1680754	1611893	64349	-128627	64349	1745103	false
	HSA 13	23226380	23449144	52476596	52637575	-	#007F00	222764	160979	-128627	-222764	-128627	94137	false
	HSA 13	23226380	23554390	52175508	52476470	+	#007F00	328010	300962	-222764	9154	-222764	105246	false
	HSA 13	23563544	27601516	48801036	52160021	-	#007F00	4037972	3358985	9154	12713	9154	4047126	false
	HSA 13	27614229	27790240	48630685	48801023	+	#007F00	176011	170338	12713	33856	12713	188724	false
	HSA 13	27824096	30208111	46468408	48629260	-	#007F00	2384015	2160852	33856	10159	33856	2417871	false
	HSA 13	30218270	31185912	45464383	46407147	-	#007F00	967642	942764	10159	64	10159	977801	false
	HSA 13	31185976	31240839	45384805	45456302	+	#007F00	54863	71497	64	21	64	54927	false
	HSA 13	31240860	35542976	41178324	45376501	-	#007F00	4302116	4198177	21	-22078	21	4302137	false
	HSA 13	35520898	35733524	40906488	41145909	-	#007F00	212626	239421	-22078	-15490	-22078	190548	false
	HSA 13	35718034	37893729	52651976	54686818	+	#007F00	2175695	2034842	-15490	683	-15490	2160205	false
	HSA 13	37894412	37968809	54709401	54788571	-	#007F00	74397	79170	683	-40476	683	75080	false
	HSA 13	37928333	38587298	54750158	55495495	+	#007F00	658965	745337	-40476	1	-40476	618489	false
	HSA 13	38587299	38804702	55495802	55709941	-	#007F00	217403	214139	1	16363	1	217404	false
	HSA 13	38821065	44628816	55722996	61808601	+	#007F00	5807751	6085605	16363	54823	16363	5824114	false

	HSA 13	44683639	59126647	61820330	76109609	+	#007F00	14443008	14289279	54823	316	54823	14497831	false
	HSA 13	59126963	59275694	76128018	76266715	-	#007F00	148731	138697	316	1547	316	149047	false
	HSA 13	59277241	69210405	76268748	86202257	+	#007F00	9933164	9933509	1547	1645	1547	9934711	false
	HSA 13	69212050	80143448	86281004	97858625	+	#007F00	10931398	11577621	1645	117049	1645	10933043	false
	HSA 15	74759029	74833109	22645965	22707093	+	#AF0926	74080	61128		466782			
	HSA 15	75299891	75429016	99815551	99906517	-	#AF0926	129125	90966	466782		466782	595907	false
	HSA 13	80260497	82789388	97878538	100682492	+	#007F00	2528891	2803954	117049	1061	117049	2645940	false
	HSA 13	82790449	82851285	100682492	100755349	-	#007F00	60836	72857	1061	35	1061	61897	false
	HSA 13	82851320	91190433	100757384	110562440	+	#007F00	8339113	9805056	35	13624	35	8339148	false
	HSA 13	91204057	91294809	110615263	110763766	+	#007F00	90752	148503	13624	1981	13624	104376	false
	HSA 13	91296790	91356890	110877748	111009431	+	#007F00	60100	131683	1981	7902	1981	62081	false
	HSA 13	91364792	91723699	111105861	111668893	+	#007F00	358907	563032	7902	129866	7902	366809	false
	HSA 13	91853565	92085756	111843416	112192219	+	#007F00	232191	348803	129866	44947	129866	362057	false
	HSA 13	92130703	92910508	112255413	113671678	+	#007F00	779805	1416265	44947	213	44947	824752	false
	HSA 13	92910721	93260380	113723068	114328985	+	#007F00	349659	605917	213		213	349872	false
**HGL 16**	HSA 12	41861	203149	27148407	27336668	-	#7444DF	161288	188261		1860176			
	HSA 22	247663	311415	35508442	35658503	-	#DB00F9	63752	150061		3621			
	HSA 22	315036	432725	35200086	35456085	-	#DB00F9	117689	255999	3621	2916	3621	121310	false
	HSA 22	435641	1595442	33199515	35148107	-	#DB00F9	1159801	1948592	2916	20	2916	1162717	false
	HSA 22	1595462	2062714	32387502	33140349	-	#DB00F9	467252	752847	20		20	467272	false
	HSA 12	2063325	3963905	105020737	107786213	-	#7444DF	1900580	2765476	1860176	19133	1860176	3760756	false
	HSA 12	3983038	4077870	104963282	105015010	+	#7444DF	94832	51728	19133	-39692	19133	113965	false
	HSA 12	4038178	6312730	101887848	104995147	-	#7444DF	2274552	3107299	-39692	2458	-39692	2234860	false
	HSA 12	6315188	7535820	100200391	101832717	-	#7444DF	1220632	1632326	2458	15706	2458	1223090	false
	HSA 12	7551526	34021885	71744847	100149294	-	#7444DF	26470359	28404447	15706	61907	15706	26486065	false
	HSA 12	34083792	37438808	68403955	71721530	-	#7444DF	3355016	3317575	61907	1401	61907	3416923	false
	HSA 12	37440209	41976223	63779114	68304293	-	#7444DF	4536014	4525179	1401	25853	1401	4537415	false
	HSA 12	42002076	48601754	56773453	63430883	-	#7444DF	6599678	6657430	25853	125093	25853	6625531	false
	HSA 12	48726847	49084004	56376746	56754592	-	#7444DF	357157	377846	125093	-14752	125093	482250	false
	HSA 12	49069252	49799908	55667422	56365220	-	#7444DF	730656	697798	-14752	553758	-14752	715904	false
	HSA 12	50353666	50458813	55370727	55453447	-	#7444DF	105147	82720	553758	-1377	553758	658905	false
	HSA 12	50457436	50683046	55248064	55453533	+	#7444DF	225610	205469	-1377	83985	-1377	224233	false

	HSA 12	50767031	51032152	55142905	55322115	-	#7444DF	265121	179210	83985	71547	83985	349106	false
	HSA 12	51103699	51343016	54934737	55136412	-	#7444DF	239317	201675	71547	12976	71547	310864	false
	HSA 12	51355992	51428022	54686119	54765420	+	#7444DF	72030	79301	12976	70650	12976	85006	false
	HSA 12	51498672	52816411	53299473	54687395	-	#7444DF	1317739	1387922	70650	76159	70650	1388389	false
	HSA 12	52892570	53766260	52337542	53299473	-	#7444DF	873690	961931	76159	1279744	76159	949849	false
	HSA 12	55046004	58343855	27515662	30282904	+	#7444DF	3297851	2767242	1279744	-796816	1279744	4577595	false
	HSA 12	57547039	57796150	29916678	30171176	+	#7444DF	249111	254498	-796816	-72729	-796816	-547705	true
	HSA 12	57723421	57823239	30190051	30282142	-	#7444DF	99818	92091	-72729	167401	-72729	27089	false
	HSA 12	57990640	58212183	29566278	29745213	+	#7444DF	221543	178935	167401	133323	167401	388944	false
	HSA 12	58345506	58766329	30380699	30791643	+	#7444DF	420823	410944	133323	2824	133323	554146	false
	HSA 12	58769153	59318261	31249288	31839479	+	#7444DF	549108	590191	2824	213876	2824	551932	false
	HSA 12	59532137	61600588	31955643	33909265	+	#7444DF	2068451	1953622	213876	-78364	213876	2282327	false
	HSA 12	61522224	61600842	33936694	33999965	+	#7444DF	78618	63271	-78364	68477	-78364	254	false
	HSA 12	61669319	61907445	38367791	38595414	+	#7444DF	238126	227623	68477	55060	68477	306603	false
	HSA 12	61962505	62731965	38643967	39464153	+	#7444DF	769460	820186	55060	51444	55060	824520	false
	HSA 12	62783409	65948255	39479696	42396093	+	#7444DF	3164846	2916397	51444	87552	51444	3216290	false
	HSA 12	66035807	71531297	42313202	47443059	+	#7444DF	5495490	5129857	87552	6	87552	5583042	false
	HSA 12	71531303	71582851	47458153	47510641	-	#7444DF	51548	52488	6	0	6	51554	false
	HSA 12	71582851	72489131	47514544	48355589	+	#7444DF	906280	841045	0	118210	0	906280	false
	HSA 12	72607341	72716414	48432928	48527858	+	#7444DF	109073	94930	118210	76069	118210	227283	false
	HSA 12	72792483	73352271	48554386	49129749	+	#7444DF	559788	575363	76069	-45248	76069	635857	false
	HSA 12	73307023	75029112	49129749	51038041	+	#7444DF	1722089	1908292	-45248	3952	-45248	1676841	false
	HSA 12	75033064	75130222	50934467	51041576	+	#7444DF	97158	107109	3952	52994	3952	101110	false
	HSA 12	75183216	76344281	51045153	52336781	+	#7444DF	1161065	1291628	52994		52994	1214059	false
**HGL 17**	HSA 8	86508	1104217	107616083	108892608	-	#02B4A9	1017709	1276525		423			
	HSA 8	1104640	4357350	102744224	107563690	-	#02B4A9	3252710	4819466	423	694	423	3253133	false
	HSA 8	4358044	4430103	102528245	102661410	-	#02B4A9	72059	133165	694	222	694	72753	false
	HSA 8	4430325	5299840	101318561	102440209	-	#02B4A9	869515	1121648	222	135	222	869737	false
	HSA 8	5299975	6003200	120122308	121018038	-	#02B4A9	703225	895730	135	-2031	135	703360	false
	HSA 8	6001169	11499549	113808203	120051292	-	#02B4A9	5498380	6243089	-2031	735	-2031	5496349	false
	HSA 8	11500284	11583418	113708561	113808195	+	#02B4A9	83134	99634	735	-44127	735	83869	false
	HSA 8	11539291	11733701	113526062	113751201	-	#02B4A9	194410	225139	-44127	13782	-44127	150283	false

	HSA 8	11747483	15927382	108909791	113470570	-	#02B4A9	4179899	4560779	13782	7439	13782	4193681	false
	HSA 8	15934821	23970567	92482976	101318358	-	#02B4A9	8035746	8835382	7439	413	7439	8043185	false
	HSA 8	23970980	29956420	85831958	92011965	-	#02B4A9	5985440	6180007	413	493	413	5985853	false
	HSA 8	29956913	30044401	85584343	85636994	+	#02B4A9	87488	52651	493	1766	493	87981	false
	HSA 8	30046167	30523500	85105478	85577901	-	#02B4A9	477333	472423	1766	65083	1766	479099	false
	HSA 8	30588583	32107706	83476280	85055828	-	#02B4A9	1519123	1579548	65083	6203	65083	1584206	false
	HSA 8	32113909	32812456	82636425	83474199	+	#02B4A9	698547	837774	6203	3679	6203	704750	false
	HSA 8	32816135	33834886	81640463	82635786	-	#02B4A9	1018751	995323	3679	-69139	3679	1022430	false
	HSA 8	33765747	42857335	72309264	81636752	-	#02B4A9	9091588	9327488	-69139	-35030	-69139	9022449	false
	HSA 8	42822305	44400828	70801472	72199638	-	#02B4A9	1578523	1398166	-35030	58128	-35030	1543493	false
	HSA 8	44458956	52573302	63160877	70760708	-	#02B4A9	8114346	7599831	58128	55145	58128	8172474	false
	HSA 8	52628447	57313958	58712215	63112161	-	#02B4A9	4685511	4399946	55145	-3616233	55145	4740656	false
	HSA 8	53697725	53798968	62467102	62538527	+	#02B4A9	101243	71425	-3616233	61680	-3616233	-3514990	true
	HSA 8	53860648	53973741	62576930	62678832	+	#02B4A9	113093	101902	61680	3396009	61680	174773	false
	HSA 8	57369750	58801720	57449186	58703914	-	#02B4A9	1431970	1254728	3396009	67381	3396009	4827979	false
	HSA 8	58869101	58953338	57013919	57081916	+	#02B4A9	84237	67997	67381	248	67381	151618	false
	HSA 8	58953586	60190451	55837965	57010486	-	#02B4A9	1236865	1172521	248		248	1237113	false
**HGL 18**	HSA 17	36474	97968	64771123	64838567	-	#C9FE31	61494	67444		-61494			
	HSA 17	36474	556250	44646415	45492153	-	#C9FE31	519776	845738	-61494	17022	-61494	458282	false
	HSA 17	573272	1131018	43477121	44564169	-	#C9FE31	557746	1087048	17022	2748	17022	574768	false
	HSA 17	1133766	1515735	42610104	43229525	-	#C9FE31	381969	619421	2748	-20737	2748	384717	false
	HSA 17	1494998	2279559	41255028	42615093	-	#C9FE31	784561	1360065	-20737	-22798	-20737	763824	false
	HSA 17	2256761	2675043	40721380	41225806	-	#C9FE31	418282	504426	-22798	-40687	-22798	395484	false
	HSA 17	2634356	3794202	39061574	40720333	-	#C9FE31	1159846	1658759	-40687	131	-40687	1119159	false
	HSA 17	3794333	4280515	38245367	39001042	-	#C9FE31	486182	755675	131	37139	131	486313	false
	HSA 17	4317654	5434255	47596155	49090113	+	#C9FE31	1116601	1493958	37139	-40436	37139	1153740	false
	HSA 17	5393819	13430814	49097493	58693054	+	#C9FE31	8036995	9595561	-40436	117888	-40436	7996559	false
	HSA 17	13548702	14708736	58547117	59969737	+	#C9FE31	1160034	1422620	117888	-39640	117888	1277922	false
	HSA 17	14669096	14755571	20711518	20830787	+	#C9FE31	86475	119269	-39640	-86475	-39640	46835	false
	HSA 17	14669096	16474843	60137319	62252028	-	#C9FE31	1805747	2114709	-86475	81385	-86475	1719272	false
	HSA 17	16556228	17770414	36480253	37843052	+	#C9FE31	1214186	1362799	81385	255	81385	1295571	false
	HSA 17	17770669	17844878	60033262	60109361	+	#C9FE31	74209	76099	255	-384	255	74464	false

	HSA 17	17844494	18045921	35968806	36151860	-	#C9FE31	201427	183054	-384	5637	-384	201043	false
	HSA 17	18051558	18356352	35531279	35901807	-	#C9FE31	304794	370528	5637	0	5637	310431	false
	HSA 17	18356352	21171506	32133854	35261219	-	#C9FE31	2815154	3127365	0	113	0	2815154	false
	HSA 17	21171619	21312943	30828803	31005961	-	#C9FE31	141324	177158	113	133546	113	141437	false
	HSA 17	21446489	21553684	30731228	30828689	-	#C9FE31	107195	97461	133546	-52916	133546	240741	false
	HSA 17	21500768	22377606	31050969	32001249	-	#C9FE31	876838	950280	-52916	74708	-52916	823922	false
	HSA 17	22452314	22813801	27231006	27651756	+	#C9FE31	361487	420750	74708	103058	74708	436195	false
	HSA 17	22916859	23347624	27739743	28197156	+	#C9FE31	430765	457413	103058	0	103058	533823	false
	HSA 17	23347624	25459762	28312310	30529600	+	#C9FE31	2112138	2217290	0	-51217	0	2112138	false
	HSA 17	25408545	26503212	142967	1335204	-	#C9FE31	1094667	1192237	-51217	15765	-51217	1043450	false
	HSA 17	26518977	28104831	1337967	3081698	+	#C9FE31	1585854	1743731	15765	270323	15765	1601619	false
	HSA 17	28375154	28469489	3307206	3394714	-	#C9FE31	94335	87508	270323	68159	270323	364658	false
	HSA 17	28537648	29656484	3400764	4628885	+	#C9FE31	1118836	1228121	68159	-4303	68159	1186995	false
	HSA 17	29652181	30934925	5529765	6845290	-	#C9FE31	1282744	1315525	-4303	14534	-4303	1278441	false
	HSA 17	30949459	31388085	4631648	5115377	+	#C9FE31	438626	483729	14534	9789	14534	453160	false
	HSA 17	31397874	31800799	5179568	5585648	+	#C9FE31	402925	406080	9789	115992	9789	412714	false
	HSA 17	31916791	35318091	6918021	10455934	+	#C9FE31	3401300	3537913	115992	-49806	115992	3517292	false
	HSA 17	35268285	37046903	10455371	12181613	+	#C9FE31	1778618	1726242	-49806	43949	-49806	1728812	false
	HSA 17	37090852	37475474	20011822	20319402	+	#C9FE31	384622	307580	43949	-134944	43949	428571	false
	HSA 17	37340530	38156993	15826087	16654105	+	#C9FE31	816463	828018	-134944	41106	-134944	681519	false
	HSA 17	38198099	41936591	12190842	15708963	-	#C9FE31	3738492	3518121	41106	88921	41106	3779598	false
	HSA 17	42025512	42306702	47090397	47346915	-	#C9FE31	281190	256518	88921	-49567	88921	370111	false
	HSA 17	42257135	42824755	45632770	46241243	-	#C9FE31	567620	608473	-49567	30173	-49567	518053	false
	HSA 17	42854928	42966496	46389423	46489133	+	#C9FE31	111568	99710	30173	-111568	30173	141741	false
	HSA 17	42854928	43244817	46607044	47003557	+	#C9FE31	389889	396513	-111568	11345	-111568	278321	false
	HSA 17	43256162	44682646	16919177	18364610	-	#C9FE31	1426484	1445433	11345	-62718	11345	1437829	false
	HSA 17	44619928	44712580	20967434	21046646	+	#C9FE31	92652	79212	-62718	288084	-62718	29934	false
	HSA 17	45000664	45352468	21099081	21496615	+	#C9FE31	351804	397534	288084	98390	288084	639888	false
	HSA 17	45450858	45642805	18856646	19072871	+	#C9FE31	191947	216225	98390	-8624	98390	290337	false
	HSA 17	45634181	46145152	19194143	19755354	+	#C9FE31	510971	561211	-8624	66652	-8624	502347	false
	HSA 17	46211804	46336726	62355766	62449551	-	#C9FE31	124922	93785	66652	21663	66652	191574	false
	HSA 17	46358389	46610302	19768463	19998433	-	#C9FE31	251913	229970	21663	22954	21663	273576	false

	HSA 17	46633256	48863076	62477529	64758892	+	#C9FE31	2229820	2281363	22954	-105429	22954	2252774	false
	HSA 17	48757647	51764346	64969422	68113639	-	#C9FE31	3006699	3144217	-105429	-20195	-105429	2901270	false
	HSA 17	51744151	56287666	68231636	73210521	+	#C9FE31	4543515	4978885	-20195	31216	-20195	4523320	false
	HSA 17	56318882	57568967	73190662	74588684	+	#C9FE31	1250085	1398022	31216	-41909	31216	1281301	false
	HSA 17	57527058	58411815	74605309	75579105	+	#C9FE31	884757	973796	-41909	45428	-41909	842848	false
	HSA 17	58457243	58538782	46934605	47054580	-	#C9FE31	81539	119975	45428	16	45428	126967	false
	HSA 17	58538798	62362911	75579141	80259880	+	#C9FE31	3824113	4680739	16	151000	16	3824129	false
	HSA 17	62513911	63716389	80260053	81735207	+	#C9FE31	1202478	1475154	151000	16	151000	1353478	false
	HSA 17	63716405	64827220	81800892	83231424	+	#C9FE31	1110815	1430532	16		16	1110831	false
**HGL 19**	HSA 16	62344	706393	15424917	16308689	+	#49FEF6	644049	883772		-58809			
	HSA 16	647584	709434	18484014	18558886	-	#49FEF6	61850	74872	-58809	8095	-58809	3041	false
	HSA 16	717529	793422	15234128	15345692	-	#49FEF6	75893	111564	8095	-68875	8095	83988	false
	HSA 16	724547	856797	16416549	16571296	+	#49FEF6	132250	154747	-68875	-132250	-68875	63375	false
	HSA 16	724547	857783	18139822	18295330	-	#49FEF6	133236	155508	-132250	46874	-132250	986	false
	HSA 16	904657	2073252	16763298	18078901	-	#49FEF6	1168595	1315603	46874	158037	46874	1215469	false
	HSA 16	2231289	2300422	18782975	18865664	+	#49FEF6	69133	82689	158037	123250	158037	227170	false
	HSA 16	2423672	2741378	18982364	19381928	-	#49FEF6	317706	399564	123250	17548	123250	440956	false
	HSA 16	2758926	3190323	19385448	19895571	+	#49FEF6	431397	510123	17548	65339	17548	448945	false
	HSA 16	3255662	3770886	19897067	20501184	+	#49FEF6	515224	604117	65339	21219	65339	580563	false
	HSA 16	3792105	3861332	20616258	20705082	-	#49FEF6	69227	88824	21219	9509	21219	90446	false
	HSA 16	3870841	6750925	11533502	14733677	-	#49FEF6	2880084	3200175	9509	1051	9509	2889593	false
	HSA 16	6751976	6816770	11188456	11289285	-	#49FEF6	64794	100829	1051	50402	1051	65845	false
	HSA 16	6867172	7361035	20709252	21333905	+	#49FEF6	493863	624653	50402	8968	50402	544265	false
	HSA 16	7370003	7766321	21945188	22419424	+	#49FEF6	396318	474236	8968	54	8968	405286	false
	HSA 16	7766375	7942161	21558877	21796206	+	#49FEF6	175786	237329	54	-37676	54	175840	false
	HSA 16	7904485	9997617	22561812	25060377	+	#49FEF6	2093132	2498565	-37676	-49134	-37676	2055456	false
	HSA 16	9948483	12779870	25087330	28330677	+	#49FEF6	2831387	3243347	-49134	265	-49134	2782253	false
	HSA 16	12780135	12938777	28820655	29032722	-	#49FEF6	158642	212067	265	56156	265	158907	false
	HSA 16	12994933	13103527	28477173	28614895	+	#49FEF6	108594	137722	56156	-33280	56156	164750	false
	HSA 16	13070247	13500612	29643419	30205408	-	#49FEF6	430365	561989	-33280	7770	-33280	397085	false
	HSA 16	13508382	14508330	30348340	31531343	+	#49FEF6	999948	1183003	7770	571477	7770	1007718	false
	HSA 4	14842043	14920546	122363691	122453936	-	#11559D	78503	90245		11488			

	HSA 4	14932034	14991819	122286931	122359774	+	#11559D	59785	72843	11488	5763614	11488	71273	false
	HSA 16	15079807	15208103	14954804	15100600	+	#49FEF6	128296	145796	571477	10623	571477	699773	false
	HSA 16	15218726	16003311	10385614	11432673	-	#49FEF6	784585	1047059	10623	175481	10623	795208	false
	HSA 16	16178792	21157891	4768626	10302309	-	#49FEF6	4979099	5533683	175481	101350	175481	5154580	false
	HSA 11	20755433	20824442	3525406	3592334	+	#69E283	69009	66928		21745			
	HSA 12	20755433	20824442	8334179	8407894	+	#7444DF	69009	73715		21745			
	HSA 3	20755433	20824442	75516752	75591357	+	#9B0496	69009	74605		21745			
	HSA 4	20755433	20824442	4052841	4118987	+	#11559D	69009	66146	5763614	21745	5763614	5832623	false
	HSA 7	20755433	20824442	97879403	97939692	+	#81A8F8	69009	60289		23801			
	HSA 8	20755433	20824442	7164570	7239333	+	#02B4A9	69009	74763		21745			
	HSA 11	20846187	20915560	71698066	71804772	+	#69E283	69373	106706	21745	-69373	21745	91118	false
	HSA 12	20846187	20916220	8226566	8332793	-	#7444DF	70033	106227	21745	16576222	21745	91778	false
	HSA 4	20846187	20916220	9059827	9171443	+	#11559D	70033	111616	21745	-70033	21745	91778	false
	HSA 8	20846187	20916220	12419822	12541384	-	#02B4A9	70033	121562	21745	-70033	21745	91778	false
	HSA 3	20846187	20918361	130091238	130192003	-	#9B0496	72174	100765	21745	-72174	21745	93919	false
	HSA 4	20846187	20918361	9591941	9710831	+	#11559D	72174	118890	-70033	-72174	-70033	2141	false
	HSA 8	20846187	20918361	8124289	8241422	+	#02B4A9	72174	117133	-70033		-70033	2141	false
	HSA 3	20846187	20918415	75414969	75515354	-	#9B0496	72228	100385	-72174	-72228	-72174	54	false
	HSA 4	20846187	20918476	3935273	4051259	-	#11559D	72289	115986	-72174		-72174	115	false
	HSA 11	20846187	20918527	3401523	3524021	-	#69E283	72340	122498	-69373		-69373	2967	false
	HSA 3	20846187	20918527	125824068	125934781	+	#9B0496	72340	110713	-72228		-72228	112	false
	HSA 7	20848243	20918527	6923581	6999914	-	#81A8F8	70284	76333	23801		23801	94085	false
	HSA 16	21259241	23135955	2895922	4757348	-	#49FEF6	1876714	1861426	101350	9619	101350	1978064	false
	HSA 16	23145574	23277513	2734074	2838023	+	#49FEF6	131939	103949	9619	208294	9619	141558	false
	HSA 16	23485807	23750210	2220353	2543771	-	#49FEF6	264403	323418	208294	70862	208294	472697	false
	HSA 16	23821072	24528907	1482612	2224068	-	#49FEF6	707835	741456	70862	165864	70862	778697	false
	HSA 16	24694771	25964115	28451	1482530	+	#49FEF6	1269344	1454079	165864		165864	1435208	false
	HSA 22	25987289	26298375	19021794	19291967	-	#DB00F9	311086	270173		16022			
	HSA 22	26314397	26577215	20392941	20626226	+	#DB00F9	262818	233285	16022	3234	16022	278840	false
	HSA 22	26580449	27008277	20700317	21081007	-	#DB00F9	427828	380690	3234	14003	3234	431062	false
	HSA 22	27022280	27935041	19356864	20323822	+	#DB00F9	912761	966958	14003	197393	14003	926764	false
	HSA 22	28132434	28497148	21544549	21985513	+	#DB00F9	364714	440964	197393	86767	197393	562107	false

	HSA 22	28583915	28854220	25473134	25730990	-	#DB00F9	270305	257856	86767	-37458	86767	357072	false
	HSA 22	28816762	29260984	24735286	25276737	-	#DB00F9	444222	541451	-37458	91722	-37458	406764	false
	HSA 22	29352706	32789504	25735671	29398529	+	#DB00F9	3436798	3662858	91722	-35320	91722	3528520	false
	HSA 22	32754184	35414174	29416456	32009282	+	#DB00F9	2659990	2592826	-35320	55341	-35320	2624670	false
	HSA 22	35469515	35564141	32034251	32111148	+	#DB00F9	94626	76897	55341	537053	55341	149967	false
	HSA 22	36101194	36745917	22855730	23347362	+	#DB00F9	644723	491632	537053	-53569	537053	1181776	false
	HSA 22	36692348	37076424	24266471	24636386	+	#DB00F9	384076	369915	-53569	-8804	-53569	330507	false
	HSA 22	37067620	37461277	23737410	24250892	-	#DB00F9	393657	513482	-8804	2156944	-8804	384853	false
	HSA 12	37492442	38193821	109253635	110053321	-	#7444DF	701379	799686	16576222	9824	16576222	17277601	false
	HSA 12	38203645	39459681	107913428	109292561	+	#7444DF	1256036	1379133	9824	213688	9824	1265860	false
	HSA 22	39618221	39699985	24715762	24792136	+	#DB00F9	81764	76374	2156944		2156944	2238708	false
	HSA 12	39673369	45567800	114125430	121065576	-	#7444DF	5894431	6940146	213688	145183	213688	6108119	false
	HSA 12	45712983	48767345	110059691	114103569	-	#7444DF	3054362	4043878	145183	-22870	145183	3199545	false
	HSA 12	48744475	50949457	121104364	124605403	+	#7444DF	2204982	3501039	-22870	45333	-22870	2182112	false
	HSA 12	50994790	52496805	124758167	127290068	+	#7444DF	1502015	2531901	45333	-1078290	45333	1547348	false
	HSA 12	51418515	51501718	127557940	127634138	+	#7444DF	83203	76198	-1078290	189227	-1078290	-995087	true
	HSA 12	51690945	51797046	126468699	126629274	+	#7444DF	106101	160575	189227	106794	189227	295328	false
	HSA 12	51903840	51969931	127287984	127376585	-	#7444DF	66091	88601	106794	527058	106794	172885	false
	HSA 12	52496989	53277197	127346260	128746456	+	#7444DF	780208	1400196	527058	10951	527058	1307266	false
	HSA 12	53288148	53962274	128798391	129994389	+	#7444DF	674126	1195998	10951	17	10951	685077	false
	HSA 12	53962291	54257975	130045076	130672866	+	#7444DF	295684	627790	17	8991	17	295701	false
	HSA 12	54266966	54442458	130790640	131139486	+	#7444DF	175492	348846	8991	-9207	8991	184483	false
	HSA 12	54433251	54484385	131390616	131534941	-	#7444DF	51134	144325	-9207	-1203	-9207	41927	false
	HSA 12	54483182	54533440	131273948	131387724	+	#7444DF	50258	113776	-1203	34568	-1203	49055	false
	HSA 12	54568008	54619671	131537341	131636297	+	#7444DF	51663	98956	34568	478	34568	86231	false
	HSA 12	54620149	55484456	131708851	133195251	+	#7444DF	864307	1486400	478		478	864785	false
**HGL 2**	HSA 21	15283	1539350	44499204	46666244	-	#FF7F00	1524067	2167040		0			
	HSA 21	1539350	1987295	43715410	44469116	+	#FF7F00	447945	753706	0	33079	0	447945	false
	HSA 21	2020374	3042401	39836973	41283948	+	#FF7F00	1022027	1446975	33079	2121	33079	1055106	false
	HSA 21	3044522	3095919	41310892	41409178	-	#FF7F00	51397	98286	2121	12473	2121	53518	false
	HSA 21	3108392	3181776	41423705	41563545	+	#FF7F00	73384	139840	12473	20081	12473	85857	false
	HSA 21	3201857	3891972	41719444	42789353	+	#FF7F00	690115	1069909	20081	6240	20081	710196	false

	HSA 21	3898212	4084812	42848016	43131622	+	#FF7F00	186600	283606	6240	31375	6240	192840	false
	HSA 21	4116187	4172382	43272556	43376608	+	#FF7F00	56195	104052	31375	2093	31375	87570	false
	HSA 21	4174475	4275534	5966517	6076907	-	#FF7F00	101059	110390	2093	-101059	2093	103152	false
	HSA 21	4174475	4361616	43461357	43694566	+	#FF7F00	187141	233209	-101059	55731	-101059	86082	false
	HSA 21	4417347	7447880	36125668	39805332	+	#FF7F00	3030533	3679664	55731	125984	55731	3086264	false
	HSA 21	7573864	10875425	32595786	36073412	-	#FF7F00	3301561	3477626	125984	48	125984	3427545	false
	HSA 21	10875473	12710822	30634836	32535052	-	#FF7F00	1835349	1900216	48	115022	48	1835397	false
	HSA 21	12825844	14800244	28921339	30639575	-	#FF7F00	1974400	1718236	115022	126370	115022	2089422	false
	HSA 21	14926614	24201054	18999994	28825275	-	#FF7F00	9274440	9825281	126370	-5937567	126370	9400810	false
	HSA 21	18263487	18414391	23958270	24102295	-	#FF7F00	150904	144025	-5937567	50369	-5937567	-5786663	true
	HSA 21	18464760	18552101	23775065	23895023	-	#FF7F00	87341	119958	50369	1362743	50369	137710	false
	HSA 21	19914844	20039129	24800547	24912042	-	#FF7F00	124285	111495	1362743	4162950	1362743	1487028	false
	HSA 21	24202079	24502460	18694393	18999733	+	#FF7F00	300381	305340	4162950	540	4162950	4463331	false
	HSA 21	24503000	26131957	17324338	18691695	-	#FF7F00	1628957	1367357	540	30519	540	1629497	false
	HSA 21	26162476	29116325	14105860	17273027	-	#FF7F00	2953849	3167167	30519		30519	2984368	false
	HSA 3	29128843	31122228	75810436	77841562	+	#9B0496	1993385	2031126		66			
	HSA 3	31122294	31280409	77845851	77969493	-	#9B0496	158115	123642	66	7	66	158181	false
	HSA 3	31280416	32255753	77973257	78870008	+	#9B0496	975337	896751	7	-49358	7	975344	false
	HSA 3	32206395	43983638	78870026	90262808	+	#9B0496	11777243	11392782	-49358	-113995	-49358	11727885	false
	HSA 3	43869643	45157054	93808606	95176191	+	#9B0496	1287411	1367585	-113995	53857	-113995	1173416	false
	HSA 3	45210911	45349066	95191543	95352817	+	#9B0496	138155	161274	53857	84147	53857	192012	false
	HSA 3	45433213	47847372	95426070	98052313	+	#9B0496	2414159	2626243	84147	-25086	84147	2498306	false
	HSA 3	47822286	47914150	98053305	98103390	-	#9B0496	91864	50085	-25086	28744	-25086	66778	false
	HSA 3	47942894	48186190	98066211	98208927	+	#9B0496	243296	142716	28744	-143402	28744	272040	false
	HSA 3	48042788	48230697	98261800	98337704	+	#9B0496	187909	75904	-143402	56483	-143402	44507	false
	HSA 3	48287180	48365754	98437159	98497254	+	#9B0496	78574	60095	56483	14720	56483	135057	false
	HSA 3	48380474	48832506	98282804	98634174	+	#9B0496	452032	351370	14720	113110	14720	466752	false
	HSA 3	48945616	51242602	98615567	100535998	+	#9B0496	2296986	1920431	113110	92678	113110	2410096	false
	HSA 3	51335280	55276836	100463365	103984870	+	#9B0496	3941556	3521505	92678	445	92678	4034234	false
	HSA 3	55277281	55370662	103995048	104096678	-	#9B0496	93381	101630	445	2171	445	93826	false
	HSA 3	55372833	60084714	104098249	108524763	+	#9B0496	4711881	4426514	2171	2168	2171	4714052	false
	HSA 3	60086882	60143753	108057463	108109451	-	#9B0496	56871	51988	2168	1225	2168	59039	false

	HSA 3	60144978	61419026	108348548	109490579	+	#9B0496	1274048	1142031	1225	45816	1225	1275273	false
	HSA 3	61464842	65408948	109567773	112841068	+	#9B0496	3944106	3273295	45816	-28400	45816	3989922	false
	HSA 3	65380548	70674569	112916471	118038744	+	#9B0496	5294021	5122273	-28400	7631	-28400	5265621	false
	HSA 3	70682200	70829123	118038744	118136728	-	#9B0496	146923	97984	7631	30962	7631	154554	false
	HSA 3	70860085	73794206	118176770	120958284	+	#9B0496	2934121	2781514	30962	7742	30962	2965083	false
	HSA 3	73801948	74548275	121008667	121757552	+	#9B0496	746327	748885	7742	297582	7742	754069	false
	HSA 14	74548783	74658630	42222472	42338219	+	#E181CE	109847	115747		5189			
	HSA 14	74663819	74797399	42488166	42637702	+	#E181CE	133580	149536	5189	16687116	5189	138769	false
	HSA 3	74845857	78821500	121767747	125524976	+	#9B0496	3975643	3757229	297582	-1677428	297582	4273225	false
	HSA 3	77144072	77329199	75243611	75325561	+	#9B0496	185127	81950	-1677428	1484783	-1677428	-1492301	true
	HSA 3	78813982	79887607	38325030	39338835	-	#9B0496	1073625	1013805	1484783	-207	1484783	2558408	false
	HSA 3	79887400	80058306	38122308	38258673	+	#9B0496	170906	136365	-207	246887	-207	170699	false
	HSA 3	80305193	81470516	8436423	9568141	+	#9B0496	1165323	1131718	246887	41184	246887	1412210	false
	HSA 3	81511700	81849362	9649290	9999251	+	#9B0496	337662	349961	41184	-50821	41184	378846	false
	HSA 3	81798541	81907970	10025460	10127304	+	#9B0496	109429	101844	-50821	40405	-50821	58608	false
	HSA 3	81948375	82581126	10141665	10753965	-	#9B0496	632751	612300	40405		40405	673156	false
	HSA 15	82903262	83598939	55162622	56254727	-	#AF0926	695677	1092105		2958			
	HSA 15	83601897	83799670	54775085	55108971	-	#AF0926	197773	333886	2958	612	2958	200731	false
	HSA 15	83800282	90528912	46209188	54713540	-	#AF0926	6728630	8504352	612	475	612	6729242	false
	HSA 15	90529387	91443037	44955999	46155444	-	#AF0926	913650	1199445	475	16050698	475	914125	false
	HSA 14	91484515	99079229	46758192	55209328	-	#E181CE	7594714	8451136	16687116	-68437	16687116	24281830	false
	HSA 14	99010792	103366701	42419197	46758113	-	#E181CE	4355909	4338916	-68437	93035	-68437	4287472	false
	HSA 14	103459736	106568913	38984000	42334522	-	#E181CE	3109177	3350522	93035	50473	93035	3202212	false
	HSA 14	106619386	107493705	38187489	38958253	-	#E181CE	874319	770764	50473	103601	50473	924792	false
	HSA 14	107597306	108500330	37308663	38187409	-	#E181CE	903024	878746	103601	1508	103601	1006625	false
	HSA 14	108501838	111387617	33859567	36809150	-	#E181CE	2885779	2949583	1508	58424	1508	2887287	false
	HSA 14	111446041	118135951	27100273	33858751	-	#E181CE	6689910	6758478	58424	8078	58424	6748334	false
	HSA 9	117395491	117460060	63146352	63202891	+	#FA2D80	64569	56539		-64569			
	HSA 9	117395491	117464558	63252822	63314145	-	#FA2D80	69067	61323	-64569	-69067	-64569	4498	false
	HSA 9	117395491	117519291	40364771	40478609	-	#FA2D80	123800	113838	-69067		-69067	54733	false
	HSA 14	118144029	118263116	27003427	27094932	+	#E181CE	119087	91505	8078	6743	8078	127165	false
	HSA 14	118269859	121545751	23816503	27002809	-	#E181CE	3275892	3186306	6743	67496	6743	3282635	false

	HSA 14	121613247	122680125	22746309	23786195	-	#E181CE	1066878	1039886	67496	61386	67496	1134374	false
	HSA 14	122741511	123103122	22346887	22702330	-	#E181CE	361611	355443	61386	53220	61386	422997	false
	HSA 14	123156342	123669464	21865116	22346026	-	#E181CE	513122	480910	53220	-88004	53220	566342	false
	HSA 14	123581460	123671922	21795996	21895187	-	#E181CE	90462	99191	-88004	11954	-88004	2458	false
	HSA 14	123683876	123743071	22003827	22101886	-	#E181CE	59195	98059	11954	-5123	11954	71149	false
	HSA 14	123737948	123789826	21911104	22003752	-	#E181CE	51878	92648	-5123	-32763	-5123	46755	false
	HSA 14	123757063	123822715	21784546	21845210	-	#E181CE	65652	60664	-32763	16232	-32763	32889	false
	HSA 14	123838947	124005927	21950434	22228832	-	#E181CE	166980	278398	16232	7465	16232	183212	false
	HSA 14	124013392	124113021	21900489	22101418	-	#E181CE	99629	200929	7465	-49338	7465	107094	false
	HSA 14	124063683	125018939	20885328	21877486	-	#E181CE	955256	992158	-49338	726722	-49338	905918	false
	HSA 15	125439895	125571140	101828368	101950224	+	#AF0926	131245	121856	17844649	805202	17844649	17975894	false
	HSA 14	125745661	125845700	20113195	20163476	-	#E181CE	100039	50281	726722	-9806	726722	826761	false
	HSA 14	125835894	125969222	20051007	20158048	-	#E181CE	133328	107041	-9806	3961	-9806	123522	false
	HSA 14	125973183	126237691	19905755	20135883	-	#E181CE	264508	230128	3961	15914	3961	268469	false
	HSA 14	126253605	126483575	19709591	19953548	-	#E181CE	229970	243957	15914	378870	15914	245884	false
	HSA 15	126376342	126677067	21526250	21685703	-	#AF0926	300725	159453	805202	1090224	805202	1105927	false
	HSA 14	126862445	127447449	20179204	20742936	+	#E181CE	585004	563732	378870	-4221	378870	963874	false
	HSA 14	127443228	127546242	20769157	20906862	+	#E181CE	103014	137705	-4221		-4221	98793	false
	HSA 15	127767291	129540402	32609272	34374869	-	#AF0926	1773111	1765597	1090224	53548	1090224	2863335	false
	HSA 15	129593950	138559950	34640418	43643671	+	#AF0926	8966000	9003253	53548	-108840	53548	9019548	false
	HSA 15	138451110	139047688	43658491	44205780	+	#AF0926	596578	547289	-108840	38684	-108840	487738	false
	HSA 15	139086372	139225271	37034775	37154645	-	#AF0926	138899	119870	38684	71702	38684	177583	false
	HSA 15	139296973	139843863	44236496	44734993	-	#AF0926	546890	498497	71702		71702	618592	false
**HGL 20**	HSA 1	14433	3500101	59945679	65110082	+	#CBE5A7	3485668	5164403		14928			
	HSA 1	3515029	5291198	65162152	67462995	+	#CBE5A7	1776169	2300843	14928	47867	14928	1791097	false
	HSA 1	5339065	5497852	67669414	67959118	+	#CBE5A7	158787	289704	47867	3	47867	206654	false
	HSA 1	5497855	6429460	68054704	69264177	+	#CBE5A7	931605	1209473	3	54035	3	931608	false
	HSA 1	6483495	7651932	69316795	70762826	+	#CBE5A7	1168437	1446031	54035	45516	54035	1222472	false
	HSA 1	7697448	18767192	70819287	83210092	+	#CBE5A7	11069744	12390805	45516	94424	45516	11115260	false
	HSA 1	18861616	22057056	83287150	86584008	+	#CBE5A7	3195440	3296858	94424	17765	94424	3289864	false
	HSA 1	22074821	24652493	86540121	89172054	+	#CBE5A7	2577672	2631933	17765	-153999	17765	2595437	false
	HSA 1	24498494	24704709	89175156	89300734	+	#CBE5A7	206215	125578	-153999	-113684	-153999	52216	false

	HSA 1	24591025	24716591	89388838	89455842	+	#CBE5A7	125566	67004	-113684	29668	-113684	11882	false
	HSA 1	24746259	28320865	89407265	93041451	+	#CBE5A7	3574606	3634186	29668	111408	29668	3604274	false
	HSA 1	28432273	28830421	93043425	93382554	+	#CBE5A7	398148	339129	111408	18512	111408	509556	false
	HSA 1	28848933	33087890	93434310	97512534	+	#CBE5A7	4238957	4078224	18512	57313	18512	4257469	false
	HSA 1	33145203	36064290	97485133	100179603	+	#CBE5A7	2919087	2694470	57313	53878	57313	2976400	false
	HSA 1	36118168	39771488	100191719	103632266	+	#CBE5A7	3653320	3440547	53878	21581	53878	3707198	false
	HSA 1	39793069	39877825	103584216	103665876	+	#CBE5A7	84756	81660	21581	-20319	21581	106337	false
	HSA 1	39857506	44206312	103686092	108212397	+	#CBE5A7	4348806	4526305	-20319	-30868	-20319	4328487	false
	HSA 1	44175444	46397755	108483698	110818162	+	#CBE5A7	2222311	2334464	-30868	82946	-30868	2191443	false
	HSA 1	46480701	46621584	110841035	110967312	+	#CBE5A7	140883	126277	82946	152722	82946	223829	false
	HSA 1	46774306	48434028	111113501	112818630	+	#CBE5A7	1659722	1705129	152722	-10661	152722	1812444	false
	HSA 1	48423367	48478252	112867564	112929863	+	#CBE5A7	54885	62299	-10661	7425	-10661	44224	false
	HSA 1	48485677	48578864	112795518	112853597	+	#CBE5A7	93187	58079	7425	-87323	7425	100612	false
	HSA 1	48491541	55009868	112868493	119489836	+	#CBE5A7	6518327	6621343	-87323	-105974	-87323	6431004	false
	HSA 1	54903894	55080681	119519215	119662899	+	#CBE5A7	176787	143684	-105974	119924	-105974	70813	false
	HSA 1	55200605	55413498	119664507	119830094	-	#CBE5A7	212893	165587	119924	24495	119924	332817	false
	HSA 1	55437993	55860618	119905260	120319916	+	#CBE5A7	422625	414656	24495	-342118	24495	447120	false
	HSA 1	55518500	55624488	149366549	149471552	-	#CBE5A7	105988	105003	-342118	-105988	-342118	-236130	true
	HSA 1	55518500	55699657	146149145	146328865	+	#CBE5A7	181157	179720	-105988	-181157	-105988	75169	false
	HSA 1	55518500	55966728	148600087	149034987	+	#CBE5A7	448228	434900	-181157	-248542	-181157	267071	false
	HSA 1	55718186	55966728	120486878	120705588	-	#CBE5A7	248542	218710	-248542	10267	-248542	0	true
	HSA 1	55976995	56118050	147101796	147296618	+	#CBE5A7	141055	194822	10267	59329	10267	151322	false
	HSA 1	56177379	56315294	147330999	147438838	+	#CBE5A7	137915	107839	59329	75979	59329	197244	false
	HSA 1	56391273	56912213	147485533	148009863	+	#CBE5A7	520940	524330	75979	-62380	75979	596919	false
	HSA 1	56849833	57272116	145600531	146050941	+	#CBE5A7	422283	450410	-62380	94434	-62380	359903	false
	HSA 1	57366550	57497637	121016655	121122313	+	#CBE5A7	131087	105658	94434	-131087	94434	225521	false
	HSA 1	57366550	58280054	149708470	150724703	+	#CBE5A7	913504	1016233	-131087	31643	-131087	782417	false
	HSA 1	58311697	58872376	150784477	151375388	+	#CBE5A7	560679	590911	31643	80350	31643	592322	false
	HSA 1	58952726	59365610	151398125	151862887	+	#CBE5A7	412884	464762	80350	97	80350	493234	false
	HSA 1	59365707	59544015	151862895	152074950	-	#CBE5A7	178308	212055	97	188	97	178405	false
	HSA 1	59544203	59704440	152075293	152246410	+	#CBE5A7	160237	171117	188	-14794	188	160425	false
	HSA 1	59689646	60431020	152298054	153072422	+	#CBE5A7	741374	774368	-14794	-3095	-14794	726580	false

	HSA 1	60427925	60680091	153138873	153414439	+	#CBE5A7	252166	275566	-3095	19784	-3095	249071	false
	HSA 1	60699875	60807233	157152852	157322813	-	#CBE5A7	107358	169961	19784	-107358	19784	127142	false
	HSA 1	60699875	60810934	157397525	157496542	+	#CBE5A7	111059	99017	-107358	862	-107358	3701	false
	HSA 1	60811796	61086412	155305297	155686591	+	#CBE5A7	274616	381294	862	-80340	862	275478	false
	HSA 1	61006072	62126870	155685764	157157608	+	#CBE5A7	1120798	1471844	-80340	9178	-80340	1040458	false
	HSA 1	62136048	63505227	153503215	155303978	-	#CBE5A7	1369179	1800763	9178		9178	1378357	false
**HGL 21**	HSA 7	645465	823629	87315933	87497811	-	#81A8F8	178164	181878		36			
	HSA 7	823665	1504044	86374078	87246819	-	#81A8F8	680379	872741	36	0	36	680415	false
	HSA 7	1504044	1685875	86045297	86372744	+	#81A8F8	181831	327447	0	190	0	181831	false
	HSA 7	1686065	9083675	77069779	86044390	-	#81A8F8	7397610	8974611	190	-97677	190	7397800	false
	HSA 7	8985998	9360864	102691867	103150919	+	#81A8F8	374866	459052	-97677	34592	-97677	277189	false
	HSA 7	9395456	18062911	103289100	112497210	+	#81A8F8	8667455	9208110	34592	69	34592	8702047	false
	HSA 7	18062980	27247653	12520581	22489717	+	#81A8F8	9184673	9969136	69	0	69	9184742	false
	HSA 7	27247653	34454850	22541026	29648663	+	#81A8F8	7207197	7107637	0	-25425	0	7207197	false
	HSA 7	34429425	35411650	29744111	30748704	+	#81A8F8	982225	1004593	-25425	78915	-25425	956800	false
	HSA 7	35490565	37223869	30748708	32480205	+	#81A8F8	1733304	1731497	78915	96120	78915	1812219	false
	HSA 7	37319989	42992811	128663903	134560644	-	#81A8F8	5672822	5896741	96120	17074	96120	5768942	false
	HSA 7	43009885	52780628	118845813	128460230	-	#81A8F8	9770743	9614417	17074	57490	17074	9787817	false
	HSA 7	52838118	59180201	112497276	118830002	-	#81A8F8	6342083	6332726	57490	17	57490	6399573	false
	HSA 7	59180218	61069490	10606832	12506036	-	#81A8F8	1889272	1899204	17	50160	17	1889289	false
	HSA 7	61119650	64757386	7000233	10605521	-	#81A8F8	3637736	3605288	50160	112	50160	3687896	false
	HSA 8	64749665	64831180	46544751	46628088	-	#02B4A9	81515	83337		-55685			
	HSA 7	64757498	69198002	93418876	97873787	-	#81A8F8	4440504	4454911	112	24009	112	4440616	false
	HSA 8	64775495	64856707	46696874	46759565	+	#02B4A9	81212	62691	-55685		-55685	25527	false
	HSA 7	69222011	72477407	90162634	93367683	-	#81A8F8	3255396	3205049	24009	122673	24009	3279405	false
	HSA 7	72600080	75320343	87503815	90105728	-	#81A8F8	2720263	2601913	122673	1162946	122673	2842936	false
	HSA 9	75390120	76068121	83242058	83822443	+	#FA2D80	678001	580385		692382			
	HSA 7	76483289	76638832	87503157	87601947	+	#81A8F8	155543	98790	1162946		1162946	1318489	false
	HSA 9	76760503	78774433	83832803	85748005	+	#FA2D80	2013930	1915202	692382	4428	692382	2706312	false
	HSA 9	78778861	78976561	85875264	86100765	+	#FA2D80	197700	225501	4428	16572	4428	202128	false
	HSA 9	78993133	79223136	87488206	87758330	-	#FA2D80	230003	270124	16572	3149	16572	246575	false
	HSA 9	79226285	79288268	87416183	87488185	+	#FA2D80	61983	72002	3149	0	3149	65132	false

	HSA 9	79288268	80564350	86225257	87409495	-	#FA2D80	1276082	1184238	0		0	1276082	false
**HGL 22**	HSA 1	1592409	1681873	248195548	248324489	-	#CBE5A7	89464	128941		231085			
	HSA 1	1912958	1981367	69789892	69871510	+	#CBE5A7	68409	81618	231085	10287	231085	299494	false
	HSA 1	1991654	2043236	247472850	247537099	+	#CBE5A7	51582	64249	10287		10287	61869	false
	HSA 11	3031473	3491356	62289922	63025523	-	#69E283	459883	735601		24902			
	HSA 11	3516258	3988646	61258570	62050038	+	#69E283	472388	791468	24902	34067	24902	497290	false
	HSA 11	4022713	4199616	60763285	61212656	-	#69E283	176903	449371	34067	16181	34067	210970	false
	HSA 11	4215797	4408356	60362252	60703625	-	#69E283	192559	341373	16181	74793	16181	208740	false
	HSA 11	4483149	4604495	56160655	56292424	-	#69E283	121346	131769	74793	356612	74793	196139	false
	HSA 11	4961107	5050137	60245161	60345052	+	#69E283	89030	99891	356612	64547	356612	445642	false
	HSA 11	5114684	5398602	59929350	60245111	-	#69E283	283918	315761	64547	31138	64547	348465	false
	HSA 11	5429740	5958529	59140042	59876897	-	#69E283	528789	736855	31138	27217	31138	559927	false
	HSA 11	5985746	6315538	58440452	58771648	-	#69E283	329792	331196	27217	505504	27217	357009	false
	HSA 11	6821042	7711183	57294363	58404488	-	#69E283	890141	1110125	505504	21370	505504	1395645	false
	HSA 11	7732553	8915825	56314100	57241109	-	#69E283	1183272	927009	21370	-25862	21370	1204642	false
	HSA 11	8889963	8994065	56188657	56298288	+	#69E283	104102	109631	-25862	676591	-25862	78240	false
	HSA 11	9670656	9826729	56023765	56106101	+	#69E283	156073	82336	676591	597016	676591	832664	false
	HSA 11	10423745	10526048	55794144	55935722	-	#69E283	102303	141578	597016	71157	597016	699319	false
	HSA 11	10597205	10719306	55602889	55727262	-	#69E283	122101	124373	71157	119694	71157	193258	false
	HSA 11	10839000	10982941	55453866	55575025	-	#69E283	143941	121159	119694	107286	119694	263635	false
	HSA 11	11090227	11223136	55314998	55473678	-	#69E283	132909	158680	107286	-132909	107286	240195	false
	HSA 11	11090227	11344601	49901753	50060782	+	#69E283	254374	159029	-132909	-180372	-132909	121465	false
	HSA 11	11164229	11290603	54579791	54668266	-	#69E283	126374	88475	-180372	-126374	-180372	-53998	true
	HSA 11	11164229	11303077	48356402	48497313	-	#69E283	138848	140911	-126374	61536	-126374	12474	false
	HSA 11	11364613	20283783	37490103	48266949	-	#69E283	8919170	10776846	61536	80875	61536	8980706	false
	HSA 11	20364658	23916032	33808338	37446763	-	#69E283	3551374	3638425	80875	9991	80875	3632249	false
	HSA 11	23926023	24031672	33696438	33794986	+	#69E283	105649	98548	9991	10174	9991	115640	false
	HSA 11	24041846	24978522	32809111	33743103	-	#69E283	936676	933992	10174	194498	10174	946850	false
	HSA 11	25173020	26605541	1620561	3239511	+	#69E283	1432521	1618950	194498	26252	194498	1627019	false
	HSA 11	26631793	27042752	71055662	71513052	-	#69E283	410959	457390	26252	3360	26252	437211	false
	HSA 11	27046112	29473595	67992983	70955499	-	#69E283	2427483	2962516	3360	29603	3360	2430843	false
	HSA 11	29503198	31792009	65170118	67678162	-	#69E283	2288811	2508044	29603	81361	29603	2318414	false

	HSA 11	31873370	33245877	63574249	65138293	-	#69E283	1372507	1564044	81361		81361	1453868	false
	HSA 9	33329101	35216687	94557424	96319054	+	#FA2D80	1887586	1761630		18983			
	HSA 9	35235670	35599562	96316731	96670546	-	#FA2D80	363892	353815	18983	18682677	18983	382875	false
	HSA 6	35611514	36580760	56423621	57436697	+	#564208	969246	1013076		95525			
	HSA 6	36676285	36848424	57531473	57663378	+	#564208	172139	131905	95525	38765	95525	267664	false
	HSA 6	36887189	37050970	60613814	60793721	+	#564208	163781	179907	38765	-99100	38765	202546	false
	HSA 6	36951870	37051222	57967053	58042502	+	#564208	99352	75449	-99100	408975	-99100	252	false
	HSA 6	37460197	38645789	61537971	62834249	+	#564208	1185592	1296278	408975	22464	408975	1594567	false
	HSA 6	38668253	38728549	62895730	62984060	+	#564208	60296	88330	22464	80890	22464	82760	false
	HSA 6	38809439	42968664	63000300	67843072	+	#564208	4159225	4842772	80890	30800	80890	4240115	false
	HSA 6	42999464	44799704	67932276	69806771	+	#564208	1800240	1874495	30800	462	30800	1831040	false
	HSA 6	44800166	47965803	69857592	73231973	+	#564208	3165637	3374381	462	17658	462	3166099	false
	HSA 6	47983461	48069973	75146681	75286726	+	#564208	86512	140045	17658	6417	17658	104170	false
	HSA 6	48076390	49310267	78833651	80148526	-	#564208	1233877	1314875	6417	95842	6417	1240294	false
	HSA 6	49406109	49548304	78478772	78581925	+	#564208	142195	103153	95842	0	95842	238037	false
	HSA 6	49548304	54236282	73392037	78477974	-	#564208	4687978	5085937	0		0	4687978	false
	HSA 9	54282239	54347329	97028601	97088795	-	#FA2D80	65090	60194	18682677	100968	18682677	18747767	false
	HSA 9	54448297	54580791	94342341	94461857	+	#FA2D80	132494	119516	100968	213383	100968	233462	false
	HSA 7	54693556	54745663	142311041	142443157	+	#81A8F8	52107	132116		-41522			
	HSA 7	54704141	55044974	142443841	142864473	+	#81A8F8	340833	420632	-41522	0	-41522	299311	false
	HSA 9	54794174	54929102	33614228	33698500	+	#FA2D80	134928	84272	213383		213383	348311	false
	HSA 7	55044974	55201915	142837295	143015437	-	#81A8F8	156941	178142	0	20320	0	156941	false
	HSA 7	55222235	56087057	142879273	143569308	+	#81A8F8	864822	690035	20320	-53230	20320	885142	false
	HSA 7	56033827	56111681	143717719	143807701	-	#81A8F8	77854	89982	-53230	-10	-53230	24624	false
	HSA 7	56111671	56295539	143818997	144053939	+	#81A8F8	183868	234942	-10	26563	-10	183858	false
	HSA 7	56322102	56382417	144186942	144262850	-	#81A8F8	60315	75908	26563	-46249	26563	86878	false
	HSA 7	56336168	60935028	144303898	149595540	+	#81A8F8	4598860	5291642	-46249	-87405	-46249	4552611	false
	HSA 7	60847623	61088922	149615394	149888344	+	#81A8F8	241299	272950	-87405	0	-87405	153894	false
	HSA 7	61088922	62840291	150320762	152640163	+	#81A8F8	1751369	2319401	0	841	0	1751369	false
	HSA 7	62841132	62928122	152696774	152762347	+	#81A8F8	86990	65573	841	54915	841	87831	false
	HSA 7	62983037	63623730	152850721	153705627	+	#81A8F8	640693	854906	54915	90497	54915	695608	false
	HSA 7	63714227	63883483	153820256	153957484	+	#81A8F8	169256	137228	90497	10305	90497	259753	false

	HSA 7	63893788	63962383	149978144	150068223	-	#81A8F8	68595	90079	10305	-68595	10305	78900	false
	HSA 7	63893788	67144732	154032442	159175861	+	#81A8F8	3250944	5143419	-68595		-68595	3182349	false
**HGL 23**	HSA 18	18919	693401	12784656	13919461	-	#0800AC	674482	1134805		200			
	HSA 18	693601	943544	12247127	12727412	-	#0800AC	249943	480285	200	38380	200	250143	false
	HSA 18	981924	1136032	11678683	11911428	-	#0800AC	154108	232745	38380	69	38380	192488	false
	HSA 18	1136101	1290178	11237419	11562202	-	#0800AC	154077	324783	69	-37780	69	154146	false
	HSA 18	1252398	1669966	10663472	11235152	-	#0800AC	417568	571680	-37780	358	-37780	379788	false
	HSA 18	1670324	1769691	10431574	10555928	-	#0800AC	99367	124354	358	338	358	99725	false
	HSA 18	1770029	1901223	10264240	10405741	+	#0800AC	131194	141501	338	10673	338	131532	false
	HSA 18	1911896	8686726	1364762	10252909	-	#0800AC	6774830	8888147	10673	2775	10673	6785503	false
	HSA 18	8689501	8879318	1158957	1360777	+	#0800AC	189817	201820	2775	0	2775	192592	false
	HSA 18	8879318	9774911	112548	1147557	-	#0800AC	895593	1035009	0	-8329	0	895593	false
	HSA 18	9766582	17713002	20949687	29931017	+	#0800AC	7946420	8981330	-8329	3106	-8329	7938091	false
	HSA 18	17716108	17861722	29933687	30085409	-	#0800AC	145614	151722	3106	17978	3106	148720	false
	HSA 18	17879700	19349562	30085757	31698626	+	#0800AC	1469862	1612869	17978	6360	17978	1487840	false
	HSA 18	19355922	26901292	31750508	39763125	+	#0800AC	7545370	8012617	6360	41600	6360	7551730	false
	HSA 18	26942892	31481146	39818637	44324993	+	#0800AC	4538254	4506356	41600	26151	41600	4579854	false
	HSA 18	31507297	33960381	44381397	46959525	+	#0800AC	2453084	2578128	26151	10837	26151	2479235	false
	HSA 18	33971218	36392546	47036742	49494341	+	#0800AC	2421328	2457599	10837	64721	10837	2432165	false
	HSA 18	36457267	41690675	49510196	54527560	+	#0800AC	5233408	5017364	64721	-31325	64721	5298129	false
	HSA 18	41659350	54113354	54527635	66364848	+	#0800AC	12454004	11837213	-31325	55529	-31325	12422679	false
	HSA 18	54168883	57103094	66406939	68945169	+	#0800AC	2934211	2538230	55529	162	55529	2989740	false
	HSA 18	57103256	69220696	68997946	80046988	+	#0800AC	12117440	11049042	162	57018	162	12117602	false
	HSA 18	69277714	69470893	80064936	80253023	+	#0800AC	193179	188087	57018		57018	250197	false
**HGL 24**	HSA 2	42028	835814	240711430	241889761	-	#4C0851	793786	1178331		1738			
	HSA 2	837552	6840653	232432078	240646317	-	#4C0851	6003101	8214239	1738	-1119919	1738	6004839	false
	HSA 2	5720734	5962844	233619604	233675883	-	#4C0851	242110	56279	-1119919	802358	-1119919	-877809	true
	HSA 2	6765202	7351865	231592498	232353496	-	#4C0851	586663	760998	802358	24	802358	1389021	false
	HSA 2	7351889	8072307	230369475	231536383	-	#4C0851	720418	1166908	24	-101897	24	720442	false
	HSA 2	7970410	28472242	208916774	230400023	-	#4C0851	20501832	21483249	-101897	6007	-101897	20399935	false
	HSA 2	28478249	28540501	208714424	208839989	-	#4C0851	62252	125565	6007	-866	6007	68259	false
	HSA 2	28539635	28640737	208581257	208704760	-	#4C0851	101102	123503	-866	17803	-866	100236	false

	HSA 2	28658540	35824115	201778160	208499029	-	#4C0851	7165575	6720869	17803		17803	7183378	false
	HSA 7	35936360	36078888	87500139	87621254	-	#81A8F8	142528	121115		94043			
	HSA 7	36172931	36627140	134503076	134990113	+	#81A8F8	454209	487037	94043	7596	94043	548252	false
	HSA 7	36634736	39477688	135138373	138044555	+	#81A8F8	2842952	2906182	7596	54237	7596	2850548	false
	HSA 7	39531925	43583762	138066027	142289663	+	#81A8F8	4051837	4223636	54237		54237	4106074	false
	HSA 9	43585302	45786812	88085921	90172851	-	#FA2D80	2201510	2086930		20742			
	HSA 9	45807554	46366277	92237366	92775086	+	#FA2D80	558723	537720	20742	29777	20742	579465	false
	HSA 9	46396054	46697088	92944590	93321320	+	#FA2D80	301034	376730	29777	81066	29777	330811	false
	HSA 9	46778154	47332644	93423222	93985444	+	#FA2D80	554490	562222	81066	45276	81066	635556	false
	HSA 9	47377920	47552132	94011040	94214148	-	#FA2D80	174212	203108	45276	40022	45276	219488	false
	HSA 9	47592154	47652502	38652408	38726908	+	#FA2D80	60348	74500	40022	1116	40022	100370	false
	HSA 9	47653618	48896862	90247132	91464196	+	#FA2D80	1243244	1217064	1116	15920	1116	1244360	false
	HSA 9	48912782	49019942	91474518	91547027	-	#FA2D80	107160	72509	15920	288	15920	123080	false
	HSA 9	49020230	49654100	91554410	92122135	+	#FA2D80	633870	567725	288		288	634158	false
**HGL 25**	HSA 1	39669	144403	224543883	224810562	+	#CBE5A7	104734	266679		2311			
	HSA 1	146714	599465	225328484	225857191	+	#CBE5A7	452751	528707	2311	975	2311	455062	false
	HSA 1	600440	901908	224042288	224538635	-	#CBE5A7	301468	496347	975	9269	975	302443	false
	HSA 1	911177	1030468	223608972	223847471	-	#CBE5A7	119291	238499	9269	97	9269	128560	false
	HSA 1	1030565	1599278	222521051	223558898	-	#CBE5A7	568713	1037847	97	4057	97	568810	false
	HSA 1	1603335	6702515	215540672	222447648	-	#CBE5A7	5099180	6906976	4057	769	4057	5103237	false
	HSA 1	6703284	9812043	211512057	215458564	-	#CBE5A7	3108759	3946507	769	45420	769	3109528	false
	HSA 1	9857463	10766163	241995645	243024826	+	#CBE5A7	908700	1029181	45420	24239	45420	954120	false
	HSA 1	10790402	14278463	243113687	246962717	+	#CBE5A7	3488061	3849030	24239	12485	24239	3512300	false
	HSA 1	14290948	14774108	226916393	227463930	-	#CBE5A7	483160	547537	12485	28354	12485	495645	false
	HSA 1	14802462	16454894	240013784	241873643	-	#CBE5A7	1652432	1859859	28354	40820	28354	1680786	false
	HSA 1	16495714	17051642	171038789	171776538	+	#CBE5A7	555928	737749	40820	90	40820	596748	false
	HSA 1	17051732	17117518	212657509	212813153	+	#CBE5A7	65786	155644	90	14442	90	65876	false
	HSA 1	17131960	22964230	171781138	178150938	+	#CBE5A7	5832270	6369800	14442	2553	14442	5846712	false
	HSA 1	22966783	24081325	178208985	179379078	+	#CBE5A7	1114542	1170093	2553	11454	2553	1117095	false
	HSA 1	24092779	24327497	179480889	179704560	+	#CBE5A7	234718	223671	11454	5032	11454	246172	false
	HSA 1	24332529	27034228	179742138	182449483	-	#CBE5A7	2701699	2707345	5032	544	5032	2706731	false
	HSA 1	27034772	39087261	182564806	194221593	+	#CBE5A7	12052489	11656787	544	114232	544	12053033	false

	HSA 1	39201493	39285256	194222570	194325039	-	#CBE5A7	83763	102469	114232	174	114232	197995	false
	HSA 1	39285430	41288729	194399446	196748034	+	#CBE5A7	2003299	2348588	174	1439	174	2003473	false
	HSA 1	41290168	41352165	196935323	197024723	-	#CBE5A7	61997	89400	1439	232	1439	63436	false
	HSA 1	41352397	44707827	197033033	200622446	+	#CBE5A7	3355430	3589413	232	76039	232	3355662	false
	HSA 1	44783866	45989215	200622446	201975171	+	#CBE5A7	1205349	1352725	76039	54558	76039	1281388	false
	HSA 1	46043773	49394472	201980238	205851601	+	#CBE5A7	3350699	3871363	54558	11004	54558	3405257	false
	HSA 1	49405476	50746902	205908526	207665664	+	#CBE5A7	1341426	1757138	11004	-1146052	11004	1352430	false
	HSA 1	49600850	49816431	121150379	121391072	+	#CBE5A7	215581	240693	-1146052	844623	-1146052	-930471	true
	HSA 1	50661054	53984480	207666868	211493072	+	#CBE5A7	3323426	3826204	844623	3827	844623	4168049	false
	HSA 1	53988307	54742351	225917581	226831819	-	#CBE5A7	754044	914238	3827		3827	757871	false
**HGL 26**	HSA 4	16224	614965	112714484	113396998	-	#11559D	598741	682514		13226			
	HSA 4	628191	3521131	108772673	112663775	-	#11559D	2892940	3891102	13226	55204	13226	2906166	false
	HSA 4	3576335	10289923	99349860	108771533	-	#11559D	6713588	9421673	55204	58617	55204	6768792	false
	HSA 4	10348540	10408517	99221303	99308150	-	#11559D	59977	86847	58617		58617	118594	false
	HSA 15	10557474	15839137	56357668	62240950	+	#AF0926	5281663	5883282		5213			
	HSA 15	15844350	18215376	62304058	64954222	+	#AF0926	2371026	2650164	5213	152964	5213	2376239	false
	HSA 15	18368340	24258440	64958289	72378933	+	#AF0926	5890100	7420644	152964	709	152964	6043064	false
	HSA 15	24259149	24383960	72454458	72600528	+	#AF0926	124811	146070	709	20153	709	125520	false
	HSA 15	24404113	25482318	72692903	74051581	+	#AF0926	1078205	1358678	20153	0	20153	1098358	false
	HSA 15	25482318	25945110	74120388	74679001	+	#AF0926	462792	558613	0	407	0	462792	false
	HSA 15	25945517	26151537	74762260	75051177	+	#AF0926	206020	288917	407	9458	407	206427	false
	HSA 15	26160995	26261363	75151231	75251562	+	#AF0926	100368	100331	9458	-39850	9458	109826	false
	HSA 15	26221513	26559970	75335956	75736397	+	#AF0926	338457	400441	-39850	-35512	-39850	298607	false
	HSA 15	26524458	28219386	75798079	77895321	-	#AF0926	1694928	2097242	-35512	-8089	-35512	1659416	false
	HSA 15	28211297	28751990	77967764	78713719	-	#AF0926	540693	745955	-8089	-24397	-8089	532604	false
	HSA 15	28727593	28779863	78757719	78813026	+	#AF0926	52270	55307	-24397	9732933	-24397	27873	false
	HSA 14	29023213	29141697	105619437	105749167	+	#E181CE	118484	129730		-45080			
	HSA 14	29096617	29311730	105763627	106014601	+	#E181CE	215113	250974	-45080		-45080	170033	false
	HSA 2	31128447	31399143	37055	684748	+	#4C0851	270696	647693	23295619	488	23295619	23566315	false
	HSA 2	31399631	31506193	1038494	1548270	-	#4C0851	106562	509776	488	69248	488	107050	false
	HSA 2	31575441	35814001	1581812	9090765	-	#4C0851	4238560	7508953	69248	15736	69248	4307808	false
	HSA 2	35829737	36148379	9201139	9768457	+	#4C0851	318642	567318	15736	154187	15736	334378	false

	HSA 2	36302566	36826043	9839659	11074848	+	#4C0851	523477	1235189	154187	5313	154187	677664	false
	HSA 2	36831356	40635545	11133044	15863551	+	#4C0851	3804189	4730507	5313	37099	5313	3809502	false
	HSA 2	40672644	41239835	16066193	16770796	+	#4C0851	567191	704603	37099		37099	604290	false
**HGL 27**	HSA 10	179490	533742	15027158	15414481	-	#7AB506	354252	387323		0			
	HSA 10	533742	1876105	13314774	14973481	-	#7AB506	1342363	1658707	0	69618	0	1342363	false
	HSA 10	1945723	2548885	28644655	29400317	+	#7AB506	603162	755662	69618	-72113	69618	672780	false
	HSA 10	2476772	2648007	30409946	30617880	-	#7AB506	171235	207934	-72113	16738	-72113	99122	false
	HSA 10	2664745	3329283	29443875	30271812	-	#7AB506	664538	827937	16738	-104485	16738	681276	false
	HSA 10	3224798	4796775	30651149	32420888	+	#7AB506	1571977	1769739	-104485	14838	-104485	1467492	false
	HSA 10	4811613	5812389	12058685	13298547	+	#7AB506	1000776	1239862	14838	92867	14838	1015614	false
	HSA 10	5905256	6903611	27486990	28636818	+	#7AB506	998355	1149828	92867	13576	92867	1091222	false
	HSA 10	6917187	8808363	32895390	35220422	-	#7AB506	1891176	2325032	13576	17770	13576	1904752	false
	HSA 10	8826133	10257159	35227153	36879500	-	#7AB506	1431026	1652347	17770		17770	1448796	false
	HSA 1	10341494	11411078	169903385	170930989	+	#CBE5A7	1069584	1027604	4472983	51335	4472983	5542567	false
	HSA 1	11462413	11581910	170131082	170361123	-	#CBE5A7	119497	230041	51335	51817	51335	170832	false
	HSA 1	11633727	14733332	166758201	170012691	-	#CBE5A7	3099605	3254490	51817	-82496	51817	3151422	false
	HSA 1	14650836	19479676	161591116	166705127	-	#CBE5A7	4828840	5114011	-82496	-84792	-82496	4746344	false
	HSA 1	19394884	19487884	161503374	161611328	-	#CBE5A7	93000	107954	-84792	23002	-84792	8208	false
	HSA 1	19510886	19934984	160947907	161429252	-	#CBE5A7	424098	481345	23002	-11435	23002	447100	false
	HSA 1	19923549	21817283	158988571	160891843	-	#CBE5A7	1893734	1903272	-11435	-65099	-11435	1882299	false
	HSA 1	21752184	21856384	158872359	159001859	-	#CBE5A7	104200	129500	-65099	-104200	-65099	39101	false
	HSA 1	21752184	22055015	158718800	158917810	-	#CBE5A7	302831	199010	-104200	44368	-104200	198631	false
	HSA 1	22099383	22241238	158517282	158713315	+	#CBE5A7	141855	196033	44368	49439	44368	186223	false
	HSA 1	22290677	22613369	157783591	158149865	-	#CBE5A7	322692	366274	49439	9226	49439	372131	false
	HSA 1	22622595	22798482	157489663	157692861	-	#CBE5A7	175887	203198	9226		9226	185113	false
	HSA 19	22869462	23437254	28098099	28737000	-	#60765F	567792	638901		106533			
	HSA 19	23543787	27089589	28818818	32686674	+	#60765F	3545802	3867856	106533	65115	106533	3652335	false
	HSA 19	27154704	27516192	32686687	33125842	+	#60765F	361488	439155	65115	5052	65115	426603	false
	HSA 19	27521244	28077979	34913786	35645883	-	#60765F	556735	732097	5052	176117	5052	561787	false
	HSA 19	28254096	29456883	33183404	34506075	-	#60765F	1202787	1322671	176117	34590	176117	1378904	false
	HSA 19	29491473	30032125	35645967	36240348	+	#60765F	540652	594381	34590	30	34590	575242	false
	HSA 19	30032155	30165826	36544311	36769453	-	#60765F	133671	225142	30	60065	30	133701	false

	HSA 19	30225891	30332706	36337707	36531492	-	#60765F	106815	193785	60065	24893	60065	166880	false
	HSA 19	30357599	30457220	36887552	37005910	-	#60765F	99621	118358	24893	334	24893	124514	false
	HSA 19	30457554	30646358	37361454	37552292	-	#60765F	188804	190838	334	331	334	189138	false
	HSA 19	30646689	30734358	37073387	37268133	-	#60765F	87669	194746	331	36800	331	88000	false
	HSA 19	30771158	30869354	37593726	37692461	+	#60765F	98196	98735	36800	5658	36800	134996	false
	HSA 19	30875012	30952971	37696336	37780764	-	#60765F	77959	84428	5658	33258	5658	83617	false
	HSA 19	30986229	31845883	37884926	38989986	+	#60765F	859654	1105060	33258	57680	33258	892912	false
	HSA 19	31903563	32438815	39021863	39607819	+	#60765F	535252	585956	57680	4757	57680	592932	false
	HSA 19	32443572	33183367	39793767	40864182	+	#60765F	739795	1070415	4757	60260	4757	744552	false
	HSA 19	33243627	33307305	40811705	40884447	-	#60765F	63678	72742	60260	-6379	60260	123938	false
	HSA 19	33300926	33354563	40989949	41065705	+	#60765F	53637	75756	-6379	180642	-6379	47258	false
	HSA 19	33535205	33729820	40920115	41010131	-	#60765F	194615	90016	180642	272513	180642	375257	false
	HSA 19	34002333	34245033	41138218	41449754	+	#60765F	242700	311536	272513	856	272513	515213	false
	HSA 19	34245889	34895848	41710721	42519576	-	#60765F	649959	808855	856	-15891	856	650815	false
	HSA 19	34879957	35328680	43222764	43881462	+	#60765F	448723	658698	-15891	18344	-15891	432832	false
	HSA 19	35347024	35546349	44106311	44530016	+	#60765F	199325	423705	18344	427	18344	217669	false
	HSA 19	35546776	35932382	44586544	45183787	+	#60765F	385606	597243	427	220935	427	386033	false
	HSA 19	36153317	36365261	45208272	45507531	+	#60765F	211944	299259	220935	44995	220935	432879	false
	HSA 19	36410256	36847674	45473820	46029963	+	#60765F	437418	556143	44995	38300	44995	482413	false
	HSA 19	36885974	37980803	46263972	47853055	+	#60765F	1094829	1589083	38300	251098	38300	1133129	false
	HSA 19	38231901	38413374	47990809	48204432	+	#60765F	181473	213623	251098	179071	251098	432571	false
	HSA 19	38592445	39774039	48265680	50067931	+	#60765F	1181594	1802251	179071	3940	179071	1360665	false
	HSA 19	39777979	40452756	50143635	51179719	+	#60765F	674777	1036084	3940	-49700	3940	678717	false
	HSA 19	40403056	40680495	51183910	51566456	+	#60765F	277439	382546	-49700	-74164	-49700	227739	false
	HSA 19	40606331	40834308	51541585	51797541	+	#60765F	227977	255956	-74164	-75955	-74164	153813	false
	HSA 19	40758353	40989669	51796018	51996896	+	#60765F	231316	200878	-75955	19615	-75955	155361	false
	HSA 19	41009284	41083901	52177811	52246345	-	#60765F	74617	68534	19615	28861	19615	94232	false
	HSA 19	41112762	41181454	53300117	53376270	+	#60765F	68692	76153	28861	-43210	28861	97553	false
	HSA 19	41138244	41189067	53068896	53151254	-	#60765F	50823	82358	-43210	-48983	-43210	7613	false
	HSA 19	41140084	41191779	52315293	52369621	+	#60765F	51695	54328	-48983	888	-48983	2712	false
	HSA 19	41192667	41290736	53199767	53283877	-	#60765F	98069	84110	888	-38817	888	98957	false
	HSA 19	41251919	41332262	53203746	53273303	+	#60765F	80343	69557	-38817	153058	-38817	41526	false

	HSA 19	41485320	41704663	53860545	54291036	+	#60765F	219343	430491	153058	19372	153058	372401	false
	HSA 19	41724035	41789569	54354082	54474628	+	#60765F	65534	120546	19372	62830	19372	84906	false
	HSA 19	41852399	42329564	55012180	55696882	+	#60765F	477165	684702	62830	128163	62830	539995	false
	HSA 19	42457727	42550060	56086963	56315735	+	#60765F	92333	228772	128163	6750	128163	220496	false
	HSA 19	42556810	42630913	56379025	56483409	-	#60765F	74103	104384	6750	180188	6750	80853	false
	HSA 19	42811101	42894610	56765768	56881101	+	#60765F	83509	115333	180188	71221	180188	263697	false
	HSA 19	42965831	43123667	57230910	57379830	+	#60765F	157836	148920	71221	-24232	71221	229057	false
	HSA 19	43099435	43222534	57394793	57499089	+	#60765F	123099	104296	-24232	18583	-24232	98867	false
	HSA 19	43241117	43408663	57571034	57753972	+	#60765F	167546	182938	18583	106925	18583	186129	false
	HSA 19	43515588	43570143	57940871	58003098	+	#60765F	54555	62227	106925	57174	106925	161480	false
	HSA 19	43627317	44037548	58011031	58564480	+	#60765F	410231	553449	57174		57174	467405	false
**HGL 28**	HSA 14	25874	688448	104507045	105531522	-	#E181CE	662574	1024477		45271			
	HSA 14	733719	1520731	103322383	104449762	-	#E181CE	787012	1127379	45271	23271	45271	832283	false
	HSA 14	1544002	3446247	100642507	103157355	-	#E181CE	1902245	2514848	23271	99785	23271	1925516	false
	HSA 14	3546032	7102435	96279451	100642507	-	#E181CE	3556403	4363056	99785	635	99785	3656188	false
	HSA 14	7103070	7267271	94367134	94569619	+	#E181CE	164201	202485	635	-35658	635	164836	false
	HSA 14	7231613	8551991	94574932	96267779	+	#E181CE	1320378	1692847	-35658	43217	-35658	1284720	false
	HSA 14	8595208	10717264	91955805	94374699	+	#E181CE	2122056	2418894	43217	3796	43217	2165273	false
	HSA 14	10721060	30622253	70581171	91998737	-	#E181CE	19901193	21417566	3796	99080	3796	19904989	false
	HSA 14	30721333	34333543	66754262	70423657	-	#E181CE	3612210	3669395	99080	13782	99080	3711290	false
	HSA 14	34347325	37443978	63589891	66692778	-	#E181CE	3096653	3102887	13782	54139	13782	3110435	false
	HSA 14	37498117	40919934	60165993	63560183	-	#E181CE	3421817	3394190	54139	91762	54139	3475956	false
	HSA 14	41011696	46254903	55266489	60191475	-	#E181CE	5243207	4924986	91762		91762	5334969	false
**HGL 29**	HSA 19	401166	760655	17048448	17734573	-	#60765F	359489	686125		1794			
	HSA 19	762449	1157674	16059663	16893903	-	#60765F	395225	834240	1794	162742	1794	397019	false
	HSA 19	1320416	2336154	17747343	19551453	+	#60765F	1015738	1804110	162742	22	162742	1178480	false
	HSA 19	2336176	2387377	19613005	19664846	+	#60765F	51201	51841	22	425920	22	51223	false
	HSA 19	2813297	3298573	7075077	7748188	+	#60765F	485276	673111	425920	2771	425920	911196	false
	HSA 19	3301344	3878398	7816575	8718076	+	#60765F	577054	901501	2771	93887	2771	579825	false
	HSA 19	3972285	4061160	8807449	8871672	+	#60765F	88875	64223	93887	-37750	93887	182762	false
	HSA 19	4023410	4073479	8871454	8945737	+	#60765F	50069	74283	-37750	7978	-37750	12319	false
	HSA 19	4081457	4169901	8997662	9085695	+	#60765F	88444	88033	7978	33996	7978	96422	false

	HSA 19	4203897	4393347	9378027	9493837	+	#60765F	189450	115810	33996	-92620	33996	223446	false
	HSA 19	4300727	4472109	9414919	9491398	+	#60765F	171382	76479	-92620	-114043	-92620	78762	false
	HSA 19	4358066	4462766	9611407	9665130	+	#60765F	104700	53723	-114043	59343	-114043	-9343	true
	HSA 19	4522109	4574493	9464049	9585290	-	#60765F	52384	121241	59343	18675	59343	111727	false
	HSA 19	4593168	5796770	9804712	11561477	+	#60765F	1203602	1756765	18675	93423	18675	1222277	false
	HSA 19	5890193	5954764	6951332	7001825	+	#60765F	64571	50493	93423	31208	93423	157994	false
	HSA 19	5985972	6151232	6352704	6605837	+	#60765F	165260	253133	31208	34154	31208	196468	false
	HSA 19	6185386	6478610	6546732	6943702	+	#60765F	293224	396970	34154	36939	34154	327378	false
	HSA 19	6515549	8122192	3957201	6337718	-	#60765F	1606643	2380517	36939	607	36939	1643582	false
	HSA 19	8122799	8368935	2416235	2814775	+	#60765F	246136	398540	607	0	607	246743	false
	HSA 19	8368935	8659358	2975099	3501652	-	#60765F	290423	526553	0	68453	0	290423	false
	HSA 19	8727811	9011488	3502539	3956663	+	#60765F	283677	454124	68453	7206	68453	352130	false
	HSA 19	9018694	10558609	278727	2438843	-	#60765F	1539915	2160116	7206	216884	7206	1547121	false
	HSA 19	10775493	11017672	15638441	15781331	+	#60765F	242179	142890	216884	-147079	216884	459063	false
	HSA 19	10870593	11635893	14931265	15697094	-	#60765F	765300	765829	-147079	-697188	-147079	618221	false
	HSA 19	10938705	11025732	15930111	15992321	+	#60765F	87027	62210	-697188	939421	-697188	-610161	true
	HSA 19	11965153	12207148	14247026	14573526	-	#60765F	241995	326500	939421	308	939421	1181416	false
	HSA 19	12207456	13353665	12645087	14189075	-	#60765F	1146209	1543988	308	-17204	308	1146517	false
	HSA 19	13336461	13456390	14574263	14658686	+	#60765F	119929	84423	-17204		-17204	102725	false
**HGL 3**	HSA 7	2975	1228774	98267340	99639392	-	#81A8F8	1225799	1372052		131290			
	HSA 7	1360064	1617812	97974383	98259188	-	#81A8F8	257748	284805	131290	120130	131290	389038	false
	HSA 7	1737942	2485760	5986263	6724797	+	#81A8F8	747818	738534	120130	2899	120130	867948	false
	HSA 7	2488659	2679549	2354077	2531271	+	#81A8F8	190890	177194	2899	6	2899	193789	false
	HSA 7	2679555	2797190	2531271	2671725	-	#81A8F8	117635	140454	6	-9176	6	117641	false
	HSA 7	2788014	4697658	2672800	4964859	+	#81A8F8	1909644	2292059	-9176	613	-9176	1900468	false
	HSA 7	4698271	5309679	5174189	5849055	+	#81A8F8	611408	674866	613	-59483	613	612021	false
	HSA 7	5250196	5414058	5030316	5161137	-	#81A8F8	163862	130821	-59483	45583	-59483	104379	false
	HSA 7	5459641	7196317	31731	2355873	-	#81A8F8	1736676	2324142	45583	68281	45583	1782259	false
	HSA 7	7264598	7993814	35071652	35910181	+	#81A8F8	729216	838529	68281	-710271	68281	797497	false
	HSA 12	7278761	7412071	63559219	63725474	+	#7444DF	133310	166255		2832800			
	HSA 7	7283543	7376214	103182246	103288790	+	#81A8F8	92671	106544	-710271	592470	-710271	-617600	true
	HSA 7	7968684	10237415	35945323	38366616	+	#81A8F8	2268731	2421293	592470	6576	592470	2861201	false

	HSA 7	10243991	10311044	35051265	35185595	+	#81A8F8	67053	134330	6576	-64636	6576	73629	false
	HSA 12	10244871	10311044	63557835	63667932	+	#7444DF	66173	110097	2832800	30474500	2832800	2898973	false
	HSA 7	10246408	10312704	29651251	29742502	-	#81A8F8	66296	91251	-64636	699	-64636	1660	false
	HSA 7	10313403	10369446	38259725	38370494	+	#81A8F8	56043	110769	699	76332	699	56742	false
	HSA 7	10445778	11817787	38370535	39755333	+	#81A8F8	1372009	1384798	76332	533	76332	1448341	false
	HSA 7	11818320	15610921	39883585	43808010	+	#81A8F8	3792601	3924425	533	189232	533	3793134	false
	HSA 7	15800153	15939583	99704619	99866637	-	#81A8F8	139430	162018	189232	-88307	189232	328662	false
	HSA 7	15851276	16248787	99817533	100137734	+	#81A8F8	397511	320201	-88307	51519	-88307	309204	false
	HSA 7	16300306	16366142	100139614	100215613	+	#81A8F8	65836	75999	51519	17495	51519	117355	false
	HSA 7	16383637	17812938	100372308	102326821	+	#81A8F8	1429301	1954513	17495	2603	17495	1446796	false
	HSA 7	17815541	17943710	102387694	102526158	+	#81A8F8	128169	138464	2603	22641	2603	130772	false
	HSA 7	17966351	18219998	76178017	76516704	-	#81A8F8	253647	338687	22641	0	22641	276288	false
	HSA 7	18219998	18614325	75530458	76070349	-	#81A8F8	394327	539891	0	23644	0	394327	false
	HSA 7	18637969	18736167	72928219	72991477	+	#81A8F8	98198	63258	23644	-92477	23644	121842	false
	HSA 7	18643690	19687299	73286443	74852673	+	#81A8F8	1043609	1566230	-92477	-99649	-92477	951132	false
	HSA 7	19587650	19776402	74923630	75219536	-	#81A8F8	188752	295906	-99649	20009	-99649	89103	false
	HSA 7	19796411	24136230	67339922	72605335	+	#81A8F8	4339819	5265413	20009	101718	20009	4359828	false
	HSA 7	24237948	24377299	66918434	67088547	-	#81A8F8	139351	170113	101718	2196	101718	241069	false
	HSA 7	24379495	24442341	66418650	66469974	+	#81A8F8	62846	51324	2196	-62846	2196	65042	false
	HSA 7	24379495	24448899	66740247	66813390	-	#81A8F8	69404	73143	-62846	33499	-62846	6558	false
	HSA 7	24482398	24642770	66137929	66363875	-	#81A8F8	160372	225946	33499	30640	33499	193871	false
	HSA 7	24673410	24846456	65726831	65976373	-	#81A8F8	173046	249542	30640	-60188	30640	203686	false
	HSA 7	24786268	24985018	55795285	56114934	-	#81A8F8	198750	319649	-60188	44722	-60188	138562	false
	HSA 7	25029740	25111339	43866311	43960172	+	#81A8F8	81599	93861	44722	19	44722	126321	false
	HSA 7	25111358	25909601	44043900	45280882	+	#81A8F8	798243	1236982	19	44213	19	798262	false
	HSA 7	25953814	26158322	45404714	45724561	+	#81A8F8	204508	319847	44213	1	44213	248721	false
	HSA 7	26158323	26589959	45858243	46446486	+	#81A8F8	431636	588243	1	-43926	1	431637	false
	HSA 7	26546033	26608602	46413887	46505002	-	#81A8F8	62569	91115	-43926	14828	-43926	18643	false
	HSA 7	26623430	27303262	46540421	47590041	+	#81A8F8	679832	1049620	14828	0	14828	694660	false
	HSA 7	27303262	29811754	47645642	51358487	+	#81A8F8	2508492	3712845	0	18257	0	2508492	false
	HSA 7	29830011	31102708	51449148	53183412	+	#81A8F8	1272697	1734264	18257	83087	18257	1290954	false
	HSA 7	31185795	31540845	53189424	53677142	-	#81A8F8	355050	487718	83087	1630	83087	438137	false

	HSA 7	31542475	31725446	53872797	54097278	+	#81A8F8	182971	224481	1630	69445	1630	184601	false
	HSA 7	31794891	32700556	54145114	55586310	+	#81A8F8	905665	1441196	69445		69445	975110	false
	HSA 6	32820649	33029433	104741551	104982386	-	#564208	208784	240835		180339			
	HSA 6	33209772	34329405	169344075	170601377	-	#564208	1119633	1257302	180339	63214	180339	1299972	false
	HSA 6	34392619	35334216	168150961	169344043	-	#564208	941597	1193082	63214	147042	63214	1004811	false
	HSA 6	35481258	39433616	52952805	56344605	-	#564208	3952358	3391800	147042	24603	147042	4099400	false
	HSA 6	39458219	39597662	109676141	109827977	-	#564208	139443	151836	24603	20807	24603	164046	false
	HSA 6	39618469	40499136	111250893	112044092	-	#564208	880667	793199	20807	5699086	20807	901474	false
	HSA 4	40933260	40991298	121943793	122001589	-	#11559D	58038	57796		-3356			
	HSA 4	40987942	41161240	122278132	122454120	-	#11559D	173298	175988	-3356		-3356	169942	false
	HSA 11	41996200	42133295	55598863	55727262	+	#69E283	137095	128399		828740			
	HSA 11	42962035	43060468	56023765	56123397	+	#69E283	98433	99632	828740	-66556	828740	927173	false
	HSA 11	42993912	43118170	56161456	56298266	-	#69E283	124258	136810	-66556	282667	-66556	57702	false
	HSA 11	43400837	43581869	55824671	55943604	+	#69E283	181032	118933	282667	82782	282667	463699	false
	HSA 11	43664651	43795810	56023766	56120951	-	#69E283	131159	97185	82782	358314	82782	213941	false
	HSA 11	44154124	44217302	56188000	56293168	+	#69E283	63178	105168	358314	100842	358314	421492	false
	HSA 11	44318144	44381791	54669015	54722633	-	#69E283	63647	53618	100842	75742	100842	164489	false
	HSA 11	44457533	44513996	48432331	48494434	+	#69E283	56463	62103	75742	61098	75742	132205	false
	HSA 11	44575094	44708914	56185362	56293087	-	#69E283	133820	107725	61098	-24052	61098	194918	false
	HSA 11	44684862	44952665	56310765	56379858	+	#69E283	267803	69093	-24052	-147058	-24052	243751	false
	HSA 11	44805607	44943578	56135615	56189380	-	#69E283	137971	53765	-147058	39324	-147058	-9087	true
	HSA 11	44982902	45039275	56466281	56603008	+	#69E283	56373	136727	39324	574636	39324	95697	false
	HSA 11	45613911	45715334	56023765	56125553	+	#69E283	101423	101788	574636	245267	574636	676059	false
	HSA 11	45960601	46089844	55794144	55935722	-	#69E283	129243	141578	245267		245267	374510	false
	HSA 6	46198222	48110180	114997807	116593592	-	#564208	1911958	1595785	5699086	51708	5699086	7611044	false
	HSA 6	48161888	50580150	112687031	114994077	-	#564208	2418262	2307046	51708	0	51708	2469970	false
	HSA 6	50580150	50722496	112555210	112685325	+	#564208	142346	130115	0	4219	0	142346	false
	HSA 6	50726715	51078074	112213922	112555039	-	#564208	351359	341117	4219	522	4219	355578	false
	HSA 6	51078596	51236880	112043251	112139994	-	#564208	158284	96743	522	213261	522	158806	false
	HSA 6	51450141	51666969	111057050	111266964	-	#564208	216828	209914	213261	58963	213261	430089	false
	HSA 6	51725932	52196693	110519453	111030728	-	#564208	470761	511275	58963	8529	58963	529724	false
	HSA 6	52205222	52982407	109653635	110439298	-	#564208	777185	785663	8529	117	8529	785714	false

	HSA 6	52982524	57174241	105426685	109545058	-	#564208	4191717	4118373	117	11159	117	4191834	false
	HSA 6	57185400	57236448	132784243	132841238	+	#564208	51048	56995	11159	61072	11159	62207	false
	HSA 6	57297520	57451635	133174380	133342903	+	#564208	154115	168523	61072	49644	61072	215187	false
	HSA 6	57501279	57637787	132567374	132782452	+	#564208	136508	215078	49644	1821	49644	186152	false
	HSA 6	57639608	59870662	103094121	105426685	-	#564208	2231054	2332564	1821	230	1821	2232875	false
	HSA 6	59870892	60008594	102986164	103066793	+	#564208	137702	80629	230	85805	230	137932	false
	HSA 6	60094399	67358570	96236485	102948672	-	#564208	7264171	6712187	85805	59955	85805	7349976	false
	HSA 6	67418525	68893707	95074884	96216479	-	#564208	1475182	1141595	59955	48	59955	1535137	false
	HSA 6	68893755	74272345	89881650	95020635	-	#564208	5378590	5138985	48	77408	48	5378638	false
	HSA 6	74349753	77916546	86515328	89821309	-	#564208	3566793	3305981	77408	3	77408	3644201	false
	HSA 6	77916549	78381605	85943664	86460192	-	#564208	465056	516528	3	71073	3	465059	false
	HSA 6	78452678	84360497	80093178	85880505	-	#564208	5907819	5787327	71073	64577	71073	5978892	false
	HSA 6	84425074	84621284	49553417	49790184	+	#564208	196210	236767	64577	10564	64577	260787	false
	HSA 6	84631848	84729057	49725385	49790895	-	#564208	97209	65510	10564	-20202	10564	107773	false
	HSA 6	84708855	84805337	49697786	49784863	-	#564208	96482	87077	-20202	4878	-20202	76280	false
	HSA 6	84810215	87844050	49739186	52688278	+	#564208	3033835	2949092	4878	23607	4878	3038713	false
	HSA 6	87867657	87945204	52838140	52914562	+	#564208	77547	76422	23607	14408	23607	101154	false
	HSA 6	87959612	88868199	159682124	160524411	+	#564208	908587	842287	14408	-46241	14408	922995	false
	HSA 6	88821958	88881401	160699909	160758468	-	#564208	59443	58559	-46241	-13179	-46241	13202	false
	HSA 6	88868222	91231184	160914525	163188055	+	#564208	2362962	2273530	-13179	10	-13179	2349783	false
	HSA 6	91231194	95072032	163437432	167247669	+	#564208	3840838	3810237	10	-126318	10	3840848	false
	HSA 6	94945714	95084103	167251444	167393888	-	#564208	138389	142444	-126318	8950	-126318	12071	false
	HSA 6	95093053	95252844	167398858	167588915	+	#564208	159791	190057	8950	245	8950	168741	false
	HSA 6	95253089	95611476	167642037	168060722	+	#564208	358387	418685	245	6427	245	358632	false
	HSA 6	95617903	98888102	146587894	149876545	+	#564208	3270199	3288651	6427	96022	6427	3276626	false
	HSA 6	98984124	104654410	150073626	155856514	+	#564208	5670286	5782888	96022	66308	96022	5766308	false
	HSA 6	104720718	107029815	155873386	158208015	+	#564208	2309097	2334629	66308	54112	66308	2375405	false
	HSA 6	107083927	108424848	158213919	159677318	-	#564208	1340921	1463399	54112	45768	54112	1395033	false
	HSA 6	108470616	108535644	132632349	132773040	+	#564208	65028	140691	45768	9068	45768	110796	false
	HSA 6	108544712	108599770	133241026	133300370	+	#564208	55058	59344	9068	9208	9068	64126	false
	HSA 6	108608978	120766344	133551016	146859923	+	#564208	12157366	13308907	9208	8466	9208	12166574	false
	HSA 6	120774810	121761124	132570337	133550122	-	#564208	986314	979785	8466	69073	8466	994780	false

	HSA 6	121830197	122862815	131427735	132570910	-	#564208	1032618	1143175	69073	63016	69073	1101691	false
	HSA 6	122925831	128794847	124900661	131424166	-	#564208	5869016	6523505	63016	77282	63016	5932032	false
	HSA 6	128872129	131494507	121676471	124888436	-	#564208	2622378	3211965	77282	33947	77282	2699660	false
	HSA 6	131528454	135280965	116621748	121619508	-	#564208	3752511	4997760	33947		33947	3786458	false
**HGL 4**	HSA 6	706478	813586	28430729	28593341	-	#564208	107108	162612		858			
	HSA 6	814444	951615	28209643	28367232	+	#564208	137171	157589	858	69521	858	138029	false
	HSA 6	1021136	1103588	27783610	27839142	-	#564208	82452	55532	69521	42018	69521	151973	false
	HSA 6	1145606	1253996	27420127	27620480	-	#564208	108390	200353	42018	57905	42018	150408	false
	HSA 6	1311901	1362579	27090021	27167005	-	#564208	50678	76984	57905	33185	57905	108583	false
	HSA 6	1395764	5299798	181356	5016062	+	#564208	3904034	4834706	33185	34408	33185	3937219	false
	HSA 6	5334206	5983767	5067037	5805913	+	#564208	649561	738876	34408	25535	34408	683969	false
	HSA 6	6009302	24464968	5857107	25970737	+	#564208	18455666	20113630	25535	121120	25535	18481201	false
	HSA 6	24586088	24832264	25970427	26200061	+	#564208	246176	229634	121120	-57369	121120	367296	false
	HSA 6	24774895	24877504	26199520	26349859	+	#564208	102609	150339	-57369	265735	-57369	45240	false
	HSA 1	24969060	25109470	212750000	212940216	+	#CBE5A7	140410	190216		410931			
	HSA 6	25143239	25271889	26479157	26666992	+	#564208	128650	187835	265735		265735	394385	false
	HSA 1	25520401	25693795	228661429	229163594	+	#CBE5A7	173394	502165	410931	1136	410931	584325	false
	HSA 1	25694931	29617999	229216393	234490100	+	#CBE5A7	3923068	5273707	1136	12	1136	3924204	false
	HSA 1	29618011	30109707	234491513	235094180	-	#CBE5A7	491696	602667	12	2799	12	491708	false
	HSA 1	30112506	34547257	235099440	239931861	+	#CBE5A7	4434751	4832421	2799		2799	4437550	false
	HSA 10	34595050	36976313	88592	2260811	+	#7AB506	2381263	2172219		9854			
	HSA 10	36986167	37219115	2262432	2409997	-	#7AB506	232948	147565	9854	3187	9854	242802	false
	HSA 10	37222302	38195053	2411635	3186999	+	#7AB506	972751	775364	3187	17359	3187	975938	false
	HSA 10	38212412	44714024	5875423	12093571	-	#7AB506	6501612	6218148	17359	54562	17359	6518971	false
	HSA 10	44768586	44959379	5673071	5875422	+	#7AB506	190793	202351	54562	7843	54562	245355	false
	HSA 10	44967222	45263418	5407677	5669797	-	#7AB506	296196	262120	7843		7843	304039	false
	HSA 3	45522830	46022752	126002140	126565932	-	#9B0496	499922	563792		36			
	HSA 3	46022788	55416279	66213651	75243253	+	#9B0496	9393491	9029602	36	-35979	36	9393527	false
	HSA 3	55380300	59663446	12066	3968415	+	#9B0496	4283146	3956349	-35979	57188	-35979	4247167	false
	HSA 3	59720634	61107230	3968415	5173746	+	#9B0496	1386596	1205331	57188	149069	57188	1443784	false
	HSA 3	61256299	62350110	5106937	5812915	+	#9B0496	1093811	705978	149069	20728	149069	1242880	false
	HSA 3	62370838	62470068	6041059	6116916	+	#9B0496	99230	75857	20728	7477	20728	119958	false

	HSA 3	62477545	62693960	5821127	6041039	-	#9B0496	216415	219912	7477	4165	7477	223892	false
	HSA 3	62698125	65437205	6126771	8442826	+	#9B0496	2739080	2316055	4165	-7259	4165	2743245	false
	HSA 3	65429946	65519653	38263446	38329698	+	#9B0496	89707	66252	-7259	-48308	-7259	82448	false
	HSA 3	65471345	66239492	37398814	38144059	-	#9B0496	768147	745245	-48308	550	-48308	719839	false
	HSA 3	66240042	81712759	22218039	37249418	-	#9B0496	15472717	15031379	550	56566	550	15473267	false
	HSA 3	81769325	82806645	21249107	22261033	-	#9B0496	1037320	1011926	56566	21165	56566	1093886	false
	HSA 3	82827810	82946091	21085530	21245452	+	#9B0496	118281	159922	21165	23504	21165	139446	false
	HSA 3	82969595	88506808	15553736	21081783	-	#9B0496	5537213	5528047	23504	17090	23504	5560717	false
	HSA 3	88523898	94288602	52315137	58256157	-	#9B0496	5764704	5941020	17090	43	17090	5781794	false
	HSA 3	94288645	94469750	15200927	15382870	+	#9B0496	181105	181943	43	7164	43	181148	false
	HSA 3	94476914	94696270	15402566	15611793	-	#9B0496	219356	209227	7164	25112	7164	226520	false
	HSA 3	94721382	95380061	58205577	59057111	+	#9B0496	658679	851534	25112	6602	25112	683791	false
	HSA 3	95386663	95450814	59076555	59142157	-	#9B0496	64151	65602	6602	11167	6602	70753	false
	HSA 3	95461981	98356253	59123844	62187702	+	#9B0496	2894272	3063858	11167	370	11167	2905439	false
	HSA 3	98356623	98418075	62254600	62331321	+	#9B0496	61452	76721	370	4229	370	61822	false
	HSA 3	98422304	99807948	62250917	63723958	+	#9B0496	1385644	1473041	4229	52473	4229	1389873	false
	HSA 3	99860421	102034657	63740283	66084791	+	#9B0496	2174236	2344508	52473		52473	2226709	false
**HGL 5**	HSA 5	211197	904055	179678290	180669574	-	#FDB568	692858	991284		5128			
	HSA 5	909183	1559712	178867965	179627338	-	#FDB568	650529	759373	5128	144335	5128	655657	false
	HSA 5	1704047	2146352	178064522	178634149	-	#FDB568	442305	569627	144335	30233	144335	586640	false
	HSA 5	2176585	2528019	151000851	151354939	-	#FDB568	351434	354088	30233	109	30233	381667	false
	HSA 5	2528128	5854029	131137458	134735661	+	#FDB568	3325901	3598203	109	24356	109	3326010	false
	HSA 5	5878385	6834143	151398155	152456968	+	#FDB568	955758	1058813	24356	15475	24356	980114	false
	HSA 5	6849618	11145002	152388046	157129287	+	#FDB568	4295384	4741241	15475	184	15475	4310859	false
	HSA 5	11145186	21975151	157002820	168520265	+	#FDB568	10829965	11517445	184	56559	184	10830149	false
	HSA 5	22031710	29484777	168527193	175958223	+	#FDB568	7453067	7431030	56559	111016	56559	7509626	false
	HSA 5	29595793	31019471	176291836	177613302	+	#FDB568	1423678	1321466	111016	97861	111016	1534694	false
	HSA 5	31117332	32161296	134705271	135730270	+	#FDB568	1043964	1024999	97861	136	97861	1141825	false
	HSA 5	32161432	34342322	135813256	137889254	+	#FDB568	2180890	2075998	136	6	136	2181026	false
	HSA 5	34342328	37216951	112940839	115691258	+	#FDB568	2874623	2750419	6	58630	6	2874629	false
	HSA 5	37275581	39415322	115705432	117709773	+	#FDB568	2139741	2004341	58630	16342	58630	2198371	false
	HSA 5	39431664	39660165	117723873	117957997	-	#FDB568	228501	234124	16342	30	16342	244843	false

	HSA 5	39660195	41434871	117958518	119636394	+	#FDB568	1774676	1677876	30	4079	30	1774706	false
	HSA 5	41438950	41665134	119649311	119864423	-	#FDB568	226184	215112	4079	0	4079	230263	false
	HSA 5	41665134	42274078	119864990	120374635	-	#FDB568	608944	509645	0	15987	0	608944	false
	HSA 5	42290065	42809516	120374639	120924701	+	#FDB568	519451	550062	15987	41677	15987	535438	false
	HSA 5	42851193	42972783	120975372	121071632	+	#FDB568	121590	96260	41677	50233	41677	163267	false
	HSA 5	43023016	43244194	121108154	121334625	+	#FDB568	221178	226471	50233	52006	50233	271411	false
	HSA 5	43296200	43403360	121384198	121499344	+	#FDB568	107160	115146	52006		52006	159166	false
	HSA 2	43484472	44312031	111457135	112345717	-	#4C0851	827559	888582		-61218			
	HSA 2	44250813	44312031	87437189	87493090	+	#4C0851	61218	55901	-61218	20371	-61218	0	true
	HSA 2	44332402	44483436	87551649	87695749	+	#4C0851	151034	144100	20371	-151034	20371	171405	false
	HSA 2	44332402	45110639	110636062	111399171	-	#4C0851	778237	763109	-151034	62864	-151034	627203	false
	HSA 2	45173503	45289687	110064741	110232565	-	#4C0851	116184	167824	62864	-26033	62864	179048	false
	HSA 2	45263654	45616879	94979408	95414295	+	#4C0851	353225	434887	-26033	1237	-26033	327192	false
	HSA 2	45618116	45904440	96024396	96375948	+	#4C0851	286324	351552	1237	4840	1237	287561	false
	HSA 2	45909280	46292229	96473238	96979218	+	#4C0851	382949	505980	4840	3386	4840	387789	false
	HSA 2	46295615	53493230	97643499	106203482	+	#4C0851	7197615	8559983	3386	76052	3386	7201001	false
	HSA 2	53569282	54746097	106531163	107825036	+	#4C0851	1176815	1293873	76052	1018	76052	1252867	false
	HSA 2	54747115	54979692	107924085	108187329	+	#4C0851	232577	263244	1018	71208	1018	233595	false
	HSA 2	55050900	55111707	108262294	108321975	+	#4C0851	60807	59681	71208	-4479	71208	132015	false
	HSA 2	55107228	55965584	108383386	109725990	+	#4C0851	858356	1342604	-4479	32650	-4479	853877	false
	HSA 2	55998234	56942124	112462173	113392708	+	#4C0851	943890	930535	32650	161301	32650	976540	false
	HSA 2	57103425	57198449	89085036	89176790	-	#4C0851	95024	91754	161301	-94848	161301	256325	false
	HSA 2	57103601	57198449	90046798	90115540	+	#4C0851	94848	68742	-94848	-90472	-94848	0	true
	HSA 2	57107977	57198501	90121462	90235559	+	#4C0851	90524	114097	-90472	-54485	-90472	52	false
	HSA 2	57144016	57202143	89220735	89273872	-	#4C0851	58127	53137	-54485	212614	-54485	3642	false
	HSA 2	57414757	57479647	88892443	89020624	-	#4C0851	64890	128181	212614	21562	212614	277504	false
	HSA 2	57501209	57596964	89189520	89245684	-	#4C0851	95755	56164	21562	-94837	21562	117317	false
	HSA 2	57502127	57599459	89220735	89330071	-	#4C0851	97332	109336	-94837	-66097	-94837	2495	false
	HSA 2	57533362	57596231	90157409	90235559	+	#4C0851	62869	78150	-66097	110487	-66097	-3228	true
	HSA 2	57706718	57775009	89085036	89255318	-	#4C0851	68291	170282	110487	-68291	110487	178778	false
	HSA 2	57706718	57775168	89967588	90039785	+	#4C0851	68450	72197	-68291	-68450	-68291	159	false
	HSA 2	57706718	57777354	89927118	90008969	+	#4C0851	70636	81851	-68450	-59690	-68450	2186	false

	HSA 2	57717664	57775061	90180125	90235559	+	#4C0851	57397	55434	-59690	-57397	-59690	-2293	true
	HSA 2	57717664	57777354	89020626	89078548	-	#4C0851	59690	57922	-57397	103649	-57397	2293	false
	HSA 1	57777868	57848274	179365639	179443576	+	#CBE5A7	70406	77937		382994			
	HSA 2	57881003	58004960	89169828	89245235	-	#4C0851	123957	75407	103649	-123957	103649	227606	false
	HSA 2	57881003	58027276	89116984	89176790	-	#4C0851	146273	59806	-123957	-146273	-123957	22316	false
	HSA 2	57881003	58027778	90005166	90102244	+	#4C0851	146775	97078	-146273	-146022	-146273	502	false
	HSA 2	57881756	57993560	89841770	89892686	+	#4C0851	111804	50916	-146022	-107605	-146022	-34218	true
	HSA 2	57885955	57946070	88925534	89020624	-	#4C0851	60115	95090	-107605	-56275	-107605	-47490	true
	HSA 2	57889795	58027778	89955547	90011777	+	#4C0851	137983	56230	-56275	-134149	-56275	81708	false
	HSA 2	57893629	58027778	90099086	90191252	+	#4C0851	134149	92166	-134149	-100783	-134149	0	true
	HSA 2	57926995	58025971	89048009	89100437	-	#4C0851	98976	52428	-100783	91779	-100783	-1807	true
	HSA 2	58117750	58171967	89220735	89273872	+	#4C0851	54217	53137	91779	139532	91779	145996	false
	HSA 1	58231268	58303986	179364298	179431123	-	#CBE5A7	72718	66825	382994		382994	455712	false
	HSA 2	58311499	58361533	89220735	89298946	+	#4C0851	50034	78211	139532	11085	139532	189566	false
	HSA 2	58372618	58586163	89011852	89103309	-	#4C0851	213545	91457	11085	-213545	11085	224630	false
	HSA 2	58372618	58588321	88978389	89049092	-	#4C0851	215703	70703	-213545	-174304	-213545	2158	false
	HSA 2	58414017	58636189	89232916	89316819	-	#4C0851	222172	83903	-174304	-158701	-174304	47868	false
	HSA 2	58477488	58588321	89142495	89273264	-	#4C0851	110833	130769	-158701	-72920	-158701	-47868	true
	HSA 2	58515401	58580607	89960083	90011462	+	#4C0851	65206	51379	-72920	-57109	-72920	-7714	true
	HSA 2	58523498	58588321	89192414	89252858	-	#4C0851	64823	60444	-57109	-55614	-57109	7714	false
	HSA 2	58532707	58786642	89850677	90073915	+	#4C0851	253935	223238	-55614	-120458	-55614	198321	false
	HSA 2	58666184	58786629	90010600	90073897	+	#4C0851	120445	63297	-120458	95739	-120458	-13	true
	HSA 2	58882368	58963092	88909877	88993014	+	#4C0851	80724	83137	95739	278589	95739	176463	false
	HSA 2	59241681	59967999	87997696	88755713	-	#4C0851	726318	758017	278589	-2385	278589	1004907	false
	HSA 2	59965614	65456817	80950793	86861180	-	#4C0851	5491203	5910387	-2385	52022	-2385	5488818	false
	HSA 2	65508839	67467922	79102644	80901935	-	#4C0851	1959083	1799291	52022	-66842	52022	2011105	false
	HSA 2	67401080	67502673	79028629	79160315	+	#4C0851	101593	131686	-66842	-46790	-66842	34751	false
	HSA 2	67455883	73194114	73772126	79088459	-	#4C0851	5738231	5316333	-46790	81850	-46790	5691441	false
	HSA 2	73275964	73381890	73670641	73747048	-	#4C0851	105926	76407	81850	-62038	81850	187776	false
	HSA 2	73319852	75625482	71178575	73645621	-	#4C0851	2305630	2467046	-62038	17378	-62038	2243592	false
	HSA 2	75642860	82245234	64477720	71006262	-	#4C0851	6602374	6528542	17378	2060	17378	6619752	false
	HSA 2	82247294	82312345	64420037	64475012	+	#4C0851	65051	54975	2060	20153	2060	67111	false

	HSA 2	82332498	83291649	63372376	64420037	-	#4C0851	959151	1047661	20153	31831	20153	979304	false
	HSA 2	83323480	84645926	61991103	63320788	-	#4C0851	1322446	1329685	31831	8801	31831	1354277	false
	HSA 2	84654727	85481411	61054361	61923744	-	#4C0851	826684	869383	8801	533	8801	835485	false
	HSA 2	85481944	85537363	27468829	27550117	+	#4C0851	55419	81288	533	20805	533	55952	false
	HSA 2	85558168	89682674	56575300	61053731	-	#4C0851	4124506	4478431	20805	4562	20805	4145311	false
	HSA 2	89687236	89824162	56417216	56573542	+	#4C0851	136926	156326	4562	1456	4562	141488	false
	HSA 2	89825618	92125297	54157928	56413562	-	#4C0851	2299679	2255634	1456	29197	1456	2301135	false
	HSA 2	92154494	92244817	165534166	165611855	+	#4C0851	90323	77689	29197	38991	29197	119520	false
	HSA 2	92283808	98918416	47333453	54164001	-	#4C0851	6634608	6830548	38991	60999	38991	6673599	false
	HSA 2	98979415	112549350	33404613	47330693	-	#4C0851	13569935	13926080	60999	57099	60999	13630934	false
	HSA 2	112606449	114311251	31661914	33403507	-	#4C0851	1704802	1741593	57099	1065	57099	1761901	false
	HSA 2	114312316	120503926	24784314	31586961	-	#4C0851	6191610	6802647	1065	76296	1065	6192675	false
	HSA 2	120580222	127547228	17223333	24784314	-	#4C0851	6967006	7560981	76296		76296	7043302	false
**HGL 6**	HSA 3	50282	604828	39378656	40463187	+	#9B0496	554546	1084531		6084			
	HSA 3	610912	2944185	40533501	44455754	+	#9B0496	2333273	3922253	6084	73238	6084	2339357	false
	HSA 3	3017423	4441155	44478012	46755913	+	#9B0496	1423732	2277901	73238	-2912	73238	1496970	false
	HSA 3	4438243	5454269	46829218	48199660	+	#9B0496	1016026	1370442	-2912	37275	-2912	1013114	false
	HSA 3	5491544	5579737	125528295	125629441	+	#9B0496	88193	101146	37275	5633	37275	125468	false
	HSA 3	5585370	6757048	183121751	184468598	+	#9B0496	1171678	1346847	5633	68442	5633	1177311	false
	HSA 3	6825490	15600702	184495780	194498238	+	#9B0496	8775212	10002458	68442	-37941	68442	8843654	false
	HSA 3	15562761	16359639	194541999	195593684	+	#9B0496	796878	1051685	-37941	42336	-37941	758937	false
	HSA 3	16401975	17600010	196049233	197605561	-	#9B0496	1198035	1556328	42336	314	42336	1240371	false
	HSA 3	17600324	17759798	195699516	195923257	-	#9B0496	159474	223741	314	5760	314	159788	false
	HSA 3	17765558	18069383	197667520	198044771	+	#9B0496	303825	377251	5760	97	5760	309585	false
	HSA 3	18069480	34124553	166795565	183121751	-	#9B0496	16055073	16326186	97	30381	97	16055170	false
	HSA 3	34154934	34259179	166608804	166732778	-	#9B0496	104245	123974	30381	58788	30381	134626	false
	HSA 3	34317967	37036471	163761990	166530596	-	#9B0496	2718504	2768606	58788	48199	58788	2777292	false
	HSA 3	37084670	37786437	163012979	163704520	-	#9B0496	701767	691541	48199	1910	48199	749966	false
	HSA 3	37788347	37842949	162952667	163012911	+	#9B0496	54602	60244	1910	176	1910	56512	false
	HSA 3	37843125	49603161	151719279	162944336	-	#9B0496	11760036	11225057	176	57553	176	11760212	false
	HSA 3	49660714	49733942	151694922	151804403	-	#9B0496	73228	109481	57553	-1692	57553	130781	false
	HSA 3	49732250	55673314	146377298	151757965	-	#9B0496	5941064	5380667	-1692	52634	-1692	5939372	false

	HSA 3	55725948	56858387	145281227	146284758	-	#9B0496	1132439	1003531	52634	47193	52634	1185073	false
	HSA 3	56905580	56979602	145193110	145279010	+	#9B0496	74022	85900	47193	2	47193	121215	false
	HSA 3	56979604	59993914	142296895	145193064	-	#9B0496	3014310	2896169	2	13699	2	3014312	false
	HSA 3	60007613	63273876	139033928	142241323	-	#9B0496	3266263	3207395	13699	56695	13699	3279962	false
	HSA 3	63330571	63656805	138650455	138993414	-	#9B0496	326234	342959	56695	77218	56695	382929	false
	HSA 3	63734023	69637778	132971570	138640200	-	#9B0496	5903755	5668630	77218	50351	77218	5980973	false
	HSA 3	69688129	70509260	132314972	132971536	-	#9B0496	821131	656564	50351	9486	50351	871482	false
	HSA 3	70518746	72588822	130360077	132261036	-	#9B0496	2070076	1900959	9486	2086	9486	2079562	false
	HSA 3	72590908	72678052	130217018	130282539	-	#9B0496	87144	65521	2086	62279	2086	89230	false
	HSA 3	72740331	75933575	48916299	52313051	-	#9B0496	3193244	3396752	62279	87718	62279	3255523	false
	HSA 3	76021293	76366003	48515353	48905228	-	#9B0496	344710	389875	87718	62648	87718	432428	false
	HSA 3	76428651	76549912	48344109	48509879	-	#9B0496	121261	165770	62648	10858761	62648	183909	false
	HSA 4	77063869	77301057	73671859	73926154	+	#11559D	237188	254295		52278			
	HSA 4	77353335	78865801	74156890	75826467	+	#11559D	1512466	1669577	52278	109893	52278	1564744	false
	HSA 4	78975694	79044021	75840134	75904702	-	#11559D	68327	64568	109893	95000	109893	178220	false
	HSA 4	79139021	82080913	85125960	88078355	-	#11559D	2941892	2952395	95000	54140	95000	3036892	false
	HSA 4	82135053	82321844	85015005	85122324	-	#11559D	186791	107319	54140	59297	54140	240931	false
	HSA 4	82381141	84540723	82924502	85013071	-	#11559D	2159582	2088569	59297		59297	2218879	false
	HSA 16	84590879	86378941	46578990	48166050	+	#49FEF6	1788062	1587060		170240			
	HSA 16	86549181	86929461	48210422	48615880	+	#49FEF6	380280	405458	170240		170240	550520	false
	HSA 3	87408673	87539700	12537148	12726957	+	#9B0496	131027	189809	10858761	70967	10858761	10989788	false
	HSA 3	87610667	87683707	12729675	12842398	+	#9B0496	73040	112723	70967	-5114	70967	144007	false
	HSA 3	87678593	88064828	129395050	129892378	+	#9B0496	386235	497328	-5114	64083	-5114	381121	false
	HSA 3	88128911	88736404	14109499	15102688	-	#9B0496	607493	993189	64083	62457	64083	671576	false
	HSA 3	88798861	88852676	127198146	127253943	-	#9B0496	53815	55797	62457	138	62457	116272	false
	HSA 3	88852814	89593589	126691286	127440270	+	#9B0496	740775	748984	138	74624	138	740913	false
	HSA 3	89668213	90051401	127570510	128002943	-	#9B0496	383188	432433	74624	16283	74624	457812	false
	HSA 3	90067684	90547823	128044019	128651317	+	#9B0496	480139	607298	16283	8966	16283	496422	false
	HSA 3	90556789	90665877	128667728	128807691	-	#9B0496	109088	139963	8966	11845	8966	118054	false
	HSA 3	90677722	90793858	129088386	129245128	+	#9B0496	116136	156742	11845	33886	11845	127981	false
	HSA 3	90827744	91675477	10753986	11847685	+	#9B0496	847733	1093699	33886	86469	33886	881619	false
	HSA 3	91761946	92266273	11929980	12533168	+	#9B0496	504327	603188	86469	1585	86469	590796	false

	HSA 3	92267858	92999287	12910140	13897637	+	#9B0496	731429	987497	1585		1585	733014	false
	HSA 10	93237868	102953666	15411024	26622661	+	#7AB506	9715798	11211637		-40631			
	HSA 10	102913035	103116825	26633506	26872931	+	#7AB506	203790	239425	-40631	4831	-40631	163159	false
	HSA 10	103121656	103258925	27106137	27242291	-	#7AB506	137269	136154	4831	20370	4831	142100	false
	HSA 10	103279295	104951878	3181979	4875311	+	#7AB506	1672583	1693332	20370	14479	20370	1692953	false
	HSA 10	104966357	105027575	22338665	22422681	+	#7AB506	61218	84016	14479	94257	14479	75697	false
	HSA 10	105121832	105225639	5047779	5107936	+	#7AB506	103807	60157	94257	5418	94257	198064	false
	HSA 10	105231057	105311489	5302362	5407032	+	#7AB506	80432	104670	5418		5418	85850	false
**HGL 7**	HSA 16	25963	583835	89259445	90049507	-	#49FEF6	557872	790062		14069			
	HSA 16	597904	1062843	88408712	89203087	-	#49FEF6	464939	794375	14069	23204	14069	479008	false
	HSA 16	1086047	1351991	87770631	88357389	-	#49FEF6	265944	586758	23204	40207	23204	289148	false
	HSA 16	1392198	3285807	84354352	87770398	+	#49FEF6	1893609	3416046	40207	13344	40207	1933816	false
	HSA 16	3299151	5623131	81884428	84329834	-	#49FEF6	2323980	2445406	13344	54708	13344	2337324	false
	HSA 16	5677839	12446973	74434592	81964749	-	#49FEF6	6769134	7530157	54708	-35096	54708	6823842	false
	HSA 16	12411877	12494255	70112553	70168140	-	#49FEF6	82378	55587	-35096	44412	-35096	47282	false
	HSA 16	12538667	13030726	70250981	70819448	+	#49FEF6	492059	568467	44412	35829	44412	536471	false
	HSA 16	13066555	16498097	70896912	74328227	+	#49FEF6	3431542	3431315	35829	52	35829	3467371	false
	HSA 16	16498149	19470014	66937544	69943578	-	#49FEF6	2971865	3006034	52	88136	52	2971917	false
	HSA 16	19558150	29367367	56652968	66945546	-	#49FEF6	9809217	10292578	88136	-46517	88136	9897353	false
	HSA 16	29320850	30095788	55831980	56599862	-	#49FEF6	774938	767882	-46517	64143	-46517	728421	false
	HSA 16	30159931	30349899	55736984	55833762	-	#49FEF6	189968	96778	64143	-184412	64143	254111	false
	HSA 16	30165487	30390086	55725693	55797565	+	#49FEF6	224599	71872	-184412	-79838	-184412	40187	false
	HSA 16	30310248	30488688	55725693	55831835	+	#49FEF6	178440	106142	-79838	-64000	-79838	98602	false
	HSA 16	30424688	30535215	55733273	55795317	+	#49FEF6	110527	62044	-64000	-39323	-64000	46527	false
	HSA 16	30495892	30648394	55725693	55798028	+	#49FEF6	152502	72335	-39323	1910	-39323	113179	false
	HSA 16	30650304	32727887	53427159	55718936	-	#49FEF6	2077583	2291777	1910	13358	1910	2079493	false
	HSA 16	32741245	37355642	48639697	53328031	-	#49FEF6	4614397	4688334	13358		13358	4627755	false
	HSA 4	37381410	43048493	77377198	82923099	-	#11559D	5667083	5545901		-8239			
	HSA 4	43040254	44473720	75905334	77274919	-	#11559D	1433466	1369585	-8239	125588	-8239	1425227	false
	HSA 4	44599308	44655789	88065437	88169371	+	#11559D	56481	103934	125588	60	125588	182069	false
	HSA 4	44655849	44845780	88023735	88169371	+	#11559D	189931	145636	60	60	60	189991	false
	HSA 4	44845840	44947660	88023735	88169371	+	#11559D	101820	145636	60	60	60	101880	false

	HSA 4	44947720	45289021	88023735	88333978	+	#11559D	341301	310243	60	53608	60	341361	false
	HSA 4	45342629	55338686	88363860	97890902	+	#11559D	9996057	9527042	53608	21252	53608	10049665	false
	HSA 4	55359938	55419314	97891205	97977814	-	#11559D	59376	86609	21252	6830	21252	80628	false
	HSA 4	55426144	56765431	97978024	99119403	+	#11559D	1339287	1141379	6830		6830	1346117	false
	HSA 1	56816501	59322338	13427172	16466235	+	#CBE5A7	2505837	3039063	43477812	469	43477812	45983649	false
	HSA 1	59322807	61914846	16911951	19979354	+	#CBE5A7	2592039	3067403	469	14472	469	2592508	false
	HSA 1	61929318	63136224	20055431	21387522	+	#CBE5A7	1206906	1332091	14472	2906	14472	1221378	false
	HSA 1	63139130	66260727	21486598	25248086	+	#CBE5A7	3121597	3761488	2906	-5283	2906	3124503	false
	HSA 1	66255444	81964184	25330721	43121732	+	#CBE5A7	15708740	17791011	-5283	-12000206	-5283	15703457	false
	HSA 1	69963978	70151130	30477206	30708342	-	#CBE5A7	187152	231136	-12000206	-69876	-12000206	-11813054	true
	HSA 1	70081254	70138937	30084925	30135061	-	#CBE5A7	57683	50136	-69876	15867	-69876	-12193	true
	HSA 1	70154804	70213829	30336779	30421230	-	#CBE5A7	59025	84451	15867	-37366	15867	74892	false
	HSA 1	70176463	70279321	29453730	29586183	+	#CBE5A7	102858	132453	-37366	-17201	-37366	65492	false
	HSA 1	70262120	70368888	30487853	30639866	-	#CBE5A7	106768	152013	-17201	-65910	-17201	89567	false
	HSA 1	70302978	70368902	30089111	30190141	-	#CBE5A7	65924	101030	-65910	-29677	-65910	14	false
	HSA 1	70339225	70417477	29671516	29745012	+	#CBE5A7	78252	73496	-29677	616	-29677	48575	false
	HSA 1	70418093	70474104	30465095	30538267	+	#CBE5A7	56011	73172	616	-46551	616	56627	false
	HSA 1	70427553	70483504	29650150	29727699	-	#CBE5A7	55951	77549	-46551	-34778	-46551	9400	false
	HSA 1	70448726	70682450	29743330	29960252	-	#CBE5A7	233724	216922	-34778	-129063	-34778	198946	false
	HSA 1	70553387	70739848	29328705	29602884	-	#CBE5A7	186461	274179	-129063	-61513	-129063	57398	false
	HSA 1	70678335	70936484	29790929	30050350	-	#CBE5A7	258149	259421	-61513	-199858	-61513	196636	false
	HSA 1	70736626	70940827	29516258	29733614	-	#CBE5A7	204201	217356	-199858	-109946	-199858	4343	false
	HSA 1	70830881	70934450	30199507	30319500	+	#CBE5A7	103569	119993	-109946	7811792	-109946	-6377	true
	HSA 1	78746242	78833878	39734674	39797934	-	#CBE5A7	87636	63260	7811792	150116	7811792	7899428	false
	HSA 1	78983994	79049205	39450598	39562599	-	#CBE5A7	65211	112001	150116	3139562	150116	215327	false
	HSA 1	82188767	83122798	43123639	44154633	+	#CBE5A7	934031	1030994	3139562	134350	3139562	4073593	false
	HSA 1	83257148	84230091	44154698	45121125	+	#CBE5A7	972943	966427	134350	69670	134350	1107293	false
	HSA 1	84299761	85923230	45167952	46824919	+	#CBE5A7	1623469	1656967	69670	139190	69670	1693139	false
	HSA 1	86062420	86117117	46967311	47050852	+	#CBE5A7	54697	83541	139190	28211	139190	193887	false
	HSA 1	86145328	87064656	47171410	48112525	+	#CBE5A7	919328	941115	28211	61192	28211	947539	false
	HSA 1	87125848	88659228	48216179	49907626	+	#CBE5A7	1533380	1691447	61192	3831	61192	1594572	false
	HSA 1	88663059	93067907	49964734	54642440	+	#CBE5A7	4404848	4677706	3831	917	3831	4408679	false

	HSA 1	93068824	97760369	54708817	59927569	+	#CBE5A7	4691545	5218752	917	637846	917	4692462	false
	HSA 1	98398215	101402074	8942009	12779292	-	#CBE5A7	3003859	3837283	637846	-2956565	637846	3641705	false
	HSA 1	98445509	98532444	12786317	12884323	+	#CBE5A7	86935	98006	-2956565	-86935	-2956565	-2869630	true
	HSA 1	98445509	98534697	13022387	13090988	+	#CBE5A7	89188	68601	-86935	2883020	-86935	2253	false
	HSA 1	101417717	105644816	2780551	8881496	-	#CBE5A7	4227099	6100945	2883020	247	2883020	7110119	false
	HSA 1	105645063	106279647	1699669	2647825	-	#CBE5A7	634584	948156	247	-52212	247	634831	false
	HSA 1	106227435	106798643	903444	1683185	-	#CBE5A7	571208	779741	-52212		-52212	518996	false
**HGL 8**	HSA 22	7256	2670106	47488614	50785612	-	#DB00F9	2662850	3296998		500			
	HSA 22	2670606	2769899	47363833	47479851	+	#DB00F9	99293	116018	500	1490	500	99793	false
	HSA 22	2771389	6264854	42548434	47363028	-	#DB00F9	3493465	4814594	1490	-35383	1490	3494955	false
	HSA 22	6229471	9207043	39099384	42521931	-	#DB00F9	2977572	3422547	-35383	30876	-35383	2942189	false
	HSA 22	9237919	11472675	36147074	38942282	-	#DB00F9	2234756	2795208	30876	44520	30876	2265632	false
	HSA 22	11517195	11621123	36188372	36265988	-	#DB00F9	103928	77616	44520	-98147	44520	148448	false
	HSA 22	11522976	11649840	36196910	36278586	+	#DB00F9	126864	81676	-98147	40127	-98147	28717	false
	HSA 22	11689967	12117541	35656223	36187812	-	#DB00F9	427574	531589	40127	79552920	40127	467701	false
	HSA 12	12161006	17244671	21434629	27040704	-	#7444DF	5083665	5606075		94054			
	HSA 12	17338725	17501852	20931225	21120620	+	#7444DF	163127	189395	94054	-163127	94054	257181	false
	HSA 12	17338725	17629514	21110030	21430338	+	#7444DF	290789	320308	-163127	6441	-163127	127662	false
	HSA 12	17635955	17688747	20958905	21090912	-	#7444DF	52792	132007	6441	-52245	6441	59233	false
	HSA 12	17636502	20462965	17861566	20918563	-	#7444DF	2826463	3056997	-52245	0	-52245	2774218	false
	HSA 12	20462965	20523505	17774401	17853926	+	#7444DF	60540	79525	0	0	0	60540	false
	HSA 12	20523505	23735677	14237648	17764355	-	#7444DF	3212172	3526707	0	72148	0	3212172	false
	HSA 12	23807825	26357762	11393979	14213249	-	#7444DF	2549937	2819270	72148	-38427	72148	2622085	false
	HSA 12	26319335	26399402	11162019	11249069	-	#7444DF	80067	87050	-38427	260093	-38427	41640	false
	HSA 12	26659495	26754816	10837878	10919728	+	#7444DF	95321	81850	260093	48002	260093	355414	false
	HSA 12	26802818	27063161	10037823	10315710	+	#7444DF	260343	277887	48002	41874	48002	308345	false
	HSA 12	27105035	27488816	10386309	10837834	+	#7444DF	383781	451525	41874	386636	41874	425655	false
	HSA 12	27875452	28136906	7882827	8131501	-	#7444DF	261454	248674	386636	-123466	386636	648090	false
	HSA 12	28013440	28225969	7648337	7854536	-	#7444DF	212529	206199	-123466	159	-123466	89063	false
	HSA 12	28226128	28376295	8608595	8841248	+	#7444DF	150167	232653	159	10371	159	150326	false
	HSA 12	28386666	28540486	8899638	9134785	+	#7444DF	153820	235147	10371	14448	10371	164191	false
	HSA 12	28554934	28625063	9052994	9113797	-	#7444DF	70129	60803	14448	14252	14448	84577	false

	HSA 12	28639315	28703254	9480090	9565913	-	#7444DF	63939	85823	14252	-63939	14252	78191	false
	HSA 12	28639315	28715650	31124760	31211945	+	#7444DF	76335	87185	-63939	65419	-63939	12396	false
	HSA 12	28781069	28834796	9720419	9800349	+	#7444DF	53727	79930	65419	136467	65419	119146	false
	HSA 12	28971263	29095059	8452104	8555864	+	#7444DF	123796	103760	136467	1484	136467	260263	false
	HSA 12	29096543	29176983	7446180	7602483	-	#7444DF	80440	156303	1484	170647	1484	81924	false
	HSA 12	29347630	33033506	2788219	7221607	-	#7444DF	3685876	4433388	170647		170647	3856523	false
	HSA 11	33693586	41453612	127785088	134973894	-	#69E283	7760026	7188806		52217			
	HSA 11	41505829	43449377	125850495	127721840	-	#69E283	1943548	1871345	52217	33505	52217	1995765	false
	HSA 11	43482882	46904613	120507601	123811607	+	#69E283	3421731	3304006	33505	-10595	33505	3455236	false
	HSA 11	46894018	47442055	123837046	124211481	+	#69E283	548037	374435	-10595	10643	-10595	537442	false
	HSA 11	47452698	47663940	124146213	124302204	+	#69E283	211242	155991	10643	29841	10643	221885	false
	HSA 11	47693781	47878422	124162323	124299953	+	#69E283	184641	137630	29841	65947	29841	214482	false
	HSA 11	47944369	48122734	124320529	124399440	+	#69E283	178365	78911	65947	241471	65947	244312	false
	HSA 11	48364205	48617249	124392010	124551799	+	#69E283	253044	159789	241471	111029	241471	494515	false
	HSA 11	48728278	49867478	124536930	125679809	+	#69E283	1139200	1142879	111029	4222	111029	1250229	false
	HSA 11	49871700	55639906	114649936	120457239	-	#69E283	5768206	5807303	4222	7221	4222	5772428	false
	HSA 11	55647127	55706656	114531151	114598421	-	#69E283	59529	67270	7221	-47014	7221	66750	false
	HSA 11	55659642	58188715	112279534	114533000	-	#69E283	2529073	2253466	-47014	66249	-47014	2482059	false
	HSA 11	58254964	65499269	105359230	112279534	-	#69E283	7244305	6920304	66249	973	66249	7310554	false
	HSA 11	65500242	65556946	105298409	105356021	+	#69E283	56704	57612	973	0	973	57677	false
	HSA 11	65556946	65931803	105000109	105298409	-	#69E283	374857	298300	0	-153508	0	374857	false
	HSA 11	65778295	80190543	90347640	104954021	-	#69E283	14412248	14606381	-153508	9321	-153508	14258740	false
	HSA 11	80199864	80351614	90080105	90290277	-	#69E283	151750	210172	9321	110944	9321	161071	false
	HSA 11	80462558	80520666	49146016	49208984	+	#69E283	58108	62968	110944	54329	110944	169052	false
	HSA 11	80574995	80760597	89405820	89614203	-	#69E283	185602	208383	54329	86473	54329	239931	false
	HSA 11	80847070	80913812	49381720	49459671	+	#69E283	66742	77951	86473	-66742	86473	153215	false
	HSA 11	80847070	81117904	89032733	89324456	-	#69E283	270834	291723	-66742	173974	-66742	204092	false
	HSA 11	81291878	84432748	85552240	88856240	-	#69E283	3140870	3304000	173974	38407	173974	3314844	false
	HSA 11	84471155	87350066	82634335	85460902	-	#69E283	2878911	2826567	38407	28734	38407	2917318	false
	HSA 11	87378800	87446841	82547219	82605783	+	#69E283	68041	58564	28734	62214	28734	96775	false
	HSA 11	87509055	93097336	76829384	82547219	-	#69E283	5588281	5717835	62214	196522	62214	5650495	false
	HSA 11	93293858	97397275	72515009	76828869	-	#69E283	4103417	4313860	196522	105802	196522	4299939	false

	HSA 11	97503077	98015495	71915623	72485575	-	#69E283	512418	569952	105802	2	105802	618220	false
	HSA 11	98015497	98577719	3605106	4194647	+	#69E283	562222	589541	2	1568	2	562224	false
	HSA 11	98579287	99283219	4374307	5110272	+	#69E283	703932	735965	1568	294238	1568	705500	false
	HSA 11	99577457	100300002	5129938	5804082	+	#69E283	722545	674144	294238	88007	294238	1016783	false
	HSA 11	100388009	100532522	5855241	5993400	+	#69E283	144513	138159	88007	-53585	88007	232520	false
	HSA 11	100478937	101195301	5996183	6747596	+	#69E283	716364	751413	-53585	66684	-53585	662779	false
	HSA 11	101261985	101436865	6708914	6787759	+	#69E283	174880	78845	66684	82234	66684	241564	false
	HSA 11	101519099	102432479	6788549	7730325	+	#69E283	913380	941776	82234	137078	82234	995614	false
	HSA 11	102569557	112394983	7727100	18118624	+	#69E283	9825426	10391524	137078	4496	137078	9962504	false
	HSA 11	112399479	112894977	18223302	18806863	+	#69E283	495498	583561	4496	916	4496	499994	false
	HSA 11	112895893	113002936	18948269	19023605	+	#69E283	107043	75336	916	-7076	916	107959	false
	HSA 11	112995860	113079803	18801169	18957739	+	#69E283	83943	156570	-7076	96208	-7076	76867	false
	HSA 11	113176011	122001293	18958157	30169720	+	#69E283	8825282	11211563	96208	-4383173	96208	8921490	false
	HSA 11	117618120	117686072	23730472	23788163	+	#69E283	67952	57691	-4383173	4271311	-4383173	-4315221	true
	HSA 11	121957383	123839539	30169419	32796426	+	#69E283	1882156	2627007	4271311	29989	4271311	6153467	false
	HSA 11	123869528	124122159	1176768	1577388	-	#69E283	252631	400620	29989	-36630	29989	282620	false
	HSA 11	124085529	124921250	192132	1176931	-	#69E283	835721	984799	-36630		-36630	799091	false
**HGL 9**	HSA 20	23444	3571015	41325742	45139333	+	#007FFF	3547571	3813591		80423			
	HSA 20	3651438	3912152	45175963	45447628	+	#007FFF	260714	271665	80423	81473	80423	341137	false
	HSA 20	3993625	4118426	45455585	45663622	+	#007FFF	124801	208037	81473	3832	81473	206274	false
	HSA 20	4122258	15521043	45720564	59521485	+	#007FFF	11398785	13800921	3832	-9378462	3832	11402617	false
	HSA 20	6142581	6289486	47789330	48077981	-	#007FFF	146905	288651	-9378462	9300489	-9378462	-9231557	true
	HSA 20	15589975	19574076	59545620	64276314	+	#007FFF	3984101	4730694	9300489	14032647	9300489	13284590	false
	HSA 11	19649394	19798540	56066570	56124970	+	#69E283	149146	58400		337845			
	HSA 11	20136385	20265535	55453866	55575025	-	#69E283	129150	121159	337845	4017385	337845	466995	false
	HSA 1	20617413	20769615	247470360	247537099	-	#CBE5A7	152202	66739		549768			
	HSA 1	21319383	21420391	247470276	247532026	-	#CBE5A7	101008	61750	549768	490407	549768	650776	false
	HSA 1	21910798	21975001	247678655	247742255	-	#CBE5A7	64203	63600	490407	-13921	490407	554610	false
	HSA 1	21961080	22043855	247671286	247742255	+	#CBE5A7	82775	70969	-13921	323075	-13921	68854	false
	HSA 1	22366930	22476017	247834885	247920412	-	#CBE5A7	109087	85527	323075	156747	323075	432162	false
	HSA 1	22632764	22733827	69495768	69583370	-	#CBE5A7	101063	87602	156747	2060584	156747	257810	false
	HSA 11	24282920	24385562	55655513	55726784	+	#69E283	102642	71271	4017385	32191	4017385	4120027	false

	HSA 11	24417753	24472038	55794144	55935722	+	#69E283	54285	141578	32191	1368254	32191	86476	false
	HSA 1	24794411	24965050	247418208	247480951	-	#CBE5A7	170639	62743	2060584	519670	2060584	2231223	false
	HSA 1	25484720	25659269	247470276	247537099	+	#CBE5A7	174549	66823	519670	1360442	519670	694219	false
	HSA 11	25840292	25990131	55453866	55575025	+	#69E283	149839	121159	1368254	399608	1368254	1518093	false
	HSA 11	26389739	26496645	56048136	56125553	-	#69E283	106906	77417	399608		399608	506514	false
	HSA 1	27019711	27116375	247929746	248025250	+	#CBE5A7	96664	95504	1360442	48935	1360442	1457106	false
	HSA 1	27165310	27245797	248212892	248279052	-	#CBE5A7	80487	66160	48935		48935	129422	false
	HSA 19	27585782	27657304	14858044	14928910	-	#60765F	71522	70866		1979573			
	HSA 19	29636877	29786630	14868976	14928910	-	#60765F	149753	59934	1979573	-98898	1979573	2129326	false
	HSA 19	29687732	29788140	14841615	14928910	+	#60765F	100408	87295	-98898		-98898	1510	false
	HSA 20	33606723	40388951	34234277	41324190	-	#007FFF	6782228	7089913	14032647	12670	14032647	20814875	false
	HSA 20	40401621	42993673	31306223	34182548	-	#007FFF	2592052	2876325	12670	6060	12670	2604722	false
	HSA 20	42999733	43639099	90257	740907	+	#007FFF	639366	650650	6060	3270	6060	645426	false
	HSA 20	43642369	43772963	872937	967957	-	#007FFF	130594	95020	3270	10293	3270	133864	false
	HSA 20	43783256	43933987	748400	986780	+	#007FFF	150731	238380	10293	258	10293	161024	false
	HSA 20	43934245	44009899	877152	992672	+	#007FFF	75654	115520	258	0	258	75912	false
	HSA 20	44009899	44078137	872937	992672	+	#007FFF	68238	119735	0	0	0	68238	false
	HSA 20	44078137	44764897	872937	1521883	+	#007FFF	686760	648946	0	-25310	0	686760	false
	HSA 20	44739587	44804150	1531460	1620369	+	#007FFF	64563	88909	-25310	145521	-25310	39253	false
	HSA 20	44949671	61009115	1763854	18215836	+	#007FFF	16059444	16451982	145521	43690	145521	16204965	false
	HSA 20	61052805	65065827	18380772	23553663	+	#007FFF	4013022	5172891	43690	546	43690	4056712	false
	HSA 20	65066373	65152193	56459890	56594731	+	#007FFF	85820	134841	546	27422	546	86366	false
	HSA 20	65179615	65231631	23827348	23880542	+	#007FFF	52016	53194	27422	-52016	27422	79438	false
	HSA 20	65179615	65231642	23639141	23689874	+	#007FFF	52027	50733	-52016	124615	-52016	11	false
	HSA 20	65356257	66255657	24044983	25616584	+	#007FFF	899400	1571601	124615		124615	1024015	false
	HSA 15	66346946	66732423	78864613	79341278	+	#AF0926	385477	476665		54441			
	HSA 15	66786864	67219622	79388150	79907354	+	#AF0926	432758	519204	54441	-15911	54441	487199	false
	HSA 15	67203711	67475606	79952019	80319984	+	#AF0926	271895	367965	-15911	112489	-15911	255984	false
	HSA 15	67588095	68571596	80325748	81589007	+	#AF0926	983501	1263259	112489	71491	112489	1095990	false
	HSA 15	68643087	69228848	81658442	82277023	+	#AF0926	585761	618581	71491	-90720	71491	657252	false
	HSA 15	69138128	70561144	82536567	84150119	-	#AF0926	1423016	1613552	-90720	2336	-90720	1332296	false
	HSA 15	70563480	71091849	84598234	85156765	-	#AF0926	528369	558531	2336	40	2336	530705	false

	HSA 15	71091889	71698491	90315186	91025013	+	#AF0926	606602	709827	40	786	40	606642	false
	HSA 15	71699277	76271971	85286423	90273577	-	#AF0926	4572694	4987154	786	-9716	786	4573480	false
	HSA 15	76262255	77935332	91048557	92722307	+	#AF0926	1673077	1673750	-9716	4131	-9716	1663361	false
	HSA 15	77939463	83213998	92780609	98536051	+	#AF0926	5274535	5755442	4131	0	4131	5278666	false
	HSA 15	83213998	83641006	98537145	99023290	-	#AF0926	427008	486145	0	11	0	427008	false
	HSA 15	83641017	84257615	99024233	99754865	+	#AF0926	616598	730632	11	7114	11	616609	false
	HSA 15	84264729	85817212	99921358	101732197	+	#AF0926	1552483	1810839	7114	0	7114	1559597	false
	HSA 15	85817212	86920042	28861005	30076423	-	#AF0926	1102830	1215418	0	51059	0	1102830	false
	HSA 15	86971101	88099224	30899423	32261428	+	#AF0926	1128123	1362005	51059	-77114	51059	1179182	false
	HSA 15	88022110	88099224	30277229	30376921	-	#AF0926	77114	99692	-77114	145794	-77114	0	true
	HSA 15	88245018	88626104	23552701	23925966	+	#AF0926	381086	373265	145794	-101049	145794	526880	false
	HSA 15	88525055	88641579	24388981	24442841	+	#AF0926	116524	53860	-101049	113003	-101049	15475	false
	HSA 15	88754582	89050213	24846093	25167128	+	#AF0926	295631	321035	113003	-27252	113003	408634	false
	HSA 15	89022961	89344924	25264668	25557391	+	#AF0926	321963	292723	-27252	600	-27252	294711	false
	HSA 15	89345524	91664173	25623425	28323528	+	#AF0926	2318649	2700103	600	113	600	2319249	false
	HSA 15	91664286	91885732	22785177	23042601	+	#AF0926	221446	257424	113		113	221559	false
	HSA 7	91902934	92053268	32867550	33064608	-	#81A8F8	150334	197058	84145840	810	84145840	84296174	false
	HSA 7	92054078	92123842	32475965	32597071	-	#81A8F8	69764	121106	810	38984	810	70574	false
	HSA 7	92162826	92259369	33180532	33334295	+	#81A8F8	96543	153763	38984	12205	38984	135527	false
	HSA 7	92271574	92332931	35007658	35073302	-	#81A8F8	61357	65644	12205	41167	12205	73562	false
	HSA 7	92374098	93007115	34133253	34950485	-	#81A8F8	633017	817232	41167	317	41167	674184	false
	HSA 7	93007432	93176203	33538462	33820546	-	#81A8F8	168771	282084	317	43098	317	169088	false
	HSA 7	93219301	93742221	33128419	33614329	-	#81A8F8	522920	485910	43098	21837	43098	566018	false
	HSA 7	93764058	93871691	99685823	99800656	-	#81A8F8	107633	114833	21837		21837	129470	false
**HGL X**	HSA X	265040	324585	283173	463087	+	#01FC33	59545	179914		3			
	HSA X	324588	420432	617118	686760	+	#01FC33	95844	69642	3	0	3	95847	false
	HSA X	420432	496368	755496	831507	+	#01FC33	75936	76011	0	167534	0	75936	false
	HSA X	663902	757185	1570783	1643140	+	#01FC33	93283	72357	167534	214829	167534	260817	false
	HSA X	972014	1035591	2301934	2427281	+	#01FC33	63577	125347	214829	5194	214829	278406	false
	HSA X	1040785	1182094	2486528	2613174	+	#01FC33	141309	126646	5194	-25971	5194	146503	false
	HSA X	1156123	1217945	2678733	2740773	+	#01FC33	61822	62040	-25971	15607	-25971	35851	false
	HSA X	1233552	1393713	2833167	3033931	+	#01FC33	160161	200764	15607	17179	15607	175768	false

	HSA X	1410892	1750252	3189940	3758449	+	#01FC33	339360	568509	17179	201078	17179	356539	false
	HSA X	1951330	2261306	4004093	4499820	+	#01FC33	309976	495727	201078	11245	201078	511054	false
	HSA X	2272551	2705598	4564535	5143800	+	#01FC33	433047	579265	11245	410070	11245	444292	false
	HSA X	3115668	6456293	5195840	8901937	+	#01FC33	3340625	3706097	410070	179920	410070	3750695	false
	HSA X	6636213	6700401	8900614	8960151	+	#01FC33	64188	59537	179920	31991	179920	244108	false
	HSA X	6732392	7504989	9034860	9949472	+	#01FC33	772597	914612	31991	155221	31991	804588	false
	HSA X	7660210	10652475	9964485	12904548	+	#01FC33	2992265	2940063	155221	61893	155221	3147486	false
	HSA X	10714368	13670627	12904581	15699768	+	#01FC33	2956259	2795187	61893	-110468	61893	3018152	false
	HSA X	13560159	13817591	15668179	15932396	+	#01FC33	257432	264217	-110468	0	-110468	146964	false
	HSA X	13817591	13895905	15932418	16006522	-	#01FC33	78314	74104	0	49	0	78314	false
	HSA X	13895954	14132002	16006747	16217088	+	#01FC33	236048	210341	49	5222	49	236097	false
	HSA X	14137224	14230268	16236858	16321408	-	#01FC33	93044	84550	5222	0	5222	98266	false
	HSA X	14230268	21643626	16327827	23415180	+	#01FC33	7413358	7087353	0	9205	0	7413358	false
	HSA X	21652831	21744484	23415180	23504569	-	#01FC33	91653	89389	9205	48083	9205	100858	false
	HSA X	21792567	21873501	23504753	23560084	-	#01FC33	80934	55331	48083	74678	48083	129017	false
	HSA X	21948179	25598918	23633192	27330296	+	#01FC33	3650739	3697104	74678	-24560	74678	3725417	false
	HSA X	25574358	28480329	27330296	30245853	+	#01FC33	2905971	2915557	-24560	118804	-24560	2881411	false
	HSA X	28599133	32616398	30220887	34116023	+	#01FC33	4017265	3895136	118804	16143	118804	4136069	false
	HSA X	32632541	34649776	34171790	35788607	+	#01FC33	2017235	1616817	16143	-1033732	16143	2033378	false
	HSA X	33616044	33770010	36787167	36870486	-	#01FC33	153966	83319	-1033732	38678	-1033732	-879766	true
	HSA X	33808688	34067010	36478190	36678475	-	#01FC33	258322	200285	38678	9476	38678	297000	false
	HSA X	34076486	34549633	36155099	36413250	-	#01FC33	473147	258151	9476	60190	9476	482623	false
	HSA X	34609823	34778230	35769150	35860700	-	#01FC33	168407	91550	60190	-36	60190	228597	false
	HSA X	34778194	35580491	35856892	36582461	+	#01FC33	802297	725569	-36	-569173	-36	802261	false
	HSA X	35011318	35114652	35637986	35714798	-	#01FC33	103334	76812	-569173	17028	-569173	-465839	true
	HSA X	35131680	35395773	35386416	35579774	-	#01FC33	264093	193358	17028	61514	17028	281121	false
	HSA X	35457287	35564814	35240829	35347563	-	#01FC33	107527	106734	61514	15856	61514	169041	false
	HSA X	35580670	35634034	36643257	36722598	+	#01FC33	53364	79341	15856	-51252	15856	69220	false
	HSA X	35582782	35763357	34891951	35130897	-	#01FC33	180575	238946	-51252	-124497	-51252	129323	false
	HSA X	35638860	35825129	36783269	36903447	+	#01FC33	186269	120178	-124497	-61361	-124497	61772	false
	HSA X	35763768	35859439	34762154	34813647	-	#01FC33	95671	51493	-61361	2458	-61361	34310	false
	HSA X	35861897	36001513	34656044	34809867	-	#01FC33	139616	153823	2458	-139616	2458	142074	false

	HSA X	35861897	36008602	36879390	36966884	+	#01FC33	146705	87494	-139616	178	-139616	7089	false
	HSA X	36008780	36211879	37291193	37443025	-	#01FC33	203099	151832	178	62767	178	203277	false
	HSA X	36274646	36408741	36968330	37064308	-	#01FC33	134095	95978	62767	-134089	62767	196862	false
	HSA X	36274652	44599685	37443076	45328349	+	#01FC33	8325033	7885273	-134089	146870	-134089	8190944	false
	HSA X	44746555	44812077	45425158	45475739	+	#01FC33	65522	50581	146870	50276	146870	212392	false
	HSA X	44862353	47075644	45499062	47301499	+	#01FC33	2213291	1802437	50276	349200	50276	2263567	false
	HSA X	47424844	47635588	47492935	47663945	+	#01FC33	210744	171010	349200	31225	349200	559944	false
	HSA X	47666813	47928174	47778394	48033258	+	#01FC33	261361	254864	31225	95949	31225	292586	false
	HSA X	48024123	48097780	48043404	48166579	+	#01FC33	73657	123175	95949	-1299	95949	169606	false
	HSA X	48096481	48922319	48391758	49346447	+	#01FC33	825838	954689	-1299	37966	-1299	824539	false
	HSA X	48960285	49512986	49670859	50145367	+	#01FC33	552701	474508	37966	125191	37966	590667	false
	HSA X	49638177	50038839	50280289	50527724	+	#01FC33	400662	247435	125191	91243	125191	525853	false
	HSA X	50130082	50676485	50538703	50936219	+	#01FC33	546403	397516	91243	128819	91243	637646	false
	HSA X	50805304	51029665	50871558	51056431	+	#01FC33	224361	184873	128819	7578	128819	353180	false
	HSA X	51037243	51110018	51209671	51279081	+	#01FC33	72775	69410	7578	270363	7578	80353	false
	HSA X	51380381	51460088	51467592	51526322	+	#01FC33	79707	58730	270363	64998	270363	350070	false
	HSA X	51525086	51594856	51681146	51736266	+	#01FC33	69770	55120	64998	-57203	64998	134768	false
	HSA X	51537653	51616336	51721763	51779876	-	#01FC33	78683	58113	-57203	-39856	-57203	21480	false
	HSA X	51576480	51908218	51735612	51957649	+	#01FC33	331738	222037	-39856	25849	-39856	291882	false
	HSA X	51934067	52065598	52074084	52154436	-	#01FC33	131531	80352	25849	80205	25849	157380	false
	HSA X	52145803	52218303	52913158	52992742	-	#01FC33	72500	79584	80205	49354	80205	152705	false
	HSA X	52267657	52357263	52887570	52940917	-	#01FC33	89606	53347	49354	-77124	49354	138960	false
	HSA X	52280139	53048522	52911480	53716484	+	#01FC33	768383	805004	-77124	442334	-77124	691259	false
	HSA X	53490856	54322716	53929217	54420890	+	#01FC33	831860	491673	442334	77902	442334	1274194	false
	HSA X	54400618	54550329	54439869	54563139	+	#01FC33	149711	123270	77902	231793	77902	227613	false
	HSA X	54782122	54842524	54773498	54844186	+	#01FC33	60402	70688	231793	-60402	231793	292195	false
	HSA X	54782122	54870211	54723470	54842724	-	#01FC33	88089	119254	-60402	167214	-60402	27687	false
	HSA X	55037425	55236038	54898285	55035798	+	#01FC33	198613	137513	167214	158276	167214	365827	false
	HSA X	55394314	55455509	52290848	52340868	-	#01FC33	61195	50020	158276	51260	158276	219471	false
	HSA X	55506769	55606366	55716729	55804750	+	#01FC33	99597	88021	51260	284245	51260	150857	false
	HSA X	55890611	56019014	56224067	56302272	+	#01FC33	128403	78205	284245	282445	284245	412648	false
	HSA X	56301459	56358281	56529384	56592049	+	#01FC33	56822	62665	282445	334385	282445	339267	false

	HSA X	56692666	56757777	56727804	56820474	+	#01FC33	65111	92670	334385	256524	334385	399496	false
	HSA X	57014301	57102559	57308907	57434098	-	#01FC33	88258	125191	256524	852875	256524	344782	false
	HSA X	57955434	58276237	63632161	63818699	+	#01FC33	320803	186538	852875	218206	852875	1173678	false
	HSA X	58494443	58700651	64178588	64274194	+	#01FC33	206208	95606	218206	0	218206	424414	false
	HSA X	58700651	58770125	64325074	64409275	+	#01FC33	69474	84201	0	7180	0	69474	false
	HSA X	58777305	58913013	64881650	64993425	+	#01FC33	135708	111775	7180	63880	7180	142888	false
	HSA X	58976893	59102166	65013842	65114429	+	#01FC33	125273	100587	63880	334095	63880	189153	false
	HSA X	59436261	59749337	65476176	65747482	+	#01FC33	313076	271306	334095	1294	334095	647171	false
	HSA X	59750631	59825596	65830350	65896232	+	#01FC33	74965	65882	1294	49917	1294	76259	false
	HSA X	59875513	60065815	65972336	66116959	+	#01FC33	190302	144623	49917	52102	49917	240219	false
	HSA X	60117917	60282684	66147141	66302407	+	#01FC33	164767	155266	52102	51660	52102	216869	false
	HSA X	60334344	60445469	66379478	66478223	+	#01FC33	111125	98745	51660	123053	51660	162785	false
	HSA X	60568522	60757490	66589898	66816140	+	#01FC33	188968	226242	123053	61970	123053	312021	false
	HSA X	60819460	60936926	66868917	66921495	-	#01FC33	117466	52578	61970	55415	61970	179436	false
	HSA X	60992341	61071031	67017410	67081329	+	#01FC33	78690	63919	55415	299992	55415	134105	false
	HSA X	61371023	61466880	67257697	67351785	+	#01FC33	95857	94088	299992	196019	299992	395849	false
	HSA X	61662899	62137385	67520132	67814121	+	#01FC33	474486	293989	196019	332558	196019	670505	false
	HSA X	62469943	63294851	67933506	68552990	+	#01FC33	824908	619484	332558	133029	332558	1157466	false
	HSA X	63427880	65017299	68583066	70057280	+	#01FC33	1589419	1474214	133029	83819	133029	1722448	false
	HSA X	65101118	65598757	70082633	70563603	+	#01FC33	497639	480970	83819	16077	83819	581458	false
	HSA X	65614834	65899113	70617276	70745468	+	#01FC33	284279	128192	16077	60991	16077	300356	false
	HSA X	65960104	66318733	70804616	71024142	+	#01FC33	358629	219526	60991	50696	60991	419620	false
	HSA X	66369429	67090612	71029960	71661441	+	#01FC33	721183	631481	50696	-19293	50696	771879	false
	HSA X	67071319	68132284	71857657	72731426	+	#01FC33	1060965	873769	-19293	916494	-19293	1041672	false
	HSA X	69048778	69110375	73689693	73792534	+	#01FC33	61597	102841	916494	177240	916494	978091	false
	HSA X	69287615	69354743	73938126	73997827	+	#01FC33	67128	59701	177240	226723	177240	244368	false
	HSA X	69581466	69790663	74212963	74325534	+	#01FC33	209197	112571	226723	220275	226723	435920	false
	HSA X	70010938	70237699	74410591	74538124	-	#01FC33	226761	127533	220275	122466	220275	447036	false
	HSA X	70360165	70510322	74535651	74677390	+	#01FC33	150157	141739	122466	83593	122466	272623	false
	HSA X	70593915	71221280	74728595	75340844	+	#01FC33	627365	612249	83593	58366	83593	710958	false
	HSA X	71279646	71420535	75360883	75517138	+	#01FC33	140889	156255	58366	100036	58366	199255	false
	HSA X	71520571	71577717	75603242	75669739	+	#01FC33	57146	66497	100036	827623	100036	157182	false

	HSA X	72405340	72462123	76689781	76804074	+	#01FC33	56783	114293	827623	60277	827623	884406	false
	HSA X	72522400	72692414	76854279	77152096	+	#01FC33	170014	297817	60277	362608	60277	230291	false
	HSA X	73055022	73468370	77415684	77723998	+	#01FC33	413348	308314	362608	28799	362608	775956	false
	HSA X	73497169	73885763	77781989	78051028	+	#01FC33	388594	269039	28799	115790	28799	417393	false
	HSA X	74001553	74064446	78100118	78150623	+	#01FC33	62893	50505	115790	13373	115790	178683	false
	HSA X	74077819	74174269	78266953	78365615	+	#01FC33	96450	98662	13373	383279	13373	109823	false
	HSA X	74557548	74981531	78940470	79286856	+	#01FC33	423983	346386	383279	21589	383279	807262	false
	HSA X	75003120	75113741	79357143	79441511	+	#01FC33	110621	84368	21589	463899	21589	132210	false
	HSA X	75577640	75723854	80002955	80141872	+	#01FC33	146214	138917	463899	129024	463899	610113	false
	HSA X	75852878	75952104	80187894	80346135	+	#01FC33	99226	158241	129024	189230	129024	228250	false
	HSA X	76141334	76319920	80650268	80749365	+	#01FC33	178586	99097	189230	54179	189230	367816	false
	HSA X	76374099	76442088	80754422	80814497	+	#01FC33	67989	60075	54179	203711	54179	122168	false
	HSA X	76645799	76837150	81111114	81298283	+	#01FC33	191351	187169	203711	1147445	203711	395062	false
	HSA X	77984595	78193480	82695994	82831658	+	#01FC33	208885	135664	1147445	498310	1147445	1356330	false
	HSA X	78691790	78962890	83415568	83576920	+	#01FC33	271100	161352	498310	407321	498310	769410	false
	HSA X	79370211	80125070	84035036	84564870	+	#01FC33	754859	529834	407321	164835	407321	1162180	false
	HSA X	80289905	80901241	84999805	85416228	+	#01FC33	611336	416423	164835	67394	164835	776171	false
	HSA X	80968635	81154806	85432355	85549412	+	#01FC33	186171	117057	67394	32478	67394	253565	false
	HSA X	81187284	81318384	85690094	85798181	+	#01FC33	131100	108087	32478	18345	32478	163578	false
	HSA X	81336729	82670191	85854959	86888340	+	#01FC33	1333462	1033381	18345	345456	18345	1351807	false
	HSA X	83015647	83167821	87301490	87435368	+	#01FC33	152174	133878	345456	27800	345456	497630	false
	HSA X	83195621	83400089	87486132	87670280	+	#01FC33	204468	184148	27800	163337	27800	232268	false
	HSA X	83563426	83663768	90131556	90189477	-	#01FC33	100342	57921	163337	494470	163337	263679	false
	HSA X	84158238	84261696	91425916	91496060	+	#01FC33	103458	70144	494470	22413	494470	597928	false
	HSA X	84284109	84363480	91548860	91617217	+	#01FC33	79371	68357	22413	13384	22413	101784	false
	HSA X	84376864	84603716	91708731	91844937	+	#01FC33	226852	136206	13384	182241	13384	240236	false
	HSA X	84785957	85427922	91997341	92422190	+	#01FC33	641965	424849	182241	80578	182241	824206	false
	HSA X	85508500	85642054	92536348	92630735	+	#01FC33	133554	94387	80578	474011	80578	214132	false
	HSA X	86116065	86410755	93410413	93599388	+	#01FC33	294690	188975	474011	80414	474011	768701	false
	HSA X	86491169	86854726	93613740	93927054	+	#01FC33	363557	313314	80414	65304	80414	443971	false
	HSA X	86920030	87395641	94050908	94377385	+	#01FC33	475611	326477	65304	4547	65304	540915	false
	HSA X	87400188	87540211	94429643	94589688	+	#01FC33	140023	160045	4547	220926	4547	144570	false

	HSA X	87761137	87883949	94768420	94857446	+	#01FC33	122812	89026	220926	3	220926	343738	false
	HSA X	87883952	87983199	94955277	95026072	+	#01FC33	99247	70795	3	238722	3	99250	false
	HSA X	88221921	88274514	95120629	95182062	+	#01FC33	52593	61433	238722	85914	238722	291315	false
	HSA X	88360428	88637501	95215229	95443465	+	#01FC33	277073	228236	85914	60344	85914	362987	false
	HSA X	88697845	88837392	95451961	95520288	+	#01FC33	139547	68327	60344	18029	60344	199891	false
	HSA X	88855421	91956904	95581522	97780359	+	#01FC33	3101483	2198837	18029	89548	18029	3119512	false
	HSA X	92046452	92326001	97826029	98008756	+	#01FC33	279549	182727	89548	61906	89548	369097	false
	HSA X	92387907	93033415	98032196	98506817	+	#01FC33	645508	474621	61906	83509	61906	707414	false
	HSA X	93116924	93738360	98508687	98906322	+	#01FC33	621436	397635	83509	77292	83509	704945	false
	HSA X	93815652	94236672	98968618	99196196	+	#01FC33	421020	227578	77292	1285	77292	498312	false
	HSA X	94237957	94492057	99248965	99455441	+	#01FC33	254100	206476	1285	71374	1285	255385	false
	HSA X	94563431	94767566	99495672	99632332	+	#01FC33	204135	136660	71374	81497	71374	275509	false
	HSA X	94849063	95544853	99663250	100127257	+	#01FC33	695790	464007	81497	11381	81497	777287	false
	HSA X	95556234	95663991	100127257	100219361	-	#01FC33	107757	92104	11381	7058	11381	119138	false
	HSA X	95671049	96035366	100241914	100516310	+	#01FC33	364317	274396	7058	64770	7058	371375	false
	HSA X	96100136	96604454	100521389	101032959	+	#01FC33	504318	511570	64770	52059	64770	569088	false
	HSA X	96656513	96968716	101035181	101293459	+	#01FC33	312203	258278	52059	18097	52059	364262	false
	HSA X	96986813	97160593	101343531	101497721	+	#01FC33	173780	154190	18097	-7819	18097	191877	false
	HSA X	97152774	97363177	101548240	101708180	+	#01FC33	210403	159940	-7819	128279	-7819	202584	false
	HSA X	97491456	97947326	101680478	102024505	+	#01FC33	455870	344027	128279	106794	128279	584149	false
	HSA X	98054120	98115725	102085739	102157653	+	#01FC33	61605	71914	106794	204536	106794	168399	false
	HSA X	98320261	98384261	102596767	102682056	-	#01FC33	64000	85289	204536	475828	204536	268536	false
	HSA X	98860089	99055751	103149665	103446518	+	#01FC33	195662	296853	475828	42308	475828	671490	false
	HSA X	99098059	99494538	103520764	103854586	+	#01FC33	396479	333822	42308	65815	42308	438787	false
	HSA X	99560353	99611800	103854259	103906120	+	#01FC33	51447	51861	65815	-26829	65815	117262	false
	HSA X	99584971	99655090	103917972	103989383	-	#01FC33	70119	71411	-26829	-70119	-26829	43290	false
	HSA X	99584971	102070630	104049479	106044838	+	#01FC33	2485659	1995359	-70119	-4471	-70119	2415540	false
	HSA X	102066159	102284843	106102600	106302674	+	#01FC33	218684	200074	-4471	-107155	-4471	214213	false
	HSA X	102177688	102286371	106244433	106350308	-	#01FC33	108683	105875	-107155	94482	-107155	1528	false
	HSA X	102380853	102458632	106476600	106538455	+	#01FC33	77779	61855	94482	28565	94482	172261	false
	HSA X	102487197	102952876	106604986	107019072	+	#01FC33	465679	414086	28565	156127	28565	494244	false
	HSA X	103109003	104832954	107061750	108486775	+	#01FC33	1723951	1425025	156127	50508	156127	1880078	false

	HSA X	104883462	105224501	108486778	108751471	+	#01FC33	341039	264693	50508	120235	50508	391547	false
	HSA X	105344736	105457978	108840383	108894686	+	#01FC33	113242	54303	120235	43965	120235	233477	false
	HSA X	105501943	105711884	108948710	109053317	+	#01FC33	209941	104607	43965	107722	43965	253906	false
	HSA X	105819606	105900451	109221364	109308771	+	#01FC33	80845	87407	107722	59475	107722	188567	false
	HSA X	105959926	107293084	109313826	110494205	+	#01FC33	1333158	1180379	59475	125689	59475	1392633	false
	HSA X	107418773	107506410	110551831	110639637	-	#01FC33	87637	87806	125689	0	125689	213326	false
	HSA X	107506410	112451854	110646173	114279885	+	#01FC33	4945444	3633712	0	5845	0	4945444	false
	HSA X	112457699	113035130	114332544	114914612	+	#01FC33	577431	582068	5845	100699	5845	583276	false
	HSA X	113135829	114060474	114950836	115727199	+	#01FC33	924645	776363	100699	40890	100699	1025344	false
	HSA X	114101364	114252898	115907531	116015605	+	#01FC33	151534	108074	40890	143944	40890	192424	false
	HSA X	114396842	114669231	116146558	116387180	+	#01FC33	272389	240622	143944	87722	143944	416333	false
	HSA X	114756953	115008755	116401055	116660216	+	#01FC33	251802	259161	87722	4685	87722	339524	false
	HSA X	115013440	115091116	116729609	116811795	+	#01FC33	77676	82186	4685	74718	4685	82361	false
	HSA X	115165834	118625426	116825197	119950104	+	#01FC33	3459592	3124907	74718	36899	74718	3534310	false
	HSA X	118662325	119555553	120199230	120876436	+	#01FC33	893228	677206	36899	-3700	36899	930127	false
	HSA X	119551853	123047260	120943263	124125746	+	#01FC33	3495407	3182483	-3700	8336	-3700	3491707	false
	HSA X	123055596	125230191	124197689	126292465	+	#01FC33	2174595	2094776	8336	50947	8336	2182931	false
	HSA X	125281138	125950480	126299720	126894449	+	#01FC33	669342	594729	50947	-2476	50947	720289	false
	HSA X	125948004	126523046	126916976	127334737	+	#01FC33	575042	417761	-2476	29650	-2476	572566	false
	HSA X	126552696	126668234	127393721	127471782	-	#01FC33	115538	78061	29650	94312	29650	145188	false
	HSA X	126762546	126856593	127564025	127624963	-	#01FC33	94047	60938	94312	1002	94312	188359	false
	HSA X	126857595	127334229	127629432	128041679	+	#01FC33	476634	412247	1002	173211	1002	477636	false
	HSA X	127507440	131475973	128249314	131677133	+	#01FC33	3968533	3427819	173211	189721	173211	4141744	false
	HSA X	131665694	134980447	131819141	135003063	+	#01FC33	3314753	3183922	189721	158045	189721	3504474	false
	HSA X	135138492	135224936	135319331	135386109	+	#01FC33	86444	66778	158045	9287	158045	244489	false
	HSA X	135234223	135294165	135026911	135093986	+	#01FC33	59942	67075	9287	52779	9287	69229	false
	HSA X	135346944	135448016	135321624	135445088	-	#01FC33	101072	123464	52779	70763	52779	153851	false
	HSA X	135518779	135571552	135130403	135216343	-	#01FC33	52773	85940	70763	-52773	70763	123536	false
	HSA X	135518779	135595997	135179165	135295100	+	#01FC33	77218	115935	-52773	-30249	-52773	24445	false
	HSA X	135565748	135653459	135135747	135195662	+	#01FC33	87711	59915	-30249	-22009	-30249	57462	false
	HSA X	135631450	135807962	135419610	135583159	+	#01FC33	176512	163549	-22009	-9456	-22009	154503	false
	HSA X	135798506	135865625	135590063	135687485	-	#01FC33	67119	97422	-9456	-64403	-9456	57663	false

	HSA X	135801222	135870195	135681772	135805908	+	#01FC33	68973	124136	-64403	8297	-64403	4570	false
	HSA X	135878492	138560594	135951016	138212298	+	#01FC33	2682102	2261282	8297	55450	8297	2690399	false
	HSA X	138616044	141285862	138245289	140879318	+	#01FC33	2669818	2634029	55450	53919	55450	2725268	false
	HSA X	141339781	141436962	140887782	140995024	+	#01FC33	97181	107242	53919	526	53919	151100	false
	HSA X	141437488	141788818	141035404	141472695	-	#01FC33	351330	437291	526	-85477	526	351856	false
	HSA X	141703341	142030727	141473986	141860993	+	#01FC33	327386	387007	-85477	1272	-85477	241909	false
	HSA X	142031999	142236544	141857815	142148667	-	#01FC33	204545	290852	1272	-24979	1272	205817	false
	HSA X	142211565	142709155	142162379	142697994	+	#01FC33	497590	535615	-24979	271	-24979	472611	false
	HSA X	142709426	142778416	142702876	142787261	-	#01FC33	68990	84385	271	-1159	271	69261	false
	HSA X	142777257	144283634	142795151	144423729	+	#01FC33	1506377	1628578	-1159	1507	-1159	1505218	false
	HSA X	144285141	144605444	144482948	144861727	+	#01FC33	320303	378779	1507	687	1507	321810	false
	HSA X	144606131	144719022	144862060	145002404	-	#01FC33	112891	140344	687	3657	687	113578	false
	HSA X	144722679	144986898	145002404	145241220	+	#01FC33	264219	238816	3657	107456	3657	267876	false
	HSA X	145094354	146635250	145274213	146764131	+	#01FC33	1540896	1489918	107456	0	107456	1648352	false
	HSA X	146635250	146818458	146766199	146931830	-	#01FC33	183208	165631	0	2701	0	183208	false
	HSA X	146821159	147071805	146948849	147152234	+	#01FC33	250646	203385	2701	42549	2701	253347	false
	HSA X	147114354	149183498	147289732	149531957	+	#01FC33	2069144	2242225	42549	782218	42549	2111693	false
	HSA X	149965716	150504965	151501615	152108586	+	#01FC33	539249	606971	782218	57884	782218	1321467	false
	HSA X	150562849	150862808	152145358	152519244	+	#01FC33	299959	373886	57884	54111	57884	357843	false
	HSA X	150916919	151090263	152519244	152692107	+	#01FC33	173344	172863	54111	43084	54111	227455	false
	HSA X	151133347	151258976	152807869	152998302	+	#01FC33	125629	190433	43084	6561	43084	168713	false
	HSA X	151265537	152024215	153280031	154219078	+	#01FC33	758678	939047	6561	-42170	6561	765239	false
	HSA X	151982045	152040221	154219582	154313258	+	#01FC33	58176	93676	-42170	28325	-42170	16006	false
	HSA X	152068546	152124598	154313284	154384525	-	#01FC33	56052	71241	28325	252	28325	84377	false
	HSA X	152124850	152271034	154397910	154553252	+	#01FC33	146184	155342	252	25980	252	146436	false
	HSA X	152297014	152730653	154674504	155134843	+	#01FC33	433639	460339	25980	2188	25980	459619	false
	HSA X	152732841	152845185	155213370	155336480	+	#01FC33	112344	123110	2188	30300	2188	114532	false
	HSA X	152875485	153264935	155463374	155944777	+	#01FC33	389450	481403	30300		30300	419750	false

Supplementary Table S2 (part 2). Syntenic blocks with a minimum cut-off length of 50 kilobases in the reference naked mole-rat genome.

scaffold	query	start	end	query_start	query_end	strand	color	target_len	query_len	dist_upstream	dist_downstream	dist_end_start	dist_end_end	embedded
HGL 1	CPOR 18	45687	1411972	41860665	43127206	-	#FA2D80	1366285	1266541		67863			

	CPOR 18	1479835	5781366	43131508	46911288	+	#FA2D80	4301531	3779780	67863	1247	67863	4369394	false
	CPOR 18	5782613	18627582	47084418	58693814	+	#FA2D80	12844969	11609396	1247	44825	1247	12846216	false
	CPOR 18	18672407	21821634	58773277	61756571	+	#FA2D80	3149227	2983294	44825	6437	44825	3194052	false
	CPOR 18	21828071	21996674	61758046	61891215	-	#FA2D80	168603	133169	6437	5976	6437	175040	false
	CPOR 18	22002650	27145174	61902893	66858664	+	#FA2D80	5142524	4955771	5976	94	5976	5148500	false
	CPOR 18	27145268	35295546	67138380	74300305	+	#FA2D80	8150278	7161925	94	70001	94	8150372	false
	CPOR 18	35365547	36277564	74334977	75237317	+	#FA2D80	912017	902340	70001	113069	70001	982018	false
	CPOR 18	36390633	36462984	78101371	78198288	-	#FA2D80	72351	96917	113069	2706	113069	185420	false
	CPOR 18	36465690	36549510	77898584	77971027	-	#FA2D80	83820	72443	2706	50705	2706	86526	false
	CPOR 18	36600215	36825960	77662511	77896964	-	#FA2D80	225745	234453	50705	71584	50705	276450	false
	CPOR 18	36897544	37152419	77356913	77660949	-	#FA2D80	254875	304036	71584	10976	71584	326459	false
	CPOR 18	37163395	37555386	76876194	77275011	-	#FA2D80	391991	398817	10976	183596	10976	402967	false
	CPOR 18	37738982	39512902	75276257	76831860	-	#FA2D80	1773920	1555603	183596	33434	183596	1957516	false
	CPOR 18	39546336	52846979	30166767	41857571	-	#FA2D80	13300643	11690804	33434	68900	33434	13334077	false
	CPOR 18	52915879	64948813	19095628	30216092	-	#FA2D80	12032934	11120464	68900	24723	68900	12101834	false
	CPOR 18	64973536	65109111	19138769	19232972	+	#FA2D80	135575	94203	24723	-51371	24723	160298	false
	CPOR 18	65057740	69455050	14956402	19091197	-	#FA2D80	4397310	4134795	-51371	56744	-51371	4345939	false
	CPOR 18	69511794	70926647	13646019	14956402	-	#FA2D80	1414853	1310383	56744	36907	56744	1471597	false
	CPOR 18	70963554	71086675	13522644	13638965	+	#FA2D80	123121	116321	36907	2633	36907	160028	false
	CPOR 18	71089308	72182652	12572794	13521307	-	#FA2D80	1093344	948513	2633	73456	2633	1095977	false
	CPOR 18	72256108	72449341	12323684	12486230	+	#FA2D80	193233	162546	73456	5175	73456	266689	false
	CPOR 18	72454516	72716777	12056280	12279825	-	#FA2D80	262261	223545	5175	480	5175	267436	false
	CPOR 18	72717257	72866398	11839765	11983002	-	#FA2D80	149141	143237	480	327023	480	149621	false
	CPOR 18	73193421	76080275	3678736	6341463	+	#FA2D80	2886854	2662727	327023	-45632	327023	3213877	false
	CPOR 18	76034643	78851406	6341869	9077174	+	#FA2D80	2816763	2735305	-45632	457	-45632	2771131	false
	CPOR 18	78851863	79071933	12083131	12277349	-	#FA2D80	220070	194218	457	28699	457	220527	false
	CPOR 18	79100632	79184584	11959581	12027730	-	#FA2D80	83952	68149	28699	129	28699	112651	false
	CPOR 18	79184713	79248708	11839765	11902458	-	#FA2D80	63995	62693	129	17297	129	64124	false
	CPOR 18	79266005	82140754	9282308	11949196	-	#FA2D80	2874749	2666888	17297	31731	17297	2892046	false
	CPOR 18	82172485	85391168	631672	3678736	+	#FA2D80	3218683	3047064	31731	5605	31731	3250414	false
	CPOR 18	85396773	85465688	12495862	12574430	-	#FA2D80	68915	78568	5605	39720	5605	74520	false
	CPOR 18	85505408	85929393	259140	631372	-	#FA2D80	423985	372232	39720		39720	463705	false

	CPOR 13	86000603	86351507	64482725	64813499	-	#11559D	350904	330774		59240			
	CPOR 13	86410747	87089998	63846662	64478220	-	#11559D	679251	631558	59240	56834	59240	738491	false
	CPOR 13	87146832	87825625	63309565	63846408	-	#11559D	678793	536843	56834	47716	56834	735627	false
	CPOR 13	87873341	87927678	63257067	63307823	+	#11559D	54337	50756	47716	12115	47716	102053	false
	CPOR 13	87939793	89028452	62455505	63256408	-	#11559D	1088659	800903	12115	12	12115	1100774	false
	CPOR 13	89028464	89256094	62257432	62455478	+	#11559D	227630	198046	12	7087	12	227642	false
	CPOR 13	89263181	92030450	59993719	62256325	-	#11559D	2767269	2262606	7087	108	7087	2774356	false
	CPOR 13	92030558	92394743	59604622	59896001	-	#11559D	364185	291379	108	400	108	364293	false
	CPOR 13	92395143	92644066	59348248	59517129	-	#11559D	248923	168881	400	35165	400	249323	false
	CPOR 13	92679231	94376748	57989645	59367724	-	#11559D	1697517	1378079	35165	-40790	35165	1732682	false
	CPOR 13	94335958	98469302	54546057	57979131	-	#11559D	4133344	3433074	-40790	25177	-40790	4092554	false
	CPOR 13	98494479	98585883	41840735	41950787	+	#11559D	91404	110052	25177	170926	25177	116581	false
	CPOR 13	98756809	105711160	48612283	54530568	-	#11559D	6954351	5918285	170926	-9509	170926	7125277	false
	CPOR 13	105701651	113211712	41861488	48601601	-	#11559D	7510061	6740113	-9509	777	-9509	7500552	false
	CPOR 13	113212489	113470073	41561525	41826617	+	#11559D	257584	265092	777	19923	777	258361	false
	CPOR 13	113489996	116268649	35769423	38257792	+	#11559D	2778653	2488369	19923	59517	19923	2798576	false
	CPOR 13	116328166	119888047	38297917	41548665	+	#11559D	3559881	3250748	59517	113259	59517	3619398	false
	CPOR 13	120001306	120118043	35499330	35585758	+	#11559D	116737	86428	113259	103106	113259	229996	false
	CPOR 13	120221149	120804518	35205376	35732049	-	#11559D	583369	526673	103106	407	103106	686475	false
	CPOR 13	120804925	125798074	30414436	35073481	-	#11559D	4993149	4659045	407	2289	407	4993556	false
	CPOR 13	125800363	131008503	25567453	30307573	-	#11559D	5208140	4740120	2289	-134229	2289	5210429	false
	CPOR 13	130874274	131008503	25411419	25538177	-	#11559D	134229	126758	-134229	-49837	-134229	0	true
	CPOR 13	130958666	132269036	20970553	22122694	-	#11559D	1310370	1152141	-49837	-1308178	-49837	1260533	false
	CPOR 13	130960858	132269036	18933012	20154393	-	#11559D	1308178	1221381	-1308178	251	-1308178	0	true
	CPOR 13	132269287	132338258	23242286	23306506	-	#11559D	68971	64220	251	431	251	69222	false
	CPOR 13	132338689	133250095	23307139	24027349	+	#11559D	911406	720210	431	-911406	431	911837	false
	CPOR 13	132338689	133260711	18086471	18864725	-	#11559D	922022	778254	-911406	-170668	-911406	10616	false
	CPOR 13	133090043	133250660	20156063	20279173	-	#11559D	160617	123110	-170668	34579	-170668	-10051	true
	CPOR 13	133285239	133337723	64918739	64969609	-	#11559D	52484	50870	34579		34579	87063	false
**HGL 10**	CPOR 10	25004	7066706	75653509	82407688	-	#CBE5A7	7041702	6754179		-67415			
	CPOR 10	6999291	17618141	65534165	75603645	-	#CBE5A7	10618850	10069480	-67415	-807743	-67415	10551435	false
	CPOR 10	16810398	28568033	54379017	65372588	-	#CBE5A7	11757635	10993571	-807743	136708	-807743	10949892	false

	CPOR 10	28704741	32163890	51255251	54394544	-	#CBE5A7	3459149	3139293	136708	14087	136708	3595857	false
	CPOR 10	32177977	32264596	51164454	51255251	+	#CBE5A7	86619	90797	14087	4369	14087	100706	false
	CPOR 10	32268965	36320948	47554219	51164439	-	#CBE5A7	4051983	3610220	4369	277	4369	4056352	false
	CPOR 10	36321225	37137613	46759701	47493901	-	#CBE5A7	816388	734200	277	89316	277	816665	false
	CPOR 10	37226929	44447715	40227608	46644371	-	#CBE5A7	7220786	6416763	89316	55697	89316	7310102	false
	CPOR 10	44503412	45631618	39153978	40195754	-	#CBE5A7	1128206	1041776	55697	18945	55697	1183903	false
	CPOR 10	45650563	50803483	34125967	38906119	-	#CBE5A7	5152920	4780152	18945	-122253	18945	5171865	false
	CPOR 10	50681230	54427879	30558245	33997020	-	#CBE5A7	3746649	3438775	-122253	97	-122253	3624396	false
	CPOR 10	54427976	55706819	29116565	30386413	-	#CBE5A7	1278843	1269848	97	-35458	97	1278940	false
	CPOR 10	55671361	59712138	25815626	29046445	-	#CBE5A7	4040777	3230819	-35458	156986	-35458	4005319	false
	CPOR 10	59869124	59943471	25725961	25814812	-	#CBE5A7	74347	88851	156986	-13196	156986	231333	false
	CPOR 10	59930275	61657742	23754469	25521881	-	#CBE5A7	1727467	1767412	-13196	-9017	-13196	1714271	false
	CPOR 10	61648725	62365882	22983574	23701695	-	#CBE5A7	717157	718121	-9017	149570	-9017	708140	false
	CPOR 10	62515452	62693976	20210544	20380849	-	#CBE5A7	178524	170305	149570	4405	149570	328094	false
	CPOR 10	62698381	63282491	19621476	20156170	-	#CBE5A7	584110	534694	4405	331047	4405	588515	false
	CPOR 10	63613538	63703123	20136820	20239725	+	#CBE5A7	89585	102905	331047	2779	331047	420632	false
	CPOR 10	63705902	65214477	21649128	22971534	-	#CBE5A7	1508575	1322406	2779	91582	2779	1511354	false
	CPOR 10	65306059	65443790	9967227	10075668	+	#CBE5A7	137731	108441	91582	191131	91582	229313	false
	CPOR 10	65634921	66925729	20356963	21514135	+	#CBE5A7	1290808	1157172	191131	96729	191131	1481939	false
	CPOR 10	67022458	68373641	18180421	19491953	-	#CBE5A7	1351183	1311532	96729	5950	96729	1447912	false
	CPOR 10	68379591	69138559	17244687	18011603	-	#CBE5A7	758968	766916	5950	877	5950	764918	false
	CPOR 10	69139436	70001299	16260901	17173804	-	#CBE5A7	861863	912903	877	8496	877	862740	false
	CPOR 10	70009795	70097658	16063387	16201943	-	#CBE5A7	87863	138556	8496		8496	96359	false
	CPOR 24	70905498	75339012	53281035	56608146	-	#AF0926	4433514	3327111	8475478	60247	8475478	12908992	false
	CPOR 24	75399259	77443781	51626259	53249618	-	#AF0926	2044522	1623359	60247	1380	60247	2104769	false
	CPOR 24	77445161	78944438	49413689	50541219	+	#AF0926	1499277	1127530	1380	3972	1380	1500657	false
	CPOR 24	78948410	79219022	50807829	51018740	+	#AF0926	270612	210911	3972	1313	3972	274584	false
	CPOR 24	79220335	79623006	51309121	51614637	+	#AF0926	402671	305516	1313	4431	1313	403984	false
	CPOR 24	79627437	79809832	48548675	48742436	+	#AF0926	182395	193761	4431	47185	4431	186826	false
	CPOR 24	79857017	79920106	48725902	48783117	-	#AF0926	63089	57215	47185	-17012	47185	110274	false
	CPOR 24	79903094	80085679	49322239	49391332	-	#AF0926	182585	69093	-17012	-133287	-17012	165573	false
	CPOR 24	79952392	80042589	49255792	49340564	+	#AF0926	90197	84772	-133287	393606	-133287	-43090	true

	CPOR 24	80436195	80799358	47595216	47896883	+	#AF0926	363163	301667	393606	91196	393606	756769	false
	CPOR 24	80890554	81020820	48555477	48668180	-	#AF0926	130266	112703	91196	54034	91196	221462	false
	CPOR 24	81074854	81284235	48307794	48530845	-	#AF0926	209381	223051	54034	-5384	54034	263415	false
	CPOR 24	81278851	81333572	48048373	48131414	-	#AF0926	54721	83041	-5384	-7149	-5384	49337	false
	CPOR 24	81326423	81400605	47929790	48059536	-	#AF0926	74182	129746	-7149	14971	-7149	67033	false
	CPOR 24	81415576	83124233	46559458	47894043	+	#AF0926	1708657	1334585	14971	474	14971	1723628	false
	CPOR 24	83124707	83284483	51192785	51306397	+	#AF0926	159776	113612	474		474	160250	false
**HGL 11**	CPOR 26	113062	4719585	9097786	13694933	+	#C9FE31	4606523	4597147		-14807			
	CPOR 26	4704778	4910951	13757158	13874456	+	#C9FE31	206173	117298	-14807	22742	-14807	191366	false
	CPOR 26	4933693	5064168	16473827	16625015	+	#C9FE31	130475	151188	22742	43306	22742	153217	false
	CPOR 26	5107474	5390489	16390578	16501722	+	#C9FE31	283015	111144	43306	-17259	43306	326321	false
	CPOR 26	5373230	5567410	16363321	16434616	-	#C9FE31	194180	71295	-17259	12967	-17259	176921	false
	CPOR 26	5580377	8004012	13895469	16276733	-	#C9FE31	2423635	2381264	12967	-37198	12967	2436602	false
	CPOR 26	7966814	8744479	16955471	17716308	+	#C9FE31	777665	760837	-37198	63152	-37198	740467	false
	CPOR 26	8807631	21734739	17720430	29863038	+	#C9FE31	12927108	12142608	63152	-747094	63152	12990260	false
	CPOR 26	20987645	21716841	29864228	30557291	-	#C9FE31	729196	693063	-747094	17898	-747094	-17898	true
	CPOR 26	21734739	21844332	30560084	30672694	-	#C9FE31	109593	112610	17898	-45766	17898	127491	false
	CPOR 26	21798566	21871691	30791675	30864706	+	#C9FE31	73125	73031	-45766	1659	-45766	27359	false
	CPOR 26	21873350	21999629	30870846	30945930	-	#C9FE31	126279	75084	1659	57742	1659	127938	false
	CPOR 26	22057371	23784367	30870846	32356430	+	#C9FE31	1726996	1485584	57742	-131559	57742	1784738	false
	CPOR 26	23652808	24531641	32358805	33007437	+	#C9FE31	878833	648632	-131559	57083	-131559	747274	false
	CPOR 26	24588724	28793095	33021965	36576827	+	#C9FE31	4204371	3554862	57083	-123614	57083	4261454	false
	CPOR 26	28669481	39430728	36576827	46235564	+	#C9FE31	10761247	9658737	-123614	2420	-123614	10637633	false
	CPOR 26	39433148	40009599	46237372	46766594	-	#C9FE31	576451	529222	2420	10196	2420	578871	false
	CPOR 26	40019795	41389969	46766594	47972662	+	#C9FE31	1370174	1206068	10196	50	10196	1380370	false
	CPOR 26	41390019	41492302	48000754	48081622	-	#C9FE31	102283	80868	50	1125	50	102333	false
	CPOR 26	41493427	42276589	48081663	48752957	+	#C9FE31	783162	671294	1125	59361	1125	784287	false
	CPOR 26	42335950	44717191	48806731	50944723	+	#C9FE31	2381241	2137992	59361	27929	59361	2440602	false
	CPOR 26	44745120	52031369	50920764	57427499	+	#C9FE31	7286249	6506735	27929	14995702	27929	7314178	false
	CPOR 31	51985295	52151031	35778650	35896363	+	#DB00F9	165736	117713		16728			
	CPOR 31	52167759	58076259	36073386	41261458	+	#DB00F9	5908500	5188072	16728	10763	16728	5925228	false
	CPOR 31	58087022	58176101	41261458	41346824	-	#DB00F9	89079	85366	10763	12257	10763	99842	false

	CPOR 31	58188358	61199428	41346827	43919796	+	#DB00F9	3011070	2572969	12257	65806	12257	3023327	false
	CPOR 31	61265234	61786815	44012785	44452771	+	#DB00F9	521581	439986	65806	61406	65806	587387	false
	CPOR 31	61848221	64528523	44720937	47078846	+	#DB00F9	2680302	2357909	61406	28422	61406	2741708	false
	CPOR 31	64556945	65038287	47139615	47570975	+	#DB00F9	481342	431360	28422	26509	28422	509764	false
	CPOR 31	65064796	65289233	47623370	47813161	+	#DB00F9	224437	189791	26509		26509	250946	false
	CPOR 27	65401558	65610705	14709620	14898827	+	#0800AC	209147	189207		83206			
	CPOR 27	65693911	65869210	14898827	15007259	+	#0800AC	175299	108432	83206		83206	258505	false
	CPOR 2	66124660	66240131	32178646	32296526	+	#7FFF00	115471	117880		-74293			
	CPOR 2	66165838	66255615	30450693	30545092	-	#7FFF00	89777	94399	-74293	58734	-74293	15484	false
	CPOR 2	66314349	66365023	30098300	30193912	-	#7FFF00	50674	95612	58734	86863	58734	109408	false
	CPOR 2	66451886	66567430	9467199	9526010	+	#7FFF00	115544	58811	86863	459641	86863	202407	false
	CPOR 26	67027071	67081004	85341	142436	-	#C9FE31	53933	57095	14995702	2858979	14995702	15049635	false
	CPOR 2	67027071	67230510	31001484	31118279	-	#7FFF00	203439	116795	459641	-127706	459641	663080	false
	CPOR 2	67102804	67301024	32124296	32210115	-	#7FFF00	198220	85819	-127706	37601	-127706	70514	false
	CPOR 2	67338625	67418193	29457541	29516434	-	#7FFF00	79568	58893	37601	-31292	37601	117169	false
	CPOR 2	67386901	67446720	29581450	29662335	+	#7FFF00	59819	80885	-31292	26072	-31292	28527	false
	CPOR 2	67472792	67531752	9783477	9843553	+	#7FFF00	58960	60076	26072	110736	26072	85032	false
	CPOR 2	67642488	67701816	32080558	32133086	-	#7FFF00	59328	52528	110736	199693	110736	170064	false
	CPOR 2	67901509	67969578	31678971	31758091	+	#7FFF00	68069	79120	199693	168248	199693	267762	false
	CPOR 2	68137826	68242425	9459049	9520734	-	#7FFF00	104599	61685	168248	-78318	168248	272847	false
	CPOR 2	68164107	68244142	9408471	9460351	-	#7FFF00	80035	51880	-78318	176306	-78318	1717	false
	CPOR 2	68420448	68503737	29257168	29314448	+	#7FFF00	83289	57280	176306	82958	176306	259595	false
	CPOR 2	68586695	68705841	32043972	32199915	+	#7FFF00	119146	155943	82958	109458	82958	202104	false
	CPOR 2	68815299	68899975	9455391	9534495	-	#7FFF00	84676	79104	109458	46477	109458	194134	false
	CPOR 2	68946452	69000273	32124149	32186723	-	#7FFF00	53821	62574	46477	34514	46477	100298	false
	CPOR 2	69034787	69111131	30415171	30484384	+	#7FFF00	76344	69213	34514	44876	34514	110858	false
	CPOR 2	69156007	69208880	30097642	30194876	+	#7FFF00	52873	97234	44876	511028	44876	97749	false
	CPOR 19	69466095	69560041	21790363	21843561	+	#7AB506	93946	53198		815249			
	CPOR 2	69719908	69925063	32124937	32210115	+	#7FFF00	205155	85178	511028	-78452	511028	716183	false
	CPOR 2	69846611	70071339	31024077	31190922	+	#7FFF00	224728	166845	-78452	-131356	-78452	146276	false
	CPOR 26	69939983	70006993	85341	145914	+	#C9FE31	67010	60573	2858979		2858979	2925989	false
	CPOR 2	69939983	70087489	30103705	30250387	-	#7FFF00	147506	146682	-131356	-64076	-131356	16150	false

	CPOR 2	70023413	70099819	29775260	29839478	+	#7FFF00	76406	64218	-64076	-76406	-64076	12330	false
	CPOR 2	70023413	70100634	9773905	9877042	+	#7FFF00	77221	103137	-76406	-56495	-76406	815	false
	CPOR 2	70044139	70100634	29690343	29757466	-	#7FFF00	56495	67123	-56495	110902	-56495	0	true
	CPOR 2	70211536	70269477	29579117	29637035	+	#7FFF00	57941	57918	110902	22696424	110902	168843	false
	CPOR 19	70375290	71112500	21128000	21787837	-	#7AB506	737210	659837	815249	5919	815249	1552459	false
	CPOR 19	71118419	80253982	12955766	21070945	-	#7AB506	9135563	8115179	5919	101327	5919	9141482	false
	CPOR 19	80355309	80448098	12764035	12840994	+	#7AB506	92789	76959	101327	-92789	101327	194116	false
	CPOR 19	80355309	90456343	3104938	12731446	-	#7AB506	10101034	9626508	-92789	68309	-92789	10008245	false
	CPOR 19	90524652	92965901	677878	3004221	-	#7AB506	2441249	2326343	68309	31636210	68309	2509558	false
	CPOR 2	92965901	95244533	7038148	9141189	-	#7FFF00	2278632	2103041	22696424	4726	22696424	24975056	false
	CPOR 2	95249259	96936093	5446699	6984295	-	#7FFF00	1686834	1537596	4726	1264893	4726	1691560	false
	CPOR 22	96841577	97941331	25392186	26335048	+	#007F00	1099754	942862		55			
	CPOR 22	97941386	98202672	25153681	25391765	+	#007F00	261286	238084	55		55	261341	false
	CPOR 2	98200986	103692749	382774	5446250	-	#7FFF00	5491763	5063476	1264893	-181452	1264893	6756656	false
	CPOR 2	103511297	103723969	157227	382040	-	#7FFF00	212672	224813	-181452	33928	-181452	31220	false
	CPOR 2	103757897	104535409	10269182	11070674	+	#7FFF00	777512	801492	33928	41867	33928	811440	false
	CPOR 2	104577276	106173753	11123142	12507568	+	#7FFF00	1596477	1384426	41867	54570	41867	1638344	false
	CPOR 2	106228323	122808356	12558687	28486386	+	#7FFF00	16580033	15927699	54570	156658	54570	16634603	false
	CPOR 2	122965014	123059220	9597632	9708042	-	#7FFF00	94206	110410	156658	100591	156658	250864	false
	CPOR 2	123159811	123372926	28789574	29011108	-	#7FFF00	213115	221534	100591	-77405	100591	313706	false
	CPOR 2	123295521	123406195	28652341	28741314	-	#7FFF00	110674	88973	-77405	7479	-77405	33269	false
	CPOR 2	123413674	123507476	31119195	31193959	+	#7FFF00	93802	74764	7479	28227	7479	101281	false
	CPOR 2	123535703	123588671	29225887	29276369	-	#7FFF00	52968	50482	28227	-30652	28227	81195	false
	CPOR 2	123558019	123613277	29139716	29203892	-	#7FFF00	55258	64176	-30652	76787	-30652	24606	false
	CPOR 2	123690064	123745005	31681827	31758091	+	#7FFF00	54941	76264	76787	224717	76787	131728	false
	CPOR 2	123969722	124030020	29597477	29693780	+	#7FFF00	60298	96303	224717	140312	224717	285015	false
	CPOR 2	124170332	124220774	32234971	32296422	+	#7FFF00	50442	61451	140312	25138	140312	190754	false
	CPOR 2	124245912	124382704	9397539	9492948	+	#7FFF00	136792	95409	25138	118647	25138	161930	false
	CPOR 2	124501351	124601580	9342040	9424374	-	#7FFF00	100229	82334	118647		118647	218876	false
**HGL 12**	CPOR 16	31887	902098	14837922	15717188	-	#81A8F8	870211	879266		-4474			
	CPOR 16	897624	1715254	0	819589	+	#81A8F8	817630	819589	-4474	52	-4474	813156	false
	CPOR 16	1715306	5722327	1084066	4860454	+	#81A8F8	4007021	3776388	52	-140532	52	4007073	false

	CPOR 16	5581795	5741966	5016009	5125935	+	#81A8F8	160171	109926	-140532	-96042	-140532	19639	false
	CPOR 16	5645924	5741966	5142149	5222354	+	#81A8F8	96042	80205	-96042	-33248	-96042	0	true
	CPOR 16	5708718	7418128	5249661	6974389	+	#81A8F8	1709410	1724728	-33248	54695	-33248	1676162	false
	CPOR 16	7472823	7558375	6974389	7055684	-	#81A8F8	85552	81295	54695	31046	54695	140247	false
	CPOR 16	7589421	10470074	7067849	10019263	+	#81A8F8	2880653	2951414	31046	8938	31046	2911699	false
	CPOR 16	10479012	10619847	10148906	10270572	+	#81A8F8	140835	121666	8938	-99923	8938	149773	false
	CPOR 16	10519924	15272556	10318686	14836922	+	#81A8F8	4752632	4518236	-99923		-99923	4652709	false
	CPOR 30	15278582	16749708	100	1450001	+	#FF7F00	1471126	1449901		97412			
	CPOR 30	16847120	26595937	1492560	10608802	+	#FF7F00	9748817	9116242	97412	50343	97412	9846229	false
	CPOR 30	26646280	28850078	10611173	12605727	+	#FF7F00	2203798	1994554	50343	60704	50343	2254141	false
	CPOR 30	28910782	29050367	12640923	12778920	+	#FF7F00	139585	137997	60704	7910	60704	200289	false
	CPOR 30	29058277	29298537	12779340	12970834	-	#FF7F00	240260	191494	7910	4513	7910	248170	false
	CPOR 30	29303050	34557779	12971453	17816804	+	#FF7F00	5254729	4845351	4513	56636	4513	5259242	false
	CPOR 30	34614415	49354179	17816804	30981486	+	#FF7F00	14739764	13164682	56636	75447	56636	14796400	false
	CPOR 30	49429626	55369163	30981519	36151272	+	#FF7F00	5939537	5169753	75447	8	75447	6014984	false
	CPOR 30	55369171	56752876	36227908	37449653	+	#FF7F00	1383705	1221745	8	70090	8	1383713	false
	CPOR 30	56822966	57480958	37493429	37998602	+	#FF7F00	657992	505173	70090	-5112	70090	728082	false
	CPOR 30	57475846	57577886	38001933	38100758	-	#FF7F00	102040	98825	-5112	2653	-5112	96928	false
	CPOR 30	57580539	60088063	38100805	40088749	+	#FF7F00	2507524	1987944	2653	65776	2653	2510177	false
	CPOR 30	60153839	60228138	40101590	40188659	+	#FF7F00	74299	87069	65776	335397	65776	140075	false
	CPOR 30	60563535	60624440	40813389	40887081	+	#FF7F00	60905	73692	335397	1062720	335397	396302	false
	CPOR 30	61687160	61773704	41385113	41498266	+	#FF7F00	86544	113153	1062720	122711	1062720	1149264	false
	CPOR 30	61896415	63877833	41525394	43041290	+	#FF7F00	1981418	1515896	122711	55065	122711	2104129	false
	CPOR 30	63932898	65370559	43051651	44283631	+	#FF7F00	1437661	1231980	55065	209088	55065	1492726	false
	CPOR 30	65579647	65856189	44361264	44644930	+	#FF7F00	276542	283666	209088	117063	209088	485630	false
	CPOR 30	65973252	66302879	44714195	45014456	+	#FF7F00	329627	300261	117063		117063	446690	false
	CPOR 9	66457375	66509189	62248162	62321552	-	#BF726D	51814	73390		126249			
	CPOR 9	66635438	66812314	61993741	62153494	-	#BF726D	176876	159753	126249	23142	126249	303125	false
	CPOR 9	66835456	68928283	59933931	61923719	-	#BF726D	2092827	1989788	23142	10255	23142	2115969	false
	CPOR 9	68938538	75516904	53040456	59761724	-	#BF726D	6578366	6721268	10255	100214	10255	6588621	false
	CPOR 9	75617118	86345360	42789355	53040333	-	#BF726D	10728242	10250978	100214	40815	100214	10828456	false
	CPOR 9	86386175	86562016	63036879	63188628	-	#BF726D	175841	151749	40815	-48452	40815	216656	false

	CPOR 9	86513564	90068718	63286465	66943108	+	#BF726D	3555154	3656643	-48452		-48452	3506702	false
	CPOR 10	90058565	91866560	44221	1804106	+	#CBE5A7	1807995	1759885		6554			
	CPOR 10	91873114	91924950	1900222	1959124	+	#CBE5A7	51836	58902	6554	0	6554	58390	false
	CPOR 10	91924950	97751209	2059011	7431761	+	#CBE5A7	5826259	5372750	0	23015	0	5826259	false
	CPOR 10	97774224	100074019	7510332	9838731	+	#CBE5A7	2299795	2328399	23015		23015	2322810	false
**HGL 13**	CPOR 28	8512	303495	150643	469540	+	#60765F	294983	318897		2			
	CPOR 28	303497	572706	737909	952112	-	#60765F	269209	214203	2	1708	2	269211	false
	CPOR 28	574414	731725	1167209	1319331	+	#60765F	157311	152122	1708	62636	1708	159019	false
	CPOR 28	794361	1385290	1514757	2120833	+	#60765F	590929	606076	62636	4	62636	653565	false
	CPOR 28	1385294	1467921	3646223	3696691	+	#60765F	82627	50468	4	-43861	4	82631	false
	CPOR 28	1424060	1616745	3694766	3974987	+	#60765F	192685	280221	-43861	-90110	-43861	148824	false
	CPOR 28	1526635	1818267	3967025	4342741	+	#60765F	291632	375716	-90110	159	-90110	201522	false
	CPOR 28	1818426	1973713	4555220	4712154	+	#60765F	155287	156934	159	60973	159	155446	false
	CPOR 28	2034686	4281936	4761262	6962492	+	#60765F	2247250	2201230	60973	-11004	60973	2308223	false
	CPOR 28	4270932	6464019	6952313	8930506	-	#60765F	2193087	1978193	-11004	-26681	-11004	2182083	false
	CPOR 28	6437338	12833059	8920370	15216443	+	#60765F	6395721	6296073	-26681	23661	-26681	6369040	false
	CPOR 28	12856720	22119098	15298549	24328028	+	#60765F	9262378	9029479	23661	122194	23661	9286039	false
	CPOR 28	22241292	23174742	24329299	25183348	+	#60765F	933450	854049	122194	58591	122194	1055644	false
	CPOR 28	23233333	23469423	25201854	25405367	+	#60765F	236090	203513	58591	21811	58591	294681	false
	CPOR 28	23491234	34711934	25463881	35393204	+	#60765F	11220700	9929323	21811	98106	21811	11242511	false
	CPOR 28	34810040	40940289	35393204	40451119	+	#60765F	6130249	5057915	98106	-34371	98106	6228355	false
	CPOR 28	40905918	43603576	40564986	42811108	+	#60765F	2697658	2246122	-34371	171849	-34371	2663287	false
	CPOR 28	43775425	43927188	42880245	42957600	+	#60765F	151763	77355	171849	77423	171849	323612	false
	CPOR 28	44004611	44250191	43004023	43225802	-	#60765F	245580	221779	77423	-13330	77423	323003	false
	CPOR 28	44236861	45888570	43228697	44676762	+	#60765F	1651709	1448065	-13330	1843	-13330	1638379	false
	CPOR 28	45890413	48833388	45808878	48398397	-	#60765F	2942975	2589519	1843		1843	2944818	false
	CPOR 2	48998029	49049628	34235967	34293120	+	#7FFF00	51599	57153		303460			
	CPOR 2	49353088	49446722	34422231	34517608	+	#7FFF00	93634	95377	303460	72580	303460	397094	false
	CPOR 2	49519302	50083058	34571610	35091880	+	#7FFF00	563756	520270	72580	71033	72580	636336	false
	CPOR 2	50154091	51538333	35104042	36373193	+	#7FFF00	1384242	1269151	71033	23443	71033	1455275	false
	CPOR 2	51561776	51747260	50487004	50700333	-	#7FFF00	185484	213329	23443	259	23443	208927	false
	CPOR 2	51747519	53756929	36373196	38228804	+	#7FFF00	2009410	1855608	259	53825	259	2009669	false

	CPOR 2	53810754	53881539	38235442	38323199	+	#7FFF00	70785	87757	53825	1662	53825	124610	false
	CPOR 2	53883201	54381365	38385836	38799010	+	#7FFF00	498164	413174	1662	81170	1662	499826	false
	CPOR 2	54462535	55053259	38859815	39423303	+	#7FFF00	590724	563488	81170	3	81170	671894	false
	CPOR 2	55053262	55309978	39477303	39644714	+	#7FFF00	256716	167411	3	393314	3	256719	false
	CPOR 2	55703292	56062696	39780470	39969925	+	#7FFF00	359404	189455	393314	-160598	393314	752718	false
	CPOR 2	55902098	59496814	39922645	43393725	+	#7FFF00	3594716	3471080	-160598	50150	-160598	3434118	false
	CPOR 2	59546964	66986476	43395184	50475008	+	#7FFF00	7439512	7079824	50150	405	50150	7489662	false
	CPOR 2	66986881	68850524	50641642	52506423	+	#7FFF00	1863643	1864781	405	67581	405	1864048	false
	CPOR 2	68918105	69336112	52510715	53007238	+	#7FFF00	418007	496523	67581	58916	67581	485588	false
	CPOR 2	69395028	69678433	53007691	53324703	+	#7FFF00	283405	317012	58916	2638	58916	342321	false
	CPOR 2	69681071	72910777	53397675	56637455	+	#7FFF00	3229706	3239780	2638	-27370	2638	3232344	false
	CPOR 2	72883407	87311750	56648915	71353990	+	#7FFF00	14428343	14705075	-27370		-27370	14400973	false
**HGL 14**	CPOR 8	65886	158466	97892266	97997679	-	#1036F2	92580	105413		-92580			
	CPOR 8	65886	180583	101809213	101918715	-	#1036F2	114697	109502	-92580		-92580	22117	false
	CPOR 1	202252	305836	47553527	47648088	+	#A6F1FC	103584	94561		14557			
	CPOR 1	320393	10894764	47734995	56969368	+	#A6F1FC	10574371	9234373	14557	-122075	14557	10588928	false
	CPOR 1	10772689	10864058	57246959	57352830	+	#A6F1FC	91369	105871	-122075	64308	-122075	-30706	true
	CPOR 1	10928366	11016747	57245706	57351260	-	#A6F1FC	88381	105554	64308	-86837	64308	152689	false
	CPOR 1	10929910	11028793	56859563	56948218	-	#A6F1FC	98883	88655	-86837	-85068	-86837	12046	false
	CPOR 1	10943725	11029640	57030690	57089610	-	#A6F1FC	85915	58920	-85068	0	-85068	847	false
	CPOR 1	11029640	11169199	56998342	57129236	+	#A6F1FC	139559	130894	0	-131591	0	139559	false
	CPOR 1	11037608	11214278	56826474	56949554	+	#A6F1FC	176670	123080	-131591	11483	-131591	45079	false
	CPOR 1	11225761	11344503	56836482	56998141	-	#A6F1FC	118742	161659	11483	-74995	11483	130225	false
	CPOR 1	11269508	11362958	56999680	57096606	-	#A6F1FC	93450	96926	-74995	20711	-74995	18455	false
	CPOR 1	11383669	11989337	57292279	57848320	+	#A6F1FC	605668	556041	20711	85419	20711	626379	false
	CPOR 1	12074756	17158405	57852136	62503484	+	#A6F1FC	5083649	4651348	85419	21472	85419	5169068	false
	CPOR 1	17179877	22006840	62580028	67227847	+	#A6F1FC	4826963	4647819	21472	6067	21472	4848435	false
	CPOR 1	22012907	36471743	67323857	81178067	+	#A6F1FC	14458836	13854210	6067	-19692	6067	14464903	false
	CPOR 1	36452051	36506185	81685242	81740476	+	#A6F1FC	54134	55234	-19692	289786	-19692	34442	false
	CPOR 1	36795971	39292936	81848083	84159884	+	#A6F1FC	2496965	2311801	289786	52444	289786	2786751	false
	CPOR 1	39345380	42874297	84219010	87150078	+	#A6F1FC	3528917	2931068	52444	-23523	52444	3581361	false
	CPOR 1	42850774	46959090	87162907	90871817	+	#A6F1FC	4108316	3708910	-23523	60419	-23523	4084793	false

	CPOR 1	47019509	48400794	90875388	91980048	+	#A6F1FC	1381285	1104660	60419	-60491	60419	1441704	false
	CPOR 1	48340303	51978268	177189741	180593228	-	#A6F1FC	3637965	3403487	-60491	52203	-60491	3577474	false
	CPOR 1	52030471	52211074	176964589	177133438	-	#A6F1FC	180603	168849	52203	287699	52203	232806	false
	CPOR 1	52498773	53586015	175882823	176956741	-	#A6F1FC	1087242	1073918	287699	909	287699	1374941	false
	CPOR 1	53586924	58501260	171128730	175763563	-	#A6F1FC	4914336	4634833	909	86608	909	4915245	false
	CPOR 1	58587868	69862525	160883211	171138774	-	#A6F1FC	11274657	10255563	86608	22	86608	11361265	false
	CPOR 1	69862547	69950915	160417524	160521301	-	#A6F1FC	88368	103777	22	-88368	22	88390	false
	CPOR 1	69862547	69963770	160328656	160442731	+	#A6F1FC	101223	114075	-88368	-31755	-88368	12855	false
	CPOR 1	69932015	71448777	158779446	160304157	-	#A6F1FC	1516762	1524711	-31755	54117	-31755	1485007	false
	CPOR 1	71502894	78088954	152257938	158774186	-	#A6F1FC	6586060	6516248	54117	37184	54117	6640177	false
	CPOR 1	78126138	79079824	181052434	182067071	+	#A6F1FC	953686	1014637	37184	30276	37184	990870	false
	CPOR 1	79110100	84475555	182142721	187222289	+	#A6F1FC	5365455	5079568	30276	37957	30276	5395731	false
	CPOR 1	84513512	84599245	187584442	187700028	+	#A6F1FC	85733	115586	37957	-38685	37957	123690	false
	CPOR 1	84560560	84725702	187711976	187894079	+	#A6F1FC	165142	182103	-38685	197947	-38685	126457	false
	CPOR 1	84923649	84988345	189167270	189242701	+	#A6F1FC	64696	75431	197947	219768	197947	262643	false
	CPOR 1	85208113	85269953	189571916	189632412	+	#A6F1FC	61840	60496	219768	-61840	219768	281608	false
	CPOR 1	85208113	91881877	189670440	196230313	+	#A6F1FC	6673764	6559873	-61840		-61840	6611924	false
**HGL 15**	CPOR 24	5423	792238	38611199	39231720	+	#AF0926	786815	620521		-786815			
	CPOR 24	5423	846984	41256285	41962640	+	#AF0926	841561	706355	-786815		-786815	54746	false
	CPOR 19	895150	1303932	22666971	23103099	+	#7AB506	408782	436128		131738			
	CPOR 19	1435670	14018073	23109926	33897029	+	#7AB506	12582403	10787103	131738	51195	131738	12714141	false
	CPOR 19	14069268	20760641	33926185	39543343	+	#7AB506	6691373	5617158	51195	38	51195	6742568	false
	CPOR 19	20760679	21611070	39605316	40386575	+	#7AB506	850391	781259	38	62056	38	850429	false
	CPOR 19	21673126	24681829	40386622	42872963	+	#7AB506	3008703	2486341	62056	19894	62056	3070759	false
	CPOR 19	24701723	27601516	58692143	61080017	-	#7AB506	2899793	2387874	19894	5749	19894	2919687	false
	CPOR 19	27607265	27790240	58569751	58692137	+	#7AB506	182975	122386	5749	9277	5749	188724	false
	CPOR 19	27799517	39093691	48422344	58565098	-	#7AB506	11294174	10142754	9277	51373	9277	11303451	false
	CPOR 19	39145064	44700933	43194217	48395051	-	#7AB506	5555869	5200834	51373	0	51373	5607242	false
	CPOR 19	44700933	44844868	42954468	43097018	-	#7AB506	143935	142550	0		0	143935	false
	CPOR 3	44827341	44892661	3251537	3361699	+	#D9C001	65320	110162		13065			
	CPOR 3	44905726	46321914	3454194	4658243	+	#D9C001	1416188	1204049	13065	122784	13065	1429253	false
	CPOR 3	46444698	77939228	4707100	33412199	+	#D9C001	31494530	28705099	122784	6772	122784	31617314	false

	CPOR 3	77946000	80164261	33534354	35675591	+	#D9C001	2218261	2141237	6772	96236	6772	2225033	false
	CPOR 3	80260497	82792342	35709314	38086393	+	#D9C001	2531845	2377079	96236	0	96236	2628081	false
	CPOR 3	82792342	82852107	38086691	38164515	-	#D9C001	59765	77824	0	0	0	59765	false
	CPOR 3	82852107	93261865	38165197	48015453	+	#D9C001	10409758	9850256	0		0	10409758	false
**HGL 16**	CPOR 8	44814	17957406	43733	16979230	+	#1036F2	17912592	16935497		0			
	CPOR 8	17957406	18017606	16979671	17037831	-	#1036F2	60200	58160	0	2	0	60200	false
	CPOR 8	18017608	34034919	17039328	31700914	+	#1036F2	16017311	14661586	2	50332	2	16017313	false
	CPOR 8	34085251	36584147	31734735	33964217	+	#1036F2	2498896	2229482	50332	-19133	50332	2549228	false
	CPOR 8	36565014	37438808	34183588	35032695	+	#1036F2	873794	849107	-19133	42161	-19133	854661	false
	CPOR 8	37480969	41972264	35261701	39485159	+	#1036F2	4491295	4223458	42161	29812	42161	4533456	false
	CPOR 8	42002076	48638138	39609624	46078143	+	#1036F2	6636062	6468519	29812	-73859	29812	6665874	false
	CPOR 8	48564279	48655714	46268694	46355369	+	#1036F2	91435	86675	-73859	68120	-73859	17576	false
	CPOR 8	48723834	49900141	46355446	47505727	+	#1036F2	1176307	1150281	68120	29512	68120	1244427	false
	CPOR 8	49929653	50058836	47630232	47747742	+	#1036F2	129183	117510	29512	90846	29512	158695	false
	CPOR 8	50149682	50286608	47886613	47985180	+	#1036F2	136926	98567	90846	157026	90846	227772	false
	CPOR 8	50443634	50662416	48317992	48486256	+	#1036F2	218782	168264	157026	-99204	157026	375808	false
	CPOR 8	50563212	50902590	48612985	48869668	+	#1036F2	339378	256683	-99204	-244459	-99204	240174	false
	CPOR 8	50658131	50902590	49175309	49284223	+	#1036F2	244459	108914	-244459	-195749	-244459	0	true
	CPOR 8	50706841	50902590	49018698	49204888	+	#1036F2	195749	186190	-195749	64	-195749	0	true
	CPOR 8	50902654	51113055	49436018	49590771	+	#1036F2	210401	154753	64	-48890	64	210465	false
	CPOR 8	51064165	51150645	49607422	49663756	+	#1036F2	86480	56334	-48890	5426	-48890	37590	false
	CPOR 8	51156071	51337056	49725777	49917370	+	#1036F2	180985	191593	5426	18936	5426	186411	false
	CPOR 8	51355992	51467575	49951904	50013979	-	#1036F2	111583	62075	18936	33774	18936	130519	false
	CPOR 8	51501349	52816411	50011917	51373717	+	#1036F2	1315062	1361800	33774	76159	33774	1348836	false
	CPOR 8	52892570	53651349	51373717	52169374	+	#1036F2	758779	795657	76159	-45083	76159	834938	false
	CPOR 8	53606266	53766260	52181356	52341800	+	#1036F2	159994	160444	-45083	1281571	-45083	114911	false
	CPOR 8	55047831	57473317	70362424	72370111	-	#1036F2	2425486	2007687	1281571	65503	1281571	3707057	false
	CPOR 8	57538820	58501970	69385267	70341630	-	#1036F2	963150	956363	65503	55982	65503	1028653	false
	CPOR 8	58557952	59380415	68637420	69311051	-	#1036F2	822463	673631	55982	1610	55982	878445	false
	CPOR 8	59382025	59441539	68500999	68559855	-	#1036F2	59514	58856	1610	19590	1610	61124	false
	CPOR 8	59461129	59640054	68323385	68440353	-	#1036F2	178925	116968	19590	6049	19590	198515	false
	CPOR 8	59646103	61507228	66334525	68118020	-	#1036F2	1861125	1783495	6049	800	6049	1867174	false

	CPOR 8	61508028	63802716	64053366	66282575	-	#1036F2	2294688	2229209	800	22753	800	2295488	false
	CPOR 8	63825469	65873168	62202562	63980558	-	#1036F2	2047699	1777996	22753	-18548	22753	2070452	false
	CPOR 8	65854620	65952718	62068254	62145244	-	#1036F2	98098	76990	-18548	-2466	-18548	79550	false
	CPOR 8	65950252	72360810	56433529	62199118	-	#1036F2	6410558	5765589	-2466	15583	-2466	6408092	false
	CPOR 8	72376393	72489175	56278496	56377434	-	#1036F2	112782	98938	15583	59244	15583	128365	false
	CPOR 8	72548419	72679027	56061310	56228235	-	#1036F2	130608	166925	59244	42170	59244	189852	false
	CPOR 8	72721197	75157396	53490786	55936508	-	#1036F2	2436199	2445722	42170	-79099	42170	2478369	false
	CPOR 8	75078297	76344281	52342772	53489574	-	#1036F2	1265984	1146802	-79099		-79099	1186885	false
**HGL 17**	CPOR 12	86334	11497807	77559382	88218628	-	#9B0496	11411473	10659246		2477			
	CPOR 12	11500284	11583481	77479043	77559345	+	#9B0496	83197	80302	2477	0	2477	85674	false
	CPOR 12	11583481	18701491	70824211	77478949	-	#9B0496	7118010	6654738	0	1248	0	7118010	false
	CPOR 12	18702739	23970570	65902979	70750575	-	#9B0496	5267831	4847596	1248	403	1248	5269079	false
	CPOR 12	23970973	30576371	59662044	65538031	-	#9B0496	6605398	5875987	403	2105	403	6605801	false
	CPOR 12	30578476	32107750	57786437	59180041	-	#9B0496	1529274	1393604	2105	6158	2105	1531379	false
	CPOR 12	32113908	32812456	57172129	57786437	+	#9B0496	698548	614308	6158	3679	6158	704706	false
	CPOR 12	32816135	53624308	38252720	57171474	-	#9B0496	20808173	18918754	3679		3679	20811852	false
	CPOR 10	54092104	57315986	12558757	15642792	-	#CBE5A7	3223882	3084035		53764			
	CPOR 10	57369750	57876406	12172258	12542716	-	#CBE5A7	506656	370458	53764	224	53764	560420	false
	CPOR 10	57876630	59599237	10540444	12013215	-	#CBE5A7	1722607	1472771	224	66311	224	1722831	false
	CPOR 10	59665548	60190451	10076120	10540075	-	#CBE5A7	524903	463955	66311		66311	591214	false
**HGL 18**	CPOR 4	33805	1300820	94080354	95350692	-	#26B63B	1267015	1270338		21822			
	CPOR 4	1322642	2313477	92853621	93857558	-	#26B63B	990835	1003937	21822	5928	21822	1012657	false
	CPOR 4	2319405	4457571	90668501	92771598	-	#26B63B	2138166	2103097	5928	1493	5928	2144094	false
	CPOR 4	4459064	6035123	89090110	90608767	-	#26B63B	1576059	1518657	1493	247	1493	1577552	false
	CPOR 4	6035370	11955044	83467540	88960868	-	#26B63B	5919674	5493328	247	-23413	247	5919921	false
	CPOR 4	11931631	11988008	83379235	83467540	+	#26B63B	56377	88305	-23413	-56377	-23413	32964	false
	CPOR 4	11931631	12963527	82166823	83216287	-	#26B63B	1031896	1049464	-56377	42338	-56377	975519	false
	CPOR 4	13005865	13426211	81638582	82090589	-	#26B63B	420346	452007	42338	-49332	42338	462684	false
	CPOR 4	13376879	13473015	59736023	59791576	-	#26B63B	96136	55553	-49332	75687	-49332	46804	false
	CPOR 4	13548702	13634726	81648678	81739803	-	#26B63B	86024	91125	75687	-79505	75687	161711	false
	CPOR 4	13555221	16484382	62283975	65255043	+	#26B63B	2929161	2971068	-79505	68655	-79505	2849656	false
	CPOR 4	16553037	17898398	65255043	66586907	+	#26B63B	1345361	1331864	68655	53040	68655	1414016	false

	CPOR 4	17951438	18356352	66883417	67355229	+	#26B63B	404914	471812	53040	0	53040	457954	false
	CPOR 4	18356352	21316843	67486319	70387507	+	#26B63B	2960491	2901188	0	125184	0	2960491	false
	CPOR 4	21442027	22038365	70390016	70960129	+	#26B63B	596338	570113	125184	-27295	125184	721522	false
	CPOR 4	22011070	22259147	81087991	81315374	-	#26B63B	248077	227383	-27295	53244	-27295	220782	false
	CPOR 4	22312391	22390278	81007594	81073752	-	#26B63B	77887	66158	53244	61140	53244	131131	false
	CPOR 4	22451418	22817113	80645562	81007594	-	#26B63B	365695	362032	61140	75968	61140	426835	false
	CPOR 4	22893081	25260454	78013591	80262544	-	#26B63B	2367373	2248953	75968	3162	75968	2443341	false
	CPOR 4	25263616	28059551	75245440	77893322	-	#26B63B	2795935	2647882	3162	1833	3162	2799097	false
	CPOR 4	28061384	28337726	74816037	75049285	-	#26B63B	276342	233248	1833	95761	1833	278175	false
	CPOR 4	28433487	30934925	72155914	74487481	-	#26B63B	2501438	2331567	95761		95761	2597199	false
	CPOR 21	30948727	31757369	9738812	10544768	+	#7444DF	808642	805956		95655			
	CPOR 21	31853024	33209282	8488014	9736131	-	#7444DF	1356258	1248117	95655	398	95655	1451913	false
	CPOR 21	33209680	34480440	7239073	8445669	+	#7444DF	1270760	1206596	398	-57636	398	1271158	false
	CPOR 21	34422804	35536784	6219709	7236023	+	#7444DF	1113980	1016314	-57636	-63296	-57636	1056344	false
	CPOR 21	35473488	37070381	4787784	6198791	-	#7444DF	1596893	1411007	-63296	2891	-63296	1533597	false
	CPOR 21	37073272	41942454	587177	4783262	+	#7444DF	4869182	4196085	2891	96099	2891	4872073	false
	CPOR 21	42038553	44716691	21985249	24480414	-	#7444DF	2678138	2495165	96099	280557	96099	2774237	false
	CPOR 21	44997248	45329786	21619340	21945499	-	#7444DF	332538	326159	280557	121073	280557	613095	false
	CPOR 21	45450859	46148468	30644688	31242615	-	#7444DF	697609	597927	121073	41507	121073	818682	false
	CPOR 21	46189975	46333714	24791040	24900605	-	#7444DF	143739	109565	41507	23455	41507	185246	false
	CPOR 21	46357169	46615687	309071	587642	-	#7444DF	258518	278571	23455	930	23455	281973	false
	CPOR 21	46616617	52519128	24891143	30508655	+	#7444DF	5902511	5617512	930	3547597	930	5903441	false
	CPOR 18	52555940	56312545	78351016	82009440	+	#FA2D80	3756605	3658424					
	CPOR 21	56066725	56131798	24608503	24659410	+	#7444DF	65073	50907	3547597	37536	3547597	3612670	false
	CPOR 21	56169334	57538147	13927550	15336780	-	#7444DF	1368813	1409230	37536	23315	37536	1406349	false
	CPOR 21	57561462	58076143	13331003	13855351	-	#7444DF	514681	524348	23315	-48499	23315	537996	false
	CPOR 21	58027644	58083910	13174380	13248120	+	#7444DF	56266	73740	-48499	-20548	-48499	7767	false
	CPOR 21	58063362	58419887	11592353	11936039	-	#7444DF	356525	343686	-20548	107613	-20548	335977	false
	CPOR 21	58527500	59496569	12108755	13027337	-	#7444DF	969069	918582	107613	19877	107613	1076682	false
	CPOR 21	59516446	62280051	15448423	18139735	+	#7444DF	2763605	2691312	19877	0	19877	2783482	false
	CPOR 21	62280051	62364231	18206136	18301127	+	#7444DF	84180	94991	0	128993	0	84180	false
	CPOR 21	62493224	64180580	18327989	20101749	+	#7444DF	1687356	1773760	128993	29	128993	1816349	false

	CPOR 21	64180609	64828114	20157152	20890131	+	#7444DF	647505	732979	29		29	647534	false
HGL 19	CPOR 17	62344	3190487	23975350	27311994	+	#02B4A9	3128143	3336644		61496			
	CPOR 17	3251983	3757666	27312044	27835230	+	#02B4A9	505683	523186	61496	-25116	61496	567179	false
	CPOR 17	3732550	4108458	27927400	28278570	+	#02B4A9	375908	351170	-25116	-325	-25116	350792	false
	CPOR 17	4108133	4158633	28312793	28362996	-	#02B4A9	50500	50203	-325	1530	-325	50175	false
	CPOR 17	4160163	6751973	28363906	31052020	+	#02B4A9	2591810	2688114	1530	5	1530	2593340	false
	CPOR 17	6751978	6816770	31243257	31305960	+	#02B4A9	64792	62703	5	19210	5	64797	false
	CPOR 17	6835980	7362897	23194169	23789122	-	#02B4A9	526917	594953	19210	3351	19210	546127	false
	CPOR 17	7366248	14661624	15004171	23127174	-	#02B4A9	7295376	8123003	3351	380526	3351	7298727	false
	CPOR 13	14842042	14920546	57024766	57112769	+	#11559D	78504	88003		11488			
	CPOR 13	14932034	14991819	57117948	57176031	-	#11559D	59785	58083	11488		11488	71273	false
	CPOR 17	15042150	15218713	23802158	23973634	-	#02B4A9	176563	171476	380526	10	380526	557089	false
	CPOR 17	15218723	16138096	31131452	32014858	+	#02B4A9	919373	883406	10	71217	10	919383	false
	CPOR 17	16209313	21157891	32071648	36834376	+	#02B4A9	4948578	4762728	71217	95010	71217	5019795	false
	CPOR 17	21252901	23751948	36849248	39190170	+	#02B4A9	2499047	2340922	95010	69107	95010	2594057	false
	CPOR 17	23821055	24528935	39185792	39919216	+	#02B4A9	707880	733424	69107	156190	69107	776987	false
	CPOR 17	24685125	25783171	40221414	41456448	-	#02B4A9	1098046	1235034	156190	-4274	156190	1254236	false
	CPOR 17	25778897	25964117	39919244	40163103	-	#02B4A9	185220	243859	-4274	23233	-4274	180946	false
	CPOR 17	25987350	27020828	43008953	43854739	+	#02B4A9	1033478	845786	23233	1452	23233	1056711	false
	CPOR 17	27022280	28507930	41555472	42981555	-	#02B4A9	1485650	1426083	1452	45094	1452	1487102	false
	CPOR 17	28553024	35621899	43843241	50445312	+	#02B4A9	7068875	6602071	45094	80585	45094	7113969	false
	CPOR 17	35702484	35778200	50514869	50601416	+	#02B4A9	75716	86547	80585	59486	80585	156301	false
	CPOR 17	35837686	35980845	51363426	51438814	+	#02B4A9	143159	75388	59486	151090	59486	202645	false
	CPOR 17	36131935	36209794	51437636	51557479	+	#02B4A9	77859	119843	151090	-21149	151090	228949	false
	CPOR 17	36188645	45666435	51993635	61136163	+	#02B4A9	9477790	9142528	-21149	-160370	-21149	9456641	false
	CPOR 17	45506065	45711028	61294811	61393937	+	#02B4A9	204963	99126	-160370	-133291	-160370	44593	false
	CPOR X	45506208	45608669	144353920	144431980	+	#01FC33	102461	78060		-102461			
	CPOR X	45506208	45703558	143425060	143551270	+	#01FC33	197350	126210	-102461		-102461	94889	false
	CPOR 17	45577737	45715764	61446993	61515471	+	#02B4A9	138027	68478	-133291	-99922	-133291	4736	false
	CPOR 17	45615842	45726310	61723467	61805989	+	#02B4A9	110468	82522	-99922	-77010	-99922	10546	false
	CPOR 17	45649300	47112257	61810823	63155124	+	#02B4A9	1462957	1344301	-77010	17	-77010	1385947	false
	CPOR 17	47112274	51518133	63215808	67218141	+	#02B4A9	4405859	4002333	17	-16956	17	4405876	false

	CPOR 17	51501177	51775180	68419113	68615475	-	#02B4A9	274003	196362	-16956	-252011	-16956	257047	false
	CPOR 17	51523169	52079772	69490266	69988455	-	#02B4A9	556603	498189	-252011	-239039	-252011	304592	false
	CPOR 17	51840733	52559623	70154247	70884440	+	#02B4A9	718890	730193	-239039	-541271	-239039	479851	false
	CPOR 17	52018352	52287380	69252691	69478240	-	#02B4A9	269028	225549	-541271	-115253	-541271	-272243	true
	CPOR 17	52172127	53021586	67270518	68028917	-	#02B4A9	849459	758399	-115253	-722648	-115253	734206	false
	CPOR 17	52298938	53021586	68619490	69201821	-	#02B4A9	722648	582331	-722648	-216935	-722648	0	true
	CPOR 17	52804651	54480564	70886088	72291345	+	#02B4A9	1675913	1405257	-216935	-75573	-216935	1458978	false
	CPOR 17	54404991	54590296	72249891	72397591	-	#02B4A9	185305	147700	-75573	-56856	-75573	109732	false
	CPOR 17	54533440	55477892	72332969	73314007	+	#02B4A9	944452	981038	-56856		-56856	887596	false
**HGL 2**	CPOR 14	15181	3102151	63443019	66656323	+	#FDB568	3086970	3213304		-43908			
	CPOR 14	3058243	3150846	66658526	66712183	-	#FDB568	92603	53657	-43908	-42601	-43908	48695	false
	CPOR 14	3108245	4369803	66714359	67916841	+	#FDB568	1261558	1202482	-42601	10101	-42601	1218957	false
	CPOR 14	4379904	7448505	67928374	70921822	-	#FDB568	3068601	2993448	10101	13978	10101	3078702	false
	CPOR 14	7462483	7532858	70917224	71036849	-	#FDB568	70375	119625	13978	29581	13978	84353	false
	CPOR 14	7562439	10025026	71013405	73461729	+	#FDB568	2462587	2448324	29581	8241166	29581	2492168	false
	CPOR 7	10009588	12815497	86124975	88894802	-	#FF0000	2805909	2769827		-34368			
	CPOR 7	12781129	14838676	84249557	86147715	-	#FF0000	2057547	1898158	-34368	87938	-34368	2023179	false
	CPOR 7	14926614	18278125	80964311	84195301	-	#FF0000	3351511	3230990	87938		87938	3439449	false
	CPOR 14	18266192	18360589	60958593	61032968	-	#FDB568	94397	74375	8241166	-94397	8241166	8335563	false
	CPOR 14	18266192	20279501	61691210	63416879	-	#FDB568	2013309	1725669	-94397	-1143503	-94397	1918912	false
	CPOR 14	19135998	19858828	59563281	60095848	-	#FDB568	722830	532567	-1143503	0	-1143503	-420673	true
	CPOR 14	19858828	24202014	55618435	59499138	-	#FDB568	4343186	3880703	0	-3312329	0	4343186	false
	CPOR 14	20889685	21047990	61258446	61379359	-	#FDB568	158305	120913	-3312329	419242	-3312329	-3154024	true
	CPOR 14	21467232	21611310	61032968	61125608	-	#FDB568	144078	92640	419242	2494225	419242	563320	false
	CPOR 14	24105535	24164884	61125780	61200158	+	#FDB568	59349	74378	2494225	37130	2494225	2553574	false
	CPOR 14	24202014	24502512	55384922	55618366	+	#FDB568	300498	233444	37130	60	37130	337628	false
	CPOR 14	24502572	31293456	49393742	55384922	-	#FDB568	6790884	5991180	60	116	60	6790944	false
	CPOR 14	31293572	31392798	49315548	49393734	+	#FDB568	99226	78186	116	0	116	99342	false
	CPOR 14	31392798	45445325	37452637	49307973	-	#FDB568	14052527	11855336	0	-1389496	0	14052527	false
	CPOR 14	44055829	46669664	33020998	35212467	+	#FDB568	2613835	2191469	-1389496	-1327072	-1389496	1224339	false
	CPOR 14	45342592	45444850	59499243	59562638	+	#FDB568	102258	63395	-1327072	-95305	-1327072	-1224814	true
	CPOR 14	45349545	45546025	31046736	31166569	-	#FDB568	196480	119833	-95305	-102226	-95305	101175	false

	CPOR 14	45443799	45593278	6953752	7048681	+	#FDB568	149479	94929	-102226	-62697	-102226	47253	false
	CPOR 14	45530581	46472562	36679546	37450647	-	#FDB568	941981	771101	-62697	-603414	-62697	879284	false
	CPOR 14	45869148	46666329	32305142	33019823	+	#FDB568	797181	714681	-603414	-193766	-603414	193767	false
	CPOR 14	46472563	47812087	31182746	32305137	-	#FDB568	1339524	1122391	-193766	412619	-193766	1145758	false
	CPOR 14	48224706	48355538	30698870	30872753	-	#FDB568	130832	173883	412619	100405	412619	543451	false
	CPOR 14	48455943	48610711	30726045	30836407	-	#FDB568	154768	110362	100405	54099	100405	255173	false
	CPOR 14	48664810	51200521	28624923	30702460	-	#FDB568	2535711	2077537	54099	139184	54099	2589810	false
	CPOR 14	51339705	61419026	19702708	28459335	-	#FDB568	10079321	8756627	139184	-1590266	139184	10218505	false
	CPOR 14	59828760	60084714	20652066	20817722	-	#FDB568	255954	165656	-1590266	60264	-1590266	-1334312	true
	CPOR 14	60144978	60379638	20864436	21059912	-	#FDB568	234660	195476	60264	1085573	60264	294924	false
	CPOR 14	61465211	64553216	17141471	19617361	-	#FDB568	3088005	2475890	1085573	170023	1085573	4173578	false
	CPOR 14	64723239	70674580	11399978	17125799	-	#FDB568	5951341	5725821	170023	6935	170023	6121364	false
	CPOR 14	70681515	70830308	11281830	11399963	+	#FDB568	148793	118133	6935	25691	6935	155728	false
	CPOR 14	70855999	74548275	7897201	11281830	-	#FDB568	3692276	3384629	25691	293159	25691	3717967	false
	CPOR 1	74548783	74663080	23740175	23808785	+	#A6F1FC	114297	68610		739			
	CPOR 1	74663819	74768003	23964618	24083717	+	#A6F1FC	104184	119099	739	8127216	739	104923	false
	CPOR 14	74841434	75465397	7295252	7883235	-	#FDB568	623963	587983	293159	-17019	293159	917122	false
	CPOR 14	75448378	78851730	3643779	6832682	-	#FDB568	3403352	3188903	-17019	-37748	-17019	3386333	false
	CPOR 14	78813982	80061088	2488263	3643386	-	#FDB568	1247106	1155123	-37748	243250	-37748	1209358	false
	CPOR 14	80304338	81912042	674342	2180896	-	#FDB568	1607704	1506554	243250	124515	243250	1850954	false
	CPOR 14	82036557	82581128	111724	668899	+	#FDB568	544571	557175	124515		124515	669086	false
	CPOR 1	82895219	89881596	40445721	47078620	-	#A6F1FC	6986377	6632899	8127216	-184929	8127216	15113593	false
	CPOR 1	89696667	90591611	39585557	40445719	+	#A6F1FC	894944	860162	-184929	-894944	-184929	710015	false
	CPOR 1	89696667	90743256	35696068	36512563	-	#A6F1FC	1046589	816495	-894944	-1046589	-894944	151645	false
	CPOR 1	89696667	90921750	37401118	38482385	-	#A6F1FC	1225083	1081267	-1046589	-1051525	-1046589	178494	false
	CPOR 1	89870225	90930725	36512563	37350295	-	#A6F1FC	1060500	837732	-1051525	-128346	-1051525	8975	false
	CPOR 1	90802379	100059089	27061153	35696068	-	#A6F1FC	9256710	8634915	-128346	-7188	-128346	9128364	false
	CPOR 1	100051901	100210348	26863859	27019400	-	#A6F1FC	158447	155541	-7188	29235	-7188	151259	false
	CPOR 1	100239583	103443980	23894306	26811302	-	#A6F1FC	3204397	2916996	29235	478	29235	3233632	false
	CPOR 1	103444458	106064733	21476826	23808955	-	#A6F1FC	2620275	2332129	478	0	478	2620753	false
	CPOR 1	106064733	106568913	20919428	21411860	-	#A6F1FC	504180	492432	0	50371	0	504180	false
	CPOR 1	106619284	108500330	19418217	20906280	-	#A6F1FC	1881046	1488063	50371	355	50371	1931417	false

	CPOR 1	108500685	120810205	7388693	18961865	-	#A6F1FC	12309520	11573172	355	-33840	355	12309875	false
	CPOR 27	120776365	120841872	34313325	34432881	-	#0800AC	65507	119556		-35858			
	CPOR 1	120776365	120843099	6332332	6481187	+	#A6F1FC	66734	148855	-33840		-33840	32894	false
	CPOR 27	120806014	121447553	33697102	34310136	-	#0800AC	641539	613034	-35858	165694	-35858	605681	false
	CPOR 27	121613247	122680125	32536840	33520022	-	#0800AC	1066878	983182	165694	58846	165694	1232572	false
	CPOR 27	122738971	123838947	31554026	32508497	-	#0800AC	1099976	954471	58846	-324500	58846	1158822	false
	CPOR 27	123514447	123664993	31195245	31320304	-	#0800AC	150546	125059	-324500	-31569	-324500	-173954	true
	CPOR 27	123633424	123695413	30978564	31046225	-	#0800AC	61989	67661	-31569	-36064	-31569	30420	false
	CPOR 27	123659349	123906865	31314526	31597223	-	#0800AC	247516	282697	-36064	-209323	-36064	211452	false
	CPOR 27	123697542	123885162	31131495	31320304	-	#0800AC	187620	188809	-209323	-175449	-209323	-21703	true
	CPOR 27	123709713	123911854	30729010	30899324	-	#0800AC	202141	170314	-175449	-11323	-175449	26692	false
	CPOR 27	123900531	123953329	31478308	31574957	-	#0800AC	52798	96649	-11323	13849	-11323	41475	false
	CPOR 27	123967178	124023527	30900783	30952048	-	#0800AC	56349	51265	13849	-56349	13849	70198	false
	CPOR 27	123967178	124041023	31069862	31127269	-	#0800AC	73845	57407	-56349	-73626	-56349	17496	false
	CPOR 27	123967397	124204714	30613231	30774212	-	#0800AC	237317	160981	-73626	-28755	-73626	163691	false
	CPOR 27	124175959	124312295	30273642	30456231	-	#0800AC	136336	182589	-28755	-12414	-28755	107581	false
	CPOR 27	124299881	125023848	29568750	30233211	-	#0800AC	723967	664461	-12414	1553195	-12414	711553	false
	CPOR 4	124916784	125001930	36979792	37090048	+	#26B63B	85146	110256		73296			
	CPOR 4	125075226	125166750	71871226	71969349	-	#26B63B	91524	98123	73296	262110	73296	164820	false
	CPOR 4	125428860	125695513	37455056	37660633	+	#26B63B	266653	205577	262110	-259595	262110	528763	false
	CPOR 4	125435918	125791488	71555728	71859946	-	#26B63B	355570	304218	-259595	-33553	-259595	95975	false
	CPOR 12	125508473	125614912	88586572	88655768	-	#9B0496	106439	69196		673630			
	CPOR 4	125757935	125826481	37661271	37742024	+	#26B63B	68546	80753	-33553	36829	-33553	34993	false
	CPOR 4	125863310	125968010	37726132	37796299	+	#26B63B	104700	70167	36829	-28375	36829	141529	false
	CPOR 4	125939635	126001282	71479077	71554902	-	#26B63B	61647	75825	-28375	323762	-28375	33272	false
	CPOR 12	126288542	126409867	88496189	88569438	-	#9B0496	121325	73249	673630		673630	794955	false
	CPOR 4	126325044	126465327	71256979	71362134	-	#26B63B	140283	105155	323762	1280441	323762	464045	false
	CPOR 27	126577043	126744917	28278665	28384824	+	#0800AC	167874	106159	1553195	115776	1553195	1721069	false
	CPOR 27	126860693	126995564	28378526	28566149	+	#0800AC	134871	187623	115776	-47397	115776	250647	false
	CPOR 27	126948167	127172856	28629581	28821914	-	#0800AC	224689	192333	-47397	-79189	-47397	177292	false
	CPOR 27	127093667	127388167	28828655	29154904	+	#0800AC	294500	326249	-79189	44867	-79189	215311	false
	CPOR 27	127433034	127517867	29330677	29473646	-	#0800AC	84833	142969	44867	104462	44867	129700	false

	CPOR 27	127622329	127720101	29400702	29465509	+	#0800AC	97772	64807	104462		104462	202234	false
	CPOR 4	127745768	139047718	26186265	36981094	-	#26B63B	11301950	10794829	1280441	56479	1280441	12582391	false
	CPOR 4	139104197	139225271	32804649	32901711	+	#26B63B	121074	97062	56479	69098	56479	177553	false
	CPOR 4	139294369	139847614	25691209	26115504	+	#26B63B	553245	424295	69098		69098	622343	false
**HGL 20**	CPOR 1	14433	22112522	131146388	152093744	-	#A6F1FC	22098089	20947356		-200235			
	CPOR 1	21912287	22112522	131106137	131255977	-	#A6F1FC	200235	149840	-200235	-200235	-200235	0	true
	CPOR 1	21912287	22137760	131041920	131205636	-	#A6F1FC	225473	163716	-200235	-185213	-200235	25238	false
	CPOR 1	21952547	24494969	128760674	131132511	-	#A6F1FC	2542422	2371837	-185213	-12672	-185213	2357209	false
	CPOR 1	24482297	24580827	96300952	96352441	+	#A6F1FC	98530	51489	-12672	-67502	-12672	85858	false
	CPOR 1	24513325	24635912	96382288	96434202	+	#A6F1FC	122587	51914	-67502	-33662	-67502	55085	false
	CPOR 1	24602250	24716516	96433640	96604163	+	#A6F1FC	114266	170523	-33662	56953	-33662	80604	false
	CPOR 1	24773469	28321157	96826298	100105143	+	#A6F1FC	3547688	3278845	56953	115423	56953	3604641	false
	CPOR 1	28436580	33082079	100105178	104201189	+	#A6F1FC	4645499	4096011	115423	63208	115423	4760922	false
	CPOR 1	33145287	39896105	104176877	109886216	+	#A6F1FC	6750818	5709339	63208	-74521	63208	6814026	false
	CPOR 1	39821584	44206349	109967709	113971603	+	#A6F1FC	4384765	4003894	-74521	2939	-74521	4310244	false
	CPOR 1	44209288	46396870	114059495	116311134	+	#A6F1FC	2187582	2251639	2939	82514	2939	2190521	false
	CPOR 1	46479384	46612562	116366196	116534830	+	#A6F1FC	133178	168634	82514	152117	82514	215692	false
	CPOR 1	46764679	46892302	116838972	116953429	+	#A6F1FC	127623	114457	152117	7170	152117	279740	false
	CPOR 1	46899472	54903895	117021165	125013361	+	#A6F1FC	8004423	7992196	7170	20397	7170	8011593	false
	CPOR 1	54924292	55080681	125282928	125500071	+	#A6F1FC	156389	217143	20397	80906	20397	176786	false
	CPOR 1	55161587	55413805	125502378	125717329	-	#A6F1FC	252218	214951	80906	23122	80906	333124	false
	CPOR 1	55436927	57276848	125725225	127728414	+	#A6F1FC	1839921	2003189	23122	16525	23122	1863043	false
	CPOR 1	57293373	57405283	127728414	127805360	-	#A6F1FC	111910	76946	16525	-40191	16525	128435	false
	CPOR 1	57365092	57435174	91991552	92078659	-	#A6F1FC	70082	87107	-40191	-70082	-40191	29891	false
	CPOR 1	57365092	57442711	128148636	128252281	+	#A6F1FC	77619	103645	-70082	-77619	-70082	7537	false
	CPOR 1	57365092	57481221	128473913	128632358	-	#A6F1FC	116129	158445	-77619	-116129	-77619	38510	false
	CPOR 1	57365092	58280763	92030651	93004689	+	#A6F1FC	915671	974038	-116129	-881237	-116129	799542	false
	CPOR 1	57399526	57481221	128258711	128360966	-	#A6F1FC	81695	102255	-881237	831040	-881237	-799542	true
	CPOR 1	58312261	58914019	93054773	93738288	+	#A6F1FC	601758	683515	831040	38707	831040	1432798	false
	CPOR 1	58952726	59365610	93869305	94330974	+	#A6F1FC	412884	461669	38707	1	38707	451591	false
	CPOR 1	59365611	59544019	94330974	94579501	-	#A6F1FC	178408	248527	1	3	1	178409	false
	CPOR 1	59544022	60005380	94579505	95090391	+	#A6F1FC	461358	510886	3	-57917	3	461361	false

	CPOR 1	59947463	60059327	95194344	95282652	-	#A6F1FC	111864	88308	-57917	-69220	-57917	53947	false
	CPOR 1	59990107	60420832	95361071	95835278	+	#A6F1FC	430725	474207	-69220	-5166	-69220	361505	false
	CPOR 1	60415666	60680689	95888330	96247769	+	#A6F1FC	265023	359439	-5166	3595425	-5166	259857	false
	CPOR 4	60811726	62126870	38010038	39515458	-	#26B63B	1315144	1505420		9178			
	CPOR 4	62136048	62640601	39515458	40090108	+	#26B63B	504553	574650	9178	-10363	9178	513731	false
	CPOR 4	62630238	62728992	40092145	40227416	-	#26B63B	98754	135271	-10363	-46234	-10363	88391	false
	CPOR 4	62682758	63510366	40235576	41204935	+	#26B63B	827608	969359	-46234		-46234	781374	false
	CPOR 1	64276114	64340779	180626588	180721942	-	#A6F1FC	64665	95354	3595425	826395	3595425	3660090	false
	CPOR 1	65167174	65269524	180626588	180709782	+	#A6F1FC	102350	83194	826395		826395	928745	false
**HGL 21**	CPOR 2	469778	570800	145756467	145850575	-	#7FFF00	101022	94108		119787			
	CPOR 2	690587	35400430	113489839	145753126	-	#7FFF00	34709843	32263287	119787	89542	119787	34829630	false
	CPOR 2	35489972	52331615	98085688	113470442	-	#7FFF00	16841643	15384754	89542	6035	89542	16931185	false
	CPOR 2	52337650	52405755	97994057	98080650	+	#7FFF00	68105	86593	6035	2075	6035	74140	false
	CPOR 2	52407830	52463018	97939117	97992836	-	#7FFF00	55188	53719	2075	63098	2075	57263	false
	CPOR 2	52526116	52792527	97643095	97916449	-	#7FFF00	266411	273354	63098	2187	63098	329509	false
	CPOR 2	52794714	52872757	97570318	97643095	+	#7FFF00	78043	72777	2187	1229	2187	80230	false
	CPOR 2	52873986	61069490	89952647	97569396	-	#7FFF00	8195504	7616749	1229	50160	1229	8196733	false
	CPOR 2	61119650	64742718	86667608	89951413	-	#7FFF00	3623068	3283805	50160	43	50160	3673228	false
	CPOR 2	64742761	66938796	84498446	86592171	-	#7FFF00	2196035	2093725	43	19535	43	2196078	false
	CPOR 2	66958331	68985347	82592974	84430031	-	#7FFF00	2027016	1837057	19535	4096	19535	2046551	false
	CPOR 2	68989443	75322653	76600128	82532622	-	#7FFF00	6333210	5932494	4096	45882	4096	6337306	false
	CPOR 2	75368535	76072257	75432307	76070451	-	#7FFF00	703722	638144	45882	386754	45882	749604	false
	CPOR 2	76459011	76642786	76404483	76542256	+	#7FFF00	183775	137773	386754	117717	386754	570529	false
	CPOR 2	76760503	77190070	74927439	75411087	-	#7FFF00	429567	483648	117717	4663	117717	547284	false
	CPOR 2	77194733	78975180	73173868	74866429	-	#7FFF00	1780447	1692561	4663	24493	4663	1785110	false
	CPOR 2	78999673	80564350	71654297	73123673	+	#7FFF00	1564677	1469376	24493		24493	1589170	false
**HGL 22**	CPOR 2	319974	373682	30756333	30812905	-	#7FFF00	53708	56572		853317			
	CPOR 1	869321	928522	180853011	180908807	+	#A6F1FC	59201	55796		984647			
	CPOR 11	879863	948268	15492887	15636842	-	#4C0851	68405	143955		2026803			
	CPOR 2	1226999	1293122	32210461	32299739	-	#7FFF00	66123	89278	853317	-65831	853317	919440	false
	CPOR 2	1227291	1304989	31637266	31704555	+	#7FFF00	77698	67289	-65831	310383	-65831	11867	false
	CPOR 2	1615372	1673106	32118854	32200387	+	#7FFF00	57734	81533	310383	496471	310383	368117	false

	CPOR 1	1913169	1981367	145640056	145705943	-	#A6F1FC	68198	65887	984647	38043961	984647	1052845	false
	CPOR 2	2169577	2360401	29544832	29732363	+	#7FFF00	190824	187531	496471	80934	496471	687295	false
	CPOR 2	2441335	2532457	29271680	29329559	-	#7FFF00	91122	57879	80934	80851	80934	172056	false
	CPOR 2	2613308	2671362	9399536	9465431	-	#7FFF00	58054	65895	80851		80851	138905	false
	CPOR 11	2975071	3134812	15897111	16082197	+	#4C0851	159741	185086	2026803	545	2026803	2186544	false
	CPOR 11	3135357	3418118	16154075	16425846	+	#4C0851	282761	271771	545	79306	545	283306	false
	CPOR 11	3497424	4351430	17178020	18159100	+	#4C0851	854006	981080	79306	-15512	79306	933312	false
	CPOR 11	4335918	4428147	18238420	18377169	+	#4C0851	92229	138749	-15512	-17315	-15512	76717	false
	CPOR 11	4410832	4477434	18412942	18486871	-	#4C0851	66602	73929	-17315	-50873	-17315	49287	false
	CPOR 11	4426561	4631840	18672258	18773105	+	#4C0851	205279	100847	-50873	0	-50873	154406	false
	CPOR 11	4631840	4754205	18613803	18678327	-	#4C0851	122365	64524	0	34929	0	122365	false
	CPOR 11	4789134	4866275	18466102	18530661	+	#4C0851	77141	64559	34929	124402	34929	112070	false
	CPOR 11	4990677	5111283	18763448	18897504	+	#4C0851	120606	134056	124402	-51767	124402	245008	false
	CPOR 11	5059516	5407661	18973884	19321166	+	#4C0851	348145	347282	-51767	39000	-51767	296378	false
	CPOR 11	5446661	5666496	19472936	19755774	+	#4C0851	219835	282838	39000	-18814	39000	258835	false
	CPOR 11	5647682	5712627	20186112	20238377	+	#4C0851	64945	52265	-18814	-11436	-18814	46131	false
	CPOR 11	5701191	6006708	20369401	20890973	+	#4C0851	305517	521572	-11436	-20646	-11436	294081	false
	CPOR 11	5986062	6449837	20932616	21319135	+	#4C0851	463775	386519	-20646	-85133	-20646	443129	false
	CPOR 11	6364704	6527336	21332960	21467931	-	#4C0851	162632	134971	-85133	17832	-85133	77499	false
	CPOR 11	6545168	6680886	21314706	21408849	-	#4C0851	135718	94143	17832	-37924	17832	153550	false
	CPOR 11	6642962	6795847	21490310	21619456	-	#4C0851	152885	129146	-37924	-76221	-37924	114961	false
	CPOR 11	6719626	6813646	21314690	21407749	-	#4C0851	94020	93059	-76221	121134	-76221	17799	false
	CPOR 11	6934780	7239330	21540918	21871118	+	#4C0851	304550	330200	121134	-16110	121134	425684	false
	CPOR 11	7223220	8006523	21921904	22769229	+	#4C0851	783303	847325	-16110	-57477	-16110	767193	false
	CPOR 11	7949046	8153735	23387677	23543414	-	#4C0851	204689	155737	-57477	-107123	-57477	147212	false
	CPOR 11	8046612	8155638	23763459	23890666	+	#4C0851	109026	127207	-107123	-64025	-107123	1903	false
	CPOR 11	8091613	8233378	23196814	23370260	-	#4C0851	141765	173446	-64025	74311	-64025	77740	false
	CPOR 21	8129636	8181156	21248416	21324507	+	#7444DF	51520	76091		-19519			
	CPOR 6	8154237	8467208	7324145	7549091	-	#7F00FF	312971	224946		-119345			
	CPOR 21	8161637	8238617	21358151	21418773	+	#7444DF	76980	60622	-19519	5528	-19519	57461	false
	CPOR 21	8244145	8339056	10582813	10638346	+	#7444DF	94911	55533	5528	294820	5528	100439	false
	CPOR 11	8307689	8604242	22977461	23183912	-	#4C0851	296553	206451	74311	-13037	74311	370864	false

	CPOR 6	8347863	8578861	48161060	48364986	-	#7F00FF	230998	203926	-119345	31446625	-119345	111653	false
	CPOR 11	8591205	8697564	18532348	18583006	+	#4C0851	106359	50658	-13037	-95933	-13037	93322	false
	CPOR 11	8601631	8743189	24763035	24820776	+	#4C0851	141558	57741	-95933	-64613	-95933	45625	false
	CPOR 21	8633876	8687009	10779011	10845014	+	#7444DF	53133	66003	294820		294820	347953	false
	CPOR 11	8678576	8855946	23546595	23672977	+	#4C0851	177370	126382	-64613	132118	-64613	112757	false
	CPOR 7	8988064	9048507	25520880	25594887	+	#FF0000	60443	74007	8040315	574281	8040315	8100758	false
	CPOR 11	8988064	9063608	15506705	15626231	+	#4C0851	75544	119526	132118	450627	132118	207662	false
	CPOR 11	9514235	9807160	15435293	15730299	-	#4C0851	292925	295006	450627	711915	450627	743552	false
	CPOR 7	9622788	9678568	25493900	25591703	-	#FF0000	55780	97803	574281		574281	630061	false
	CPOR 11	10519075	10650523	14406832	14486606	-	#4C0851	131448	79774	711915	-131448	711915	843363	false
	CPOR 11	10519075	10662421	8664354	8741926	-	#4C0851	143346	77572	-131448	8903	-131448	11898	false
	CPOR 11	10671324	10731411	24529094	24581934	+	#4C0851	60087	52840	8903	64833	8903	68990	false
	CPOR 8	10722717	10790721	94714891	94765353	+	#1036F2	68004	50462		-67882			
	CPOR 8	10722839	10790764	94599925	94666344	-	#1036F2	67925	66419	-67882		-67882	43	false
	CPOR 5	10742301	10812490	17385069	17466597	+	#09FC8D	70189	81528		183042			
	CPOR 11	10796244	10858149	8612275	8664354	+	#4C0851	61905	52079	64833	-50227	64833	126738	false
	CPOR 11	10807922	10859268	28216890	28269347	+	#4C0851	51346	52457	-50227	71816	-50227	1119	false
	CPOR 11	10931084	11014882	13894768	13953587	-	#4C0851	83798	58819	71816	-43355	71816	155614	false
	CPOR 11	10971527	11051660	14899528	14953601	-	#4C0851	80133	54073	-43355	80486	-43355	36778	false
	CPOR 5	10995532	11066563	17530915	17587148	+	#09FC8D	71031	56233	183042	469796	183042	254073	false
	CPOR 11	11132146	11341086	14117199	14178626	+	#4C0851	208940	61427	80486	-176857	80486	289426	false
	CPOR 11	11164229	11406769	28365679	28509557	+	#4C0851	242540	143878	-176857	-123241	-176857	65683	false
	CPOR 11	11283528	11342091	24633058	24684321	+	#4C0851	58563	51263	-123241	32823	-123241	-64678	true
	CPOR 11	11374914	11510931	28566614	28641310	-	#4C0851	136017	74696	32823	-51275	32823	168840	false
	CPOR 11	11459656	20287544	28610174	37429095	+	#4C0851	8827888	8818921	-51275	5934	-51275	8776613	false
	CPOR 5	11536359	11609544	17625243	17695103	+	#09FC8D	73185	69860	469796		469796	542981	false
	CPOR 11	20293478	20358316	37429719	37484546	-	#4C0851	64838	54827	5934	66328	5934	70772	false
	CPOR 11	20424644	23918501	37533259	40742004	+	#4C0851	3493857	3208745	66328	7520	66328	3560185	false
	CPOR 11	23926021	24029014	40752641	40856771	-	#4C0851	102993	104130	7520	12832	7520	110513	false
	CPOR 11	24041846	24979579	40808528	41746314	+	#4C0851	937733	937786	12832	100416	12832	950565	false
	CPOR 11	25079995	26455154	41753904	43198648	+	#4C0851	1375159	1444744	100416	22844	100416	1475575	false
	CPOR 11	26477998	27630047	43513702	44591305	+	#4C0851	1152049	1077603	22844	-122024	22844	1174893	false

	CPOR 11	27508023	31800173	44679168	48750376	+	#4C0851	4292150	4071208	-122024	70710	-122024	4170126	false
	CPOR 11	31870883	33249489	48758290	50058044	+	#4C0851	1378606	1299754	70710	45815	70710	1449316	false
	CPOR 11	33295304	35216687	50111204	51833106	+	#4C0851	1921383	1721902	45815	18875	45815	1967198	false
	CPOR 11	35235562	35600079	51923377	52160032	+	#4C0851	364517	236655	18875	18656604	18875	383392	false
	CPOR 12	35627141	37329254	2221341	4053191	+	#9B0496	1702113	1831850		63668			
	CPOR 12	37392922	39487995	4117545	6391459	+	#9B0496	2095073	2273914	63668		63668	2158741	false
	CPOR 17	39501248	44799704	164947	5362390	+	#02B4A9	5298456	5197443		462			
	CPOR 1	40025328	40117074	196461938	196521429	-	#A6F1FC	91746	59491	38043961	486879	38043961	38135707	false
	CPOR 1	40603953	40777487	196322955	196443530	-	#A6F1FC	173534	120575	486879		486879	660413	false
	CPOR 17	44800166	49321418	5441027	9940015	+	#02B4A9	4521252	4498988	462	84691	462	4521714	false
	CPOR 17	49406109	49548304	10164576	10299042	-	#02B4A9	142195	134466	84691	0	84691	226886	false
	CPOR 17	49548304	54244450	10299071	14979505	+	#02B4A9	4696146	4680434	0		0	4696146	false
	CPOR 11	54256683	54370929	52222541	52305010	+	#4C0851	114246	82469	18656604	59424	18656604	18770850	false
	CPOR 11	54430353	54672085	52357680	52576619	-	#4C0851	241732	218939	59424		59424	301156	false
	CPOR 4	54696951	55343005	49466560	50051444	+	#26B63B	646054	584884		-260518			
	CPOR 4	55082487	55161765	49990425	50072123	-	#26B63B	79278	81698	-260518	64245	-260518	-181240	true
	CPOR 4	55226010	55446028	49836881	49935888	-	#26B63B	220018	99007	64245	-175881	64245	284263	false
	CPOR 4	55270147	55986441	50007888	50702266	+	#26B63B	716294	694378	-175881	-10238	-175881	540413	false
	CPOR 4	55976203	56332259	50749045	51229435	+	#26B63B	356056	480390	-10238	2470	-10238	345818	false
	CPOR 4	56334729	60552044	51505113	55403389	+	#26B63B	4217315	3898276	2470	71232	2470	4219785	false
	CPOR 4	60623276	60891998	55418061	55689766	+	#26B63B	268722	271705	71232	27144	71232	339954	false
	CPOR 4	60919142	65018153	55762526	59735858	+	#26B63B	4099011	3973332	27144	3870	27144	4126155	false
	CPOR 4	65022023	67196853	59838227	61820492	+	#26B63B	2174830	1982265	3870		3870	2178700	false
**HGL 23**	CPOR 23	19468	265333	141132	414265	+	#E181CE	245865	273133		118174			
	CPOR 23	383507	1794291	423368	1793268	+	#E181CE	1410784	1369900	118174	-13263	118174	1528958	false
	CPOR 23	1781028	1905714	1788759	1867776	-	#E181CE	124686	79017	-13263	2765	-13263	111423	false
	CPOR 23	1908479	17433939	1882375	16859081	+	#E181CE	15525460	14976706	2765	0	2765	15528225	false
	CPOR 23	17433939	19347185	16914244	18750814	+	#E181CE	1913246	1836570	0	38531	0	1913246	false
	CPOR 23	19385716	21711100	18988140	21200050	+	#E181CE	2325384	2211910	38531	52080	38531	2363915	false
	CPOR 23	21763180	25142155	21232484	24428580	+	#E181CE	3378975	3196096	52080	0	52080	3431055	false
	CPOR 23	25142155	25197990	24428580	24479816	-	#E181CE	55835	51236	0	14	0	55835	false
	CPOR 23	25198004	47238072	24479828	45546971	+	#E181CE	22040068	21067143	14	53114	14	22040082	false

	CPOR 23	47291186	51309981	45551312	49221357	+	#E181CE	4018795	3670045	53114	-40507	53114	4071909	false
	CPOR 23	51269474	51723647	49220573	49589676	+	#E181CE	454173	369103	-40507	-125197	-40507	413666	false
	CPOR 23	51598450	51725736	49589935	49678494	-	#E181CE	127286	88559	-125197	0	-125197	2089	false
	CPOR 23	51725736	52141491	52038939	52421388	+	#E181CE	415755	382449	0	-168991	0	415755	false
	CPOR 23	51972500	52964792	50891481	51768839	-	#E181CE	992292	877358	-168991	-362462	-168991	823301	false
	CPOR 23	52602330	53415983	49679099	50499899	-	#E181CE	813653	820800	-362462	-347670	-362462	451191	false
	CPOR 23	53068313	57103094	52426939	56175058	+	#E181CE	4034781	3748119	-347670	0	-347670	3687111	false
	CPOR 23	57103094	59620344	56232318	58635496	+	#E181CE	2517250	2403178	0	1113	0	2517250	false
	CPOR 23	59621457	59704325	58640885	58734530	-	#E181CE	82868	93645	1113	0	1113	83981	false
	CPOR 23	59704325	66129806	58735011	64735842	+	#E181CE	6425481	6000831	0	72627	0	6425481	false
	CPOR 23	66202433	66569371	64837600	65177397	+	#E181CE	366938	339797	72627	3569	72627	439565	false
	CPOR 23	66572940	69225831	65311024	67944660	+	#E181CE	2652891	2633636	3569	50025	3569	2656460	false
	CPOR 23	69275856	69472106	67964453	68190443	+	#E181CE	196250	225990	50025		50025	246275	false
**HGL 24**	CPOR 27	41738	108926	27993570	28068761	-	#0800AC	67188	75191		-25373			
	CPOR 27	83553	5748218	22246987	27851383	-	#0800AC	5664665	5604396	-25373	117553	-25373	5639292	false
	CPOR 27	5865771	12669605	15310813	22119870	-	#0800AC	6803834	6809057	117553	5900423	117553	6921387	false
	CPOR 1	12691544	14464838	4054093	6006258	-	#A6F1FC	1773294	1952165		-49045			
	CPOR 1	14415793	18602371	36451	4054093	-	#A6F1FC	4186578	4017642	-49045		-49045	4137533	false
	CPOR 27	18570028	35824115	382	14633358	+	#0800AC	17254087	14632976	5900423		5900423	23154510	false
	CPOR 4	35939705	36080260	41491042	41595709	-	#26B63B	140555	104667		90595			
	CPOR 4	36170855	36627140	41648012	42135275	+	#26B63B	456285	487263	90595	7593	90595	546880	false
	CPOR 4	36634733	43278881	42226243	48698626	+	#26B63B	6644148	6472383	7593	-135205	7593	6651741	false
	CPOR 4	43143676	43487719	48708390	48979152	+	#26B63B	344043	270762	-135205		-135205	208838	false
	CPOR 11	43585302	44144085	55908892	56362533	-	#4C0851	558783	453641		51278			
	CPOR 11	44195363	45276126	55159917	55888892	-	#4C0851	1080763	728975	51278	58783	51278	1132041	false
	CPOR 11	45334909	45527048	55004181	55157149	-	#4C0851	192139	152968	58783	56149	58783	250922	false
	CPOR 11	45583197	45705859	54892364	54990442	-	#4C0851	122662	98078	56149	105144	56149	178811	false
	CPOR 11	45811003	47546816	53289394	54724625	+	#4C0851	1735813	1435231	105144	18178	105144	1840957	false
	CPOR 11	47564994	47652328	53086454	53202447	-	#4C0851	87334	115993	18178	23487	18178	105512	false
	CPOR 11	47675815	49654116	56403031	57845444	+	#4C0851	1978301	1442413	23487		23487	2001788	false
**HGL 25**	CPOR 27	39669	145348	50631335	50726438	+	#0800AC	105679	95103		2572			
	CPOR 27	147920	599465	51005817	51406101	+	#0800AC	451545	400284	2572	952	2572	454117	false

	CPOR 27	600417	803474	50422462	50621171	-	#0800AC	203057	198709	952	5829	952	204009	false
	CPOR 27	809303	6702690	44533586	50363811	-	#0800AC	5893387	5830225	5829	594	5829	5899216	false
	CPOR 27	6703284	15214610	36455088	44476275	-	#0800AC	8511326	8021187	594	1837122	594	8511920	false
	CPOR 31	15216091	17051645	11843659	13485511	-	#DB00F9	1835554	1641852		80315			
	CPOR 27	17051732	17117518	42309773	42402767	+	#0800AC	65786	92994	1837122	35369956	1837122	1902908	false
	CPOR 31	17131960	24081696	5213404	11841431	-	#DB00F9	6949736	6628027	80315	4330	80315	7030051	false
	CPOR 31	24086026	24329734	4969774	5142274	-	#DB00F9	243708	172500	4330	2795	4330	248038	false
	CPOR 31	24332529	24907046	2438928	2987577	+	#DB00F9	574517	548649	2795	51296	2795	577312	false
	CPOR 31	24958342	27034228	2993642	4895725	+	#DB00F9	2075886	1902083	51296	402	51296	2127182	false
	CPOR 31	27034630	29773827	7	2330578	-	#DB00F9	2739197	2330571	402	23206	402	2739599	false
	CPOR 31	29797033	39020799	27323248	35772809	-	#DB00F9	9223766	8449561	23206	55712	23206	9246972	false
	CPOR 31	39076511	47739859	18743049	27267423	-	#DB00F9	8663348	8524374	55712	42251	55712	8719060	false
	CPOR 31	47782110	50450450	15772719	18673864	-	#DB00F9	2668340	2901145	42251	-21567	42251	2710591	false
	CPOR 31	50428883	52487474	13494116	15735007	-	#DB00F9	2058591	2240891	-21567		-21567	2037024	false
	CPOR 27	52487474	53948068	34695399	36454227	-	#0800AC	1460594	1758828	35369956		35369956	36830550	false
**HGL 26**	CPOR 13	16515	935755	65085019	65924859	+	#11559D	919240	839840		6692			
	CPOR 13	942447	3521132	66117109	68830010	+	#11559D	2578685	2712901	6692	55203	6692	2585377	false
	CPOR 13	3576335	10289923	68830415	76037444	+	#11559D	6713588	7207029	55203	58617	55203	6768791	false
	CPOR 13	10348540	10427360	76049690	76100318	+	#11559D	78820	50628	58617		58617	137437	false
	CPOR 8	10537485	12708642	88884158	90726212	-	#1036F2	2171157	1842054		-4565			
	CPOR 8	12704077	15632893	98964964	101761777	-	#1036F2	2928816	2796813	-4565	-58086	-4565	2924251	false
	CPOR 8	15574807	15699489	98093976	98221928	+	#1036F2	124682	127952	-58086	-98221	-58086	66596	false
	CPOR 8	15601268	16891783	94773372	96004299	-	#1036F2	1290515	1230927	-98221	287503	-98221	1192294	false
	CPOR 8	17179286	17239062	103782101	103862044	-	#1036F2	59776	79943	287503	959348	287503	347279	false
	CPOR 12	17319782	18192136	6400890	7252038	+	#9B0496	872354	851148		1597214			
	CPOR 8	18198410	18313478	103258482	103349682	-	#1036F2	115068	91200	959348	0	959348	1074416	false
	CPOR 8	18313478	18465323	90731384	90907911	+	#1036F2	151845	176527	0	9443	0	151845	false
	CPOR 8	18474766	18576331	85170487	85234486	+	#1036F2	101565	63999	9443	4845	9443	111008	false
	CPOR 8	18581176	19789350	101984750	103118083	+	#1036F2	1208174	1133333	4845	4642114	4845	1213019	false
	CPOR 12	19789350	21824042	9279	2019349	-	#9B0496	2034692	2010070	1597214		1597214	3631906	false
	CPOR 3	21824917	24383970	481888	3015569	-	#D9C001	2559053	2533681	4573006		4573006	7132059	false
	CPOR 8	24431464	25650632	96251114	97412952	+	#1036F2	1219168	1161838	4642114	-16938	4642114	5861282	false

	CPOR 8	25633694	28782003	91417833	94512262	+	#1036F2	3148309	3094429	-16938	134111	-16938	3131371	false
	CPOR 8	28916114	29029077	72613137	72725615	+	#1036F2	112963	112478	134111	243351	134111	247074	false
	CPOR 8	29272428	29341316	72984626	73107415	+	#1036F2	68888	122789	243351	1786449	243351	312239	false
	CPOR 8	31127765	36156500	79284237	84091742	+	#1036F2	5028735	4807505	1786449	145846	1786449	6815184	false
	CPOR 8	36302346	40327303	85263285	88750037	-	#1036F2	4024957	3486752	145846	-747881	145846	4170803	false
	CPOR 8	39579422	39770489	91210395	91389564	-	#1036F2	191067	179169	-747881	165287	-747881	-556814	true
	CPOR 8	39935776	40280783	91011823	91210395	-	#1036F2	345007	198572	165287	26924	165287	510294	false
	CPOR 8	40307707	40498061	97506116	97660623	-	#1036F2	190354	154507	26924	-190354	26924	217278	false
	CPOR 8	40307707	40498071	98306002	98460016	-	#1036F2	190364	154014	-190354	-46084	-190354	10	false
	CPOR 8	40451987	41216681	84495966	85157726	-	#1036F2	764694	661760	-46084		-46084	718610	false
**HGL 27**	CPOR 7	184520	4798293	25747432	30799102	+	#FF0000	4613773	5051670		13165			
	CPOR 7	4811458	5813629	30875593	31927645	+	#FF0000	1002171	1052052	13165	8351	13165	1015336	false
	CPOR 7	5821980	5900284	31907410	32014112	-	#FF0000	78304	106702	8351	5393	8351	86655	false
	CPOR 7	5905677	8815443	32016236	35215163	+	#FF0000	2909766	3198927	5393	1195	5393	2915159	false
	CPOR 7	8816638	18169718	35569121	44940792	+	#FF0000	9353080	9371671	1195	-6707528	1195	9354275	false
	CPOR 7	11462190	11581910	37305298	37549748	-	#FF0000	119720	244450	-6707528	50301	-6707528	-6587808	true
	CPOR 7	11632211	11726432	37065915	37174932	-	#FF0000	94221	109017	50301	6493685	50301	144522	false
	CPOR 7	18220117	19942210	44976649	46616275	+	#FF0000	1722093	1639626	6493685	-34720	6493685	8215778	false
	CPOR 7	19907490	21810054	46859002	48829926	+	#FF0000	1902564	1970924	-34720	-36030	-34720	1867844	false
	CPOR 7	21774024	22063104	48932324	49256425	+	#FF0000	289080	324101	-36030	36279	-36030	253050	false
	CPOR 7	22099383	22242678	49258772	49454735	-	#FF0000	143295	195963	36279	47999	36279	179574	false
	CPOR 7	22290677	22613369	51087751	51427842	+	#FF0000	322692	340091	47999	7625	47999	370691	false
	CPOR 7	22620994	27091854	51514211	55849227	+	#FF0000	4470860	4335016	7625	62798	7625	4478485	false
	CPOR 7	27154652	27373694	55850216	56052086	+	#FF0000	219042	201870	62798	-96641	62798	281840	false
	CPOR 7	27277053	27747805	56082302	56596786	+	#FF0000	470752	514484	-96641	-25301	-96641	374111	false
	CPOR 7	27722504	28174853	56609990	57115135	+	#FF0000	452349	505145	-25301	75017	-25301	427048	false
	CPOR 7	28249870	30457381	78093430	80375222	-	#FF0000	2207511	2281792	75017	69	75017	2282528	false
	CPOR 7	30457450	32398899	76085963	78037212	-	#FF0000	1941449	1951249	69	41889	69	1941518	false
	CPOR 7	32440788	33165245	75070354	75791490	-	#FF0000	724457	721136	41889	43908	41889	766346	false
	CPOR 7	33209153	33263396	74894509	74962899	+	#FF0000	54243	68390	43908	4298	43908	98151	false
	CPOR 7	33267694	33354559	74611625	74711145	-	#FF0000	86865	99520	4298	34032	4298	91163	false
	CPOR 7	33388591	33470295	74226566	74280402	-	#FF0000	81704	53836	34032	65128	34032	115736	false

	CPOR 7	33535423	33592849	74226534	74286630	-	#FF0000	57426	60096	65128	27987	65128	122554	false
	CPOR 7	33620836	33721870	74496427	74569982	-	#FF0000	101034	73555	27987	3842	27987	129021	false
	CPOR 7	33725712	33835688	74205496	74289516	+	#FF0000	109976	84020	3842	166394	3842	113818	false
	CPOR 7	34002082	34262691	73927430	74212623	-	#FF0000	260609	285193	166394	4858	166394	427003	false
	CPOR 7	34267549	36010947	71469743	73292867	-	#FF0000	1743398	1823124	4858	-69674	4858	1748256	false
	CPOR 7	35941273	36012422	71469743	71530681	+	#FF0000	71149	60938	-69674	-70895	-69674	1475	false
	CPOR 7	35941527	36107084	71394658	71486444	+	#FF0000	165557	91786	-70895	-165557	-70895	94662	false
	CPOR 7	35941527	36365261	71118558	71462914	-	#FF0000	423734	344356	-165557	-312220	-165557	258177	false
	CPOR 7	36053041	36124872	71336551	71399347	+	#FF0000	71831	62796	-312220	281417	-312220	-240389	true
	CPOR 7	36406289	36864244	70708332	71144503	-	#FF0000	457955	436171	281417	13096	281417	739372	false
	CPOR 7	36877340	37982632	69538578	70624577	-	#FF0000	1105292	1085999	13096	44639	13096	1118388	false
	CPOR 7	38027271	38167170	68869915	68939128	+	#FF0000	139899	69213	44639	19643	44639	184538	false
	CPOR 7	38186813	38419893	68406487	68687097	-	#FF0000	233080	280610	19643	119665	19643	252723	false
	CPOR 7	38539558	40231545	65987354	67853283	-	#FF0000	1691987	1865929	119665	-11349	119665	1811652	false
	CPOR 7	40220196	40814539	65226798	65964015	-	#FF0000	594343	737217	-11349	-65553	-11349	582994	false
	CPOR 7	40748986	40994426	64882624	65166843	-	#FF0000	245440	284219	-65553	15021	-65553	179887	false
	CPOR 7	41009447	41097577	64266958	64379242	-	#FF0000	88130	112284	15021	-9622	15021	103151	false
	CPOR 7	41087955	41255738	64169516	64245392	-	#FF0000	167783	75876	-9622	-33754	-9622	158161	false
	CPOR 7	41221984	41297027	63652940	63728065	+	#FF0000	75043	75125	-33754	-75043	-33754	41289	false
	CPOR 7	41221984	41298986	63491039	63580668	+	#FF0000	77002	89629	-75043	-77002	-75043	1959	false
	CPOR 7	41221984	41309201	63239925	63332076	+	#FF0000	87217	92151	-77002	46539	-77002	10215	false
	CPOR 7	41355740	41678968	61813381	62164263	-	#FF0000	323228	350882	46539	43013	46539	369767	false
	CPOR 7	41721981	41798239	60765261	60858224	-	#FF0000	76258	92963	43013	53443	43013	119271	false
	CPOR 7	41851682	42986811	58108743	59470273	-	#FF0000	1135129	1361530	53443	-49592	53443	1188572	false
	CPOR 7	42937219	43148577	57942727	58110357	-	#FF0000	211358	167630	-49592	54180	-49592	161766	false
	CPOR 7	43202757	43271856	57846290	57901131	-	#FF0000	69099	54841	54180	39278	54180	123279	false
	CPOR 7	43311134	43373371	57716462	57780350	-	#FF0000	62237	63888	39278	140292	39278	101515	false
	CPOR 7	43513663	43639108	57405152	57531690	-	#FF0000	125445	126538	140292	52939	140292	265737	false
	CPOR 7	43692047	44037548	80538817	80874076	+	#FF0000	345501	335259	52939		52939	398440	false
**HGL 28**	CPOR 29	16484	2991045	42973302	45857281	-	#007FFF	2974561	2883979		43613			
	CPOR 29	3034658	3454301	42190898	42634533	-	#007FFF	419643	443635	43613	88644	43613	463256	false
	CPOR 29	3542945	7138134	38199300	41772607	-	#007FFF	3595189	3573307	88644	-22023	88644	3683833	false

	CPOR 29	7116111	8607932	36708753	38232920	-	#007FFF	1491821	1524167	-22023	-63533	-22023	1469798	false
	CPOR 29	8544399	8682560	34499265	34572481	+	#007FFF	138161	73216	-63533	-123908	-63533	74628	false
	CPOR 29	8558652	10749253	34535785	36709921	-	#007FFF	2190601	2174136	-123908	-73683	-123908	2066693	false
	CPOR 29	10675570	10749139	36713182	36791398	+	#007FFF	73569	78216	-73683	-46595	-73683	-114	true
	CPOR 29	10702544	25091243	20638617	34378049	-	#007FFF	14388699	13739432	-46595	21042	-46595	14342104	false
	CPOR 29	25112285	27928389	17850782	20579906	-	#007FFF	2816104	2729124	21042	-6465	21042	2837146	false
	CPOR 29	27921924	37941916	8099485	17788109	-	#007FFF	10019992	9688624	-6465	518	-6465	10013527	false
	CPOR 29	37942434	40704620	5268152	8019718	-	#007FFF	2762186	2751566	518	287	518	2762704	false
	CPOR 29	40704907	40979152	4940377	5212997	-	#007FFF	274245	272620	287	89426	287	274532	false
	CPOR 29	41068578	41281098	4662805	4842162	-	#007FFF	212520	179357	89426	-94777	89426	301946	false
	CPOR 29	41186321	45852605	396271	4662376	+	#007FFF	4666284	4266105	-94777	-26234	-94777	4571507	false
	CPOR 29	45826371	45980772	196678	375777	+	#007FFF	154401	179099	-26234	75591	-26234	128167	false
	CPOR 29	46056363	46252118	29255	178576	-	#007FFF	195755	149321	75591		75591	271346	false
**HGL 29**	CPOR 11	80884	154782	24070826	24131320	-	#4C0851	73898	60494		170138			
	CPOR 11	324920	378909	25897536	25961296	-	#4C0851	53989	63760	170138	16576	170138	224127	false
	CPOR 11	395485	1077275	25088078	25682261	-	#4C0851	681790	594183	16576	14643	16576	698366	false
	CPOR 11	1091918	1169802	26138064	26212604	+	#4C0851	77884	74540	14643	137995	14643	92527	false
	CPOR 11	1307797	2384959	27083915	28041137	-	#4C0851	1077162	957222	137995	260299	137995	1215157	false
	CPOR 11	2645258	2718351	114270	249400	+	#4C0851	73093	135130	260299	92745	260299	333392	false
	CPOR 11	2811096	3103528	10687317	10936944	+	#4C0851	292432	249627	92745	-82643	92745	385177	false
	CPOR 11	3020885	3299780	10945528	11275410	+	#4C0851	278895	329882	-82643	798	-82643	196252	false
	CPOR 11	3300578	3928688	12468266	13219576	-	#4C0851	628110	751310	798	-12141	798	628908	false
	CPOR 11	3916547	4181084	12116409	12429429	-	#4C0851	264537	313020	-12141	5472	-12141	252396	false
	CPOR 11	4186556	4503802	384564	710201	+	#4C0851	317246	325637	5472	38470	5472	322718	false
	CPOR 11	4542272	5731086	2326897	3363562	+	#4C0851	1188814	1036665	38470	5323	38470	1227284	false
	CPOR 11	5736409	5938749	3436998	3653587	+	#4C0851	202340	216589	5323	47173	5323	207663	false
	CPOR 11	5985922	6163474	3987103	4174249	-	#4C0851	177552	187146	47173	39174	47173	224725	false
	CPOR 11	6202648	6468205	3682750	3878203	-	#4C0851	265557	195453	39174	47200	39174	304731	false
	CPOR 11	6515405	7895009	4174368	5199883	+	#4C0851	1379604	1025515	47200	-27419	47200	1426804	false
	CPOR 11	7867590	8380753	5233716	5595974	+	#4C0851	513163	362258	-27419	15368	-27419	485744	false
	CPOR 11	8396121	8491140	5596393	5657261	-	#4C0851	95019	60868	15368	22592	15368	110387	false
	CPOR 11	8513732	10634129	5657353	7245250	+	#4C0851	2120397	1587897	22592	164999	22592	2142989	false

	CPOR 11	10799128	11666539	7274378	7884794	+	#4C0851	867411	610416	164999	267762	164999	1032410	false
	CPOR 11	11934301	13420173	8764323	9801549	+	#4C0851	1485872	1037226	267762	682598	267762	1753634	false
	CPOR 11	14102771	14153762	13565610	13643723	-	#4C0851	50991	78113	682598	526559	682598	733589	false
	CPOR 11	14680321	14744336	1318561	1374626	-	#4C0851	64015	56065	526559		526559	590574	false
**HGL 3**	CPOR 24	1211	1233567	22279867	23459535	+	#AF0926	1232356	1179668		123739			
	CPOR 24	1357306	1914804	23461084	24033900	+	#AF0926	557498	572816	123739	-77247	123739	681237	false
	CPOR 24	1837557	2479590	24117153	24724347	+	#AF0926	642033	607194	-77247	-21094	-77247	564786	false
	CPOR 24	2458496	2580905	27443112	27518624	-	#AF0926	122409	75512	-21094	-122409	-21094	101315	false
	CPOR 24	2458496	2696053	24851231	25057302	-	#AF0926	237557	206071	-122409	-32574	-122409	115148	false
	CPOR 24	2663479	4703209	25549305	27443112	-	#AF0926	2039730	1893807	-32574	20180	-32574	2007156	false
	CPOR 24	4723389	5225091	27723025	28230428	+	#AF0926	501702	507403	20180	14304	20180	521882	false
	CPOR 24	5239395	5415647	28307967	28469938	+	#AF0926	176252	161971	14304	-9862	14304	190556	false
	CPOR 24	5405785	6399936	28569067	29500328	+	#AF0926	994151	931261	-9862	-38766	-9862	984289	false
	CPOR 24	6361170	10138464	29704411	33327480	+	#AF0926	3777294	3623069	-38766	-2742368	-38766	3738528	false
	CPOR 24	7396096	7467911	30541059	30591469	-	#AF0926	71815	50410	-2742368	2543064	-2742368	-2670553	true
	CPOR 24	10010975	10121499	33390241	33444704	+	#AF0926	110524	54463	2543064	-110305	2543064	2653588	false
	CPOR 24	10011194	10369058	33305099	33600140	+	#AF0926	357864	295041	-110305	75101	-110305	247559	false
	CPOR 24	10444159	15611287	33556536	38447014	+	#AF0926	5167128	4890478	75101	235648	75101	5242229	false
	CPOR 24	15846935	15921999	21458656	21534628	+	#AF0926	75064	75972	235648	50736	235648	310712	false
	CPOR 24	15972735	16248787	21045510	21309914	-	#AF0926	276052	264404	50736	-45040	50736	326788	false
	CPOR 24	16203747	19402663	17541938	20861269	-	#AF0926	3198916	3319331	-45040	12338	-45040	3153876	false
	CPOR 24	19415001	19839328	32107	480548	+	#AF0926	424327	448441	12338	10324	12338	436665	false
	CPOR 24	19849652	26543051	2860528	9432522	-	#AF0926	6693399	6571994	10324	2	10324	6703723	false
	CPOR 24	26543053	26611954	2769386	2860521	+	#AF0926	68901	91135	2	3	2	68903	false
	CPOR 24	26611957	27651562	1732334	2769386	-	#AF0926	1039605	1037052	3	-74351	3	1039608	false
	CPOR 24	27577211	30083724	13869605	16330459	-	#AF0926	2506513	2460854	-74351	-2079515	-74351	2432162	false
	CPOR 28	27681127	27753039	48463414	48524434	-	#60765F	71912	61020		0			
	CPOR 28	27753039	27985173	48530616	48758319	+	#60765F	232134	227703	0		0	232134	false
	CPOR 24	28004209	28665757	16349433	17011003	-	#AF0926	661548	661570	-2079515	-530655	-2079515	-1417967	true
	CPOR 24	28135102	28208405	637428	692479	-	#AF0926	73303	55051	-530655	1706623	-530655	-457352	true
	CPOR 24	29915028	30312034	11858719	12274375	-	#AF0926	397006	415656	1706623	-241553	1706623	2103629	false
	CPOR 24	30070481	30641352	13345110	13869490	-	#AF0926	570871	524380	-241553	-16081	-241553	329318	false

	CPOR 24	30625271	31633237	12275468	13340779	+	#AF0926	1007966	1065311	-16081	-264242	-16081	991885	false
	CPOR 24	31368995	31887710	11253457	11720990	-	#AF0926	518715	467533	-264242	-266940	-264242	254473	false
	CPOR 24	31620770	32537606	9953271	10819484	-	#AF0926	916836	866213	-266940	5369	-266940	649896	false
	CPOR 24	32542975	32702238	17214826	17432823	+	#AF0926	159263	217997	5369		5369	164632	false
	CPOR 7	32820649	32893550	4882234	4937682	-	#FF0000	72901	55448		5235			
	CPOR 7	32898785	33027355	4713727	4817020	-	#FF0000	128570	103293	5235	209686	5235	133805	false
	CPOR 7	33237041	35363757	20294951	22269423	-	#FF0000	2126716	1974472	209686	136928	209686	2336402	false
	CPOR 7	35500685	39590291	22284330	25480651	-	#FF0000	4089606	3196321	136928	28919	136928	4226534	false
	CPOR 7	39619210	40690468	15016332	15879700	-	#FF0000	1071258	863368	28919	2203537	28919	1100177	false
	CPOR 13	40933260	40991355	57439287	57491195	+	#11559D	58095	51908		-3413			
	CPOR 13	40987942	41154479	57024573	57178521	+	#11559D	166537	153948	-3413		-3413	163124	false
	CPOR 11	41887168	42048301	28150957	28269347	-	#4C0851	161133	118390		-136533			
	CPOR 11	41911768	42047876	24526026	24631947	-	#4C0851	136108	105921	-136533	725742	-136533	-425	true
	CPOR 11	42773618	42827756	8475476	8532284	+	#4C0851	54138	56808	725742	50203	725742	779880	false
	CPOR 11	42877959	42971248	15489070	15585167	-	#4C0851	93289	96097	50203	148253	50203	143492	false
	CPOR 7	42894005	42970033	25493900	25594887	-	#FF0000	76028	100987	2203537	166175	2203537	2279565	false
	CPOR 11	43119501	43224592	15506415	15621999	-	#4C0851	105091	115584	148253	440557	148253	253344	false
	CPOR 7	43136208	43224592	25520581	25593605	-	#FF0000	88384	73024	166175	549456	166175	254559	false
	CPOR 11	43665149	43911121	15451505	15630367	+	#4C0851	245972	178862	440557	167145	440557	686529	false
	CPOR 7	43774048	43870339	25493900	25594887	+	#FF0000	96291	100987	549456	1683587	549456	645747	false
	CPOR 11	44078266	44142617	23619441	23672977	+	#4C0851	64351	53536	167145	314903	167145	231496	false
	CPOR 11	44457520	44512605	8214511	8267746	+	#4C0851	55085	53235	314903	238253	314903	369988	false
	CPOR 11	44750858	44895904	23546595	23672982	-	#4C0851	145046	126387	238253	53583	238253	383299	false
	CPOR 11	44949487	45039275	22977461	23099431	+	#4C0851	89788	121970	53583	393418	53583	143371	false
	CPOR 11	45432693	45718237	15459592	15730299	-	#4C0851	285544	270707	393418		393418	678962	false
	CPOR 7	45553926	45622311	25493900	25595723	-	#FF0000	68385	101823	1683587	568743	1683587	1751972	false
	CPOR 7	46191054	48108167	18671581	20277431	-	#FF0000	1917113	1605850	568743	89082	568743	2485856	false
	CPOR 7	48197249	48295155	18555777	18635068	+	#FF0000	97906	79291	89082	232	89082	186988	false
	CPOR 7	48295387	51078215	16097683	18555708	-	#FF0000	2782828	2458025	232	374	232	2783060	false
	CPOR 7	51078589	51277924	15929015	16036366	-	#FF0000	199335	107351	374	176490	374	199709	false
	CPOR 7	51454414	51826214	14675222	15008383	-	#FF0000	371800	333161	176490	3168	176490	548290	false
	CPOR 7	51829382	51922716	14549960	14624783	-	#FF0000	93334	74823	3168	1003	3168	96502	false

	CPOR 7	51923719	52198514	14206613	14483478	-	#FF0000	274795	276865	1003	26750	1003	275798	false
	CPOR 7	52225264	57174241	5380924	9730460	-	#FF0000	4948977	4349536	26750	465367	26750	4975727	false
	CPOR 15	57185400	57236391	51604871	51655925	+	#564208	50991	51054		61129			
	CPOR 15	57297520	57451635	51966479	52116547	+	#564208	154115	150068	61129	614	61129	215244	false
	CPOR 15	57452249	57537237	51324236	51425086	+	#564208	84988	100850	614	-84734	614	85602	false
	CPOR 15	57452503	57532762	50759465	50847816	+	#564208	80259	88351	-84734	5046	-84734	-4475	true
	CPOR 15	57537808	57636556	51486695	51601492	+	#564208	98748	114797	5046	5762051	5046	103794	false
	CPOR 7	57639608	63384581	0	5380924	-	#FF0000	5744973	5380924	465367		465367	6210340	false
	CPOR 15	63398607	63581104	400660	623158	+	#564208	182497	222498	5762051	-12453	5762051	5944548	false
	CPOR 15	63568651	63623816	636650	693221	+	#564208	55165	56571	-12453	7751	-12453	42712	false
	CPOR 15	63631567	67358570	750588	4370044	+	#564208	3727003	3619456	7751	59955	7751	3734754	false
	CPOR 15	67418525	77640006	4387726	13598050	+	#564208	10221481	9210324	59955	620	59955	10281436	false
	CPOR 15	77640626	77710805	13782889	13841200	-	#564208	70179	58311	620	70049	620	70799	false
	CPOR 15	77780854	77989015	13608357	13779948	-	#564208	208161	171591	70049	15833	70049	278210	false
	CPOR 15	78004848	78233017	13854201	14047837	+	#564208	228169	193636	15833	264836	15833	244002	false
	CPOR 15	78497853	84610209	14261781	19684656	+	#564208	6112356	5422875	264836	17081	264836	6377192	false
	CPOR 15	84627290	84802726	19614333	19803737	-	#564208	175436	189404	17081	-93853	17081	192517	false
	CPOR 15	84708873	84805326	19808809	19863785	-	#564208	96453	54976	-93853	4052	-93853	2600	false
	CPOR 15	84809378	87741556	19815260	22694902	+	#564208	2932178	2879642	4052	13194	4052	2936230	false
	CPOR 15	87754750	87945647	22833252	23051403	+	#564208	190897	218151	13194	-90229	13194	204091	false
	CPOR 15	87855418	87945647	23207976	23278272	-	#564208	90229	70296	-90229	-27138	-90229	0	true
	CPOR 15	87918509	91231184	23323002	26278981	+	#564208	3312675	2955979	-27138	10	-27138	3285537	false
	CPOR 15	91231194	98890985	26501175	33381766	+	#564208	7659791	6880591	10	119799	10	7659801	false
	CPOR 15	99010784	99294533	33623528	33913516	+	#564208	283749	289988	119799	10045	119799	403548	false
	CPOR 15	99304578	104655135	40994853	45784425	-	#564208	5350557	4789572	10045	77902	10045	5360602	false
	CPOR 15	104733037	107024935	38800519	40953857	-	#564208	2291898	2153338	77902	49524	77902	2369800	false
	CPOR 15	107074459	108426329	37469977	38795624	+	#564208	1351870	1325647	49524	44287	49524	1401394	false
	CPOR 15	108470616	108535644	51507982	51594983	+	#564208	65028	87001	44287	67292	44287	109315	false
	CPOR 15	108602936	112354637	34001602	37459235	-	#564208	3751701	3457633	67292	4698	67292	3818993	false
	CPOR 15	112359335	121734720	51422199	60319526	-	#564208	9375385	8897327	4698	25362	4698	9380083	false
	CPOR 15	121760082	121887196	50605624	50690730	+	#564208	127114	85106	25362	-85612	25362	152476	false
	CPOR 15	121801584	121904214	51208038	51279152	-	#564208	102630	71114	-85612	-20517	-85612	17018	false

	CPOR 15	121883697	122485594	50040901	50595607	-	#564208	601897	554706	-20517	956	-20517	581380	false
	CPOR 15	122486550	122865166	49618234	49980292	-	#564208	378616	362058	956	54112	956	379572	false
	CPOR 15	122919278	126422690	46176264	49618141	-	#564208	3503412	3441877	54112	-26260	54112	3557524	false
	CPOR 15	126396430	126799605	45867128	46167851	-	#564208	403175	300723	-26260	2818953	-26260	376915	false
	CPOR 26	126666438	128803191	5273465	7342453	+	#C9FE31	2136753	2068988	14148031	77156	14148031	16284784	false
	CPOR 26	128880347	133922172	229598	5248612	-	#C9FE31	5041825	5019014	77156	-17311	77156	5118981	false
	CPOR 15	129618558	129688900	60388474	60451056	-	#564208	70342	62582	2818953		2818953	2889295	false
	CPOR 26	133904861	135225928	7650246	8969992	+	#C9FE31	1321067	1319746	-17311	21	-17311	1303756	false
	CPOR 26	135225949	135280462	8979973	9037161	-	#C9FE31	54513	57188	21		21	54534	false
**HGL 4**	CPOR 20	106400	282578	1477132	1540225	+	#69E283	176178	63093		-151244			
	CPOR 20	131334	238005	1499089	1563360	+	#69E283	106671	64271	-151244	67746	-151244	-44573	true
	CPOR 20	305751	360832	894097	1002514	-	#69E283	55081	108417	67746	113	67746	122827	false
	CPOR 20	360945	412620	345277	398986	+	#69E283	51675	53709	113	65686	113	51788	false
	CPOR 20	478306	534182	250588	340777	-	#69E283	55876	90189	65686	26186	65686	121562	false
	CPOR 20	560368	867502	2020528	2281889	+	#69E283	307134	261361	26186	-47592	26186	333320	false
	CPOR 20	819910	952293	2277119	2351818	-	#69E283	132383	74699	-47592	6293	-47592	84791	false
	CPOR 20	958586	1018759	2361037	2419337	+	#69E283	60173	58300	6293	3104	6293	66466	false
	CPOR 20	1021863	1363217	2532272	2837756	+	#69E283	341354	305484	3104	10468	3104	344458	false
	CPOR 20	1373685	3596852	3742881	5913843	+	#69E283	2223167	2170962	10468	-50847	10468	2233635	false
	CPOR 20	3546005	24953617	6176886	26644573	+	#69E283	21407612	20467687	-50847	-446482	-50847	21356765	false
	CPOR 20	24507135	24577938	26216328	26273133	+	#69E283	70803	56805	-446482	-53882	-446482	-375679	true
	CPOR 20	24524056	24606524	26232235	26300578	-	#69E283	82468	68343	-53882	302394	-53882	28586	false
	CPOR 20	24908918	24968963	27010581	27072921	+	#69E283	60045	62340	302394	145307	302394	362439	false
	CPOR 20	25114270	44714024	27073324	44803084	+	#69E283	19599754	17729760	145307	54516	145307	19745061	false
	CPOR 20	44768540	44959540	44803102	44980901	-	#69E283	191000	177799	54516	1843	54516	245516	false
	CPOR 20	44961383	45373385	44989767	45341506	+	#69E283	412002	351739	1843	37121	1843	413845	false
	CPOR 20	45410506	45492415	45477575	45564231	-	#69E283	81909	86656	37121	33765	37121	119030	false
	CPOR 20	45526180	59663446	45624174	58609093	+	#69E283	14137266	12984919	33765	57491	33765	14171031	false
	CPOR 20	59720937	61096182	58612798	59977223	+	#69E283	1375245	1364425	57491	-74048	57491	1432736	false
	CPOR 20	61022134	61107245	60140256	60192413	+	#69E283	85111	52157	-74048	-73619	-74048	11063	false
	CPOR 20	61033626	61209447	60063227	60176773	+	#69E283	175821	113546	-73619	-62000	-73619	102202	false
	CPOR 20	61147447	62352894	60064185	61053147	+	#69E283	1205447	988962	-62000	17601	-62000	1143447	false

	CPOR X	61687713	61808224	91703752	91781893	-	#01FC33	120511	78141		86601			
	CPOR X	61894825	62352894	91406589	91703752	-	#01FC33	458069	297163	86601		86601	544670	false
	CPOR 20	62370495	62470071	61244611	61321917	+	#69E283	99576	77306	17601	10733	17601	117177	false
	CPOR 20	62480804	62693960	61070230	61244611	-	#69E283	213156	174381	10733	4165	10733	223889	false
	CPOR 20	62698125	62956996	61355999	61549160	+	#69E283	258871	193161	4165	8393	4165	263036	false
	CPOR 20	62965389	63021150	61549160	61628790	-	#69E283	55761	79630	8393	14020	8393	64154	false
	CPOR 20	63035170	66239990	61628790	64556561	+	#69E283	3204820	2927771	14020	58	14020	3218840	false
	CPOR 20	66240048	70846679	64696534	68959858	+	#69E283	4606631	4263324	58		58	4606689	false
	CPOR 12	70863440	82815515	13102427	24390191	-	#9B0496	11952075	11287764		13741			
	CPOR 12	82829256	82949946	12953508	13102427	+	#9B0496	120690	148919	13741	18168	13741	134431	false
	CPOR 12	82968114	83443676	12472333	12947118	-	#9B0496	475562	474785	18168	0	18168	493730	false
	CPOR 12	83443676	83712694	12196437	12472327	+	#9B0496	269018	275890	0	0	0	269018	false
	CPOR 12	83712694	84227996	11616764	12196430	-	#9B0496	515302	579666	0	1624	0	515302	false
	CPOR 12	84229620	88486472	7377223	11559706	-	#9B0496	4256852	4182483	1624	40620	1624	4258476	false
	CPOR 12	88527092	98356253	28314100	38089967	-	#9B0496	9829161	9775867	40620	370	40620	9869781	false
	CPOR 12	98356623	98420297	28151234	28241729	-	#9B0496	63674	90495	370	2007	370	64044	false
	CPOR 12	98422304	98530822	28153206	28244506	-	#9B0496	108518	91300	2007	-106055	2007	110525	false
	CPOR 12	98424767	99807948	26637679	28028100	-	#9B0496	1383181	1390421	-106055	52473	-106055	1277126	false
	CPOR 12	99860421	102034657	24467582	26619652	-	#9B0496	2174236	2152070	52473		52473	2226709	false
**HGL 5**	CPOR 9	160511	2224222	622879	2642131	+	#BF726D	2063711	2019252		-72381			
	CPOR 9	2151841	5856607	2808917	6261932	+	#BF726D	3704766	3453015	-72381	-2099	-72381	3632385	false
	CPOR 9	5854508	11118028	6290784	11530941	+	#BF726D	5263520	5240157	-2099	-4268357	-2099	5261421	false
	CPOR 9	6849671	6912892	7220418	7281990	+	#BF726D	63221	61572	-4268357	4020585	-4268357	-4205136	true
	CPOR 9	10933477	11067377	12144425	12198959	-	#BF726D	133900	54534	4020585	-133900	4020585	4154485	false
	CPOR 9	10933477	11101346	12047560	12124935	+	#BF726D	167869	77375	-133900	-167869	-133900	33969	false
	CPOR 9	10933477	11178331	11823643	11952055	-	#BF726D	244854	128412	-167869	-105309	-167869	76985	false
	CPOR 9	11073022	37218802	12126642	37045753	+	#BF726D	26145780	24919111	-105309	-26108429	-105309	26040471	false
	CPOR 9	11110373	11178854	11874525	11936188	-	#BF726D	68481	61663	-26108429	-65432	-26108429	-26039948	true
	CPOR 9	11113422	11178854	12010566	12069841	-	#BF726D	65432	59275	-65432	26091714	-65432	0	true
	CPOR 9	37270568	40714713	37089463	40244965	+	#BF726D	3444145	3155502	26091714	1183	26091714	29535859	false
	CPOR 9	40715896	42805753	40302792	42377458	+	#BF726D	2089857	2074666	1183		1183	2091040	false
	CPOR 16	43484719	43756470	16945116	17177797	+	#81A8F8	271751	232681		35072			

	CPOR 16	43791542	47618543	17276350	20673278	+	#81A8F8	3827001	3396928	35072	32	35072	3862073	false
	CPOR 16	47618575	49408760	20735505	22286915	+	#81A8F8	1790185	1551410	32	11025	32	1790217	false
	CPOR 16	49419785	50279310	22392129	23069087	+	#81A8F8	859525	676958	11025	-24057	11025	870550	false
	CPOR 16	50255253	50435562	23076995	23228214	-	#81A8F8	180309	151219	-24057	-180309	-24057	156252	false
	CPOR 16	50255253	54999490	23288735	27050105	+	#81A8F8	4744237	3761370	-180309	51138	-180309	4563928	false
	CPOR 16	55050628	55117723	27062115	27172404	+	#81A8F8	67095	110289	51138	-67095	51138	118233	false
	CPOR 16	55050628	55965592	27193238	28091983	+	#81A8F8	914964	898745	-67095	34079	-67095	847869	false
	CPOR 16	55999671	56339424	16590523	16919291	-	#81A8F8	339753	328768	34079	-7969	34079	373832	false
	CPOR 16	56331455	56429859	16501790	16566913	+	#81A8F8	98404	65123	-7969	-11426	-7969	90435	false
	CPOR 16	56418433	56575347	16358560	16482824	+	#81A8F8	156914	124264	-11426	-156914	-11426	145488	false
	CPOR 16	56418433	56927790	15879413	16381924	-	#81A8F8	509357	502511	-156914	20654	-156914	352443	false
	CPOR 16	56948444	56999400	28939461	29014833	+	#81A8F8	50956	75372	20654	111652	20654	71610	false
	CPOR 16	57111052	57289924	29603072	29720825	+	#81A8F8	178872	117753	111652	-144495	111652	290524	false
	CPOR 16	57145429	57212544	29845711	29897106	+	#81A8F8	67115	51395	-144495	-67115	-144495	-77380	true
	CPOR 16	57145429	57270194	29680187	29785120	+	#81A8F8	124765	104933	-67115	153166	-67115	57650	false
	CPOR 31	57289983	57365641	5177194	5239661	-	#DB00F9	75658	62467		412222			
	CPOR 16	57423360	57483709	30028351	30096302	+	#81A8F8	60349	67951	153166	47302	153166	213515	false
	CPOR 16	57531011	57595051	29939005	29999378	+	#81A8F8	64040	60373	47302	-64039	47302	111342	false
	CPOR 16	57531012	57604967	29616605	29720825	+	#81A8F8	73955	104220	-64039	284229	-64039	9916	false
	CPOR 31	57777863	57845688	5172874	5239716	-	#DB00F9	67825	66842	412222	383906	412222	480047	false
	CPOR 16	57889196	58029916	29949024	30106722	+	#81A8F8	140720	157698	284229	-51012	284229	424949	false
	CPOR 16	57978904	58033786	30050769	30117851	-	#81A8F8	54882	67082	-51012	445506	-51012	3870	false
	CPOR 31	58229594	58303031	5177194	5239661	+	#DB00F9	73437	62467	383906		383906	457343	false
	CPOR 16	58479292	58678902	30252082	30321568	+	#81A8F8	199610	69486	445506	95112	445506	645116	false
	CPOR 16	58774014	59031640	31071994	31193235	-	#81A8F8	257626	121241	95112	-84100	95112	352738	false
	CPOR 16	58947540	59031640	31464208	31530234	-	#81A8F8	84100	66026	-84100	81407	-84100	0	true
	CPOR 16	59113047	65456817	32014157	37661983	+	#81A8F8	6343770	5647826	81407	-5657127	81407	6425177	false
	CPOR 16	59799690	59864902	32659769	32752031	-	#81A8F8	65212	92262	-5657127	5643318	-5657127	-5591915	true
	CPOR 16	65508220	74721307	37698190	45540831	+	#81A8F8	9213087	7842641	5643318	52083	5643318	14856405	false
	CPOR 16	74773390	82247296	45583721	52380258	+	#81A8F8	7473906	6796537	52083	85202	52083	7525989	false
	CPOR 16	82332498	85481808	52428930	55484301	+	#81A8F8	3149310	3055371	85202	75973	85202	3234512	false
	CPOR 16	85557781	89252965	55484301	59079113	+	#81A8F8	3695184	3594812	75973	223	75973	3771157	false

	CPOR 16	89253188	89314666	59081006	59145532	-	#81A8F8	61478	64526	223	20	223	61701	false
	CPOR 16	89314686	92125297	59145532	62008067	+	#81A8F8	2810611	2862535	20	158511	20	2810631	false
	CPOR 16	92283808	92829925	62003829	62564633	+	#81A8F8	546117	560804	158511		158511	704628	false
	CPOR 1	92830201	98918447	196814789	202583274	+	#A6F1FC	6088246	5768485	7295655	60968	7295655	13383901	false
	CPOR 1	98979415	109903081	202585559	212890697	+	#A6F1FC	10923666	10305138	60968	158	60968	10984634	false
	CPOR 1	109903239	109994552	212896735	212973647	-	#A6F1FC	91313	76912	158	2535	158	91471	false
	CPOR 1	109997087	112343888	212985719	215196011	+	#A6F1FC	2346801	2210292	2535	-1793657	2535	2349336	false
	CPOR 1	110550231	112294441	215198714	216755282	+	#A6F1FC	1744210	1556568	-1793657	-646572	-1793657	-49447	true
	CPOR 1	111647869	112273692	216764121	217305240	-	#A6F1FC	625823	541119	-646572	12655	-646572	-20749	true
	CPOR 1	112286347	120503927	218591039	226882127	+	#A6F1FC	8217580	8291088	12655	76313	12655	8230235	false
	CPOR 1	120580240	127447574	226882137	233464054	+	#A6F1FC	6867334	6581917	76313		76313	6943647	false
**HGL 6**	CPOR 13	53270	2962397	15007539	17742328	-	#11559D	2909127	2734789		59169			
	CPOR 13	3021566	7562574	10589846	14975432	-	#11559D	4541008	4385586	59169	-79303	59169	4600177	false
	CPOR 13	7483271	16359987	2019948	10618512	-	#11559D	8876716	8598564	-79303	54826	-79303	8797413	false
	CPOR 13	16414813	18375906	8821	1988855	-	#11559D	1961093	1980034	54826		54826	2015919	false
	CPOR 11	18375945	29298700	57927822	68288498	+	#4C0851	10922755	10360676		-30599			
	CPOR 11	29268101	29334338	68371935	68430248	-	#4C0851	66237	58313	-30599	-64020	-30599	35638	false
	CPOR 11	29270318	29346013	68288499	68359769	-	#4C0851	75695	71270	-64020	4	-64020	11675	false
	CPOR 11	29346017	33304229	68444469	72247757	+	#4C0851	3958212	3803288	4		4	3958216	false
	CPOR 6	33780949	37784365	7582570	11399342	+	#7F00FF	4003416	3816772		59241			
	CPOR 6	37843606	41598797	11451090	14609913	+	#7F00FF	3755191	3158823	59241	69	59241	3814432	false
	CPOR 6	41598866	41706270	14612818	14703087	-	#7F00FF	107404	90269	69	2181	69	107473	false
	CPOR 6	41708451	49452302	14703087	21908987	+	#7F00FF	7743851	7205900	2181	-29414	2181	7746032	false
	CPOR 6	49422888	49507953	22019814	22075404	+	#7F00FF	85065	55590	-29414	-74395	-29414	55651	false
	CPOR 6	49433558	49527579	21919646	21999032	-	#7F00FF	94021	79386	-74395	-14366	-74395	19626	false
	CPOR 6	49513213	49606243	22145943	22265396	+	#7F00FF	93030	119453	-14366	82055	-14366	78664	false
	CPOR 6	49688298	51736434	22242716	24068584	+	#7F00FF	2048136	1825868	82055	-5680	82055	2130191	false
	CPOR 6	51730754	55704986	24652445	28151950	+	#7F00FF	3974232	3499505	-5680	23039	-5680	3968552	false
	CPOR 6	55728025	70509260	28140016	41627552	+	#7F00FF	14781235	13487536	23039	51223	23039	14804274	false
	CPOR 6	70560483	74236542	41685571	45271456	+	#7F00FF	3676059	3585885	51223	128168	51223	3727282	false
	CPOR 6	74364710	75933585	45468180	47101818	+	#7F00FF	1568875	1633638	128168	127495	128168	1697043	false
	CPOR 6	76061080	76587950	47184372	47693513	+	#7F00FF	526870	509141	127495	87681	127495	654365	false

	CPOR 6	76675631	76732894	47698522	47763402	+	#7F00FF	57263	64880	87681	1866184	87681	144944	false
	CPOR 2	76936750	78593218	32680360	34122398	+	#7FFF00	1656468	1442038					
	CPOR 6	78599078	78865801	315556	568704	+	#7F00FF	266723	253148	1866184	63678	1866184	2132907	false
	CPOR 6	78929479	79054510	574190	658571	-	#7F00FF	125031	84381	63678	84439	63678	188709	false
	CPOR 6	79138949	82080936	660314	3199632	+	#7F00FF	2941987	2539318	84439	54119	84439	3026426	false
	CPOR 6	82135055	82323026	3211105	3380093	+	#7F00FF	187971	168988	54119	58553	54119	242090	false
	CPOR 6	82381579	84540727	3381923	5296173	+	#7F00FF	2159148	1914250	58553	52119	58553	2217701	false
	CPOR 6	84592846	86378941	5613944	7089514	-	#7F00FF	1786095	1475570	52119	170230	52119	1838214	false
	CPOR 6	86549171	86929475	5302771	5613944	-	#7F00FF	380304	311173	170230		170230	550534	false
	CPOR 22	87282749	87539703	4698813	4964490	+	#007F00	256954	265677		69687			
	CPOR 22	87609390	88797449	4964490	6104822	+	#007F00	1188059	1140332	69687	1693	69687	1257746	false
	CPOR 22	88799142	88852698	6643868	6710162	-	#007F00	53556	66294	1693	564	1693	55249	false
	CPOR 22	88853262	89593589	6183405	6863375	+	#007F00	740327	679970	564	70412	564	740891	false
	CPOR 22	89664001	90053070	6971335	7356584	-	#007F00	389069	385249	70412	13584	70412	459481	false
	CPOR 22	90066654	90546222	7362643	7818934	+	#007F00	479568	456291	13584	9874	13584	493152	false
	CPOR 22	90556096	90665896	7818934	7908214	-	#007F00	109800	89280	9874	4695	9874	119674	false
	CPOR 22	90670591	90794167	11843605	11981309	+	#007F00	123576	137704	4695	33515	4695	128271	false
	CPOR 22	90827682	91675542	19238136	20017854	-	#007F00	847860	779718	33515	76533	33515	881375	false
	CPOR 22	91752075	92268788	18695501	19237578	-	#007F00	516713	542077	76533	20772	76533	593246	false
	CPOR 22	92289560	93005856	17585457	18307956	+	#007F00	716296	722499	20772	5290	20772	737068	false
	CPOR 22	93011146	93133145	8678382	8778673	-	#007F00	121999	100291	5290	-120885	5290	127289	false
	CPOR 22	93012260	93145138	18584372	18640496	-	#007F00	132878	56124	-120885	-2560	-120885	11993	false
	CPOR 22	93142578	93221522	8535903	8616936	+	#007F00	78944	81033	-2560	-78944	-2560	76384	false
	CPOR 22	93142578	93649799	8884886	9399818	+	#007F00	507221	514932	-78944	-507162	-78944	428277	false
	CPOR 22	93142637	93204928	18554059	18607920	+	#007F00	62291	53861	-507162	-62250	-507162	-444871	true
	CPOR 22	93142678	93204934	18521318	18607926	+	#007F00	62256	86608	-62250	442878	-62250	6	false
	CPOR 22	93647812	93703748	17479099	17531272	+	#007F00	55936	52173	442878	-35335	442878	498814	false
	CPOR 22	93668413	95990731	9484791	11798581	+	#007F00	2322318	2313790	-35335	-134518	-35335	2286983	false
	CPOR 22	95856213	95994097	4370078	4440720	-	#007F00	137884	70642	-134518	-914	-134518	3366	false
	CPOR 22	95993183	101089990	12241542	17403874	+	#007F00	5096807	5162332	-914	-2723483	-914	5095893	false
	CPOR 22	98366507	98451149	12103917	12200135	-	#007F00	84642	96218	-2723483	2639171	-2723483	-2638841	true
	CPOR 22	101090320	103210437	20222239	22281942	+	#007F00	2120117	2059703	2639171	-76082	2639171	4759288	false

	CPOR 22	103134355	103224621	8309841	8421187	-	#007F00	90266	111346	-76082	84305	-76082	14184	false
	CPOR 22	103308926	104929248	22504226	24440822	-	#007F00	1620322	1936596	84305	894	84305	1704627	false
	CPOR 22	104930142	105129292	24540373	24796914	+	#007F00	199150	256541	894	25132	894	200044	false
	CPOR 22	105154424	105228490	25027465	25120606	+	#007F00	74066	93141	25132	2510	25132	99198	false
	CPOR 9	105172084	105228490	42429597	42518050	+	#BF726D	56406	88453		2510			
	CPOR 22	105231000	105291485	26345809	26440086	+	#007F00	60485	94277	2510		2510	62995	false
	CPOR 9	105231000	105293070	42612282	42698551	+	#BF726D	62070	86269	2510		2510	64580	false
**HGL 7**	CPOR 3	21547	335793	111979175	112335634	-	#D9C001	314246	356459		4			
	CPOR 3	335797	3299754	109250884	111928801	-	#D9C001	2963957	2677917	4	-16396	4	2963961	false
	CPOR 3	3283358	5668860	107095601	109262571	+	#D9C001	2385502	2166970	-16396	-42549	-16396	2369106	false
	CPOR 3	5626311	10005725	102944139	107196308	-	#D9C001	4379414	4252169	-42549	-265055	-42549	4336865	false
	CPOR 3	9740670	10364834	101952929	102538937	+	#D9C001	624164	586008	-265055	-624164	-265055	359109	false
	CPOR 3	9740670	10390467	100351698	100933917	+	#D9C001	649797	582219	-624164	-272376	-624164	25633	false
	CPOR 3	10118091	10389922	101101017	101373644	+	#D9C001	271831	272627	-272376	-25985	-272376	-545	true
	CPOR 3	10363937	11134220	99647304	100349082	-	#D9C001	770283	701778	-25985	2383	-25985	744298	false
	CPOR 3	11136603	11740811	98898511	99587245	-	#D9C001	604208	688734	2383	-41211	2383	606591	false
	CPOR 3	11699600	23539408	87427697	98899128	-	#D9C001	11839808	11471431	-41211	-2093434	-41211	11798597	false
	CPOR 3	21445974	23788051	83588957	85684683	+	#D9C001	2342077	2095726	-2093434	-392749	-2093434	248643	false
	CPOR 3	23395302	24109839	80876159	81596501	-	#D9C001	714537	720342	-392749	-267313	-392749	321788	false
	CPOR 3	23842526	24458776	82978406	83588957	+	#D9C001	616250	610551	-267313	-310810	-267313	348937	false
	CPOR 3	24147966	30245060	74907012	80823651	-	#D9C001	6097094	5916639	-310810	-38659	-310810	5786284	false
	CPOR 3	30206401	30353758	74769489	74863512	-	#D9C001	147357	94023	-38659	-120542	-38659	108698	false
	CPOR 3	30233216	30353758	74575396	74678647	-	#D9C001	120542	103251	-120542	-120542	-120542	0	true
	CPOR 3	30233216	30399461	74525260	74613978	-	#D9C001	166245	88718	-120542	-3417	-120542	45703	false
	CPOR 3	30396044	43017579	48189355	60266580	+	#D9C001	12621535	12077225	-3417	69881	-3417	12618118	false
	CPOR 3	43087460	44474356	60454216	61827707	+	#D9C001	1386896	1373491	69881	109497	69881	1456777	false
	CPOR 3	44583853	44637301	62966465	63081285	+	#D9C001	53448	114820	109497	-12057	109497	162945	false
	CPOR 3	44625244	44732433	63321615	63477361	+	#D9C001	107189	155746	-12057	-50603	-12057	95132	false
	CPOR 3	44681830	44742518	63000993	63076786	+	#D9C001	60688	75793	-50603	-9741	-50603	10085	false
	CPOR 3	44732777	45494384	63316846	63905264	+	#D9C001	761607	588418	-9741	-109748	-9741	751866	false
	CPOR 3	45384636	55355362	63871594	73037175	+	#D9C001	9970726	9165581	-109748	73357	-109748	9860978	false
	CPOR 3	55428719	56358806	73092209	73922764	+	#D9C001	930087	830555	73357	20947	73357	1003444	false

	CPOR 3	56379753	56749025	73999183	74312644	-	#D9C001	369272	313461	20947		20947	390219	false
	CPOR 25	56820696	61825209	37958802	42647389	-	#49FEF6	5004513	4688587		-25813			
	CPOR 25	61799396	61894671	37764794	37840693	+	#49FEF6	95275	75899	-25813	-95275	-25813	69462	false
	CPOR 25	61799396	74970514	25019525	37624104	-	#49FEF6	13171118	12604579	-95275	-13133536	-95275	13075843	false
	CPOR 25	61836978	61894402	37678341	37735332	+	#49FEF6	57424	56991	-13133536	13076165	-13133536	-13076112	true
	CPOR 25	74970567	77252718	22702871	24947132	-	#49FEF6	2282151	2244261	13076165	5	13076165	15358316	false
	CPOR 25	77252723	79713231	20294726	22619957	-	#49FEF6	2460508	2325231	5	6585	5	2460513	false
	CPOR 25	79719816	81243637	18709675	20227691	-	#49FEF6	1523821	1518016	6585	-677	6585	1530406	false
	CPOR 25	81242960	81358774	18317388	18444037	-	#49FEF6	115814	126649	-677	12	-677	115137	false
	CPOR 25	81358786	82141039	15134859	15916029	+	#49FEF6	782253	781170	12	-80469	12	782265	false
	CPOR 25	82060570	82655070	15926317	16519371	+	#49FEF6	594500	593054	-80469	-7917	-80469	514031	false
	CPOR 25	82647153	82710097	22628507	22695834	-	#49FEF6	62944	67327	-7917	-62944	-7917	55027	false
	CPOR 25	82647153	82738711	18451422	18544940	-	#49FEF6	91558	93518	-62944	-1549	-62944	28614	false
	CPOR 25	82737162	82813955	16548246	16616433	-	#49FEF6	76793	68187	-1549	-53529	-1549	75244	false
	CPOR 25	82760426	83090321	16616796	17015943	+	#49FEF6	329895	399147	-53529	205569	-53529	276366	false
	CPOR 25	83295890	84364726	17096684	18117857	+	#49FEF6	1068836	1021173	205569	590	205569	1274405	false
	CPOR 25	84365316	85903985	13481325	15082206	-	#49FEF6	1538669	1600881	590	178725	590	1539259	false
	CPOR 25	86082710	87066802	11980734	12967627	-	#49FEF6	984092	986893	178725	67286	178725	1162817	false
	CPOR 25	87134088	87226888	11693576	11783434	+	#49FEF6	92800	89858	67286	-35563	67286	160086	false
	CPOR 25	87191325	91504078	7227135	11601136	-	#49FEF6	4312753	4374001	-35563	24437	-35563	4277190	false
	CPOR 25	91528515	93211161	5325966	7085700	-	#49FEF6	1682646	1759734	24437	19940	24437	1707083	false
	CPOR 25	93231101	97668547	524448	5267659	-	#49FEF6	4437446	4743211	19940	414458	19940	4457386	false
	CPOR 25	98083005	98146440	43269564	43337091	-	#49FEF6	63435	67527	414458	9001	414458	477893	false
	CPOR 25	98155441	98361970	43022983	43157520	+	#49FEF6	206529	134537	9001	-203916	9001	215530	false
	CPOR 25	98158054	98290999	43118079	43260958	+	#49FEF6	132945	142879	-203916	-132945	-203916	-70971	true
	CPOR 25	98158054	98361970	42982276	43075973	+	#49FEF6	203916	93697	-132945	-203916	-132945	70971	false
	CPOR 25	98158054	98362608	42705963	42855626	+	#49FEF6	204554	149663	-203916	-104048	-203916	638	false
	CPOR 25	98258560	98362608	43331020	43391932	+	#49FEF6	104048	60912	-104048	159507	-104048	0	true
	CPOR 25	98522115	98609557	43545522	43673090	+	#49FEF6	87442	127568	159507	31035	159507	246949	false
	CPOR 25	98640592	98710245	44154390	44227591	+	#49FEF6	69653	73201	31035	-20755	31035	100688	false
	CPOR 25	98689490	106804146	44473307	52303456	+	#49FEF6	8114656	7830149	-20755		-20755	8093901	false
**HGL 8**	CPOR 22	10448	6595891	54154562	59846039	-	#007F00	6585443	5691477		-30829			

	CPOR 22	6565062	6620939	53684256	53776033	+	#007F00	55877	91777	-30829	25120	-30829	25048	false
	CPOR 22	6646059	9523318	50667954	53346445	-	#007F00	2877259	2678491	25120	-5334	25120	2902379	false
	CPOR 22	9517984	11482057	48840207	50626403	-	#007F00	1964073	1786196	-5334	199268	-5334	1958739	false
	CPOR 22	11681325	15279874	44802215	48277873	-	#007F00	3598549	3475658	199268	2575	199268	3797817	false
	CPOR 22	15282449	17244682	42722723	44663963	-	#007F00	1962233	1941240	2575	100836	2575	1964808	false
	CPOR 22	17345518	17517083	42558480	42713091	-	#007F00	171565	154611	100836	-169806	100836	272401	false
	CPOR 22	17347277	18606766	41106709	42274749	-	#007F00	1259489	1168040	-169806	-1259264	-169806	1089683	false
	CPOR 22	17347502	17535086	42338964	42509617	-	#007F00	187584	170653	-1259264	1076335	-1259264	-1071680	true
	CPOR 22	18611421	18716779	40975788	41090358	+	#007F00	105358	114570	1076335	18858	1076335	1181693	false
	CPOR 22	18735637	23753970	36187840	40963979	-	#007F00	5018333	4776139	18858	28719	18858	5037191	false
	CPOR 22	23782689	26331805	33455645	36122066	-	#007F00	2549116	2666421	28719	-43526	28719	2577835	false
	CPOR 22	26288279	26358442	33363008	33453381	-	#007F00	70163	90373	-43526	9676	-43526	26637	false
	CPOR 22	26368118	26432122	32954440	33076790	-	#007F00	64004	122350	9676	51329	9676	73680	false
	CPOR 22	26483451	26556401	33238476	33338708	+	#007F00	72950	100232	51329	-2726	51329	124279	false
	CPOR 22	26553675	26643331	33268724	33322833	+	#007F00	89656	54109	-2726	-37983	-2726	86930	false
	CPOR 22	26605348	26656123	32595262	32665879	-	#007F00	50775	70617	-37983	-2751	-37983	12792	false
	CPOR 22	26653372	26733162	32535115	32587264	+	#007F00	79790	52149	-2751	-34769	-2751	77039	false
	CPOR 22	26698393	26778157	32487108	32539972	-	#007F00	79764	52864	-34769	-7897	-34769	44995	false
	CPOR 22	26770260	27067622	32119421	32465043	-	#007F00	297362	345622	-7897	36612	-7897	289465	false
	CPOR 22	27104234	27269766	31712842	32029561	-	#007F00	165532	316719	36612	-100046	36612	202144	false
	CPOR 22	27169720	27514320	30958960	31345924	-	#007F00	344600	386964	-100046	-52354	-100046	244554	false
	CPOR 22	27461966	27517582	30870500	30945142	-	#007F00	55616	74642	-52354	353633	-52354	3262	false
	CPOR 22	27871215	28094106	30525929	30754056	-	#007F00	222891	228127	353633	-26652	353633	576524	false
	CPOR 22	28067454	28218092	30288916	30496157	+	#007F00	150638	207241	-26652	166	-26652	123986	false
	CPOR 22	28218258	28306427	30065794	30125594	-	#007F00	88169	59800	166	-86318	166	88335	false
	CPOR 22	28220109	28464356	29442986	29797385	-	#007F00	244247	354399	-86318	531	-86318	157929	false
	CPOR 22	28464887	28758909	28920860	29380303	-	#007F00	294022	459443	531	22160	531	294553	false
	CPOR 22	28781069	29050206	28515273	28846759	-	#007F00	269137	331486	22160	-18833	22160	291297	false
	CPOR 22	29031373	29176983	28253008	28366871	-	#007F00	145610	113863	-18833	31871	-18833	126777	false
	CPOR 22	29208854	29333729	27445936	27624094	+	#007F00	124875	178158	31871	-123147	31871	156746	false
	CPOR 22	29210582	29315614	27896704	27963225	+	#007F00	105032	66521	-123147	-81667	-123147	-18115	true
	CPOR 22	29233947	29333724	27717680	27865349	+	#007F00	99777	147669	-81667	9188	-81667	18110	false

	CPOR 22	29342912	30068981	56954	909205	+	#007F00	726069	852251	9188	6	9188	735257	false
	CPOR 22	30068987	32170530	1913664	4214281	-	#007F00	2101543	2300617	6	8957	6	2101549	false
	CPOR 22	32179487	32958140	972376	1858032	-	#007F00	778653	885656	8957	2427	8957	787610	false
	CPOR 22	32960567	33035695	4588157	4657851	+	#007F00	75128	69694	2427	38506649	2427	77555	false
	CPOR 5	33798859	41453642	62919518	69764854	+	#09FC8D	7654783	6845336		55384			
	CPOR 5	41509026	43449392	69800776	71586203	+	#09FC8D	1940366	1785427	55384	33490	55384	1995750	false
	CPOR 5	43482882	47297901	75677548	79006181	-	#09FC8D	3815019	3328633	33490	-51381	33490	3848509	false
	CPOR 5	47246520	47441129	74579690	74740463	+	#09FC8D	194609	160773	-51381	-96629	-51381	143228	false
	CPOR 5	47344500	47473237	73776115	73927243	-	#09FC8D	128737	151128	-96629	-57055	-96629	32108	false
	CPOR 5	47416182	47473237	73559789	73626173	-	#09FC8D	57055	66384	-57055	-20536	-57055	0	true
	CPOR 5	47452701	47665621	74643452	74802559	+	#09FC8D	212920	159107	-20536	-189167	-20536	192384	false
	CPOR 5	47476454	47665621	73762685	73925788	-	#09FC8D	189167	163103	-189167	-115435	-189167	0	true
	CPOR 5	47550186	47665621	75001551	75052002	+	#09FC8D	115435	50451	-115435	-77537	-115435	0	true
	CPOR 5	47588084	48047427	75340145	75601962	+	#09FC8D	459343	261817	-77537	-314798	-77537	381806	false
	CPOR 5	47732629	48017571	74724247	74984951	+	#09FC8D	284942	260704	-314798	-152963	-314798	-29856	true
	CPOR 5	47864608	48017571	73466026	73577203	-	#09FC8D	152963	111177	-152963	-152963	-152963	0	true
	CPOR 5	47864608	48047427	75016664	75245038	+	#09FC8D	182819	228374	-152963	-64366	-152963	29856	false
	CPOR 5	47983061	48111458	73638606	73700490	+	#09FC8D	128397	61884	-64366	-7297	-64366	64031	false
	CPOR 5	48104161	48163826	74880002	74980789	+	#09FC8D	59665	100787	-7297	-26027	-7297	52368	false
	CPOR 5	48137799	48206970	74150583	74202494	-	#09FC8D	69171	51911	-26027	-69155	-26027	43144	false
	CPOR 5	48137815	48206964	75533723	75588355	+	#09FC8D	69149	54632	-69155	-61184	-69155	-6	true
	CPOR 5	48145780	48206970	75196069	75281187	+	#09FC8D	61190	85118	-61184	-53381	-61184	6	false
	CPOR 5	48153589	48270711	73638609	73727721	-	#09FC8D	117122	89112	-53381	-65920	-53381	63741	false
	CPOR 5	48204791	48361848	73640084	73713384	+	#09FC8D	157057	73300	-65920	-123785	-65920	91137	false
	CPOR 5	48238063	48427577	74854229	74963281	+	#09FC8D	189514	109052	-123785	-160025	-123785	65729	false
	CPOR 5	48267552	48617250	73055633	73262949	-	#09FC8D	349698	207316	-160025	110753	-160025	189673	false
	CPOR 5	48728003	48778610	72928760	72985343	+	#09FC8D	50607	56583	110753	-3596	110753	161360	false
	CPOR 5	48775014	49867478	71852273	72885145	-	#09FC8D	1092464	1032872	-3596	4222	-3596	1088868	false
	CPOR 5	49871700	55716609	79067334	84521568	+	#09FC8D	5844909	5454234	4222	-99060	4222	5849131	false
	CPOR 5	55617549	55756560	84538431	84670173	-	#09FC8D	139011	131742	-99060	84	-99060	39951	false
	CPOR 5	55756644	63421931	84686338	91323700	+	#09FC8D	7665287	6637362	84	2244	84	7665371	false
	CPOR 5	63424175	64348172	61569781	62281512	+	#09FC8D	923997	711731	2244	256	2244	926241	false

	CPOR 5	64348428	64549197	62365974	62610940	-	#09FC8D	200769	244966	256	0	256	201025	false
	CPOR 5	64549197	65769798	60357854	61542438	-	#09FC8D	1220601	1184584	0	-787	0	1220601	false
	CPOR 5	65769011	71974544	54619881	60284241	-	#09FC8D	6205533	5664360	-787	15	-787	6204746	false
	CPOR 5	71974559	72186163	54458947	54619881	+	#09FC8D	211604	160934	15	1	15	211619	false
	CPOR 5	72186164	75625157	51571290	54458947	-	#09FC8D	3438993	2887657	1	37549	1	3438994	false
	CPOR 5	75662706	80368720	46761150	51205999	-	#09FC8D	4706014	4444849	37549	54577	37549	4743563	false
	CPOR 5	80423297	84446214	42960289	46722066	-	#09FC8D	4022917	3761777	54577	24933	54577	4077494	false
	CPOR 5	84471147	87208501	40449038	42905529	-	#09FC8D	2737354	2456491	24933	0	24933	2762287	false
	CPOR 5	87208501	87350334	40263728	40394428	-	#09FC8D	141833	130700	0	106454	0	141833	false
	CPOR 5	87456788	93121220	34989906	40263619	-	#09FC8D	5664432	5273713	106454	-47875	106454	5770886	false
	CPOR 5	93073345	93129979	33887398	33952032	-	#09FC8D	56634	64634	-47875	8263	-47875	8759	false
	CPOR 5	93138242	93209149	34810860	34867439	+	#09FC8D	70907	56579	8263	-30226	8263	79170	false
	CPOR 5	93178923	93319018	34452376	34559334	+	#09FC8D	140095	106958	-30226	-140095	-30226	109869	false
	CPOR 5	93178923	94429804	32585908	33741428	-	#09FC8D	1250881	1155520	-140095	-6952	-140095	1110786	false
	CPOR 5	94422852	97403939	29370531	32275074	-	#09FC8D	2981087	2904543	-6952	97303	-6952	2974135	false
	CPOR 5	97501242	98575047	28262902	29362086	-	#09FC8D	1073805	1099184	97303	2720	97303	1171108	false
	CPOR 5	98577767	99022782	27667623	28156391	-	#09FC8D	445015	488768	2720	-132040	2720	447735	false
	CPOR 5	98890742	99169346	27025029	27286421	+	#09FC8D	278604	261392	-132040	-153083	-132040	146564	false
	CPOR 5	99016263	99087524	27530154	27640416	+	#09FC8D	71261	110262	-153083	-12119	-153083	-81822	true
	CPOR 5	99075405	99145235	26020420	26092819	-	#09FC8D	69830	72399	-12119	-66246	-12119	57711	false
	CPOR 5	99078989	99268453	24559679	24650007	+	#09FC8D	189464	90328	-66246	-82838	-66246	123218	false
	CPOR 5	99185615	99268453	27348315	27406513	+	#09FC8D	82838	58198	-82838	-7099	-82838	0	true
	CPOR 5	99261354	99329364	25014136	25085939	-	#09FC8D	68010	71803	-7099	-68010	-7099	60911	false
	CPOR 5	99261354	99488956	25521234	25739187	-	#09FC8D	227602	217953	-68010	-227330	-68010	159592	false
	CPOR 5	99261626	99634916	26237720	26544310	+	#09FC8D	373290	306590	-227330	-372296	-227330	145960	false
	CPOR 5	99262620	99337937	25228648	25304021	-	#09FC8D	75317	75373	-372296	-49923	-372296	-296979	true
	CPOR 5	99288014	99351680	26912415	27012774	+	#09FC8D	63666	100359	-49923	-36549	-49923	13743	false
	CPOR 5	99315131	99475572	24407207	24486619	+	#09FC8D	160441	79412	-36549	-155061	-36549	123892	false
	CPOR 5	99320511	99621400	26618627	26823033	+	#09FC8D	300889	204406	-155061	-45222	-155061	145828	false
	CPOR 5	99576178	100008151	23550860	24010302	-	#09FC8D	431973	459442	-45222	26478	-45222	386751	false
	CPOR 5	100034629	100228920	22478184	22654509	-	#09FC8D	194291	176325	26478	-194291	26478	220769	false
	CPOR 5	100034629	100364698	23008704	23305812	-	#09FC8D	330069	297108	-194291	-201644	-194291	135778	false

	CPOR 5	100163054	100269375	22809420	22877560	-	#09FC8D	106321	68140	-201644	-96219	-201644	-95323	true
	CPOR 5	100173156	100447354	22070974	22423675	-	#09FC8D	274198	352701	-96219	-32653	-96219	177979	false
	CPOR 5	100414701	101321141	21181294	22058499	-	#09FC8D	906440	877205	-32653	-60205	-32653	873787	false
	CPOR 5	101260936	101436897	20994533	21160710	+	#09FC8D	175961	166177	-60205	4037	-60205	115756	false
	CPOR 5	101440934	101675556	20522285	20701623	-	#09FC8D	234622	179338	4037	-92719	4037	238659	false
	CPOR 5	101582837	101741461	20256917	20474365	+	#09FC8D	158624	217448	-92719	-145924	-92719	65905	false
	CPOR 5	101595537	102580210	19363821	20232894	-	#09FC8D	984673	869073	-145924	-148815	-145924	838749	false
	CPOR 5	102431395	102768252	18980903	19272134	-	#09FC8D	336857	291231	-148815	-324033	-148815	188042	false
	CPOR 5	102444219	102586828	19274767	19334303	-	#09FC8D	142609	59536	-324033	246786	-324033	-181424	true
	CPOR 5	102833614	104078146	17777196	18973732	-	#09FC8D	1244532	1196536	246786	8336144	246786	1491318	false
	CPOR 4	104078146	111642137	96437258	103695223	-	#26B63B	7563991	7257965		-34990			
	CPOR 4	111607147	112413322	95587122	96341110	-	#26B63B	806175	753988	-34990	11429990	-34990	771185	false
	CPOR 5	112414290	113086009	49797	751757	+	#09FC8D	671719	701960	8336144	89280	8336144	9007863	false
	CPOR 5	113175289	114799613	934346	2467263	+	#09FC8D	1624324	1532917	89280	-260839	89280	1713604	false
	CPOR 5	114538774	114801228	4749161	4987532	-	#09FC8D	262454	238371	-260839	-262454	-260839	1615	false
	CPOR 5	114538774	114808750	2917667	3162965	+	#09FC8D	269976	245298	-262454	50910	-262454	7522	false
	CPOR 5	114859660	114980237	5661379	5809223	+	#09FC8D	120577	147844	50910	-120577	50910	171487	false
	CPOR 5	114859660	115105005	5137482	5336235	-	#09FC8D	245345	198753	-120577	-245345	-120577	124768	false
	CPOR 5	114859660	115195772	3179589	3526792	+	#09FC8D	336112	347203	-245345	-336112	-245345	90767	false
	CPOR 5	114859660	115293260	2490019	2914354	+	#09FC8D	433600	424335	-336112	-433600	-336112	97488	false
	CPOR 5	114859660	115293489	4261527	4730820	-	#09FC8D	433829	469293	-433600	-362442	-433600	229	false
	CPOR 5	114931047	115455141	5828558	6347908	+	#09FC8D	524094	519350	-362442	-253679	-362442	161652	false
	CPOR 5	115201462	115293260	4991231	5046535	-	#09FC8D	91798	55304	-253679	5416	-253679	-161881	true
	CPOR 5	115298676	117391960	6463817	8545387	+	#09FC8D	2093284	2081570	5416	4399	5416	2098700	false
	CPOR 5	117396359	117466322	8553432	8627443	-	#09FC8D	69963	74011	4399	-20237	4399	74362	false
	CPOR 5	117446085	118412511	8564579	9582810	+	#09FC8D	966426	1018231	-20237	-725130	-20237	946189	false
	CPOR 5	117687381	118737617	9582810	10638946	-	#09FC8D	1050236	1056136	-725130	-1050236	-725130	325106	false
	CPOR 5	117687381	123857878	11209272	17157488	+	#09FC8D	6170497	5948216	-1050236		-1050236	5120261	false
	CPOR 4	123843312	124925466	103759772	104768829	+	#26B63B	1082154	1009057	11429990		11429990	12512144	false
**HGL 9**	CPOR 6	23557	3988579	78912862	82737163	+	#7F00FF	3965022	3824301		-48788			
	CPOR 6	3939791	3991893	82846905	83008368	+	#7F00FF	52102	161463	-48788	-10225	-48788	3314	false
	CPOR 6	3981668	4130175	83081905	83316954	+	#7F00FF	148507	235049	-10225	1868	-10225	138282	false

	CPOR 6	4132043	15565291	83370228	94489667	+	#7F00FF	11433248	11119439	1868	-9383937	1868	11435116	false
	CPOR 6	6181354	6287588	84999261	85138030	-	#7F00FF	106234	138769	-9383937	9288417	-9383937	-9277703	true
	CPOR 6	15576005	19578468	94756768	98618828	+	#7F00FF	4002463	3862060	9288417	14026748	9288417	13290880	false
	CPOR 11	20037811	20133642	24556142	24631947	+	#4C0851	95831	75805		72015			
	CPOR 11	20205657	20289357	13900235	13954391	-	#4C0851	83700	54156	72015	3856479	72015	155715	false
	CPOR 2	20452633	20568406	29457541	29521906	+	#7FFF00	115773	64365		28204			
	CPOR 2	20596610	20806083	9397772	9534495	-	#7FFF00	209473	136723	28204	513300	28204	237677	false
	CPOR 2	21319383	21405062	9467200	9522253	-	#7FFF00	85679	55053	513300	99102	513300	598979	false
	CPOR 2	21504164	21600366	9619696	9697887	+	#7FFF00	96202	78191	99102	285353	99102	195304	false
	CPOR 2	21885719	21944567	29661922	29771238	+	#7FFF00	58848	109316	285353	-44638	285353	344201	false
	CPOR 2	21899929	21974500	29667890	29760072	-	#7FFF00	74571	92182	-44638	-11226	-44638	29933	false
	CPOR 2	21963274	22087808	29663777	29743352	+	#7FFF00	124534	79575	-11226	-106169	-11226	113308	false
	CPOR 2	21981639	22097502	9756557	9879172	-	#7FFF00	115863	122615	-106169	6099	-106169	9694	false
	CPOR 2	22103601	22169512	29607978	29662335	-	#7FFF00	65911	54357	6099	60355	6099	72010	false
	CPOR 2	22229867	22290177	9397539	9463294	+	#7FFF00	60310	65755	60355	83891	60355	120665	false
	CPOR 2	22374068	22434131	31400179	31459002	-	#7FFF00	60063	58823	83891	-9932	83891	143954	false
	CPOR 2	22424199	22482184	30755847	30813547	+	#7FFF00	57985	57700	-9932	-57985	-9932	48053	false
	CPOR 2	22424199	22501430	32400199	32457829	+	#7FFF00	77231	57630	-57985	875680	-57985	19246	false
	CPOR 2	23377110	23473178	29579117	29640420	+	#7FFF00	96068	61303	875680	238440	875680	971748	false
	CPOR 2	23711618	23787383	29135139	29222362	-	#7FFF00	75765	87223	238440	115816	238440	314205	false
	CPOR 2	23903199	23991762	9783477	9859408	-	#7FFF00	88563	75931	115816	-82240	115816	204379	false
	CPOR 2	23909522	24000542	9755719	9809042	-	#7FFF00	91020	53323	-82240	526480	-82240	8780	false
	CPOR 11	24145836	24300055	24526026	24617343	-	#4C0851	154219	91317	3856479	9102	3856479	4010698	false
	CPOR 11	24309157	24424668	8664354	8741926	+	#4C0851	115511	77572	9102	-100024	9102	124613	false
	CPOR 11	24324644	24463836	14554544	14686234	-	#4C0851	139192	131690	-100024	1360747	-100024	39168	false
	CPOR 2	24527022	24595164	30709376	30759556	-	#7FFF00	68142	50180	526480	-68142	526480	594622	false
	CPOR 2	24527022	24696501	32296917	32403818	-	#7FFF00	169479	106901	-68142	-107752	-68142	101337	false
	CPOR 2	24588749	24696501	31960960	32029250	+	#7FFF00	107752	68290	-107752	91321	-107752	0	true
	CPOR 2	24787822	24862832	9397539	9463294	-	#7FFF00	75010	65755	91321	222274	91321	166331	false
	CPOR 2	25085106	25299519	9755719	9912476	+	#7FFF00	214413	156757	222274	268734	222274	436687	false
	CPOR 2	25568253	25661943	9469112	9529118	+	#7FFF00	93690	60006	268734	1357891	268734	362424	false
	CPOR 11	25824583	25917637	13900235	13954391	+	#4C0851	93054	54156	1360747	644706	1360747	1453801	false

	CPOR 21	25848001	25911854	21489249	21549241	+	#7444DF	63853	59992	5572490	2435371	5572490	5636343	false
	CPOR 11	26562343	26662825	56291027	56363295	+	#4C0851	100482	72268	644706	233296	644706	745188	false
	CPOR 11	26896121	26957976	14420404	14486606	+	#4C0851	61855	66202	233296	1819620	233296	295151	false
	CPOR 2	27019834	27087685	32234971	32291007	-	#7FFF00	67851	56036	1357891	-33833	1357891	1425742	false
	CPOR 2	27053852	27116406	31676348	31730480	-	#7FFF00	62554	54132	-33833	-19924	-33833	28721	false
	CPOR 2	27096482	27153529	30504818	30571773	-	#7FFF00	57047	66955	-19924	8697	-19924	37123	false
	CPOR 2	27162226	27225115	32124968	32186351	+	#7FFF00	62889	61383	8697	188090	8697	71586	false
	CPOR 2	27413205	27476588	32399817	32453790	-	#7FFF00	63383	53973	188090	5697418	188090	251473	false
	CPOR 21	28347225	28414678	10896157	10964770	-	#7444DF	67453	68613	2435371		2435371	2502824	false
	CPOR 11	28777596	28832882	13565610	13643769	-	#4C0851	55286	78159	1819620	2073429	1819620	1874906	false
	CPOR 9	30195664	30265034	444443	503674	+	#BF726D	69370	59231	5671845	1854755	5671845	5741215	false
	CPOR 11	30906311	30966829	1318561	1374626	+	#4C0851	60518	56065	2073429	2027193	2073429	2133947	false
	CPOR 9	32119789	32180697	438552	507092	-	#BF726D	60908	68540	1854755	4098663	1854755	1915663	false
	CPOR 11	32994022	33058223	8004985	8065664	+	#4C0851	64201	60679	2027193		2027193	2091394	false
	CPOR 6	33605216	36297624	76423930	78910965	-	#7F00FF	2692408	2487035	14026748	-49543	14026748	16719156	false
	CPOR 6	36248081	36381031	76316968	76406339	+	#7F00FF	132950	89371	-49543	-123011	-49543	83407	false
	CPOR 6	36258020	36495647	76009797	76231140	-	#7F00FF	237627	221343	-123011	-38562	-123011	114616	false
	CPOR 6	36457085	40388951	72287564	75999719	-	#7F00FF	3931866	3712155	-38562	0	-38562	3893304	false
	CPOR 6	40388951	41337550	71218693	72165261	-	#7F00FF	948599	946568	0	40730	0	948599	false
	CPOR 6	41378280	44029154	68469380	70979858	-	#7F00FF	2650874	2510478	40730	-69062	40730	2691604	false
	CPOR 6	43960092	44034771	68422260	68473668	+	#7F00FF	74679	51408	-69062	-41705	-69062	5617	false
	CPOR 6	43993066	44135561	68236768	68291424	+	#7F00FF	142495	54656	-41705	-73913	-41705	100790	false
	CPOR 6	44061648	44138803	68687642	68738353	+	#7F00FF	77155	50711	-73913	-60813	-73913	3242	false
	CPOR 6	44077990	44163335	68534698	68602476	-	#7F00FF	85345	67778	-60813	-17580	-60813	24532	false
	CPOR 6	44145755	44805012	67601908	68233462	-	#7F00FF	659257	631554	-17580	-53301	-17580	641677	false
	CPOR 6	44751711	44805253	67255970	67312097	-	#7F00FF	53542	56127	-53301	142721	-53301	241	false
	CPOR 6	44947974	65066256	48423401	66281817	+	#7F00FF	20118282	17858416	142721	117	142721	20261003	false
	CPOR 6	65066373	65157349	91873216	91960313	+	#7F00FF	90976	87097	117	8884	117	91093	false
	CPOR 6	65166233	65231633	66289814	66383437	+	#7F00FF	65400	93623	8884	121401	8884	74284	false
	CPOR 6	65353034	66266865	66435669	67175838	+	#7F00FF	913831	740169	121401		121401	1035232	false
	CPOR 4	66342245	66732423	724175	1088968	+	#26B63B	390178	364793	39584464	54824	39584464	39974642	false
	CPOR 4	66787247	67475606	1116095	1702777	+	#26B63B	688359	586682	54824	113933	54824	743183	false

	CPOR 4	67589539	88736298	5212370	25641953	-	#26B63B	21146759	20429583	113933	-3298	113933	21260692	false
	CPOR 4	88733000	91888718	1713965	4740711	-	#26B63B	3155718	3026746	-3298		-3298	3152420	false
	CPOR 24	91905920	92124237	39660311	39956466	+	#AF0926	218317	296155		47234			
	CPOR 24	92171471	92259369	39322579	39435160	-	#AF0926	87898	112581	47234	2514	47234	135132	false
	CPOR 24	92261883	93006516	40450487	41260883	-	#AF0926	744633	810396	2514	916	2514	747147	false
	CPOR 24	93007432	93176203	38905373	39125319	+	#AF0926	168771	219946	916	48640	916	169687	false
	CPOR 24	93224843	93742403	39071676	39459193	+	#AF0926	517560	387517	48640	-382090	48640	566200	false
	CPOR 24	93360313	93529319	41845565	41955234	+	#AF0926	169006	109669	-382090	4423	-382090	-213084	true
	CPOR 24	93533742	93742470	41921383	42030438	+	#AF0926	208728	109055	4423		4423	213151	false
**HGL X**	CPOR X	1125418	1236271	152223440	152291774	+	#01FC33	110853	68334		760389			
	CPOR X	1996660	2179912	152031809	152089444	-	#01FC33	183252	57635	760389	74153	760389	943641	false
	CPOR X	2254065	2697006	151763302	152030698	-	#01FC33	442941	267396	74153	13041	74153	517094	false
	CPOR X	2710047	2804414	151383105	151446496	+	#01FC33	94367	63391	13041	6261	13041	107408	false
	CPOR X	2810675	2985311	151362752	151728633	-	#01FC33	174636	365881	6261	-12139	6261	180897	false
	CPOR X	2973172	5620862	148609060	151403426	-	#01FC33	2647690	2794366	-12139	-127481	-12139	2635551	false
	CPOR 8	4730108	4978092	103363013	103616692	+	#1036F2	247984	253679		514897			
	CPOR 8	5492989	5579667	103656343	103782101	+	#1036F2	86678	125758	514897		514897	601575	false
	CPOR X	5493381	6430354	146940549	147925241	+	#01FC33	936973	984692	-127481	102202	-127481	809492	false
	CPOR X	6532556	6608924	145191442	145259965	+	#01FC33	76368	68523	102202	-48087	102202	178570	false
	CPOR X	6560837	7524595	145386082	146435248	+	#01FC33	963758	1049166	-48087	104194	-48087	915671	false
	CPOR X	7628789	12880758	138029529	143181214	-	#01FC33	5251969	5151685	104194	22231	104194	5356163	false
	CPOR X	12902989	13608078	137203793	137962004	-	#01FC33	705089	758211	22231	-59963	22231	727320	false
	CPOR X	13548115	21643626	129532429	137217629	-	#01FC33	8095511	7685200	-59963	8019	-59963	8035548	false
	CPOR X	21651645	21742165	129456550	129532428	+	#01FC33	90520	75878	8019	11873	8019	98539	false
	CPOR X	21754038	21873501	129385571	129455155	+	#01FC33	119463	69584	11873	23265	11873	131336	false
	CPOR X	21896766	24490949	126576907	129225028	-	#01FC33	2594183	2648121	23265	-32555	23265	2617448	false
	CPOR X	24458394	24510951	126183733	126235834	+	#01FC33	52557	52101	-32555	-52557	-32555	20002	false
	CPOR X	24458394	24517419	125753249	125807922	-	#01FC33	59025	54673	-52557	-59025	-52557	6468	false
	CPOR X	24458394	24533092	125871330	125944427	-	#01FC33	74698	73097	-59025	-74698	-59025	15673	false
	CPOR X	24458394	24533908	126439215	126506180	-	#01FC33	75514	66965	-74698	-75514	-74698	816	false
	CPOR X	24458394	24534047	125780064	125851942	-	#01FC33	75653	71878	-75514	-75653	-75514	139	false
	CPOR X	24458394	28481185	121369974	125687835	-	#01FC33	4022791	4317861	-75653	-41696	-75653	3947138	false

	CPOR X	28439489	28587719	121261077	121383644	-	#01FC33	148230	122567	-41696	-124052	-41696	106534	false
	CPOR X	28463667	28588015	121317303	121432985	+	#01FC33	124348	115682	-124052	-34383	-124052	296	false
	CPOR X	28553632	28611762	121185619	121321054	+	#01FC33	58130	135435	-34383	-5977	-34383	23747	false
	CPOR X	28605785	32264105	117446658	121077240	-	#01FC33	3658320	3630582	-5977	535	-5977	3652343	false
	CPOR X	32264640	32970166	116513806	117396088	-	#01FC33	705526	882282	535	4	535	706061	false
	CPOR X	32970170	33127173	115507882	115636348	-	#01FC33	157003	128466	4	78834	4	157007	false
	CPOR X	33206007	34621409	113863964	115413253	-	#01FC33	1415402	1549289	78834	-974128	78834	1494236	false
	CPOR X	33647281	33764448	112780926	112851951	+	#01FC33	117167	71025	-974128	872393	-974128	-856961	true
	CPOR X	34636841	34767345	113708332	113845480	+	#01FC33	130504	137148	872393	10849	872393	1002897	false
	CPOR X	34778194	36214378	112157681	113700066	-	#01FC33	1436184	1542385	10849	-351445	10849	1447033	false
	CPOR X	35862933	35969919	114995265	115116639	+	#01FC33	106986	121374	-351445	326341	-351445	-244459	true
	CPOR X	36296260	42883494	105387340	111979066	-	#01FC33	6587234	6591726	326341	57289	326341	6913575	false
	CPOR X	42940783	44599689	103776338	105369951	-	#01FC33	1658906	1593613	57289	143061	57289	1716195	false
	CPOR X	44742750	44798257	103595644	103666664	-	#01FC33	55507	71020	143061	63695	143061	198568	false
	CPOR X	44861952	44949641	103437842	103531936	-	#01FC33	87689	94094	63695	145306	63695	151384	false
	CPOR X	45094947	47080829	101342526	103144139	-	#01FC33	1985882	1801613	145306	356293	145306	2131188	false
	CPOR X	47437122	47822862	100658026	101081517	-	#01FC33	385740	423491	356293	31700	356293	742033	false
	CPOR X	47854562	47928176	100509565	100602901	-	#01FC33	73614	93336	31700	108397	31700	105314	false
	CPOR X	48036573	49085865	99372281	100506889	-	#01FC33	1049292	1134608	108397	27819	108397	1157689	false
	CPOR X	49113684	49544534	98964132	99311830	-	#01FC33	430850	347698	27819	75581	27819	458669	false
	CPOR X	49620115	50094421	98394244	98963957	-	#01FC33	474306	569713	75581	35661	75581	549887	false
	CPOR X	50130082	50791087	97681956	98332034	-	#01FC33	661005	650078	35661	21271	35661	696666	false
	CPOR X	50812358	51311212	97322724	97593884	-	#01FC33	498854	271160	21271	134640	21271	520125	false
	CPOR X	51445852	51530452	97155345	97240138	-	#01FC33	84600	84793	134640	81563	134640	219240	false
	CPOR X	51612015	51868283	96804337	97012636	-	#01FC33	256268	208299	81563	117533	81563	337831	false
	CPOR X	51985816	52041722	96540609	96641172	+	#01FC33	55906	100563	117533	150790	117533	173439	false
	CPOR X	52192512	52350577	96232739	96472229	+	#01FC33	158065	239490	150790	-83283	150790	308855	false
	CPOR X	52267294	53053010	95539639	96350960	-	#01FC33	785716	811321	-83283	439979	-83283	702433	false
	CPOR X	53492989	53792801	94871030	95089781	-	#01FC33	299812	218751	439979	-5558	439979	739791	false
	CPOR X	53787243	53838434	94803004	94869089	+	#01FC33	51191	66085	-5558	0	-5558	45633	false
	CPOR X	53838434	54248481	94346293	94802974	-	#01FC33	410047	456681	0	150639	0	410047	false
	CPOR X	54399120	54553415	94039605	94220167	-	#01FC33	154295	180562	150639	228707	150639	304934	false

	CPOR X	54782122	54903334	93746406	93919268	-	#01FC33	121212	172862	228707	146479	228707	349919	false
	CPOR X	55049813	55235972	93430681	93605962	-	#01FC33	186159	175281	146479	270797	146479	332638	false
	CPOR X	55506769	55579162	93176626	93238117	-	#01FC33	72393	61491	270797	311414	270797	343190	false
	CPOR X	55890576	56022938	92938536	93082381	-	#01FC33	132362	143845	311414	300176	311414	443776	false
	CPOR X	56323114	56383846	92682700	92740862	-	#01FC33	60732	58162	300176	307167	300176	360908	false
	CPOR X	56691013	56759197	92472712	92545407	-	#01FC33	68184	72695	307167	717056	307167	375351	false
	CPOR X	57476253	57604862	92026123	92143631	+	#01FC33	128609	117508	717056	347853	717056	845665	false
	CPOR X	57952715	58267528	86416855	86655616	+	#01FC33	314813	238761	347853	205880	347853	662666	false
	CPOR X	58473408	58587234	86856728	86957500	-	#01FC33	113826	100772	205880	241090	205880	319706	false
	CPOR X	58828324	58913013	87105731	87173543	+	#01FC33	84689	67812	241090	63063	241090	325779	false
	CPOR X	58976076	59123523	87205757	87353344	+	#01FC33	147447	147587	63063	259927	63063	210510	false
	CPOR X	59383450	59783395	87544540	88085487	+	#01FC33	399945	540947	259927	97541	259927	659872	false
	CPOR X	59880936	60245791	88143859	88522086	+	#01FC33	364855	378227	97541	86857	97541	462396	false
	CPOR X	60332648	60408084	88535680	88631654	+	#01FC33	75436	95974	86857	67302	86857	162293	false
	CPOR X	60475386	60759653	88824899	89078673	+	#01FC33	284267	253774	67302	59449	67302	351569	false
	CPOR X	60819102	60936926	89149393	89265713	-	#01FC33	117824	116320	59449	38736	59449	177273	false
	CPOR X	60975662	61074573	89333339	89391820	+	#01FC33	98911	58481	38736	63813	38736	137647	false
	CPOR X	61138386	62242272	89413850	90475102	+	#01FC33	1103886	1061252	63813	193862	63813	1167699	false
	CPOR X	62436134	62548185	90547020	90650795	+	#01FC33	112051	103775	193862	75952	193862	305913	false
	CPOR X	62624137	63258693	90697104	91225543	+	#01FC33	634556	528439	75952	339201	75952	710508	false
	CPOR X	63597894	65040805	84401197	86204875	-	#01FC33	1442911	1803678	339201	60305	339201	1782112	false
	CPOR X	65101110	65597370	83869601	84347529	-	#01FC33	496260	477928	60305	363193	60305	556565	false
	CPOR X	65960563	66175385	83485333	83656702	-	#01FC33	214822	171369	363193	213312	363193	578015	false
	CPOR X	66388697	68128468	81255238	82964641	-	#01FC33	1739771	1709403	213312	152823	213312	1953083	false
	CPOR X	68281291	68485616	80917965	81116942	-	#01FC33	204325	198977	152823	229189	152823	357148	false
	CPOR X	68714805	68854716	80601662	80725645	-	#01FC33	139911	123983	229189	429990	229189	369100	false
	CPOR X	69284706	69354784	80007776	80081686	-	#01FC33	70078	73910	429990	194923	429990	500068	false
	CPOR X	69549707	69757993	79674095	79848773	-	#01FC33	208286	174678	194923	253189	194923	403209	false
	CPOR X	70011182	70267260	79312771	79640134	-	#01FC33	256078	327363	253189	42820	253189	509267	false
	CPOR X	70310080	70449129	79072100	79206558	-	#01FC33	139049	134458	42820	140688	42820	181869	false
	CPOR X	70589817	70688512	78714867	78854819	-	#01FC33	98695	139952	140688	19301	140688	239383	false
	CPOR X	70707813	71107463	78214042	78641101	-	#01FC33	399650	427059	19301	172638	19301	418951	false

	CPOR X	71280101	71421404	77667276	77790041	-	#01FC33	141303	122765	172638	64997	172638	313941	false
	CPOR X	71486401	71551865	77505588	77612919	-	#01FC33	65464	107331	64997	946641	64997	130461	false
	CPOR X	72498506	72663330	76494813	76708827	-	#01FC33	164824	214014	946641	24148	946641	1111465	false
	CPOR X	72687478	72745968	76328003	76388711	-	#01FC33	58490	60708	24148	357700	24148	82638	false
	CPOR X	73103668	73885763	75253721	76008164	-	#01FC33	782095	754443	357700	115740	357700	1139795	false
	CPOR X	74001503	74130598	74988504	75153921	-	#01FC33	129095	165417	115740	548297	115740	244835	false
	CPOR X	74678895	74794237	74453653	74556082	-	#01FC33	115342	102429	548297	25233	548297	663639	false
	CPOR X	74819470	74967207	74209864	74391177	-	#01FC33	147737	181313	25233	925485	25233	172970	false
	CPOR X	75892692	75989847	74026077	74152071	-	#01FC33	97155	125994	925485	151441	925485	1022640	false
	CPOR X	76141288	76438865	73652174	73884494	-	#01FC33	297577	232320	151441	300156	151441	449018	false
	CPOR X	76739021	76902657	73255164	73399827	-	#01FC33	163636	144663	300156	1055513	300156	463792	false
	CPOR X	77958170	78070843	72837103	72991492	-	#01FC33	112673	154389	1055513	427789	1055513	1168186	false
	CPOR X	78498632	78609910	72412630	72497152	-	#01FC33	111278	84522	427789	109909	427789	539067	false
	CPOR X	78719819	79059637	72025904	72392638	-	#01FC33	339818	366734	109909	309413	109909	449727	false
	CPOR X	79369050	79590017	71490610	71763287	-	#01FC33	220967	272677	309413	110088	309413	530380	false
	CPOR X	79700105	80073627	71225174	71461182	-	#01FC33	373522	236008	110088	161633	110088	483610	false
	CPOR X	80235260	81162900	70383289	71155031	-	#01FC33	927640	771742	161633	183265	161633	1089273	false
	CPOR X	81346165	81414997	70169388	70259947	-	#01FC33	68832	90559	183265	77017	183265	252097	false
	CPOR X	81492014	81627225	70077469	70168236	-	#01FC33	135211	90767	77017	713733	77017	212228	false
	CPOR X	82340958	82407018	69894265	70074235	-	#01FC33	66060	179970	713733	608628	713733	779793	false
	CPOR X	83015646	83162671	69725654	69861349	-	#01FC33	147025	135695	608628	73825	608628	755653	false
	CPOR X	83236496	83384139	69529965	69724685	-	#01FC33	147643	194720	73825	992667	73825	221468	false
	CPOR X	84376806	84712463	68459504	68839069	+	#01FC33	335657	379565	992667	225796	992667	1328324	false
	CPOR X	84938259	85109480	68990495	69144575	+	#01FC33	171221	154080	225796	149551	225796	397017	false
	CPOR X	85259031	85329524	69156987	69250622	+	#01FC33	70493	93635	149551	175169	149551	220044	false
	CPOR X	85504693	85598447	69288906	69347988	+	#01FC33	93754	59082	175169	2708742	175169	268923	false
	CPOR X	88307189	88614037	67669346	67882344	-	#01FC33	306848	212998	2708742	286113	2708742	3015590	false
	CPOR X	88900150	89361976	67180217	67666369	-	#01FC33	461826	486152	286113	0	286113	747939	false
	CPOR X	89361976	89443674	67080421	67172545	+	#01FC33	81698	92124	0	12019	0	81698	false
	CPOR X	89455693	89602702	66756553	67048151	-	#01FC33	147009	291598	12019	84350	12019	159028	false
	CPOR X	89687052	90058368	66281589	66731551	-	#01FC33	371316	449962	84350	115307	84350	455666	false
	CPOR X	90173675	90315083	65980728	66129503	-	#01FC33	141408	148775	115307	162781	115307	256715	false

	CPOR X	90477864	91841480	64612033	65817990	-	#01FC33	1363616	1205957	162781	43364	162781	1526397	false
	CPOR X	91884844	92326197	64050977	64529767	-	#01FC33	441353	478790	43364	62634	43364	484717	false
	CPOR X	92388831	93037578	63487519	64050837	-	#01FC33	648747	563318	62634	80638	62634	711381	false
	CPOR X	93118216	93638265	62994795	63474783	-	#01FC33	520049	479988	80638	50079	80638	600687	false
	CPOR X	93688344	93815282	62789728	62994174	-	#01FC33	126938	204446	50079	76185	50079	177017	false
	CPOR X	93891467	94772180	61835858	62678379	-	#01FC33	880713	842521	76185	11348	76185	956898	false
	CPOR X	94783528	94841904	61749665	61835609	+	#01FC33	58376	85944	11348	7159	11348	69724	false
	CPOR X	94849063	95281223	61400637	61749658	-	#01FC33	432160	349021	7159	9919	7159	439319	false
	CPOR X	95291142	95544974	61139856	61349551	-	#01FC33	253832	209695	9919	23038	9919	263751	false
	CPOR X	95568012	95665238	61024963	61139844	+	#01FC33	97226	114881	23038	5811	23038	120264	false
	CPOR X	95671049	96031904	60687798	61011563	-	#01FC33	360855	323765	5811	65782	5811	366666	false
	CPOR X	96097686	96584856	60173368	60650867	-	#01FC33	487170	477499	65782	-15204	65782	552952	false
	CPOR X	96569652	97363280	59394241	60124501	-	#01FC33	793628	730260	-15204	206928	-15204	778424	false
	CPOR X	97570208	98107683	58955205	59397968	-	#01FC33	537475	442763	206928	211374	206928	744403	false
	CPOR X	98319057	98391576	58694775	58762676	+	#01FC33	72519	67901	211374	82848	211374	283893	false
	CPOR X	98474424	98536547	58589659	58666956	+	#01FC33	62123	77297	82848	332439	82848	144971	false
	CPOR X	98868986	99472617	57997230	58540762	-	#01FC33	603631	543532	332439	63969	332439	936070	false
	CPOR X	99536586	101970731	55974838	57989521	-	#01FC33	2434145	2014683	63969	51392	63969	2498114	false
	CPOR X	102022123	102185503	55850903	55974443	-	#01FC33	163380	123540	51392	101861	51392	214772	false
	CPOR X	102287364	102381077	55607266	55701724	-	#01FC33	93713	94458	101861	-24127	101861	195574	false
	CPOR X	102356950	102458632	55242844	55488163	+	#01FC33	101682	245319	-24127	32401	-24127	77555	false
	CPOR X	102491033	102997118	54653122	55156319	-	#01FC33	506085	503197	32401	110251	32401	538486	false
	CPOR X	103107369	103502805	54085747	54474244	-	#01FC33	395436	388497	110251	16382	110251	505687	false
	CPOR X	103519187	104197944	53361940	54027665	-	#01FC33	678757	665725	16382	7067	16382	695139	false
	CPOR X	104205011	104832954	52753378	53309867	-	#01FC33	627943	556489	7067	50506	7067	635010	false
	CPOR X	104883460	105247517	52421622	52752521	-	#01FC33	364057	330899	50506	96226	50506	414563	false
	CPOR X	105343743	105408214	52306635	52364185	-	#01FC33	64471	57550	96226	93729	96226	160697	false
	CPOR X	105501943	105576767	52123595	52277755	-	#01FC33	74824	154160	93729	85343	93729	168553	false
	CPOR X	105662110	105891929	51785494	52123595	-	#01FC33	229819	338101	85343	69654	85343	315162	false
	CPOR X	105961583	107293084	50472209	51662721	-	#01FC33	1331501	1190512	69654	67636	69654	1401155	false
	CPOR X	107360720	107829466	49969732	50433297	-	#01FC33	468746	463565	67636	53863	67636	536382	false
	CPOR X	107883329	107945218	49852131	49944261	-	#01FC33	61889	92130	53863	34694	53863	115752	false

	CPOR X	107979912	110765674	47344387	49790430	-	#01FC33	2785762	2446043	34694	54645	34694	2820456	false
	CPOR X	110820319	111023840	47080000	47230637	+	#01FC33	203521	150637	54645	53564	54645	258166	false
	CPOR X	111077404	111230898	46854205	46965830	-	#01FC33	153494	111625	53564	9032	53564	207058	false
	CPOR X	111239930	112491134	45586947	46786384	-	#01FC33	1251204	1199437	9032	50964	9032	1260236	false
	CPOR X	112542098	113103336	45098507	45584598	-	#01FC33	561238	486091	50964	57662	50964	612202	false
	CPOR X	113160998	114060557	44105317	45096353	-	#01FC33	899559	991036	57662	85299	57662	957221	false
	CPOR X	114145856	114201168	43965297	44021389	-	#01FC33	55312	56092	85299	288367	85299	140611	false
	CPOR X	114489535	114673329	43414213	43614969	-	#01FC33	183794	200756	288367	102766	288367	472161	false
	CPOR X	114776095	115524278	42753940	43413702	-	#01FC33	748183	659762	102766	63497	102766	850949	false
	CPOR X	115587775	118433288	40036265	42696718	-	#01FC33	2845513	2660453	63497	196232	63497	2909010	false
	CPOR X	118629520	120225609	38241808	39675151	-	#01FC33	1596089	1433343	196232	3550	196232	1792321	false
	CPOR X	120229159	123047524	35551066	38188171	-	#01FC33	2818365	2637105	3550	10170	3550	2821915	false
	CPOR X	123057694	126544566	32179253	35493921	-	#01FC33	3486872	3314668	10170	4576	10170	3497042	false
	CPOR X	126549142	126740731	31905276	32100677	-	#01FC33	191589	195401	4576	44110	4576	196165	false
	CPOR X	126784841	127030615	31453562	31795702	+	#01FC33	245774	342140	44110	0	44110	289884	false
	CPOR X	127030615	127174840	31346748	31453544	-	#01FC33	144225	106796	0	0	0	144225	false
	CPOR X	127174840	127298318	31198498	31293552	-	#01FC33	123478	95054	0	64601	0	123478	false
	CPOR X	127362919	127412930	31125404	31195065	-	#01FC33	50011	69661	64601	2146	64601	114612	false
	CPOR X	127415076	127926243	30542151	31053887	-	#01FC33	511167	511736	2146	12801	2146	513313	false
	CPOR X	127939044	129347261	28860569	30205599	-	#01FC33	1408217	1345030	12801	-13788	12801	1421018	false
	CPOR X	129333473	131474207	26586865	28538329	-	#01FC33	2140734	1951464	-13788	53999	-13788	2126946	false
	CPOR X	131528206	134957891	23260185	26514501	-	#01FC33	3429685	3254316	53999	13029	53999	3483684	false
	CPOR X	134970920	135051560	22919377	22987402	+	#01FC33	80640	68025	13029	-30185	13029	93669	false
	CPOR X	135021375	135076750	22359753	22427714	-	#01FC33	55375	67961	-30185	-34726	-30185	25190	false
	CPOR X	135042024	135138136	21204067	21319139	-	#01FC33	96112	115072	-34726	-6639	-34726	61386	false
	CPOR X	135131497	135350583	20739827	21071351	-	#01FC33	219086	331524	-6639	-65112	-6639	212447	false
	CPOR X	135285471	135618417	20017167	20467967	+	#01FC33	332946	450800	-65112	-36519	-65112	267834	false
	CPOR X	135581898	135637580	20717637	20839271	+	#01FC33	55682	121634	-36519	-55016	-36519	19163	false
	CPOR X	135582564	145042120	11186144	20109147	-	#01FC33	9459556	8923003	-55016	27818	-55016	9404540	false
	CPOR X	145069938	146865966	9194448	11010451	-	#01FC33	1796028	1816003	27818	-3682	27818	1823846	false
	CPOR X	146862284	147074944	8973265	9186865	+	#01FC33	212660	213600	-3682	39410	-3682	208978	false
	CPOR X	147114354	149183498	6885904	8857218	-	#01FC33	2069144	1971314	39410	-531	39410	2108554	false

	CPOR X	149182967	149384869	5233522	5446176	+	#01FC33	201902	212654	-531	191854	-531	201371	false
	CPOR X	149576723	149715024	6723100	6841114	+	#01FC33	138301	118014	191854	-123561	191854	330155	false
	CPOR X	149591463	149765543	6226750	6365534	+	#01FC33	174080	138784	-123561	4831	-123561	50519	false
	CPOR X	149770374	150354094	3649062	4113201	-	#01FC33	583720	464139	4831	-458951	4831	588551	false
	CPOR X	149895143	149966793	6512818	6581845	-	#01FC33	71650	69027	-458951	403677	-458951	-387301	true
	CPOR X	150370470	150513617	3467614	3645942	+	#01FC33	143147	178328	403677	-7795	403677	546824	false
	CPOR X	150505822	151259266	2783900	3456359	-	#01FC33	753444	672459	-7795	4879	-7795	745649	false
	CPOR X	151264145	152068050	1597625	2310649	-	#01FC33	803905	713024	4879	56570	4879	808784	false
	CPOR X	152124620	152294811	1372738	1555415	-	#01FC33	170191	182677	56570	2222	56570	226761	false
	CPOR X	152297033	152730871	734599	1184137	-	#01FC33	433838	449538	2222	1970	2222	436060	false
	CPOR X	152732841	153250973	0	644339	-	#01FC33	518132	644339	1970		1970	520102	false

The column names are similar to the column names in the Supplementary Table S1, but the color code of the chromosome corresponds to the block in the query genome in the Figure 3 and in the Supplementary Figure S4.

Supplementary Table S3 (part 1). Syntenic blocks with a minimum cut-off length of 50 kilobases in the reference human genome.

scaffold	query	start	end	query_start	query_end	strand	color	target_len	query_len	dist_upstream	dist_downstream	dist_end_start	dist_end_end	embedded
**HSA 1**	HGL 7	903444	1683185	106227435	106798643	-	#C2FB8F	779741	571208		16484			
	HGL 7	1699669	2647825	105645063	106279647	-	#C2FB8F	948156	634584	16484	132726	16484	964640	false
	HGL 7	2780551	8881496	101417717	105644816	-	#C2FB8F	6100945	4227099	132726	60513	132726	6233671	false
	HGL 7	8942009	12778186	98527889	101402074	-	#C2FB8F	3836177	2874185	60513	8131	60513	3896690	false
	HGL 7	12786317	12884323	98445509	98515637	+	#C2FB8F	98006	70128	8131	542849	8131	106137	false
	HGL 7	13427172	16466235	56816501	59322338	+	#C2FB8F	3039063	2505837	542849	445716	542849	3581912	false
	HGL 7	16911951	20009043	59322807	61824624	+	#C2FB8F	3097092	2501817	445716	46388	445716	3542808	false
	HGL 7	20055431	21387522	61929318	63136224	+	#C2FB8F	1332091	1206906	46388	99076	46388	1378479	false
	HGL 7	21486598	25268026	63139130	66260363	+	#C2FB8F	3781428	3121233	99076	62695	99076	3880504	false
	HGL 7	25330721	43121732	66255444	81964184	+	#C2FB8F	17791011	15708740	62695	-13605474	62695	17853706	false
	HGL 7	29516258	29733614	70736626	70940827	-	#C2FB8F	217356	204201	-13605474	-62098	-13605474	-13388118	true
	HGL 7	29671516	29745012	70339225	70417477	+	#C2FB8F	73496	78252	-62098	-1682	-62098	11398	false
	HGL 7	29743330	29960252	70448726	70682450	-	#C2FB8F	216922	233724	-1682	-169323	-1682	215240	false
	HGL 7	29790929	30050350	70678335	70936484	-	#C2FB8F	259421	258149	-169323	-79676	-169323	90098	false
	HGL 7	29970674	30048348	69848562	69931364	+	#C2FB8F	77674	82802	-79676	3874	-79676	-2002	true

	HGL 7	30052222	30123164	70742523	70793124	+	#C2FB8F	70942	50601	3874	-38239	3874	74816	false
	HGL 7	30084925	30135061	70081254	70138937	-	#C2FB8F	50136	57683	-38239	-45950	-38239	11897	false
	HGL 7	30089111	30190141	70302978	70368902	-	#C2FB8F	101030	65924	-45950	9366	-45950	55080	false
	HGL 7	30199507	30452033	69836453	70058153	-	#C2FB8F	252526	221700	9366	-115254	9366	261892	false
	HGL 7	30336779	30421230	70154804	70213829	-	#C2FB8F	84451	59025	-115254	43865	-115254	-30803	true
	HGL 7	30465095	30538267	70418093	70474104	+	#C2FB8F	73172	56011	43865	-61061	43865	117037	false
	HGL 7	30477206	30708342	69963978	70151130	-	#C2FB8F	231136	187152	-61061	-122321	-61061	170075	false
	HGL 7	30586021	30695474	70534079	70637473	+	#C2FB8F	109453	103394	-122321	8755124	-122321	-12868	true
	HGL 7	39450598	39562599	78983994	79049205	-	#C2FB8F	112001	65211	8755124	172075	8755124	8867125	false
	HGL 7	39734674	39797934	78746242	78833878	-	#C2FB8F	63260	87636	172075	3325705	172075	235335	false
	HGL 7	43123639	44154633	82188767	83122798	+	#C2FB8F	1030994	934031	3325705	65	3325705	4356699	false
	HGL 7	44154698	45121125	83257148	84230091	+	#C2FB8F	966427	972943	65	46827	65	966492	false
	HGL 7	45167952	46824921	84299761	85891071	+	#C2FB8F	1656969	1591310	46827	105073	46827	1703796	false
	HGL 7	46929994	47050852	85998395	86117117	+	#C2FB8F	120858	118722	105073	120558	105073	225931	false
	HGL 7	47171410	48112525	86145328	87064656	+	#C2FB8F	941115	919328	120558	103654	120558	1061673	false
	HGL 7	48216179	49907626	87125848	88659228	+	#C2FB8F	1691447	1533380	103654	57108	103654	1795101	false
	HGL 7	49964734	54642440	88663059	93067907	+	#C2FB8F	4677706	4404848	57108	66377	57108	4734814	false
	HGL 7	54708817	59927569	93068824	97760369	+	#C2FB8F	5218752	4691545	66377	86613870	66377	5285129	false
	HGL 20	59945679	65110082	14433	3500101	+	#F7FE2D	5164403	3485668		52070			
	HGL 20	65162152	67462995	3515029	5291198	+	#F7FE2D	2300843	1776169	52070	206419	52070	2352913	false
	HGL 20	67669414	67959118	5339065	5497852	+	#F7FE2D	289704	158787	206419	95586	206419	496123	false
	HGL 20	68054704	69264177	5497855	6429460	+	#F7FE2D	1209473	931605	95586	52618	95586	1305059	false
	HGL 20	69316795	70762826	6483495	7651932	+	#F7FE2D	1446031	1168437	52618	56461	52618	1498649	false
	HGL 20	70819287	83210092	7697448	18767192	+	#F7FE2D	12390805	11069744	56461	77058	56461	12447266	false
	HGL 20	83287150	86585701	18861616	22001599	+	#F7FE2D	3298551	3139983	77058	-45580	77058	3375609	false
	HGL 20	86540121	89300734	22074821	24704709	+	#F7FE2D	2760613	2629888	-45580	-192011	-45580	2715033	false
	HGL 20	89108723	89202524	24488244	24548131	+	#F7FE2D	93801	59887	-192011	55379	-192011	-98210	true
	HGL 20	89257903	89383086	24702828	24759084	-	#F7FE2D	125183	56256	55379	-7061	55379	180562	false
	HGL 20	89376025	89429384	24486105	24555988	-	#F7FE2D	53359	69883	-7061	-40546	-7061	46298	false
	HGL 20	89388838	89455842	24591025	24716591	+	#F7FE2D	67004	125566	-40546	-48577	-40546	26458	false
	HGL 20	89407265	93041451	24746259	28320865	+	#F7FE2D	3634186	3574606	-48577	1974	-48577	3585609	false
	HGL 20	93043425	93382554	28432273	28830421	+	#F7FE2D	339129	398148	1974	51756	1974	341103	false

	HGL 20	93434310	97512534	28848933	33087890	+	#F7FE2D	4078224	4238957	51756	-27401	51756	4129980	false
	HGL 20	97485133	100179603	33145203	36064290	+	#F7FE2D	2694470	2919087	-27401	12116	-27401	2667069	false
	HGL 20	100191719	103632266	36118168	39771488	+	#F7FE2D	3440547	3653320	12116	-48050	12116	3452663	false
	HGL 20	103584216	108212397	39793069	44206312	+	#F7FE2D	4628181	4413243	-48050	271301	-48050	4580131	false
	HGL 20	108483698	109723848	44175444	45256810	+	#F7FE2D	1240150	1081366	271301	-68007	271301	1511451	false
	HGL 20	109655841	110818162	45282818	46397755	+	#F7FE2D	1162321	1114937	-68007	22873	-68007	1094314	false
	HGL 20	110841035	110967312	46480701	46621584	+	#F7FE2D	126277	140883	22873	146189	22873	149150	false
	HGL 20	111113501	112818630	46774306	48434028	+	#F7FE2D	1705129	1659722	146189	-23112	146189	1851318	false
	HGL 20	112795518	112853597	48485677	48578864	+	#F7FE2D	58079	93187	-23112	13967	-23112	34967	false
	HGL 20	112867564	112929863	48423367	48478252	+	#F7FE2D	62299	54885	13967	-61370	13967	76266	false
	HGL 20	112868493	119662899	48491541	55080681	+	#F7FE2D	6794406	6589140	-61370	-193848	-61370	6733036	false
	HGL 20	119469051	119616353	54918196	54996952	+	#F7FE2D	147302	78756	-193848	48154	-193848	-46546	true
	HGL 20	119664507	119830094	55200605	55413498	-	#F7FE2D	165587	212893	48154	75166	48154	213741	false
	HGL 20	119905260	120319916	55437993	55860618	+	#F7FE2D	414656	422625	75166	166962	75166	489822	false
	HGL 20	120486878	120705588	55718186	55966728	-	#F7FE2D	218710	248542	166962	0	166962	385672	false
	HGL 20	120705588	120801217	55518500	55619925	-	#F7FE2D	95629	101425	0	219530	0	95629	false
	HGL 20	121020747	121122313	57380461	57497637	+	#F7FE2D	101566	117176	219530	22681699	219530	321096	false
	HGL 20	143804012	143909665	57380461	57497637	+	#F7FE2D	105653	117176	22681699	1690866	22681699	22787352	false
	HGL 25	143938007	144044467	49600850	49702069	+	#682292	106460	101219	22546935	844848	22546935	22653395	false
	HGL 25	144889315	145128659	49604718	49816254	-	#682292	239344	211536	844848	25910130	844848	1084192	false
	HGL 20	145600531	146050941	56849833	57272116	+	#F7FE2D	450410	422283	1690866	98204	1690866	2141276	false
	HGL 20	146149145	146328865	55518500	55699657	+	#F7FE2D	179720	181157	98204	772931	98204	277924	false
	HGL 20	147101796	147296618	55976995	56118050	+	#F7FE2D	194822	141055	772931	34381	772931	967753	false
	HGL 20	147330999	147456612	56177379	56293928	+	#F7FE2D	125613	116549	34381	28921	34381	159994	false
	HGL 20	147485533	148009863	56391273	56912213	+	#F7FE2D	524330	520940	28921	590224	28921	553251	false
	HGL 20	148600087	149034987	55518500	55966728	+	#F7FE2D	434900	448228	590224	162771	590224	1025124	false
	HGL 20	149197758	149361340	55675538	55860618	-	#F7FE2D	163582	185080	162771	5209	162771	326353	false
	HGL 20	149366549	149471552	55518500	55624488	-	#F7FE2D	105003	105988	5209	241018	5209	110212	false
	HGL 20	149712570	150724703	57380461	58280054	+	#F7FE2D	1012133	899593	241018	59774	241018	1253151	false
	HGL 20	150784477	151375388	58311697	58872376	+	#F7FE2D	590911	560679	59774	22737	59774	650685	false
	HGL 20	151398125	151862887	58952726	59365610	+	#F7FE2D	464762	412884	22737	8	22737	487499	false
	HGL 20	151862895	152074950	59365707	59544015	-	#F7FE2D	212055	178308	8	343	8	212063	false

	HGL 20	152075293	152246410	59544203	59704440	+	#F7FE2D	171117	160237	343	51644	343	171460	false
	HGL 20	152298054	153414439	59689646	60680091	+	#F7FE2D	1116385	990445	51644	88776	51644	1168029	false
	HGL 20	153503215	155303978	62136048	63505227	-	#F7FE2D	1800763	1369179	88776	1319	88776	1889539	false
	HGL 20	155305297	155686591	60811796	61086412	+	#F7FE2D	381294	274616	1319	-827	1319	382613	false
	HGL 20	155685764	157157608	61006072	62126870	+	#F7FE2D	1471844	1120798	-827	-4756	-827	1471017	false
	HGL 20	157152852	157322813	60699875	60807233	-	#F7FE2D	169961	107358	-4756	74712	-4756	165205	false
	HGL 20	157397525	157496542	60699875	60810934	+	#F7FE2D	99017	111059	74712		74712	173729	false
	HGL 27	157489663	157692861	22622595	22798482	-	#FF7F00	203198	175887		90730			
	HGL 27	157783591	158149865	22290677	22613369	-	#FF7F00	366274	322692	90730	52612	90730	457004	false
	HGL 27	158202477	158272179	5814134	5868511	+	#FF7F00	69702	54377	52612	245103	52612	122314	false
	HGL 27	158517282	158713315	22099383	22241238	+	#FF7F00	196033	141855	245103	8	245103	441136	false
	HGL 27	158713323	160891843	19923549	22060049	-	#FF7F00	2178520	2136500	8	-2050949	8	2178528	false
	HGL 27	158840894	159001859	21752184	21853691	-	#FF7F00	160965	101507	-2050949	1946048	-2050949	-1889984	true
	HGL 27	160947907	161429252	19510886	19934984	-	#FF7F00	481345	424098	1946048	74122	1946048	2427393	false
	HGL 27	161503374	161611328	19394884	19487884	-	#FF7F00	107954	93000	74122	-20212	74122	182076	false
	HGL 27	161591116	166687608	14674666	19479676	-	#FF7F00	5096492	4805010	-20212	70593	-20212	5076280	false
	HGL 27	166758201	170012691	11633727	14733332	-	#FF7F00	3254490	3099605	70593	-109306	70593	3325083	false
	HGL 27	169903385	170930989	10341494	11411078	+	#FF7F00	1027604	1069584	-109306		-109306	918298	false
	HGL 25	171038789	171776538	16495714	17051642	+	#682292	737749	555928	25910130	4600	25910130	26647879	false
	HGL 25	171781138	178150938	17131960	22964230	+	#682292	6369800	5832270	4600	58047	4600	6374400	false
	HGL 25	178208985	179379078	22966783	24081325	+	#682292	1170093	1114542	58047	101811	58047	1228140	false
	HGL 25	179480889	179704560	24092779	24327497	+	#682292	223671	234718	101811	1387	101811	325482	false
	HGL 25	179705947	179779202	26965757	27033786	+	#682292	73255	68029	1387	-37064	1387	74642	false
	HGL 25	179742138	182449483	24332529	27034228	-	#682292	2707345	2701699	-37064	115323	-37064	2670281	false
	HGL 25	182564806	184673131	27034772	29382433	+	#682292	2108325	2347661	115323	-35200	115323	2223648	false
	HGL 25	184637931	194221593	29383162	39087261	+	#682292	9583662	9704099	-35200	977	-35200	9548462	false
	HGL 25	194222570	194325039	39201493	39285256	-	#682292	102469	83763	977	74407	977	103446	false
	HGL 25	194399446	196832715	39285430	41288729	+	#682292	2433269	2003299	74407	102608	74407	2507676	false
	HGL 25	196935323	197024723	41290168	41352165	-	#682292	89400	61997	102608	8310	102608	192008	false
	HGL 25	197033033	200622446	41352397	44707827	+	#682292	3589413	3355430	8310	0	8310	3597723	false
	HGL 25	200622446	201975171	44783866	45989215	+	#682292	1352725	1205349	0	5067	0	1352725	false
	HGL 25	201980238	205851601	46043773	49394472	+	#682292	3871363	3350699	5067	56925	5067	3876430	false

HGL 25	205908526	207665664	49405476	50746902	+	#682292	1757138	1341426	56925	1204	56925	1814063	false
HGL 25	207666868	211493072	50661054	53984480	+	#682292	3826204	3323426	1204	18985	1204	3827408	false
HGL 25	211512057	215458564	6703284	9812043	-	#682292	3946507	3108759	18985	82108	18985	3965492	false
HGL 25	215540672	222447648	1603335	6702515	-	#682292	6906976	5099180	82108	73403	82108	6989084	false
HGL 25	222521051	223558898	1030565	1599278	-	#682292	1037847	568713	73403	50074	73403	1111250	false
HGL 25	223608972	223847471	911177	1030468	-	#682292	238499	119291	50074	194817	50074	288573	false
HGL 25	224042288	224538635	600440	901908	-	#682292	496347	301468	194817	5248	194817	691164	false
HGL 25	224543883	224810562	39669	144403	+	#682292	266679	104734	5248	517922	5248	271927	false
HGL 25	225328484	225881306	146714	587805	+	#682292	552822	441091	517922	36275	517922	1070744	false
HGL 25	225917581	226831819	53988307	54742351	-	#682292	914238	754044	36275	84574	36275	950513	false
HGL 25	226916393	227463930	14290948	14774108	-	#682292	547537	483160	84574	12549854	84574	632111	false
HGL 4	228661429	229163594	25520401	25693795	+	#0C404C	502165	173394	15721213	52799	15721213	16223378	false
HGL 4	229216393	234490100	25694931	29617999	+	#0C404C	5273707	3923068	52799	1413	52799	5326506	false
HGL 4	234491513	235094180	29618011	30109707	-	#0C404C	602667	491696	1413	5260	1413	604080	false
HGL 4	235099440	239931861	30112506	34547257	+	#0C404C	4832421	4434751	5260		5260	4837681	false
HGL 25	240013784	241873643	14802462	16454894	-	#682292	1859859	1652432	12549854	122002	12549854	14409713	false
HGL 25	241995645	243024826	9857463	10766163	+	#682292	1029181	908700	122002	88861	122002	1151183	false
HGL 25	243113687	246962717	10790402	14278463	+	#682292	3849030	3488061	88861		88861	3937891	false
HGL 11	247296485	247479264	124501702	124622386	-	#7F00FF	182779	120684	18776204	-10830	18776204	18958983	false
HGL 11	247468434	247537099	68825657	68884420	-	#7F00FF	68665	58763	-10830	345968	-10830	57835	false
HGL 9	247470360	247537099	20617413	20720844	-	#6E0025	66739	103431	177886990	134187	177886990	177953729	false
HGL 9	247671286	247742255	21961080	22043855	+	#6E0025	70969	82775	134187	92630	134187	205156	false
HGL 9	247834885	247970688	22393822	22476017	-	#6E0025	135803	82195	92630	-40942	92630	228433	false
HGL 11	247883067	248048153	69062956	69115829	+	#7F00FF	165086	52873	345968	-128255	345968	511054	false
HGL 11	247919898	248063510	66198581	66251629	-	#7F00FF	143612	53048	-128255	-82047	-128255	15357	false
HGL 9	247929746	248100738	27019711	27116281	+	#6E0025	170992	96570	-40942	112154	-40942	130050	false
HGL 11	247981463	248100738	68613549	68664109	-	#7F00FF	119275	50560	-82047	37494	-82047	37228	false
HGL 11	248138232	248275087	67991877	68050915	-	#7F00FF	136855	59038	37494	-136855	37494	174349	false
HGL 11	248138232	248279052	69722708	69821963	-	#7F00FF	140820	99255	-136855	-140820	-136855	3965	false
HGL 11	248138232	248295863	67204546	67256396	+	#7F00FF	157631	51850	-140820	393157	-140820	16811	false
HGL 9	248212892	248279052	27165310	27245797	-	#6E0025	66160	80487	112154		112154	178314	false
HGL 11	248689020	248918969	123168261	123405821	-	#7F00FF	229949	237560	393157		393157	623106	false

**HSA 10**	HGL 4	88592	2260811	34595050	36976313	+	#0C404C	2172219	2381263		1621			
	HGL 4	2262432	2409997	36986167	37219115	-	#0C404C	147565	232948	1621	1638	1621	149186	false
	HGL 4	2411635	3186999	37222302	38195053	+	#0C404C	775364	972751	1638	2220678	1638	777002	false
	HGL 6	3181979	4875311	103279295	104951878	+	#60FA36	1693332	1672583		-17995			
	HGL 6	4857316	4923255	105124095	105214799	-	#60FA36	65939	90704	-17995	124524	-17995	47944	false
	HGL 6	5047779	5105714	105145866	105195934	+	#60FA36	57935	50068	124524	-57935	124524	182459	false
	HGL 6	5047779	5107936	105121832	105197283	+	#60FA36	60157	75451	-57935	194426	-57935	2222	false
	HGL 6	5302362	5407032	105231057	105311489	+	#60FA36	104670	80432	194426	10003992	194426	299096	false
	HGL 4	5407677	5669797	44967222	45263418	-	#0C404C	262120	296196	2220678	3274	2220678	2482798	false
	HGL 4	5673071	5875422	44768586	44959379	+	#0C404C	202351	190793	3274	1	3274	205625	false
	HGL 4	5875423	12093571	38212412	44714024	-	#0C404C	6218148	6501612	1		1	6218149	false
	HGL 27	12058685	13298547	4811613	5812389	+	#FF7F00	1239862	1000776		16227			
	HGL 27	13314774	14973481	533742	1876105	-	#FF7F00	1658707	1342363	16227	53677	16227	1674934	false
	HGL 27	15027158	15414481	179490	533742	-	#FF7F00	387323	354252	53677	12072509	53677	441000	false
	HGL 6	15411024	26622661	93237868	102953666	+	#60FA36	11211637	9715798	10003992	10845	10003992	21215629	false
	HGL 6	26633506	26872931	102913035	103116825	+	#60FA36	239425	203790	10845	222805	10845	250270	false
	HGL 6	27095736	27242291	103121656	103258925	-	#60FA36	146555	137269	222805		222805	369360	false
	HGL 27	27486990	28636818	5905256	6903611	+	#FF7F00	1149828	998355	12072509	7837	12072509	13222337	false
	HGL 27	28644655	29400317	1945723	2548885	+	#FF7F00	755662	603162	7837	43558	7837	763499	false
	HGL 27	29443875	30349535	2649098	3329283	-	#FF7F00	905660	680185	43558	60411	43558	949218	false
	HGL 27	30409946	30617880	2476772	2648007	-	#FF7F00	207934	171235	60411	33269	60411	268345	false
	HGL 27	30651149	32420888	3224798	4796775	+	#FF7F00	1769739	1571977	33269	474502	33269	1803008	false
	HGL 27	32895390	35220422	6917187	8808363	-	#FF7F00	2325032	1891176	474502	6731	474502	2799534	false
	HGL 27	35227153	36879500	8826133	10257159	-	#FF7F00	1652347	1431026	6731		6731	1659078	false
	HGL 10	37755779	37903698	81424498	81568124	-	#00007F	147919	143626	10704794	43136	10704794	10852713	false
	HGL 10	37946834	38057630	81421057	81483773	-	#00007F	110796	62716	43136	4726393	43136	153932	false
	HGL 10	42784023	43462053	81575319	82040893	+	#00007F	678030	465574	4726393	93534	4726393	5404423	false
	HGL 10	43555587	44938667	82048232	83124281	+	#00007F	1383080	1076049	93534	-476727	93534	1476614	false
	HGL 10	44461940	44938689	80444209	80799397	+	#00007F	476749	355188	-476727	435156	-476727	22	false
	HGL 10	45373845	45601010	81074870	81233239	-	#00007F	227165	158369	435156	11380	435156	662321	false
	HGL 10	45612390	45723249	79628640	79711544	+	#00007F	110859	82904	11380	612	11380	122239	false
	HGL 10	45723861	45795688	79654993	79733298	-	#00007F	71827	78305	612	-71827	612	72439	false

	HGL 10	45723861	45800807	80898492	80971806	+	#00007F	76946	73314	-71827	76018	-71827	5119	false
	HGL 10	45876825	46035729	77445161	77596040	-	#00007F	158904	150879	76018	352751	76018	234922	false
	HGL 10	46388480	46453372	83124707	83208221	+	#00007F	64892	83514	352751	126967	352751	417643	false
	HGL 10	46580339	46665611	79220335	79316747	+	#00007F	85272	96412	126967	371661	126967	212239	false
	HGL 10	47037272	47323862	78948410	79219022	+	#00007F	286590	270612	371661	352555	371661	658251	false
	HGL 10	47676417	47761469	79220335	79316747	-	#00007F	85052	96412	352555	169783	352555	437607	false
	HGL 10	47931252	47988907	83131760	83208221	-	#00007F	57655	76461	169783	357127	169783	227438	false
	HGL 10	48346034	49969746	77480560	78944438	-	#00007F	1623712	1463878	357127	52251	357127	1980839	false
	HGL 10	50021997	50136256	79654993	79738448	-	#00007F	114259	83455	52251	-114259	52251	166510	false
	HGL 10	50021997	50141381	80893244	80971806	+	#00007F	119384	78562	-114259	29778411	-114259	5125	false
	HGL 13	50194148	50655359	40286723	40877045	+	#097F8D	461211	590322		133251			
	HGL 13	50788610	56031978	35194762	40236394	-	#097F8D	5243368	5041632	133251	-2769148	133251	5376619	false
	HGL 13	53262830	53318609	34819506	34879925	+	#097F8D	55779	60419	-2769148	156401	-2769148	-2713369	true
	HGL 13	53475010	53538264	35074553	35147504	+	#097F8D	63254	72951	156401	2471626	156401	219655	false
	HGL 13	56009890	56450375	34810067	35178899	-	#097F8D	440485	368832	2471626	-131871	2471626	2912111	false
	HGL 13	56318504	56401695	33590246	33686419	-	#097F8D	83191	96173	-131871	49688	-131871	-48680	true
	HGL 13	56451383	67552200	23486860	34709495	-	#097F8D	11100817	11222635	49688	-9679646	49688	11150505	false
	HGL 13	57872554	57924078	34319537	34393988	-	#097F8D	51524	74451	-9679646	85925	-9679646	-9628122	true
	HGL 13	58010003	58166342	34125454	34252455	-	#097F8D	156339	127001	85925	9439065	85925	242264	false
	HGL 13	67605407	67861570	23233754	23465974	-	#097F8D	256163	232220	9439065	17980	9439065	9695228	false
	HGL 13	67879550	69018130	22241123	23175327	-	#097F8D	1138580	934204	17980	6000	17980	1156560	false
	HGL 13	69024130	73659578	18215563	22126081	-	#097F8D	4635448	3910518	6000	81573	6000	4641448	false
	HGL 13	73741151	79498116	13036128	18215055	-	#097F8D	5756965	5178927	81573	217100	81573	5838538	false
	HGL 13	79715216	79794251	40877045	40996759	-	#097F8D	79035	119714	217100	233929	217100	296135	false
	HGL 10	79919792	80053805	70804681	70969668	-	#00007F	134013	164987	29778411	205004	29778411	29912424	false
	HGL 13	80028180	80211643	12897815	13019129	+	#097F8D	183463	121314	233929	7025798	233929	417392	false
	HGL 10	80258809	84556897	70990745	75340940	+	#00007F	4298088	4350195	205004	33360	205004	4503092	false
	HGL 10	84590257	86529279	75399259	77339738	+	#00007F	1939022	1940479	33360	77387	33360	1972382	false
	HGL 10	86606666	86730153	77341939	77443781	+	#00007F	123487	101842	77387	10590	77387	200874	false
	HGL 10	86740743	87216735	79223768	79622802	-	#00007F	475992	399034	10590		10590	486582	false
	HGL 13	87237441	87316418	40877045	40996759	-	#097F8D	78977	119714	7025798	113758	7025798	7104775	false
	HGL 13	87430176	94612562	40889860	48821154	+	#097F8D	7182386	7931294	113758	68321	113758	7296144	false

	HGL 13	94680883	94735254	49225296	49282545	+	#097F8D	54371	57249	68321	-41740	68321	122692	false
	HGL 13	94693514	94854609	48923292	49049576	+	#097F8D	161095	126284	-41740	-73887	-41740	119355	false
	HGL 13	94780722	95036547	48935029	49049438	+	#097F8D	255825	114409	-73887	-29753	-73887	181938	false
	HGL 13	95006794	95066231	48923292	49013772	-	#097F8D	59437	90480	-29753	-29680	-29753	29684	false
	HGL 13	95036551	95116937	49225296	49294266	-	#097F8D	80386	68970	-29680	-79451	-29680	50706	false
	HGL 13	95037486	95150297	48972338	49048226	-	#097F8D	112811	75888	-79451	84629	-79451	33360	false
	HGL 13	95234926	95338333	49355991	49445998	+	#097F8D	103407	90007	84629	-26940	84629	188036	false
	HGL 13	95311393	95880521	49519302	50092660	+	#097F8D	569128	573358	-26940	25340	-26940	542188	false
	HGL 13	95905861	97206311	50169518	51538790	+	#097F8D	1300450	1369272	25340	16	25340	1325790	false
	HGL 13	97206327	99771386	51747291	54381302	+	#097F8D	2565059	2634011	16	10931	16	2565075	false
	HGL 13	99782317	100421136	54466035	55066263	+	#097F8D	638819	600228	10931	-124752	10931	649750	false
	HGL 13	100296384	120290701	69395029	87301593	-	#097F8D	19994317	17906564	-124752	421	-124752	19869565	false
	HGL 13	120291122	122574276	67028938	69336112	-	#097F8D	2283154	2307174	421	65601	421	2283575	false
	HGL 13	122639877	122831390	51562399	51747260	+	#097F8D	191513	184861	65601	12060	65601	257114	false
	HGL 13	122843450	130348606	59547685	66972436	-	#097F8D	7505156	7424751	12060	5	12060	7517216	false
	HGL 13	130348611	133423664	56167516	59496470	-	#097F8D	3075053	3328954	5	83632	5	3075058	false
	HGL 13	133507296	133561533	55164208	55250211	-	#097F8D	54237	86003	83632		83632	137869	false
**HSA 11**	HGL 8	192132	335286	124749347	124921250	-	#6A78D4	143154	171903		-27547			
	HGL 8	307739	1577388	123869528	124745457	-	#6A78D4	1269649	875929	-27547	2027718	-27547	1242102	false
	HGL 22	1620561	3239511	25173020	26605541	+	#00FFFF	1618950	1432521		29569600			
	HGL 19	3401523	3524021	20846187	20918527	-	#0000FF	122498	72340		1385			
	HGL 19	3525406	3592334	20755433	20824442	+	#0000FF	66928	69009	1385	64192703	1385	68313	false
	HGL 8	3605106	4194647	98015497	98577719	+	#6A78D4	589541	562222	2027718	179660	2027718	2617259	false
	HGL 8	4374307	4737316	98579287	98851018	+	#6A78D4	363009	271731	179660	-45458	179660	542669	false
	HGL 8	4691858	5110272	98850942	99283219	+	#6A78D4	418414	432277	-45458	19666	-45458	372956	false
	HGL 8	5129938	5804082	99577457	100300002	+	#6A78D4	674144	722545	19666	35559	19666	693810	false
	HGL 8	5839641	6747596	100387987	101195301	+	#6A78D4	907955	807314	35559	-38682	35559	943514	false
	HGL 8	6708914	6788491	101261985	101405259	+	#6A78D4	79577	143274	-38682	58	-38682	40895	false
	HGL 8	6788549	7795977	101519099	102390990	+	#6A78D4	1007428	871891	58	-68877	58	1007486	false
	HGL 8	7727100	18118624	102569557	112394983	+	#6A78D4	10391524	9825426	-68877	104678	-68877	10322647	false
	HGL 8	18223302	18806863	112399479	112894977	+	#6A78D4	583561	495498	104678	-5694	104678	688239	false
	HGL 8	18801169	18973428	112995860	113079803	+	#6A78D4	172259	83943	-5694	-107803	-5694	166565	false

HGL 8	18865625	32796426	113176011	123839539	+	#6A78D4	13930801	10663528	-107803	-13894192	-107803	13822998	false
HGL 8	18902234	19023605	112895893	113002936	+	#6A78D4	121371	107043	-13894192	4706867	-13894192	-13772821	true
HGL 8	23730472	23788163	117618120	117686072	+	#6A78D4	57691	67952	4706867	452770	4706867	4764558	false
HGL 8	24240933	24435438	116648436	116759391	-	#6A78D4	194505	110955	452770	24710578	452770	647275	false
HGL 22	32809111	33743103	24041846	24978522	-	#00FFFF	933992	936676	29569600	-46665	29569600	30503592	false
HGL 22	33696438	33794986	23926023	24031672	+	#00FFFF	98548	105649	-46665	13352	-46665	51883	false
HGL 22	33808338	37446763	20364658	23916032	-	#00FFFF	3638425	3551374	13352	43340	13352	3651777	false
HGL 22	37490103	48226581	11500877	20283783	-	#00FFFF	10736478	8782906	43340	91387	43340	10779818	false
HGL 22	48317968	48497313	11164229	11351900	-	#00FFFF	179345	187671	91387	1418037	91387	270732	false
HGL 8	49146016	49208984	80462558	80520666	+	#6A78D4	62968	58108	24710578	172736	24710578	24773546	false
HGL 8	49381720	49459671	80847070	80913812	+	#6A78D4	77951	66742	172736	32371	172736	250687	false
HGL 8	49492042	49592500	81015569	81110069	+	#6A78D4	100458	94500	32371	22323123	32371	132829	false
HGL 22	49915350	50060782	11164982	11344601	+	#00FFFF	145432	179619	1418037	4519009	1418037	1563469	false
HGL 22	54579791	54668266	11164229	11290603	-	#00FFFF	88475	126374	4519009	10036	4519009	4607484	false
HGL 3	54669015	54722633	44318144	44381791	-	#1240C3	53618	63647		876230			
HGL 22	54678302	54739515	11156661	11233465	+	#00FFFF	61213	76804	10036	574050	10036	71249	false
HGL 22	55313565	55473678	11155940	11236008	+	#00FFFF	160113	80068	574050	-19812	574050	734163	false
HGL 22	55453866	55554806	10915333	10982941	-	#00FFFF	100940	67608	-19812	48083	-19812	81128	false
HGL 9	55453866	55555514	25840292	25923378	+	#6E0025	101648	83086		-101648			
HGL 9	55453866	55575025	20136385	20265535	-	#6E0025	121159	129150	-101648	80488	-101648	19511	false
HGL 3	55598863	55727262	41996200	42133295	+	#1240C3	128399	137095	876230	66882	876230	1004629	false
HGL 22	55602889	55728734	10597487	10719306	-	#00FFFF	125845	121819	48083	65410	48083	173928	false
HGL 9	55655513	55728734	24282920	24378893	+	#6E0025	73221	95973	80488	65410	80488	153709	false
HGL 22	55794144	55935722	10431836	10526048	-	#00FFFF	141578	94212	65410	88043	65410	206988	false
HGL 3	55794144	55935722	45974176	46089844	-	#1240C3	141578	115668	66882	-126812	66882	208460	false
HGL 9	55794144	55935722	24417753	24472038	+	#6E0025	141578	54285	65410	112414	65410	206988	false
HGL 3	55808910	55943604	43400837	43581869	+	#1240C3	134694	181032	-126812	80161	-126812	7882	false
HGL 3	56023765	56125553	45613911	45715334	+	#1240C3	101788	101423	80161	-101788	80161	181949	false
HGL 22	56023765	56129221	9670656	9798307	+	#00FFFF	105456	127651	88043	11981	88043	193499	false
HGL 3	56023765	56129221	42962035	43041373	+	#1240C3	105456	79338	-101788	6394	-101788	3668	false
HGL 9	56048136	56125550	26403286	26496645	-	#6E0025	77414	93359	112414	-58980	112414	189828	false
HGL 9	56066570	56125553	19649394	19736779	+	#6E0025	58983	87385	-58980		-58980	3	false

	HGL 3	56135615	56204764	44827902	44943578	-	#1240C3	69149	115676	6394	-58278	6394	75543	false
	HGL 22	56141202	56215926	8737100	8796822	+	#00FFFF	74724	59722	11981	-70831	11981	86705	false
	HGL 22	56145095	56292424	4483149	4604501	-	#00FFFF	147329	121352	-70831	-60121	-70831	76498	false
	HGL 3	56146486	56298266	42993912	43118163	-	#1240C3	151780	124251	-58278	-112904	-58278	93502	false
	HGL 3	56185362	56294560	44633497	44708914	-	#1240C3	109198	75417	-112904	-41884	-112904	-3706	true
	HGL 22	56232303	56292502	4710705	4762097	+	#00FFFF	60199	51392	-60121	22437	-60121	78	false
	HGL 3	56252676	56360973	44742720	44869314	+	#1240C3	108297	126594	-41884	-46034	-41884	66413	false
	HGL 3	56314939	56379858	44878280	44952665	+	#1240C3	64919	74385	-46034	91448	-46034	18885	false
	HGL 22	56314939	56701362	8306696	8694683	-	#00FFFF	386423	387987	22437	-4723	22437	408860	false
	HGL 3	56471306	56603008	44963314	45039275	+	#1240C3	131702	75961	91448		91448	223150	false
	HGL 22	56696639	57241109	7732553	8265559	-	#00FFFF	544470	533006	-4723	53254	-4723	539747	false
	HGL 22	57294363	58412853	6948919	7711183	-	#00FFFF	1118490	762264	53254	27599	53254	1171744	false
	HGL 22	58440452	58761109	6118596	6315538	-	#00FFFF	320657	196942	27599	378933	27599	348256	false
	HGL 22	59140042	59876897	5429740	5958529	-	#00FFFF	736855	528789	378933	52453	378933	1115788	false
	HGL 22	59929350	60245111	5114684	5398602	-	#00FFFF	315761	283918	52453	-9110	52453	368214	false
	HGL 22	60236001	60345052	4964000	5050137	+	#00FFFF	109051	86137	-9110	17200	-9110	99941	false
	HGL 22	60362252	60703625	4215797	4408356	-	#00FFFF	341373	192559	17200	59660	17200	358573	false
	HGL 22	60763285	61212656	4022713	4199616	-	#00FFFF	449371	176903	59660	45914	59660	509031	false
	HGL 22	61258570	62050038	3516258	3988646	+	#00FFFF	791468	472388	45914	239884	45914	837382	false
	HGL 22	62289922	63025523	3031473	3491356	-	#00FFFF	735601	459883	239884	548726	239884	975485	false
	HGL 22	63574249	65138293	31873370	33245877	-	#00FFFF	1564044	1372507	548726	31825	548726	2112770	false
	HGL 22	65170118	67586711	29564196	31792009	-	#00FFFF	2416593	2227813	31825	-47635	31825	2448418	false
	HGL 22	67539076	67678162	29503198	29564825	-	#00FFFF	139086	61627	-47635	314821	-47635	91451	false
	HGL 19	67785037	67898009	20846187	20918527	-	#0000FF	112972	72340	64192703	1376	64192703	64305675	false
	HGL 19	67899385	67966930	20755433	20824442	+	#0000FF	67545	69009	1376	3661413	1376	68921	false
	HGL 22	67992983	70955499	27046112	29473595	-	#00FFFF	2962516	2427483	314821	100163	314821	3277337	false
	HGL 22	71055662	71513052	26631793	27042752	-	#00FFFF	457390	410959	100163		100163	557553	false
	HGL 19	71628343	71696683	20755433	20824442	-	#0000FF	68340	69009	3661413	1383	3661413	3729753	false
	HGL 19	71698066	71804772	20846187	20915560	+	#0000FF	106706	69373	1383		1383	108089	false
	HGL 8	71915623	72485575	97503077	98015495	-	#6A78D4	569952	512418	22323123	29434	22323123	22893075	false
	HGL 8	72515009	76828869	93293858	97397275	-	#6A78D4	4313860	4103417	29434	515	29434	4343294	false
	HGL 8	76829384	76990674	92953471	93097336	-	#6A78D4	161290	143865	515	-42768	515	161805	false

	HGL 8	76947906	82547219	87509055	92953419	-	#6A78D4	5599313	5444364	-42768	0	-42768	5556545	false
	HGL 8	82547219	82605783	87378800	87446841	+	#6A78D4	58564	68041	0	28552	0	58564	false
	HGL 8	82634335	85460902	84471155	87350066	-	#6A78D4	2826567	2878911	28552	62244	28552	2855119	false
	HGL 8	85523146	88856240	81291878	84442418	-	#6A78D4	3333094	3150540	62244	175436	62244	3395338	false
	HGL 8	89031676	89324456	80847070	81110069	-	#6A78D4	292780	262999	175436	81364	175436	468216	false
	HGL 8	89405820	89614203	80574995	80760597	-	#6A78D4	208383	185602	81364	465902	81364	289747	false
	HGL 8	90080105	90290277	80199864	80351614	-	#6A78D4	210172	151750	465902	57363	465902	676074	false
	HGL 8	90347640	105298409	65556946	80190543	-	#6A78D4	14950769	14633597	57363	-347607	57363	15008132	false
	HGL 8	104950802	105140285	65781104	65875599	-	#6A78D4	189483	94495	-347607	158124	-347607	-158124	true
	HGL 8	105298409	105356021	65500242	65556946	+	#6A78D4	57612	56704	158124	3209	158124	215736	false
	HGL 8	105359230	112279534	58254964	65499269	-	#6A78D4	6920304	7244305	3209	0	3209	6923513	false
	HGL 8	112279534	114598421	55647127	58188715	-	#6A78D4	2318887	2541588	0	51515	0	2318887	false
	HGL 8	114649936	120457239	49871700	55639906	-	#6A78D4	5807303	5768206	51515	50362	51515	5858818	false
	HGL 8	120507601	124089933	43482882	47183986	+	#6A78D4	3582332	3701104	50362	-3390	50362	3632694	false
	HGL 8	124086543	124212581	47237261	47438185	+	#6A78D4	126038	200924	-3390	-5656	-3390	122648	false
	HGL 8	124206925	124299953	47772100	47878422	+	#6A78D4	93028	106322	-5656	-92816	-5656	87372	false
	HGL 8	124207137	124315282	47786230	47863798	+	#6A78D4	108145	77568	-92816	5247	-92816	15329	false
	HGL 8	124320529	124408095	47944369	48093836	+	#6A78D4	87566	149467	5247	-26700	5247	92813	false
	HGL 8	124381395	124551799	48365026	48617249	+	#6A78D4	170404	252223	-26700	-14869	-26700	143704	false
	HGL 8	124536930	125679809	48728278	49867478	+	#6A78D4	1142879	1139200	-14869	170686	-14869	1128010	false
	HGL 8	125850495	127721840	41505829	43449377	-	#6A78D4	1871345	1943548	170686	63248	170686	2042031	false
	HGL 8	127785088	134345146	34526248	41453612	-	#6A78D4	6560058	6927364	63248	-68323	63248	6623306	false
	HGL 8	134276823	134973894	33693586	34531033	-	#6A78D4	697071	837447	-68323		-68323	628748	false
**HSA 12**	HGL 12	12052	951892	90991008	91883611	-	#DD528A	939840	892603		181			
	HGL 12	952073	2745935	88187549	89966389	+	#DD528A	1793862	1778840	181		181	1794043	false
	HGL 8	2788219	7221607	29347630	33033506	-	#6A78D4	4433388	3685876		224573			
	HGL 8	7446180	7602483	29096543	29176983	-	#6A78D4	156303	80440	224573	45854	224573	380876	false
	HGL 8	7648337	7854536	28013440	28225969	-	#6A78D4	206199	212529	45854	28291	45854	252053	false
	HGL 8	7882827	8131501	27875452	28136906	-	#6A78D4	248674	261454	28291	-25669	28291	276965	false
	HGL 8	8105832	8157259	28946014	29012468	+	#6A78D4	51427	66454	-25669	294845	-25669	25758	false
	HGL 19	8226566	8332793	20846187	20916220	-	#0000FF	106227	70033		1386			
	HGL 19	8334179	8407894	20755433	20824442	+	#0000FF	73715	69009	1386	99462571	1386	75101	false

	HGL 8	8452104	8555864	28971263	29095059	+	#6A78D4	103760	123796	294845	16761	294845	398605	false
	HGL 8	8572625	8841248	28213221	28376295	+	#6A78D4	268623	163074	16761	58390	16761	285384	false
	HGL 8	8899638	9134785	28386666	28540486	+	#6A78D4	235147	153820	58390	23184	58390	293537	false
	HGL 8	9157969	9218313	28554941	28605510	-	#6A78D4	60344	50569	23184	261777	23184	83528	false
	HGL 8	9480090	9565913	28639315	28703254	-	#6A78D4	85823	63939	261777	154506	261777	347600	false
	HGL 8	9720419	9800349	28781069	28834796	+	#6A78D4	79930	53727	154506	237474	154506	234436	false
	HGL 8	10037823	10315710	26802818	27063161	+	#6A78D4	277887	260343	237474	70599	237474	515361	false
	HGL 8	10386309	10837834	27105035	27488816	+	#6A78D4	451525	383781	70599	44	70599	522124	false
	HGL 8	10837878	10919728	26659495	26754816	+	#6A78D4	81850	95321	44	18178	44	81894	false
	HGL 8	10937906	11249069	26319335	26431476	-	#6A78D4	311163	112141	18178	15863	18178	329341	false
	HGL 8	11264932	11398048	27715527	27766244	+	#6A78D4	133116	50717	15863	-44044	15863	148979	false
	HGL 8	11354004	14213249	23807825	26357762	-	#6A78D4	2859245	2549937	-44044	24399	-44044	2815201	false
	HGL 8	14237648	17764355	20523505	23735677	-	#6A78D4	3526707	3212172	24399	10046	24399	3551106	false
	HGL 8	17774401	17853926	20462965	20523505	+	#6A78D4	79525	60540	10046	7640	10046	89571	false
	HGL 8	17861566	20918563	17636502	20462965	-	#6A78D4	3056997	2826463	7640	-117256	7640	3064637	false
	HGL 8	20801307	20932695	17345547	17499293	+	#6A78D4	131388	153746	-117256	-1470	-117256	14132	false
	HGL 8	20931225	21120620	17338725	17501852	+	#6A78D4	189395	163127	-1470	-10590	-1470	187925	false
	HGL 8	21110030	21430338	17338725	17629514	+	#6A78D4	320308	290789	-10590	4291	-10590	309718	false
	HGL 8	21434629	27040704	12161006	17244671	-	#6A78D4	5606075	5083665	4291	3792248	4291	5610366	false
	HGL 16	27063978	27148407	55113545	55217667	+	#80E0F6	84429	104122		0			
	HGL 16	27148407	27336668	41861	203149	-	#80E0F6	188261	161288	0	178994	0	188261	false
	HGL 16	27515662	30282904	55046004	58343855	+	#80E0F6	2767242	3297851	178994	-716626	178994	2946236	false
	HGL 16	29566278	29745213	57990640	58212183	+	#80E0F6	178935	221543	-716626	171465	-716626	-537691	true
	HGL 16	29916678	30171176	57547039	57796150	+	#80E0F6	254498	249111	171465	18875	171465	425963	false
	HGL 16	30190051	30282142	57723421	57823239	-	#80E0F6	92091	99818	18875	98557	18875	110966	false
	HGL 16	30380699	30791643	58345506	58766329	+	#80E0F6	410944	420823	98557	457645	98557	509501	false
	HGL 8	30832952	31012395	32428716	32595624	+	#6A78D4	179443	166908	3792248	112365	3792248	3971691	false
	HGL 8	31124760	31211945	28639315	28715650	+	#6A78D4	87185	76335	112365		112365	199550	false
	HGL 16	31249288	31839479	58769153	59318261	+	#80E0F6	590191	549108	457645	116164	457645	1047836	false
	HGL 16	31955643	33999965	59532137	61600842	+	#80E0F6	2044322	2068705	116164	4213482	116164	2160486	false
	HGL 16	38213447	38294053	61522224	61600842	+	#80E0F6	80606	78618	4213482	73738	4213482	4294088	false
	HGL 16	38367791	38595414	61669319	61907445	+	#80E0F6	227623	238126	73738	48553	73738	301361	false

HGL 16	38643967	39464153	61962505	62731965	+	#80E0F6	820186	769460	48553	15543	48553	868739	false
HGL 16	39479696	40380565	62783409	63633415	+	#80E0F6	900869	850006	15543	-28630	15543	916412	false
HGL 16	40351935	42396093	63633415	65948255	+	#80E0F6	2044158	2314840	-28630	-82891	-28630	2015528	false
HGL 16	42313202	47443059	66035807	71531297	+	#80E0F6	5129857	5495490	-82891	15094	-82891	5046966	false
HGL 16	47458153	47510641	71531303	71582851	-	#80E0F6	52488	51548	15094	3903	15094	67582	false
HGL 16	47514544	48355589	71582851	72489131	+	#80E0F6	841045	906280	3903	77339	3903	844948	false
HGL 16	48432928	48527858	72607341	72716414	+	#80E0F6	94930	109073	77339	26528	77339	172269	false
HGL 16	48554386	51038041	72792483	75029112	+	#80E0F6	2483655	2236629	26528	-103574	26528	2510183	false
HGL 16	50934467	51041576	75033064	75130222	+	#80E0F6	107109	97158	-103574	3577	-103574	3535	false
HGL 16	51045153	52306320	75183216	76266632	+	#80E0F6	1261167	1083416	3577	-40041	3577	1264744	false
HGL 16	52266279	52336781	76265509	76344281	+	#80E0F6	70502	78772	-40041	761	-40041	30461	false
HGL 16	52337542	53299473	52892570	53766260	-	#80E0F6	961931	873690	761	0	761	962692	false
HGL 16	53299473	54682875	51512350	52816411	-	#80E0F6	1383402	1304061	0	3244	0	1383402	false
HGL 16	54686119	54765420	51355992	51428022	+	#80E0F6	79301	72030	3244	169317	3244	82545	false
HGL 16	54934737	55118610	51119079	51343016	-	#80E0F6	183873	223937	169317	24295	169317	353190	false
HGL 16	55142905	55300419	50849986	51032152	-	#80E0F6	157514	182166	24295	-52355	24295	181809	false
HGL 16	55248064	55453533	50457436	50683046	+	#80E0F6	205469	225610	-52355	213889	-52355	153114	false
HGL 16	55667422	56754592	48726847	49799908	-	#80E0F6	1087170	1073061	213889	18861	213889	1301059	false
HGL 16	56773453	63430883	42002076	48601754	-	#80E0F6	6657430	6599678	18861	348231	18861	6676291	false
HGL 3	63557835	63667932	10244871	10311044	+	#1240C3	110097	66173	55955352	-108713	55955352	56065449	false
HGL 3	63559219	63725474	7278761	7412071	+	#1240C3	166255	133310	-108713		-108713	57542	false
HGL 16	63779114	64885913	40934984	41976223	-	#80E0F6	1106799	1041239	348231	-45497	348231	1455030	false
HGL 16	64840416	68304293	37440209	40934983	-	#80E0F6	3463877	3494774	-45497	99662	-45497	3418380	false
HGL 16	68403955	71721530	34083792	37438808	-	#80E0F6	3317575	3355016	99662	23317	99662	3417237	false
HGL 16	71744847	100149294	7551526	34021885	-	#80E0F6	28404447	26470359	23317	51097	23317	28427764	false
HGL 16	100200391	101832717	6315188	7535820	-	#80E0F6	1632326	1220632	51097	55131	51097	1683423	false
HGL 16	101887848	105022980	4053545	6312730	-	#80E0F6	3135132	2259185	55131	-2243	55131	3190263	false
HGL 16	105020737	107786213	2063325	3963905	-	#80E0F6	2765476	1900580	-2243		-2243	2763233	false
HGL 19	107870465	109292561	38219971	39459681	+	#0000FF	1422096	1239710	99462571	-38926	99462571	100884667	false
HGL 19	109253635	110053321	37492442	38193821	-	#0000FF	799686	701379	-38926	6370	-38926	760760	false
HGL 19	110059691	114103569	45712983	48767345	-	#0000FF	4043878	3054362	6370	21861	6370	4050248	false
HGL 19	114125430	121051272	39699985	45567800	-	#0000FF	6925842	5867815	21861	53092	21861	6947703	false

	HGL 19	121104364	124605403	48744475	50949457	+	#0000FF	3501039	2204982	53092	152764	53092	3554131	false
	HGL 19	124758167	127290068	50994790	52496805	+	#0000FF	2531901	1502015	152764	-821369	152764	2684665	false
	HGL 19	126468699	126629274	51690945	51797046	+	#0000FF	160575	106101	-821369	145234	-821369	-660794	true
	HGL 19	126774508	126865843	51372690	51495538	+	#0000FF	91335	122848	145234	129097	145234	236569	false
	HGL 19	126994940	127053240	51743438	51793810	-	#0000FF	58300	50372	129097	227409	129097	187397	false
	HGL 19	127280649	127439656	51330697	51517500	-	#0000FF	159007	186803	227409	-93396	227409	386416	false
	HGL 19	127346260	128746456	52496989	53277197	+	#0000FF	1400196	780208	-93396	51935	-93396	1306800	false
	HGL 19	128798391	129994389	53288148	53962274	+	#0000FF	1195998	674126	51935	50687	51935	1247933	false
	HGL 19	130045076	130672866	53962291	54257975	+	#0000FF	627790	295684	50687	117774	50687	678477	false
	HGL 19	130790640	131139486	54266966	54442458	+	#0000FF	348846	175492	117774	134462	117774	466620	false
	HGL 19	131273948	131387724	54483182	54533440	+	#0000FF	113776	50258	134462	2892	134462	248238	false
	HGL 19	131390616	131534941	54433251	54484385	-	#0000FF	144325	51134	2892	2400	2892	147217	false
	HGL 19	131537341	131636297	54568008	54619671	+	#0000FF	98956	51663	2400	72554	2400	101356	false
	HGL 19	131708851	133200058	54620149	55477892	+	#0000FF	1491207	857743	72554		72554	1563761	false
**HSA 13**	HGL 15	19348575	19408886	1315004	1416584	+	#FAB28C	60311	101580		538			
	HGL 15	19409424	19529308	23291823	23423892	+	#FAB28C	119884	132069	538	2630	538	120422	false
	HGL 15	19531938	19604184	23216192	23291823	+	#FAB28C	72246	75631	2630	864	2630	74876	false
	HGL 15	19605048	21187939	1416896	3063281	+	#FAB28C	1582891	1646385	864	184621	864	1583755	false
	HGL 15	21372560	22297559	3084992	4121544	+	#FAB28C	924999	1036552	184621	68311	184621	1109620	false
	HGL 15	22365870	22911586	4156357	4721169	+	#FAB28C	545716	564812	68311	86750	68311	614027	false
	HGL 15	22998336	23895278	4757392	5603715	+	#FAB28C	896942	846323	86750	62554	86750	983692	false
	HGL 15	23957832	24316955	5603715	6031289	-	#FAB28C	359123	427574	62554	103229	62554	421677	false
	HGL 15	24420184	24525072	1270339	1416584	-	#FAB28C	104888	146245	103229	86873	103229	208117	false
	HGL 15	24611945	24931784	901231	1306553	+	#FAB28C	319839	405322	86873	123773	86873	406712	false
	HGL 15	25055557	26097513	5955254	7057969	+	#FAB28C	1041956	1102715	123773	80697	123773	1165729	false
	HGL 15	26178210	38889063	7075876	21375558	+	#FAB28C	12710853	14299682	80697	64762	80697	12791550	false
	HGL 15	38953825	39259361	21375558	21609904	+	#FAB28C	305536	234346	64762	303	64762	370298	false
	HGL 15	39259664	40871557	21674253	23355007	+	#FAB28C	1611893	1680754	303	34931	303	1612196	false
	HGL 15	40906488	41145909	35520898	35733524	-	#FAB28C	239421	212626	34931	32415	34931	274352	false
	HGL 15	41178324	45376501	31240860	35542976	-	#FAB28C	4198177	4302116	32415	8304	32415	4230592	false
	HGL 15	45384805	45456302	31185976	31240839	+	#FAB28C	71497	54863	8304	8081	8304	79801	false
	HGL 15	45464383	46407147	30218270	31185912	-	#FAB28C	942764	967642	8081	61261	8081	950845	false

	HGL 15	46468408	48629260	27824096	30208111	-	#FAB28C	2160852	2384015	61261	1425	61261	2222113	false
	HGL 15	48630685	48801023	27614229	27790240	+	#FAB28C	170338	176011	1425	13	1425	171763	false
	HGL 15	48801036	52160021	23563544	27601516	-	#FAB28C	3358985	4037972	13	15487	13	3358998	false
	HGL 15	52175508	52476470	23226380	23554390	+	#FAB28C	300962	328010	15487	126	15487	316449	false
	HGL 15	52476596	52637575	23226380	23449144	-	#FAB28C	160979	222764	126	14401	126	161105	false
	HGL 15	52651976	54686818	35718034	37893729	+	#FAB28C	2034842	2175695	14401	22583	14401	2049243	false
	HGL 15	54709401	54788571	37894412	37968809	-	#FAB28C	79170	74397	22583	-38413	22583	101753	false
	HGL 15	54750158	55495495	37928333	38587298	+	#FAB28C	745337	658965	-38413	307	-38413	706924	false
	HGL 15	55495802	55709941	38587299	38804702	-	#FAB28C	214139	217403	307	1261	307	214446	false
	HGL 15	55711202	61808601	38804992	44628816	+	#FAB28C	6097399	5823824	1261	11729	1261	6098660	false
	HGL 15	61820330	76109609	44683639	59126647	+	#FAB28C	14289279	14443008	11729	18409	11729	14301008	false
	HGL 15	76128018	76266715	59126963	59275694	-	#FAB28C	138697	148731	18409	2033	18409	157106	false
	HGL 15	76268748	86202257	59277241	69210405	+	#FAB28C	9933509	9933164	2033	78747	2033	9935542	false
	HGL 15	86281004	97858625	69212050	80143448	+	#FAB28C	11577621	10931398	78747	19913	78747	11656368	false
	HGL 15	97878538	100682492	80260497	82789388	+	#FAB28C	2803954	2528891	19913	0	19913	2823867	false
	HGL 15	100682492	100755349	82790449	82851285	-	#FAB28C	72857	60836	0	2035	0	72857	false
	HGL 15	100757384	110562440	82851320	91190433	+	#FAB28C	9805056	8339113	2035	52823	2035	9807091	false
	HGL 15	110615263	110763766	91204057	91294809	+	#FAB28C	148503	90752	52823	113982	52823	201326	false
	HGL 15	110877748	111009431	91296790	91356890	+	#FAB28C	131683	60100	113982	96430	113982	245665	false
	HGL 15	111105861	111668893	91364792	91723699	+	#FAB28C	563032	358907	96430	174523	96430	659462	false
	HGL 15	111843416	112192219	91853565	92085756	+	#FAB28C	348803	232191	174523	63194	174523	523326	false
	HGL 15	112255413	113671678	92130703	92910508	+	#FAB28C	1416265	779805	63194	51390	63194	1479459	false
	HGL 15	113723068	114328985	92910721	93260380	+	#FAB28C	605917	349659	51390		51390	657307	false
**HSA 14**	HGL 2	19709591	19953548	126253605	126483575	-	#C008AD	243957	229970		-47793			
	HGL 2	19905755	19977213	126163978	126237691	-	#C008AD	71458	73713	-47793	-53932	-47793	23665	false
	HGL 2	19923281	20135883	125973183	126144407	-	#C008AD	212602	171224	-53932	-84876	-53932	158670	false
	HGL 2	20051007	20139690	125863310	125969222	-	#C008AD	88683	105912	-84876	-52632	-84876	3807	false
	HGL 2	20087058	20163476	125745661	125797828	-	#C008AD	76418	52167	-52632	26594	-52632	23786	false
	HGL 2	20190070	20969066	126910456	127546242	+	#C008AD	778996	635786	26594	-84591	26594	805590	false
	HGL 2	20884475	21816120	124134037	125001889	-	#C008AD	931645	867852	-84591	-31574	-84591	847054	false
	HGL 2	21784546	22101886	123683876	123822715	-	#C008AD	317340	138839	-31574	-311151	-31574	285766	false
	HGL 2	21790735	22346026	123156342	123612931	-	#C008AD	555291	456589	-311151	-401348	-311151	244140	false

	HGL 2	21944678	22228832	123838947	124009197	-	#C008AD	284154	170250	-401348	118055	-401348	-117194	true
	HGL 2	22346887	22702330	122741511	123103122	-	#C008AD	355443	361611	118055	43979	118055	473498	false
	HGL 2	22746309	23786195	121613247	122680125	-	#C008AD	1039886	1066878	43979	30308	43979	1083865	false
	HGL 2	23816503	23906144	121483094	121545751	-	#C008AD	89641	62657	30308	-7105	30308	119949	false
	HGL 2	23899039	24252143	121194288	121443603	-	#C008AD	353104	249315	-7105	-55938	-7105	345999	false
	HGL 2	24196205	27002809	118269859	121193923	-	#C008AD	2806604	2924064	-55938	618	-55938	2750666	false
	HGL 2	27003427	27094932	118144029	118263116	+	#C008AD	91505	119087	618	5341	618	92123	false
	HGL 2	27100273	33858751	111446041	118135951	-	#C008AD	6758478	6689910	5341	816	5341	6763819	false
	HGL 2	33859567	36809150	108501838	111387617	-	#C008AD	2949583	2885779	816	499513	816	2950399	false
	HGL 2	37308663	38187409	107597306	108500330	-	#C008AD	878746	903024	499513	80	499513	1378259	false
	HGL 2	38187489	38958253	106619386	107493705	-	#C008AD	770764	874319	80	25747	80	770844	false
	HGL 2	38984000	42334522	103459736	106568913	-	#C008AD	3350522	3109177	25747	-112050	25747	3376269	false
	HGL 2	42222472	42338219	74548783	74658630	+	#C008AD	115747	109847	-112050	80978	-112050	3697	false
	HGL 2	42419197	55209328	91484515	103366701	-	#C008AD	12790131	11882186	80978		80978	12871109	false
	HGL 28	55266489	60191475	41011696	46254903	-	#007FFF	4924986	5243207		-25482			
	HGL 28	60165993	63560183	37498117	40919934	-	#007FFF	3394190	3421817	-25482	29708	-25482	3368708	false
	HGL 28	63589891	64354672	36669783	37443978	-	#007FFF	764781	774195	29708	-30213	29708	794489	false
	HGL 28	64324459	66692778	34347325	36669782	-	#007FFF	2368319	2322457	-30213	61484	-30213	2338106	false
	HGL 28	66754262	70423657	30721333	34333543	-	#007FFF	3669395	3612210	61484	157514	61484	3730879	false
	HGL 28	70581171	91998737	10721060	30622253	-	#007FFF	21417566	19901193	157514	-42932	157514	21575080	false
	HGL 28	91955805	94382816	8595208	10708158	+	#007FFF	2427011	2112950	-42932	-15682	-42932	2384079	false
	HGL 28	94367134	94569619	7103070	7267271	+	#007FFF	202485	164201	-15682	5313	-15682	186803	false
	HGL 28	94574932	96267779	7231613	8551991	+	#007FFF	1692847	1320378	5313	11672	5313	1698160	false
	HGL 28	96279451	100642507	3546032	7102435	-	#007FFF	4363056	3556403	11672	0	11672	4374728	false
	HGL 28	100642507	103157355	1544002	3446247	-	#007FFF	2514848	1902245	0	165028	0	2514848	false
	HGL 28	103322383	104449762	733719	1520731	-	#007FFF	1127379	787012	165028	57283	165028	1292407	false
	HGL 28	104507045	105531522	25874	688448	-	#007FFF	1024477	662574	57283		57283	1081760	false
	HGL 26	105576085	105670149	29029212	29116962	+	#7FBF7F	94064	87750		-50712			
	HGL 26	105619437	105776869	29023213	29123456	+	#7FBF7F	157432	100243	-50712	-34494	-50712	106720	false
	HGL 26	105742375	106014601	29115955	29311730	+	#7FBF7F	272226	195775	-34494		-34494	237732	false
**HSA 15**	HGL 9	20429206	20507549	91551686	91664173	+	#6E0025	78343	112487		1985952			
	HGL 2	21550198	21685703	126376342	126521994	-	#C008AD	135505	145652		301788			

	HGL 2	21987491	22128173	126376342	126521994	-	#C008AD	140682	145652	301788	10481099	301788	442470	false
	HGL 9	22493501	22575114	91549006	91664173	-	#6E0025	81613	115167	1985952	210063	1985952	2067565	false
	HGL 9	22785177	23042601	91664286	91885732	+	#6E0025	257424	221446	210063	510100	210063	467487	false
	HGL 9	23552701	23925966	88245018	88626104	+	#6E0025	373265	381086	510100	171858	510100	883365	false
	HGL 9	24097824	24171486	88536622	88626172	+	#6E0025	73662	89550	171858	217495	171858	245520	false
	HGL 9	24388981	24493230	88525055	88641579	+	#6E0025	104249	116524	217495	352863	217495	321744	false
	HGL 9	24846093	25167128	88754582	89050213	+	#6E0025	321035	295631	352863	97540	352863	673898	false
	HGL 9	25264668	25557391	89022961	89344924	+	#6E0025	292723	321963	97540	66034	97540	390263	false
	HGL 9	25623425	28323528	89345524	91664173	+	#6E0025	2700103	2318649	66034	264566	66034	2766137	false
	HGL 9	28588094	28669396	91549006	91664173	-	#6E0025	81302	115167	264566	191609	264566	345868	false
	HGL 9	28861005	30076423	85817212	86920042	-	#6E0025	1215418	1102830	191609	200806	191609	1407027	false
	HGL 9	30277229	30376921	88022110	88099224	-	#6E0025	99692	77114	200806	318301	200806	300498	false
	HGL 9	30695222	30762387	87581218	87657014	-	#6E0025	67165	75796	318301	137036	318301	385466	false
	HGL 9	30899423	32261428	86971101	88099224	+	#6E0025	1362005	1128123	137036	46603185	137036	1499041	false
	HGL 2	32609272	34374869	127767291	129540402	-	#C008AD	1765597	1773111	10481099	265549	10481099	12246696	false
	HGL 2	34640418	43657405	129593950	138554718	+	#C008AD	9016987	8960768	265549	-35736	265549	9282536	false
	HGL 2	43621669	43718537	107493735	107595246	+	#C008AD	96868	101511	-35736	-60046	-35736	61132	false
	HGL 2	43658491	44205780	138451110	139047688	+	#C008AD	547289	596578	-60046	30716	-60046	487243	false
	HGL 2	44236496	44734993	139296973	139843863	-	#C008AD	498497	546890	30716	82743	30716	529213	false
	HGL 2	44817736	44918792	91372278	91428152	+	#C008AD	101056	55874	82743	37207	82743	183799	false
	HGL 2	44955999	46155444	90529387	91443037	-	#C008AD	1199445	913650	37207	53744	37207	1236652	false
	HGL 2	46209188	54713540	83800282	90528912	-	#C008AD	8504352	6728630	53744	61545	53744	8558096	false
	HGL 2	54775085	55108971	83601897	83799670	-	#C008AD	333886	197773	61545	53651	61545	395431	false
	HGL 2	55162622	56254727	82903262	83598939	-	#C008AD	1092105	695677	53651	45612238	53651	1145756	false
	HGL 26	56357668	62240950	10557474	15839137	+	#7FBF7F	5883282	5281663	35928746	63108	35928746	41812028	false
	HGL 26	62304058	64954222	15844350	18215376	+	#7FBF7F	2650164	2371026	63108	4067	63108	2713272	false
	HGL 26	64958289	72378933	18368340	24258440	+	#7FBF7F	7420644	5890100	4067	75525	4067	7424711	false
	HGL 26	72454458	72600528	24259149	24383960	+	#7FBF7F	146070	124811	75525	92375	75525	221595	false
	HGL 26	72692903	74051581	24404113	25482318	+	#7FBF7F	1358678	1078205	92375	68807	92375	1451053	false
	HGL 26	74120388	74679001	25482318	25945110	+	#7FBF7F	558613	462792	68807	83259	68807	627420	false
	HGL 26	74762260	75051177	25945517	26151537	+	#7FBF7F	288917	206020	83259	100054	83259	372176	false
	HGL 26	75151231	75251562	26160995	26261363	+	#7FBF7F	100331	100368	100054	84394	100054	200385	false

	HGL 26	75335956	75736397	26221513	26559970	+	#7FBF7F	400441	338457	84394	61682	84394	484835	false
	HGL 26	75798079	77895321	26524458	28219386	-	#7FBF7F	2097242	1694928	61682	72443	61682	2158924	false
	HGL 26	77967764	78713719	28211297	28751990	-	#7FBF7F	745955	540693	72443	44000	72443	818398	false
	HGL 26	78757719	78813026	28727593	28779863	+	#7FBF7F	55307	52270	44000	5710678	44000	99307	false
	HGL 9	78864613	79341278	66346946	66732423	+	#6E0025	476665	385477	46603185	46872	46603185	47079850	false
	HGL 9	79388150	79907354	66786864	67219622	+	#6E0025	519204	432758	46872	44665	46872	566076	false
	HGL 9	79952019	80319984	67203711	67475606	+	#6E0025	367965	271895	44665	5764	44665	412630	false
	HGL 9	80325748	81589007	67588095	68571596	+	#6E0025	1263259	983501	5764	69435	5764	1269023	false
	HGL 9	81658442	82277023	68643087	69228848	+	#6E0025	618581	585761	69435	259544	69435	688016	false
	HGL 9	82536567	84150119	69138128	70561144	-	#6E0025	1613552	1423016	259544	448115	259544	1873096	false
	HGL 9	84598234	85156765	70563480	71091849	-	#6E0025	558531	528369	448115	129658	448115	1006646	false
	HGL 9	85286423	90273577	71699277	76271971	-	#6E0025	4987154	4572694	129658	41609	129658	5116812	false
	HGL 9	90315186	91025013	71091889	71698491	+	#6E0025	709827	606602	41609	23544	41609	751436	false
	HGL 9	91048557	92722307	76262255	77935332	+	#6E0025	1673750	1673077	23544	58302	23544	1697294	false
	HGL 9	92780609	98536051	77939463	83213998	+	#6E0025	5755442	5274535	58302	1094	58302	5813744	false
	HGL 9	98537145	99023290	83213998	83641006	-	#6E0025	486145	427008	1094	943	1094	487239	false
	HGL 9	99024233	99754865	83641017	84257615	+	#6E0025	730632	616598	943	166493	943	731575	false
	HGL 9	99921358	101732197	84264729	85817212	+	#6E0025	1810839	1552483	166493		166493	1977332	false
**HSA 16**	HGL 19	28451	1482530	24694771	25964115	+	#0000FF	1454079	1269344		82			
	HGL 19	1482612	2224068	23821072	24528907	-	#0000FF	741456	707835	82	-3715	82	741538	false
	HGL 19	2220353	2543771	23485807	23750210	-	#0000FF	323418	264403	-3715	190303	-3715	319703	false
	HGL 19	2734074	2838023	23145574	23277513	+	#0000FF	103949	131939	190303	44704	190303	294252	false
	HGL 19	2882727	4757348	21259241	23143473	-	#0000FF	1874621	1884232	44704	11278	44704	1919325	false
	HGL 19	4768626	10302309	16178792	21157891	-	#0000FF	5533683	4979099	11278	83305	11278	5544961	false
	HGL 19	10385614	11432673	15218726	16003311	-	#0000FF	1047059	784585	83305	100829	83305	1130364	false
	HGL 19	11533502	14733677	3870841	6750925	-	#0000FF	3200175	2880084	100829	103634	100829	3301004	false
	HGL 19	14837311	14910034	647584	706393	+	#0000FF	72723	58809	103634	44770	103634	176357	false
	HGL 19	14954804	15100600	15079807	15208103	+	#0000FF	145796	128296	44770	133528	44770	190566	false
	HGL 19	15234128	15345692	717529	793422	-	#0000FF	111564	75893	133528	79225	133528	245092	false
	HGL 19	15424917	16308689	62344	706393	+	#0000FF	883772	644049	79225	107860	79225	962997	false
	HGL 19	16416549	16571296	724547	856797	+	#0000FF	154747	132250	107860	192002	107860	262607	false
	HGL 19	16763298	18078901	904657	2073252	-	#0000FF	1315603	1168595	192002	60921	192002	1507605	false

	HGL 19	18139822	18295330	724547	857783	-	#0000FF	155508	133236	60921	188684	60921	216429	false
	HGL 19	18484014	18558886	647584	709434	-	#0000FF	74872	61850	188684	224089	188684	263556	false
	HGL 19	18782975	18865664	2231289	2300422	+	#0000FF	82689	69133	224089	116700	224089	306778	false
	HGL 19	18982364	19381928	2423672	2741378	-	#0000FF	399564	317706	116700	3520	116700	516264	false
	HGL 19	19385448	19895571	2758926	3190323	+	#0000FF	510123	431397	3520	1496	3520	513643	false
	HGL 19	19897067	20501184	3255662	3770886	+	#0000FF	604117	515224	1496	192	1496	605613	false
	HGL 19	20501376	20607539	3710315	3792652	-	#0000FF	106163	82337	192	8719	192	106355	false
	HGL 19	20616258	20705082	3792105	3861332	-	#0000FF	88824	69227	8719	4170	8719	97543	false
	HGL 19	20709252	21333905	6867172	7361035	+	#0000FF	624653	493863	4170	224972	4170	628823	false
	HGL 19	21558877	21796206	7766375	7942161	+	#0000FF	237329	175786	224972	148982	224972	462301	false
	HGL 19	21945188	22419424	7370003	7766321	+	#0000FF	474236	396318	148982	142388	148982	623218	false
	HGL 19	22561812	25060377	7904485	9997617	+	#0000FF	2498565	2093132	142388	26953	142388	2640953	false
	HGL 19	25087330	28330677	9948483	12779870	+	#0000FF	3243347	2831387	26953	146496	26953	3270300	false
	HGL 19	28477173	28614895	12994933	13103527	+	#0000FF	137722	108594	146496	205760	146496	284218	false
	HGL 19	28820655	29032722	12780135	12938777	-	#0000FF	212067	158642	205760	610697	205760	417827	false
	HGL 19	29643419	30205408	13070247	13500612	-	#0000FF	561989	430365	610697	142932	610697	1172686	false
	HGL 19	30348340	31531343	13508382	14508330	+	#0000FF	1183003	999948	142932		142932	1325935	false
	HGL 6	46578990	46849686	84590879	84867067	+	#60FA36	270696	276188		-19131			
	HGL 6	46830555	48166050	84897943	86378941	+	#60FA36	1335495	1480998	-19131	1243	-19131	1316364	false
	HGL 6	48167293	48227957	86284872	86344563	+	#60FA36	60664	59691	1243	-17535	1243	61907	false
	HGL 6	48210422	48615880	86549181	86929461	+	#60FA36	405458	380280	-17535		-17535	387923	false
	HGL 7	48639697	53328031	32741245	37355642	-	#C2FB8F	4688334	4614397	19366452	99128	19366452	24054786	false
	HGL 7	53427159	55718936	30650304	32727887	-	#C2FB8F	2291777	2077583	99128	272	99128	2390905	false
	HGL 7	55719208	55797545	30535239	30590426	+	#C2FB8F	78337	55187	272	-71852	272	78609	false
	HGL 7	55725693	55843823	30391053	30484402	-	#C2FB8F	118130	93349	-71852	-114294	-71852	46278	false
	HGL 7	55729529	55844402	30360864	30470582	+	#C2FB8F	114873	109718	-114294	-12422	-114294	579	false
	HGL 7	55831980	56705298	29345308	30095788	-	#C2FB8F	873318	750480	-12422	-16369	-12422	860896	false
	HGL 7	56688929	63133132	23340779	29299389	-	#C2FB8F	6444203	5958610	-16369	-27175	-16369	6427834	false
	HGL 7	63105957	66945546	19558150	23340678	-	#C2FB8F	3839589	3782528	-27175	-11017	-27175	3812414	false
	HGL 7	66934529	68010163	18296443	19382114	-	#C2FB8F	1075634	1085671	-11017	-5055	-11017	1064617	false
	HGL 7	68005108	69943578	16498149	18282099	-	#C2FB8F	1938470	1783950	-5055	168975	-5055	1933415	false
	HGL 7	70112553	70168140	12411877	12494255	-	#C2FB8F	55587	82378	168975	82841	168975	224562	false

	HGL 7	70250981	70819448	12538667	13030726	+	#C2FB8F	568467	492059	82841	77464	82841	651308	false
	HGL 7	70896912	74328227	13066555	16498097	+	#C2FB8F	3431315	3431542	77464	102085	77464	3508779	false
	HGL 7	74430312	81964749	5677839	12413351	-	#C2FB8F	7534437	6735512	102085	-80321	102085	7636522	false
	HGL 7	81884428	84329834	3299151	5623131	-	#C2FB8F	2445406	2323980	-80321	24518	-80321	2365085	false
	HGL 7	84354352	87770398	1392198	3285807	+	#C2FB8F	3416046	1893609	24518	233	24518	3440564	false
	HGL 7	87770631	88357389	1086047	1351991	-	#C2FB8F	586758	265944	233	51323	233	586991	false
	HGL 7	88408712	89203087	597904	1062843	-	#C2FB8F	794375	464939	51323	56358	51323	845698	false
	HGL 7	89259445	90049507	25963	583835	-	#C2FB8F	790062	557872	56358		56358	846420	false
**HSA 17**	HGL 18	142967	1335204	25408545	26503212	-	#00FF7F	1192237	1094667		2763			
	HGL 18	1337967	3142056	26518977	28107464	+	#00FF7F	1804089	1588487	2763	165150	2763	1806852	false
	HGL 18	3307206	3394714	28375154	28469489	-	#00FF7F	87508	94335	165150	2502	165150	252658	false
	HGL 18	3397216	4628885	28523035	29656484	+	#00FF7F	1231669	1133449	2502	2763	2502	1234171	false
	HGL 18	4631648	5115377	30949459	31388085	+	#00FF7F	483729	438626	2763	64191	2763	486492	false
	HGL 18	5179568	5585648	31397874	31800799	+	#00FF7F	406080	402925	64191	-55883	64191	470271	false
	HGL 18	5529765	6845290	29652181	30934925	-	#00FF7F	1315525	1282744	-55883	72731	-55883	1259642	false
	HGL 18	6918021	12181613	31916791	37046903	+	#00FF7F	5263592	5130112	72731	9229	72731	5336323	false
	HGL 18	12190842	15708963	38198099	41936591	-	#00FF7F	3518121	3738492	9229	117124	9229	3527350	false
	HGL 18	15826087	16654105	37340530	38156993	+	#00FF7F	828018	816463	117124	198345	117124	945142	false
	HGL 14	16704978	16771441	104872	169431	+	#98C405	66463	64559		1772809			
	HGL 10	16711372	16771441	62371174	62428172	+	#00007F	60069	56998		1772809			
	HGL 18	16852450	16917780	44528531	44595354	+	#00FF7F	65330	66823	198345	1397	198345	263675	false
	HGL 18	16919177	18364610	43256162	44682646	-	#00FF7F	1445433	1426484	1397	327337	1397	1446830	false
	HGL 10	18544250	18604046	62371174	62428172	-	#00007F	59796	56998	1772809	1738005	1772809	1832605	false
	HGL 14	18544250	18610464	104872	169431	-	#98C405	66214	64559	1772809	1725195	1772809	1839023	false
	HGL 18	18691947	18823588	38222357	38376017	+	#00FF7F	131641	153660	327337	33058	327337	458978	false
	HGL 18	18856646	19072871	45450858	45642805	+	#00FF7F	216225	191947	33058	121272	33058	249283	false
	HGL 18	19194143	19755354	45634181	46145152	+	#00FF7F	561211	510971	121272	13109	121272	682483	false
	HGL 18	19768463	19998433	46358389	46610302	-	#00FF7F	229970	251913	13109	13389	13109	243079	false
	HGL 18	20011822	20319402	37090852	37475474	+	#00FF7F	307580	384622	13389	392116	13389	320969	false
	HGL 14	20335659	20399985	104872	169431	+	#98C405	64326	64559	1725195		1725195	1789521	false
	HGL 10	20342051	20399985	62371174	62428172	+	#00007F	57934	56998	1738005		1738005	1795939	false
	HGL 18	20711518	20830787	14669096	14755571	+	#00FF7F	119269	86475	392116	136647	392116	511385	false

	HGL 18	20967434	21046646	44619928	44712580	+	#00FF7F	79212	92652	136647	52435	136647	215859	false
	HGL 18	21099081	21496615	45000664	45352468	+	#00FF7F	397534	351804	52435	5734391	52435	449969	false
	HGL 18	27231006	27651756	22452314	22813801	+	#00FF7F	420750	361487	5734391	87987	5734391	6155141	false
	HGL 18	27739743	28197156	22916859	23347624	+	#00FF7F	457413	430765	87987	115154	87987	545400	false
	HGL 18	28312310	30529600	23347624	25459762	+	#00FF7F	2217290	2112138	115154	201628	115154	2332444	false
	HGL 18	30731228	30828689	21446489	21553684	-	#00FF7F	97461	107195	201628	114	201628	299089	false
	HGL 18	30828803	31005961	21171619	21312943	-	#00FF7F	177158	141324	114	45008	114	177272	false
	HGL 18	31050969	32001249	21500768	22377606	-	#00FF7F	950280	876838	45008	132605	45008	995288	false
	HGL 18	32133854	35261219	18356352	21171506	-	#00FF7F	3127365	2815154	132605	270060	132605	3259970	false
	HGL 18	35531279	35901807	18051558	18356352	-	#00FF7F	370528	304794	270060	66999	270060	640588	false
	HGL 18	35968806	36151860	17844494	18045921	-	#00FF7F	183054	201427	66999	328393	66999	250053	false
	HGL 18	36480253	37843052	16556228	17770414	+	#00FF7F	1362799	1214186	328393	402315	328393	1691192	false
	HGL 18	38245367	39001042	3794333	4280515	-	#00FF7F	755675	486182	402315	60532	402315	1157990	false
	HGL 18	39061574	43229525	1133766	3794202	-	#00FF7F	4167951	2660436	60532	247596	60532	4228483	false
	HGL 18	43477121	44564169	573272	1131018	-	#00FF7F	1087048	557746	247596	82246	247596	1334644	false
	HGL 18	44646415	45492153	36474	556250	-	#00FF7F	845738	519776	82246	140617	82246	927984	false
	HGL 18	45632770	46241243	42257135	42824755	-	#00FF7F	608473	567620	140617	148180	140617	749090	false
	HGL 18	46389423	46489133	42854928	42966496	+	#00FF7F	99710	111568	148180	117911	148180	247890	false
	HGL 18	46607044	47003557	42854928	43244817	+	#00FF7F	396513	389889	117911	-68952	117911	514424	false
	HGL 18	46934605	47054580	58457243	58538782	-	#00FF7F	119975	81539	-68952	35817	-68952	51023	false
	HGL 18	47090397	47346915	42025512	42306702	-	#00FF7F	256518	281190	35817	133827	35817	292335	false
	HGL 18	47480742	47542756	4214624	4280515	+	#00FF7F	62014	65891	133827	53399	133827	195841	false
	HGL 18	47596155	58693054	4317654	13430814	+	#00FF7F	11096899	9113160	53399	-145937	53399	11150298	false
	HGL 18	58547117	59969737	13548702	14708736	+	#00FF7F	1422620	1160034	-145937	63525	-145937	1276683	false
	HGL 18	60033262	60109361	17770669	17844878	+	#00FF7F	76099	74209	63525	27958	63525	139624	false
	HGL 18	60137319	62252028	14669096	16474843	-	#00FF7F	2114709	1805747	27958	103738	27958	2142667	false
	HGL 18	62355766	62449551	46211804	46336726	-	#00FF7F	93785	124922	103738	27978	103738	197523	false
	HGL 18	62477529	64758892	46633256	48863076	+	#00FF7F	2281363	2229820	27978	12231	27978	2309341	false
	HGL 18	64771123	64838567	36474	97968	-	#00FF7F	67444	61494	12231	130855	12231	79675	false
	HGL 18	64969422	68113639	48757647	51764346	-	#00FF7F	3144217	3006699	130855	117997	130855	3275072	false
	HGL 18	68231636	73210521	51744151	56287666	+	#00FF7F	4978885	4543515	117997	-19859	117997	5096882	false
	HGL 18	73190662	74588684	56318882	57568967	+	#00FF7F	1398022	1250085	-19859	16625	-19859	1378163	false

	HGL 18	74605309	75579105	57527058	58411815	+	#00FF7F	973796	884757	16625	36	16625	990421	false
	HGL 18	75579141	80259880	58538798	62362911	+	#00FF7F	4680739	3824113	36	173	36	4680775	false
	HGL 18	80260053	81735207	62513911	63716389	+	#00FF7F	1475154	1202478	173	65685	173	1475327	false
	HGL 18	81800892	83231424	63716405	64827220	+	#00FF7F	1430532	1110815	65685		65685	1496217	false
**HSA 18**	HGL 23	112548	1147557	8879318	9774911	-	#D284F9	1035009	895593		11400			
	HGL 23	1158957	1360777	8689501	8879318	+	#D284F9	201820	189817	11400	3985	11400	213220	false
	HGL 23	1364762	10252909	1911896	8686726	-	#D284F9	8888147	6774830	3985	11331	3985	8892132	false
	HGL 23	10264240	10405741	1770029	1901223	+	#D284F9	141501	131194	11331	25833	11331	152832	false
	HGL 23	10431574	10555928	1670324	1769691	-	#D284F9	124354	99367	25833	107544	25833	150187	false
	HGL 23	10663472	11562202	1136101	1669966	-	#D284F9	898730	533865	107544	116481	107544	1006274	false
	HGL 23	11678683	11911428	981924	1136032	-	#D284F9	232745	154108	116481	335699	116481	349226	false
	HGL 23	12247127	12727412	693601	943544	-	#D284F9	480285	249943	335699	57244	335699	815984	false
	HGL 23	12784656	13919461	18919	693401	-	#D284F9	1134805	674482	57244	7031950	57244	1192049	false
	HGL 23	20951411	29931017	9768319	17713002	+	#D284F9	8979606	7944683	7031950	2670	7031950	16011556	false
	HGL 23	29933687	30085409	17716108	17861722	-	#D284F9	151722	145614	2670	348	2670	154392	false
	HGL 23	30085757	31698626	17879700	19349562	+	#D284F9	1612869	1469862	348	51882	348	1613217	false
	HGL 23	31750508	39763125	19355922	26901292	+	#D284F9	8012617	7545370	51882	55512	51882	8064499	false
	HGL 23	39818637	44324993	26942892	31481146	+	#D284F9	4506356	4538254	55512	56404	55512	4561868	false
	HGL 23	44381397	46959525	31507297	33960381	+	#D284F9	2578128	2453084	56404	77217	56404	2634532	false
	HGL 23	47036742	49494341	33971218	36392546	+	#D284F9	2457599	2421328	77217	15855	77217	2534816	false
	HGL 23	49510196	55971772	36457267	43049580	+	#D284F9	6461576	6592313	15855	-7966	15855	6477431	false
	HGL 23	55963806	66364848	43050203	54113354	+	#D284F9	10401042	11063151	-7966	42091	-7966	10393076	false
	HGL 23	66406939	68945169	54168883	57103094	+	#D284F9	2538230	2934211	42091	52777	42091	2580321	false
	HGL 23	68997946	80046988	57103256	69220696	+	#D284F9	11049042	12117440	52777	17948	52777	11101819	false
	HGL 23	80064936	80254828	69277714	69467108	+	#D284F9	189892	189394	17948		17948	207840	false
**HSA 19**	HGL 29	278727	2438843	9018694	10558609	-	#FF00FF	2160116	1539915		-22608			
	HGL 29	2416235	2814775	8122799	8368935	+	#FF00FF	398540	246136	-22608	160324	-22608	375932	false
	HGL 29	2975099	3501652	8368935	8659358	-	#FF00FF	526553	290423	160324	887	160324	686877	false
	HGL 29	3502539	3956663	8727811	9011488	+	#FF00FF	454124	283677	887	538	887	455011	false
	HGL 29	3957201	5874268	6846307	8122192	-	#FF00FF	1917067	1275885	538	-43856	538	1917605	false
	HGL 29	5830412	6337718	6515549	6842391	-	#FF00FF	507306	326842	-43856	14986	-43856	463450	false
	HGL 29	6352704	6605837	5985972	6151232	+	#FF00FF	253133	165260	14986	-59105	14986	268119	false

HGL 29	6546732	6943702	6185386	6478610	+	#FF00FF	396970	293224	-59105	7630	-59105	337865	false
HGL 29	6951332	7001825	5890193	5954764	+	#FF00FF	50493	64571	7630	16321	7630	58123	false
HGL 29	7018146	7098499	3896328	3956920	+	#FF00FF	80353	60592	16321	-23422	16321	96674	false
HGL 29	7075077	7748188	2813297	3298573	+	#FF00FF	673111	485276	-23422	68387	-23422	649689	false
HGL 29	7816575	8718076	3301344	3878398	+	#FF00FF	901501	577054	68387	89373	68387	969888	false
HGL 29	8807449	8945737	3972285	4073479	+	#FF00FF	138288	101194	89373	51925	89373	227661	false
HGL 29	8997662	9085695	4081457	4169901	+	#FF00FF	88033	88444	51925	292332	51925	139958	false
HGL 29	9378027	9566788	4203897	4405060	+	#FF00FF	188761	201163	292332	-151869	292332	481093	false
HGL 29	9414919	9493837	4300727	4393347	+	#FF00FF	78918	92620	-151869	-29788	-151869	-72951	true
HGL 29	9464049	9585290	4522109	4574493	-	#FF00FF	121241	52384	-29788	60054	-29788	91453	false
HGL 29	9645344	9699232	4399846	4460899	+	#FF00FF	53888	61053	60054	105480	60054	113942	false
HGL 29	9804712	11561477	4593168	5796770	+	#FF00FF	1756765	1203602	105480	1083610	105480	1862245	false
HGL 29	12645087	14189075	12207456	13353665	-	#FF00FF	1543988	1146209	1083610	57951	1083610	2627598	false
HGL 29	14247026	14573526	11965153	12207148	-	#FF00FF	326500	241995	57951	737	57951	384451	false
HGL 29	14574263	14658686	13336461	13456390	+	#FF00FF	84423	119929	737	272579	737	85160	false
HGL 20	14824841	14922902	65377628	65452198	-	#F7FE2D	98061	74570		-96022			
HGL 9	14826747	14903908	29636877	29688221	+	#6E0025	77161	51344		-77161			
HGL 9	14826747	14926353	27587550	27657191	-	#6E0025	99606	69641	-77161	-99606	-77161	22445	false
HGL 9	14826747	14928910	29636877	29788140	-	#6E0025	102163	151263	-99606		-99606	2557	false
HGL 20	14826880	14929667	65337137	65393879	+	#F7FE2D	102787	56742	-96022		-96022	6765	false
HGL 29	14931265	15697406	10963601	11635893	-	#FF00FF	766141	672292	272579	-58965	272579	1038720	false
HGL 29	15638441	15786030	10775493	10943649	+	#FF00FF	147589	168156	-58965	103583	-58965	88624	false
HGL 29	15889613	15997579	10896913	10971634	+	#FF00FF	107966	74721	103583	62084	103583	211549	false
HGL 29	16059663	16893903	762449	1157674	-	#FF00FF	834240	395225	62084	154545	62084	896324	false
HGL 29	17048448	17734573	401166	760655	-	#FF00FF	686125	359489	154545	12770	154545	840670	false
HGL 29	17747343	19551453	1320416	2336154	+	#FF00FF	1804110	1015738	12770	61552	12770	1816880	false
HGL 29	19613005	19664846	2336176	2387377	+	#FF00FF	51841	51201	61552		61552	113393	false
HGL 27	28098099	28737000	22869462	23437254	-	#FF7F00	638901	567792		81818			
HGL 27	28818818	32686674	23543787	27089589	+	#FF7F00	3867856	3545802	81818	13	81818	3949674	false
HGL 27	32686687	32906380	27154704	27291645	+	#FF7F00	219693	136941	13	-77312	13	219706	false
HGL 27	32829068	33125842	27310172	27516192	+	#FF7F00	296774	206020	-77312	57562	-77312	219462	false
HGL 27	33183404	34506075	28254096	29456883	-	#FF7F00	1322671	1202787	57562	407711	57562	1380233	false

	HGL 27	34913786	35645883	27521244	28077979	-	#FF7F00	732097	556735	407711	84	407711	1139808	false
	HGL 27	35645967	36240348	29491473	30032125	+	#FF7F00	594381	540652	84	97359	84	594465	false
	HGL 27	36337707	36531492	30225891	30332706	-	#FF7F00	193785	106815	97359	12819	97359	291144	false
	HGL 27	36544311	36769453	30032155	30165826	-	#FF7F00	225142	133671	12819	118099	12819	237961	false
	HGL 27	36887552	37005910	30357599	30457220	-	#FF7F00	118358	99621	118099	67477	118099	236457	false
	HGL 27	37073387	37268133	30646689	30734358	-	#FF7F00	194746	87669	67477	64577	67477	262223	false
	HGL 27	37332710	37552292	30457554	30651387	-	#FF7F00	219582	193833	64577	41434	64577	284159	false
	HGL 27	37593726	37695884	30771158	30853205	+	#FF7F00	102158	82047	41434	452	41434	143592	false
	HGL 27	37696336	37780764	30875012	30952971	-	#FF7F00	84428	77959	452	43323	452	84880	false
	HGL 27	37824087	37921424	30172603	30223497	-	#FF7F00	97337	50894	43323	-36498	43323	140660	false
	HGL 27	37884926	38989986	30986229	31845883	+	#FF7F00	1105060	859654	-36498	31877	-36498	1068562	false
	HGL 27	39021863	39709792	31903563	32438815	+	#FF7F00	687929	535252	31877	83975	31877	719806	false
	HGL 27	39793767	40864182	32443572	33183367	+	#FF7F00	1070415	739795	83975	-52477	83975	1154390	false
	HGL 27	40811705	40884447	33243627	33307305	-	#FF7F00	72742	63678	-52477	35668	-52477	20265	false
	HGL 27	40920115	41003968	33377990	33468569	-	#FF7F00	83853	90579	35668	-83853	35668	119521	false
	HGL 27	40920115	41010131	33535205	33591080	-	#FF7F00	90016	55875	-83853	-77287	-83853	6163	false
	HGL 27	40932844	41019959	33537971	33634804	-	#FF7F00	87115	96833	-77287	-29262	-77287	9828	false
	HGL 27	40990697	41065705	33301223	33354563	+	#FF7F00	75008	53340	-29262	72513	-29262	45746	false
	HGL 27	41138218	41449754	34002333	34245033	+	#FF7F00	311536	242700	72513	247046	72513	384049	false
	HGL 27	41696800	42588820	34260232	34888854	-	#FF7F00	892020	628622	247046	145003	247046	1139066	false
	HGL 27	42733823	42825784	34879402	34936515	+	#FF7F00	91961	57113	145003	23277	145003	236964	false
	HGL 27	42849061	42994093	34879402	34936515	+	#FF7F00	145032	57113	23277	185825	23277	168309	false
	HGL 27	43179918	43881462	34879402	35328680	+	#FF7F00	701544	449278	185825	84256	185825	887369	false
	HGL 27	43965718	44530016	35347024	35546349	+	#FF7F00	564298	199325	84256	56528	84256	648554	false
	HGL 27	44586544	45183787	35546776	35932382	+	#FF7F00	597243	385606	56528	24485	56528	653771	false
	HGL 27	45208272	45507531	36153317	36365261	+	#FF7F00	299259	211944	24485	-33711	24485	323744	false
	HGL 27	45473820	46029963	36410256	36847674	+	#FF7F00	556143	437418	-33711	234009	-33711	522432	false
	HGL 27	46263972	47853055	36885974	37980803	+	#FF7F00	1589083	1094829	234009	137754	234009	1823092	false
	HGL 27	47990809	48204432	38231901	38413374	+	#FF7F00	213623	181473	137754	61248	137754	351377	false
	HGL 27	48265680	50067931	38592445	39774039	+	#FF7F00	1802251	1181594	61248	75704	61248	1863499	false
	HGL 27	50143635	51566456	39777979	40680495	+	#FF7F00	1422821	902516	75704	-24871	75704	1498525	false
	HGL 27	51541585	51829191	40606331	40768595	+	#FF7F00	287606	162264	-24871	-66499	-24871	262735	false

	HGL 27	51762692	51858509	40780231	40844888	+	#FF7F00	95817	64657	-66499	-3245	-66499	29318	false
	HGL 27	51855264	52019187	40876933	40975682	+	#FF7F00	163923	98749	-3245	158624	-3245	160678	false
	HGL 27	52177811	52246345	41009284	41083901	-	#FF7F00	68534	74617	158624	-5596	158624	227158	false
	HGL 27	52240749	52435493	41095532	41180691	+	#FF7F00	194744	85159	-5596	764274	-5596	189148	false
	HGL 27	53199767	53285941	41221984	41290736	-	#FF7F00	86174	68752	764274	14176	764274	850448	false
	HGL 27	53300117	53376270	41112762	41181454	+	#FF7F00	76153	68692	14176	484275	14176	90329	false
	HGL 27	53860545	54291036	41485320	41704663	+	#FF7F00	430491	219343	484275	63046	484275	914766	false
	HGL 27	54354082	54474628	41724035	41789569	+	#FF7F00	120546	65534	63046	537552	63046	183592	false
	HGL 27	55012180	55696882	41852399	42329564	+	#FF7F00	684702	477165	537552	390081	537552	1222254	false
	HGL 27	56086963	56315735	42457727	42550060	+	#FF7F00	228772	92333	390081	63290	390081	618853	false
	HGL 27	56379025	56483409	42556810	42630913	-	#FF7F00	104384	74103	63290	282359	63290	167674	false
	HGL 27	56765768	56881101	42811101	42894610	+	#FF7F00	115333	83509	282359	286629	282359	397692	false
	HGL 27	57167730	57499089	42932779	43222534	+	#FF7F00	331359	289755	286629	71945	286629	617988	false
	HGL 27	57571034	57859266	43241117	43408663	+	#FF7F00	288232	167546	71945	81605	71945	360177	false
	HGL 27	57940871	58003098	43515588	43570143	+	#FF7F00	62227	54555	81605	7933	81605	143832	false
	HGL 27	58011031	58576795	43627317	44036224	+	#FF7F00	565764	408907	7933		7933	573697	false
**HSA 2**	HGL 26	37055	684748	31128447	31399143	+	#7FBF7F	647693	270696		353746			
	HGL 26	1038494	1548270	31399631	31506193	-	#7FBF7F	509776	106562	353746	33542	353746	863522	false
	HGL 26	1581812	9090765	31575441	35814001	-	#7FBF7F	7508953	4238560	33542	121286	33542	7542495	false
	HGL 26	9212051	9768457	35815587	36148379	+	#7FBF7F	556406	332792	121286	71202	121286	677692	false
	HGL 26	9839659	11074848	36302566	36826043	+	#7FBF7F	1235189	523477	71202	58196	71202	1306391	false
	HGL 26	11133044	15863551	36831356	40635545	+	#7FBF7F	4730507	3804189	58196	202642	58196	4788703	false
	HGL 26	16066193	16770796	40672644	41239835	+	#7FBF7F	704603	567191	202642	75088182	202642	907245	false
	HGL 5	17223333	24784314	120580222	127547228	-	#2DCDC1	7560981	6967006		0			
	HGL 5	24784314	31586961	114312316	120503926	-	#2DCDC1	6802647	6191610	0	74953	0	6802647	false
	HGL 5	31661914	33403507	112606449	114311251	-	#2DCDC1	1741593	1704802	74953	1106	74953	1816546	false
	HGL 5	33404613	47330693	98979415	112549350	-	#2DCDC1	13926080	13569935	1106	2760	1106	13927186	false
	HGL 5	47333453	54164001	92283808	98918416	-	#2DCDC1	6830548	6634608	2760	-6073	2760	6833308	false
	HGL 5	54157928	56413562	89825618	92125297	-	#2DCDC1	2255634	2299679	-6073	3654	-6073	2249561	false
	HGL 5	56417216	56573542	89687236	89824162	+	#2DCDC1	156326	136926	3654	1758	3654	159980	false
	HGL 5	56575300	61053731	85558168	89682674	-	#2DCDC1	4478431	4124506	1758	630	1758	4480189	false
	HGL 5	61054361	61923744	84654727	85481411	-	#2DCDC1	869383	826684	630	67359	630	870013	false

	HGL 5	61991103	63320788	83323480	84645926	-	#2DCDC1	1329685	1322446	67359	51588	67359	1397044	false
	HGL 5	63372376	64420037	82332498	83291649	-	#2DCDC1	1047661	959151	51588	0	51588	1099249	false
	HGL 5	64420037	64475012	82247294	82312345	+	#2DCDC1	54975	65051	0	2708	0	54975	false
	HGL 5	64477720	71091031	75619498	82245234	-	#2DCDC1	6613311	6625736	2708	87544	2708	6616019	false
	HGL 5	71178575	73645621	73319852	75625482	-	#2DCDC1	2467046	2305630	87544	25020	87544	2554590	false
	HGL 5	73670641	73747048	73275964	73381890	-	#2DCDC1	76407	105926	25020	-1461	25020	101427	false
	HGL 5	73745587	80901935	65508839	73205870	-	#2DCDC1	7156348	7697031	-1461	48858	-1461	7154887	false
	HGL 5	80950793	86866856	59975274	65456817	-	#2DCDC1	5916063	5481543	48858	258005	48858	5964921	false
	HGL 5	87124861	87246317	43952251	44059942	+	#2DCDC1	121456	107691	258005	185130	258005	379461	false
	HGL 5	87431447	87493090	44248042	44312031	+	#2DCDC1	61643	63989	185130	58559	185130	246773	false
	HGL 5	87551649	87695749	44332402	44483436	+	#2DCDC1	144100	151034	58559	301947	58559	202659	false
	HGL X	87826010	87961255	143949561	144093620	-	#00FF00	135245	144059		61824			
	HGL 5	87997696	88132125	59771041	59967999	-	#2DCDC1	134429	196958	301947	-18394	301947	436376	false
	HGL X	88023079	88080856	97798401	97897584	+	#00FF00	57777	99183	61824	97665174	61824	119601	false
	HGL 5	88113731	88755713	59241681	59739303	-	#2DCDC1	641982	497622	-18394	136730	-18394	623588	false
	HGL 5	88892443	89079108	57414365	57479647	-	#2DCDC1	186665	65282	136730	-156188	136730	323395	false
	HGL 5	88922920	89310686	58515506	58606307	-	#2DCDC1	387766	90801	-156188	-381809	-156188	231578	false
	HGL 5	88928877	89049092	58372618	58441177	-	#2DCDC1	120215	68559	-381809	-115398	-381809	-261594	true
	HGL 5	88933694	89319735	58426429	58586028	-	#2DCDC1	386041	159599	-115398	-373974	-115398	270643	false
	HGL 5	88945761	89171404	58372987	58531906	-	#2DCDC1	225643	158919	-373974	-225643	-373974	-148331	true
	HGL 5	88945761	89171479	57893226	58004631	-	#2DCDC1	225718	111405	-225643	-225690	-225643	75	false
	HGL 5	88945789	89256403	57882859	58027778	-	#2DCDC1	310614	144919	-225690	-309355	-225690	84924	false
	HGL 5	88947048	89118059	58666073	58718417	-	#2DCDC1	171011	52344	-309355	-171011	-309355	-138344	true
	HGL 5	88947048	89171404	58479394	58580607	-	#2DCDC1	224356	101213	-171011	-222391	-171011	53345	false
	HGL 5	88949013	89330722	58611921	58785849	-	#2DCDC1	381709	173928	-222391	-352446	-222391	159318	false
	HGL 5	88978276	89135254	57501803	57596231	-	#2DCDC1	156978	94428	-352446	-156978	-352446	-195468	true
	HGL 5	88978276	89255318	57706718	57775061	-	#2DCDC1	277042	68343	-156978	-193892	-156978	120064	false
	HGL 5	89061426	89319735	58625274	58723201	-	#2DCDC1	258309	97927	-193892	-177240	-193892	64417	false
	HGL 5	89142495	89310686	58533751	58588321	-	#2DCDC1	168191	54570	-177240	-118080	-177240	-9049	true
	HGL 5	89192606	89255257	57706775	57774736	-	#2DCDC1	62651	67961	-118080	-34522	-118080	-55429	true
	HGL 5	89220735	89298946	58311499	58361533	+	#2DCDC1	78211	50034	-34522	-78211	-34522	43689	false
	HGL 5	89220735	89330071	57502127	57599459	-	#2DCDC1	109336	97332	-78211	511719	-78211	31125	false

	HGL 5	89841790	89892686	57881914	57993560	+	#2DCDC1	50896	111646	511719	-50896	511719	562615	false
	HGL 5	89841790	89961715	57502519	57599459	+	#2DCDC1	119925	96940	-50896	-114321	-50896	69029	false
	HGL 5	89847394	90200430	58594547	58723201	+	#2DCDC1	353036	128654	-114321	-349403	-114321	238715	false
	HGL 5	89851027	90235446	58515363	58588321	+	#2DCDC1	384419	72958	-349403	-372578	-349403	35016	false
	HGL 5	89862868	90235446	58426429	58566428	+	#2DCDC1	372578	139999	-372578	-326403	-372578	0	true
	HGL 5	89909043	90235507	58396520	58566494	+	#2DCDC1	326464	169974	-326403	-308389	-326403	61	false
	HGL 5	89927118	90235559	57706718	57775061	+	#2DCDC1	308441	68343	-308389	-297445	-308389	52	false
	HGL 5	89938114	90013024	57939518	57990652	+	#2DCDC1	74910	51134	-297445	-8317	-297445	-222535	true
	HGL 5	90004707	90115540	57501209	57596180	+	#2DCDC1	110833	94971	-8317	-110374	-8317	102516	false
	HGL 5	90005166	90223260	57881003	57970775	+	#2DCDC1	218094	89772	-110374	-218094	-110374	107720	false
	HGL 5	90005166	90235559	57103425	57198501	+	#2DCDC1	230393	95076	-218094	-225086	-218094	12299	false
	HGL 5	90010473	90222888	57893226	57955464	+	#2DCDC1	212415	62238	-225086	-212340	-225086	-12671	true
	HGL 5	90010548	90235507	58372987	58524289	+	#2DCDC1	224959	151302	-212340	-188709	-212340	12619	false
	HGL 5	90046798	90235559	57501803	57596231	+	#2DCDC1	188761	94428	-188709	-155706	-188709	52	false
	HGL 5	90079853	90234806	58372618	58482414	+	#2DCDC1	154953	109796	-155706	-151855	-155706	-753	true
	HGL 5	90082951	90222888	57954413	58027778	+	#2DCDC1	139937	73365	-151855	4756520	-151855	-11918	true
	HGL 5	94979408	95414295	45263654	45616879	+	#2DCDC1	434887	353225	4756520	227687	4756520	5191407	false
	HGL 5	95641982	95744187	56825406	56928422	+	#2DCDC1	102205	103016	227687	280209	227687	329892	false
	HGL 5	96024396	96375948	45618116	45904440	+	#2DCDC1	351552	286324	280209	97290	280209	631761	false
	HGL 5	96473238	96979218	45909280	46292229	+	#2DCDC1	505980	382949	97290	664281	97290	603270	false
	HGL 5	97643499	106203482	46295615	53493230	+	#2DCDC1	8559983	7197615	664281	327681	664281	9224264	false
	HGL 5	106531163	107825036	53569282	54746097	+	#2DCDC1	1293873	1176815	327681	99049	327681	1621554	false
	HGL 5	107924085	108187329	54747115	54979692	+	#2DCDC1	263244	232577	99049	196057	99049	362293	false
	HGL 5	108383386	109725990	55107228	55965584	+	#2DCDC1	1342604	858356	196057	63275	196057	1538661	false
	HGL 5	109789265	109841441	55310677	55373399	+	#2DCDC1	52176	62722	63275	223300	63275	115451	false
	HGL 5	110064741	110232565	45173503	45289687	-	#2DCDC1	167824	116184	223300	297500	223300	391124	false
	HGL 5	110530065	110582224	55310677	55373399	-	#2DCDC1	52159	62722	297500	53838	297500	349659	false
	HGL 5	110636062	111399171	44332402	45110639	-	#2DCDC1	763109	778237	53838	57964	53838	816947	false
	HGL 5	111457135	112345717	43484472	44312031	-	#2DCDC1	888582	827559	57964	116456	57964	946546	false
	HGL 5	112462173	113392708	55998234	56942124	+	#2DCDC1	930535	943890	116456	52141458	116456	1046991	false
	HGL 1	113672181	113782199	21158180	21255460	+	#13C840	110018	97280		531			
	HGL 1	113782730	114185212	21308396	21821634	+	#13C840	402482	513238	531	0	531	403013	false

	HGL 1	114185212	114331835	21828097	21996491	-	#13C840	146623	168394	0	0	0	146623	false
	HGL 1	114331835	115959391	22003526	23576759	+	#13C840	1627556	1573233	0	57594	0	1627556	false
	HGL 1	116016985	126494621	23586746	34728706	+	#13C840	10477636	11141960	57594	7783	57594	10535230	false
	HGL 1	126502404	126939634	34780374	35295546	+	#13C840	437230	515172	7783	10694	7783	445013	false
	HGL 1	126950328	127191818	35365547	35642323	+	#13C840	241490	276776	10694	60798	10694	252184	false
	HGL 1	127252616	127896208	35652599	36272839	+	#13C840	643592	620240	60798	12727	60798	704390	false
	HGL 1	127908935	129657995	37585426	39509930	-	#13C840	1749060	1924504	12727	14098	12727	1761787	false
	HGL 1	129672093	129920729	37167276	37525295	-	#13C840	248636	358019	14098	414458	14098	262734	false
	HGL 19	130135519	130196437	26546542	26597384	+	#0000FF	60918	50842		1281660			
	HGL 1	130335187	130393705	36388674	36469687	+	#13C840	58518	81013	414458	326498	414458	472976	false
	HGL 1	130720203	131157934	36660286	37149606	+	#13C840	437731	489320	326498	1208574	326498	764229	false
	HGL 19	131478097	131537437	26546542	26597384	-	#0000FF	59340	50842	1281660	69945461	1281660	1341000	false
	HGL 1	132366508	147180561	5781851	21131304	-	#13C840	14814053	15349453	1208574	168897	1208574	16022627	false
	HGL 1	147349458	151335751	1479835	5781366	-	#13C840	3986293	4301531	168897	3941	168897	4155190	false
	HGL 1	151339692	152717753	45687	1412081	+	#13C840	1378061	1366394	3941	1	3941	1382002	false
	HGL 1	152717754	165454019	39542721	52846980	+	#13C840	12736265	13304259	1	-20929	1	12736266	false
	HGL 1	165433090	178162136	52913088	64964956	+	#13C840	12729046	12051868	-20929	-53926	-20929	12708117	false
	HGL 1	178108210	182707284	65111919	69454370	+	#13C840	4599074	4342451	-53926	20	-53926	4545148	false
	HGL 1	182707304	183544663	69515644	70255325	+	#13C840	837359	739681	20	2334	20	837379	false
	HGL 1	183546997	183771413	70259014	70486712	-	#13C840	224416	227698	2334	12799	2334	226750	false
	HGL 1	183784212	184309976	70486813	70926638	+	#13C840	525764	439825	12799	5956	12799	538563	false
	HGL 1	184315932	184464341	70928421	71086674	-	#13C840	148409	158253	5956	5074	5956	154365	false
	HGL 1	184469415	185579819	71089308	72022942	+	#13C840	1110404	933634	5074	-23630	5074	1115478	false
	HGL 1	185556189	185739743	72033313	72182652	+	#13C840	183554	149339	-23630	166713	-23630	159924	false
	HGL 1	185906456	186118940	72256597	72449341	-	#13C840	212484	192744	166713	48091	166713	379197	false
	HGL 1	186167031	186395602	72454516	72716669	+	#13C840	228571	262153	48091	-216458	48091	276662	false
	HGL 1	186179144	186479346	78851863	79184583	+	#13C840	300202	332720	-216458	-21438	-216458	83744	false
	HGL 1	186457908	186606117	72717257	72866703	+	#13C840	148209	149446	-21438	-123562	-21438	126771	false
	HGL 1	186482555	189694630	79266005	82140754	+	#13C840	3212075	2874749	-123562	236416	-123562	3088513	false
	HGL 1	189931046	194116180	75398274	78851422	-	#13C840	4185134	3453148	236416	52532	236416	4421550	false
	HGL 1	194168712	196828428	73193429	75398116	-	#13C840	2659716	2204687	52532	3958	52532	2712248	false
	HGL 1	196832386	200778123	82536069	85389892	-	#13C840	3945737	2853823	3958	-180799	3958	3949695	false

	HGL 1	200597324	201222745	82171453	82578723	-	#13C840	625421	407270	-180799	272	-180799	444622	false
	HGL 1	201223017	201481682	85505168	85748687	+	#13C840	258665	243519	272	140167	272	258937	false
	HGL 1	201621849	201785979	85759102	85931005	+	#13C840	164130	171903	140167		140167	304297	false
	HGL 24	201778160	208499029	28658540	35824115	-	#F50241	6720869	7165575		82228			
	HGL 24	208581257	208839989	28478249	28640737	-	#F50241	258732	162488	82228	76785	82228	340960	false
	HGL 24	208916774	230400023	7970410	28472242	-	#F50241	21483249	20501832	76785	-30548	76785	21560034	false
	HGL 24	230369475	231536383	7351889	8072307	-	#F50241	1166908	720418	-30548	56115	-30548	1136360	false
	HGL 24	231592498	233683528	5829901	7351865	-	#F50241	2091030	1521964	56115	-104184	56115	2147145	false
	HGL 24	233579344	240110036	1287214	5726296	-	#F50241	6530692	4439082	-104184	-6508325	-104184	6426508	false
	HGL 24	233601711	233683152	5756752	5820776	-	#F50241	81441	64024	-6508325	-66224	-6508325	-6426884	true
	HGL 24	233616928	233683528	5769815	5820281	-	#F50241	66600	50466	-66224	6395229	-66224	376	false
	HGL 24	240078757	240646317	837552	1178141	-	#F50241	567560	340589	6395229	65113	6395229	6962789	false
	HGL 24	240711430	241889761	42028	835814	-	#F50241	1178331	793786	65113		65113	1243444	false
**HSA 20**	HGL 9	90257	740907	42999733	43639099	+	#6E0025	650650	639366		7493			
	HGL 9	748400	986780	43783256	43933987	+	#6E0025	238380	150731	7493	-113843	7493	245873	false
	HGL 9	872937	992672	44009899	44078137	+	#6E0025	119735	68238	-113843	-119735	-113843	5892	false
	HGL 9	872937	1708626	44078137	44804150	+	#6E0025	835689	726013	-119735	-113831	-119735	715954	false
	HGL 9	1594795	1707669	44749768	44803081	+	#6E0025	112874	53313	-113831	56185	-113831	-957	true
	HGL 9	1763854	18215836	44949671	61009115	+	#6E0025	16451982	16059444	56185	164936	56185	16508167	false
	HGL 9	18380772	23606586	61052805	65065827	+	#6E0025	5225814	4013022	164936	5379	164936	5390750	false
	HGL 9	23611965	23777090	65166822	65231633	+	#6E0025	165125	64811	5379	-91776	5379	170504	false
	HGL 9	23685314	23923110	65174718	65231633	+	#6E0025	237796	56915	-91776	121873	-91776	146020	false
	HGL 9	24044983	25623076	65356257	66254371	+	#6E0025	1578093	898114	121873	5665694	121873	1699966	false
	HGL 9	31288770	33194298	41375255	42993673	-	#6E0025	1905528	1618418	5665694	-25559	5665694	7571222	false
	HGL 9	33168739	34182548	40401621	41375073	-	#6E0025	1013809	973452	-25559	51729	-25559	988250	false
	HGL 9	34234277	41324190	33606723	40388951	-	#6E0025	7089913	6782228	51729	1552	51729	7141642	false
	HGL 9	41325742	45139333	23444	3571015	+	#6E0025	3813591	3547571	1552	31834	1552	3815143	false
	HGL 9	45171167	45449162	3645812	3883467	+	#6E0025	277995	237655	31834	6423	31834	309829	false
	HGL 9	45455585	45663622	3993625	4118426	+	#6E0025	208037	124801	6423	56942	6423	214460	false
	HGL 9	45720564	59521485	4122258	15521043	+	#6E0025	13800921	11398785	56942	-11246446	56942	13857863	false
	HGL 9	48275039	48570538	5787394	6021953	-	#6E0025	295499	234559	-11246446	7889352	-11246446	-10950947	true
	HGL 9	56459890	56594731	65066373	65152193	+	#6E0025	134841	85820	7889352	2950889	7889352	8024193	false

	HGL 9	59545620	64276314	15589975	19574076	+	#6E0025	4730694	3984101	2950889		2950889	7681583	false
**HSA 21**	HGL 2	5011613	5165890	1759230	1852947	-	#C008AD	154277	93717		800627			
	HGL 2	5966517	6076907	4174475	4275534	-	#C008AD	110390	101059	800627	7079245	800627	911017	false
	HGL 2	13156152	13342198	15813753	16031117	-	#C008AD	186046	217364	7079245	763662	7079245	7265291	false
	HGL 2	14105860	17273027	26162476	29116325	-	#C008AD	3167167	2953849	763662	51311	763662	3930829	false
	HGL 2	17324338	18691695	24503000	26131957	-	#C008AD	1367357	1628957	51311	2698	51311	1418668	false
	HGL 2	18694393	18999733	24202079	24502460	+	#C008AD	305340	300381	2698	261	2698	308038	false
	HGL 2	18999994	28825275	14926614	24201054	-	#C008AD	9825281	9274440	261	-4867005	261	9825542	false
	HGL 2	23958270	24102295	18263487	18414391	-	#C008AD	144025	150904	-4867005	698252	-4867005	-4722980	true
	HGL 2	24800547	24912042	19914844	20039129	-	#C008AD	111495	124285	698252	4009297	698252	809747	false
	HGL 2	28921339	30526856	13093612	14800244	-	#C008AD	1605517	1706632	4009297	-4190	4009297	5614814	false
	HGL 2	30522666	30639575	12825844	13091871	-	#C008AD	116909	266027	-4190	-4739	-4190	112719	false
	HGL 2	30634836	30751789	12530850	12710822	-	#C008AD	116953	179972	-4739	-12489	-4739	112214	false
	HGL 2	30739300	32535052	10875473	12527770	-	#C008AD	1795752	1652297	-12489	60734	-12489	1783263	false
	HGL 2	32595786	36075957	7579178	10875425	-	#C008AD	3480171	3296247	60734	49711	60734	3540905	false
	HGL 2	36125668	39805332	4417347	7447880	+	#C008AD	3679664	3030533	49711	31641	49711	3729375	false
	HGL 2	39836973	41283948	2020374	3042401	+	#C008AD	1446975	1022027	31641	26944	31641	1478616	false
	HGL 2	41310892	41462533	3043260	3095919	-	#C008AD	151641	52659	26944	-38828	26944	178585	false
	HGL 2	41423705	41563545	3108392	3181776	+	#C008AD	139840	73384	-38828	155899	-38828	101012	false
	HGL 2	41719444	42789353	3201857	3891972	+	#C008AD	1069909	690115	155899	58663	155899	1225808	false
	HGL 2	42848016	43131622	3898212	4084812	+	#C008AD	283606	186600	58663	140934	58663	342269	false
	HGL 2	43272556	43376608	4116187	4172382	+	#C008AD	104052	56195	140934	84749	140934	244986	false
	HGL 2	43461357	43694566	4174475	4361616	+	#C008AD	233209	187141	84749	20844	84749	317958	false
	HGL 2	43715410	44469116	1539350	1987295	+	#C008AD	753706	447945	20844	30088	20844	774550	false
	HGL 2	44499204	46666244	15283	1539350	-	#C008AD	2167040	1524067	30088		30088	2197128	false
**HSA 22**	HGL 12	17064707	18178377	89967493	90990455	+	#DD528A	1113670	1022962					
	HGL 19	19021794	19291967	25987289	26298375	-	#0000FF	270173	311086		64897			
	HGL 19	19356864	20323822	27022280	27935041	+	#0000FF	966958	912761	64897	69119	64897	1031855	false
	HGL 19	20392941	20626226	26314397	26577215	+	#0000FF	233285	262818	69119	74091	69119	302404	false
	HGL 19	20700317	21081007	26580449	27008277	-	#0000FF	380690	427828	74091	463542	74091	454781	false
	HGL 19	21544549	21985513	28132434	28497148	+	#0000FF	440964	364714	463542	892018	463542	904506	false
	HGL 19	22877531	23347362	36276222	36745917	+	#0000FF	469831	469695	892018	390048	892018	1361849	false

	HGL 19	23737410	24250892	37067620	37461277	-	#0000FF	513482	393657	390048	15579	390048	903530	false
	HGL 19	24266471	24636386	36692348	37076424	+	#0000FF	369915	384076	15579	79376	15579	385494	false
	HGL 19	24715762	24792136	39618221	39699985	+	#0000FF	76374	81764	79376	-56850	79376	155750	false
	HGL 19	24735286	25276737	28816762	29260984	-	#0000FF	541451	444222	-56850	178104	-56850	484601	false
	HGL 19	25454841	25730990	28583915	28878415	-	#0000FF	276149	294500	178104	4681	178104	454253	false
	HGL 19	25735671	29398529	29352706	32789504	+	#0000FF	3662858	3436798	4681	17927	4681	3667539	false
	HGL 19	29416456	32009282	32754184	35414174	+	#0000FF	2592826	2659990	17927	24969	17927	2610753	false
	HGL 19	32034251	32111148	35469515	35564141	+	#0000FF	76897	94626	24969		24969	101866	false
	HGL 16	32387502	33140349	1595462	2062714	-	#80E0F6	752847	467252		59166			
	HGL 16	33199515	35148107	435641	1595442	-	#80E0F6	1948592	1159801	59166	51979	59166	2007758	false
	HGL 16	35200086	35456085	315036	432725	-	#80E0F6	255999	117689	51979	52357	51979	307978	false
	HGL 16	35508442	35658503	247663	311415	-	#80E0F6	150061	63752	52357		52357	202418	false
	HGL 8	35648762	36187812	11689967	12083015	-	#6A78D4	539050	393048	18655412	-57009	18655412	19194462	false
	HGL 8	36130803	38942282	9237919	11459434	-	#6A78D4	2811479	2221515	-57009	157102	-57009	2754470	false
	HGL 8	39099384	41198078	7376676	9207043	-	#6A78D4	2098694	1830367	157102	-24059	157102	2255796	false
	HGL 8	41174019	42521931	6229471	7322830	-	#6A78D4	1347912	1093359	-24059	26503	-24059	1323853	false
	HGL 8	42548434	47363028	2771389	6264854	-	#6A78D4	4814594	3493465	26503	805	26503	4841097	false
	HGL 8	47363833	47479851	2670606	2769899	+	#6A78D4	116018	99293	805	8763	805	116823	false
	HGL 8	47488614	50785612	7256	2670106	-	#6A78D4	3296998	2662850	8763		8763	3305761	false
**HSA 3**	HGL 4	12066	3968415	55380300	59663446	+	#0C404C	3956349	4283146		0			
	HGL 4	3968415	5173746	59720634	61107230	+	#0C404C	1205331	1386596	0	-66809	0	1205331	false
	HGL 4	5106937	5812915	61256299	62350110	+	#0C404C	705978	1093811	-66809	8212	-66809	639169	false
	HGL 4	5821127	6041039	62477545	62693960	-	#0C404C	219912	216415	8212	20	8212	228124	false
	HGL 4	6041059	6116916	62370838	62470068	+	#0C404C	75857	99230	20	9855	20	75877	false
	HGL 4	6126771	8442826	62698125	65437205	+	#0C404C	2316055	2739080	9855	6758101	9855	2325910	false
	HGL 2	8436423	9568141	80305193	81470516	+	#C008AD	1131718	1165323		81149			
	HGL 2	9649290	10127304	81511700	81907970	+	#C008AD	478014	396270	81149	14361	81149	559163	false
	HGL 2	10141665	10753965	81948375	82581126	-	#C008AD	612300	632751	14361	27368343	14361	626661	false
	HGL 6	10753986	11847685	90827744	91675477	+	#60FA36	1093699	847733		82295			
	HGL 6	11929980	12533168	91761946	92266273	+	#60FA36	603188	504327	82295	3980	82295	685483	false
	HGL 6	12537148	12726957	87408673	87539700	+	#60FA36	189809	131027	3980	2718	3980	193789	false
	HGL 6	12729675	12842398	87610667	87683707	+	#60FA36	112723	73040	2718	67742	2718	115441	false

	HGL 6	12910140	13897637	92267858	92999287	+	#60FA36	987497	731429	67742	211862	67742	1055239	false
	HGL 6	14109499	15102688	88128911	88736404	-	#60FA36	993189	607493	211862	24275968	211862	1205051	false
	HGL 4	15200927	15382870	94288645	94469750	+	#0C404C	181943	181105	6758101	19696	6758101	6940044	false
	HGL 4	15402566	15611793	94476914	94696270	-	#0C404C	209227	219356	19696	-58057	19696	228923	false
	HGL 4	15553736	21081783	82969595	88506808	-	#0C404C	5528047	5537213	-58057	3747	-58057	5469990	false
	HGL 4	21085530	21245452	82827810	82946091	+	#0C404C	159922	118281	3747	3655	3747	163669	false
	HGL 4	21249107	22261033	81769325	82806645	-	#0C404C	1011926	1037320	3655	-42994	3655	1015581	false
	HGL 4	22218039	37249418	66240042	81712759	-	#0C404C	15031379	15472717	-42994	149396	-42994	14988385	false
	HGL 4	37398814	38144059	65471345	66239492	-	#0C404C	745245	768147	149396	119387	149396	894641	false
	HGL 2	38122308	38258673	79887400	80058306	+	#C008AD	136365	170906	27368343	66357	27368343	27504708	false
	HGL 4	38263446	38329698	65429946	65519653	+	#0C404C	66252	89707	119387	13985439	119387	185639	false
	HGL 2	38325030	39381900	78824757	79887607	-	#C008AD	1056870	1062850	66357	35861711	66357	1123227	false
	HGL 6	39378656	40463187	50282	604828	+	#60FA36	1084531	554546	24275968	70314	24275968	25360499	false
	HGL 6	40533501	44455754	610912	2944185	+	#60FA36	3922253	2333273	70314	22258	70314	3992567	false
	HGL 6	44478012	46755913	3017423	4441155	+	#60FA36	2277901	1423732	22258	73305	22258	2300159	false
	HGL 6	46829218	48199660	4438243	5454269	+	#60FA36	1370442	1016026	73305	144449	73305	1443747	false
	HGL 6	48344109	48509879	76428651	76549912	-	#60FA36	165770	121261	144449	5474	144449	310219	false
	HGL 6	48515353	48905228	76021293	76366003	-	#60FA36	389875	344710	5474	11071	5474	395349	false
	HGL 6	48916299	52313051	72740331	75933575	-	#60FA36	3396752	3193244	11071	73215244	11071	3407823	false
	HGL 4	52315137	58256157	88523898	94288602	-	#0C404C	5941020	5764704	13985439	-50580	13985439	19926459	false
	HGL 4	58205577	59057111	94721382	95380061	+	#0C404C	851534	658679	-50580	66733	-50580	800954	false
	HGL 4	59123844	62187702	95461981	98356253	+	#0C404C	3063858	2894272	66733	63215	66733	3130591	false
	HGL 4	62250917	63723958	98422304	99807948	+	#0C404C	1473041	1385644	63215	16325	63215	1536256	false
	HGL 4	63740283	66084791	99860421	102034657	+	#0C404C	2344508	2174236	16325	128860	16325	2360833	false
	HGL 4	66213651	75243253	46022788	55416279	+	#0C404C	9029602	9393491	128860	50758887	128860	9158462	false
	HGL 2	75243611	75325561	77144072	77329199	+	#C008AD	81950	185127	35861711	484875	35861711	35943661	false
	HGL 19	75414969	75515354	20846187	20918415	-	#0000FF	100385	72228		1398			
	HGL 19	75516752	75591357	20755433	20824442	+	#0000FF	74605	69009	1398	50164025	1398	76003	false
	HGL 2	75810436	77841562	29128843	31122228	+	#C008AD	2031126	1993385	484875	4289	484875	2516001	false
	HGL 2	77845851	77969493	31122294	31280409	-	#C008AD	123642	158115	4289	3764	4289	127931	false
	HGL 2	77973257	90262808	31280416	43983638	+	#C008AD	12289551	12703222	3764	3545798	3764	12293315	false
	HGL 2	93808606	95176191	43869643	45157054	+	#C008AD	1367585	1287411	3545798	15352	3545798	4913383	false

	HGL 2	95191543	95352817	45210911	45349066	+	#C008AD	161274	138155	15352	73253	15352	176626	false
	HGL 2	95426070	98088659	45433213	47827434	+	#C008AD	2662589	2394221	73253	-35354	73253	2735842	false
	HGL 2	98053305	98103390	47822286	47914150	-	#C008AD	50085	91864	-35354	-43784	-35354	14731	false
	HGL 2	98059606	98314363	47992420	48083351	+	#C008AD	254757	90931	-43784	-248152	-43784	210973	false
	HGL 2	98066211	98337704	47942894	48230697	+	#C008AD	271493	287803	-248152	-203256	-248152	23341	false
	HGL 2	98134448	98328590	48083066	48162425	+	#C008AD	194142	79359	-203256	-182529	-203256	-9114	true
	HGL 2	98146061	98500190	48373706	48607507	+	#C008AD	354129	233801	-182529	-111624	-182529	171600	false
	HGL 2	98388566	98500192	48278402	48355434	+	#C008AD	111626	77032	-111624	-29950	-111624	2	false
	HGL 2	98470242	98634174	48605906	48832506	+	#C008AD	163932	226600	-29950	-18607	-29950	133982	false
	HGL 2	98615567	100535998	48945616	51242602	+	#C008AD	1920431	2296986	-18607	-72633	-18607	1901824	false
	HGL 2	100463365	103984870	51335280	55276836	+	#C008AD	3521505	3941556	-72633	10178	-72633	3448872	false
	HGL 2	103995048	104096678	55277281	55370662	-	#C008AD	101630	93381	10178	1571	10178	111808	false
	HGL 2	104098249	108524763	55372833	60084714	+	#C008AD	4426514	4711881	1571	-176215	1571	4428085	false
	HGL 2	108348548	109490579	60144978	61419026	+	#C008AD	1142031	1274048	-176215	77194	-176215	965816	false
	HGL 2	109567773	118038744	61464842	70674569	+	#C008AD	8470971	9209727	77194	0	77194	8548165	false
	HGL 2	118038744	118136728	70682200	70829123	-	#C008AD	97984	146923	0	40042	0	97984	false
	HGL 2	118176770	120958284	70860085	73794206	+	#C008AD	2781514	2934121	40042	50383	40042	2821556	false
	HGL 2	121008667	121757552	73801948	74548275	+	#C008AD	748885	746327	50383	10195	50383	799268	false
	HGL 2	121767747	125524976	74845857	78859934	+	#C008AD	3757229	4014077	10195		10195	3767424	false
	HGL 6	125528295	125629441	5491544	5579737	+	#60FA36	101146	88193	73215244	1061845	73215244	73316390	false
	HGL 19	125755382	125822670	20755433	20824442	-	#0000FF	67288	69009	50164025	1398	50164025	50231313	false
	HGL 19	125824068	125934781	20846187	20918527	+	#0000FF	110713	72340	1398	4156457	1398	112111	false
	HGL 6	126691286	127440270	88852814	89593589	+	#60FA36	748984	740775	1061845	130240	1061845	1810829	false
	HGL 6	127570510	128002943	89668213	90051401	-	#60FA36	432433	383188	130240	41076	130240	562673	false
	HGL 6	128044019	128651317	90067684	90547823	+	#60FA36	607298	480139	41076	16411	41076	648374	false
	HGL 6	128667728	128807691	90556789	90665877	-	#60FA36	139963	109088	16411	280695	16411	156374	false
	HGL 6	129088386	129245128	90677722	90793858	+	#60FA36	156742	116136	280695	149922	280695	437437	false
	HGL 6	129395050	129893834	87678593	88063129	+	#60FA36	498784	384536	149922	323184	149922	648706	false
	HGL 6	130217018	130282539	72590908	72678052	-	#60FA36	65521	87144	323184	77538	323184	388705	false
	HGL 6	130360077	132261036	70518746	72588822	-	#60FA36	1900959	2070076	77538	53936	77538	1978497	false
	HGL 6	132314972	132971536	69688129	70509260	-	#60FA36	656564	821131	53936	34	53936	710500	false
	HGL 6	132971570	138640200	63734023	69637778	-	#60FA36	5668630	5903755	34	10255	34	5668664	false

	HGL 6	138650455	138993414	63330571	63656805	-	#60FA36	342959	326234	10255	11595	10255	353214	false
	HGL 6	139005009	142241323	60007613	63253530	-	#60FA36	3236314	3245917	11595	55572	11595	3247909	false
	HGL 6	142296895	145193064	56979604	59993914	-	#60FA36	2896169	3014310	55572	46	55572	2951741	false
	HGL 6	145193110	145279010	56905580	56979602	+	#60FA36	85900	74022	46	2217	46	85946	false
	HGL 6	145281227	146284758	55725948	56858387	-	#60FA36	1003531	1132439	2217	45146	2217	1005748	false
	HGL 6	146329904	146386361	55725957	55797287	-	#60FA36	56457	71330	45146	-9063	45146	101603	false
	HGL 6	146377298	146551745	55561010	55673314	-	#60FA36	174447	112304	-9063	-123616	-9063	165384	false
	HGL 6	146428129	151757965	49732250	55532509	-	#60FA36	5329836	5800259	-123616	-63043	-123616	5206220	false
	HGL 6	151694922	151804403	49660714	49733942	-	#60FA36	109481	73228	-63043	-85124	-63043	46438	false
	HGL 6	151719279	151827374	49518249	49603161	-	#60FA36	108095	84912	-85124	-50703	-85124	22971	false
	HGL 6	151776671	162944336	37843125	49425623	-	#60FA36	11167665	11582498	-50703	8331	-50703	11116962	false
	HGL 6	162952667	163012911	37788347	37842949	+	#60FA36	60244	54602	8331	68	8331	68575	false
	HGL 6	163012979	163704520	37084670	37786437	-	#60FA36	691541	701767	68	57470	68	691609	false
	HGL 6	163761990	166530596	34317967	37036471	-	#60FA36	2768606	2718504	57470	78208	57470	2826076	false
	HGL 6	166608804	166732778	34154934	34259179	-	#60FA36	123974	104245	78208	62787	78208	202182	false
	HGL 6	166795565	183121751	18069480	34124553	-	#60FA36	16326186	16055073	62787	0	62787	16388973	false
	HGL 6	183121751	184468598	5585370	6757048	+	#60FA36	1346847	1171678	0	27182	0	1346847	false
	HGL 6	184495780	194498238	6825490	15600702	+	#60FA36	10002458	8775212	27182	43761	27182	10029640	false
	HGL 6	194541999	195600694	15562761	16350477	+	#60FA36	1058695	787716	43761	98822	43761	1102456	false
	HGL 6	195699516	195923257	17600324	17759798	-	#60FA36	223741	159474	98822	125976	98822	322563	false
	HGL 6	196049233	197605508	16417855	17600010	-	#60FA36	1556275	1182155	125976	62012	125976	1682251	false
	HGL 6	197667520	198044771	17765558	18069383	+	#60FA36	377251	303825	62012		62012	439263	false
**HSA 4**	HGL 12	554457	3863252	201356	2392872	+	#DD528A	3308795	2191516		333858			
	HGL 19	3935273	4051259	20846187	20918476	-	#0000FF	115986	72289		1582			
	HGL 19	4052841	4118987	20755433	20824442	+	#0000FF	66146	69009	1582	4870760	1582	67728	false
	HGL 12	4197110	8478611	2599205	5276121	-	#DD528A	4281501	2676916	333858	111514	333858	4615359	false
	HGL 12	8590125	8913117	2393524	2598892	-	#DD528A	322992	205368	111514	831999	111514	434506	false
	HGL 19	8989747	9058438	20755433	20824442	-	#0000FF	68691	69009	4870760	1389	4870760	4939451	false
	HGL 19	9059827	9171443	20846187	20916220	+	#0000FF	111616	70033	1389	349763	1389	113005	false
	HGL 19	9521206	9590555	20755433	20824442	-	#0000FF	69349	69009	349763	1386	349763	419112	false
	HGL 19	9591941	9710831	20846187	20918361	+	#0000FF	118890	72174	1386	112576100	1386	120276	false
	HGL 12	9745116	10310482	5277702	5693335	+	#DD528A	565366	415633	831999	88750	831999	1397365	false

	HGL 12	10399232	12352377	5753570	7418128	+	#DD528A	1953145	1664558	88750	0	88750	2041895	false
	HGL 12	12352377	12447922	7472823	7558375	-	#DD528A	95545	85552	0	19555	0	95545	false
	HGL 12	12467477	22320411	7593464	16749705	+	#DD528A	9852934	9156241	19555	52018	19555	9872489	false
	HGL 12	22372429	25580287	16847570	19759635	+	#DD528A	3207858	2912065	52018	55880	52018	3259876	false
	HGL 12	25636167	35391791	19781543	29008250	+	#DD528A	9755624	9226707	55880	70980	55880	9811504	false
	HGL 12	35462771	35683735	29050363	29262601	-	#DD528A	220964	212238	70980	611	70980	291944	false
	HGL 12	35684346	37638021	29306480	31197078	+	#DD528A	1953675	1890598	611	-50551	611	1954286	false
	HGL 12	37587470	40005993	31246147	33445343	+	#DD528A	2418523	2199196	-50551	40446	-50551	2367972	false
	HGL 12	40046439	41247237	33497508	34556387	+	#DD528A	1200798	1058879	40446	0	40446	1241244	false
	HGL 12	41247237	43434335	34614484	36940702	+	#DD528A	2187098	2326218	0	3712	0	2187098	false
	HGL 12	43438047	43522057	36941640	36999529	-	#DD528A	84010	57889	3712	917	3712	87722	false
	HGL 12	43522974	44495991	37001508	37930142	+	#DD528A	973017	928634	917	64534	917	973934	false
	HGL 12	44560525	49083563	37993536	42507411	+	#DD528A	4523038	4513875	64534	2730908	64534	4587572	false
	HGL 12	51814471	56882945	42512443	47874571	+	#DD528A	5068474	5362128	2730908	21379	2730908	7799382	false
	HGL 12	56904324	63032849	47938988	54193063	+	#DD528A	6128525	6254075	21379	50718	21379	6149904	false
	HGL 12	63083567	64014179	54194365	55129728	+	#DD528A	930612	935363	50718	20749	50718	981330	false
	HGL 12	64034928	65688042	55184110	56752728	+	#DD528A	1653114	1568618	20749	29140	20749	1673863	false
	HGL 12	65717182	66221312	56819780	57477164	+	#DD528A	504130	657384	29140	2816	29140	533270	false
	HGL 12	66224128	66288276	57478370	57576659	-	#DD528A	64148	98289	2816	56	2816	66964	false
	HGL 12	66288332	66443696	57577207	57739997	+	#DD528A	155364	162790	56	552	56	155420	false
	HGL 12	66444248	66517863	57739997	57811265	-	#DD528A	73615	71268	552	1112	552	74167	false
	HGL 12	66518975	67401298	57815102	58894387	+	#DD528A	882323	1079285	1112	62477	1112	883435	false
	HGL 12	67463775	68273573	58918067	59855779	+	#DD528A	809798	937712	62477	36812	62477	872275	false
	HGL 12	68310385	68503309	59865358	60083696	-	#DD528A	192924	218338	36812	516825	36812	229736	false
	HGL 12	69020134	69072136	61132936	61220099	+	#DD528A	52002	87163	516825	317585	516825	568827	false
	HGL 12	69389721	69443319	61161916	61220099	+	#DD528A	53598	58183	317585	144469	317585	371183	false
	HGL 12	69587788	69727584	61687465	61759887	+	#DD528A	139796	72422	144469	-45566	144469	284265	false
	HGL 12	69682018	70062042	61851375	62307868	+	#DD528A	380024	456493	-45566	-71270	-45566	334458	false
	HGL 12	69990772	70383091	62304136	62613114	+	#DD528A	392319	308978	-71270	88067	-71270	321049	false
	HGL 12	70471158	71879370	62669598	64301252	+	#DD528A	1408212	1631654	88067	62747	88067	1496279	false
	HGL 12	71942117	73456941	64340963	66078249	+	#DD528A	1514824	1737286	62747	22997	62747	1577571	false
	HGL 12	73479938	73660001	66131657	66330555	+	#DD528A	180063	198898	22997		22997	203060	false

HGL 6	73671859	73926154	77063869	77301057	+	#60FA36	254295	237188		1497			
HGL 6	73927651	74025390	77165688	77257489	-	#60FA36	97739	91801	1497	131500	1497	99236	false
HGL 6	74156890	75826467	77353335	78865801	+	#60FA36	1669577	1512466	131500	13667	131500	1801077	false
HGL 6	75840134	75904702	78975694	79044021	-	#60FA36	64568	68327	13667	7019800	13667	78235	false
HGL 7	75905334	77290768	43070491	44473720	-	#C2FB8F	1385434	1403229		86430			
HGL 7	77377198	82923099	37381410	43048493	-	#C2FB8F	5545901	5667083	86430	5100636	86430	5632331	false
HGL 6	82924502	85013071	82381141	84540723	-	#60FA36	2088569	2159582	7019800	1934	7019800	9108369	false
HGL 6	85015005	85122324	82135053	82321844	-	#60FA36	107319	186791	1934	3636	1934	109253	false
HGL 6	85125960	88078355	79139021	82080913	-	#60FA36	2952395	2941892	3636		3636	2956031	false
HGL 7	88023735	88169371	44655849	44845780	+	#C2FB8F	145636	189931	5100636	-145636	5100636	5246272	false
HGL 7	88023735	88333978	44947720	45289021	+	#C2FB8F	310243	341301	-145636	29882	-145636	164607	false
HGL 7	88363860	97890902	45342629	55338686	+	#C2FB8F	9527042	9996057	29882	303	29882	9556924	false
HGL 7	97891205	97977814	55359938	55419314	-	#C2FB8F	86609	59376	303	210	303	86912	false
HGL 7	97978024	99119403	55426144	56765431	+	#C2FB8F	1141379	1339287	210		210	1141589	false
HGL 26	99221303	99337057	10348540	10408517	-	#7FBF7F	115754	59977		-48648			
HGL 26	99288409	108771533	3576335	10289923	-	#7FBF7F	9483124	6713588	-48648	1140	-48648	9434476	false
HGL 26	108772673	112663775	628191	3521131	-	#7FBF7F	3891102	2892940	1140	50709	1140	3892242	false
HGL 26	112714484	113396998	16224	614965	-	#7FBF7F	682514	598741	50709		50709	733223	false
HGL 1	113477832	113830399	86000603	86351247	+	#13C840	352567	350644		-49588			
HGL 1	113780811	113834740	86356393	86410100	-	#13C840	53929	53707	-49588	7	-49588	4341	false
HGL 1	113834747	114513553	86410747	87091761	+	#13C840	678806	681014	7	17473	7	678813	false
HGL 1	114531026	115154917	87148424	87832201	+	#13C840	623891	683777	17473	-686	17473	641364	false
HGL 1	115154231	115215012	87873341	87933137	-	#13C840	60781	59796	-686	675	-686	60095	false
HGL 1	115215687	118418020	87939793	91499383	+	#13C840	3202333	3559590	675	255194	675	3203008	false
HGL 1	118673214	119239527	91500469	92030450	+	#13C840	566313	529981	255194	234519	255194	821507	false
HGL 1	119474046	120081476	92030558	92644066	+	#13C840	607430	613508	234519	-22659	234519	841949	false
HGL 1	120058817	125290328	92680591	98466147	+	#13C840	5231511	5785556	-22659	12304	-22659	5208852	false
HGL 3	121937057	122001589	40933260	40987942	-	#1240C3	64532	54682		276543			
HGL 3	122278132	122454120	40987942	41161240	-	#1240C3	175988	173298	276543		276543	452531	false
HGL 19	122286931	122359774	14932034	14991819	+	#0000FF	72843	59785	112576100	3917	112576100	112648943	false
HGL 19	122363691	122453936	14842043	14920546	-	#0000FF	90245	78503	3917		3917	94162	false
HGL 1	125302632	129045507	98759530	102592307	+	#13C840	3742875	3832777	12304	61281	12304	3755179	false

	HGL 1	129106788	140330595	102616881	113212490	+	#13C840	11223807	10595609	61281	-93820	61281	11285088	false
	HGL 1	140236775	140347254	98494676	98585883	-	#13C840	110479	91207	-93820	20421	-93820	16659	false
	HGL 1	140367675	140612801	113282238	113469179	-	#13C840	245126	186941	20421	6806	20421	265547	false
	HGL 1	140619607	143524710	113510624	116290657	+	#13C840	2905103	2780033	6806	50368	6806	2911909	false
	HGL 1	143575078	143726270	116467944	116562230	+	#13C840	151192	94286	50368	56964	50368	201560	false
	HGL 1	143783234	143908830	116564534	116634761	+	#13C840	125596	70227	56964	35953	56964	182560	false
	HGL 1	143944783	144022694	116577350	116634862	+	#13C840	77911	57512	35953	32033	35953	113864	false
	HGL 1	144054727	147591526	116577350	119888047	+	#13C840	3536799	3310697	32033	25083	32033	3568832	false
	HGL 1	147616609	147688494	113212556	113282225	+	#13C840	71885	69669	25083	25225	25083	96968	false
	HGL 1	147713719	148345072	120221245	120804518	+	#13C840	631353	583273	25225	140012	25225	656578	false
	HGL 1	148485084	154550904	120804934	126623353	+	#13C840	6065820	5818419	140012	0	140012	6205832	false
	HGL 1	154550904	154622851	126623400	126687002	-	#13C840	71947	63602	0	36633	0	71947	false
	HGL 1	154659484	154765434	116328850	116445880	+	#13C840	105950	117030	36633	-32075	36633	142583	false
	HGL 1	154733359	158425988	126913028	130294597	-	#13C840	3692629	3381569	-32075	51667	-32075	3660554	false
	HGL 1	158477655	158667516	126735840	126889743	-	#13C840	189861	153903	51667	2263	51667	241528	false
	HGL 1	158669779	160965552	130356100	132265343	+	#13C840	2295773	1909243	2263	16362	2263	2298036	false
	HGL 1	160981914	161085747	132269287	132325575	-	#13C840	103833	56288	16362	3516	16362	120195	false
	HGL 1	161089263	162125322	132338739	133260703	+	#13C840	1036059	921964	3516		3516	1039575	false
	HGL 11	162576717	164496606	16920006	18712573	+	#7F00FF	1919889	1792567		0			
	HGL 11	164496606	164632174	18713072	18852948	-	#7F00FF	135568	139876	0	4054	0	135568	false
	HGL 11	164636228	164958677	18853063	19144599	+	#7F00FF	322449	291536	4054	72628	4054	326503	false
	HGL 11	165031305	168188286	19122322	21864725	+	#7F00FF	3156981	2742403	72628	26613	72628	3229609	false
	HGL 11	168214899	168320960	21871693	21999736	-	#7F00FF	106061	128043	26613	-106041	26613	132674	false
	HGL 11	168214919	168337213	22057353	22159567	+	#7F00FF	122294	102214	-106041	20646	-106041	16253	false
	HGL 11	168357859	168475757	21864879	21996883	-	#7F00FF	117898	132004	20646	-117898	20646	138544	false
	HGL 11	168357859	170932574	22061842	24527018	+	#7F00FF	2574715	2465176	-117898	4811	-117898	2456817	false
	HGL 11	170937385	174996264	24588724	28579881	+	#7F00FF	4058879	3991157	4811	148058	4811	4063690	false
	HGL 11	175144322	178198251	28581117	31307562	+	#7F00FF	3053929	2726445	148058	8644	148058	3201987	false
	HGL 11	178206895	178375245	31307562	31487600	-	#7F00FF	168350	180038	8644	11460	8644	176994	false
	HGL 11	178386705	186583016	31487600	39433211	+	#7F00FF	8196311	7945611	11460	634	11460	8207771	false
	HGL 11	186583650	187090425	39433281	39990996	-	#7F00FF	506775	557715	634	3768	634	507409	false
	HGL 11	187094193	188340037	40015946	41388577	+	#7F00FF	1245844	1372631	3768	3524	3768	1249612	false

	HGL 11	188343561	188423087	41390019	41492302	-	#7F00FF	79526	102283	3524	41	3524	83050	false
	HGL 11	188423128	189181117	41493427	42276936	+	#7F00FF	757989	783509	41	47109	41	758030	false
	HGL 11	189228226	189633532	42335950	42727420	+	#7F00FF	405306	391470	47109	67601	47109	452415	false
	HGL 11	189701133	189906408	42791234	43066400	+	#7F00FF	205275	275166	67601		67601	272876	false
**HSA 5**	HGL 10	167066	769994	17737	431877	+	#00007F	602928	414140		10			
	HGL 10	770004	1551887	368944	779800	+	#00007F	781883	410856	10	98372	10	781893	false
	HGL 10	1650259	6509306	782851	4259354	+	#00007F	4859047	3476503	98372	57699	98372	4957419	false
	HGL 10	6567005	12813648	4265998	9845683	+	#00007F	6246643	5579685	57699	-4723405	57699	6304342	false
	HGL 10	8090243	8154310	6161976	6231093	+	#00007F	64067	69117	-4723405	-54288	-4723405	-4659338	true
	HGL 10	8100022	8159754	5640535	5697968	-	#00007F	59732	57433	-54288	17832	-54288	5444	false
	HGL 10	8177586	8248135	6077575	6155245	-	#00007F	70549	77670	17832	107418	17832	88381	false
	HGL 10	8355553	8411632	6486462	6569724	+	#00007F	56079	83262	107418	83904	107418	163497	false
	HGL 10	8495536	8642512	5723734	5848145	-	#00007F	146976	124411	83904	128829	83904	230880	false
	HGL 10	8771341	9048012	5494426	5652550	-	#00007F	276671	158124	128829	3765660	128829	405500	false
	HGL 10	12813672	17529960	9926632	14180904	+	#00007F	4716288	4254272	3765660	75210	3765660	8481948	false
	HGL 10	17605170	19099192	14180026	15519160	+	#00007F	1494022	1339134	75210	51882	75210	1569232	false
	HGL 10	19151074	20793179	15558022	16977635	+	#00007F	1642105	1419613	51882	130620	51882	1693987	false
	HGL 10	20923799	21452985	17054754	17498464	+	#00007F	529186	443710	130620	90972	130620	659806	false
	HGL 10	21543957	22142396	17515622	18071731	+	#00007F	598439	556109	90972	64855	90972	689411	false
	HGL 10	22207251	29401685	18132581	24284606	+	#00007F	7194434	6152025	64855	54369	64855	7259289	false
	HGL 10	29456054	33988791	24284692	28565372	+	#00007F	4532737	4280680	54369	-20119	54369	4587106	false
	HGL 10	33968672	34060253	28704741	28788230	+	#00007F	91581	83489	-20119	8489	-20119	71462	false
	HGL 10	34068742	34173691	16884375	16977635	-	#00007F	104949	93260	8489	234550	8489	113438	false
	HGL 10	34408241	36072170	28788447	30239348	+	#00007F	1663929	1450901	234550	-131607	234550	1898479	false
	HGL 10	35940563	38032150	30228356	32216881	+	#00007F	2091587	1988525	-131607	-63729	-131607	1959980	false
	HGL 10	37968421	38048058	32177983	32264596	-	#00007F	79637	86613	-63729	20	-63729	15908	false
	HGL 10	38048078	42890698	32268965	37108347	+	#00007F	4842620	4839382	20	212993	20	4842640	false
	HGL 10	43103691	45911317	37232343	39862860	+	#00007F	2807626	2630517	212993	4364241	212993	3020619	false
	HGL 10	50275558	50485276	39952054	40187981	+	#00007F	209718	235927	4364241	52781	4364241	4573959	false
	HGL 10	50538057	54611691	40220051	44444436	+	#00007F	4073634	4224385	52781	31598	52781	4126415	false
	HGL 10	54643289	64404782	44503412	54439914	+	#00007F	9761493	9936502	31598	50612	31598	9793091	false
	HGL 10	64455394	69136586	54450031	59712138	+	#00007F	4681192	5262107	50612	13172	50612	4731804	false

	HGL 10	69149758	69623795	59869704	60306662	+	#00007F	474037	436958	13172	34464	13172	487209	false
	HGL 10	69658259	69778888	17359140	17441839	-	#00007F	120629	82699	34464	11089	34464	155093	false
	HGL 10	69789977	69894939	17366490	17441839	+	#00007F	104962	75349	11089	130261	11089	116051	false
	HGL 10	70025200	70128504	60295814	60360418	-	#00007F	103304	64604	130261	109496	130261	233565	false
	HGL 10	70238000	70358699	17359140	17441839	-	#00007F	120699	82699	109496	51074	109496	230195	false
	HGL 10	70409773	70478930	60228080	60306662	+	#00007F	69157	78582	51074	55454	51074	120231	false
	HGL 10	70534384	70640256	17366490	17441839	-	#00007F	105872	75349	55454	11107	55454	161326	false
	HGL 10	70651363	70770821	17359140	17441839	+	#00007F	119458	82699	11107	129797	11107	130565	false
	HGL 10	70900618	71084787	60217196	60360418	-	#00007F	184169	143222	129797	153555	129797	313966	false
	HGL 10	71238342	71358139	17359140	17441839	-	#00007F	119797	82699	153555	92375	153555	273352	false
	HGL 10	71450514	73481897	60360419	62367080	+	#00007F	2031383	2006661	92375	12082	92375	2123758	false
	HGL 10	73493979	74888042	63706948	65214477	+	#00007F	1394063	1507529	12082	-9995	12082	1406145	false
	HGL 10	74878047	75058314	63350288	63544617	+	#00007F	180267	194329	-9995	20	-9995	170272	false
	HGL 10	75058334	75482080	62695750	63281328	-	#00007F	423746	585578	20	-24787	20	423766	false
	HGL 10	75457293	75551919	63613711	63703123	+	#00007F	94626	89412	-24787	-10899	-24787	69839	false
	HGL 10	75541020	75707455	62515455	62693961	-	#00007F	166435	178506	-10899	-32260	-10899	155536	false
	HGL 10	75675195	80086509	65635258	70100096	+	#00007F	4411314	4464838	-32260	100914663	-32260	4379054	false
	HGL 11	80116042	91193899	113672175	122742221	-	#7F00FF	11077857	9070046		59359			
	HGL 11	91253258	99236845	106336987	113672175	-	#7F00FF	7983587	7335188	59359	75258	59359	8042946	false
	HGL 11	99312103	99467738	105980586	106173753	-	#7F00FF	155635	193167	75258	114845	75258	230893	false
	HGL 11	99582583	100374684	105250131	106067655	+	#7F00FF	792101	817524	114845	27302	114845	906946	false
	HGL 11	100401986	100468206	105069930	105141907	-	#7F00FF	66220	71977	27302	66030	27302	93522	false
	HGL 11	100534236	112940839	93235440	105017458	-	#7F00FF	12406603	11782018	66030	8986211	66030	12472633	false
	HGL 5	112940839	115691258	34342328	37216951	+	#2DCDC1	2750419	2874623		14174			
	HGL 5	115705432	117709773	37275581	39415322	+	#2DCDC1	2004341	2139741	14174	14100	14174	2018515	false
	HGL 5	117723873	117957997	39431664	39660165	-	#2DCDC1	234124	228501	14100	521	14100	248224	false
	HGL 5	117958518	119636394	39660195	41434871	+	#2DCDC1	1677876	1774676	521	12917	521	1678397	false
	HGL 5	119649311	119864423	41438950	41665134	-	#2DCDC1	215112	226184	12917	567	12917	228029	false
	HGL 5	119864990	120374635	41665134	42274078	-	#2DCDC1	509645	608944	567	4	567	510212	false
	HGL 5	120374639	120924701	42290065	42809516	+	#2DCDC1	550062	519451	4	50671	4	550066	false
	HGL 5	120975372	121071632	42851193	42972783	+	#2DCDC1	96260	121590	50671	36522	50671	146931	false
	HGL 5	121108154	121334625	43023016	43244194	+	#2DCDC1	226471	221178	36522	49573	36522	262993	false

	HGL 5	121384198	121499344	43296200	43403360	+	#2DCDC1	115146	107160	49573	9638114	49573	164719	false
	HGL 11	121927050	122040999	69466600	69560580	-	#7F00FF	113949	93980	8986211	14227	8986211	9100160	false
	HGL 11	122055226	131038347	70372771	80253982	+	#7F00FF	8983121	9881211	14227	6850941	14227	8997348	false
	HGL 5	131137458	134735661	2528128	5854029	+	#2DCDC1	3598203	3325901	9638114	-30390	9638114	13236317	false
	HGL 5	134705271	135730270	31117332	32161296	+	#2DCDC1	1024999	1043964	-30390	82986	-30390	994609	false
	HGL 5	135813256	137889254	32161432	34342322	+	#2DCDC1	2075998	2180890	82986	13111597	82986	2158984	false
	HGL 11	137889288	139129804	92060882	93235431	-	#7F00FF	1240516	1174549	6850941	-41020	6850941	8091457	false
	HGL 11	139088784	150809278	80447650	92056281	-	#7F00FF	11720494	11608631	-41020	83831	-41020	11679474	false
	HGL 11	150893109	150986473	80310626	80382250	-	#7F00FF	93364	71624	83831		83831	177195	false
	HGL 5	151000851	151354939	2176585	2528019	-	#2DCDC1	354088	351434	13111597	43216	13111597	13465685	false
	HGL 5	151398155	152456968	5878385	6834143	+	#2DCDC1	1058813	955758	43216	-68922	43216	1102029	false
	HGL 5	152388046	157129287	6849618	11145002	+	#2DCDC1	4741241	4295384	-68922	-126467	-68922	4672319	false
	HGL 5	157002820	168520265	11145186	21975151	+	#2DCDC1	11517445	10829965	-126467	6928	-126467	11390978	false
	HGL 5	168527193	171395297	22031710	24754497	+	#2DCDC1	2868104	2722787	6928	-60525	6928	2875032	false
	HGL 5	171334772	175999482	24756671	29439534	+	#2DCDC1	4664710	4682863	-60525	292354	-60525	4604185	false
	HGL 5	176291836	177613302	29595793	31019471	+	#2DCDC1	1321466	1423678	292354	451220	292354	1613820	false
	HGL 5	178064522	178634149	1704047	2146352	-	#2DCDC1	569627	442305	451220	233816	451220	1020847	false
	HGL 5	178867965	179627338	909183	1559712	-	#2DCDC1	759373	650529	233816	50952	233816	993189	false
	HGL 5	179678290	180669574	211197	904055	-	#2DCDC1	991284	692858	50952		50952	1042236	false
	HGL 10	181001172	181093451	83551801	83648508	-	#00007F	92279	96707	100914663	30458	100914663	101006942	false
	HGL 10	181123909	181251982	83301306	83444765	-	#00007F	128073	143459	30458		30458	158531	false
**HSA 6**	HGL 4	181356	5016062	1395764	5299798	+	#0C404C	4834706	3904034		50975			
	HGL 4	5067037	5805913	5334206	5983767	+	#0C404C	738876	649561	50975	51194	50975	789851	false
	HGL 4	5857107	25970737	6009302	24464968	+	#0C404C	20113630	18455666	51194	-8451	51194	20164824	false
	HGL 4	25962286	26349859	24580336	24877504	+	#0C404C	387573	297168	-8451	126785	-8451	379122	false
	HGL 4	26476644	26666992	25141134	25271889	+	#0C404C	190348	130755	126785	423029	126785	317133	false
	HGL 4	27090021	27167005	1311901	1362579	-	#0C404C	76984	50678	423029	253122	423029	500013	false
	HGL 4	27420127	27620480	1145606	1253996	-	#0C404C	200353	108390	253122	163130	253122	453475	false
	HGL 4	27783610	27891792	1022660	1103588	-	#0C404C	108182	80928	163130	317851	163130	271312	false
	HGL 4	28209643	28367232	814444	951615	+	#0C404C	157589	137171	317851	63497	317851	475440	false
	HGL 4	28430729	28593341	706478	813586	-	#0C404C	162612	107108	63497		63497	226109	false
	HGL 13	29569284	29683416	10928	87941	+	#097F8D	114132	77013		223111			

	HGL 13	29906527	29976269	736365	809804	-	#097F8D	69742	73439	223111	68820	223111	292853	false
	HGL 13	30045089	30265604	106311	272387	+	#097F8D	220515	166076	68820	276677	68820	289335	false
	HGL 13	30542281	30977925	332711	653796	+	#097F8D	435644	321085	276677	56732	276677	712321	false
	HGL 13	31034657	31181375	653787	722475	+	#097F8D	146718	68688	56732	219709	56732	203450	false
	HGL 13	31401084	31473165	736365	807539	+	#097F8D	72081	71174	219709	22130	219709	291790	false
	HGL 13	31495295	32230431	736365	1374398	+	#097F8D	735136	638033	22130	181840	22130	757266	false
	HGL 13	32412271	33325085	1385294	1973713	+	#097F8D	912814	588419	181840	58462	181840	1094654	false
	HGL 13	33383547	34155072	2047397	2557366	+	#097F8D	771525	509969	58462	70003	58462	829987	false
	HGL 13	34225075	41218101	2564872	7876535	+	#097F8D	6993026	5311663	70003	50086	70003	7063029	false
	HGL 13	41268187	48795326	7883773	12764208	+	#097F8D	7527139	4880435	50086	89945	50086	7577225	false
	HGL 13	48885271	49049403	12764520	12826731	+	#097F8D	164132	62211	89945		89945	254077	false
	HGL 3	49553417	49790184	84425074	84621284	+	#1240C3	236767	196210		-96537			
	HGL 3	49693647	49790895	84631848	84726676	-	#1240C3	97248	94828	-96537	-51709	-96537	711	false
	HGL 3	49739186	52688278	84810215	87844050	+	#1240C3	2949092	3033835	-51709	53220	-51709	2897383	false
	HGL 3	52741498	52875184	87858968	87922546	+	#1240C3	133686	63578	53220	-129114	53220	186906	false
	HGL 3	52746070	52914562	87854064	87945204	+	#1240C3	168492	91140	-129114	38243	-129114	39378	false
	HGL 3	52952805	56344605	35481258	39433616	-	#1240C3	3391800	3952358	38243	23748573	38243	3430043	false
	HGL 22	56423621	57436697	35611514	36580760	+	#00FFFF	1013076	969246		94776			
	HGL 22	57531473	57663378	36676285	36848424	+	#00FFFF	131905	172139	94776	303675	94776	226681	false
	HGL 22	57967053	58042502	36951870	37051222	+	#00FFFF	75449	99352	303675	2198361	303675	379124	false
	HGL 22	60240863	60327650	36477184	36576853	+	#00FFFF	86787	99669	2198361	96779	2198361	2285148	false
	HGL 22	60424429	60562216	36676285	36848424	+	#00FFFF	137787	172139	96779	51598	96779	234566	false
	HGL 22	60613814	60793721	36887189	37050970	+	#00FFFF	179907	163781	51598	744250	51598	231505	false
	HGL 22	61537971	62834249	37460197	38645789	+	#00FFFF	1296278	1185592	744250	53361	744250	2040528	false
	HGL 22	62887610	62984060	38645929	38728549	+	#00FFFF	96450	82620	53361	16240	53361	149811	false
	HGL 22	63000300	67843072	38809439	42968664	+	#00FFFF	4842772	4159225	16240	89204	16240	4859012	false
	HGL 22	67932276	69806771	42999464	44799704	+	#00FFFF	1874495	1800240	89204	50821	89204	1963699	false
	HGL 22	69857592	73246969	44800166	47965681	+	#00FFFF	3389377	3165515	50821	145068	50821	3440198	false
	HGL 22	73392037	78477974	49548304	54236282	-	#00FFFF	5085937	4687978	145068	798	145068	5231005	false
	HGL 22	78478772	78581925	49406109	49548304	+	#00FFFF	103153	142195	798	251726	798	103951	false
	HGL 22	78833651	80148526	48076390	49310267	-	#00FFFF	1314875	1233877	251726		251726	1566601	false
	HGL 3	80093178	85880505	78452678	84360497	-	#1240C3	5787327	5907819	23748573	63159	23748573	29535900	false

	HGL 3	85943664	86460192	77916549	78381605	-	#1240C3	516528	465056	63159	55136	63159	579687	false
	HGL 3	86515328	89821309	74349753	77916546	-	#1240C3	3305981	3566793	55136	60341	55136	3361117	false
	HGL 3	89881650	95020635	68893755	74272345	-	#1240C3	5138985	5378590	60341	54249	60341	5199326	false
	HGL 3	95074884	96216479	67418525	68893707	-	#1240C3	1141595	1475182	54249	20006	54249	1195844	false
	HGL 3	96236485	102948672	60094399	67358570	-	#1240C3	6712187	7264171	20006	37492	20006	6732193	false
	HGL 3	102986164	103066793	59870892	60008594	+	#1240C3	80629	137702	37492	27328	37492	118121	false
	HGL 3	103094121	105426685	57639608	59870662	-	#1240C3	2332564	2231054	27328	0	27328	2359892	false
	HGL 3	105426685	109545058	52982524	57174241	-	#1240C3	4118373	4191717	0	108577	0	4118373	false
	HGL 3	109653635	110439298	52205222	52982407	-	#1240C3	785663	777185	108577	80155	108577	894240	false
	HGL 3	110519453	111030728	51725932	52196693	-	#1240C3	511275	470761	80155	26322	80155	591430	false
	HGL 3	111057050	111266964	51450141	51666969	-	#1240C3	209914	216828	26322	-16071	26322	236236	false
	HGL 3	111250893	112044092	39618469	40499136	-	#1240C3	793199	880667	-16071	-841	-16071	777128	false
	HGL 3	112043251	112139994	51078596	51236880	-	#1240C3	96743	158284	-841	73928	-841	95902	false
	HGL 3	112213922	112555039	50726715	51078074	-	#1240C3	341117	351359	73928	171	73928	415045	false
	HGL 3	112555210	112685325	50580150	50722496	+	#1240C3	130115	142346	171	1706	171	130286	false
	HGL 3	112687031	114994077	48161888	50580150	-	#1240C3	2307046	2418262	1706	3730	1706	2308752	false
	HGL 3	114997807	116593592	46198222	48110180	-	#1240C3	1595785	1911958	3730	20996	3730	1599515	false
	HGL 3	116614588	121619508	131528454	135286592	-	#1240C3	5004920	3758138	20996	56963	20996	5025916	false
	HGL 3	121676471	124888436	128872129	131494507	-	#1240C3	3211965	2622378	56963	12225	56963	3268928	false
	HGL 3	124900661	127592200	126496735	128794847	-	#1240C3	2691539	2298112	12225	-58429	12225	2703764	false
	HGL 3	127533771	131424166	122925831	126493366	-	#1240C3	3890395	3567535	-58429	3569	-58429	3831966	false
	HGL 3	131427735	132569081	121850600	122862815	-	#1240C3	1141346	1012215	3569	-1707	3569	1144915	false
	HGL 3	132567374	132782452	57501279	57637787	+	#1240C3	215078	136508	-1707	-212115	-1707	213371	false
	HGL 3	132570337	133550122	120774810	121761124	-	#1240C3	979785	986314	-212115	-765879	-212115	767670	false
	HGL 3	132784243	132841238	57185400	57236448	+	#1240C3	56995	51048	-765879	333142	-765879	-708884	true
	HGL 3	133174380	133342903	57297520	57451635	+	#1240C3	168523	154115	333142	208113	333142	501665	false
	HGL 3	133551016	146859923	108608978	120766344	+	#1240C3	13308907	12157366	208113	-272029	208113	13517020	false
	HGL 3	146587894	149876545	95617903	98888102	+	#1240C3	3288651	3270199	-272029	197081	-272029	3016622	false
	HGL 3	150073626	155856514	98984124	104654410	+	#1240C3	5782888	5670286	197081	16872	197081	5979969	false
	HGL 3	155873386	158208015	104720718	107029815	+	#1240C3	2334629	2309097	16872	5904	16872	2351501	false
	HGL 3	158213919	159677318	107083927	108424848	-	#1240C3	1463399	1340921	5904	4806	5904	1469303	false
	HGL 3	159682124	160524411	87959612	88868199	+	#1240C3	842287	908587	4806	7007	4806	847093	false

	HGL 3	160531418	160665874	88828984	88879692	+	#1240C3	134456	50708	7007	34035	7007	141463	false
	HGL 3	160699909	160758468	88821958	88881401	-	#1240C3	58559	59443	34035	156057	34035	92594	false
	HGL 3	160914525	163188055	88868222	91231184	+	#1240C3	2273530	2362962	156057	249377	156057	2429587	false
	HGL 3	163437432	167247669	91231194	95072032	+	#1240C3	3810237	3840838	249377	3775	249377	4059614	false
	HGL 3	167251444	167393888	94945714	95084103	-	#1240C3	142444	138389	3775	4970	3775	146219	false
	HGL 3	167398858	167588915	95093053	95252844	+	#1240C3	190057	159791	4970	53122	4970	195027	false
	HGL 3	167642037	168060722	95253089	95611476	+	#1240C3	418685	358387	53122	90239	53122	471807	false
	HGL 3	168150961	169344043	34392619	35334216	-	#1240C3	1193082	941597	90239	32	90239	1283321	false
	HGL 3	169344075	170601377	33209772	34329405	-	#1240C3	1257302	1119633	32		32	1257334	false
**HSA 7**	HGL 3	31731	2355873	5459641	7196317	-	#1240C3	2324142	1736676		-1796			
	HGL 3	2354077	2531271	2488659	2679549	+	#1240C3	177194	190890	-1796	0	-1796	175398	false
	HGL 3	2531271	2671725	2679555	2797190	-	#1240C3	140454	117635	0	1075	0	140454	false
	HGL 3	2672800	4964859	2788014	4697658	+	#1240C3	2292059	1909644	1075	65457	1075	2293134	false
	HGL 3	5030316	5161137	5250196	5414058	-	#1240C3	130821	163862	65457	13052	65457	196278	false
	HGL 3	5174189	5849055	4698271	5309679	+	#1240C3	674866	611408	13052	137208	13052	687918	false
	HGL 3	5986263	6731631	1737942	2472096	+	#1240C3	745368	734154	137208	22919620	137208	882576	false
	HGL 21	7000233	10605521	61119650	64757386	-	#0C8017	3605288	3637736		1311			
	HGL 21	10606832	12506036	59180218	61069490	-	#0C8017	1899204	1889272	1311	14545	1311	1900515	false
	HGL 21	12520581	22489717	18062980	27247653	+	#0C8017	9969136	9184673	14545	51309	14545	9983681	false
	HGL 21	22541026	29648663	27247653	34454850	+	#0C8017	7107637	7207197	51309	95448	51309	7158946	false
	HGL 3	29651251	29742502	10246408	10312704	-	#1240C3	91251	66296	22919620	-91251	22919620	23010871	false
	HGL 3	29651251	29742504	7279551	7359493	-	#1240C3	91253	79942	-91251	5308761	-91251	2	false
	HGL 21	29744111	30748704	34429425	35411650	+	#0C8017	1004593	982225	95448	4	95448	1100041	false
	HGL 21	30748708	32480205	35490565	37223869	+	#0C8017	1731497	1733304	4	44589574	4	1731501	false
	HGL 9	32475965	32597071	92054078	92123842	-	#6E0025	121106	69764		270479			
	HGL 9	32867550	33064608	91902934	92053268	-	#6E0025	197058	150334	270479	63811	270479	467537	false
	HGL 9	33128419	33412465	93533919	93742221	-	#6E0025	284046	208302	63811	-71242	63811	347857	false
	HGL 9	33341223	33496941	93360313	93529526	-	#6E0025	155718	169213	-71242	-51630	-71242	84476	false
	HGL 9	33445311	33614329	93219301	93353905	-	#6E0025	169018	134604	-51630	-75867	-51630	117388	false
	HGL 9	33538462	33820546	93007432	93176203	-	#6E0025	282084	168771	-75867	312707	-75867	206217	false
	HGL 9	34133253	34950485	92374098	93007115	-	#6E0025	817232	633017	312707	57173	312707	1129939	false
	HGL 9	35007658	35073302	92271574	92332931	-	#6E0025	65644	61357	57173	22248034	57173	122817	false

	HGL 3	35051265	35185595	10243991	10311044	+	#1240C3	134330	67053	5308761	-113943	5308761	5443091	false
	HGL 3	35071652	35910181	7264598	7993814	+	#1240C3	838529	729216	-113943	35142	-113943	724586	false
	HGL 3	35945323	38366548	7968684	10086198	+	#1240C3	2421225	2117514	35142	-134717	35142	2456367	false
	HGL 3	38231831	38369656	10155713	10235911	+	#1240C3	137825	80198	-134717	-129995	-134717	3108	false
	HGL 3	38239661	38370494	10313403	10369446	+	#1240C3	130833	56043	-129995	41	-129995	838	false
	HGL 3	38370535	39755333	10445778	11817787	+	#1240C3	1384798	1372009	41	128252	41	1384839	false
	HGL 3	39883585	43808010	11818320	15610921	+	#1240C3	3924425	3792601	128252	58301	128252	4052677	false
	HGL 3	43866311	43960172	25029740	25111339	+	#1240C3	93861	81599	58301	83728	58301	152162	false
	HGL 3	44043900	45280882	25111358	25909601	+	#1240C3	1236982	798243	83728	123832	83728	1320710	false
	HGL 3	45404714	45724561	25953814	26158322	+	#1240C3	319847	204508	123832	133682	123832	443679	false
	HGL 3	45858243	46446486	26158323	26589959	+	#1240C3	588243	431636	133682	-32599	133682	721925	false
	HGL 3	46413887	46505002	26546033	26608602	-	#1240C3	91115	62569	-32599	35419	-32599	58516	false
	HGL 3	46540421	47590041	26623430	27303262	+	#1240C3	1049620	679832	35419	55601	35419	1085039	false
	HGL 3	47645642	51358487	27303262	29811754	+	#1240C3	3712845	2508492	55601	90661	55601	3768446	false
	HGL 3	51449148	53183412	29830011	31102708	+	#1240C3	1734264	1272697	90661	6012	90661	1824925	false
	HGL 3	53189424	53677142	31185795	31540845	-	#1240C3	487718	355050	6012	195655	6012	493730	false
	HGL 3	53872797	54097278	31542475	31725446	+	#1240C3	224481	182971	195655	47836	195655	420136	false
	HGL 3	54145114	55586310	31794891	32700556	+	#1240C3	1441196	905665	47836	208975	47836	1489032	false
	HGL 3	55795285	56114934	24786268	24985018	-	#1240C3	319649	198750	208975	12549	208975	528624	false
	HGL 3	56127483	56226019	21247825	21350323	-	#1240C3	98536	102498	12549	9500812	12549	111085	false
	HGL 3	65726831	65976373	24673410	24846456	-	#1240C3	249542	173046	9500812	161556	9500812	9750354	false
	HGL 3	66137929	66363875	24482398	24642770	-	#1240C3	225946	160372	161556	54775	161556	387502	false
	HGL 3	66418650	66469974	24379495	24442341	+	#1240C3	51324	62846	54775	270273	54775	106099	false
	HGL 3	66740247	66813390	24379495	24448899	-	#1240C3	73143	69404	270273	105044	270273	343416	false
	HGL 3	66918434	67088547	24237948	24377299	-	#1240C3	170113	139351	105044	251375	105044	275157	false
	HGL 3	67339922	72605335	19796411	24136230	+	#1240C3	5265413	4339819	251375	322884	251375	5516788	false
	HGL 3	72928219	72991477	18637969	18736167	+	#1240C3	63258	98198	322884	181658	322884	386142	false
	HGL 3	73173135	73280569	19587650	19673334	+	#1240C3	107434	85684	181658	5874	181658	289092	false
	HGL 3	73286443	74852673	18643690	19687299	+	#1240C3	1566230	1043609	5874	70957	5874	1572104	false
	HGL 3	74923630	75219536	19587650	19776402	-	#1240C3	295906	188752	70957	154086	70957	366863	false
	HGL 3	75373622	75439390	18637969	18736167	-	#1240C3	65768	98198	154086	91068	154086	219854	false
	HGL 3	75530458	76070349	18219998	18614325	-	#1240C3	539891	394327	91068	107668	91068	630959	false

	HGL 3	76178017	76516704	17966351	18219998	-	#1240C3	338687	253647	107668	21457679	107668	446355	false
	HGL 21	77069779	86044390	1686065	9083675	-	#0C8017	8974611	7397610	44589574	907	44589574	53564185	false
	HGL 21	86045297	86372744	1504044	1685875	+	#0C8017	327447	181831	907	1334	907	328354	false
	HGL 21	86374078	87246819	823665	1504044	-	#0C8017	872741	680379	1334	69114	1334	874075	false
	HGL 21	87315933	87497811	645465	823629	-	#0C8017	181878	178164	69114	5346	69114	250992	false
	HGL 21	87503157	87601947	76483289	76638832	+	#0C8017	98790	155543	5346	-98132	5346	104136	false
	HGL 21	87503815	90105728	72600080	75320343	-	#0C8017	2601913	2720263	-98132	56906	-98132	2503781	false
	HGL 21	90162634	93367683	69222011	72477407	-	#0C8017	3205049	3255396	56906	51193	56906	3261955	false
	HGL 21	93418876	97873787	64757498	69198002	-	#0C8017	4454911	4440504	51193	4818080	51193	4506104	false
	HGL 3	97974383	98259188	1360064	1617812	-	#1240C3	284805	257748	21457679	8152	21457679	21742484	false
	HGL 3	98267340	99639392	2975	1228774	-	#1240C3	1372052	1225799	8152	8273	8152	1380204	false
	HGL 3	99647665	99866637	15800153	15939583	-	#1240C3	218972	139430	8273	-49104	8273	227245	false
	HGL 3	99817533	99872710	15851276	15910335	+	#1240C3	55177	59059	-49104	-16720	-49104	6073	false
	HGL 3	99855990	100137734	15910394	16248787	+	#1240C3	281744	338393	-16720	1880	-16720	265024	false
	HGL 3	100139614	100215613	16300306	16366142	+	#1240C3	75999	65836	1880	122575	1880	77879	false
	HGL 3	100338188	102326821	16341290	17812938	+	#1240C3	1988633	1471648	122575	60873	122575	2111208	false
	HGL 3	102387694	102526158	17815541	17943710	+	#1240C3	138464	128169	60873	639805	60873	199337	false
	HGL 21	102691867	103150919	8985998	9360864	+	#0C8017	459052	374866	4818080	138181	4818080	5277132	false
	HGL 3	103165963	103279708	10243991	10311044	+	#1240C3	113745	67053	639805	-97462	639805	753550	false
	HGL 3	103182246	103288790	7283543	7376214	+	#1240C3	106544	92671	-97462		-97462	9082	false
	HGL 21	103289100	112497210	9395456	18062911	+	#0C8017	9208110	8667455	138181	66	138181	9346291	false
	HGL 21	112497276	118830002	52838118	59180201	-	#0C8017	6332726	6342083	66	15811	66	6332792	false
	HGL 21	118845813	128460230	43009885	52780628	-	#0C8017	9614417	9770743	15811	203673	15811	9630228	false
	HGL 21	128663903	134631335	37336400	42992811	-	#0C8017	5967432	5656411	203673		203673	6171105	false
	HGL 24	134503076	134990113	36172931	36627140	+	#F50241	487037	454209	46881822	148260	46881822	47368859	false
	HGL 24	135138373	138044555	36634736	39477688	+	#F50241	2906182	2842952	148260	21472	148260	3054442	false
	HGL 24	138066027	142099900	39531925	43202020	+	#F50241	4033873	3670095	21472	-32912	21472	4055345	false
	HGL 24	142066988	142297012	43246460	43553861	+	#F50241	230024	307401	-32912		-32912	197112	false
	HGL 22	142312535	142633174	54695230	54829042	+	#00FFFF	320639	133812		-9176			
	HGL 22	142623998	142864473	54841060	55044974	+	#00FFFF	240475	203914	-9176	-27178	-9176	231299	false
	HGL 22	142837295	143015437	55044974	55201915	-	#00FFFF	178142	156941	-27178	-122950	-27178	150964	false
	HGL 22	142892487	143147612	55316384	55555235	+	#00FFFF	255125	238851	-122950	-18874	-122950	132175	false

	HGL 22	143128738	143569308	55617122	56087057	+	#00FFFF	440570	469935	-18874	148411	-18874	421696	false
	HGL 22	143717719	143807701	56033827	56111681	-	#00FFFF	89982	77854	148411	11296	148411	238393	false
	HGL 22	143818997	144053939	56111671	56295539	+	#00FFFF	234942	183868	11296	133003	11296	246238	false
	HGL 22	144186942	144262850	56322102	56382417	-	#00FFFF	75908	60315	133003	33338	133003	208911	false
	HGL 22	144296188	149888344	56329551	61088922	+	#00FFFF	5592156	4759371	33338	89800	33338	5625494	false
	HGL 22	149978144	150068223	63893788	63962383	-	#00FFFF	90079	68595	89800	252539	89800	179879	false
	HGL 22	150320762	152640163	61088922	62840291	+	#00FFFF	2319401	1751369	252539	56611	252539	2571940	false
	HGL 22	152696774	152762347	62841132	62928122	+	#00FFFF	65573	86990	56611	88374	56611	122184	false
	HGL 22	152850721	153705627	62983037	63623730	+	#00FFFF	854906	640693	88374	114629	88374	943280	false
	HGL 22	153820256	153957484	63714227	63883483	+	#00FFFF	137228	169256	114629	74958	114629	251857	false
	HGL 22	154032442	159175861	63893788	67144732	+	#00FFFF	5143419	3250944	74958		74958	5218377	false
**HSA 8**	HGL 11	362093	1189282	109262	692096	+	#7F00FF	827189	582834		52348			
	HGL 11	1241630	2244724	712865	1225069	+	#7F00FF	1003094	512204	52348	257215	52348	1055442	false
	HGL 11	2501939	2583288	1330024	1423210	+	#7F00FF	81349	93186	257215	19083	257215	338564	false
	HGL 11	2602371	5502550	1473369	3647755	+	#7F00FF	2900179	2174386	19083	10015	19083	2919262	false
	HGL 11	5512565	5893311	3648036	3954561	-	#7F00FF	380746	306525	10015	232	10015	390761	false
	HGL 11	5893543	7060477	3954561	4759402	+	#7F00FF	1166934	804841	232	247562	232	1167166	false
	HGL 19	7082267	7147439	20758173	20824442	-	#0000FF	65172	66269		17131			
	HGL 19	7164570	7239333	20755433	20824442	+	#0000FF	74763	69009	17131	808883	17131	91894	false
	HGL 11	7308039	7367127	5470799	5567869	-	#7F00FF	59088	97070	247562	13943	247562	306650	false
	HGL 11	7381070	7491065	4933283	5046038	-	#7F00FF	109995	112755	13943	329427	13943	123938	false
	HGL 11	7820492	7930817	4933283	5046038	+	#7F00FF	110325	112755	329427	12412	329427	439752	false
	HGL 11	7943229	8002331	5470799	5567869	+	#7F00FF	59102	97070	12412	241543	12412	71514	false
	HGL 19	8048216	8122911	20755433	20824442	-	#0000FF	74695	69009	808883	1378	808883	883578	false
	HGL 19	8124289	8241422	20846187	20918361	+	#0000FF	117133	72174	1378	4178400	1378	118511	false
	HGL 11	8243874	8743188	49027750	49543471	+	#7F00FF	499314	515721	241543	5482	241543	740857	false
	HGL 11	8748670	9787481	49607539	50604613	+	#7F00FF	1038811	997074	5482	40615	5482	1044293	false
	HGL 11	9828096	11992454	5591840	7237889	-	#7F00FF	2164358	1646049	40615	111542	40615	2204973	false
	HGL 11	12103996	12162860	5470799	5567869	+	#7F00FF	58864	97070	111542	187379	111542	170406	false
	HGL 11	12350239	12405183	5470799	5567869	+	#7F00FF	54944	97070	187379	316355	187379	242323	false
	HGL 19	12419822	12541384	20846187	20916220	-	#0000FF	121562	70033	4178400	81426	4178400	4299962	false
	HGL 19	12622810	12677088	20760848	20824442	+	#0000FF	54278	63594	81426		81426	135704	false

	HGL 11	12721538	16754449	44731700	49018661	-	#7F00FF	4032911	4286961	316355	-19200	316355	4349266	false
	HGL 11	16735249	18103888	43161889	44717191	-	#7F00FF	1368639	1555302	-19200	230766	-19200	1349439	false
	HGL 11	18334654	23046467	12576724	16875710	-	#7F00FF	4711813	4298986	230766	86499	230766	4942579	false
	HGL 11	23132966	27548288	8807631	12599780	-	#7F00FF	4415322	3792149	86499	-21492	86499	4501821	false
	HGL 11	27526796	29276267	7244667	8668242	-	#7F00FF	1749471	1423575	-21492	50178	-21492	1727979	false
	HGL 11	29326445	36173972	50605473	58076233	+	#7F00FF	6847527	7470760	50178	6	50178	6897705	false
	HGL 11	36173978	36273413	58087088	58188359	-	#7F00FF	99435	101271	6	369	6	99441	false
	HGL 11	36273782	37979306	58188359	60035409	+	#7F00FF	1705524	1847050	369	12446	369	1705893	false
	HGL 11	37991752	39163352	60108221	61227319	+	#7F00FF	1171600	1119098	12446	-52686	12446	1184046	false
	HGL 11	39110666	39287648	61635898	61789256	+	#7F00FF	176982	153358	-52686	23649	-52686	124296	false
	HGL 11	39311297	39442620	61481189	61635400	+	#7F00FF	131323	154211	23649	3338	23649	154972	false
	HGL 11	39445958	39523831	61266409	61460754	+	#7F00FF	77873	194345	3338	6851	3338	81211	false
	HGL 11	39530682	39585745	61265234	61406884	-	#7F00FF	55063	141650	6851	36692	6851	61914	false
	HGL 11	39622437	39730224	61848221	61924028	+	#7F00FF	107787	75807	36692	12555	36692	144479	false
	HGL 11	39742779	39838258	8671713	8744032	+	#7F00FF	95479	72319	12555	51361	12555	108034	false
	HGL 11	39889619	41452572	61963493	63782503	+	#7F00FF	1562953	1819010	51361	18060	51361	1614314	false
	HGL 11	41470632	42807225	63867319	65289944	+	#7F00FF	1336593	1422625	18060	26601	18060	1354653	false
	HGL 11	42833826	43030662	65403528	65610706	+	#7F00FF	196836	207178	26601	25579	26601	223437	false
	HGL 11	43056241	43205136	65695286	65899910	+	#7F00FF	148895	204624	25579		25579	174474	false
	HGL 21	46544751	46628088	64749665	64831180	-	#0C8017	83337	81515		68786			
	HGL 21	46696874	46759565	64775495	64856707	+	#0C8017	62691	81212	68786		68786	131477	false
	HGL 12	47257053	48205803	91953133	92905580	+	#DD528A	948750	952447		52005			
	HGL 12	48257808	53128662	92919378	97753444	+	#DD528A	4870854	4834066	52005	57811	52005	4922859	false
	HGL 12	53186473	53436639	97764520	97993413	+	#DD528A	250166	228893	57811	52533	57811	307977	false
	HGL 12	53489172	55551938	98002184	100074019	+	#DD528A	2062766	2071835	52533	65463915	52533	2115299	false
	HGL 17	55837965	57010486	58953586	60190451	-	#8C5319	1172521	1236865		3433			
	HGL 17	57013919	57081916	58869101	58953338	+	#8C5319	67997	84237	3433	0	3433	71430	false
	HGL 17	57081916	57140294	58807088	58866986	-	#8C5319	58378	59898	0	308892	0	58378	false
	HGL 17	57449186	58703914	57369750	58801720	-	#8C5319	1254728	1431970	308892	8301	308892	1563620	false
	HGL 17	58712215	61501115	54394340	57313958	-	#8C5319	2788900	2919618	8301	-47377	8301	2797201	false
	HGL 17	61453738	63112161	52628447	54393956	-	#8C5319	1658423	1765509	-47377	-645059	-47377	1611046	false
	HGL 17	62467102	62538527	53697725	53798968	+	#8C5319	71425	101243	-645059	38403	-645059	-573634	true

	HGL 17	62576930	62678832	53860648	53973741	+	#8C5319	101902	113093	38403	482045	38403	140305	false
	HGL 17	63160877	70760708	44458956	52573302	-	#8C5319	7599831	8114346	482045	40764	482045	8081876	false
	HGL 17	70801472	72199638	42822305	44400828	-	#8C5319	1398166	1578523	40764	14	40764	1438930	false
	HGL 17	72199652	72285508	42917528	43011260	-	#8C5319	85856	93732	14	23756	14	85870	false
	HGL 17	72309264	82635786	32816135	42857335	-	#8C5319	10326522	10041200	23756	639	23756	10350278	false
	HGL 17	82636425	83474199	32113909	32812456	+	#8C5319	837774	698547	639	2081	639	838413	false
	HGL 17	83476280	85055828	30588583	32107706	-	#8C5319	1579548	1519123	2081	49650	2081	1581629	false
	HGL 17	85105478	85577901	30046167	30523500	-	#8C5319	472423	477333	49650	6442	49650	522073	false
	HGL 17	85584343	85636994	29956913	30044401	+	#8C5319	52651	87488	6442	194964	6442	59093	false
	HGL 17	85831958	92011965	23970980	29956420	-	#8C5319	6180007	5985440	194964	471011	194964	6374971	false
	HGL 17	92482976	101318358	15934821	23970567	-	#8C5319	8835382	8035746	471011	203	471011	9306393	false
	HGL 17	101318561	102440209	4430325	5299840	-	#8C5319	1121648	869515	203	88036	203	1121851	false
	HGL 17	102528245	102661410	4358044	4430103	-	#8C5319	133165	72059	88036	82814	88036	221201	false
	HGL 17	102744224	107563690	1104640	4357350	-	#8C5319	4819466	3252710	82814	52393	82814	4902280	false
	HGL 17	107616083	108892608	86508	1104217	-	#8C5319	1276525	1017709	52393	17183	52393	1328918	false
	HGL 17	108909791	113470570	11747483	15927382	-	#8C5319	4560779	4179899	17183	55492	17183	4577962	false
	HGL 17	113526062	113751201	11539291	11733701	-	#8C5319	225139	194410	55492	-42640	55492	280631	false
	HGL 17	113708561	113808195	11500284	11583418	+	#8C5319	99634	83134	-42640	8	-42640	56994	false
	HGL 17	113808203	120051292	6001169	11499549	-	#8C5319	6243089	5498380	8	71016	8	6243097	false
	HGL 17	120122308	121018038	5299975	6003200	-	#8C5319	895730	703225	71016		71016	966746	false
	HGL 12	121015853	133931054	66457344	77961097	+	#DD528A	12915201	11503753	65463915	963	65463915	78379116	false
	HGL 12	133932017	133993313	77965124	78020395	-	#DD528A	61296	55271	963	2663	963	62259	false
	HGL 12	133995976	142918205	78027933	86389658	+	#DD528A	8922229	8361725	2663	-81126	2663	8924892	false
	HGL 12	142837079	144756069	86410774	87998367	+	#DD528A	1918990	1587593	-81126	118713	-81126	1837864	false
	HGL 12	144874782	144952043	88083360	88145568	+	#DD528A	77261	62208	118713		118713	195974	false
**HSA 9**	HGL 14	69631	6070021	16138957	22006840	+	#98C405	6000390	5867883		57433			
	HGL 14	6127454	6667938	22013069	22537541	+	#98C405	540484	524472	57433	73679	57433	597917	false
	HGL 14	6741617	11747676	22633045	27408250	+	#98C405	5006059	4775205	73679	58915	73679	5079738	false
	HGL 14	11806591	14966243	27438649	30531481	+	#98C405	3159652	3092832	58915	108927	58915	3218567	false
	HGL 14	15075170	19133542	30548291	34463718	+	#98C405	4058372	3915427	108927	50545	108927	4167299	false
	HGL 14	19184087	21146281	34487938	36484171	+	#98C405	1962194	1996233	50545	332583	50545	2012739	false
	HGL 14	21478864	23939633	36795971	39292936	+	#98C405	2460769	2496965	332583	69526	332583	2793352	false

	HGL 14	24009159	29858746	39345380	45502561	+	#98C405	5849587	6157181	69526	94	69526	5919113	false
	HGL 14	29858840	29958038	45556886	45714788	+	#98C405	99198	157902	94	36682	94	99292	false
	HGL 14	29994720	30538920	45772302	46308503	+	#98C405	544200	536201	36682	65495	36682	580882	false
	HGL 14	30604415	33474445	46384520	49566797	+	#98C405	2870030	3182277	65495	338884	65495	2935525	false
	HGL 14	33813329	36173464	49568859	51968609	+	#98C405	2360135	2399750	338884	-1913	338884	2699019	false
	HGL 14	36171551	36334261	52032833	52211056	+	#98C405	162710	178223	-1913	486	-1913	160797	false
	HGL 14	36334747	38472134	52498773	54522307	+	#98C405	2137387	2023534	486	2543202	486	2137873	false
	HGL 1	39879026	40100914	105077425	105298202	+	#13C840	221888	220777		1263683			
	HGL 2	40364771	40478609	117395491	117519291	-	#C008AD	113838	123800		2512539			
	HGL 14	41015336	41196458	16081913	16284605	+	#98C405	181122	202692	2543202	22877101	2543202	2724324	false
	HGL 1	41364597	41643581	105046776	105298202	-	#13C840	278984	251426	1263683	20620351	1263683	1542667	false
	HGL 2	42991148	43106942	117395491	117519291	-	#C008AD	115794	123800	2512539	20039410	2512539	2628333	false
	HGL 1	62263932	62371698	105077425	105170453	-	#13C840	107766	93028	20620351	1187281	20620351	20728117	false
	HGL 2	63146352	63202891	117395491	117460060	+	#C008AD	56539	64569	20039410	49931	20039410	20095949	false
	HGL 2	63252822	63314145	117395491	117464558	-	#C008AD	61323	69067	49931	1190700	49931	111254	false
	HGL 1	63558979	63698186	105145273	105298202	+	#13C840	139207	152929	1187281	2577550	1187281	1326488	false
	HGL 14	64073559	64127045	58790	158665	+	#98C405	53486	99875	22877101	1541734	22877101	22930587	false
	HGL 2	64504845	64618625	117395491	117519291	-	#C008AD	113780	123800	1190700	1279085	1190700	1304480	false
	HGL 14	65668779	65738803	16211271	16284605	-	#98C405	70024	73334	1541734	2496033	1541734	1611758	false
	HGL 2	65897710	66011550	117395491	117519291	+	#C008AD	113840	123800	1279085		1279085	1392925	false
	HGL 1	66275736	66391475	105165366	105298202	-	#13C840	115739	132836	2577550	1335637	2577550	2693289	false
	HGL 1	67727112	67831306	105046776	105135539	+	#13C840	104194	88763	1335637		1335637	1439831	false
	HGL 14	68234836	72257808	12164772	16284605	-	#98C405	4022972	4119833	2496033	-36392	2496033	6519005	false
	HGL 14	72221416	72317356	12076202	12131781	-	#98C405	95940	55579	-36392	3261	-36392	59548	false
	HGL 14	72320617	72903763	11381453	11989396	-	#98C405	583146	607943	3261	-32984	3261	586407	false
	HGL 14	72870779	73014362	11247349	11340090	+	#98C405	143583	92741	-32984	-143583	-32984	110599	false
	HGL 14	72870779	83146021	320393	10864113	-	#98C405	10275242	10543720	-143583	9062469	-143583	10131659	false
	HGL 21	83242058	83822443	75390120	76068121	+	#0C8017	580385	678001		10360			
	HGL 21	83832803	85748005	76760503	78774433	+	#0C8017	1915202	2013930	10360	127259	10360	1925562	false
	HGL 21	85875264	86100765	78778861	78976561	+	#0C8017	225501	197700	127259	124492	127259	352760	false
	HGL 21	86225257	87409495	79288268	80564350	-	#0C8017	1184238	1276082	124492	6688	124492	1308730	false
	HGL 21	87416183	87488185	79226285	79288268	+	#0C8017	72002	61983	6688	21	6688	78690	false

	HGL 21	87488206	87758330	78993133	79223136	-	#0C8017	270124	230003	21		21	270145	false
	HGL 24	88085921	90172851	43585302	45786812	-	#F50241	2086930	2201510	49359013	74281	49359013	51445943	false
	HGL 24	90247132	91464196	47653618	48896862	+	#F50241	1217064	1243244	74281	10322	74281	1291345	false
	HGL 24	91474518	91547027	48912782	49019942	-	#F50241	72509	107160	10322	7383	10322	82831	false
	HGL 24	91554410	92122135	49020230	49654100	+	#F50241	567725	633870	7383	115231	7383	575108	false
	HGL 24	92237366	92775086	45807554	46366277	+	#F50241	537720	558723	115231	169504	115231	652951	false
	HGL 24	92944590	93321320	46396054	46697088	+	#F50241	376730	301034	169504	101902	169504	546234	false
	HGL 24	93423222	93985444	46778154	47332644	+	#F50241	562222	554490	101902	25596	101902	664124	false
	HGL 24	94011040	94214148	47377920	47552132	-	#F50241	203108	174212	25596		25596	228704	false
	HGL 22	94342341	94461857	54448297	54580791	+	#00FFFF	119516	132494	60643841	95567	60643841	60763357	false
	HGL 22	94557424	96319054	33329101	35216687	+	#00FFFF	1761630	1887586	95567	-2323	95567	1857197	false
	HGL 22	96316731	96670546	35235670	35599562	-	#00FFFF	353815	363892	-2323	278779	-2323	351492	false
	HGL 22	96949325	97026015	54475685	54559169	-	#00FFFF	76690	83484	278779	2586	278779	355469	false
	HGL 22	97028601	97088795	54282239	54347329	-	#00FFFF	60194	65090	2586		2586	62780	false
	HGL 14	97275303	101388124	54522307	58501252	+	#98C405	4112821	3978945	4926125	-52233	4926125	9038946	false
	HGL 14	101335891	104630107	58549917	61741574	+	#98C405	3294216	3191657	-52233	-40558	-52233	3241983	false
	HGL 14	104589549	104812806	61740747	61899660	+	#98C405	223257	158913	-40558	-69412	-40558	182699	false
	HGL 14	104743394	115976644	61904053	72699954	+	#98C405	11233250	10795901	-69412	45	-69412	11163838	false
	HGL 14	115976689	116037751	72699954	72766682	-	#98C405	61062	66728	45	59	45	61107	false
	HGL 14	116037810	121097938	72766683	77718624	+	#98C405	5060128	4951941	59	-84598	59	5060187	false
	HGL 14	121013340	121421489	77718670	78088869	+	#98C405	408149	370199	-84598	17556	-84598	323551	false
	HGL 14	121439045	122454578	78145872	79067914	+	#98C405	1015533	922042	17556	18347	17556	1033089	false
	HGL 14	122472925	128154332	79457245	84471448	+	#98C405	5681407	5014203	18347	1925	18347	5699754	false
	HGL 14	128156257	133173334	85279784	88637611	+	#98C405	5017077	3357827	1925	157971	1925	5019002	false
	HGL 14	133331305	135530996	88809472	90156911	-	#98C405	2199691	1347439	157971	8553	157971	2357662	false
	HGL 14	135539549	136724262	90187988	90875328	-	#98C405	1184713	687340	8553	14515	8553	1193266	false
	HGL 14	136738777	138124581	90881933	91878552	+	#98C405	1385804	996619	14515		14515	1400319	false
**HSA X**	HGL X	283173	463087	265040	324585	+	#00FF00	179914	59545		154031			
	HGL X	617118	686760	324588	420432	+	#00FF00	69642	95844	154031	68736	154031	223673	false
	HGL X	755496	831507	420432	496368	+	#00FF00	76011	75936	68736	739276	68736	144747	false
	HGL X	1570783	1643140	663902	757185	+	#00FF00	72357	93283	739276	658794	739276	811633	false
	HGL X	2301934	2427281	972014	1035591	+	#00FF00	125347	63577	658794	59247	658794	784141	false

	HGL X	2486528	2624990	1040785	1182044	+	#00FF00	138462	141259	59247	53743	59247	197709	false
	HGL X	2678733	2740773	1156123	1217945	+	#00FF00	62040	61822	53743	92394	53743	115783	false
	HGL X	2833167	3033931	1233552	1393713	+	#00FF00	200764	160161	92394	156009	92394	293158	false
	HGL X	3189940	3758449	1410892	1750252	+	#00FF00	568509	339360	156009	245644	156009	724518	false
	HGL X	4004093	4499820	1951330	2261306	+	#00FF00	495727	309976	245644	64715	245644	741371	false
	HGL X	4564535	5143800	2272551	2705598	+	#00FF00	579265	433047	64715	40038	64715	643980	false
	HGL X	5183838	8901937	3112310	6456293	+	#00FF00	3718099	3343983	40038	-1323	40038	3758137	false
	HGL X	8900614	8960151	6636213	6700401	+	#00FF00	59537	64188	-1323	74709	-1323	58214	false
	HGL X	9034860	9949472	6732392	7504989	+	#00FF00	914612	772597	74709	15013	74709	989321	false
	HGL X	9964485	12904548	7660210	10652475	+	#00FF00	2940063	2992265	15013	33	15013	2955076	false
	HGL X	12904581	15932396	10714368	13817591	+	#00FF00	3027815	3103223	33	22	33	3027848	false
	HGL X	15932418	16006522	13817591	13895905	-	#00FF00	74104	78314	22	225	22	74126	false
	HGL X	16006747	16217088	13895954	14132002	+	#00FF00	210341	236048	225	19770	225	210566	false
	HGL X	16236858	16321408	14137224	14230268	-	#00FF00	84550	93044	19770	6419	19770	104320	false
	HGL X	16327827	18374506	14230268	16306809	+	#00FF00	2046679	2076541	6419	-2967	6419	2053098	false
	HGL X	18371539	23415180	16326728	21643626	+	#00FF00	5043641	5316898	-2967	0	-2967	5040674	false
	HGL X	23415180	23504569	21652831	21744484	-	#00FF00	89389	91653	0	184	0	89389	false
	HGL X	23504753	23560084	21792567	21873501	-	#00FF00	55331	80934	184	43688	184	55515	false
	HGL X	23603772	30266263	21911378	28465906	+	#00FF00	6662491	6554528	43688	-45376	43688	6706179	false
	HGL X	30220887	34116023	28599133	32616398	+	#00FF00	3895136	4017265	-45376	55767	-45376	3849760	false
	HGL X	34171790	35790564	32632541	34621407	+	#00FF00	1618774	1988866	55767	-899084	55767	1674541	false
	HGL X	34891480	35130897	35582782	35762485	-	#00FF00	239417	179703	-899084	109932	-899084	-659667	true
	HGL X	35240829	35347563	35457287	35564814	-	#00FF00	106734	107527	109932	38853	109932	216666	false
	HGL X	35386416	35579774	35131680	35395773	-	#00FF00	193358	264093	38853	58212	38853	232211	false
	HGL X	35637986	35714798	35011318	35114652	-	#00FF00	76812	103334	58212	83857	58212	135024	false
	HGL X	35798655	35860700	34609823	34720674	-	#00FF00	62045	110851	83857	-32573	83857	145902	false
	HGL X	35828127	35898440	34918946	34999863	-	#00FF00	70313	80917	-32573	-41548	-32573	37740	false
	HGL X	35856892	36582461	34778194	35580491	+	#00FF00	725569	802297	-41548	-104271	-41548	684021	false
	HGL X	36478190	36678475	33808688	34067010	-	#00FF00	200285	258322	-104271	-35218	-104271	96014	false
	HGL X	36643257	36722598	35580670	35634034	+	#00FF00	79341	53364	-35218	60671	-35218	44123	false
	HGL X	36783269	36913294	35638860	35819817	+	#00FF00	130025	180957	60671	-35491	60671	190696	false
	HGL X	36877803	36945193	33444749	33536606	-	#00FF00	67390	91857	-35491	-65803	-35491	31899	false

	HGL X	36879390	36966884	35861897	36008602	+	#00FF00	87494	146705	-65803	1446	-65803	21691	false
	HGL X	36968330	37076881	36274652	36408741	-	#00FF00	108551	134089	1446	214312	1446	109997	false
	HGL X	37291193	37507034	36008785	36211879	-	#00FF00	215841	203094	214312	-63958	214312	430153	false
	HGL X	37443076	45328349	36274652	44599685	+	#00FF00	7885273	8325033	-63958	96809	-63958	7821315	false
	HGL X	45425158	45475739	44746555	44812077	+	#00FF00	50581	65522	96809	23323	96809	147390	false
	HGL X	45499062	47301499	44862353	47075644	+	#00FF00	1802437	2213291	23323	191436	23323	1825760	false
	HGL X	47492935	47663945	47424844	47635588	+	#00FF00	171010	210744	191436	114449	191436	362446	false
	HGL X	47778394	48033258	47666813	47928174	+	#00FF00	254864	261361	114449	10146	114449	369313	false
	HGL X	48043404	48166579	48024123	48097780	+	#00FF00	123175	73657	10146	225179	10146	133321	false
	HGL X	48391758	49346447	48096481	48922319	+	#00FF00	954689	825838	225179	324412	225179	1179868	false
	HGL X	49670859	50145367	48960285	49512986	+	#00FF00	474508	552701	324412	134922	324412	798920	false
	HGL X	50280289	50527724	49638177	50038839	+	#00FF00	247435	400662	134922	10979	134922	382357	false
	HGL X	50538703	50936219	50130082	50676485	+	#00FF00	397516	546403	10979	-64661	10979	408495	false
	HGL X	50871558	51056431	50805304	51029665	+	#00FF00	184873	224361	-64661	153240	-64661	120212	false
	HGL X	51209671	51279081	51037243	51110018	+	#00FF00	69410	72775	153240	8954	153240	222650	false
	HGL X	51288035	51378436	51403267	51460058	-	#00FF00	90401	56791	8954	89156	8954	99355	false
	HGL X	51467592	51526322	51380381	51460088	+	#00FF00	58730	79707	89156	154824	89156	147886	false
	HGL X	51681146	51957649	51525086	51908218	+	#00FF00	276503	383132	154824	116435	154824	431327	false
	HGL X	52074084	52244882	51951442	52065598	-	#00FF00	170798	114156	116435	45966	116435	287233	false
	HGL X	52290848	52340868	55394314	55455509	-	#00FF00	50020	61195	45966	546702	45966	95986	false
	HGL X	52887570	52944333	52280139	52357263	-	#00FF00	56763	77124	546702	-32853	546702	603465	false
	HGL X	52911480	53716484	52280139	53048522	+	#00FF00	805004	768383	-32853	212733	-32853	772151	false
	HGL X	53929217	54420890	53490856	54322716	+	#00FF00	491673	831860	212733	18979	212733	704406	false
	HGL X	54439869	54563139	54400618	54550329	+	#00FF00	123270	149711	18979	160331	18979	142249	false
	HGL X	54723470	54842724	54782122	54870211	-	#00FF00	119254	88089	160331	-69226	160331	279585	false
	HGL X	54773498	54844186	54782122	54842524	+	#00FF00	70688	60402	-69226	54099	-69226	1462	false
	HGL X	54898285	55035798	55037425	55236038	+	#00FF00	137513	198613	54099	63865	54099	191612	false
	HGL X	55099663	55159962	55281019	55343206	-	#00FF00	60299	62187	63865	556767	63865	124164	false
	HGL X	55716729	55804750	55506769	55606366	+	#00FF00	88021	99597	556767	419317	556767	644788	false
	HGL X	56224067	56302272	55890611	56019014	+	#00FF00	78205	128403	419317	227112	419317	497522	false
	HGL X	56529384	56592049	56301459	56358281	+	#00FF00	62665	56822	227112	135755	227112	289777	false
	HGL X	56727804	56820474	56692666	56757777	+	#00FF00	92670	65111	135755	488433	135755	228425	false

	HGL X	57308907	57434098	57014301	57102559	-	#00FF00	125191	88258	488433	6198063	488433	613624	false
	HGL X	63632161	63818699	57955434	58276237	+	#00FF00	186538	320803	6198063	359889	6198063	6384601	false
	HGL X	64178588	64274194	58494443	58700651	+	#00FF00	95606	206208	359889	50880	359889	455495	false
	HGL X	64325074	64447061	58700651	58760208	+	#00FF00	121987	59557	50880	434589	50880	172867	false
	HGL X	64881650	64993425	58777305	58913013	+	#00FF00	111775	135708	434589	20417	434589	546364	false
	HGL X	65013842	65114429	58976893	59102166	+	#00FF00	100587	125273	20417	361747	20417	121004	false
	HGL X	65476176	65747482	59436261	59749337	+	#00FF00	271306	313076	361747	82868	361747	633053	false
	HGL X	65830350	65896232	59750631	59825596	+	#00FF00	65882	74965	82868	76104	82868	148750	false
	HGL X	65972336	66116959	59875513	60065815	+	#00FF00	144623	190302	76104	30182	76104	220727	false
	HGL X	66147141	66302407	60117917	60282684	+	#00FF00	155266	164767	30182	77071	30182	185448	false
	HGL X	66379478	66478223	60334344	60445469	+	#00FF00	98745	111125	77071	111675	77071	175816	false
	HGL X	66589898	66816140	60568522	60757490	+	#00FF00	226242	188968	111675	52777	111675	337917	false
	HGL X	66868917	66921495	60819460	60936926	-	#00FF00	52578	117466	52777	95915	52777	105355	false
	HGL X	67017410	67081329	60992341	61071031	+	#00FF00	63919	78690	95915	176368	95915	159834	false
	HGL X	67257697	67351785	61371023	61466880	+	#00FF00	94088	95857	176368	168347	176368	270456	false
	HGL X	67520132	67814121	61662899	62137385	+	#00FF00	293989	474486	168347	119385	168347	462336	false
	HGL X	67933506	68552990	62469943	63294851	+	#00FF00	619484	824908	119385	30076	119385	738869	false
	HGL X	68583066	70057280	63427880	65017299	+	#00FF00	1474214	1589419	30076	25353	30076	1504290	false
	HGL X	70082633	70563603	65101118	65598757	+	#00FF00	480970	497639	25353	53673	25353	506323	false
	HGL X	70617276	70745468	65614834	65899113	+	#00FF00	128192	284279	53673	59148	53673	181865	false
	HGL X	70804616	71024142	65960104	66318733	+	#00FF00	219526	358629	59148	5818	59148	278674	false
	HGL X	71029960	71661441	66369429	67090612	+	#00FF00	631481	721183	5818	196216	5818	637299	false
	HGL X	71857657	72731426	67071319	68132284	+	#00FF00	873769	1060965	196216	958267	196216	1069985	false
	HGL X	73689693	73792534	69048778	69110375	+	#00FF00	102841	61597	958267	145592	958267	1061108	false
	HGL X	73938126	73997827	69287615	69354743	+	#00FF00	59701	67128	145592	215136	145592	205293	false
	HGL X	74212963	74325534	69581466	69790663	+	#00FF00	112571	209197	215136	85057	215136	327707	false
	HGL X	74410591	74538124	70010938	70237699	-	#00FF00	127533	226761	85057	-2473	85057	212590	false
	HGL X	74535651	74677390	70360165	70510322	+	#00FF00	141739	150157	-2473	51205	-2473	139266	false
	HGL X	74728595	75340844	70593915	71221280	+	#00FF00	612249	627365	51205	20039	51205	663454	false
	HGL X	75360883	75517138	71279646	71420535	+	#00FF00	156255	140889	20039	86104	20039	176294	false
	HGL X	75603242	75669739	71520571	71577717	+	#00FF00	66497	57146	86104	1020042	86104	152601	false
	HGL X	76689781	76804074	72405340	72462123	+	#00FF00	114293	56783	1020042	50205	1020042	1134335	false

	HGL X	76854279	77152096	72522400	72692414	+	#00FF00	297817	170014	50205	263588	50205	348022	false
	HGL X	77415684	77723998	73055022	73468370	+	#00FF00	308314	413348	263588	50228	263588	571902	false
	HGL X	77774226	78051028	73493465	73885763	+	#00FF00	276802	392298	50228	49090	50228	327030	false
	HGL X	78100118	78150623	74001553	74064446	+	#00FF00	50505	62893	49090	116330	49090	99595	false
	HGL X	78266953	78365615	74077819	74174269	+	#00FF00	98662	96450	116330	574855	116330	214992	false
	HGL X	78940470	79286856	74557548	74981531	+	#00FF00	346386	423983	574855	70287	574855	921241	false
	HGL X	79357143	79441511	75003120	75113741	+	#00FF00	84368	110621	70287	561444	70287	154655	false
	HGL X	80002955	80141872	75577640	75723854	+	#00FF00	138917	146214	561444	46022	561444	700361	false
	HGL X	80187894	80346135	75852878	75952104	+	#00FF00	158241	99226	46022	304133	46022	204263	false
	HGL X	80650268	80749365	76141334	76319920	+	#00FF00	99097	178586	304133	5057	304133	403230	false
	HGL X	80754422	80814497	76374099	76442088	+	#00FF00	60075	67989	5057	296617	5057	65132	false
	HGL X	81111114	81298283	76645799	76837150	+	#00FF00	187169	191351	296617	1397711	296617	483786	false
	HGL X	82695994	82831658	77984595	78193480	+	#00FF00	135664	208885	1397711	583910	1397711	1533375	false
	HGL X	83415568	83576920	78691790	78962890	+	#00FF00	161352	271100	583910	458116	583910	745262	false
	HGL X	84035036	84564870	79370211	80125070	+	#00FF00	529834	754859	458116	434935	458116	987950	false
	HGL X	84999805	85416228	80289905	80901241	+	#00FF00	416423	611336	434935	16127	434935	851358	false
	HGL X	85432355	85549412	80968635	81154806	+	#00FF00	117057	186171	16127	140682	16127	133184	false
	HGL X	85690094	85798181	81187284	81318384	+	#00FF00	108087	131100	140682	56778	140682	248769	false
	HGL X	85854959	86888340	81336729	82670191	+	#00FF00	1033381	1333462	56778	413150	56778	1090159	false
	HGL X	87301490	87435368	83015647	83167821	+	#00FF00	133878	152174	413150	50764	413150	547028	false
	HGL X	87486132	87670280	83195621	83400089	+	#00FF00	184148	204468	50764	2461276	50764	234912	false
	HGL X	90131556	90189477	83563426	83663768	-	#00FF00	57921	100342	2461276	1236439	2461276	2519197	false
	HGL X	91425916	91496060	84158238	84261696	+	#00FF00	70144	103458	1236439	52800	1236439	1306583	false
	HGL X	91548860	91617217	84284109	84363480	+	#00FF00	68357	79371	52800	91514	52800	121157	false
	HGL X	91708731	91844937	84376864	84603716	+	#00FF00	136206	226852	91514	152404	91514	227720	false
	HGL X	91997341	92422190	84785957	85427922	+	#00FF00	424849	641965	152404	114158	152404	577253	false
	HGL X	92536348	92630735	85508500	85642054	+	#00FF00	94387	133554	114158	779678	114158	208545	false
	HGL X	93410413	93599388	86116065	86410755	+	#00FF00	188975	294690	779678	14352	779678	968653	false
	HGL X	93613740	93927054	86491169	86854726	+	#00FF00	313314	363557	14352	123854	14352	327666	false
	HGL X	94050908	94377385	86920030	87395641	+	#00FF00	326477	475611	123854	52258	123854	450331	false
	HGL X	94429643	94589688	87400188	87540211	+	#00FF00	160045	140023	52258	178732	52258	212303	false
	HGL X	94768420	94857446	87761137	87883949	+	#00FF00	89026	122812	178732	97831	178732	267758	false

	HGL X	94955277	95026072	87883952	87983199	+	#00FF00	70795	99247	97831	94557	97831	168626	false
	HGL X	95120629	95182062	88221921	88274514	+	#00FF00	61433	52593	94557	33167	94557	155990	false
	HGL X	95215229	95443465	88360428	88637501	+	#00FF00	228236	277073	33167	8496	33167	261403	false
	HGL X	95451961	95520288	88697845	88837392	+	#00FF00	68327	139547	8496	61234	8496	76823	false
	HGL X	95581522	96571553	88855421	90418177	+	#00FF00	990031	1562756	61234	-24154	61234	1051265	false
	HGL X	96547399	97780359	90432683	91956904	+	#00FF00	1232960	1524221	-24154	45670	-24154	1208806	false
	HGL X	97826029	98008756	92046452	92326001	+	#00FF00	182727	279549	45670	23440	45670	228397	false
	HGL X	98032196	98506817	92387907	93033415	+	#00FF00	474621	645508	23440	1870	23440	498061	false
	HGL X	98508687	98906322	93116924	93738360	+	#00FF00	397635	621436	1870	62296	1870	399505	false
	HGL X	98968618	99196196	93815652	94236672	+	#00FF00	227578	421020	62296	52769	62296	289874	false
	HGL X	99248965	99455441	94237957	94492057	+	#00FF00	206476	254100	52769	40231	52769	259245	false
	HGL X	99495672	99632332	94563431	94767566	+	#00FF00	136660	204135	40231	30918	40231	176891	false
	HGL X	99663250	100127257	94849063	95544853	+	#00FF00	464007	695790	30918	0	30918	494925	false
	HGL X	100127257	100219361	95556234	95663991	-	#00FF00	92104	107757	0	22553	0	92104	false
	HGL X	100241914	100516310	95671049	96035366	+	#00FF00	274396	364317	22553	5079	22553	296949	false
	HGL X	100521389	101032959	96100136	96604454	+	#00FF00	511570	504318	5079	2222	5079	516649	false
	HGL X	101035181	101293459	96656513	96968716	+	#00FF00	258278	312203	2222	50072	2222	260500	false
	HGL X	101343531	101497721	96986813	97160593	+	#00FF00	154190	173780	50072	50519	50072	204262	false
	HGL X	101548240	101708180	97152774	97363177	+	#00FF00	159940	210403	50519	-27702	50519	210459	false
	HGL X	101680478	102024505	97491456	97947326	+	#00FF00	344027	455870	-27702	61234	-27702	316325	false
	HGL X	102085739	102157653	98054120	98115725	+	#00FF00	71914	61605	61234	439114	61234	133148	false
	HGL X	102596767	102682056	98320261	98384261	-	#00FF00	85289	64000	439114	467609	439114	524403	false
	HGL X	103149665	103446518	98860089	99055751	+	#00FF00	296853	195662	467609	74246	467609	764462	false
	HGL X	103520764	103854586	99098059	99494538	+	#00FF00	333822	396479	74246	-327	74246	408068	false
	HGL X	103854259	103906120	99560353	99611800	+	#00FF00	51861	51447	-327	11852	-327	51534	false
	HGL X	103917972	103989383	99584971	99655090	-	#00FF00	71411	70119	11852	-12089	11852	83263	false
	HGL X	103977294	106044838	99587493	102070630	+	#00FF00	2067544	2483137	-12089	57762	-12089	2055455	false
	HGL X	106102600	106302674	102066159	102284843	+	#00FF00	200074	218684	57762	-58241	57762	257836	false
	HGL X	106244433	106350308	102177688	102286371	-	#00FF00	105875	108683	-58241	36364	-58241	47634	false
	HGL X	106386672	106459097	150964396	151024245	-	#00FF00	72425	59849	36364	17503	36364	108789	false
	HGL X	106476600	106538455	102380853	102458632	+	#00FF00	61855	77779	17503	66531	17503	79358	false
	HGL X	106604986	107019072	102487197	102952876	+	#00FF00	414086	465679	66531	42678	66531	480617	false

	HGL X	107061750	108486775	103109003	104832954	+	#00FF00	1425025	1723951	42678	3	42678	1467703	false
	HGL X	108486778	108751471	104883462	105224501	+	#00FF00	264693	341039	3	88912	3	264696	false
	HGL X	108840383	108894686	105344736	105457978	+	#00FF00	54303	113242	88912	54024	88912	143215	false
	HGL X	108948710	109053317	105501943	105711884	+	#00FF00	104607	209941	54024	168047	54024	158631	false
	HGL X	109221364	109308771	105819606	105900451	+	#00FF00	87407	80845	168047	5055	168047	255454	false
	HGL X	109313826	110494205	105959926	107293084	+	#00FF00	1180379	1333158	5055	57626	5055	1185434	false
	HGL X	110551831	110639637	107418773	107506410	-	#00FF00	87806	87637	57626	6536	57626	145432	false
	HGL X	110646173	114279885	107506410	112451854	+	#00FF00	3633712	4945444	6536	52659	6536	3640248	false
	HGL X	114332544	114914612	112457699	113035130	+	#00FF00	582068	577431	52659	36224	52659	634727	false
	HGL X	114950836	115727199	113135829	114060474	+	#00FF00	776363	924645	36224	214191	36224	812587	false
	HGL X	115941390	116015605	114107552	114252898	+	#00FF00	74215	145346	214191	130953	214191	288406	false
	HGL X	116146558	116387180	114396842	114669231	+	#00FF00	240622	272389	130953	13875	130953	371575	false
	HGL X	116401055	116660216	114756953	115008755	+	#00FF00	259161	251802	13875	69393	13875	273036	false
	HGL X	116729609	116811795	115013440	115091116	+	#00FF00	82186	77676	69393	13402	69393	151579	false
	HGL X	116825197	119950104	115165834	118625426	+	#00FF00	3124907	3459592	13402	249126	13402	3138309	false
	HGL X	120199230	120879056	118662325	119553774	+	#00FF00	679826	891449	249126	64207	249126	928952	false
	HGL X	120943263	124125746	119551853	123047260	+	#00FF00	3182483	3495407	64207	71943	64207	3246690	false
	HGL X	124197689	126292465	123055596	125230191	+	#00FF00	2094776	2174595	71943	7255	71943	2166719	false
	HGL X	126299720	127334737	125281138	126523046	+	#00FF00	1035017	1241908	7255	58984	7255	1042272	false
	HGL X	127393721	127471782	126552696	126668234	-	#00FF00	78061	115538	58984	92243	58984	137045	false
	HGL X	127564025	127624963	126762546	126856593	-	#00FF00	60938	94047	92243	4469	92243	153181	false
	HGL X	127629432	128041679	126857595	127334229	+	#00FF00	412247	476634	4469	207635	4469	416716	false
	HGL X	128249314	131677133	127507440	131475973	+	#00FF00	3427819	3968533	207635	90049	207635	3635454	false
	HGL X	131767182	135003063	131657156	134980447	+	#00FF00	3235881	3323291	90049	3733	90049	3325930	false
	HGL X	135006796	135093986	135154425	135294165	+	#00FF00	87190	139740	3733	36417	3733	90923	false
	HGL X	135130403	135216343	135518779	135571552	-	#00FF00	85940	52773	36417	-60143	36417	122357	false
	HGL X	135156200	135295100	135518779	135595997	+	#00FF00	138900	77218	-60143	26524	-60143	78757	false
	HGL X	135321624	135445088	135346944	135448016	-	#00FF00	123464	101072	26524	-25478	26524	149988	false
	HGL X	135419610	135615638	135631450	135806753	+	#00FF00	196028	175303	-25478	-25575	-25478	170550	false
	HGL X	135590063	135687485	135798506	135865625	-	#00FF00	97422	67119	-25575	-5713	-25575	71847	false
	HGL X	135681772	135874990	135801222	135870195	+	#00FF00	193218	68973	-5713	76026	-5713	187505	false
	HGL X	135951016	138212298	135878492	138560594	+	#00FF00	2261282	2682102	76026	32991	76026	2337308	false

	HGL X	138245289	140879318	138616044	141285862	+	#00FF00	2634029	2669818	32991	8464	32991	2667020	false
	HGL X	140887782	140995024	141339781	141436962	+	#00FF00	107242	97181	8464	40380	8464	115706	false
	HGL X	141035404	141472695	141437488	141788818	-	#00FF00	437291	351330	40380	1291	40380	477671	false
	HGL X	141473986	141860993	141703341	142030727	+	#00FF00	387007	327386	1291	-3178	1291	388298	false
	HGL X	141857815	142148667	142031999	142236544	-	#00FF00	290852	204545	-3178	-4527	-3178	287674	false
	HGL X	142144140	142697994	142194138	142709155	+	#00FF00	553854	515017	-4527	4882	-4527	549327	false
	HGL X	142702876	142787261	142709426	142778416	-	#00FF00	84385	68990	4882	7890	4882	89267	false
	HGL X	142795151	144423729	142777257	144283634	+	#00FF00	1628578	1506377	7890	59219	7890	1636468	false
	HGL X	144482948	144861727	144285141	144605444	+	#00FF00	378779	320303	59219	333	59219	437998	false
	HGL X	144862060	145002404	144606131	144719022	-	#00FF00	140344	112891	333	0	333	140677	false
	HGL X	145002404	145241220	144722679	144986898	+	#00FF00	238816	264219	0	32993	0	238816	false
	HGL X	145274213	146764131	145094354	146635250	+	#00FF00	1489918	1540896	32993	2068	32993	1522911	false
	HGL X	146766199	146931830	146635250	146818458	-	#00FF00	165631	183208	2068	17019	2068	167699	false
	HGL X	146948849	147152234	146821159	147071805	+	#00FF00	203385	250646	17019	137498	17019	220404	false
	HGL X	147289732	149531957	147114354	149183498	+	#00FF00	2242225	2069144	137498	1969658	137498	2379723	false
	HGL X	151501615	152108586	149965716	150504965	+	#00FF00	606971	539249	1969658	36772	1969658	2576629	false
	HGL X	152145358	152519244	150562849	150862808	+	#00FF00	373886	299959	36772	0	36772	410658	false
	HGL X	152519244	152692107	150916919	151090263	+	#00FF00	172863	173344	0	115762	0	172863	false
	HGL X	152807869	152998302	151133347	151258976	+	#00FF00	190433	125629	115762	281729	115762	306195	false
	HGL X	153280031	154313258	151265537	152040221	+	#00FF00	1033227	774684	281729	26	281729	1314956	false
	HGL X	154313284	154384525	152068546	152124598	-	#00FF00	71241	56052	26	13385	26	71267	false
	HGL X	154397910	154553252	152124850	152271034	+	#00FF00	155342	146184	13385	121252	13385	168727	false
	HGL X	154674504	155134843	152297014	152730653	+	#00FF00	460339	433639	121252	78527	121252	581591	false
	HGL X	155213370	155336480	152732841	152845185	+	#00FF00	123110	112344	78527	126894	78527	201637	false
	HGL X	155463374	155944777	152875485	153264935	+	#00FF00	481403	389450	126894		126894	608297	false

Supplementary Table S3 (part 2). Syntenic blocks with a minimum cut-off length of 50 kilobases in the reference human genome.

scaffold	query	start	end	query_start	query_end	strand	color	target_len	query_len	dist_upstream	dist_downstream	dist_end_start	dist_end_end	embedded
HSA 1	CPOR 25	903444	1683185	51733408	52276457	-	#D284F9	779741	543049		16503			
	CPOR 25	1699688	2647825	51172383	51774622	-	#D284F9	948137	602239	16503	132726	16503	964640	false
	CPOR 25	2780551	8881496	47129509	51172161	-	#D284F9	6100945	4042652	132726	60513	132726	6233671	false
	CPOR 25	8942009	12632099	44647163	47121552	-	#D284F9	3690090	2474389	60513	26516	60513	3750603	false

	CPOR 25	12658615	12762286	43562195	43681448	-	#D284F9	103671	119253	26516	696196	26516	130187	false
	CPOR 25	13458482	16466235	40326397	42646505	-	#D284F9	3007753	2320108	696196	445716	696196	3703949	false
	CPOR 25	16911951	20009083	37959409	40325752	-	#D284F9	3097132	2366343	445716	46348	445716	3542848	false
	CPOR 25	20055431	21387522	36330148	37449445	-	#D284F9	1332091	1119297	46348	99076	46348	1378439	false
	CPOR 25	21486598	25265653	33357451	36328782	-	#D284F9	3779055	2971331	99076	66930	99076	3878131	false
	CPOR 25	25332583	35165572	25019563	33358487	-	#D284F9	9832989	8338924	66930	-5522906	66930	9899919	false
	CPOR 25	29642666	29960252	29049927	29404795	+	#D284F9	317586	354868	-5522906	-288083	-5522906	-5205320	true
	CPOR 25	29672169	29745014	29435416	29512965	-	#D284F9	72845	77549	-288083	45915	-288083	-215238	true
	CPOR 25	29790929	30050350	28957064	29168862	+	#D284F9	259421	211798	45915	-79676	45915	305336	false
	CPOR 25	29970674	30048348	29878380	29962824	-	#D284F9	77674	84444	-79676	40784	-79676	-2002	true
	CPOR 25	30089132	30190141	29486410	29542503	+	#D284F9	101009	56093	40784	9366	40784	141793	false
	CPOR 25	30199507	30452033	29764424	29973712	+	#D284F9	252526	209288	9366	13062	9366	261892	false
	CPOR 25	30465095	30695474	29219898	29434767	-	#D284F9	230379	214869	13062	-218268	13062	243441	false
	CPOR 25	30477206	30708342	29680732	29850073	+	#D284F9	231136	169341	-218268	4461438	-218268	12868	false
	CPOR 25	35169780	37771319	22705599	24945683	-	#D284F9	2601539	2240084	4461438	11614	4461438	7062977	false
	CPOR 25	37782933	40518181	20294586	22619262	-	#D284F9	2735248	2324676	11614	-783507	11614	2746862	false
	CPOR 25	39734674	39797934	21122800	21211685	+	#D284F9	63260	88885	-783507	733386	-783507	-720247	true
	CPOR 25	40531320	42192710	18710296	20225209	-	#D284F9	1661390	1514913	733386	102	733386	2394776	false
	CPOR 25	42192812	42348728	18317388	18443448	-	#D284F9	155916	126060	102	12	102	156018	false
	CPOR 25	42348740	43123531	15134859	15780675	+	#D284F9	774791	645816	12	102	12	774803	false
	CPOR 25	43123633	43665323	16069837	16519037	+	#D284F9	541690	449200	102	-8020	102	541792	false
	CPOR 25	43657303	43722629	22630074	22697333	-	#D284F9	65326	67259	-8020	-65326	-8020	57306	false
	CPOR 25	43657303	43764113	18451422	18546009	-	#D284F9	106810	94587	-65326	-3359	-65326	41484	false
	CPOR 25	43760754	43825376	16548332	16616423	-	#D284F9	64622	68091	-3359	-43724	-3359	61263	false
	CPOR 25	43781652	44132331	16616790	17015710	+	#D284F9	350679	398920	-43724	49334	-43724	306955	false
	CPOR 25	44181665	45121125	17092179	18008085	+	#D284F9	939460	915906	49334	48457	49334	988794	false
	CPOR 25	45169582	45226794	18064162	18117857	+	#D284F9	57212	53695	48457	0	48457	105669	false
	CPOR 25	45226794	46824921	13490545	15082206	-	#D284F9	1598127	1591661	0	18383	0	1598127	false
	CPOR 25	46843304	46900501	12902806	12962057	+	#D284F9	57197	59251	18383	26437	18383	75580	false
	CPOR 25	46926938	46996410	13222828	13292850	-	#D284F9	69472	70022	26437	176889	26437	95909	false
	CPOR 25	47173299	48112525	11983535	12924528	-	#D284F9	939226	940993	176889	115146	176889	1116115	false
	CPOR 25	48227671	48307271	11693576	11783413	+	#D284F9	79600	89837	115146	-5099	115146	194746	false

	CPOR 25	48302172	49910560	10083009	11599256	-	#D284F9	1608388	1516247	-5099	51256	-5099	1603289	false
	CPOR 25	49961816	52922694	7227135	10082974	-	#D284F9	2960878	2855839	51256	3142	51256	3012134	false
	CPOR 25	52925836	54891463	5325966	7082372	-	#D284F9	1965627	1756406	3142	12200	3142	1968769	false
	CPOR 25	54903663	59881074	524448	5260769	-	#D284F9	4977411	4736321	12200		12200	4989611	false
	CPOR 1	59944564	65110082	148796938	152094638	-	#C18B50	5165518	3297700		143956			
	CPOR 1	65254038	67460916	147206709	148760299	-	#C18B50	2206878	1553590	143956	211472	143956	2350834	false
	CPOR 1	67672388	67884683	147078635	147175343	-	#C18B50	212295	96708	211472	128768	211472	423767	false
	CPOR 1	68013451	69264177	146163737	147069433	-	#C18B50	1250726	905696	128768	52618	128768	1379494	false
	CPOR 1	69316795	70762714	145034478	146131977	-	#C18B50	1445919	1097499	52618	56573	52618	1498537	false
	CPOR 1	70819287	83210092	134328082	144991921	-	#C18B50	12390805	10663839	56573	76957	56573	12447378	false
	CPOR 1	83287049	86584307	131228161	134310165	-	#C18B50	3297258	3082004	76957	-46689	76957	3374215	false
	CPOR 1	86537618	89059762	128760735	131132083	-	#C18B50	2522144	2371348	-46689	-67163	-46689	2475455	false
	CPOR 1	88992599	89311190	96301079	96548612	+	#C18B50	318591	247533	-67163	-196843	-67163	251428	false
	CPOR 1	89114347	89191959	96484037	96564346	+	#C18B50	77612	80309	-196843	214575	-196843	-119231	true
	CPOR 1	89406534	93382723	96761869	100428664	+	#C18B50	3976189	3666795	214575	51587	214575	4190764	false
	CPOR 1	93434310	103759625	100443034	109866511	+	#C18B50	10325315	9423477	51587	-142970	51587	10376902	false
	CPOR 1	103616655	103710143	109780005	109901116	-	#C18B50	93488	121111	-142970	39528	-142970	-49482	true
	CPOR 1	103749671	108212397	109971886	113971566	+	#C18B50	4462726	3999680	39528	59931	39528	4502254	false
	CPOR 1	108272328	108340816	113972481	114042507	-	#C18B50	68488	70026	59931	170493	59931	128419	false
	CPOR 1	108511309	109723848	114065863	115104227	+	#C18B50	1212539	1038364	170493	-68007	170493	1383032	false
	CPOR 1	109655841	110964572	115149704	116533460	+	#C18B50	1308731	1383756	-68007	148929	-68007	1240724	false
	CPOR 1	111113501	112818630	116846056	118476358	+	#C18B50	1705129	1630302	148929	-23112	148929	1854058	false
	CPOR 1	112795518	112853597	118542152	118632418	+	#C18B50	58079	90266	-23112	13967	-23112	34967	false
	CPOR 1	112867564	112929863	118468755	118534450	+	#C18B50	62299	65695	13967	-61370	13967	76266	false
	CPOR 1	112868493	119396311	118550802	125013361	+	#C18B50	6527818	6462559	-61370	19829	-61370	6466448	false
	CPOR 1	119416140	119616465	125187866	125304700	+	#C18B50	200325	116834	19829	-199671	19829	220154	false
	CPOR 1	119416794	119619241	125274641	125408605	+	#C18B50	202447	133964	-199671	-202186	-199671	2776	false
	CPOR 1	119417055	119830094	125344511	125709186	+	#C18B50	413039	364675	-202186	59341	-202186	210853	false
	CPOR 1	119889435	120319916	125709930	126147333	+	#C18B50	430481	437403	59341	100000	59341	489822	false
	CPOR 1	120419916	120705588	125978794	126242482	-	#C18B50	285672	263688	100000	10	100000	385672	false
	CPOR 1	120705598	120801192	125797838	125888327	-	#C18B50	95594	90489	10	164599	10	95604	false
	CPOR 1	120965791	121061215	128407081	128468714	+	#C18B50	95424	61633	164599	-95424	164599	260023	false

	CPOR 1	120965791	121098587	127918081	127988252	+	#C18B50	132796	70171	-95424	22648451	-95424	37372	false
	CPOR 1	143747038	143848358	128407081	128468714	+	#C18B50	101320	61633	22648451	-101320	22648451	22749771	false
	CPOR 1	143747038	143886008	127918081	127988252	+	#C18B50	138970	70171	-101320	-86153	-101320	37650	false
	CPOR 1	143799855	143909665	92032282	92201508	+	#C18B50	109810	169226	-86153	1690866	-86153	23657	false
	CPOR 31	143938351	144044467	16614073	16715721	-	#FF00FF	106116	101648	22547279	844848	22547279	22653395	false
	CPOR 31	144889315	145128659	16477216	16715302	+	#FF00FF	239344	238086	844848	25910130	844848	1084192	false
	CPOR 1	145600531	146050941	127263803	127722493	+	#C18B50	450410	458690	1690866	98229	1690866	2141276	false
	CPOR 1	146149170	146328881	125797838	125965663	+	#C18B50	179711	167825	98229	614479	98229	277940	false
	CPOR 1	146943360	147049727	126178761	126242482	-	#C18B50	106367	63721	614479	68904	614479	720846	false
	CPOR 1	147118631	147456612	126235206	126568450	+	#C18B50	337981	333244	68904	-23773	68904	406885	false
	CPOR 1	147432839	147483685	126591491	126658460	-	#C18B50	50846	66969	-23773	316	-23773	27073	false
	CPOR 1	147484001	148009863	126679692	127328295	+	#C18B50	525862	648603	316	55551	316	526178	false
	CPOR 1	148065414	148143616	126167479	126242482	+	#C18B50	78202	75003	55551	456496	55551	133753	false
	CPOR 1	148600112	149098639	125797838	126242482	+	#C18B50	498527	444644	456496	99119	456496	955023	false
	CPOR 1	149197758	149471527	125797838	126147333	-	#C18B50	273769	349495	99119	185993	99119	372888	false
	CPOR 1	149657520	149756565	128407081	128468714	+	#C18B50	99045	61633	185993	-99045	185993	285038	false
	CPOR 1	149657520	149793950	127918081	127988252	+	#C18B50	136430	70171	-99045	-85476	-99045	37385	false
	CPOR 1	149708474	151387494	92032282	93692526	+	#C18B50	1679020	1660244	-85476	3290	-85476	1593544	false
	CPOR 1	151390784	152247036	93863721	94781398	+	#C18B50	856252	917677	3290	51018	3290	859542	false
	CPOR 1	152298054	152650366	94765605	95155207	+	#C18B50	352312	389602	51018	-84647	51018	403330	false
	CPOR 1	152565719	152698767	95199248	95282652	-	#C18B50	133048	83404	-84647	-131404	-84647	48401	false
	CPOR 1	152567363	152698767	95229786	95352816	+	#C18B50	131404	123030	-131404	-21710	-131404	0	true
	CPOR 1	152677057	153034950	95314612	95830301	+	#C18B50	357893	515689	-21710	4560	-21710	336183	false
	CPOR 1	153039510	153414439	95960440	96246709	+	#C18B50	374929	286269	4560		4560	379489	false
	CPOR 4	153503215	154613939	40235576	41202299	-	#C008AD	1110724	966723		-53426			
	CPOR 4	154560513	154661804	40093572	40231802	+	#C008AD	101291	138230	-53426	410	-53426	47865	false
	CPOR 4	154662214	155681866	39249947	40089695	-	#C008AD	1019652	839748	410	1988	410	1020062	false
	CPOR 4	155683854	157192434	38004956	39329654	-	#C008AD	1508580	1324698	1988		1988	1510568	false
	CPOR 7	157489667	158186144	51054547	51704827	-	#2DCDC1	696477	650280		16046			
	CPOR 7	158202190	158293677	50909445	50980578	-	#2DCDC1	91487	71133	16046	-41380	16046	107533	false
	CPOR 7	158252297	158331926	50045530	50118551	+	#2DCDC1	79629	73021	-41380	20727	-41380	38249	false
	CPOR 7	158352653	158446014	49540502	49648672	-	#2DCDC1	93361	108170	20727	-51487	20727	114088	false

CPOR 7	158394527	158804762	49145355	49541511	-	#2DCDC1	410235	396156	-51487	-18946	-51487	358748	false
CPOR 7	158785816	159062877	48932324	49124177	-	#2DCDC1	277061	191853	-18946	-226531	-18946	258115	false
CPOR 7	158836346	160887868	46865117	48977771	-	#2DCDC1	2051522	2112654	-226531	-2015509	-226531	1824991	false
CPOR 7	158872359	159062462	48984841	49038423	-	#2DCDC1	190103	53582	-2015509	1882449	-2015509	-1825406	true
CPOR 7	160944911	161429252	46166927	46616170	-	#2DCDC1	484341	449243	1882449	74122	1882449	2366790	false
CPOR 7	161503374	161611327	46045152	46136359	-	#2DCDC1	107953	91207	74122	-20211	74122	182075	false
CPOR 7	161591116	166705127	41358673	46128334	-	#2DCDC1	5114011	4769661	-20211	103456	-20211	5093800	false
CPOR 7	166808583	170012691	38397393	41358673	-	#2DCDC1	3204108	2961280	103456	-94494	103456	3307564	false
CPOR 7	169918197	170930989	37081223	38159179	+	#2DCDC1	1012792	1077956	-94494		-94494	918298	false
CPOR 31	171038789	171345812	12155382	12374839	-	#FF00FF	307023	219457	25910130	-86752	25910130	26217153	false
CPOR 31	171259060	174043149	9888293	12153897	-	#FF00FF	2784089	2265604	-86752	53198	-86752	2697337	false
CPOR 31	174096347	184974136	7	9857103	-	#FF00FF	10877789	9857096	53198	19099	53198	10930987	false
CPOR 31	184993235	194165612	27325794	35772809	-	#FF00FF	9172377	8447015	19099	56958	19099	9191476	false
CPOR 31	194222570	194325039	27073135	27161317	+	#FF00FF	102469	88182	56958	74407	56958	159427	false
CPOR 31	194399446	196832715	25221870	27073041	-	#FF00FF	2433269	1851171	74407	21711	74407	2507676	false
CPOR 31	196854426	197009599	25222691	25286863	-	#FF00FF	155173	64172	21711	-74276	21711	176884	false
CPOR 31	196935323	197024723	25150004	25219280	+	#FF00FF	89400	69276	-74276	8310	-74276	15124	false
CPOR 31	197033033	200622446	21792805	25149766	-	#FF00FF	3589413	3356961	8310	0	8310	3597723	false
CPOR 31	200622446	201975171	20523834	21734580	-	#FF00FF	1352725	1210746	0	5067	0	1352725	false
CPOR 31	201980238	203892016	18743913	20453423	-	#FF00FF	1911778	1709510	5067	40103	5067	1916845	false
CPOR 31	203932119	205851601	16949299	18672856	-	#FF00FF	1919482	1723557	40103	56925	40103	1959585	false
CPOR 31	205908526	207657825	15357207	16942696	-	#FF00FF	1749299	1585489	56925	7931	56925	1806224	false
CPOR 31	207665756	209684464	13494144	15457550	-	#FF00FF	2018708	1963406	7931	30329320	7931	2026639	false
CPOR 27	209684464	211398034	34722178	36453560	-	#682292	1713570	1731382		114023			
CPOR 27	211512057	222447648	41463972	49577327	+	#682292	10935591	8113355	114023	73403	114023	11049614	false
CPOR 27	222521051	223558898	49581903	50149778	+	#682292	1037847	567875	73403	50074	73403	1111250	false
CPOR 27	223608972	223847471	50149856	50259589	+	#682292	238499	109733	50074	194817	50074	288573	false
CPOR 27	224042288	224193005	50271663	50363707	+	#682292	150717	92044	194817	34117	194817	345534	false
CPOR 27	224227122	224810562	50443862	50726178	+	#682292	583440	282316	34117	147806	34117	617557	false
CPOR 27	224958368	225881306	50752313	51422768	+	#682292	922938	670455	147806	1026240	147806	1070744	false
CPOR 27	226907546	227463930	36825576	37302869	+	#682292	556384	477293	1026240	13995355	1026240	1582624	false
CPOR 20	228661429	229163594	27470819	27646102	+	#00FF7F	502165	175283		52799			

	CPOR 20	229216393	234490100	27646203	31238646	+	#00FF7F	5273707	3592443	52799	1413	52799	5326506	false
	CPOR 20	234491513	235094180	31238658	31695527	-	#00FF7F	602667	456869	1413	5260	1413	604080	false
	CPOR 20	235099440	239931861	31696839	35694920	+	#00FF7F	4832421	3998081	5260		5260	4837681	false
	CPOR 31	240013784	241450246	12403809	13485510	+	#FF00FF	1436462	1081701	30329320		30329320	31765782	false
	CPOR 27	241459285	241891115	36456683	36824731	+	#682292	431830	368048	13995355	104530	13995355	14427185	false
	CPOR 27	241995645	243024826	40603800	41424640	-	#682292	1029181	820840	104530	88861	104530	1133711	false
	CPOR 27	243113687	246962717	37305534	40575811	-	#682292	3849030	3270277	88861		88861	3937891	false
	CPOR 2	247370354	247620680	9342588	9595029	+	#766A7A	250326	252441		27148			
	CPOR 2	247647828	247742255	9840105	9912384	-	#766A7A	94427	72279	27148	140812	27148	121575	false
	CPOR 2	247883067	248061956	31397967	31578150	+	#766A7A	178889	180183	140812	-142058	140812	319701	false
	CPOR 2	247919898	248048153	32041363	32097859	+	#766A7A	128255	56496	-142058	-125339	-142058	-13803	true
	CPOR 2	247922814	248100738	31676999	31761232	-	#766A7A	177924	84233	-125339	-136537	-125339	52585	false
	CPOR 2	247964201	248048153	30617383	30675271	-	#766A7A	83952	57888	-136537	-83952	-136537	-52585	true
	CPOR 2	247964201	248066743	31431893	31518216	+	#766A7A	102542	86323	-83952	112760	-83952	18590	false
	CPOR 2	248179503	248282329	32129205	32201114	-	#766A7A	102826	71909	112760	116234	112760	215586	false
	CPOR 2	248398563	248459786	29373282	29450388	+	#766A7A	61223	77106	116234	229234	116234	177457	false
	CPOR 2	248689020	248779635	28652766	28741563	+	#766A7A	90615	88797	229234	-78506	229234	319849	false
	CPOR 2	248701129	248918969	28743735	29003940	+	#766A7A	217840	260205	-78506		-78506	139334	false
**HSA 10**	CPOR 20	88592	2260811	35729009	37937599	+	#00FF7F	2172219	2208590		1621			
	CPOR 20	2262432	2409997	37948349	38109655	-	#00FF7F	147565	161306	1621	1638	1621	149186	false
	CPOR 20	2411635	3194890	38112784	38907718	+	#00FF7F	783255	794934	1638	2212787	1638	784893	false
	CPOR 22	3213662	4815017	22504226	24437084	-	#F7FE2D	1601355	1932858		0			
	CPOR 22	4815017	4893552	24540373	24627071	+	#F7FE2D	78535	86698	0	-36236	0	78535	false
	CPOR 22	4857316	4972745	24822170	24913243	+	#F7FE2D	115429	91073	-36236	-50564	-36236	79193	false
	CPOR 22	4922181	5007637	24729095	24838971	+	#F7FE2D	85456	109876	-50564	-48782	-50564	34892	false
	CPOR 22	4958855	5051926	24729195	24838971	-	#F7FE2D	93071	109776	-48782	-56604	-48782	44289	false
	CPOR 22	4995322	5162169	24781206	24913243	-	#F7FE2D	166847	132037	-56604	-32472	-56604	110243	false
	CPOR 22	5129697	5234018	24822568	24911313	+	#F7FE2D	104321	88745	-32472	65701	-32472	71849	false
	CPOR 22	5299719	5382295	26335131	26440086	+	#F7FE2D	82576	104955	65701	10028729	65701	148277	false
	CPOR 20	5407677	12093571	38938654	45256559	-	#00FF7F	6685894	6317905	2212787		2212787	8898681	false
	CPOR 7	12058685	13075460	30875614	31713561	+	#2DCDC1	1016775	837947		22622			
	CPOR 7	13098082	13298547	31769191	31922764	+	#2DCDC1	200465	153573	22622	16237	22622	223087	false

CPOR 7	13314784	14973481	26138671	27593137	-	#2DCDC1	1658697	1454466	16237	53677	16237	1674934	false
CPOR 7	15027158	15411017	25747432	26138671	-	#2DCDC1	383859	391239	53677	12037477	53677	437536	false
CPOR 22	15411024	15871694	9010130	9399818	+	#F7FE2D	460670	389688	10028729	-3073	10028729	10489399	false
CPOR 22	15868621	15944069	17479099	17531192	+	#F7FE2D	75448	52093	-3073	-51271	-3073	72375	false
CPOR 22	15892798	18663949	9484791	11797896	+	#F7FE2D	2771151	2313105	-51271	-127361	-51271	2719880	false
CPOR 22	18536588	18664600	4376907	4436292	-	#F7FE2D	128012	59385	-127361	165	-127361	651	false
CPOR 22	18664765	24560351	12248791	17396251	+	#F7FE2D	5895586	5147460	165	-3283950	165	5895751	false
CPOR 22	21276401	21513484	12103917	12200135	-	#F7FE2D	237083	96218	-3283950	3060585	-3283950	-3046867	true
CPOR 22	24574069	26622661	20222246	21994500	+	#F7FE2D	2048592	1772254	3060585	10845	3060585	5109177	false
CPOR 22	26633506	26872931	21933355	22177271	+	#F7FE2D	239425	243916	10845	254765	10845	250270	false
CPOR 8	27023811	27080089	96160894	96222510	-	#60FA36	56278	61616		-56056			
CPOR 8	27024033	27089379	97893695	97990753	+	#60FA36	65346	97058	-56056	10041335	-56056	9290	false
CPOR 22	27127696	27223115	8309841	8421172	+	#F7FE2D	95419	111331	254765	-74902	254765	350184	false
CPOR 22	27148213	27242291	22186050	22282258	-	#F7FE2D	94078	96208	-74902		-74902	19176	false
CPOR 7	27448494	28639252	31981570	33122346	+	#2DCDC1	1190758	1140776	12037477	5403	12037477	13228235	false
CPOR 7	28644655	29400317	27789912	28424257	+	#2DCDC1	755662	634345	5403	43558	5403	761065	false
CPOR 7	29443875	30248826	28627514	29304875	-	#2DCDC1	804951	677361	43558	161120	43558	848509	false
CPOR 7	30409946	30597723	28410063	28537102	-	#2DCDC1	187777	127039	161120	89363	161120	348897	false
CPOR 7	30687086	32420888	29263033	30792989	+	#2DCDC1	1733802	1529956	89363	24504	89363	1823165	false
CPOR 7	32445392	32586551	35439059	35543045	-	#2DCDC1	141159	103986	24504	218374	24504	165663	false
CPOR 7	32804925	35222403	33144895	35333612	-	#2DCDC1	2417478	2188717	218374	4750	218374	2635852	false
CPOR 7	35227153	36885576	35564389	37023384	-	#2DCDC1	1658423	1458995	4750		4750	1663173	false
CPOR 8	37130714	37200833	98020226	98071953	-	#60FA36	70119	51727	10041335	-70119	10041335	10111454	false
CPOR 8	37130714	37230832	101817427	101905142	-	#60FA36	100118	87715	-70119		-70119	29999	false
CPOR 24	37130714	37246935	46467129	46552384	+	#00FFFF	116221	85255	10041643	508844	10041643	10157864	false
CPOR 24	37755779	37903698	46573057	46710583	-	#00FFFF	147919	137526	508844	43136	508844	656763	false
CPOR 24	37946834	38057630	46572247	46623603	-	#00FFFF	110796	51356	43136	4346116	43136	153932	false
CPOR 24	42403746	42455418	48942143	49037864	+	#00FFFF	51672	95721	4346116	328605	4346116	4397788	false
CPOR 24	42784023	43462053	46711692	47078475	+	#00FFFF	678030	366783	328605	93534	328605	1006635	false
CPOR 24	43555587	45035319	47087706	47968481	+	#00FFFF	1479732	880775	93534	219913	93534	1573266	false
CPOR 24	45255232	45309346	48292325	48342648	-	#00FFFF	54114	50323	219913	64499	219913	274027	false
CPOR 24	45373845	45723178	48382880	48635153	+	#00FFFF	349333	252273	64499	683	64499	413832	false

CPOR 24	45723861	45800807	48595571	48649017	-	#00FFFF	76946	53446	683	76018	683	77629	false
CPOR 24	45876825	46035758	49413493	49544071	-	#00FFFF	158933	130578	76018	196835	76018	234951	false
CPOR 24	46232593	46329312	51094823	51176569	-	#00FFFF	96719	81746	196835	-94678	196835	293554	false
CPOR 24	46234634	46453372	51094823	51265823	+	#00FFFF	218738	171000	-94678	61643	-94678	124060	false
CPOR 24	46515015	46688793	51277873	51408979	+	#00FFFF	173778	131106	61643	188291	61643	235421	false
CPOR 24	46877084	47454439	50692792	51125810	+	#00FFFF	577355	433018	188291	197173	188291	765646	false
CPOR 24	47651612	47780423	51299811	51408979	-	#00FFFF	128811	109168	197173	150829	197173	325984	false
CPOR 24	47931252	47992022	51179286	51265823	-	#00FFFF	60770	86537	150829	71554	150829	211599	false
CPOR 24	48063576	49969746	49443393	50773785	-	#00FFFF	1906170	1330392	71554	52251	71554	1977724	false
CPOR 24	50021997	50141381	48595571	48654311	-	#00FFFF	119384	58740	52251	30117428	52251	171635	false
CPOR 28	50194392	50655359	39978936	40403866	+	#7FBF7F	460967	424930		133251			
CPOR 28	50788610	56209738	35538654	39922311	-	#7FBF7F	5421128	4383657	133251	57288	133251	5554379	false
CPOR 28	56267026	56351510	37797761	37855147	+	#7FBF7F	84484	57386	57288	-66416	57288	141772	false
CPOR 28	56285094	73659578	20447682	35499388	-	#7FBF7F	17374484	15051706	-66416	-16707341	-66416	17308068	false
CPOR 28	56952237	57013674	33876944	33952783	-	#7FBF7F	61437	75839	-16707341	858880	-16707341	-16645904	true
CPOR 28	57872554	57924078	35100831	35155331	-	#7FBF7F	51524	54500	858880	15817073	858880	910404	false
CPOR 28	73741151	79498116	15484969	20441014	-	#7FBF7F	5756965	4956045	15817073	217100	15817073	21574038	false
CPOR 28	79715216	79778547	40521808	40635908	-	#7FBF7F	63331	114100	217100	267578	217100	280431	false
CPOR 28	80046125	80211745	15331373	15464651	+	#7FBF7F	165620	133278	267578	7025696	267578	433198	false
CPOR 24	80258809	86529279	51707151	56542663	-	#00FFFF	6270470	4835512	30117428	77387	30117428	36387898	false
CPOR 24	86606666	87216735	51313348	51704950	-	#00FFFF	610069	391602	77387		77387	687456	false
CPOR 28	87237441	87300698	40521808	40635908	-	#7FBF7F	63257	114100	7025696	136571	7025696	7088953	false
CPOR 28	87437269	89836583	40521808	42828498	+	#7FBF7F	2399314	2306690	136571	29324	136571	2535885	false
CPOR 28	89865907	89987733	42880599	42961145	+	#7FBF7F	121826	80546	29324	109625	29324	151150	false
CPOR 28	90097358	90259669	43003378	43197187	-	#7FBF7F	162311	193809	109625	-22245	109625	271936	false
CPOR 28	90237424	91721828	43228702	44676762	+	#7FBF7F	1484404	1448060	-22245	1204	-22245	1462159	false
CPOR 28	91723032	94612562	45817809	48398397	-	#7FBF7F	2889530	2580588	1204		1204	2890734	false
CPOR 2	94680127	94852724	34245673	34370748	+	#766A7A	172597	125075		-100088			
CPOR 2	94752636	94854626	34241568	34293098	+	#766A7A	101990	51530	-100088	67152	-100088	1902	false
CPOR 2	94921778	95036547	34241568	34292938	+	#766A7A	114769	51370	67152	198379	67152	181921	false
CPOR 2	95234926	95342175	34425163	34529141	+	#766A7A	107249	103978	198379	-30782	198379	305628	false
CPOR 2	95311393	96790965	34571610	35972802	+	#766A7A	1479572	1401192	-30782	1512	-30782	1448790	false

	CPOR 2	96792477	100371138	36033838	39423303	+	#766A7A	3578661	3389465	1512	-62492	1512	3580173	false
	CPOR 2	100308646	133423664	40143518	71347568	-	#766A7A	33115018	31204050	-62492	-10575594	-62492	33052526	false
	CPOR 2	122848070	122921769	50414955	50474628	+	#766A7A	73699	59673	-10575594	-42587	-10575594	-10501895	true
	CPOR 2	122879182	122955632	50369720	50442266	+	#766A7A	76450	72546	-42587	5083325	-42587	33863	false
**HSA 11**	CPOR 4	192132	335286	104610284	104764596	-	#C008AD	143154	154312		-27547			
	CPOR 4	307739	1577388	103787879	104602038	-	#C008AD	1269649	814159	-27547	7699793	-27547	1242102	false
	CPOR 11	1595851	2224680	41718381	42409164	+	#6E0025	628829	690783		30700			
	CPOR 11	2255380	3077562	42460792	43223705	+	#6E0025	822182	762913	30700	-9775	30700	852882	false
	CPOR 11	3067787	3239511	43479151	43633272	+	#6E0025	171724	154121	-9775	29562931	-9775	161949	false
	CPOR 17	3401523	3521111	36535404	36600602	-	#FAB28C	119588	65198		4295			
	CPOR 17	3525406	3592334	36437925	36514257	+	#FAB28C	66928	76332	4295	64192703	4295	71223	false
	CPOR 5	3605106	4194491	28262902	28792730	-	#1240C3	589385	529828		178362			
	CPOR 5	4372853	4765663	27763529	28198143	-	#1240C3	392810	434614	178362	-74916	178362	571172	false
	CPOR 5	4690747	4827355	27670366	27762198	-	#1240C3	136608	91832	-74916	-135599	-74916	61692	false
	CPOR 5	4691756	4851463	24711675	24795705	-	#1240C3	159707	84030	-135599	-138703	-135599	24108	false
	CPOR 5	4712760	4860911	27021131	27166503	+	#1240C3	148151	145372	-138703	-56756	-138703	9448	false
	CPOR 5	4804155	4890739	27565649	27640416	+	#1240C3	86584	74767	-56756	-86584	-56756	29828	false
	CPOR 5	4804155	4948171	27177285	27288832	+	#1240C3	144016	111547	-86584	-71588	-86584	57432	false
	CPOR 5	4876583	4981378	27430079	27554418	-	#1240C3	104795	124339	-71588	-14098	-71588	33207	false
	CPOR 5	4967280	5085045	27346879	27406502	+	#1240C3	117765	59623	-14098	-50387	-14098	103667	false
	CPOR 5	5034658	5110272	25666045	25753847	-	#1240C3	75614	87802	-50387	-51666	-50387	25227	false
	CPOR 5	5058606	5156322	25073942	25205607	+	#1240C3	97716	131665	-51666	-37958	-51666	46050	false
	CPOR 5	5118364	5188806	25407638	25472933	-	#1240C3	70442	65295	-37958	-70442	-37958	32484	false
	CPOR 5	5118364	5681220	23519446	23994135	-	#1240C3	562856	474689	-70442	-63767	-70442	492414	false
	CPOR 5	5617453	5713072	23266574	23456827	-	#1240C3	95619	190253	-63767	-15991	-63767	31852	false
	CPOR 5	5697081	5804082	22206777	22472554	-	#1240C3	107001	265777	-15991	-30288	-15991	91010	false
	CPOR 5	5773794	5832561	22179801	22230319	+	#1240C3	58767	50518	-30288	11593	-30288	28479	false
	CPOR 5	5844154	5949628	22070967	22153408	-	#1240C3	105474	82441	11593	-65410	11593	117067	false
	CPOR 5	5884218	6747596	21299587	22058485	-	#1240C3	863378	758898	-65410	-38968	-65410	797968	false
	CPOR 5	6708628	6786847	21088406	21221138	-	#1240C3	78219	132732	-38968	-77933	-38968	39251	false
	CPOR 5	6708914	6788537	21023184	21163483	+	#1240C3	79623	140299	-77933	12	-77933	1690	false
	CPOR 5	6788549	6870913	20558656	20648716	-	#1240C3	82364	90060	12	-56233	12	82376	false

CPOR 5	6814680	7728687	19421423	20250173	-	#1240C3	914007	828750	-56233	-1587	-56233	857774	false
CPOR 5	7727100	9275140	17778236	19220483	-	#1240C3	1548040	1442247	-1587	8961514	-1587	1546453	false
CPOR 4	9277181	17379721	96441389	103686347	-	#C008AD	8102540	7244958	7699793	-67479	7699793	15802333	false
CPOR 4	17312242	17386638	96346267	96405543	+	#C008AD	74396	59276	-67479	-72091	-67479	6917	false
CPOR 4	17314547	18118624	95610087	96341110	-	#C008AD	804077	731023	-72091		-72091	731986	false
CPOR 5	18236654	18806863	49297	547692	+	#1240C3	570209	498395	8961514	-5694	8961514	9531723	false
CPOR 5	18801169	19063956	651599	743762	+	#1240C3	262787	92163	-5694	-198331	-5694	257093	false
CPOR 5	18865625	21457782	934437	2919845	+	#1240C3	2592157	1985408	-198331	-2555548	-198331	2393826	false
CPOR 5	18902234	19063969	550310	724301	+	#1240C3	161735	173991	-2555548	1551820	-2555548	-2393813	true
CPOR 5	20615789	20953814	4004791	4256803	-	#1240C3	338025	252012	1551820	-338025	1551820	1889845	false
CPOR 5	20615789	21212976	5137482	5542998	-	#1240C3	597187	405516	-338025	-597187	-338025	259162	false
CPOR 5	20615789	21326952	2919847	3526186	+	#1240C3	711163	606339	-597187	-711163	-597187	113976	false
CPOR 5	20615789	21457782	4256805	4985324	-	#1240C3	841993	728519	-711163	-455005	-711163	130830	false
CPOR 5	21002777	21631665	5809224	6346486	+	#1240C3	628888	537262	-455005	-295804	-455005	173883	false
CPOR 5	21335861	21457782	4985326	5045678	-	#1240C3	121921	60352	-295804	586	-295804	-173883	true
CPOR 5	21458368	24168381	6463817	8545387	+	#1240C3	2710013	2081570	586	-438464	586	2710599	false
CPOR 5	23729917	23788163	8785348	8858575	+	#1240C3	58246	73227	-438464	397756	-438464	-380218	true
CPOR 5	24185919	24287639	8553503	8624930	-	#1240C3	101720	71427	397756	-46706	397756	499476	false
CPOR 5	24240933	24435438	7799194	7887940	-	#1240C3	194505	88746	-46706	-181124	-46706	147799	false
CPOR 5	24254314	25583867	8563180	9582810	+	#1240C3	1329553	1019630	-181124	-996947	-181124	1148429	false
CPOR 5	24586920	25975120	9583738	10639693	-	#1240C3	1388200	1055955	-996947	-1388200	-996947	391253	false
CPOR 5	24586920	32796688	11208531	17141136	+	#1240C3	8209768	5932605	-1388200	15284013	-1388200	6821568	false
CPOR 11	32802442	33172332	41364047	41689197	-	#6E0025	369890	325150	29562931	4201	29562931	29932821	false
CPOR 11	33176533	48245061	28566614	41296660	-	#6E0025	15068528	12730046	4201	72907	4201	15072729	false
CPOR 5	48080701	48246590	17592168	17695103	-	#1240C3	165889	102935	15284013	829408	15284013	15449902	false
CPOR 11	48317968	48497804	28346364	28446043	-	#6E0025	179836	99679	72907	1417546	72907	252743	false
CPOR 5	49075998	49243489	46509179	46760380	-	#1240C3	167491	251201	829408	138231	829408	996899	false
CPOR 5	49381720	49457878	46214873	46288109	-	#1240C3	76158	73236	138231	34165	138231	214389	false
CPOR 5	49492043	49660480	45959357	46135679	-	#1240C3	168437	176322	34165	22255143	34165	202602	false
CPOR 11	49915350	50060108	14103575	14179624	+	#6E0025	144758	76049	1417546	-110039	1417546	1562304	false
CPOR 11	49950069	50060782	28381010	28441781	+	#6E0025	110713	60771	-110039	4526836	-110039	674	false
CPOR 11	54587618	54668756	28346364	28403993	-	#6E0025	81138	57629	4526836	-8131	4526836	4607974	false

	CPOR 11	54660625	54734336	13922283	14002603	+	#6E0025	73711	80320	-8131	-71387	-8131	65580	false
	CPOR 11	54662949	54734969	13894577	14003276	+	#6E0025	72020	108699	-71387	607576	-71387	633	false
	CPOR 11	55342545	55473305	14070747	14123903	-	#6E0025	130760	53156	607576	-130760	607576	738336	false
	CPOR 11	55342545	55473678	13888532	14003336	+	#6E0025	131133	114804	-130760	31065	-130760	373	false
	CPOR 11	55504743	55555511	28289082	28343370	-	#6E0025	50768	54288	31065	90883	31065	81833	false
	CPOR 13	55563118	55673257	76695984	76804629	+	#7F00FF	110139	108645		993536			
	CPOR 11	55646394	55726873	8612672	8699459	+	#6E0025	80479	86787	90883	-80218	90883	171362	false
	CPOR 11	55646655	55728558	14368512	14429518	+	#6E0025	81903	61006	-80218	-17708	-80218	1685	false
	CPOR 11	55710850	55800201	8358385	8423085	-	#6E0025	89351	64700	-17708	224644	-17708	71643	false
	CPOR 11	56024845	56123397	15435293	15503740	-	#6E0025	98552	68447	224644	21698	224644	323196	false
	CPOR 11	56145095	56253989	18697252	18754669	-	#6E0025	108894	57417	21698	56776	21698	130592	false
	CPOR 11	56310765	56360973	23578084	23655672	-	#6E0025	50208	77588	56776	-16825	56776	106984	false
	CPOR 11	56344148	56412002	18532310	18584080	-	#6E0025	67854	51770	-16825	-36383	-16825	51029	false
	CPOR 11	56375619	56546289	22977461	23092834	+	#6E0025	170670	115373	-36383	50601	-36383	134287	false
	CPOR 6	56405663	56641400	48156238	48364986	+	#0C404C	235737	208748		-160838			
	CPOR 6	56480562	56801157	7319949	7549918	+	#0C404C	320595	229969	-160838		-160838	159757	false
	CPOR 11	56596890	56663006	23892034	23949902	-	#6E0025	66116	57868	50601	-66116	50601	116717	false
	CPOR 11	56596890	56893191	23105342	23370260	+	#6E0025	296301	264918	-66116	-118260	-66116	230185	false
	CPOR 21	56660495	56742890	10582812	10638346	-	#0000FF	82395	55534		-25438			
	CPOR 13	56666793	56824200	76937866	77082057	-	#7F00FF	157407	144191	993536		993536	1150943	false
	CPOR 21	56717452	56772582	21358158	21418037	-	#0000FF	55130	59879	-25438	-24363	-25438	29692	false
	CPOR 21	56748219	56824200	21251061	21324773	-	#0000FF	75981	73712	-24363		-24363	51618	false
	CPOR 11	56774931	56937909	23765623	23884863	-	#6E0025	162978	119240	-118260	-162978	-118260	44718	false
	CPOR 11	56774931	57019034	23375744	23539760	+	#6E0025	244103	164016	-162978	-178976	-162978	81125	false
	CPOR 11	56840058	57023102	22727653	22929906	-	#6E0025	183044	202253	-178976	-70688	-178976	4068	false
	CPOR 11	56952414	57241109	22473407	22663631	-	#6E0025	288695	190224	-70688	56392	-70688	218007	false
	CPOR 11	57297501	58069886	21961604	22444349	-	#6E0025	772385	482745	56392	39742	56392	828777	false
	CPOR 11	58109628	58410127	21583667	21862667	-	#6E0025	300499	279000	39742	-100017	39742	340241	false
	CPOR 11	58310110	58423306	19898516	19961498	+	#6E0025	113196	62982	-100017	-79780	-100017	13179	false
	CPOR 11	58343526	58410127	19960321	20015241	+	#6E0025	66601	54920	-79780	-8755	-79780	-13179	true
	CPOR 11	58401372	58763784	21005410	21348633	-	#6E0025	362412	343223	-8755	-57636	-8755	353657	false
	CPOR 11	58706148	58768381	20932617	20997579	-	#6E0025	62233	64962	-57636	273420	-57636	4597	false

CPOR 11	59041801	59463801	20382792	20815187	-	#6E0025	422000	432395	273420	-5928	273420	695420	false
CPOR 11	59457873	59541044	20127101	20190564	-	#6E0025	83171	63463	-5928	-43784	-5928	77243	false
CPOR 11	59497260	59853080	19472936	19755572	-	#6E0025	355820	282636	-43784	-32183	-43784	312036	false
CPOR 11	59820897	59875156	19406024	19471715	+	#6E0025	54259	65691	-32183	54194	-32183	22076	false
CPOR 11	59929350	60245111	18996816	19305428	-	#6E0025	315761	308612	54194	26757	54194	369955	false
CPOR 11	60271868	60383230	18995906	19055184	+	#6E0025	111362	59278	26757	-88808	26757	138119	false
CPOR 11	60294422	60514313	18233109	18412910	-	#6E0025	219891	179801	-88808	-33131	-88808	131083	false
CPOR 11	60481182	60630526	17999991	18216162	-	#6E0025	149344	216171	-33131	-20250	-33131	116213	false
CPOR 11	60610276	60709601	17940741	17997641	-	#6E0025	99325	56900	-20250	53700	-20250	79075	false
CPOR 11	60763301	61251224	17730631	17919619	-	#6E0025	487923	188988	53700	7346	53700	541623	false
CPOR 11	61258570	62050038	17195638	17659683	+	#6E0025	791468	464045	7346	151937	7346	798814	false
CPOR 11	62201975	62265723	16645028	16724768	-	#6E0025	63748	79740	151937	71753	151937	215685	false
CPOR 11	62337476	62839931	16154478	16425791	-	#6E0025	502455	271313	71753	12855	71753	574208	false
CPOR 11	62852786	63025523	15958398	16081233	-	#6E0025	172737	122835	12855	54263	12855	185592	false
CPOR 11	63079786	63146573	15755747	15869469	-	#6E0025	66787	113722	54263	11839	54263	121050	false
CPOR 11	63158412	63274439	15755478	15871056	-	#6E0025	116027	115578	11839	91860	11839	127866	false
CPOR 11	63366299	63517749	15755670	15946557	+	#6E0025	151450	190887	91860	31975	91860	243310	false
CPOR 11	63549724	67678162	46585514	50098413	-	#6E0025	4128438	3512899	31975	314821	31975	4160413	false
CPOR 17	67785037	67894382	36535404	36600602	-	#FAB28C	109345	65198	64192703	5003	64192703	64302048	false
CPOR 17	67899385	67966930	36437925	36514257	+	#FAB28C	67545	76332	5003	3661413	5003	72548	false
CPOR 11	67992983	70387428	44683016	46566870	-	#6E0025	2394445	1883854	314821	-115830	314821	2709266	false
CPOR 11	70271598	70955499	44108754	44590653	-	#6E0025	683901	481899	-115830	100163	-115830	568071	false
CPOR 11	71055662	71504575	43705713	44105169	-	#6E0025	448913	399456	100163		100163	549076	false
CPOR 17	71628343	71696683	36437925	36514257	-	#FAB28C	68340	76332	3661413	4298	3661413	3729753	false
CPOR 17	71700981	71804772	36535404	36597606	+	#FAB28C	103791	62202	4298		4298	108089	false
CPOR 5	71915623	75717280	28792732	32276451	+	#1240C3	3801657	3483719	22255143	-14749	22255143	26056800	false
CPOR 5	75702531	76828869	32585909	33643567	+	#1240C3	1126338	1057658	-14749	515	-14749	1111589	false
CPOR 5	76829384	82715514	35042807	40394346	+	#1240C3	5886130	5351539	515	2613	515	5886645	false
CPOR 5	82718127	85470439	40451607	42929195	+	#1240C3	2752312	2477588	2613	52707	2613	2754925	false
CPOR 5	85523146	88856240	42964571	45893360	+	#1240C3	3333094	2928789	52707	102512	52707	3385801	false
CPOR 5	88958752	89324456	45959357	46288109	+	#1240C3	365704	328752	102512	81364	102512	468216	false
CPOR 5	89405820	89759861	46354675	46760380	+	#1240C3	354041	405705	81364	320244	81364	435405	false

	CPOR 5	90080105	90289732	46722833	46947141	+	#1240C3	209627	224308	320244	57908	320244	529871	false
	CPOR 5	90347640	95059888	46958892	51208376	+	#1240C3	4712248	4249484	57908	-46870	57908	4770156	false
	CPOR 5	95013018	98408622	51562965	54458862	+	#1240C3	3395604	2895897	-46870	8	-46870	3348734	false
	CPOR 5	98408630	98683473	54460625	54619576	-	#1240C3	274843	158951	8	0	8	274851	false
	CPOR 5	98683473	101859032	54620154	57252883	+	#1240C3	3175559	2632729	0	526	0	3175559	false
	CPOR 5	101859558	105141179	57305596	60238594	+	#1240C3	3281621	2932998	526	-160413	526	3282147	false
	CPOR 5	104980766	105132144	60146040	60228371	+	#1240C3	151378	82331	-160413	-138011	-160413	-9035	true
	CPOR 5	104994133	105138733	60260619	60356777	-	#1240C3	144600	96158	-138011	-3454	-138011	6589	false
	CPOR 5	105135279	105298388	60368311	60561864	+	#1240C3	163109	193553	-3454	60842	-3454	159655	false
	CPOR 5	105359230	106376898	60612278	61542438	+	#1240C3	1017668	930160	60842	0	60842	1078510	false
	CPOR 5	106376898	106581964	62365974	62610940	+	#1240C3	205066	244966	0	299	0	205066	false
	CPOR 5	106582263	107490197	61569781	62281512	-	#1240C3	907934	711731	299	2836	299	908233	false
	CPOR 5	107493033	114479473	84686338	91323700	-	#1240C3	6986440	6637362	2836	40107	2836	6989276	false
	CPOR 5	114519580	114598421	84457456	84520936	-	#1240C3	78841	63480	40107	51604	40107	118948	false
	CPOR 5	114650025	124114498	75677546	84448366	-	#1240C3	9464473	8770820	51604	-64196	51604	9516077	false
	CPOR 5	124050302	124315812	74527969	74847795	+	#1240C3	265510	319826	-64196	-159614	-64196	201314	false
	CPOR 5	124156198	124368125	75364223	75593999	+	#1240C3	211927	229776	-159614	-198194	-159614	52313	false
	CPOR 5	124169931	124401501	73700783	73927208	-	#1240C3	231570	226425	-198194	-183637	-198194	33376	false
	CPOR 5	124217864	124368050	75011585	75162757	+	#1240C3	150186	151172	-183637	-59762	-183637	-33451	true
	CPOR 5	124308288	124399276	73207812	73327990	-	#1240C3	90988	120178	-59762	-76928	-59762	31226	false
	CPOR 5	124322348	124404544	73639981	73703054	+	#1240C3	82196	63073	-76928	-30782	-76928	5268	false
	CPOR 5	124373762	124518419	73082885	73197634	-	#1240C3	144657	114749	-30782	2218	-30782	113875	false
	CPOR 5	124520637	124571461	72961357	73052201	-	#1240C3	50824	90844	2218	-2673	2218	53042	false
	CPOR 5	124568788	134345146	63459122	72882823	-	#1240C3	9776358	9423701	-2673	-68323	-2673	9773685	false
	CPOR 5	134276823	134885434	62919520	63466905	-	#1240C3	608611	547385	-68323		-68323	540288	false
**HSA 12**	CPOR 9	952073	2745935	65105595	66826945	+	#C2FB8F	1793862	1721350					
	CPOR 22	2788219	2844599	4584049	4655538	-	#F7FE2D	56380	71489		-23			
	CPOR 22	2844576	6278419	962570	4213966	+	#F7FE2D	3433843	3251396	-23	2997	-23	3433820	false
	CPOR 22	6281416	7221824	62039	908746	-	#F7FE2D	940408	846707	2997	114784	2997	943405	false
	CPOR 22	7336608	7602483	28226569	28313549	+	#F7FE2D	265875	86980	114784	45823	114784	380659	false
	CPOR 22	7648306	7744892	29862500	29957755	+	#F7FE2D	96586	95255	45823	174964	45823	142409	false
	CPOR 22	7919856	8142244	30530971	30756593	+	#F7FE2D	222388	225622	174964	-36412	174964	397352	false

	CPOR 22	8105832	8157259	28606784	28696367	-	#F7FE2D	51427	89583	-36412	294845	-36412	15015	false
	CPOR 17	8226566	8329904	36535404	36598244	-	#FAB28C	103338	62840		4275			
	CPOR 17	8334179	8407894	36437925	36514257	+	#FAB28C	73715	76332	4275	99505534	4275	77990	false
	CPOR 22	8452104	8543743	28568007	28657546	-	#F7FE2D	91639	89539	294845	54312	294845	386484	false
	CPOR 22	8598055	8700584	30213207	30272352	+	#F7FE2D	102529	59145	54312	-102529	54312	156841	false
	CPOR 22	8598055	9020280	29443085	29799507	-	#F7FE2D	422225	356422	-102529	4228	-102529	319696	false
	CPOR 22	9024508	9134785	29216984	29380303	-	#F7FE2D	110277	163319	4228	23184	4228	114505	false
	CPOR 22	9157969	9218313	29113155	29192426	+	#F7FE2D	60344	79271	23184	261777	23184	83528	false
	CPOR 22	9480090	9565954	28976976	29044435	+	#F7FE2D	85864	67459	261777	368921	261777	347641	false
	CPOR 22	9934875	10315710	32123830	32464960	-	#F7FE2D	380835	341130	368921	55033	368921	749756	false
	CPOR 22	10370743	10498493	31955449	32042463	-	#F7FE2D	127750	87014	55033	-127750	55033	182783	false
	CPOR 22	10370743	10510559	31596576	31682611	-	#F7FE2D	139816	86035	-127750	-15396	-127750	12066	false
	CPOR 22	10495163	10596865	31712842	31901969	-	#F7FE2D	101702	189127	-15396	-101702	-15396	86306	false
	CPOR 22	10495163	10837104	30965704	31376251	-	#F7FE2D	341941	410547	-101702	-29860	-101702	240239	false
	CPOR 22	10807244	10886475	30810973	30945142	-	#F7FE2D	79231	134169	-29860	8350	-29860	49371	false
	CPOR 22	10894825	11075616	32558364	32651509	+	#F7FE2D	180791	93145	8350	-138480	8350	189141	false
	CPOR 22	10937136	11195940	32977548	33075608	+	#F7FE2D	258804	98060	-138480	30144	-138480	120324	false
	CPOR 22	11226084	14213249	33357204	36107717	+	#F7FE2D	2987165	2750513	30144	-2937048	30144	3017309	false
	CPOR 22	11276201	11423698	33405113	33464402	-	#F7FE2D	147497	59289	-2937048	-103650	-2937048	-2789551	true
	CPOR 22	11320048	11430511	30818688	30879492	+	#F7FE2D	110463	60804	-103650	2807137	-103650	6813	false
	CPOR 22	14237648	17764355	36190022	39357353	+	#F7FE2D	3526707	3167331	2807137	97206	2807137	6333844	false
	CPOR 22	17861561	19790841	39406559	40963150	+	#F7FE2D	1929280	1556591	97206	0	97206	2026486	false
	CPOR 22	19790841	19898974	40978313	41091900	-	#F7FE2D	108133	113587	0	5	0	108133	false
	CPOR 22	19898979	20918563	41103764	42022164	+	#F7FE2D	1019584	918400	5	-109827	5	1019589	false
	CPOR 22	20808736	20932695	42149260	42274749	-	#F7FE2D	123959	125489	-109827	-123751	-109827	14132	false
	CPOR 22	20808944	21120620	42146708	42338964	-	#F7FE2D	311676	192256	-123751	-240881	-123751	187925	false
	CPOR 22	20879739	21090619	42415452	42555366	-	#F7FE2D	210880	139914	-240881	-173683	-240881	-30001	true
	CPOR 22	20916936	21090912	42583225	42675756	-	#F7FE2D	173976	92531	-173683	-132007	-173683	293	false
	CPOR 22	20958905	21106474	41972498	42029970	+	#F7FE2D	147569	57472	-132007	-92428	-132007	15562	false
	CPOR 22	21014046	21430338	42031879	42326500	-	#F7FE2D	416292	294621	-92428	-308355	-92428	323864	false
	CPOR 22	21121983	21255112	41961664	42017647	+	#F7FE2D	133129	55983	-308355	-125381	-308355	-175226	true
	CPOR 22	21129731	21255700	42272213	42338964	-	#F7FE2D	125969	66751	-125381	-1342	-125381	588	false

	CPOR 22	21254358	21306802	42338964	42411205	-	#F7FE2D	52444	72241	-1342	127827	-1342	51102	false
	CPOR 22	21434629	23636528	42722734	44663963	+	#F7FE2D	2201899	1941229	127827	4092	127827	2329726	false
	CPOR 22	23640620	27040704	44802215	47753260	+	#F7FE2D	3400084	2951045	4092	3792248	4092	3404176	false
	CPOR 8	27063978	27148407	72204076	72296326	-	#60FA36	84429	92250		1484			
	CPOR 8	27149891	27332558	43733	254108	-	#60FA36	182667	210375	1484	176297	1484	184151	false
	CPOR 8	27508855	30282681	69514562	72401448	-	#60FA36	2773826	2886886	176297	-723547	176297	2950123	false
	CPOR 8	29559134	29621470	69429463	69503513	+	#60FA36	62336	74050	-723547	41832	-723547	-661211	true
	CPOR 8	29663302	29745213	69705748	69805045	-	#60FA36	81911	99297	41832	-24983	41832	123743	false
	CPOR 8	29720230	29801418	68188922	68256260	+	#60FA36	81188	67338	-24983	115260	-24983	56205	false
	CPOR 8	29916678	30171176	70071111	70324637	-	#60FA36	254498	253526	115260	18875	115260	369758	false
	CPOR 8	30190051	30282142	70036904	70150693	+	#60FA36	92091	113789	18875	98557	18875	110966	false
	CPOR 8	30380699	30532212	69385267	69514524	-	#60FA36	151513	129257	98557	-91632	98557	250070	false
	CPOR 8	30440580	30564412	70243563	70324404	+	#60FA36	123832	80841	-91632	52506	-91632	32200	false
	CPOR 8	30616918	30791643	69140237	69322060	-	#60FA36	174725	181823	52506	457645	52506	227231	false
	CPOR 22	30832952	31012395	1371120	1590482	-	#F7FE2D	179443	219362	3792248	112324	3792248	3971691	false
	CPOR 22	31124719	31211945	28968070	29044435	-	#F7FE2D	87226	76365	112324		112324	199550	false
	CPOR 8	31249288	31839479	68701167	69137380	-	#60FA36	590191	436213	457645	116164	457645	1047836	false
	CPOR 8	31955643	32055908	68322590	68401208	-	#60FA36	100265	78618	116164	7928	116164	216429	false
	CPOR 8	32063836	33997133	66136929	68117680	-	#60FA36	1933297	1980751	7928	4211980	7928	1941225	false
	CPOR 8	38209113	38291177	66136929	66265067	-	#60FA36	82064	128138	4211980	76614	4211980	4294044	false
	CPOR 8	38367791	42339259	62201518	66077581	-	#60FA36	3971468	3876063	76614	-74100	76614	4048082	false
	CPOR 8	42265159	47460185	57168232	62179203	-	#60FA36	5195026	5010971	-74100	-2032	-74100	5120926	false
	CPOR 8	47458153	47510641	57159769	57215491	+	#60FA36	52488	55722	-2032	3903	-2032	50456	false
	CPOR 8	47514544	48443087	56195805	57159769	-	#60FA36	928543	963964	3903	-12188	3903	932446	false
	CPOR 8	48430899	48523350	56064104	56147892	-	#60FA36	92451	83788	-12188	31036	-12188	80263	false
	CPOR 8	48554386	51038041	53617115	55879444	-	#60FA36	2483655	2262329	31036	-103574	31036	2514691	false
	CPOR 8	50934467	51041576	53518308	53614426	-	#60FA36	107109	96118	-103574	-89007	-103574	3535	false
	CPOR 8	50952569	52493528	52164736	53516798	-	#60FA36	1540959	1352062	-89007	-46466	-89007	1451952	false
	CPOR 8	52447062	54768372	49925491	52170319	-	#60FA36	2321310	2244828	-46466	181357	-46466	2274844	false
	CPOR 8	54949729	55096950	49724691	49917370	-	#60FA36	147221	192679	181357	28102	181357	328578	false
	CPOR 8	55125052	55224631	49448656	49587443	-	#60FA36	99579	138787	28102	14338	28102	127681	false
	CPOR 8	55238969	55322140	49320046	49391185	-	#60FA36	83171	71139	14338	-74076	14338	97509	false

CPOR 8	55248064	55453539	48322139	48490550	+	#60FA36	205475	168411	-74076	-169926	-74076	131399	false
CPOR 8	55283613	55370432	48147451	48207595	-	#60FA36	86819	60144	-169926	-6131	-169926	-83107	true
CPOR 8	55364301	55453392	48616262	48716652	+	#60FA36	89091	100390	-6131	59037	-6131	82960	false
CPOR 8	55512429	55620509	47816743	47911268	-	#60FA36	108080	94525	59037	46913	59037	167117	false
CPOR 8	55667422	56815580	46331832	47361730	-	#60FA36	1148158	1029898	46913	-42528	46913	1195071	false
CPOR 8	56773052	56960443	45826700	46020523	-	#60FA36	187391	193823	-42528	-24495	-42528	144863	false
CPOR 8	56935948	57637534	45025308	45794320	-	#60FA36	701586	769012	-24495	52014	-24495	677091	false
CPOR 8	57689548	63022766	40128516	45005196	-	#60FA36	5333218	4876680	52014	30205	52014	5385232	false
CPOR 8	63052971	63551845	39526085	40073491	-	#60FA36	498874	547406	30205	179666	30205	529079	false
CPOR 24	63557835	63667932	33488468	33550207	+	#00FFFF	110097	61739		-110097			
CPOR 24	63557835	63725474	30612097	30749135	+	#00FFFF	167639	137038	-110097		-110097	57542	false
CPOR 8	63731511	68258795	35261701	39551956	-	#60FA36	4527284	4290255	179666	0	179666	4706950	false
CPOR 8	68258795	69355329	34131401	35171635	-	#60FA36	1096534	1040234	0	-11506	0	1096534	false
CPOR 8	69343823	71719722	31740144	33934015	-	#60FA36	2375899	2193871	-11506	25125	-11506	2364393	false
CPOR 8	71744847	88697237	17040020	31687784	-	#60FA36	16952390	14647764	25125	16502	25125	16977515	false
CPOR 8	88713739	88773680	16984267	17039335	+	#60FA36	59941	55068	16502	53	16502	76443	false
CPOR 8	88773733	100148402	7113669	16979656	-	#60FA36	11374669	9865987	53	51989	53	11374722	false
CPOR 8	100200391	101832717	5962311	7112535	-	#60FA36	1632326	1150224	51989	55131	51989	1684315	false
CPOR 8	101887848	107786213	2086503	5960510	-	#60FA36	5898365	3874007	55131		55131	5953496	false
CPOR 17	107913428	109270715	54019660	55190923	+	#FAB28C	1357287	1171263	99505534	-75221	99505534	100862821	false
CPOR 17	109195494	110053321	53374380	54004629	-	#FAB28C	857827	630249	-75221	6370	-75221	782606	false
CPOR 17	110059691	112113298	63215808	64689882	-	#FAB28C	2053607	1474074	6370	32	6370	2059977	false
CPOR 17	112113330	114103569	61776340	63154977	-	#FAB28C	1990239	1378637	32	62425	32	1990271	false
CPOR 17	114165994	121046632	55442317	61052760	-	#FAB28C	6880638	5610443	62425	57732	62425	6943063	false
CPOR 17	121104364	124605403	64664730	66701801	+	#FAB28C	3501039	2037071	57732	152764	57732	3558771	false
CPOR 17	124758167	125685611	66742044	67218141	+	#FAB28C	927444	476097	152764	-24598	152764	1080208	false
CPOR 17	125661013	126083725	68419080	68615469	-	#FAB28C	422712	196389	-24598	-390477	-24598	398114	false
CPOR 17	125693248	126395917	69575575	69990596	-	#FAB28C	702669	415021	-390477	-219316	-390477	312192	false
CPOR 17	126176601	126880569	70154756	70588416	+	#FAB28C	703968	433660	-219316	-455617	-219316	484652	false
CPOR 17	126424952	126880569	69267854	69487313	-	#FAB28C	455617	219459	-455617	-123831	-455617	0	true
CPOR 17	126756738	126880569	67957067	68028917	-	#FAB28C	123831	71850	-123831	1756	-123831	0	true
CPOR 17	126882325	126978165	67892185	67956043	+	#FAB28C	95840	63858	1756	16775	1756	97596	false

	CPOR 17	126994940	127053240	69768135	69818450	+	#FAB28C	58300	50315	16775	-56225	16775	75075	false
	CPOR 17	126997015	127290068	67708490	67889883	-	#FAB28C	293053	181393	-56225	-9298	-56225	236828	false
	CPOR 17	127280770	127439656	67044044	67248731	-	#FAB28C	158886	204687	-9298	-93396	-9298	149588	false
	CPOR 17	127346260	127442795	70832213	70884440	+	#FAB28C	96535	52227	-93396	-96535	-93396	3139	false
	CPOR 17	127346260	127925602	68718480	69028446	-	#FAB28C	579342	309966	-96535	-579342	-96535	482807	false
	CPOR 17	127346260	128024927	67298722	67708308	-	#FAB28C	678667	409586	-579342	-211848	-579342	99325	false
	CPOR 17	127813079	128024927	70884856	70993930	+	#FAB28C	211848	109074	-211848	68762	-211848	0	true
	CPOR 17	128093689	128746456	71009989	71302234	+	#FAB28C	652767	292245	68762	51935	68762	721529	false
	CPOR 17	128798391	129764639	71314975	71788287	+	#FAB28C	966248	473312	51935	76598	51935	1018183	false
	CPOR 17	129841237	129994389	71788678	71857674	+	#FAB28C	153152	68996	76598	50687	76598	229750	false
	CPOR 17	130045076	130672866	71857691	72111691	+	#FAB28C	627790	254000	50687	117774	50687	678477	false
	CPOR 17	130790640	131139486	72119226	72270156	+	#FAB28C	348846	150930	117774	251130	117774	466620	false
	CPOR 17	131390616	131636297	72329255	72424912	+	#FAB28C	245681	95657	251130	72554	251130	496811	false
	CPOR 17	131708851	133204347	72425323	73322090	+	#FAB28C	1495496	896767	72554		72554	1568050	false
**HSA 13**	CPOR 8	18820217	18874366	101819742	101906004	+	#60FA36	54149	86262		-54149			
	CPOR 8	18820217	18874747	96157980	96229903	-	#60FA36	54530	71923	-54149		-54149	381	false
	CPOR 24	18821921	18874747	46466090	46541231	-	#00FFFF	52826	75141		390439			
	CPOR 24	19265186	19339989	46481381	46552214	-	#00FFFF	74803	70833	390439		390439	465242	false
	CPOR 19	19409424	19529306	41763341	41894594	+	#8C5319	119882	131253		103811			
	CPOR 19	19633117	21187939	23109931	24577741	+	#8C5319	1554822	1467810	103811	184621	103811	1658633	false
	CPOR 19	21372560	22911586	24584880	25954432	+	#8C5319	1539026	1369552	184621	94611	184621	1723647	false
	CPOR 19	23006197	23895791	25972803	26699353	+	#8C5319	889594	726550	94611	61820	94611	984205	false
	CPOR 19	23957611	24317249	26699353	27032516	-	#8C5319	359638	333163	61820	293845	61820	421458	false
	CPOR 19	24611094	24925161	22670890	23103105	+	#8C5319	314067	432215	293845	130396	293845	607912	false
	CPOR 19	25055557	26098100	26999260	27968472	+	#8C5319	1042543	969212	130396	80110	130396	1172939	false
	CPOR 19	26178210	38330384	27992891	39543343	+	#8C5319	12152174	11550452	80110	0	80110	12232284	false
	CPOR 19	38330384	38889063	39605316	40108138	+	#8C5319	558679	502822	0	64762	0	558679	false
	CPOR 19	38953825	40871558	40108138	41834605	+	#8C5319	1917733	1726467	64762	35550	64762	1982495	false
	CPOR 19	40907108	41088894	51552065	51688297	+	#8C5319	181786	136232	35550	97146	35550	217336	false
	CPOR 19	41186040	41264708	51754573	51810644	+	#8C5319	78668	56071	97146	50893	97146	175814	false
	CPOR 19	41315601	45376501	51830760	55600097	+	#8C5319	4060900	3769337	50893	8304	50893	4111793	false
	CPOR 19	45384805	45456302	55600118	55670584	-	#8C5319	71497	70466	8304	8081	8304	79801	false

	CPOR 19	45464383	46402820	55671061	56500893	+	#8C5319	938437	829832	8081	66124	8081	946518	false
	CPOR 19	46468944	51136864	56501111	61080017	+	#8C5319	4667920	4578906	66124	29072	66124	4734044	false
	CPOR 19	51165936	52160021	42027585	42872963	-	#8C5319	994085	845378	29072	15529	29072	1023157	false
	CPOR 19	52175550	52476575	41720976	42009617	+	#8C5319	301025	288641	15529	21	15529	316554	false
	CPOR 19	52476596	52637533	41720976	41912215	-	#8C5319	160937	191239	21	14443	21	160958	false
	CPOR 19	52651976	54686818	49573308	51566118	-	#8C5319	2034842	1992810	14443	66014	14443	2049285	false
	CPOR 19	54752832	55495495	48895144	49552048	-	#8C5319	742663	656904	66014	307	66014	808677	false
	CPOR 19	55495802	55709941	48682324	48895144	+	#8C5319	214139	212820	307	1261	307	214446	false
	CPOR 19	55711202	61839788	43194217	48681968	-	#8C5319	6128586	5487751	1261	0	1261	6129847	false
	CPOR 19	61839788	61958664	42954484	43097018	-	#8C5319	118876	142534	0		0	118876	false
	CPOR 3	61952113	62014436	3254123	3361699	+	#13C840	62323	107576		15770			
	CPOR 3	62030206	63375214	3454194	4659097	+	#13C840	1345008	1204903	15770	99558	15770	1360778	false
	CPOR 3	63474772	76109609	4660073	16328883	+	#13C840	12634837	11668810	99558	18409	99558	12734395	false
	CPOR 3	76128018	76266715	16329183	16433127	-	#13C840	138697	103944	18409	2033	18409	157106	false
	CPOR 3	76268748	86202257	16434684	25315682	+	#13C840	9933509	8880998	2033	78873	2033	9935542	false
	CPOR 3	86281130	95382590	25316720	33414228	+	#13C840	9101460	8097508	78873	620	78873	9180333	false
	CPOR 3	95383210	110562440	33534887	46071594	+	#13C840	15179230	12536707	620	52818	620	15179850	false
	CPOR 3	110615258	110763766	46084762	46178205	+	#13C840	148508	93443	52818	113982	52818	201326	false
	CPOR 3	110877748	111009431	46179780	46241402	+	#13C840	131683	61622	113982	96430	113982	245665	false
	CPOR 3	111105861	111668893	46247475	46563295	+	#13C840	563032	315820	96430	174523	96430	659462	false
	CPOR 3	111843416	112192219	46664736	46895510	+	#13C840	348803	230774	174523	63194	174523	523326	false
	CPOR 3	112255413	113671678	46939933	47656000	+	#13C840	1416265	716067	63194	51390	63194	1479459	false
	CPOR 3	113723068	114328985	47656420	48011603	+	#13C840	605917	355183	51390		51390	657307	false
**HSA 14**	CPOR 4	20051007	20163476	71523033	71605379	+	#C008AD	112469	82346		720667			
	CPOR 27	20186768	20248826	28438372	28581470	+	#682292	62058	143098		-24099			
	CPOR 27	20224727	20421800	28641508	28821913	-	#682292	197073	180405	-24099	-63957	-24099	172974	false
	CPOR 27	20357843	20668724	28830986	29153566	+	#682292	310881	322580	-63957	28993	-63957	246924	false
	CPOR 27	20697717	20876119	29334598	29473646	-	#682292	178402	139048	28993	8356	28993	207395	false
	CPOR 4	20884143	21011591	36978174	37090581	-	#C008AD	127448	112407	720667	-103785	720667	848115	false
	CPOR 27	20884475	21643676	29572391	30233210	+	#682292	759201	660819	8356	-22684	8356	767557	false
	CPOR 4	20907806	20979986	37035029	37087867	-	#C008AD	72180	52838	-103785		-103785	-31605	true
	CPOR 27	21620992	21924061	30231201	30557725	+	#682292	303069	326524	-22684	-190300	-22684	280385	false

	CPOR 27	21733761	21967242	31532275	31633957	+	#682292	233481	101682	-190300	-218694	-190300	43181	false
	CPOR 27	21748548	22707227	31693423	32480483	+	#682292	958679	787060	-218694	-922681	-218694	739985	false
	CPOR 27	21784546	22133956	31170156	31311474	+	#682292	349410	141318	-922681	-292562	-922681	-573271	true
	CPOR 27	21841394	21895187	31759381	31828134	+	#682292	53793	68753	-292562	-36761	-292562	-238769	true
	CPOR 27	21858426	22003752	30997680	31073338	+	#682292	145326	75658	-36761	-145326	-36761	108565	false
	CPOR 27	21858426	22133956	30822992	30895957	+	#682292	275530	72965	-145326	-263277	-145326	130204	false
	CPOR 27	21870679	22224989	30609193	30799631	+	#682292	354310	190438	-263277	-201713	-263277	91033	false
	CPOR 27	22023276	22224989	31070040	31167539	+	#682292	201713	97499	-201713	-153623	-201713	0	true
	CPOR 27	22071366	22224989	30900783	30972511	+	#682292	153623	71728	-153623	516526	-153623	0	true
	CPOR 27	22741515	23789898	32530841	33537367	+	#682292	1048383	1006526	516526	109141	516526	1564909	false
	CPOR 27	23899039	24251439	33701009	33944990	+	#682292	352400	243981	109141	-55234	109141	461541	false
	CPOR 27	24196205	24633202	33950061	34322507	+	#682292	436997	372446	-55234	-58063	-55234	381763	false
	CPOR 27	24575139	24636404	34322074	34410376	+	#682292	61265	88302	-58063		-58063	3202	false
	CPOR 1	24624505	27002809	7402142	9836406	+	#C18B50	2378304	2434264		618			
	CPOR 1	27003427	27094932	9844238	9931644	-	#C18B50	91505	87406	618	5341	618	92123	false
	CPOR 1	27100273	33858751	9941942	16097042	+	#C18B50	6758478	6155100	5341	816	5341	6763819	false
	CPOR 1	33859567	38186011	16163023	20195893	+	#C18B50	4326444	4032870	816	1478	816	4327260	false
	CPOR 1	38187489	39577803	20298933	21420616	+	#C18B50	1390314	1121683	1478	0	1478	1391792	false
	CPOR 1	39577803	42619694	21476828	24087839	+	#C18B50	3041891	2611011	0	2525	0	3041891	false
	CPOR 1	42622219	45710666	24159078	27018675	+	#C18B50	3088447	2859597	2525	-5525	2525	3090972	false
	CPOR 1	45705141	55209328	27062754	35112351	+	#C18B50	9504187	8049597	-5525		-5525	9498662	false
	CPOR 29	55269884	55442914	29255	177476	+	#FF7F00	173030	148221		108188			
	CPOR 29	55551102	55688960	196678	349496	-	#FF7F00	137858	152818	108188	-28555	108188	246046	false
	CPOR 29	55660405	59999237	396271	4662376	-	#FF7F00	4338832	4266105	-28555	-135466	-28555	4310277	false
	CPOR 29	59863771	60175904	4662805	4863204	+	#FF7F00	312133	200399	-135466	7605	-135466	176667	false
	CPOR 29	60183509	63099043	5023421	8000858	+	#FF7F00	2915534	2977437	7605	2	7605	2923139	false
	CPOR 29	63099045	63803000	8099871	8806654	+	#FF7F00	703955	706783	2	35815	2	703957	false
	CPOR 29	63838815	66692778	8868790	11517537	+	#FF7F00	2853963	2648747	35815	61484	35815	2889778	false
	CPOR 29	66754262	70423657	11533705	14942235	+	#FF7F00	3669395	3408530	61484	157514	61484	3730879	false
	CPOR 29	70581171	73595844	15006517	17820151	+	#FF7F00	3014673	2813634	157514	-54411	157514	3172187	false
	CPOR 29	73541433	76685048	17854163	20579227	+	#FF7F00	3143615	2725064	-54411	8109	-54411	3089204	false
	CPOR 29	76693157	92021852	20638251	34447364	+	#FF7F00	15328695	13809113	8109	-59123	8109	15336804	false

	CPOR 29	91962729	94383343	34586360	36712463	-	#FF7F00	2420614	2126103	-59123	-19878	-59123	2361491	false
	CPOR 29	94363465	94524701	38074784	38273787	-	#FF7F00	161236	199003	-19878	50231	-19878	141358	false
	CPOR 29	94574932	96267764	36803537	38068214	-	#FF7F00	1692832	1264677	50231	-1384	50231	1743063	false
	CPOR 29	96266380	100642507	38280166	41768844	+	#FF7F00	4376127	3488678	-1384	439	-1384	4374743	false
	CPOR 29	100642946	101079411	42244402	42634519	+	#FF7F00	436465	390117	439	3000	439	436904	false
	CPOR 29	101082411	103157355	42972232	44417142	+	#FF7F00	2074944	1444910	3000	166008	3000	2077944	false
	CPOR 29	103323363	104449762	44442849	45181631	+	#FF7F00	1126399	738782	166008	57283	166008	1292407	false
	CPOR 29	104507045	105531522	45217683	45851339	+	#FF7F00	1024477	633656	57283		57283	1081760	false
	CPOR 8	105703723	105776869	72639254	72691814	+	#60FA36	73146	52560		42244			
	CPOR 8	105819113	105924710	72843934	72938790	+	#60FA36	105597	94856	42244		42244	147841	false
**HSA 15**	CPOR 4	20430774	20507549	1972484	2088601	-	#C008AD	76775	116117		1096507			
	CPOR 4	21604056	21685093	71293876	71347716	+	#C008AD	81037	53840	1096507	354316	1096507	1177544	false
	CPOR 4	22039409	22127563	71293876	71347716	+	#C008AD	88154	53840	354316	365938	354316	442470	false
	CPOR 4	22493501	22575114	1972484	2091024	+	#C008AD	81613	118540	365938	210063	365938	447551	false
	CPOR 4	22785177	23042641	1713201	1972353	-	#C008AD	257464	259152	210063	510060	210063	467527	false
	CPOR 4	23552701	23875360	5353541	5578962	-	#C008AD	322659	225421	510060	513621	510060	832719	false
	CPOR 4	24388981	24493230	5287057	5350699	-	#C008AD	104249	63642	513621	354577	513621	617870	false
	CPOR 4	24847807	25167128	4449186	4724361	-	#C008AD	319321	275175	354577	97540	354577	673898	false
	CPOR 4	25264668	28323528	1972484	4457033	-	#C008AD	3058860	2484549	97540	264566	97540	3156400	false
	CPOR 4	28588094	28669396	1972484	2091024	+	#C008AD	81302	118540	264566	191609	264566	345868	false
	CPOR 4	28861005	30076423	6941688	8080960	+	#C008AD	1215418	1139272	191609	200806	191609	1407027	false
	CPOR 4	30277229	30376921	5765231	5843574	+	#C008AD	99692	78343	200806	318301	200806	300498	false
	CPOR 4	30695222	30775192	6183895	6260200	+	#C008AD	79970	76305	318301	124231	318301	398271	false
	CPOR 4	30899423	32261428	5765231	6877345	-	#C008AD	1362005	1112114	124231	347844	124231	1486236	false
	CPOR 4	32609272	34374869	35247159	36961217	+	#C008AD	1765597	1714058	347844	265549	347844	2113441	false
	CPOR 4	34640418	43657409	26663709	35193666	-	#C008AD	9016991	8529957	265549	1082	265549	9282540	false
	CPOR 1	43623446	43718537	16097056	16156908	+	#C18B50	95091	59852		1237439			
	CPOR 4	43658491	44748401	25680698	26775911	-	#C008AD	1089910	1095213	1082	34104784	1082	1090992	false
	CPOR 1	44955976	45808774	35144936	35696068	+	#C18B50	852798	551132	1237439	-117160	1237439	2090237	false
	CPOR 1	45691614	46953424	36512563	37350165	+	#C18B50	1261810	837602	-117160	-1249870	-117160	1144650	false
	CPOR 1	45703554	47180292	37402465	38480730	+	#C18B50	1476738	1078265	-1249870	-1288660	-1249870	226868	false
	CPOR 1	45891632	46150678	35696068	35874939	+	#C18B50	259046	178871	-1288660	58755	-1288660	-1029614	true

CPOR 1	46209433	46934118	35875466	36380751	+	#C18B50	724685	505285	58755	-724685	58755	783440	false
CPOR 1	46209433	47180292	39587335	40414635	-	#C18B50	970859	827300	-724685	-237392	-724685	246174	false
CPOR 1	46942900	54713540	40445721	46246095	+	#C18B50	7770640	5800374	-237392	61545	-237392	7533248	false
CPOR 1	54775085	55104102	46246710	46437393	+	#C18B50	329017	190683	61545	60994	61545	390562	false
CPOR 1	55165096	56254727	46441217	47077202	+	#C18B50	1089631	635985	60994		60994	1150625	false
CPOR 8	56357668	58684334	88889117	90716551	-	#60FA36	2326666	1827434	35928746	-444	35928746	38255412	false
CPOR 8	58683890	61981067	98964964	101761934	-	#60FA36	3297177	2796970	-444	-66795	-444	3296733	false
CPOR 8	61914272	62073569	98093462	98221928	+	#60FA36	159297	128466	-66795	-127733	-66795	92502	false
CPOR 8	61945836	62233484	95788059	96004299	-	#60FA36	287648	216240	-127733	71253	-127733	159915	false
CPOR 8	62304737	63466363	94773437	95788055	-	#60FA36	1161626	1014618	71253	286803	71253	1232879	false
CPOR 8	63753166	63823906	103782109	103862045	-	#60FA36	70740	79936	286803	1134383	286803	357543	false
CPOR 12	63900391	64931589	6388664	7252038	+	#00007F	1031198	863374		1640862			
CPOR 8	64958289	65037359	90785431	90906168	+	#60FA36	79070	120737	1134383	593	1134383	1213453	false
CPOR 8	65037952	65145938	85170486	85234493	+	#60FA36	107986	64007	593	2038	593	108579	false
CPOR 8	65147976	66572101	101984750	103118061	+	#60FA36	1424125	1133311	2038	6151449	2038	1426163	false
CPOR 12	66572451	69292682	9489	2017955	-	#00007F	2720231	2008466	1640862		1640862	4361093	false
CPOR 3	69293230	72378933	585813	3015569	-	#13C840	3085703	2429756	5406699	75525	5406699	8492402	false
CPOR 3	72454458	72600528	481898	585662	-	#13C840	146070	103764	75525	27215023	75525	221595	false
CPOR 8	72723550	74051581	96251203	97251978	+	#60FA36	1328031	1000775	6151449	68807	6151449	7479480	false
CPOR 8	74120388	74343079	97251978	97412952	+	#60FA36	222691	160974	68807	-24259	68807	291498	false
CPOR 8	74318820	75051177	91417833	91971949	+	#60FA36	732357	554116	-24259	100502	-24259	708098	false
CPOR 8	75151679	75251551	92008429	92090918	+	#60FA36	99872	82489	100502	84405	100502	200374	false
CPOR 8	75335956	75736397	92053132	92325796	+	#60FA36	400441	272664	84405	61682	84405	484846	false
CPOR 8	75798079	77895321	92292314	93960809	-	#60FA36	2097242	1668495	61682	72481	61682	2158924	false
CPOR 8	77967802	78713705	93952347	94482573	-	#60FA36	745903	530226	72481	44580	72481	818384	false
CPOR 8	78758285	78828929	94462238	94517885	+	#60FA36	70644	55647	44580	5694775	44580	115224	false
CPOR 4	78853185	79907354	721750	1488745	+	#C008AD	1054169	766995	34104784	44665	34104784	35158953	false
CPOR 4	79952019	80324135	1473249	1707309	+	#C008AD	372116	234060	44665	2740	44665	416781	false
CPOR 4	80326875	82277023	24043502	25641192	-	#C008AD	1950148	1597690	2740	259544	2740	1952888	false
CPOR 4	82536567	84150119	22761291	24144813	+	#C008AD	1613552	1383522	259544	448115	259544	1873096	false
CPOR 4	84598234	85156765	22300930	22758920	+	#C008AD	558531	457990	448115	129658	448115	1006646	false
CPOR 4	85286423	90273575	17406999	21752078	+	#C008AD	4987152	4345079	129658	41611	129658	5116810	false

	CPOR 4	90315186	91025213	21752932	22300897	-	#C008AD	710027	547965	41611	23344	41611	751638	false
	CPOR 4	91048557	92721951	15708457	17415655	-	#C008AD	1673394	1707198	23344	58753	23344	1696738	false
	CPOR 4	92780704	98536051	10664788	15692502	-	#C008AD	5755347	5027714	58753	1094	58753	5814100	false
	CPOR 4	98537145	99023290	10259915	10664788	+	#C008AD	486145	404873	1094	943	1094	487239	false
	CPOR 4	99024233	99754865	9654331	10259904	-	#C008AD	730632	605573	943	166493	943	731575	false
	CPOR 4	99921358	101732197	8080960	9649804	-	#C008AD	1810839	1568844	166493	74469	166493	1977332	false
	CPOR 4	101806666	101950224	71730078	71888908	-	#C008AD	143558	158830	74469		74469	218027	false
**HSA 16**	CPOR 17	28451	1289092	40261031	41444288	-	#FAB28C	1260641	1183257		-29790			
	CPOR 17	1259302	2543394	38903653	40164821	-	#FAB28C	1284092	1261168	-29790	190680	-29790	1254302	false
	CPOR 17	2734074	2871351	38591961	38784598	+	#FAB28C	137277	192637	190680	11376	190680	327957	false
	CPOR 17	2882727	10277096	32050434	38586069	-	#FAB28C	7394369	6535635	11376	108518	11376	7405745	false
	CPOR 17	10385614	14362060	28340007	31896658	-	#FAB28C	3976446	3556651	108518	49823	108518	4084964	false
	CPOR 17	14411883	14733677	28059676	28278233	-	#FAB28C	321794	218557	49823	103634	49823	371617	false
	CPOR 17	14837311	14910034	24575354	24637042	+	#FAB28C	72723	61688	103634	44770	103634	176357	false
	CPOR 17	14954804	15100600	23815180	23938040	-	#FAB28C	145796	122860	44770	133528	44770	190566	false
	CPOR 17	15234128	15345692	24647422	24736356	-	#FAB28C	111564	88934	133528	49519	133528	245092	false
	CPOR 17	15395211	16308689	23938069	24637042	+	#FAB28C	913478	698973	49519	107860	49519	962997	false
	CPOR 17	16416549	16570806	24653877	24811967	+	#FAB28C	154257	158090	107860	192492	107860	262117	false
	CPOR 17	16763298	18078901	24869921	26110821	-	#FAB28C	1315603	1240900	192492	62812	192492	1508095	false
	CPOR 17	18141713	18295330	24653877	24811967	-	#FAB28C	153617	158090	62812	188684	62812	216429	false
	CPOR 17	18484014	18558886	24575354	24639675	-	#FAB28C	74872	64321	188684	224089	188684	263556	false
	CPOR 17	18782975	18865664	26310067	26377198	+	#FAB28C	82689	67131	224089	116700	224089	306778	false
	CPOR 17	18982364	19381928	26526418	26862965	-	#FAB28C	399564	336547	116700	3191	116700	516264	false
	CPOR 17	19385119	20445089	26879324	27830675	+	#FAB28C	1059970	951351	3191	171169	3191	1063161	false
	CPOR 17	20616258	20705082	27968346	28050532	-	#FAB28C	88824	82186	171169	4343	171169	259993	false
	CPOR 17	20709425	21340339	23177330	23732823	-	#FAB28C	630914	555493	4343	218538	4343	635257	false
	CPOR 17	21558877	21796206	22436625	22647951	-	#FAB28C	237329	211326	218538	140956	218538	455867	false
	CPOR 17	21937162	22419424	22647992	23097802	-	#FAB28C	482262	449810	140956	142388	140956	623218	false
	CPOR 17	22561812	25060377	20056792	22482386	-	#FAB28C	2498565	2425594	142388	26952	142388	2640953	false
	CPOR 17	25087329	28323949	17005146	20115042	-	#FAB28C	3236620	3109896	26952	153224	26952	3263572	false
	CPOR 17	28477173	28610355	16627828	16723988	-	#FAB28C	133182	96160	153224	209155	153224	286406	false
	CPOR 17	28819510	29032722	16780010	16931599	+	#FAB28C	213212	151589	209155	610697	209155	422367	false

	CPOR 17	29643419	30210361	16162170	16628933	+	#FAB28C	566942	466763	610697	137979	610697	1177639	false
	CPOR 17	30348340	31531343	15146175	16156088	-	#FAB28C	1183003	1009913	137979		137979	1320982	false
	CPOR 6	46581690	48615880	5302785	7089514	-	#0C404C	2034190	1786729					
	CPOR 3	48639697	53328031	50480909	54860603	-	#13C840	4688334	4379694	19366452	99128	19366452	24054786	false
	CPOR 3	53427159	55718936	48349662	50472752	-	#13C840	2291777	2123090	99128	272	99128	2390905	false
	CPOR 3	55719208	55795318	74471285	74525210	-	#13C840	76110	53925	272	-69625	272	76382	false
	CPOR 3	55725693	55782672	74918088	74995145	-	#13C840	56979	77057	-69625	19404	-69625	-12646	true
	CPOR 3	55802076	64578961	74973284	82978357	+	#13C840	8776885	8005073	19404	-2648324	19404	8796289	false
	CPOR 3	61930637	65014593	83588957	86308802	-	#13C840	3083956	2719845	-2648324	-2096892	-2648324	435632	false
	CPOR 3	62917701	66958550	87428653	91318312	+	#13C840	4040849	3889659	-2096892	-24021	-2096892	1943957	false
	CPOR 3	66934529	68013492	91480576	92459848	+	#13C840	1078963	979272	-24021	-8134	-24021	1054942	false
	CPOR 3	68005358	69943578	92466315	94299623	+	#13C840	1938220	1833308	-8134	163565	-8134	1930086	false
	CPOR 3	70107143	70189637	98131739	98249301	-	#13C840	82494	117562	163565	61344	163565	246059	false
	CPOR 3	70250981	70819448	97635985	98119931	-	#13C840	568467	483946	61344	77464	61344	629811	false
	CPOR 3	70896912	74328227	94299675	97604268	-	#13C840	3431315	3304593	77464	106365	77464	3508779	false
	CPOR 3	74434592	75226343	98200234	98899116	+	#13C840	791751	698882	106365	-24426	106365	898116	false
	CPOR 3	75201917	75884693	99027900	99583291	+	#13C840	682776	555391	-24426	466	-24426	658350	false
	CPOR 3	75885159	76575402	99647304	100349082	+	#13C840	690243	701778	466	-28515	466	690709	false
	CPOR 3	76546887	77192066	101373644	101952928	-	#13C840	645179	579284	-28515	-645179	-28515	616664	false
	CPOR 3	76546887	77194090	100351698	100933917	-	#13C840	647203	582219	-645179	-643634	-645179	2024	false
	CPOR 3	76550456	77194090	101952929	102539800	-	#13C840	643634	586871	-643634	-221479	-643634	0	true
	CPOR 3	76972611	81964749	102944139	107078604	+	#13C840	4992138	4134465	-221479	-84270	-221479	4770659	false
	CPOR 3	81880479	84330325	107193417	109249508	-	#13C840	2449846	2056091	-84270	24027	-84270	2365576	false
	CPOR 3	84354352	87770398	109254123	110987516	-	#13C840	3416046	1733393	24027	233	24027	3440073	false
	CPOR 3	87770631	88357389	111022426	111264942	+	#13C840	586758	242516	233	51323	233	586991	false
	CPOR 3	88408712	88977072	111282339	111604422	+	#13C840	568360	322083	51323	68840	51323	619683	false
	CPOR 3	89045912	89203087	111604825	111678752	+	#13C840	157175	73927	68840	71284	68840	226015	false
	CPOR 3	89274371	90051543	111702711	112335960	+	#13C840	777172	633249	71284		71284	848456	false
**HSA 17**	CPOR 4	142967	1334471	76766601	77760505	+	#C008AD	1191504	993904		3496			
	CPOR 4	1337967	3039912	75245501	76747477	-	#C008AD	1701945	1501976	3496	106963	3496	1705441	false
	CPOR 4	3146875	3242603	74918740	75024101	+	#C008AD	95728	105361	106963	64525	106963	202691	false
	CPOR 4	3307128	3438711	74701725	74757191	-	#C008AD	131583	55466	64525	-41563	64525	196108	false

	CPOR 4	3397148	4628870	73315349	74351333	-	#C008AD	1231722	1035984	-41563	885279	-41563	1190159	false
	CPOR 21	4631636	5115377	9736943	10162506	+	#0000FF	483741	425563		64191			
	CPOR 21	5179568	5510359	10166924	10540324	+	#0000FF	330791	373400	64191	1361376	64191	394982	false
	CPOR 4	5514149	6845307	72057731	73319050	+	#C008AD	1331158	1261319	885279	13866211	885279	2216437	false
	CPOR 21	6871735	8194777	8487966	9733726	-	#0000FF	1323042	1245760	1361376	84	1361376	2684418	false
	CPOR 21	8194861	9631383	7238521	8445739	+	#0000FF	1436522	1207218	84	-74019	84	1436606	false
	CPOR 21	9557364	10733562	6219430	7236023	+	#0000FF	1176198	1016593	-74019	-61221	-74019	1102179	false
	CPOR 21	10672341	15708963	1569524	6198791	-	#0000FF	5036622	4629267	-61221	117124	-61221	4975401	false
	CPOR 21	15826087	16654105	784662	1548981	+	#0000FF	828018	764319	117124	198345	117124	945142	false
	CPOR 24	16701463	16771441	46477434	46552275	+	#00FFFF	69978	74841		1772809			
	CPOR 21	16852450	16913561	22072674	22159461	-	#0000FF	61111	86787	198345	5616	198345	259456	false
	CPOR 21	16919177	18364610	22010203	23331288	+	#0000FF	1445433	1321085	5616	327337	5616	1451049	false
	CPOR 24	18544250	18613974	46477434	46552275	-	#00FFFF	69724	74841	1772809	1718823	1772809	1842533	false
	CPOR 21	18691947	18823588	1580850	1730806	+	#0000FF	131641	149956	327337	33059	327337	458978	false
	CPOR 21	18856647	19072871	31057388	31242615	-	#0000FF	216224	185227	33059	121276	33059	249283	false
	CPOR 21	19194147	19755354	30648128	31068370	-	#0000FF	561207	420242	121276	13109	121276	682483	false
	CPOR 21	19768463	20319402	375322	881789	+	#0000FF	550939	506467	13109	648032	13109	564048	false
	CPOR 24	20332797	20399985	46477434	46552275	+	#00FFFF	67188	74841	1718823		1718823	1786011	false
	CPOR 21	20967434	21046646	21989652	22078289	-	#0000FF	79212	88637	648032	52435	648032	727244	false
	CPOR 21	21099081	21466836	21619340	21941799	-	#0000FF	367755	322459	52435	24165934	52435	420190	false
	CPOR 4	27197291	27650843	80629768	81047963	-	#C008AD	453552	418195	6366504	90523	6366504	6820056	false
	CPOR 4	27741366	28197156	79844416	80269824	-	#C008AD	455790	425408	90523	115154	90523	546313	false
	CPOR 4	28312310	30343066	78013591	79844416	-	#C008AD	2030756	1830825	115154	269	115154	2145910	false
	CPOR 4	30343335	30529492	77710611	77894631	-	#C008AD	186157	184020	269	201736	269	186426	false
	CPOR 4	30731228	31005961	70262379	70498945	-	#C008AD	274733	236566	201736	45008	201736	476469	false
	CPOR 4	31050969	31385750	81016878	81316486	+	#C008AD	334781	299608	45008	-29901	45008	379789	false
	CPOR 4	31355849	32001249	70449340	70960076	-	#C008AD	645400	510736	-29901	132605	-29901	615499	false
	CPOR 4	32133854	35273799	67480658	70262177	-	#C008AD	3139945	2781519	132605	76792	132605	3272550	false
	CPOR 4	35350591	35444919	67340190	67432426	-	#C008AD	94328	92236	76792	85635	76792	171120	false
	CPOR 4	35530554	35901807	67071929	67356717	-	#C008AD	371253	284788	85635	67025	85635	456888	false
	CPOR 4	35968832	36076332	66952427	67058856	-	#C008AD	107500	106429	67025	-20045	67025	174525	false
	CPOR 4	36056287	36158055	66513262	66579735	-	#C008AD	101768	66473	-20045	28818	-20045	81723	false

CPOR 4	36186873	36245689	66513262	66584019	-	#C008AD	58816	70757	28818	211306	28818	87634	false
CPOR 4	36456995	37843052	65225534	66455148	+	#C008AD	1386057	1229614	211306	402315	211306	1597363	false
CPOR 4	38245367	39001042	90824577	91285777	+	#C008AD	755675	461200	402315	60532	402315	1157990	false
CPOR 4	39061574	41179372	91285907	92767507	+	#C008AD	2117798	1481600	60532	-21777	60532	2178330	false
CPOR 4	41157595	42924900	92815417	93857039	+	#C008AD	1767305	1041622	-21777	25641	-21777	1745528	false
CPOR 4	42950541	43228883	94083347	94205969	+	#C008AD	278342	122622	25641	248238	25641	303983	false
CPOR 4	43477121	44564169	94222366	94758804	+	#C008AD	1087048	536438	248238	82246	248238	1335286	false
CPOR 4	44646415	45499992	94783567	95382766	+	#C008AD	853577	599199	82246	1980750	82246	935823	false
CPOR 21	45632770	46241243	23734703	24270464	+	#0000FF	608473	535761	24165934	5074	24165934	24774407	false
CPOR 21	46246317	46343415	13067390	13138699	+	#0000FF	97098	71309	5074	46008	5074	102172	false
CPOR 21	46389423	46489133	23607180	23716824	-	#0000FF	99710	109644	46008	117911	46008	145718	false
CPOR 21	46607044	47003557	23349387	23716824	-	#0000FF	396513	367437	117911	86840	117911	514424	false
CPOR 21	47090397	47335354	24222399	24485602	+	#0000FF	244957	263203	86840	15031320	86840	331797	false
CPOR 4	47480742	47542756	90824577	90880793	-	#C008AD	62014	56216	1980750	53524	1980750	2042764	false
CPOR 4	47596280	47757543	90668501	90788378	-	#C008AD	161263	119877	53524	4648	53524	214787	false
CPOR 4	47762191	49999457	89102951	90608116	-	#C008AD	2237266	1505165	4648	20976	4648	2241914	false
CPOR 4	50020433	56869725	83522566	88960868	-	#C008AD	6849292	5438302	20976	-7663	20976	6870268	false
CPOR 4	56862062	58608223	81646132	83221027	-	#C008AD	1746161	1574895	-7663	-58524	-7663	1738498	false
CPOR 4	58549699	59967259	62285036	63489998	+	#C008AD	1417560	1204962	-58524	66003	-58524	1359036	false
CPOR 4	60033262	60109361	66455373	66544173	+	#C008AD	76099	88800	66003	29302	66003	142102	false
CPOR 4	60138663	62228764	63518768	65225253	-	#C008AD	2090101	1706485	29302	2542359	29302	2119403	false
CPOR 21	62366674	64758892	24791961	27102203	+	#0000FF	2392218	2310242	15031320	210530	15031320	17423538	false
CPOR 4	64771123	64846418	95283163	95382766	+	#C008AD	75295	99603	2542359		2542359	2617654	false
CPOR 21	64969422	68113639	26990400	29819576	-	#0000FF	3144217	2829176	210530	117997	210530	3354747	false
CPOR 21	68231636	69146693	29801547	30508655	+	#0000FF	915057	707108	117997	3825744	117997	1033054	false
CPOR 18	69240889	73209171	78351017	81963280	+	#80E0F6	3968282	3612263					
CPOR 21	72972437	73034645	24608503	24659422	+	#0000FF	62208	50919	3825744	34984	3825744	3887952	false
CPOR 21	73069629	73209171	15222304	15336781	-	#0000FF	139542	114477	34984	-16212	34984	174526	false
CPOR 21	73192959	75209608	13332002	15148939	-	#0000FF	2016649	1816937	-16212	-45415	-16212	2000437	false
CPOR 21	75164193	75217534	13171878	13248726	+	#0000FF	53341	76848	-45415	-12379	-45415	7926	false
CPOR 21	75205155	75579105	11601010	11933348	-	#0000FF	373950	332338	-12379	36	-12379	361571	false
CPOR 21	75579141	76594775	12110673	13022666	-	#0000FF	1015634	911993	36	28072	36	1015670	false

	CPOR 21	76622847	80120974	15452415	18139478	+	#0000FF	3498127	2687063	28072	213	28072	3526199	false
	CPOR 21	80121187	81735207	18206139	19549857	+	#0000FF	1614020	1343718	213	65685	213	1614233	false
	CPOR 21	81800892	82354930	19549873	20097668	+	#0000FF	554038	547795	65685	254	65685	619723	false
	CPOR 21	82355184	83231424	20160883	20889258	+	#0000FF	876240	728375	254		254	876494	false
**HSA 18**	CPOR 23	112548	1147557	8724465	9595017	-	#0C8017	1035009	870552		11400			
	CPOR 23	1158957	1360777	8538919	8724465	+	#0C8017	201820	185546	11400	3985	11400	213220	false
	CPOR 23	1364762	10555928	1689785	8535568	-	#0C8017	9191166	6845783	3985	107544	3985	9195151	false
	CPOR 23	10663472	11562202	1199572	1689407	-	#0C8017	898730	489835	107544	116481	107544	1006274	false
	CPOR 23	11678683	11911428	1059106	1199509	-	#0C8017	232745	140403	116481	335699	116481	349226	false
	CPOR 23	12247127	12727399	778894	1011721	-	#0C8017	480272	232827	335699	57274	335699	815971	false
	CPOR 23	12784673	13374070	423368	777293	-	#0C8017	589397	353925	57274	190887	57274	646671	false
	CPOR 23	13564957	13834038	241827	413243	-	#0C8017	269081	171416	190887	7113700	190887	459968	false
	CPOR 8	14182732	14241276	96155091	96229903	+	#60FA36	58544	74812		512070			
	CPOR 24	14182853	14239580	46464945	46541231	+	#00FFFF	56727	76286		513766			
	CPOR 8	14753346	14839896	97964986	98071953	-	#60FA36	86550	106967	512070		512070	598620	false
	CPOR 24	14753346	14854208	46467129	46529316	+	#00FFFF	100862	62187	513766		513766	614628	false
	CPOR 23	20947738	29579589	9582467	16859023	+	#0C8017	8631851	7276556	7113700	17464	7113700	15745551	false
	CPOR 23	29597053	29931017	16914263	17203913	+	#0C8017	333964	289650	17464	2670	17464	351428	false
	CPOR 23	29933687	30070950	17210506	17343395	-	#0C8017	137263	132889	2670	14807	2670	139933	false
	CPOR 23	30085757	31742879	17349862	18786827	+	#0C8017	1657122	1436965	14807	-49838	14807	1671929	false
	CPOR 23	31693041	31785860	18882047	18950054	-	#0C8017	92819	68007	-49838	10194	-49838	42981	false
	CPOR 23	31796054	38137873	18988182	24428581	+	#0C8017	6341819	5440399	10194	40184	10194	6352013	false
	CPOR 23	38178057	44327037	24479810	30261927	+	#0C8017	6148980	5782117	40184	54360	40184	6189164	false
	CPOR 23	44381397	46959525	30266581	32621834	+	#0C8017	2578128	2355253	54360	77217	54360	2632488	false
	CPOR 23	47036742	49494341	32629735	34830365	+	#0C8017	2457599	2200630	77217	16419	77217	2534816	false
	CPOR 23	49510760	63665165	34904352	49110455	+	#0C8017	14154405	14206103	16419	-28402	16419	14170824	false
	CPOR 23	63636763	64082580	49178662	49589676	+	#0C8017	445817	411014	-28402	-112202	-28402	417415	false
	CPOR 23	63970378	64084877	49589935	49660937	-	#0C8017	114499	71002	-112202	-647	-112202	2297	false
	CPOR 23	64084230	64479976	52038441	52414177	+	#0C8017	395746	375736	-647	-175228	-647	395099	false
	CPOR 23	64304748	65260504	50891481	51768839	-	#0C8017	955756	877358	-175228	-344119	-175228	780528	false
	CPOR 23	64916385	65651963	49679099	50499899	-	#0C8017	735578	820800	-344119	-288421	-344119	391459	false
	CPOR 23	65363542	68075566	52421272	55222958	+	#0C8017	2712024	2801686	-288421	23627	-288421	2423603	false

	CPOR 23	68099193	71233344	55282978	58635496	+	#0C8017	3134151	3352518	23627	1119	23627	3157778	false
	CPOR 23	71234463	71292438	58651724	58734530	-	#0C8017	57975	82806	1119	4342	1119	59094	false
	CPOR 23	71296780	77171495	58735047	64735606	+	#0C8017	5874715	6000559	4342	56751	4342	5879057	false
	CPOR 23	77228246	77535643	64834474	65177381	+	#0C8017	307397	342907	56751	1421	56751	364148	false
	CPOR 23	77537064	80047540	65311024	67939885	+	#0C8017	2510476	2628861	1421	17396	1421	2511897	false
	CPOR 23	80064936	80254828	68011586	68194102	+	#0C8017	189892	182516	17396		17396	207288	false
**HSA 19**	CPOR 4	82349	162460	37510528	37596007	-	#C008AD	80111	85479		-80111			
	CPOR 4	82349	166725	71730078	71804419	+	#C008AD	84376	74341	-80111		-80111	4265	false
	CPOR 11	474906	2436928	6030340	7172107	-	#6E0025	1962022	1141767		-20693			
	CPOR 11	2416235	2814772	5416407	5587404	+	#6E0025	398537	170997	-20693	160327	-20693	377844	false
	CPOR 11	2975099	3208580	5657261	5777759	-	#6E0025	233481	120498	160327	62440	160327	393808	false
	CPOR 11	3271020	3454947	5596393	5657261	+	#6E0025	183927	60868	62440	47592	62440	246367	false
	CPOR 11	3502539	3956686	5818607	6029486	+	#6E0025	454147	210879	47592	-23	47592	501739	false
	CPOR 11	3956663	4283287	5233716	5424715	-	#6E0025	326624	190999	-23	-44125	-23	326601	false
	CPOR 11	4239162	6566184	4031132	5199883	-	#6E0025	2327022	1168751	-44125	19352	-44125	2282897	false
	CPOR 11	6585536	6929063	3682949	3872051	-	#6E0025	343527	189102	19352	146014	19352	362879	false
	CPOR 11	7075077	7355103	10689800	10889816	+	#6E0025	280026	200016	146014	-16563	146014	426040	false
	CPOR 11	7338540	7748188	10937050	11283495	+	#6E0025	409648	346445	-16563	14911	-16563	393085	false
	CPOR 11	7763099	8733250	12589654	13225261	-	#6E0025	970151	635607	14911	74199	14911	985062	false
	CPOR 11	8807449	8941662	12214313	12334071	-	#6E0025	134213	119758	74199	56000	74199	208412	false
	CPOR 11	8997662	9079923	12132334	12211143	-	#6E0025	82261	78809	56000	298104	56000	138261	false
	CPOR 11	9378027	9538134	406163	465696	+	#6E0025	160107	59533	298104	-123215	298104	458211	false
	CPOR 11	9414919	9537503	522629	677616	+	#6E0025	122584	154987	-123215	-112344	-123215	-631	true
	CPOR 11	9425159	9742162	467526	690967	+	#6E0025	317003	223441	-112344	-127526	-112344	204659	false
	CPOR 11	9614636	9699232	584524	677154	+	#6E0025	84596	92630	-127526	105480	-127526	-42930	true
	CPOR 11	9804712	11460744	2405680	3363562	+	#6E0025	1656032	957882	105480	8119	105480	1761512	false
	CPOR 11	11468863	11561477	3436998	3492678	+	#6E0025	92614	55680	8119	1083588	8119	100733	false
	CPOR 11	12645065	13601276	9256622	9779914	-	#6E0025	956211	523292	1083588	68639	1083588	2039799	false
	CPOR 11	13669915	14189078	8969927	9254847	-	#6E0025	519163	284920	68639	57505	68639	587802	false
	CPOR 11	14246583	14573526	8789573	8969774	-	#6E0025	326943	180201	57505	247489	57505	384448	false
	CPOR 11	14821015	14923565	8070187	8127061	+	#6E0025	102550	56874	247489	7700	247489	350039	false
	CPOR 11	14931265	15684290	7360160	7869002	-	#6E0025	753025	508842	7700	-111648	7700	760725	false

CPOR 11	15572642	15721910	7302459	7365732	-	#6E0025	149268	63273	-111648	-105599	-111648	37620	false
CPOR 11	15616311	15781331	7314511	7401540	+	#6E0025	165020	87029	-105599	63069	-105599	59421	false
CPOR 11	15844400	15997579	7302466	7393599	+	#6E0025	153179	91133	63069	62084	63069	216248	false
CPOR 11	16059663	16173864	26142706	26196587	-	#6E0025	114201	53881	62084	9669	62084	176285	false
CPOR 11	16183533	16893903	25088125	25373851	+	#6E0025	710370	285726	9669	154545	9669	720039	false
CPOR 11	17048448	17734567	25374805	25677619	+	#6E0025	686119	302814	154545	12776	154545	840664	false
CPOR 11	17747343	19551453	27126432	28028633	-	#6E0025	1804110	902201	12776		12776	1816886	false
CPOR 24	27935729	27986739	40019672	40089739	-	#00FFFF	51010	70067		-51010			
CPOR 24	27935729	27990024	48942143	49037864	-	#00FFFF	54295	95721	-51010		-51010	3285	false
CPOR 7	27999787	28052990	52539223	52596863	+	#2DCDC1	53203	57640		45109			
CPOR 7	28098099	28737000	51755272	52299725	-	#2DCDC1	638901	544453	45109	81818	45109	684010	false
CPOR 7	28818818	32933682	52423666	56052092	+	#2DCDC1	4114864	3628426	81818	-106859	81818	4196682	false
CPOR 7	32826823	33125842	56122110	56324951	+	#2DCDC1	299019	202841	-106859	57562	-106859	192160	false
CPOR 7	33183404	34506075	79102861	80370108	+	#2DCDC1	1322671	1267247	57562	227236	57562	1380233	false
CPOR 7	34733311	34786315	57056041	57114946	-	#2DCDC1	53004	58905	227236	127471	227236	280240	false
CPOR 7	34913786	35407425	56602321	57006274	-	#2DCDC1	493639	403953	127471	-59752	127471	621110	false
CPOR 7	35347673	35645873	56333275	56596702	-	#2DCDC1	298200	263427	-59752	94	-59752	238448	false
CPOR 7	35645967	36240348	78526678	79059721	-	#2DCDC1	594381	533043	94	97374	94	594475	false
CPOR 7	36337722	36531492	78206887	78306587	+	#2DCDC1	193770	99700	97374	12834	97374	291144	false
CPOR 7	36544326	36769453	78401821	78526648	+	#2DCDC1	225127	124827	12834	118099	12834	237961	false
CPOR 7	36887552	37005910	78093576	78186488	+	#2DCDC1	118358	92912	118099	67477	118099	236457	false
CPOR 7	37073387	37268119	77762162	77856234	+	#2DCDC1	194732	94072	67477	64591	67477	262209	false
CPOR 7	37332710	37552292	77853461	78036931	+	#2DCDC1	219582	183470	64591	41434	64591	284173	false
CPOR 7	37593726	37695884	77609348	77691907	-	#2DCDC1	102158	82559	41434	128203	41434	143592	false
CPOR 7	37824087	37918966	78310975	78398148	+	#2DCDC1	94879	87173	128203	-94761	128203	223082	false
CPOR 7	37824205	39569689	76084638	77458072	-	#2DCDC1	1745484	1373434	-94761	224068	-94761	1650723	false
CPOR 7	39793757	40813936	75088404	75786895	-	#2DCDC1	1020179	698491	224068	106179	224068	1244247	false
CPOR 7	40920115	41019959	74228791	74345434	+	#2DCDC1	99844	116643	106179	-87115	106179	206023	false
CPOR 7	40932844	41019959	74345462	74464122	+	#2DCDC1	87115	118660	-87115	3094	-87115	0	true
CPOR 7	41023053	41074142	74606597	74694279	-	#2DCDC1	51089	87682	3094	64032	3094	54183	false
CPOR 7	41138174	41449754	73945300	74212895	-	#2DCDC1	311580	267595	64032	134116	64032	375612	false
CPOR 7	41583870	41690117	73862131	73942200	-	#2DCDC1	106247	80069	134116	-8465	134116	240363	false

CPOR 7	41681652	41809113	73461009	73652275	+	#2DCDC1	127461	191266	-8465	-126359	-8465	118996	false
CPOR 7	41682754	41813628	73584589	73644567	-	#2DCDC1	130874	59978	-126359	-114315	-126359	4515	false
CPOR 7	41699313	42632261	72619190	73288288	+	#2DCDC1	932948	669098	-114315	-924672	-114315	818633	false
CPOR 7	41707589	41808048	73318987	73385865	-	#2DCDC1	100459	66878	-924672	-100459	-924672	-824213	true
CPOR 7	41707589	41809113	73455475	73535629	-	#2DCDC1	101524	80154	-100459	-97032	-100459	1065	false
CPOR 7	41712081	41798839	73709510	73759726	+	#2DCDC1	86758	50216	-97032	-83295	-97032	-10274	true
CPOR 7	41715544	41813970	72642756	72717429	+	#2DCDC1	98426	74673	-83295	690016	-83295	15131	false
CPOR 7	42503986	42632225	73358884	73441927	+	#2DCDC1	128239	83043	690016	-128239	690016	818255	false
CPOR 7	42503986	42632261	73449459	73533551	+	#2DCDC1	128275	84092	-128239	-128256	-128239	36	false
CPOR 7	42504005	42656985	73660991	73721416	-	#2DCDC1	152980	60425	-128256	-152980	-128256	24724	false
CPOR 7	42504005	42662788	73589942	73659259	-	#2DCDC1	158783	69317	-152980	-142858	-152980	5803	false
CPOR 7	42519930	42662788	73529026	73644567	+	#2DCDC1	142858	115541	-142858	47255	-142858	0	true
CPOR 7	42710043	42788485	73227870	73322774	+	#2DCDC1	78442	94904	47255	-59218	47255	125697	false
CPOR 7	42729267	42788485	73319150	73407652	+	#2DCDC1	59218	88502	-59218	42880	-59218	0	true
CPOR 7	42831365	42952277	73227870	73322774	+	#2DCDC1	120912	94904	42880	-107774	42880	163792	false
CPOR 7	42844503	42952214	73283607	73341465	+	#2DCDC1	107711	57858	-107774	-107711	-107774	-63	true
CPOR 7	42844503	42952277	73327006	73407652	+	#2DCDC1	107774	80646	-107711	-103443	-107711	63	false
CPOR 7	42848834	42952272	73531554	73594072	+	#2DCDC1	103438	62518	-103443	-102014	-103443	-5	true
CPOR 7	42850258	42952277	73458072	73527520	+	#2DCDC1	102019	69448	-102014	-101282	-102014	5	false
CPOR 7	42850995	42952214	73494789	73594013	+	#2DCDC1	101219	99224	-101282	-101219	-101282	-63	true
CPOR 7	42850995	42952232	73491004	73562999	+	#2DCDC1	101237	71995	-101219	-54439	-101219	18	false
CPOR 7	42897793	42952277	73227870	73314710	+	#2DCDC1	54484	86840	-54439	49850	-54439	45	false
CPOR 7	43002127	43099219	73227870	73314710	+	#2DCDC1	97092	86840	49850	-83959	49850	146942	false
CPOR 7	43015260	43099219	73327006	73407652	+	#2DCDC1	83959	80646	-83959	-78215	-83959	0	true
CPOR 7	43021004	43099219	73709510	73771166	-	#2DCDC1	78215	61656	-78215	62905	-78215	0	true
CPOR 7	43162124	43333380	73227870	73385865	+	#2DCDC1	171256	157995	62905	-158021	62905	234161	false
CPOR 7	43175359	43881462	72128818	72671480	-	#2DCDC1	706103	542662	-158021	-700214	-158021	548082	false
CPOR 7	43181248	43333380	73458086	73535629	+	#2DCDC1	152132	77543	-700214	-87053	-700214	-548082	true
CPOR 7	43246327	43300414	73228140	73279243	+	#2DCDC1	54087	51103	-87053	665304	-87053	-32966	true
CPOR 7	43965718	44530128	71942055	72112963	-	#2DCDC1	564410	170908	665304	56416	665304	1229714	false
CPOR 7	44586544	45193511	71508128	71941339	-	#2DCDC1	606967	433211	56416	-8021	56416	663383	false
CPOR 7	45185490	46029963	70727000	71348102	-	#2DCDC1	844473	621102	-8021	233704	-8021	836452	false

	CPOR 7	46263667	47886526	69474364	70621599	-	#2DCDC1	1622859	1147235	233704	104283	233704	1856563	false
	CPOR 7	47990809	48204432	68412910	68637004	-	#2DCDC1	213623	224094	104283	61248	104283	317906	false
	CPOR 7	48265680	50067931	66500573	67793473	-	#2DCDC1	1802251	1292900	61248	75715	61248	1863499	false
	CPOR 7	50143646	51427529	65457558	66499688	-	#2DCDC1	1283883	1042130	75715	-576538	75715	1359598	false
	CPOR 7	50850991	50909529	65987353	66046409	-	#2DCDC1	58538	59056	-576538	544283	-576538	-518000	true
	CPOR 7	51453812	51505217	65701726	65778684	+	#2DCDC1	51405	76958	544283	37003	544283	595688	false
	CPOR 7	51542220	51780444	65192814	65443788	-	#2DCDC1	238224	250974	37003	-29867	37003	275227	false
	CPOR 7	51750577	51818237	65202866	65265237	-	#2DCDC1	67660	62371	-29867	65998	-29867	37793	false
	CPOR 7	51884235	51996896	64887753	64968936	-	#2DCDC1	112661	81183	65998	177898	65998	178659	false
	CPOR 7	52174794	52264395	64295260	64400712	+	#2DCDC1	89601	105452	177898	-23646	177898	267499	false
	CPOR 7	52240749	52369621	64199679	64290872	-	#2DCDC1	128872	91193	-23646	700640	-23646	105226	false
	CPOR 7	53070261	53175910	64158752	64215287	+	#2DCDC1	105649	56535	700640	23867	700640	806289	false
	CPOR 7	53199777	53287112	63239756	63317872	-	#2DCDC1	87335	78116	23867	508704	23867	111202	false
	CPOR 7	53795816	54256588	61779164	62043783	-	#2DCDC1	460772	264619	508704	-40963	508704	969476	false
	CPOR 7	54215625	54313215	61189710	61273283	-	#2DCDC1	97590	83573	-40963	-97590	-40963	56627	false
	CPOR 7	54215625	54340089	61048462	61130405	-	#2DCDC1	124464	81943	-97590	-121069	-97590	26874	false
	CPOR 7	54219020	54337809	59850270	59920802	+	#2DCDC1	118789	70532	-121069	-118789	-121069	-2280	true
	CPOR 7	54219020	54340089	60463767	60536510	+	#2DCDC1	121069	72743	-118789	-121069	-118789	2280	false
	CPOR 7	54219020	54340539	60506897	60625578	+	#2DCDC1	121519	118681	-121069	-118081	-121069	450	false
	CPOR 7	54222458	54340539	61203372	61327571	-	#2DCDC1	118081	124199	-118081	-102056	-118081	0	true
	CPOR 7	54238483	54340539	59916576	59995811	+	#2DCDC1	102056	79235	-102056	-102056	-102056	0	true
	CPOR 7	54238483	54357561	60043613	60105521	+	#2DCDC1	119078	61908	-102056	-90813	-102056	17022	false
	CPOR 7	54266748	54340539	61510419	61561727	-	#2DCDC1	73791	51308	-90813	-45718	-90813	-17022	true
	CPOR 7	54294821	54357410	60246468	60311306	+	#2DCDC1	62589	64838	-45718	-3339	-45718	16871	false
	CPOR 7	54354071	54474628	60777266	60858964	-	#2DCDC1	120557	81698	-3339	95977	-3339	117218	false
	CPOR 7	54570605	54713225	59939864	60071996	-	#2DCDC1	142620	132132	95977	-139902	95977	238597	false
	CPOR 7	54573323	54713506	59827172	59922536	-	#2DCDC1	140183	95364	-139902	-140016	-139902	281	false
	CPOR 7	54573490	54710026	60507667	60616847	-	#2DCDC1	136536	109180	-140016	-136536	-140016	-3480	true
	CPOR 7	54573490	54710506	59851616	59993823	-	#2DCDC1	137016	142207	-136536	-137016	-136536	480	false
	CPOR 7	54573490	54713506	60043525	60165258	-	#2DCDC1	140016	121733	-137016	-127593	-137016	3000	false
	CPOR 7	54585913	54713506	60263153	60353439	-	#2DCDC1	127593	90286	-127593	-127529	-127593	0	true
	CPOR 7	54585977	54714410	60103535	60194274	-	#2DCDC1	128433	90739	-127529	-52160	-127529	904	false

	CPOR 7	54662250	54712720	61190260	61252092	+	#2DCDC1	50470	61832	-52160	-50470	-52160	-1690	true
	CPOR 7	54662250	54713222	60562434	60639043	-	#2DCDC1	50972	76609	-50470	329328	-50470	502	false
	CPOR 7	55042550	55696882	58810743	59301463	-	#2DCDC1	654332	490720	329328	390081	329328	983660	false
	CPOR 7	56086963	56315735	58533863	58653161	-	#2DCDC1	228772	119298	390081	63290	390081	618853	false
	CPOR 7	56379025	56483409	58455317	58516879	+	#2DCDC1	104384	61562	63290	23742	63290	167674	false
	CPOR 7	56507151	56581043	58393346	58455313	-	#2DCDC1	73892	61967	23742	184725	23742	97634	false
	CPOR 7	56765768	56881101	58219664	58275580	-	#2DCDC1	115333	55916	184725	276081	184725	300058	false
	CPOR 7	57157182	57323819	58108563	58193001	-	#2DCDC1	166637	84438	276081	-131718	276081	442718	false
	CPOR 7	57192101	57564148	57912207	58113927	-	#2DCDC1	372047	201720	-131718	30905	-131718	240329	false
	CPOR 7	57595053	57679095	57718350	57780350	-	#2DCDC1	84042	62000	30905	196070	30905	114947	false
	CPOR 7	57875165	58029460	57405417	57519954	-	#2DCDC1	154295	114537	196070	88319	196070	350365	false
	CPOR 7	58117779	58223262	80540369	80606752	+	#2DCDC1	105483	66383	88319	55517	88319	193802	false
	CPOR 7	58278779	58574293	80639912	80882754	+	#2DCDC1	295514	242842	55517		55517	351031	false
**HSA 2**	CPOR 8	37055	684748	79284937	79551842	+	#60FA36	647693	266905		353746			
	CPOR 8	1038494	1548270	79552348	79662028	-	#60FA36	509776	109680	353746	33542	353746	863522	false
	CPOR 8	1581812	9090765	79718859	83718620	-	#60FA36	7508953	3999761	33542	66849	33542	7542495	false
	CPOR 8	9157614	9731412	83728094	84071971	+	#60FA36	573798	343877	66849	68391	66849	640647	false
	CPOR 8	9799803	9953115	96004299	96078691	-	#60FA36	153312	74392	68391	-125021	68391	221703	false
	CPOR 8	9828094	11074848	88289532	88754683	-	#60FA36	1246754	465151	-125021	58196	-125021	1121733	false
	CPOR 8	11133044	15310187	85263285	88288224	-	#60FA36	4177143	3024939	58196	-815622	58196	4235339	false
	CPOR 8	14494565	14688260	91210395	91389564	-	#60FA36	193695	179169	-815622	143704	-815622	-621927	true
	CPOR 8	14831964	15239204	91011822	91210395	-	#60FA36	407240	198573	143704	43502	143704	550944	false
	CPOR 8	15282706	15559200	97506195	97660594	-	#60FA36	276494	154399	43502	-48408	43502	319996	false
	CPOR 8	15510792	15863551	85006221	85157727	-	#60FA36	352759	151506	-48408	202642	-48408	304351	false
	CPOR 8	16066193	16741051	84496118	84943310	-	#60FA36	674858	447192	202642	34262377	202642	877500	false
	CPOR 1	17215029	31586961	220609433	233513972	-	#C18B50	14371932	12904539		74953			
	CPOR 1	31661914	33682916	218592791	220608334	-	#C18B50	2021002	2015543	74953	-52210	74953	2095955	false
	CPOR 1	33630706	36022787	212987617	215196035	-	#C18B50	2392081	2208418	-52210	-1696326	-52210	2339871	false
	CPOR 1	34326461	35197136	217335100	218020905	+	#C18B50	870675	685805	-1696326	48961	-1696326	-825651	true
	CPOR 1	35246097	35723480	218090049	218499885	+	#C18B50	477383	409836	48961	301383	48961	526344	false
	CPOR 1	36024863	36125651	212894788	212970202	+	#C18B50	100788	75414	301383	53	301383	402171	false
	CPOR 1	36125704	53634302	196814789	212888505	-	#C18B50	17508598	16073716	53	40973232	53	17508651	false

	CPOR 16	53634559	56413562	59722145	62564633	-	#98C405	2779003	2842488		3654			
	CPOR 16	56417216	56573542	59575197	59720748	+	#98C405	156326	145551	3654	1758	3654	159980	false
	CPOR 16	56575300	57035651	59150673	59575081	-	#98C405	460351	424408	1758	7001	1758	462109	false
	CPOR 16	57042652	57125923	59082618	59143953	+	#98C405	83271	61335	7001	9385	7001	90272	false
	CPOR 16	57135308	61923744	54679566	59081738	-	#98C405	4788436	4402172	9385	175811	9385	4797821	false
	CPOR 16	62099555	71091031	46403920	54598733	-	#98C405	8991476	8194813	175811	87544	175811	9167287	false
	CPOR 16	71178575	73747048	44177628	46410062	-	#98C405	2568473	2232434	87544	-106732	87544	2656017	false
	CPOR 16	73640316	86866856	32828843	44132349	-	#98C405	13226540	11303506	-106732	258005	-106732	13119808	false
	CPOR 16	87124861	87246317	17426438	17498201	+	#98C405	121456	71763	258005	185130	258005	379461	false
	CPOR 16	87431447	87493090	17687875	17757032	+	#98C405	61643	69157	185130	58559	185130	246773	false
	CPOR 16	87551649	87695749	17776271	17907497	+	#98C405	144100	131226	58559	301947	58559	202659	false
	CPOR 16	87997696	88127486	32735902	32823129	-	#98C405	129790	87227	301947	-29593	301947	431737	false
	CPOR 16	88097893	88755713	32164223	32647107	-	#98C405	657820	482884	-29593	141564	-29593	628227	false
	CPOR 16	88897277	89118339	29986823	30108496	-	#98C405	221062	121673	141564	-206620	141564	362626	false
	CPOR 16	88911719	89048898	31110832	31164772	-	#98C405	137179	53940	-206620	-135660	-206620	-69441	true
	CPOR 16	88913238	89142931	30460435	30555302	+	#98C405	229693	94867	-135660	-219990	-135660	94033	false
	CPOR 16	88922941	89158100	29388585	29525086	-	#98C405	235159	136501	-219990	-235159	-219990	15169	false
	CPOR 16	88922941	89194236	29820114	29952170	-	#98C405	271295	132056	-235159	-271295	-235159	36136	false
	CPOR 16	88922941	89278674	29427399	29609979	-	#98C405	355733	182580	-271295	-353140	-271295	84438	false
	CPOR 16	88925534	89104369	29543152	29609083	-	#98C405	178835	65931	-353140	-173434	-353140	-174305	true
	CPOR 16	88930935	89276259	29999372	30214686	-	#98C405	345324	215314	-173434	-330498	-173434	171890	false
	CPOR 16	88945761	89103370	29559673	29620869	-	#98C405	157609	61196	-330498	-157609	-330498	-172889	true
	CPOR 16	88945761	89117091	29984037	30045801	-	#98C405	171330	61764	-157609	-171302	-157609	13721	false
	CPOR 16	88945789	89119493	30087934	30141159	+	#98C405	173704	53225	-171302	-173337	-171302	2402	false
	CPOR 16	88946156	89049569	31130862	31187689	+	#98C405	103413	56827	-173337	-103377	-173337	-69924	true
	CPOR 16	88946192	89117940	31112872	31203545	-	#98C405	171748	90673	-103377	-171013	-103377	68371	false
	CPOR 16	88946927	89046595	30076208	30136513	-	#98C405	99668	60305	-171013	-99668	-171013	-71345	true
	CPOR 16	88946927	89049080	30252074	30312356	-	#98C405	102153	60282	-99668	-102153	-99668	2485	false
	CPOR 16	88946927	89103297	30252074	30332974	-	#98C405	156370	80900	-102153	-156370	-102153	54217	false
	CPOR 16	88946927	89278674	29258941	29429280	-	#98C405	331747	170339	-156370	-331433	-156370	175377	false
	CPOR 16	88947241	89197070	30345408	30522227	-	#98C405	249829	176819	-331433	-246340	-331433	-81604	true
	CPOR 16	88950730	89049269	28102716	28207846	-	#98C405	98539	105130	-246340	-98539	-246340	-147801	true

	CPOR 16	88950730	89049569	28180984	28267513	-	#98C405	98839	86529	-98539	-98808	-98539	300	false
	CPOR 16	88950761	89161951	29273280	29327109	-	#98C405	211190	53829	-98808	-203398	-98808	112382	false
	CPOR 16	88958553	89049569	28348918	28404699	-	#98C405	91016	55781	-203398	-84713	-203398	-112382	true
	CPOR 16	88964856	89330024	30034773	30164099	-	#98C405	365168	129326	-84713	-363822	-84713	280455	false
	CPOR 16	88966202	89119209	30440006	30522227	-	#98C405	153007	82221	-363822	-140933	-363822	-210815	true
	CPOR 16	88978276	89118339	29584890	29713909	-	#98C405	140063	129019	-140933	-20016	-140933	-870	true
	CPOR 16	89098323	89171476	29877319	29929866	-	#98C405	73153	52547	-20016	-71987	-20016	53137	false
	CPOR 16	89099489	89301247	30188353	30312356	-	#98C405	201758	124003	-71987	-145700	-71987	129771	false
	CPOR 16	89155547	89245684	29142388	29218931	-	#98C405	90137	76543	-145700	-24194	-145700	-55563	true
	CPOR 16	89221490	89330071	29494233	29601669	-	#98C405	108581	107436	-24194	-56025	-24194	84387	false
	CPOR 16	89274046	89327042	29717737	29829457	-	#98C405	52996	111720	-56025	514748	-56025	-3029	true
	CPOR 16	89841790	90025329	29143718	29217894	+	#98C405	183539	74176	514748	-183539	514748	698287	false
	CPOR 16	89841790	90115540	29494677	29713846	+	#98C405	273750	219169	-183539	-273750	-183539	90211	false
	CPOR 16	89841790	90151452	29276782	29327113	+	#98C405	309662	50331	-273750	-303881	-273750	35912	false
	CPOR 16	89847571	90222772	30002485	30164099	+	#98C405	375201	161614	-303881	-371749	-303881	71320	false
	CPOR 16	89851023	90211919	30025775	30164099	+	#98C405	360896	138324	-371749	-349051	-371749	-10853	true
	CPOR 16	89862868	89930314	30189087	30263671	+	#98C405	67446	74584	-349051	-49083	-349051	-281605	true
	CPOR 16	89881231	90101107	30188353	30332974	+	#98C405	219876	144621	-49083	-197236	-49083	170793	false
	CPOR 16	89903871	90101064	29258941	29325989	+	#98C405	197193	67048	-197236	-197193	-197236	-43	true
	CPOR 16	89903871	90222916	29427399	29554862	+	#98C405	319045	127463	-197193	-319045	-197193	121852	false
	CPOR 16	89903871	90224114	29844144	30062705	+	#98C405	320243	218561	-319045	-238404	-319045	1198	false
	CPOR 16	89985710	90221755	30345408	30491060	+	#98C405	236045	145652	-238404	-211282	-238404	-2359	true
	CPOR 16	90010473	90221619	29967220	30044609	+	#98C405	211146	77389	-211282	-201633	-211282	-136	true
	CPOR 16	90019986	90207065	29273280	29327109	+	#98C405	187079	53829	-201633	-181736	-201633	-14554	true
	CPOR 16	90025329	90222916	29358971	29546110	+	#98C405	197587	187139	-181736	-144133	-181736	15851	false
	CPOR 16	90078783	90222916	29543152	29620869	+	#98C405	144133	77717	-144133	-143530	-144133	0	true
	CPOR 16	90079386	90221441	30439947	30522227	+	#98C405	142055	82280	-143530	-141649	-143530	-1475	true
	CPOR 16	90079792	90221631	29559673	29623761	+	#98C405	141839	64088	-141649	-141766	-141649	190	false
	CPOR 16	90079865	90221755	30252074	30332974	+	#98C405	141890	80900	-141766	-141708	-141766	124	false
	CPOR 16	90080047	90222486	31110832	31203545	+	#98C405	142439	92713	-141708	-107440	-141708	731	false
	CPOR 16	90115046	90221557	30472569	30555302	-	#98C405	106511	82733	-107440	-70560	-107440	-929	true
	CPOR 16	90150997	90207100	28180984	28267513	+	#98C405	56103	86529	-70560	-56103	-70560	-14457	true

CPOR 16	90150997	90222916	29102501	29165192	+	#98C405	71919	62691	-56103	-69340	-56103	15816	false
CPOR 16	90153576	90221744	30082108	30164001	+	#98C405	68168	81893	-69340	-68168	-69340	-1172	true
CPOR 16	90153576	90222772	30383714	30441572	+	#98C405	69196	57858	-68168	-68358	-68168	1028	false
CPOR 16	90154414	90235559	29623355	29713909	+	#98C405	81145	90554	-68358	-80810	-68358	12787	false
CPOR 16	90154749	90221755	29428950	29507264	+	#98C405	67006	78314	-80810	4757653	-80810	-13804	true
CPOR 8	94799874	94850761	96179428	96229903	-	#60FA36	50887	50475	43729705	-50887	43729705	43780592	false
CPOR 8	94799874	94854439	101819742	101906324	+	#60FA36	54565	86582	-50887	2666483	-50887	3678	false
CPOR 24	94801576	94854439	46464945	46541230	-	#00FFFF	52863	76285		2654618			
CPOR 16	94979408	95414295	18561974	18864991	+	#98C405	434887	303017	4757653	227687	4757653	5192540	false
CPOR 5	95500001	95558810	46679023	46775798	+	#1240C3	58809	96775		-39223			
CPOR 5	95519587	95591869	46688441	46789761	-	#1240C3	72282	101320	-39223		-39223	33059	false
CPOR 16	95641982	95743177	15879417	15978543	-	#98C405	101195	99126	227687	281219	227687	328882	false
CPOR 16	96024396	96375948	18866217	19139468	+	#98C405	351552	273251	281219	97290	281219	632771	false
CPOR 16	96473238	96979218	19140846	19500011	+	#98C405	505980	359165	97290	341752	97290	603270	false
CPOR 16	97320970	97377278	30348787	30417873	-	#98C405	56308	69086	341752	-46051	341752	398060	false
CPOR 16	97331227	97387534	30348787	30417873	+	#98C405	56307	69086	-46051	255965	-46051	10256	false
CPOR 24	97509057	97585893	46464944	46529951	-	#00FFFF	76836	65007	2654618	110124772	2654618	2731454	false
CPOR 8	97520922	97584276	97930496	98002131	+	#60FA36	63354	71635	2666483	-63354	2666483	2729837	false
CPOR 8	97520922	97585893	101836031	101906325	+	#60FA36	64971	70294	-63354		-63354	1617	false
CPOR 16	97643499	99154642	19510473	20673278	+	#98C405	1511143	1162805	255965	39	255965	1767108	false
CPOR 16	99154681	101195745	20735506	22281566	+	#98C405	2041064	1546060	39	54827	39	2041103	false
CPOR 16	101250572	102281034	22392147	23074575	+	#98C405	1030462	682428	54827	-58042	54827	1085289	false
CPOR 16	102222992	102473346	23076995	23231909	-	#98C405	250354	154914	-58042	-250354	-58042	192312	false
CPOR 16	102222992	105964771	23284987	25991341	+	#98C405	3741779	2706354	-250354	50302	-250354	3491425	false
CPOR 16	106015073	106203482	25995013	26079607	+	#98C405	188409	84594	50302	327681	50302	238711	false
CPOR 16	106531163	107825036	26120003	26861944	+	#98C405	1293873	741941	327681	99046	327681	1621554	false
CPOR 16	107924082	108181013	26862848	27043198	+	#98C405	256931	180350	99046	73008	99046	355977	false
CPOR 16	108254021	108304711	27187800	27238950	+	#98C405	50690	51150	73008	-41316	73008	123698	false
CPOR 16	108263395	108321975	27079444	27168495	+	#98C405	58580	89051	-41316	61411	-41316	17264	false
CPOR 16	108383386	109725990	27253539	28091975	+	#98C405	1342604	838436	61411	65097	61411	1404015	false
CPOR 16	109791087	109841441	27419072	27472024	+	#98C405	50354	52952	65097	223300	65097	115451	false
CPOR 16	110064741	110235529	18480577	18580690	-	#98C405	170788	100113	223300	294536	223300	394088	false

CPOR 16	110530065	110580402	27419072	27472024	-	#98C405	50337	52952	294536	55660	294536	344873	false
CPOR 16	110636062	111399171	17776271	18431281	-	#98C405	763109	655010	55660	57964	55660	818769	false
CPOR 16	111457135	112037683	17276351	17757032	-	#98C405	580548	480681	57964	24901	57964	638512	false
CPOR 16	112062584	112345869	16935070	17177090	-	#98C405	283285	242020	24901	93304	24901	308186	false
CPOR 16	112439173	112907165	16566235	16929604	-	#98C405	467992	363369	93304	-123769	93304	561296	false
CPOR 16	112783396	112916797	16486121	16566913	+	#98C405	133401	80792	-123769	-10757	-123769	9632	false
CPOR 16	112906040	113033149	16358432	16482824	+	#98C405	127109	124392	-10757	-127109	-10757	116352	false
CPOR 16	112906040	113384792	15879417	16382052	-	#98C405	478752	502635	-127109		-127109	351643	false
CPOR 18	113672181	119406730	61206891	66858353	+	#80E0F6	5734549	5651462		464			
CPOR 18	119407194	127196526	67138814	74553928	+	#80E0F6	7789332	7415114	464	56090	464	7789796	false
CPOR 18	127252616	129483268	74581674	76828085	+	#80E0F6	2230652	2246411	56090	191703	56090	2286742	false
CPOR 18	129674971	129920729	76904125	77271187	+	#80E0F6	245758	367062	191703	415368	191703	437461	false
CPOR 17	130135519	130196446	43424751	43476889	+	#FAB28C	60927	52138		1281642			
CPOR 18	130336097	130387596	78101371	78198288	-	#80E0F6	51499	96917	415368	126293	415368	466867	false
CPOR 18	130513889	130566559	78027228	78100483	+	#80E0F6	52670	73255	126293	-12375	126293	178963	false
CPOR 18	130554184	130606867	78027228	78100483	-	#80E0F6	52683	73255	-12375	113336	-12375	40308	false
CPOR 18	130720203	131157934	77359838	77836041	-	#80E0F6	437731	476203	113336	1208574	113336	551067	false
CPOR 17	131478088	131537437	43424751	43476889	-	#FAB28C	59349	52138	1281642	69945461	1281642	1340991	false
CPOR 18	132366508	178164184	19092465	61176548	-	#80E0F6	45797676	42084083	1208574	-55703	1208574	47006250	false
CPOR 18	178108481	183544663	14293927	19055075	-	#80E0F6	5436182	4761148	-55703	2334	-55703	5380479	false
CPOR 18	183546997	183771413	14084134	14290440	+	#80E0F6	224416	206306	2334	12799	2334	226750	false
CPOR 18	183784212	193236620	6341869	14084055	-	#80E0F6	9452408	7742186	12799	-52806	12799	9465207	false
CPOR 18	193183814	194116180	5722365	6341463	-	#80E0F6	932366	619098	-52806	52532	-52806	879560	false
CPOR 18	194168712	200778123	976278	5722217	-	#80E0F6	6609411	4745939	52532	-180799	52532	6661943	false
CPOR 18	200597324	201481682	419592	1006561	-	#80E0F6	884358	586969	-180799	140167	-180799	703559	false
CPOR 18	201621849	201783809	259315	415633	-	#80E0F6	161960	156318	140167		140167	302127	false
CPOR 27	201778160	208499027	8615763	14633358	-	#682292	6720867	6017595		98990			
CPOR 27	208598017	218475084	576	8602314	-	#682292	9877067	8601738	98990	6271341	98990	9976057	false
CPOR 1	218435809	224710822	36451	6006258	+	#C18B50	6275013	5969807	104890089		104890089	111165102	false
CPOR 27	224746425	230400016	15310813	20133588	+	#682292	5653591	4822775	6271341	-30274	6271341	11924932	false
CPOR 27	230369742	231536383	20017077	20761376	+	#682292	1166641	744299	-30274	56115	-30274	1136367	false
CPOR 27	231592498	233688404	20761401	22087616	+	#682292	2095906	1326215	56115	-100895	56115	2152021	false

	CPOR 27	233587509	240110786	22214593	26760506	+	#682292	6523277	4545913	-100895	-32029	-100895	6422382	false
	CPOR 27	240078757	240646317	26789189	27137331	+	#682292	567560	348142	-32029	65117	-32029	535531	false
	CPOR 27	240711434	241810090	27138807	27863192	+	#682292	1098656	724385	65117	-18250	65117	1163773	false
	CPOR 27	241791840	241892126	27994179	28080366	+	#682292	100286	86187	-18250		-18250	82036	false
**HSA 20**	CPOR 6	85809	740907	68826509	69398163	-	#0C404C	655098	571654		7493			
	CPOR 6	748400	962613	68534698	68687596	-	#0C404C	214213	152898	7493	-78616	7493	221706	false
	CPOR 6	883997	952386	68352722	68421222	-	#0C404C	68389	68500	-78616	-11792	-78616	-10227	true
	CPOR 6	940594	1521865	67671461	68233462	-	#0C404C	581271	562001	-11792	39492	-11792	569479	false
	CPOR 6	1561357	1694746	67311600	67427503	-	#0C404C	133389	115903	39492	-133389	39492	172881	false
	CPOR 6	1561357	1694752	67596028	67676099	-	#0C404C	133395	80071	-133389	-133395	-133389	6	false
	CPOR 6	1561357	1702835	67364355	67442948	-	#0C404C	141478	78593	-133395	-141478	-133395	8083	false
	CPOR 6	1561357	1711268	67254070	67313663	-	#0C404C	149911	59593	-141478	-145453	-141478	8433	false
	CPOR 6	1565815	1722167	67482233	67595943	-	#0C404C	156352	113710	-145453	-147935	-145453	10899	false
	CPOR 6	1574232	1702837	67434857	67502502	-	#0C404C	128605	67645	-147935	-128603	-147935	-19330	true
	CPOR 6	1574234	1721509	67473806	67541118	-	#0C404C	147275	67312	-128603	42345	-128603	18672	false
	CPOR 6	1763854	5444701	48426408	51637051	+	#0C404C	3680847	3210643	42345	54837	42345	3723192	false
	CPOR 6	5499538	18223139	51646659	62821463	+	#0C404C	12723601	11174804	54837	157633	54837	12778438	false
	CPOR 6	18380772	23926606	62809224	66431637	+	#0C404C	5545834	3622413	157633	-292978	157633	5703467	false
	CPOR 6	23633628	23777422	66330223	66430042	+	#0C404C	143794	99819	-292978	-92108	-292978	-149184	true
	CPOR 6	23685314	23942604	66297604	66394486	+	#0C404C	257290	96882	-92108	102380	-92108	165182	false
	CPOR 6	24044984	24823824	66438214	66758771	+	#0C404C	778840	320557	102380	84469	102380	881220	false
	CPOR 6	24908293	25624405	66765474	67165169	+	#0C404C	716112	399695	84469	5670721	84469	800581	false
	CPOR 8	30666621	30721121	101819742	101906324	+	#60FA36	54500	86582		-54500			
	CPOR 8	30666621	30721238	96155091	96229903	-	#60FA36	54617	74812	-54500		-54500	117	false
	CPOR 6	31295126	31451060	69443714	69556327	+	#0C404C	155934	112613	5670721	9041	5670721	5826655	false
	CPOR 6	31460101	33183960	69613585	71012358	+	#0C404C	1723859	1398773	9041	22274	9041	1732900	false
	CPOR 6	33206234	34199051	71218693	72187637	+	#0C404C	992817	968944	22274	-29946	22274	1015091	false
	CPOR 6	34169105	38440657	72213030	75996855	+	#0C404C	4271552	3783825	-29946	-20608	-29946	4241606	false
	CPOR 6	38420049	38676963	76035229	76231140	+	#0C404C	256914	195911	-20608	-150501	-20608	236306	false
	CPOR 6	38526462	38688536	76316968	76406265	-	#0C404C	162074	89297	-150501	-67811	-150501	11573	false
	CPOR 6	38620725	45229369	76423930	82392292	+	#0C404C	6608644	5968362	-67811	-105735	-67811	6540833	false
	CPOR 6	45123634	45248281	82305104	82481171	-	#0C404C	124647	176067	-105735	-100200	-105735	18912	false

	CPOR 6	45148081	45449162	82361779	82723623	+	#0C404C	301081	361844	-100200	6423	-100200	200881	false
	CPOR 6	45455585	45728658	83160171	83314227	+	#0C404C	273073	154056	6423	-26885	6423	279496	false
	CPOR 6	45701773	59521485	83326757	94403416	+	#0C404C	13819712	11076659	-26885	-11246446	-26885	13792827	false
	CPOR 6	48275039	48570378	84963892	85181071	-	#0C404C	295339	217179	-11246446	10975242	-11246446	-10951107	true
	CPOR 6	59545620	64276314	94776590	98614970	+	#0C404C	4730694	3838380	10975242		10975242	15705936	false
**HSA 21**	CPOR 14	5011613	5165890	65369529	65466078	-	#DD528A	154277	96549		800627			
	CPOR 14	5966517	6076907	67718132	67811698	-	#DD528A	110390	93566	800627	3172607	800627	911017	false
	CPOR 8	13918700	13976434	101819742	101906324	+	#60FA36	57734	86582		-57734			
	CPOR 8	13918700	13976557	96155091	96229903	-	#60FA36	57857	74812	-57734		-57734	123	false
	CPOR 14	14105860	17273027	51469919	54137694	+	#DD528A	3167167	2667775	4805604	51311	4805604	7972771	false
	CPOR 14	17324338	23665031	54159763	59499167	+	#DD528A	6340693	5339404	51311	-1855763	51311	6392004	false
	CPOR 14	21809268	21932233	61032968	61125538	+	#DD528A	122965	92570	-1855763	473655	-1855763	-1732798	true
	CPOR 14	22405888	22608637	61259538	61386556	+	#DD528A	202749	127018	473655	66355	473655	676404	false
	CPOR 14	22674992	23060161	61386604	61689875	+	#DD528A	385169	303271	66355	103815	66355	451524	false
	CPOR 14	23163976	23943543	61689898	62294632	+	#DD528A	779567	604734	103815	-278192	103815	883382	false
	CPOR 14	23665351	24513475	59563292	60095848	+	#DD528A	848124	532556	-278192	-555205	-278192	569932	false
	CPOR 14	23958270	24092804	63304321	63412620	+	#DD528A	134534	108299	-555205	-95206	-555205	-420671	true
	CPOR 14	23997598	25391714	62306600	63416879	+	#DD528A	1394116	1110279	-95206	-1115995	-95206	1298910	false
	CPOR 14	24275719	25172621	60217630	60957929	+	#DD528A	896902	740299	-1115995	-372074	-1115995	-219093	true
	CPOR 14	24800547	24926676	59366574	59456122	+	#DD528A	126129	89548	-372074	190487	-372074	-245945	true
	CPOR 14	25117163	25228781	59649010	59740694	+	#DD528A	111618	91684	190487	67577	190487	302105	false
	CPOR 14	25296358	25391714	60958555	61032968	+	#DD528A	95356	74413	67577	8081726	67577	162933	false
	CPOR 7	25371677	28859123	80964311	84237062	+	#2DCDC1	3487446	3272751	12029479	64176	12029479	15516925	false
	CPOR 7	28923299	30640335	84288245	86091250	+	#2DCDC1	1717036	1803005	64176	-4038	64176	1781212	false
	CPOR 7	30636297	30751789	86249548	86399203	+	#2DCDC1	115492	149655	-4038	-9146	-4038	111454	false
	CPOR 7	30742643	32535052	86402787	88046474	+	#2DCDC1	1792409	1643687	-9146	60734	-9146	1783263	false
	CPOR 7	32595786	33480516	88046539	88883894	+	#2DCDC1	884730	837355	60734		60734	945464	false
	CPOR 14	33473440	36075957	71035463	73461731	-	#DD528A	2602517	2426268	8081726	36808	8081726	10684243	false
	CPOR 14	36112765	39805344	67928954	70902264	-	#DD528A	3692579	2973310	36808	31629	36808	3729387	false
	CPOR 14	39836973	41283948	65632565	66595228	+	#DD528A	1446975	962663	31629	7461	31629	1478604	false
	CPOR 14	41291409	41409178	66597437	66653658	-	#DD528A	117769	56221	7461	310266	7461	125230	false
	CPOR 14	41719444	42789353	66782171	67434492	+	#DD528A	1069909	652321	310266	58663	310266	1380175	false

	CPOR 14	42848016	43131622	67438671	67612967	+	#DD528A	283606	174296	58663	140934	58663	342269	false
	CPOR 14	43272556	43376608	67649781	67716172	+	#DD528A	104052	66391	140934	84749	140934	244986	false
	CPOR 14	43461357	43694566	67718132	67902555	+	#DD528A	233209	184423	84749	20844	84749	317958	false
	CPOR 14	43715410	44469116	65150654	65616524	+	#DD528A	753706	465870	20844	30088	20844	774550	false
	CPOR 14	44499204	46666244	63443130	65150654	-	#DD528A	2167040	1707524	30088		30088	2197128	false
**HSA 22**	CPOR 10	17141817	18178377	44225	943290	+	#6A78D4	1036560	899065					
	CPOR 17	19021794	20323822	42124912	43223087	-	#FAB28C	1302028	1098175		69119			
	CPOR 17	20392941	20626226	43226098	43458494	+	#FAB28C	233285	232396	69119	74091	69119	302404	false
	CPOR 17	20700317	21081007	43461668	43846927	-	#FAB28C	380690	385259	74091	463524	74091	454781	false
	CPOR 17	21544531	21985513	41559149	41933505	-	#FAB28C	440982	374356	463524	355525	463524	904506	false
	CPOR 17	22341038	22450969	50791701	50847111	+	#FAB28C	109931	55410	355525	24617	355525	465456	false
	CPOR 17	22475586	22610793	51363478	51450568	+	#FAB28C	135207	87090	24617	-9291	24617	159824	false
	CPOR 17	22601502	22772025	51967302	52080720	+	#FAB28C	170523	113418	-9291	-4866	-9291	161232	false
	CPOR 17	22767159	22882043	51724760	51785224	+	#FAB28C	114884	60464	-4866	-113158	-4866	110018	false
	CPOR 17	22768885	22882111	51502731	51633276	+	#FAB28C	113226	130545	-113158	-4580	-113158	68	false
	CPOR 17	22877531	23347362	52077162	52562317	+	#FAB28C	469831	485155	-4580	390079	-4580	465251	false
	CPOR 17	23737441	24250892	52921752	53327959	-	#FAB28C	513451	406207	390079	15579	390079	903530	false
	CPOR 17	24266471	24636386	52529075	52929708	+	#FAB28C	369915	400633	15579	79376	15579	385494	false
	CPOR 17	24715762	24791836	55354835	55440855	+	#FAB28C	76074	86020	79376	-56550	79376	155450	false
	CPOR 17	24735286	25276737	44022314	44423786	-	#FAB28C	541451	401472	-56550	178103	-56550	484901	false
	CPOR 17	25454840	25516018	44037990	44091701	-	#FAB28C	61178	53711	178103	-44225	178103	239281	false
	CPOR 17	25471793	25730990	43855986	44031210	-	#FAB28C	259197	175224	-44225	2265	-44225	214972	false
	CPOR 17	25733255	29398529	44483510	47808192	+	#FAB28C	3665274	3324682	2265	17927	2265	3667539	false
	CPOR 17	29416456	32009282	47770044	50255051	+	#FAB28C	2592826	2485007	17927	24829	17927	2610753	false
	CPOR 17	32034111	32111152	50317342	50387512	+	#FAB28C	77041	70170	24829		24829	101870	false
	CPOR 8	32387502	33140349	1648828	2085917	-	#60FA36	752847	437089		59166			
	CPOR 8	33199515	35456085	371236	1648808	-	#60FA36	2256570	1277572	59166	52357	59166	2315736	false
	CPOR 8	35508442	35625560	305237	367062	-	#60FA36	117118	61825	52357		52357	169475	false
	CPOR 22	35656240	36130755	47832066	48211389	+	#F7FE2D	474515	379323		8057			
	CPOR 22	36138812	36266502	48362845	48431580	-	#F7FE2D	127690	68735	8057	-125699	8057	135747	false
	CPOR 22	36140803	36278011	48506893	48570317	-	#F7FE2D	137208	63424	-125699	-21714	-125699	11509	false
	CPOR 22	36256297	38600987	48804828	50626403	+	#F7FE2D	2344690	1821575	-21714	38850	-21714	2322976	false

	CPOR 22	38639837	38942282	50714592	50952766	+	#F7FE2D	302445	238174	38850	157102	38850	341295	false
	CPOR 22	39099384	42095238	50983541	53349301	+	#F7FE2D	2995854	2365760	157102	33275	157102	3152956	false
	CPOR 22	42128513	47363028	54198137	57337032	+	#F7FE2D	5234515	3138895	33275	805	33275	5267790	false
	CPOR 22	47363833	47479851	57338492	57434042	-	#F7FE2D	116018	95550	805	8763	805	116823	false
	CPOR 22	47488614	50783890	57434489	59845734	+	#F7FE2D	3295276	2411245	8763		8763	3304039	false
**HSA 3**	CPOR 20	12066	5160223	54678304	59977223	+	#00FF7F	5148157	5298919		-53286			
	CPOR 20	5106937	6326628	60139875	61540048	+	#00FF7F	1219691	1400173	-53286	52237	-53286	1166405	false
	CPOR 20	6378865	8442826	61628788	63763684	+	#00FF7F	2063961	2134896	52237	24242120	52237	2116198	false
	CPOR 14	8427346	9568141	1103474	2197843	-	#DD528A	1140795	1094369		81087			
	CPOR 14	9649228	10727172	111898	1029924	-	#DD528A	1077944	918026	81087	27395074	81087	1159031	false
	CPOR 22	10753986	11855486	19228818	20017799	-	#F7FE2D	1101500	788981		74494			
	CPOR 22	11929980	12533168	18699551	19228541	-	#F7FE2D	603188	528990	74494	3980	74494	677682	false
	CPOR 22	12537148	12842398	4810356	5035136	+	#F7FE2D	305250	224780	3980	55328	3980	309230	false
	CPOR 22	12897726	13897637	17566294	18295166	+	#F7FE2D	999911	728872	55328	211862	55328	1055239	false
	CPOR 22	14109499	15102688	5457365	6068790	-	#F7FE2D	993189	611425	211862	111464560	211862	1205051	false
	CPOR 12	15200927	15357432	32300050	32460447	-	#00007F	156505	160397		45134			
	CPOR 12	15402566	15611793	32053594	32250812	+	#00007F	209227	197218	45134	-14961	45134	254361	false
	CPOR 12	15596832	19765134	7379674	11483834	+	#00007F	4168302	4104160	-14961	52089	-14961	4153341	false
	CPOR 12	19817223	20348862	11615703	12196429	+	#00007F	531639	580726	52089	1	52089	583728	false
	CPOR 12	20348863	20645711	12196438	12472327	-	#00007F	296848	275889	1	0	1	296849	false
	CPOR 12	20645711	32650039	12472333	24392446	+	#00007F	12004328	11920113	0	19665098	0	12004328	false
	CPOR 20	32684946	38138616	63790711	68960963	-	#00FF7F	5453670	5170252	24242120	28075035	24242120	29695790	false
	CPOR 14	38122246	38267285	2480685	2698291	-	#DD528A	145039	217606	27395074	57745	27395074	27540113	false
	CPOR 14	38325030	39381900	2698021	3696854	+	#DD528A	1056870	998833	57745	35861711	57745	1114615	false
	CPOR 13	39383014	40054743	17379444	17742331	-	#7F00FF	671729	362887		76158			
	CPOR 13	40130901	40463187	17217882	17377281	-	#7F00FF	332286	159399	76158	70314	76158	408444	false
	CPOR 13	40533501	46756275	13573334	17212674	-	#7F00FF	6222774	3639340	70314	53546	70314	6293088	false
	CPOR 13	46809821	48199660	12610163	13583598	-	#7F00FF	1389839	973435	53546	17336633	53546	1443385	false
	CPOR 6	48348173	48509879	47520678	47631742	-	#0C404C	161706	111064		5474			
	CPOR 6	48515353	52313051	43822599	47467152	-	#0C404C	3797698	3644553	5474	77899725	5474	3803172	false
	CPOR 12	52315137	58228598	32461393	38114432	+	#00007F	5913461	5653039	19665098	-23021	19665098	25578559	false
	CPOR 12	58205577	59064453	31326225	32038442	-	#00007F	858876	712217	-23021	2260	-23021	835855	false

CPOR 12	59066713	59117889	31248373	31323954	+	#00007F	51176	75581	2260	0	2260	53436	false
CPOR 12	59117889	62331123	28153206	31248371	-	#00007F	3213234	3095165	0	-144039	0	3213234	false
CPOR 12	62187084	66141471	24392502	28152993	-	#00007F	3954387	3760491	-144039	25215739	-144039	3810348	false
CPOR 20	66213651	75243253	46073111	54709391	+	#00FF7F	9029602	8636280	28075035	50758887	28075035	37104637	false
CPOR 14	75243611	75325561	5089291	5192744	-	#DD528A	81950	103453	35861711	484875	35861711	35943661	false
CPOR 17	75414969	75512459	36535404	36600486	-	#FAB28C	97490	65082		4293			
CPOR 17	75516752	75591357	36437925	36514257	+	#FAB28C	74605	76332	4293	50164025	4293	78898	false
CPOR 14	75810436	77841562	49552506	51454185	-	#DD528A	2031126	1901679	484875	4289	484875	2516001	false
CPOR 14	77845851	77969493	49411071	49552446	+	#DD528A	123642	141375	4289	27925	4289	127931	false
CPOR 14	77997418	78073973	49316357	49393733	+	#DD528A	76555	77376	27925	0	27925	104480	false
CPOR 14	78073973	90261169	38701103	49315552	-	#DD528A	12187196	10614449	0	3547437	0	12187196	false
CPOR 14	93808606	95340565	37487699	38793475	-	#DD528A	1531959	1305776	3547437	-1308302	3547437	5079396	false
CPOR 14	94032263	95352807	33020998	34192369	+	#DD528A	1320544	1171371	-1308302	52194	-1308302	12242	false
CPOR 14	95405001	95526685	31055527	31166569	-	#DD528A	121684	111042	52194	-121684	52194	173878	false
CPOR 14	95405001	96640693	34192657	35126990	+	#DD528A	1235692	934333	-121684	-1114487	-121684	1114008	false
CPOR 14	95526206	96660756	36679547	37450763	-	#DD528A	1134550	771216	-1114487	-759417	-1114487	20063	false
CPOR 14	95901339	96803782	32305142	32976212	+	#DD528A	902443	671070	-759417	-138626	-759417	143026	false
CPOR 14	96665156	98039919	31182746	32304380	-	#DD528A	1374763	1121634	-138626	20535	-138626	1236137	false
CPOR 14	98060454	98328591	7090214	7166252	-	#DD528A	268137	76038	20535	4704	20535	288672	false
CPOR 14	98333295	98609714	30599454	30872753	-	#DD528A	276419	273299	4704	14469	4704	281123	false
CPOR 14	98624183	100484405	28624928	30497486	-	#DD528A	1860222	1872558	14469	-2303	14469	1874691	false
CPOR 14	100482102	103984870	25017752	28459253	-	#DD528A	3502768	3441501	-2303	10178	-2303	3500465	false
CPOR 14	103995048	104096678	24933707	25017214	+	#DD528A	101630	83507	10178	1571	10178	111808	false
CPOR 14	104098249	108524763	20864436	24933571	-	#DD528A	4426514	4069135	1571	-171568	1571	4428085	false
CPOR 14	108353195	112139397	17141472	20820782	-	#DD528A	3786202	3679310	-171568	183848	-171568	3614634	false
CPOR 14	112323245	120957664	8571476	17126241	-	#DD528A	8634419	8554765	183848	50355	183848	8818267	false
CPOR 14	121008019	122344117	7295377	8558179	-	#DD528A	1336098	1262802	50355	-22760	50355	1386453	false
CPOR 14	122321357	125524976	3708411	6837243	-	#DD528A	3203619	3128832	-22760		-22760	3180859	false
CPOR 17	125755382	125822670	36437925	36514257	-	#FAB28C	67288	76332	50164025	4281	50164025	50231313	false
CPOR 17	125826951	125934781	36535404	36600602	+	#FAB28C	107830	65198	4281	4156457	4281	112111	false
CPOR 22	126567248	128816833	6101597	7914244	+	#F7FE2D	2249585	1812647	111464560	271553	111464560	113714145	false
CPOR 8	128875928	128995083	103258478	103341407	-	#60FA36	119155	82929		-28			

	CPOR 8	128995055	129067723	90731213	90785425	+	#60FA36	72668	54212	-28		-28	72640	false
	CPOR 22	129088386	129278320	11857242	12015139	+	#F7FE2D	189934	157897	271553	116730	271553	461487	false
	CPOR 22	129395050	129994482	5029298	5456788	+	#F7FE2D	599432	427490	116730		116730	716162	false
	CPOR 6	130212776	130286544	43562938	43689033	-	#0C404C	73768	126095	77899725	54226	77899725	77973493	false
	CPOR 6	130340770	138637925	35652306	43562356	-	#0C404C	8297155	7910050	54226	12530	54226	8351381	false
	CPOR 6	138650455	139042767	35159828	35582357	-	#0C404C	392312	422529	12530	-37758	12530	404842	false
	CPOR 6	139005009	142241255	32119640	35159448	-	#0C404C	3236246	3039808	-37758	55640	-37758	3198488	false
	CPOR 6	142296895	145193064	29306953	32096138	-	#0C404C	2896169	2789185	55640	46	55640	2951809	false
	CPOR 6	145193110	145279010	29214444	29306951	+	#0C404C	85900	92507	46	1940	46	85946	false
	CPOR 6	145280950	146284980	28139568	29193338	-	#0C404C	1004030	1053770	1940	44924	1940	1005970	false
	CPOR 6	146329904	149812192	24658653	28187536	-	#0C404C	3482288	3528883	44924	-2963	44924	3527212	false
	CPOR 6	149809229	151833818	22146643	24067457	-	#0C404C	2024589	1920814	-2963	-57147	-2963	2021626	false
	CPOR 6	151776671	151833824	22065451	22158151	-	#0C404C	57153	92700	-57147	-57153	-57147	6	false
	CPOR 6	151776671	151842910	22004955	22071013	-	#0C404C	66239	66058	-57153	-50773	-57153	9086	false
	CPOR 6	151792137	159417464	14703746	21900423	-	#0C404C	7625327	7196677	-50773	0	-50773	7574554	false
	CPOR 6	159417464	159515475	14613558	14703087	+	#0C404C	98011	89529	0	11	0	98011	false
	CPOR 6	159515486	163704520	10818955	14609876	-	#0C404C	4189034	3790921	11	57470	11	4189045	false
	CPOR 6	163761990	166530596	8153833	10800255	-	#0C404C	2768606	2646422	57470	78208	57470	2826076	false
	CPOR 6	166608804	166732778	8009894	8082879	-	#0C404C	123974	72985	78208	62787	78208	202182	false
	CPOR 6	166795565	167172132	7620056	7991266	-	#0C404C	376567	371210	62787		62787	439354	false
	CPOR 11	167638488	171499576	68444469	72247757	-	#6E0025	3861088	3803288		43945			
	CPOR 11	171543521	182728884	57927822	68280999	-	#6E0025	11185363	10353177	43945		43945	11229308	false
	CPOR 13	182729166	183121751	8586	354324	+	#7F00FF	392585	345738	57099725	0	57099725	57492310	false
	CPOR 13	183121751	184468598	11315652	12479221	-	#7F00FF	1346847	1163569	0	28008	0	1346847	false
	CPOR 13	184496606	194498238	2746856	11263245	-	#7F00FF	10001632	8516389	28008	43761	28008	10029640	false
	CPOR 13	194541999	195600691	2014769	2780594	-	#7F00FF	1058692	765825	43761	98825	43761	1102453	false
	CPOR 13	195699516	195923257	641688	804056	+	#7F00FF	223741	162368	98825	125996	98825	322566	false
	CPOR 13	196049253	197610193	804496	2014351	+	#7F00FF	1560940	1209855	125996	57327	125996	1686936	false
	CPOR 13	197667520	198044771	354421	629946	-	#7F00FF	377251	275525	57327		57327	434578	false
**HSA 4**	CPOR 16	498744	1659398	14837922	15633177	-	#98C405	1160654	795255		-4529			
	CPOR 16	1654869	2841422	63	819585	+	#98C405	1186553	819522	-4529	317	-4529	1182024	false
	CPOR 16	2841739	3863252	1092445	1708551	+	#98C405	1021513	616106	317	333858	317	1021830	false

CPOR 17	3935273	4049608	36535404	36600549	-	#FAB28C	114335	65145		3233			
CPOR 17	4052841	4118987	36437925	36514257	+	#FAB28C	66146	76332	3233	4870760	3233	69379	false
CPOR 16	4197110	8478611	1899213	4487190	-	#98C405	4281501	2587977	333858	111514	333858	4615359	false
CPOR 16	8590125	8913117	1709100	1898853	-	#98C405	322992	189753	111514	850042	111514	434506	false
CPOR 17	8989747	9058438	36437925	36514257	-	#FAB28C	68691	76332	4870760	4285	4870760	4939451	false
CPOR 17	9062723	9171443	36535404	36598244	+	#FAB28C	108720	62840	4285	349763	4285	113005	false
CPOR 17	9521206	9590555	36437925	36514257	-	#FAB28C	69349	76332	349763	4279	349763	419112	false
CPOR 17	9594834	9710831	36535404	36600430	+	#FAB28C	115997	65026	4279		4279	120276	false
CPOR 16	9763159	10130232	4489376	4721253	+	#98C405	367073	231877	850042	84914	850042	1217115	false
CPOR 16	10215146	10310482	4802694	4863893	+	#98C405	95336	61199	84914	88750	84914	180250	false
CPOR 16	10399232	15665498	5290783	10019244	+	#98C405	5266266	4728461	88750	14832	88750	5355016	false
CPOR 16	15680330	15851096	10152045	10270577	+	#98C405	170766	118532	14832	-120523	14832	185598	false
CPOR 16	15730573	20745871	10318686	14836922	+	#98C405	5015298	4518236	-120523		-120523	4894775	false
CPOR 30	20748954	25580287	64	4307867	+	#007FFF	4831333	4307803		55880			
CPOR 30	25636167	35391791	4323905	12732083	+	#007FFF	9755624	8408178	55880	74814	55880	9811504	false
CPOR 30	35466605	40007948	12801462	16748472	+	#007FFF	4541343	3947010	74814	38491	74814	4616157	false
CPOR 30	40046439	43434335	16808734	19960963	+	#007FFF	3387896	3152229	38491	88639	38491	3426387	false
CPOR 30	43522974	44495991	20012870	20837078	+	#007FFF	973017	824208	88639	64534	88639	1061656	false
CPOR 30	44560525	49083563	20905818	24914214	+	#007FFF	4523038	4008396	64534	2730908	64534	4587572	false
CPOR 30	51814471	58297847	24924212	30981486	+	#007FFF	6483376	6057274	2730908	92760	2730908	9214284	false
CPOR 30	58390607	63032849	30981521	35191485	+	#007FFF	4642242	4209964	92760	50718	92760	4735002	false
CPOR 30	63083567	64248679	35192743	36151272	+	#007FFF	1165112	958529	50718	0	50718	1215830	false
CPOR 30	64248679	66443696	36227908	38224770	+	#007FFF	2195017	1996862	0	17	0	2195017	false
CPOR 30	66443713	66517863	38224770	38283322	-	#007FFF	74150	58552	17	1112	17	74167	false
CPOR 30	66518975	67402987	38284915	39202511	+	#007FFF	884012	917596	1112	63957	1112	885124	false
CPOR 30	67466944	68273573	39202644	39948332	+	#007FFF	806629	745688	63957	36812	63957	870586	false
CPOR 30	68310385	68503309	39956058	40084394	-	#007FFF	192924	128336	36812	3900	36812	229736	false
CPOR 30	68507209	68587297	40466122	40528724	-	#007FFF	80088	62602	3900	-80041	3900	83988	false
CPOR 30	68507256	68596659	40584028	40641103	-	#007FFF	89403	57075	-80041	-89403	-80041	9362	false
CPOR 30	68507256	68719318	40584028	40705125	-	#007FFF	212062	121097	-89403	-201913	-89403	122659	false
CPOR 30	68517405	68727649	40670035	40815793	-	#007FFF	210244	145758	-201913	-159395	-201913	8331	false
CPOR 30	68568254	68727649	40738535	40815784	-	#007FFF	159395	77249	-159395	103744	-159395	0	true

	CPOR 30	68831393	68898760	40647025	40756781	-	#007FFF	67367	109756	103744	-67367	103744	171111	false
	CPOR 30	68831393	68900992	40610123	40673222	-	#007FFF	69599	63099	-67367	-68809	-67367	2232	false
	CPOR 30	68832183	68907336	40500195	40584248	-	#007FFF	75153	84053	-68809	-32036	-68809	6344	false
	CPOR 30	68875300	68976048	41077440	41135677	+	#007FFF	100748	58237	-32036	51354	-32036	68712	false
	CPOR 30	69027402	69135515	40682830	40796225	+	#007FFF	108113	113395	51354	-108113	51354	159467	false
	CPOR 30	69027402	69138303	40466969	40584248	+	#007FFF	110901	117279	-108113	-110241	-108113	2788	false
	CPOR 30	69028062	69141406	40636242	40758152	+	#007FFF	113344	121910	-110241	-108960	-110241	3103	false
	CPOR 30	69032446	69141406	40611407	40674582	+	#007FFF	108960	63175	-108960	205031	-108960	0	true
	CPOR 30	69346437	69443453	41077445	41142867	-	#007FFF	97016	65422	205031	-55163	205031	302047	false
	CPOR 30	69388290	69446706	40681559	40758152	+	#007FFF	58416	76593	-55163	-56985	-55163	3253	false
	CPOR 30	69389721	69495631	40775828	40854241	-	#007FFF	105910	78413	-56985	-95698	-56985	48925	false
	CPOR 30	69399933	69494246	40326024	40414788	+	#007FFF	94313	88764	-95698	-36364	-95698	-1385	true
	CPOR 30	69457882	69518561	41085376	41142862	+	#007FFF	60679	57486	-36364	69127	-36364	24315	false
	CPOR 30	69587688	69657608	41385120	41460987	+	#007FFF	69920	75867	69127	41545	69127	139047	false
	CPOR 30	69699153	70403122	41528710	42017499	+	#007FFF	703969	488789	41545	59335	41545	745514	false
	CPOR 30	70462457	71879149	42017499	43337117	+	#007FFF	1416692	1319618	59335	59963	59335	1476027	false
	CPOR 30	71939112	73650509	43337684	45052184	+	#007FFF	1711397	1714500	59963		59963	1771360	false
	CPOR 2	73671859	73926154	32767307	33005050	+	#766A7A	254295	237743		1497			
	CPOR 2	73927651	74024202	32854985	32928829	-	#766A7A	96551	73844	1497	133787	1497	98048	false
	CPOR 2	74157989	75567434	33049182	34122097	+	#766A7A	1409445	1072915	133787		133787	1543232	false
	CPOR 3	75897187	77293063	60449342	61847139	-	#13C840	1395876	1397797		127595			
	CPOR 3	77420658	82923099	54886720	60273895	-	#13C840	5502441	5387175	127595	5100636	127595	5630036	false
	CPOR 6	82923215	83485311	4912364	5297415	-	#0C404C	562096	385051	7018513	33223	7018513	7580609	false
	CPOR 6	83518534	88078355	660577	4849443	-	#0C404C	4559821	4188866	33223		33223	4593044	false
	CPOR 3	88023735	88141016	63370512	63489123	+	#13C840	117281	118611	5100636	-107273	5100636	5217917	false
	CPOR 3	88033743	98811188	63559677	73921257	+	#13C840	10777445	10361580	-107273	-10773521	-107273	10670172	false
	CPOR 3	88037667	88145580	62717774	62813248	+	#13C840	107913	95474	-10773521	-107913	-10773521	-10665608	true
	CPOR 3	88037667	88145604	62815519	62895701	+	#13C840	107937	80182	-107913	-96414	-107913	24	false
	CPOR 3	88049190	88151139	62416643	62537716	-	#13C840	101949	121073	-96414	-64312	-96414	5535	false
	CPOR 3	88086827	88145604	62178276	62235960	-	#13C840	58777	57684	-64312	-53395	-64312	-5535	true
	CPOR 3	88092209	88151284	62672684	62734644	+	#13C840	59075	61960	-53395	252768	-53395	5680	false
	CPOR 3	88404052	88501114	63905826	63982725	+	#13C840	97062	76899	252768	10332392	252768	349830	false

	CPOR 13	99221303	99322847	76033570	76083955	-	#7F00FF	101544	50385		-46344			
	CPOR 13	99276503	112222270	66100130	76050087	-	#7F00FF	12945767	9949957	-46344	-8972956	-46344	12899423	false
	CPOR 13	103249314	103330997	65925081	65993663	+	#7F00FF	81683	68582	-8972956	8892287	-8972956	-8891273	true
	CPOR 13	112223284	113405763	65057142	65924801	-	#7F00FF	1182479	867659	8892287	39941	8892287	10074766	false
	CPOR 13	113445704	116192442	62459771	64844556	-	#7F00FF	2746738	2384785	39941	0	39941	2786679	false
	CPOR 13	116192442	116423421	62260460	62455478	+	#7F00FF	230979	195018	0	0	0	230979	false
	CPOR 13	116423421	118417944	60507780	62256325	-	#7F00FF	1994523	1748545	0	255270	0	1994523	false
	CPOR 13	118673214	119331286	59900630	60507433	-	#7F00FF	658072	606803	255270	140052	255270	913342	false
	CPOR 13	119471338	119800687	59605018	59898810	-	#7F00FF	329349	293792	140052	0	140052	469401	false
	CPOR 13	119800687	140617918	41634098	59516393	-	#7F00FF	20817231	17882295	0	1689	0	20817231	false
	CPOR 13	140619607	143555023	35782493	38277727	+	#7F00FF	2935416	2495234	1689	19827	1689	2937105	false
	CPOR 13	143574850	143908830	38415032	38592056	+	#7F00FF	333980	177024	19827	35953	19827	353807	false
	CPOR 13	143944783	144022694	38523615	38592149	+	#7F00FF	77911	68534	35953	32033	35953	113864	false
	CPOR 13	144054727	147695241	38523615	41634098	+	#7F00FF	3640514	3110483	32033	82	32033	3672547	false
	CPOR 13	147695323	153716604	30414436	35738125	-	#7F00FF	6021281	5323689	82	1130	82	6021363	false
	CPOR 13	153717734	154550904	29531241	30307573	-	#7F00FF	833170	776332	1130	0	1130	834300	false
	CPOR 13	154550904	154622851	29465904	29531195	+	#7F00FF	71947	65291	0	36633	0	71947	false
	CPOR 13	154659484	154762685	38298618	38393562	+	#7F00FF	103201	94944	36633	16219	36633	139834	false
	CPOR 13	154778904	158672588	26222840	29454795	+	#7F00FF	3893684	3231955	16219	-2581	16219	3909903	false
	CPOR 13	158670007	159502610	25567482	26168557	-	#7F00FF	832603	601075	-2581	-189004	-2581	830022	false
	CPOR 13	159313606	159502677	25411712	25538177	-	#7F00FF	189071	126465	-189004	-46149	-189004	67	false
	CPOR 13	159456528	160431100	24031716	24769795	+	#7F00FF	974572	738079	-46149	-974571	-46149	928423	false
	CPOR 13	159456529	161903596	20291663	22116104	-	#7F00FF	2447067	1824441	-974571	-2447067	-974571	1472496	false
	CPOR 13	159456529	162184448	18039503	20151052	-	#7F00FF	2727919	2111549	-2447067	-722185	-2447067	280852	false
	CPOR 13	161462263	161651326	25035497	25145609	+	#7F00FF	189063	110112	-722185	305943	-722185	-533122	true
	CPOR 13	161957269	162117705	20156094	20279184	-	#7F00FF	160436	123090	305943		305943	466379	false
	CPOR 26	162585130	164496606	25544846	27110385	+	#F50241	1911476	1565539		0			
	CPOR 26	164496606	164632174	27110888	27229935	-	#F50241	135568	119047	0	4054	0	135568	false
	CPOR 26	164636228	164958677	27230061	27466741	+	#F50241	322449	236680	4054	72628	4054	326503	false
	CPOR 26	165031305	167995182	27457947	29862903	+	#F50241	2963877	2404956	72628	-472237	72628	3036505	false
	CPOR 26	167522945	167992807	29854001	30204004	-	#F50241	469862	350003	-472237	2382	-472237	-2375	true
	CPOR 26	167995189	168100014	30592160	30674736	-	#F50241	104825	82576	2382	-7513	2382	107207	false

	CPOR 26	168092501	168337213	30791675	30964775	+	#F50241	244712	173100	-7513	20646	-7513	237199	false
	CPOR 26	168357859	170162539	30873535	32338774	+	#F50241	1804680	1465239	20646	-100567	20646	1825326	false
	CPOR 26	170061972	174996264	32358805	36404037	+	#F50241	4934292	4045232	-100567	148058	-100567	4833725	false
	CPOR 26	175144322	175326176	36405882	36502998	+	#F50241	181854	97116	148058	59726	148058	329912	false
	CPOR 26	175385902	175554619	36512727	36571680	+	#F50241	168717	58953	59726	-18360	59726	228443	false
	CPOR 26	175536259	178200566	36661552	38938550	+	#F50241	2664307	2276998	-18360	6372	-18360	2645947	false
	CPOR 26	178206938	178375665	38938550	39093897	-	#F50241	168727	155347	6372	12285	6372	175099	false
	CPOR 26	178387950	189633532	39095760	49154690	+	#F50241	11245582	10058930	12285	67601	12285	11257867	false
	CPOR 26	189701133	189915207	49202477	49428813	+	#F50241	214074	226336	67601		67601	281675	false
**HSA 5**	CPOR 10	189445	769994	81939076	82402609	-	#6A78D4	580549	463533		10			
	CPOR 10	770004	1551887	81612598	82004353	-	#6A78D4	781883	391755	10	98372	10	781893	false
	CPOR 10	1650259	6509306	78250061	81612000	-	#6A78D4	4859047	3361939	98372	57699	98372	4957419	false
	CPOR 10	6567005	8081296	77012863	78249396	-	#6A78D4	1514291	1236533	57699	-155746	57699	1571990	false
	CPOR 10	7925550	8082584	76206323	76390865	+	#6A78D4	157034	184542	-155746	4264	-155746	1288	false
	CPOR 10	8086848	9630680	75609125	76950057	-	#6A78D4	1543832	1340932	4264	-1522120	4264	1548096	false
	CPOR 10	8108560	8159754	76941122	77012807	+	#6A78D4	51194	71685	-1522120	17832	-1522120	-1470926	true
	CPOR 10	8177586	8291854	76491506	76608227	+	#6A78D4	114268	116721	17832	63699	17832	132100	false
	CPOR 10	8355553	8411632	76087373	76167125	-	#6A78D4	56079	79752	63699	83904	63699	119778	false
	CPOR 10	8495536	8642512	76768458	76913194	+	#6A78D4	146976	144736	83904	129733	83904	230880	false
	CPOR 10	8772245	8961835	76995574	77108215	+	#6A78D4	189590	112641	129733	136137	129733	319323	false
	CPOR 10	9097972	9190932	76687501	76756457	-	#6A78D4	92960	68956	136137	435792	136137	229097	false
	CPOR 10	9626724	12813648	72885261	75603648	-	#6A78D4	3186924	2718387	435792	24	435792	3622716	false
	CPOR 10	12813672	17529960	68775366	72819730	-	#6A78D4	4716288	4044364	24	98573	24	4716312	false
	CPOR 10	17628533	19099192	67452716	68780999	-	#6A78D4	1470659	1328283	98573	51882	98573	1569232	false
	CPOR 10	19151074	20793187	66089415	67414133	-	#6A78D4	1642113	1324718	51882	130612	51882	1693995	false
	CPOR 10	20923799	21453104	65639185	66018366	-	#6A78D4	529305	379181	130612	91999	130612	659917	false
	CPOR 10	21545103	21652543	65534165	65624258	-	#6A78D4	107440	90093	91999	-107440	91999	199439	false
	CPOR 10	21545103	22142396	64198651	64699667	-	#6A78D4	597293	501016	-107440	64827	-107440	489853	false
	CPOR 10	22207223	29401685	58264458	64191970	-	#6A78D4	7194462	5927512	64827	54369	64827	7259289	false
	CPOR 10	29456054	34060253	54296336	58264371	-	#6A78D4	4604199	3968035	54369	8481	54369	4658568	false
	CPOR 10	34068734	34173691	65159457	65267777	+	#6A78D4	104957	108320	8481	24649	8481	113438	false
	CPOR 10	34198340	34263815	64751686	64829203	+	#6A78D4	65475	77517	24649	144426	24649	90124	false

	CPOR 10	34408241	42037338	47557010	54295973	-	#6A78D4	7629097	6738963	144426	4022	144426	7773523	false
	CPOR 10	42041360	42882543	46813048	47493889	-	#6A78D4	841183	680841	4022	221116	4022	845205	false
	CPOR 10	43103659	45911317	44305302	46634539	-	#6A78D4	2807658	2329237	221116	4364241	221116	3028774	false
	CPOR 10	50275558	50485276	43995337	44255408	-	#6A78D4	209718	260071	4364241	52957	4364241	4573959	false
	CPOR 10	50538233	55718204	39156487	43961323	-	#6A78D4	5179971	4804836	52957	5684	52957	5232928	false
	CPOR 10	55723888	60964859	34126045	38904800	-	#6A78D4	5240971	4778755	5684	-180491	5684	5246655	false
	CPOR 10	60784368	64383466	30558102	34004172	-	#6A78D4	3599098	3446070	-180491	17886	-180491	3418607	false
	CPOR 10	64401352	65748787	29116711	30380166	-	#6A78D4	1347435	1263455	17886	-30849	17886	1365321	false
	CPOR 10	65717938	69207167	25782720	29046445	-	#6A78D4	3489229	3263725	-30849	10129	-30849	3458380	false
	CPOR 10	69217296	69557336	25156879	25479170	-	#6A78D4	340040	322291	10129	101826	10129	350169	false
	CPOR 10	69659162	69773097	64751686	64856365	+	#6A78D4	113935	104679	101826	16880	101826	215761	false
	CPOR 10	69789977	69894041	64751686	64852261	-	#6A78D4	104064	100575	16880	131159	16880	120944	false
	CPOR 10	70025200	70128504	24990420	25062830	+	#6A78D4	103304	72410	131159	110399	131159	234463	false
	CPOR 10	70238903	70352942	64751686	64856365	+	#6A78D4	114039	104679	110399	182345	110399	224438	false
	CPOR 10	70535287	70640256	64751686	64852261	+	#6A78D4	104969	100575	182345	16862	182345	287314	false
	CPOR 10	70657118	70769923	64751686	64856365	-	#6A78D4	112805	104679	16862	130695	16862	129667	false
	CPOR 10	70900618	71067130	24990420	25096741	+	#6A78D4	166512	106321	130695	172115	130695	297207	false
	CPOR 10	71239245	71352369	64751686	64856365	+	#6A78D4	113124	104679	172115	98145	172115	285239	false
	CPOR 10	71450514	72717095	23701703	24990419	-	#6A78D4	1266581	1288716	98145	-4803	98145	1364726	false
	CPOR 10	72712292	74929418	21588847	23701695	-	#6A78D4	2217126	2112848	-4803	87112	-4803	2212323	false
	CPOR 10	75016530	75279458	19581613	19933831	+	#6A78D4	262928	352218	87112	35300	87112	350040	false
	CPOR 10	75314758	76976918	19984323	21514135	+	#6A78D4	1662160	1529812	35300	43462	35300	1697460	false
	CPOR 10	77020380	78346120	18180421	19491953	-	#6A78D4	1325740	1311532	43462	8507	43462	1369202	false
	CPOR 10	78354627	79134762	17245244	18011603	-	#6A78D4	780135	766359	8507	8768	8507	788642	false
	CPOR 10	79143530	79998086	16265989	17173804	-	#6A78D4	854556	907815	8768	0	8768	863324	false
	CPOR 10	79998086	80084130	16063387	16198781	-	#6A78D4	86044	135394	0		0	86044	false
	CPOR 2	80116042	91193899	19647769	28424237	-	#766A7A	11077857	8776468		59359			
	CPOR 2	91253258	99236845	12654640	19647769	-	#766A7A	7983587	6993129	59359	780	59359	8042946	false
	CPOR 2	99237625	99467738	12359414	12599420	-	#766A7A	230113	240006	780	114845	780	230893	false
	CPOR 2	99582583	100374684	11715688	12429616	+	#766A7A	792101	713928	114845	27302	114845	906946	false
	CPOR 2	100401986	100468206	11549776	11620018	-	#766A7A	66220	70242	27302	66030	27302	93522	false
	CPOR 2	100534236	100996474	11120658	11485168	-	#766A7A	462238	364510	66030	72135	66030	528268	false

CPOR 2	101068609	102055469	10269182	11076621	-	#766A7A	986860	807439	72135	-6197	72135	1058995	false
CPOR 2	102049272	102120233	10565682	10632913	-	#766A7A	70961	67231	-6197	-32882	-6197	64764	false
CPOR 2	102087351	102389145	157227	382040	+	#766A7A	301794	224813	-32882	-258407	-32882	268912	false
CPOR 2	102130738	107849966	382562	5446250	+	#766A7A	5719228	5063688	-258407	1294701	-258407	5460821	false
CPOR 22	107846821	108125720	25153681	25391765	-	#F7FE2D	278899	238084		54			
CPOR 22	108125774	109228131	25392189	26335048	-	#F7FE2D	1102357	942859	54		54	1102411	false
CPOR 2	109144667	110898309	5447184	6987765	+	#766A7A	1753642	1540581	1294701	19447	1294701	3048343	false
CPOR 2	110917756	112940839	7041191	8870339	+	#766A7A	2023083	1829148	19447	24948449	19447	2042530	false
CPOR 9	112940839	117709773	34263286	39066354	+	#C2FB8F	4768934	4803068		14100			
CPOR 9	117723873	117957997	39078529	39292252	-	#C2FB8F	234124	213723	14100	521	14100	248224	false
CPOR 9	117958518	119636394	39292281	41003651	+	#C2FB8F	1677876	1711370	521	12916	521	1678397	false
CPOR 9	119649310	119864423	41023247	41224277	-	#C2FB8F	215113	201030	12916	567	12916	228029	false
CPOR 9	119864990	120374635	41224277	41752972	-	#C2FB8F	509645	528695	567	448	567	510212	false
CPOR 9	120375083	120921505	41761868	42377458	+	#C2FB8F	546422	615590	448	10215953	448	546870	false
CPOR 19	121849424	122720613	21130933	21918254	-	#8C5319	871189	787321		53			
CPOR 19	122720666	131070339	12924255	21077608	-	#8C5319	8349673	8153353	53	7117833	53	8349726	false
CPOR 9	131137458	134735661	3129752	6254649	+	#C2FB8F	3598203	3124897	10215953	-22017	10215953	13814156	false
CPOR 9	134713644	135730270	31243787	32201979	+	#C2FB8F	1016626	958192	-22017	82986	-22017	994609	false
CPOR 9	135813256	137889254	32202112	34263280	+	#C2FB8F	2075998	2061168	82986	13111597	82986	2158984	false
CPOR 19	138188172	140728751	677957	3008535	+	#8C5319	2540579	2330578	7117833	35031	7117833	9658412	false
CPOR 19	140763782	145713965	3104938	7852499	+	#8C5319	4950183	4747561	35031	17258	35031	4985214	false
CPOR 19	145731223	150809278	7912778	12655975	+	#8C5319	5078055	4743197	17258	101146	17258	5095313	false
CPOR 19	150910424	150986473	12852889	12924115	+	#8C5319	76049	71226	101146		101146	177195	false
CPOR 9	151000851	151354939	2849809	3129643	-	#C2FB8F	354088	279834	13111597	40288	13111597	13465685	false
CPOR 9	151395227	152456968	6332260	7281990	+	#C2FB8F	1061741	949730	40288	-68922	40288	1102029	false
CPOR 9	152388046	157089854	7286280	11531408	+	#C2FB8F	4701808	4245128	-68922	-157399	-68922	4632886	false
CPOR 9	156932455	171400992	12080460	25160362	+	#C2FB8F	14468537	13079902	-157399	-14397820	-157399	14311138	false
CPOR 9	157003172	157104389	11918426	12040747	-	#C2FB8F	101217	122321	-14397820	14230391	-14397820	-14296603	true
CPOR 9	171334780	175999482	25163598	29669187	+	#C2FB8F	4664702	4505589	14230391	292352	14230391	18895093	false
CPOR 9	176291834	177613302	29790006	31168400	+	#C2FB8F	1321468	1378394	292352	231009	292352	1613820	false
CPOR 9	177844311	177934273	29611974	29669187	-	#C2FB8F	89962	57213	231009	130249	231009	320971	false
CPOR 9	178064522	178622736	2205936	2580474	-	#C2FB8F	558214	374538	130249	236979	130249	688463	false

	CPOR 9	178859715	179627338	1382217	1966681	-	#C2FB8F	767623	584464	236979	50952	236979	1004602	false
	CPOR 9	179678290	180669574	668075	1376763	-	#C2FB8F	991284	708688	50952		50952	1042236	false
	CPOR 1	180777946	180909452	47546466	47657439	-	#C18B50	131506	110973		278670			
	CPOR 1	181188122	181251982	47394896	47465207	-	#C18B50	63860	70311	278670		278670	342530	false
**HSA 6**	CPOR 20	181356	2935662	3748789	5912724	+	#00FF7F	2754306	2163935		-49056			
	CPOR 20	2886606	5016062	6220186	8003262	+	#00FF7F	2129456	1783076	-49056	50975	-49056	2080400	false
	CPOR 20	5067037	5805913	8029136	8647269	+	#00FF7F	738876	618133	50975	51194	50975	789851	false
	CPOR 20	5857107	26000938	8679296	26178109	+	#00FF7F	20143831	17498813	51194	-3643505	51194	20195025	false
	CPOR 20	22357433	22448101	303617	356314	-	#00FF7F	90668	52697	-3643505	3514185	-3643505	-3552837	true
	CPOR 20	25962286	26436739	26274758	26599024	+	#00FF7F	474453	324266	3514185	-54491	3514185	3988638	false
	CPOR 20	26382248	26664028	27069244	27262690	+	#00FF7F	281780	193446	-54491	359050	-54491	227289	false
	CPOR 20	27023078	27167005	2799619	2868632	-	#00FF7F	143927	69013	359050	253122	359050	502977	false
	CPOR 20	27420127	27620480	2621023	2735931	-	#00FF7F	200353	114908	253122	163138	253122	453475	false
	CPOR 20	27783618	28005009	2440032	2586655	-	#00FF7F	221391	146623	163138	63066	163138	384529	false
	CPOR 20	28068075	28366930	2277815	2434504	-	#00FF7F	298855	156689	63066	63799	63066	361921	false
	CPOR 20	28430729	28593341	2157949	2257480	-	#00FF7F	162612	99531	63799		63799	226411	false
	CPOR 28	29548395	29683416	95584	235088	+	#7FBF7F	135021	139504		204303			
	CPOR 28	29887719	30009752	666153	723268	+	#7FBF7F	122033	57115	204303	-122019	204303	326336	false
	CPOR 28	29887733	30009834	1320946	1396380	-	#7FBF7F	122101	75434	-122019	-103307	-122019	82	false
	CPOR 28	29906527	30009748	1473361	1524096	-	#7FBF7F	103221	50735	-103307	39709	-103307	-86	true
	CPOR 28	30049457	30380673	248083	488392	+	#7FBF7F	331216	240309	39709	61643	39709	370925	false
	CPOR 28	30442316	30863034	738009	1008112	-	#7FBF7F	420718	270103	61643	1025	61643	482361	false
	CPOR 28	30864059	30988418	1167248	1240615	+	#7FBF7F	124359	73367	1025	46248	1025	125384	false
	CPOR 28	31034666	31181375	1230853	1310409	+	#7FBF7F	146709	79556	46248	341633	46248	192957	false
	CPOR 28	31523008	32230431	1520235	2104364	+	#7FBF7F	707423	584129	341633	60016	341633	1049056	false
	CPOR 28	32290447	32373819	2481470	2579547	+	#7FBF7F	83372	98077	60016	7034	60016	143388	false
	CPOR 28	32380853	32593637	3614814	3704875	+	#7FBF7F	212784	90061	7034	-75735	7034	219818	false
	CPOR 28	32517902	32795288	3686949	3975079	+	#7FBF7F	277386	288130	-75735	-49194	-75735	201651	false
	CPOR 28	32746094	33144085	3974192	4311794	+	#7FBF7F	397991	337602	-49194	2815	-49194	348797	false
	CPOR 28	33146900	34155072	4569587	5271538	+	#7FBF7F	1008172	701951	2815	70003	2815	1010987	false
	CPOR 28	34225075	36559381	5281176	6922672	+	#7FBF7F	2334306	1641496	70003	-4953	70003	2404309	false
	CPOR 28	36554428	39335376	6946093	8916029	-	#7FBF7F	2780948	1969936	-4953	-19621	-4953	2775995	false

	CPOR 28	39315755	48795326	8921022	15123698	+	#7FBF7F	9479571	6202676	-19621	89945	-19621	9459950	false
	CPOR 28	48885271	49050869	15124007	15191709	+	#7FBF7F	165598	67702	89945		89945	255543	false
	CPOR 15	49596597	49790895	19519132	19686506	+	#097F8D	194298	167374		-65510			
	CPOR 15	49725385	49830712	19697894	19752004	+	#097F8D	105327	54110	-65510	-105327	-65510	39817	false
	CPOR 15	49725385	52603202	19803126	22698720	+	#097F8D	2877817	2895594	-105327	0	-105327	2772490	false
	CPOR 15	52603202	52688278	22806193	22920876	+	#097F8D	85076	114683	0	51053	0	85076	false
	CPOR 15	52739331	52814346	23224625	23278039	-	#097F8D	75015	53414	51053	-70566	51053	126068	false
	CPOR 15	52743780	52910338	23300429	23352751	+	#097F8D	166558	52322	-70566	-160049	-70566	95992	false
	CPOR 15	52750289	52932556	23175966	23278039	-	#097F8D	182267	102073	-160049	-175304	-160049	22218	false
	CPOR 15	52757252	52933893	23164762	23254633	+	#097F8D	176641	89871	-175304	27167493	-175304	1337	false
	CPOR 7	52942853	56352609	22428012	25493079	+	#2DCDC1	3409756	3065067		43521821			
	CPOR 12	56355395	57092928	2121928	2774485	+	#00007F	737533	652557		-43450			
	CPOR 12	57049478	57436697	2774349	3168694	+	#00007F	387219	394345	-43450	94776	-43450	343769	false
	CPOR 12	57531473	57663378	3246157	3405127	+	#00007F	131905	158970	94776	303675	94776	226681	false
	CPOR 12	57967053	58042502	3594799	3711108	+	#00007F	75449	116309	303675	11364	303675	379124	false
	CPOR 12	58053866	58110659	3744239	3816877	-	#00007F	56793	72638	11364	152322	11364	68157	false
	CPOR 12	58262981	58315846	4114287	4180937	+	#00007F	52865	66650	152322	1925017	152322	205187	false
	CPOR 12	60240863	60329275	3062771	3162486	+	#00007F	88412	99715	1925017	95154	1925017	2013429	false
	CPOR 12	60424429	60562216	3246157	3405127	+	#00007F	137787	158970	95154	51598	95154	232941	false
	CPOR 12	60613814	60793721	3474755	3710853	+	#00007F	179907	236098	51598	14601	51598	231505	false
	CPOR 12	60808322	60861935	3742189	3816877	-	#00007F	53613	74688	14601	272803	14601	68214	false
	CPOR 12	61134738	61267388	4060150	4180937	-	#00007F	132650	120787	272803	270583	272803	405453	false
	CPOR 12	61537971	62834249	4180937	5490317	+	#00007F	1296278	1309380	270583	53361	270583	1566861	false
	CPOR 12	62887610	62984060	5490426	5575887	+	#00007F	96450	85461	53361	16247	53361	149811	false
	CPOR 12	63000307	63857271	5645824	6382783	+	#00007F	856964	736959	16247		16247	873211	false
	CPOR 17	63872374	67843072	164956	3620936	+	#FAB28C	3970698	3455980		87584			
	CPOR 17	67930656	73246969	3658007	8490018	+	#FAB28C	5316313	4832011	87584	145068	87584	5403897	false
	CPOR 17	73392037	78728131	9937651	14958805	-	#FAB28C	5336094	5021154	145068	105500	145068	5481162	false
	CPOR 17	78833631	80148526	8654265	9930649	-	#FAB28C	1314895	1276384	105500		105500	1420395	false
	CPOR 15	80101386	84591076	15478673	19469629	-	#097F8D	4489690	3990956	27167493	50481	27167493	31657183	false
	CPOR 15	84641557	85757319	14287693	15444943	-	#097F8D	1115762	1157250	50481	129678	50481	1166243	false
	CPOR 15	85886997	85968412	14165269	14287678	-	#097F8D	81415	122409	129678	148966	129678	211093	false

CPOR 15	86117378	86364133	13854998	14047837	-	#097F8D	246755	192839	148966	218	148966	395721	false
CPOR 15	86364351	86445708	13598048	13696764	+	#097F8D	81357	98716	218	79817	218	81575	false
CPOR 15	86525525	86728083	13700716	13840906	+	#097F8D	202558	140190	79817	0	79817	282375	false
CPOR 15	86728083	89821309	10549500	13594252	-	#097F8D	3093226	3044752	0	60341	0	3093226	false
CPOR 15	89881650	95020635	5563101	10474513	-	#097F8D	5138985	4911412	60341	54249	60341	5199326	false
CPOR 15	95074884	99685500	753522	5563053	-	#097F8D	4610616	4809531	54249	22503	54249	4664865	false
CPOR 15	99708003	99870796	401966	688827	-	#097F8D	162793	286861	22503	24119930	22503	185296	false
CPOR 7	99874430	102948672	348	3081940	+	#2DCDC1	3074242	3081592	43521821	37492	43521821	46596063	false
CPOR 7	102986164	103048815	3135796	3209435	-	#2DCDC1	62651	73639	37492	45306	37492	100143	false
CPOR 7	103094121	109545058	3209668	9025415	+	#2DCDC1	6450937	5815747	45306	108577	45306	6496243	false
CPOR 7	109653635	110424691	9025505	9739800	+	#2DCDC1	771056	714295	108577	49766	108577	879633	false
CPOR 7	110474457	110950072	14166857	14624783	+	#2DCDC1	475615	457926	49766	4882	49766	525381	false
CPOR 7	110954954	111030728	14675181	14754569	+	#2DCDC1	75774	79388	4882	26322	4882	80656	false
CPOR 7	111057050	111268499	14805096	15015382	+	#2DCDC1	211449	210286	26322	-10200	26322	237771	false
CPOR 7	111258299	112112155	15185756	15948782	+	#2DCDC1	853856	763026	-10200	-68063	-10200	843656	false
CPOR 7	112044092	112555039	15949704	16428000	+	#2DCDC1	510947	478296	-68063	171	-68063	442884	false
CPOR 7	112555210	112685325	16434840	16549939	-	#2DCDC1	130115	115099	171	1706	171	130286	false
CPOR 7	112687031	114839053	16549939	18555687	+	#2DCDC1	2152022	2005748	1706	2155	1706	2153728	false
CPOR 7	114841208	114955305	18555795	18634281	-	#2DCDC1	114097	78486	2155	5489	2155	116252	false
CPOR 7	114960794	116593592	18639325	20269640	+	#2DCDC1	1632798	1630315	5489	51557369	5489	1638287	false
CPOR 26	116614588	116673663	8974490	9037991	+	#F50241	59075	63501		0			
CPOR 26	116673663	118345336	7651128	8969958	-	#F50241	1671673	1318830	0	-31469	0	1671673	false
CPOR 26	118313867	121619508	229598	2568124	+	#F50241	3305641	2338526	-31469	56963	-31469	3274172	false
CPOR 26	121676471	124873081	2601104	5248612	+	#F50241	3196610	2647508	56963	3	56963	3253573	false
CPOR 26	124873084	127403458	5273465	7353818	-	#F50241	2530374	2080353	3	10245537	3	2530377	false
CPOR 15	127269648	127442040	50978404	51107320	-	#097F8D	172392	128916	3200496	-172392	3200496	3372888	false
CPOR 15	127269648	131915202	45867256	49983071	+	#097F8D	4645554	4115815	-172392	22	-172392	4473162	false
CPOR 15	131915224	132571106	50044102	50651710	+	#097F8D	655882	607608	22	-9638	22	655904	false
CPOR 15	132561468	133550122	51356912	52305740	+	#097F8D	988654	948828	-9638	894	-9638	979016	false
CPOR 15	133551016	137648909	34001999	37454539	-	#097F8D	4097893	3452540	894	11172	894	4098787	false
CPOR 15	137660081	146859923	52309660	60319301	-	#097F8D	9199842	8009641	11172	-272076	11172	9211014	false
CPOR 15	146587847	149922269	30480384	33442320	+	#097F8D	3334422	2961936	-272076	77611	-272076	3062346	false

	CPOR 15	149999880	150096305	33412570	33502677	+	#097F8D	96425	90107	77611	-96323	77611	174036	false
	CPOR 15	149999982	150398427	33528204	33910418	+	#097F8D	398445	382214	-96323	6748	-96323	302122	false
	CPOR 15	150405175	159656885	37490334	45784425	-	#097F8D	9251710	8294091	6748	25239	6748	9258458	false
	CPOR 15	159682124	160524411	23381404	24295738	+	#097F8D	842287	914334	25239	7007	25239	867526	false
	CPOR 15	160531418	160665874	24255806	24316212	+	#097F8D	134456	60406	7007	34035	7007	141463	false
	CPOR 15	160699909	160758468	24251841	24320126	-	#097F8D	58559	68285	34035	156057	34035	92594	false
	CPOR 15	160914525	167247669	24295761	29976963	+	#097F8D	6333144	5681202	156057	3302	156057	6489201	false
	CPOR 15	167250971	167393888	29862101	29980523	-	#097F8D	142917	118422	3302	4970	3302	146219	false
	CPOR 15	167398858	167588915	29985272	30134180	+	#097F8D	190057	148908	4970	53122	4970	195027	false
	CPOR 15	167642037	168060722	30134428	30478803	+	#097F8D	418685	344375	53122		53122	471807	false
	CPOR 7	168150961	170581093	20316720	22269423	+	#2DCDC1	2430132	1952703	51557369		51557369	53987501	false
**HSA 7**	CPOR 24	31731	1141300	29747892	30505989	-	#00FFFF	1109569	758097		-6071			
	CPOR 24	1135229	2358128	28594334	29505894	-	#00FFFF	1222899	911560	-6071	-4051	-6071	1216828	false
	CPOR 24	2354077	2531271	24862470	25029271	-	#00FFFF	177194	166801	-4051	0	-4051	173143	false
	CPOR 24	2531271	2671725	27308929	27428447	+	#00FFFF	140454	119518	0	1075	0	140454	false
	CPOR 24	2672800	4964859	25556773	27315942	-	#00FFFF	2292059	1759169	1075	65457	1075	2293134	false
	CPOR 24	5030316	5161137	28312939	28468156	-	#00FFFF	130821	155217	65457	20105	65457	196278	false
	CPOR 24	5181242	5788368	27723026	28216819	+	#00FFFF	607126	493793	20105	197895	20105	627231	false
	CPOR 24	5986263	6202448	23863044	24033900	+	#00FFFF	216185	170856	197895	-105265	197895	414080	false
	CPOR 24	6097183	6730960	24130661	24717455	+	#00FFFF	633777	586794	-105265	-619980	-105265	528512	false
	CPOR 24	6110980	6190056	27637232	27696058	-	#00FFFF	79076	58826	-619980	23461195	-619980	-540904	true
	CPOR 2	7021513	12506036	86670603	91646774	+	#766A7A	5484523	4976171		14545			
	CPOR 2	12520581	22489340	120714822	129379159	-	#766A7A	9968759	8664337	14545	52046	14545	9983304	false
	CPOR 2	22541386	29648663	114362832	120714822	-	#766A7A	7107277	6351990	52046	95448	52046	7159323	false
	CPOR 24	29651251	29742500	33489896	33551928	-	#00FFFF	91249	62032	23461195	2733445	23461195	23552444	false
	CPOR 2	29744111	32480205	111936723	114369556	-	#766A7A	2736094	2432833	95448	15938551	95448	2831542	false
	CPOR 24	32475945	32597071	39841733	39941927	-	#00FFFF	121126	100194	2733445	270479	2733445	2854571	false
	CPOR 24	32867550	33110202	39647849	39841693	-	#00FFFF	242652	193844	270479	18217	270479	513131	false
	CPOR 24	33128419	34154824	38610956	39459024	-	#00FFFF	1026405	848068	18217	-28150	18217	1044622	false
	CPOR 24	34126674	34950485	40446923	41148796	+	#00FFFF	823811	701873	-28150	57173	-28150	795661	false
	CPOR 24	35007658	35076956	41195665	41254148	+	#00FFFF	69298	58483	57173	-25691	57173	126471	false
	CPOR 24	35051265	35185595	33487638	33550207	+	#00FFFF	134330	62569	-25691	-121729	-25691	108639	false

CPOR 24	35063866	35910181	30592639	31319026	+	#00FFFF	846315	726387	-121729	35142	-121729	724586	false
CPOR 24	35945323	38365082	31297313	33426103	+	#00FFFF	2419759	2128790	35142	-134304	35142	2454901	false
CPOR 24	38230778	38368567	33355902	33425174	+	#00FFFF	137789	69272	-134304	-128918	-134304	3485	false
CPOR 24	38239649	39755333	33552401	34877080	+	#00FFFF	1515684	1324679	-128918	128252	-128918	1386766	false
CPOR 24	39883585	43808010	34877635	38446644	+	#00FFFF	3924425	3569009	128252	58301	128252	4052677	false
CPOR 24	43866311	43960172	4305220	4394343	-	#00FFFF	93861	89123	58301	83728	58301	152162	false
CPOR 24	44043900	45282048	3523415	4305197	-	#00FFFF	1238148	781782	83728	76101	83728	1321876	false
CPOR 24	45358149	45724561	3256933	3522828	-	#00FFFF	366412	265895	76101	133682	76101	442513	false
CPOR 24	45858243	47590041	2058755	3256932	-	#00FFFF	1731798	1198177	133682	55601	133682	1865480	false
CPOR 24	47645642	48244277	1731667	2058755	-	#00FFFF	598635	327088	55601	-87488	55601	654236	false
CPOR 24	48156789	51358487	14143015	16330459	-	#00FFFF	3201698	2187444	-87488	-2654007	-87488	3114210	false
CPOR 28	48297709	48381817	48463414	48523970	-	#7FBF7F	84108	60556		0			
CPOR 28	48381817	48672779	48530616	48758319	+	#7FBF7F	290962	227703	0		0	290962	false
CPOR 24	48704480	49600039	16339096	17010997	-	#00FFFF	895559	671901	-2654007	-670588	-2654007	-1758448	true
CPOR 24	48929451	49034667	638953	691328	-	#00FFFF	105216	52375	-670588	2414481	-670588	-565372	true
CPOR 24	51449148	52582804	13345271	14122808	-	#00FFFF	1133656	777537	2414481	-17249	2414481	3548137	false
CPOR 24	52565555	53183412	12274798	12812812	+	#00FFFF	617857	538014	-17249	6012	-17249	600608	false
CPOR 24	53189424	53382814	11565400	11721027	+	#00FFFF	193390	155627	6012	-193390	6012	199402	false
CPOR 24	53189424	53677142	12907317	13244749	-	#00FFFF	487718	337432	-193390	78535	-193390	294328	false
CPOR 24	53755677	53861296	12814216	12868955	-	#00FFFF	105619	54739	78535	11240	78535	184154	false
CPOR 24	53872536	53991977	13246605	13340779	+	#00FFFF	119441	94174	11240	-119441	11240	130681	false
CPOR 24	53872536	54341947	11253457	11563544	-	#00FFFF	469411	310087	-119441	-349970	-119441	349970	false
CPOR 24	53991977	55314860	9953271	10817980	-	#00FFFF	1322883	864709	-349970	12486	-349970	972913	false
CPOR 24	55327346	55586310	17211222	17431445	+	#00FFFF	258964	220223	12486	208975	12486	271450	false
CPOR 24	55795285	56114670	4461336	4630103	+	#00FFFF	319385	168767	208975	12813	208975	528360	false
CPOR 24	56127483	56226019	8050025	8144718	+	#00FFFF	98536	94693	12813	9500812	12813	111349	false
CPOR 24	65726831	65976373	4566778	4747323	+	#00FFFF	249542	180545	9500812	161556	9500812	9750354	false
CPOR 24	66137929	66363875	4790976	4983795	+	#00FFFF	225946	192819	161556	54775	161556	387502	false
CPOR 24	66418650	66469974	5032985	5096606	-	#00FFFF	51324	63621	54775	270273	54775	106099	false
CPOR 24	66740247	66813390	5024845	5096606	+	#00FFFF	73143	71761	270273	105044	270273	343416	false
CPOR 24	66918434	67088460	5109966	5229601	+	#00FFFF	170026	119635	105044	251462	105044	275070	false
CPOR 24	67339922	67406208	423697	485685	+	#00FFFF	66286	61988	251462	13433	251462	317748	false

	CPOR 24	67419641	72605335	5320453	9432522	-	#00FFFF	5185694	4112069	13433	567800	13433	5199127	false
	CPOR 24	73173135	73280569	201263	286524	+	#00FFFF	107434	85261	567800	5874	567800	675234	false
	CPOR 24	73286443	74440615	17541938	18318237	-	#00FFFF	1154172	776299	5874	4068	5874	1160046	false
	CPOR 24	74444683	74852673	32230	300276	+	#00FFFF	407990	268046	4068	70957	4068	412058	false
	CPOR 24	74923630	75219536	201263	388388	-	#00FFFF	295906	187125	70957	310922	70957	366863	false
	CPOR 24	75530458	76070349	18354018	18773058	+	#00FFFF	539891	419040	310922	107668	310922	850813	false
	CPOR 24	76178017	76516704	18773058	19015357	+	#00FFFF	338687	242299	107668	21457679	107668	446355	false
	CPOR 2	77069779	86044390	137651389	144822872	+	#766A7A	8974611	7171483	28554002	945	28554002	37528613	false
	CPOR 2	86045335	86372744	144823056	145021299	-	#766A7A	327409	198243	945	1375	945	328354	false
	CPOR 2	86374119	87445826	145021459	145753126	+	#766A7A	1071707	731667	1375	57331	1375	1073082	false
	CPOR 2	87503157	87615866	76437412	76585157	+	#766A7A	112709	147745	57331	-112051	57331	170040	false
	CPOR 2	87503815	90106200	76601813	79239582	+	#766A7A	2602385	2637769	-112051	56434	-112051	2490334	false
	CPOR 2	90162634	95734353	79331473	84430031	+	#766A7A	5571719	5098558	56434	22422	56434	5628153	false
	CPOR 2	95756775	97873787	84498453	86579440	+	#766A7A	2117012	2080987	22422	4818080	22422	2139434	false
	CPOR 24	97974383	99797319	22228680	23772106	-	#00FFFF	1822936	1543426	21457679	-153954	21457679	23280615	false
	CPOR 24	99643365	99786897	21984760	22047381	-	#00FFFF	143532	62621	-153954	-143532	-153954	-10422	true
	CPOR 24	99643365	99800656	22106964	22200746	+	#00FFFF	157291	93782	-143532	-154512	-143532	13759	false
	CPOR 24	99646144	99867990	21706043	21830069	-	#00FFFF	221846	124026	-154512	-221567	-154512	67334	false
	CPOR 24	99646423	99870427	21706043	21828966	+	#00FFFF	224004	122923	-221567	-189740	-221567	2437	false
	CPOR 24	99680687	99782038	21991526	22087064	-	#00FFFF	101351	95538	-189740	-99094	-189740	-88389	true
	CPOR 24	99682944	99786791	21706043	21765049	+	#00FFFF	103847	59006	-99094	30742	-99094	4753	false
	CPOR 24	99817533	99870427	21989123	22079119	+	#00FFFF	52894	89996	30742	56962	30742	83636	false
	CPOR 24	99927389	99989771	21221527	21306081	-	#00FFFF	62382	84554	56962	-43582	56962	119344	false
	CPOR 24	99946189	100140566	21005399	21227114	-	#00FFFF	194377	221715	-43582	-1991	-43582	150795	false
	CPOR 24	100138575	100221979	20695011	20785481	-	#00FFFF	83404	90470	-1991	116209	-1991	81413	false
	CPOR 24	100338188	102326821	19166542	20732337	-	#00FFFF	1988633	1565795	116209	60873	116209	2104842	false
	CPOR 24	102387694	102526158	19039032	19165274	-	#00FFFF	138464	126242	60873	639805	60873	199337	false
	CPOR 2	102691867	103150919	137413660	137755151	-	#766A7A	459052	341491	4818080	138180	4818080	5277132	false
	CPOR 24	103165963	103279708	33487638	33550207	+	#00FFFF	113745	62569	639805	-97462	639805	753550	false
	CPOR 24	103182246	103288790	30618654	30710246	+	#00FFFF	106544	91592	-97462		-97462	9082	false
	CPOR 2	103289099	112497210	129379237	137379989	-	#766A7A	9208111	8000752	138180	66	138180	9346291	false
	CPOR 2	112497276	118786956	91646791	97569998	+	#766A7A	6289680	5923207	66	-114947	66	6289746	false

	CPOR 2	118672009	118819412	97803231	97965637	-	#766A7A	147403	162406	-114947	26401	-114947	32456	false
	CPOR 2	118845813	119245267	97663948	97992493	+	#766A7A	399454	328545	26401	20	26401	425855	false
	CPOR 2	119245287	119343181	97994057	98080941	-	#766A7A	97894	86884	20	16	20	97914	false
	CPOR 2	119343197	128460230	98083293	106444882	+	#766A7A	9117033	8361589	16	203673	16	9117049	false
	CPOR 2	128663903	134631312	106466536	111827622	+	#766A7A	5967409	5361086	203673		203673	6171082	false
	CPOR 4	134509504	138044555	41703161	45039918	+	#C008AD	3535051	3336757	46891809	21431	46891809	50426860	false
	CPOR 4	138065986	141842118	45159137	48534890	+	#C008AD	3776132	3375753	21431	82393	21431	3797563	false
	CPOR 4	141924511	142099900	48551070	48702507	+	#C008AD	175389	151437	82393	-37978	82393	257782	false
	CPOR 4	142061922	142545337	48720813	49077592	+	#C008AD	483415	356779	-37978	-232802	-37978	445437	false
	CPOR 4	142312535	142581490	49219899	49410310	+	#C008AD	268955	190411	-232802	-261366	-232802	36153	false
	CPOR 4	142320124	142445241	49030417	49141027	+	#C008AD	125117	110610	-261366	-125117	-261366	-136249	true
	CPOR 4	142320124	142581487	49375791	49455826	+	#C008AD	261363	80035	-125117	-261363	-125117	136246	false
	CPOR 4	142320124	142581490	49180560	49268274	+	#C008AD	261366	87714	-261363	-261366	-261363	3	false
	CPOR 4	142320124	142646948	49469510	49640786	+	#C008AD	326824	171276	-261366	-47704	-261366	65458	false
	CPOR 4	142599244	142864560	49640786	49836765	+	#C008AD	265316	195979	-47704	-39501	-47704	217612	false
	CPOR 4	142825059	143015437	49836765	49986625	-	#C008AD	190378	149860	-39501	-122334	-39501	150877	false
	CPOR 4	142893103	143494158	50078990	50702247	+	#C008AD	601055	623257	-122334	-23478	-122334	478721	false
	CPOR 4	143470680	143569308	50719787	50845413	+	#C008AD	98628	125626	-23478	148243	-23478	75150	false
	CPOR 4	143717551	143807701	50797509	50898170	-	#C008AD	90150	100661	148243	10706	148243	238393	false
	CPOR 4	143818407	144158438	50894812	51215095	+	#C008AD	340031	320283	10706	28504	10706	350737	false
	CPOR 4	144186942	144256377	51442389	51564555	-	#C008AD	69435	122166	28504	39811	28504	97939	false
	CPOR 4	144296188	149888344	51443672	55945803	+	#C008AD	5592156	4502131	39811	115966	39811	5631967	false
	CPOR 4	150004310	150068223	58649333	58706681	-	#C008AD	63913	57348	115966	252539	115966	179879	false
	CPOR 4	150320762	152762347	55945803	57673966	+	#C008AD	2441585	1728163	252539	88374	252539	2694124	false
	CPOR 4	152850721	153705056	57721929	58386851	+	#C008AD	854335	664922	88374	115200	88374	942709	false
	CPOR 4	153820256	153957172	58475914	58638265	+	#C008AD	136916	162351	115200	75270	115200	252116	false
	CPOR 4	154032442	154093014	58649333	58706681	+	#C008AD	60572	57348	75270	74254	75270	135842	false
	CPOR 4	154167268	155782655	58753022	59735319	+	#C008AD	1615387	982297	74254	12450	74254	1689641	false
	CPOR 4	155795105	159175861	59843442	61777377	+	#C008AD	3380756	1933935	12450	67718	12450	3393206	false
	CPOR 4	159243579	159331441	61800142	61882269	+	#C008AD	87862	82127	67718		67718	155580	false
**HSA 8**	CPOR 26	352195	1189282	9071624	9647195	+	#F50241	837087	575571		52348			
	CPOR 26	1241630	2241498	9671872	10166913	+	#F50241	999868	495041	52348	260441	52348	1052216	false

	CPOR 26	2501939	5502550	10276594	12571276	+	#F50241	3000611	2294682	260441	7197	260441	3261052	false
	CPOR 26	5509747	5893311	12571552	12894034	-	#F50241	383564	322482	7197	230	7197	390761	false
	CPOR 26	5893541	6878939	12894034	13694933	+	#F50241	985398	800899	230	-10255	230	985628	false
	CPOR 26	6868684	7060496	13760705	13874989	+	#F50241	191812	114284	-10255	320574	-10255	181557	false
	CPOR 17	7082267	7147439	36440641	36514257	-	#FAB28C	65172	73616		17131			
	CPOR 17	7164570	7239333	36437925	36514257	+	#FAB28C	74763	76332	17131	808883	17131	91894	false
	CPOR 26	7381070	7493036	16472417	16616506	-	#F50241	111966	144089	320574	325485	320574	432540	false
	CPOR 26	7818521	7930817	16472417	16616506	+	#F50241	112296	144089	325485	313057	325485	437781	false
	CPOR 17	8048216	8122911	36437925	36514257	-	#FAB28C	74695	76332	808883	4283	808883	883578	false
	CPOR 17	8127194	8241422	36535404	36600430	+	#FAB28C	114228	65026	4283	4178400	4283	118511	false
	CPOR 26	8243874	9787481	54645864	56047302	+	#F50241	1543607	1401438	313057	40615	313057	1856664	false
	CPOR 26	9828096	12102835	14679957	16328838	+	#F50241	2274739	1648881	40615	192308	40615	2315354	false
	CPOR 26	12295143	12349078	16259082	16328838	+	#F50241	53935	69756	192308	372460	192308	246243	false
	CPOR 17	12419822	12538488	36535404	36598244	-	#FAB28C	118666	62840	4178400	84322	4178400	4297066	false
	CPOR 17	12622810	12677088	36443419	36514257	+	#FAB28C	54278	70838	84322		84322	138600	false
	CPOR 26	12721538	18103888	49543790	54636822	-	#F50241	5382350	5093032	372460	29799	372460	5754810	false
	CPOR 26	18133687	18223427	25444363	25530991	-	#F50241	89740	86628	29799	111227	29799	119539	false
	CPOR 26	18334654	18422755	25430680	25500862	-	#F50241	88101	70182	111227	-53444	111227	199328	false
	CPOR 26	18369311	23046467	21414696	25401444	-	#F50241	4677156	3986748	-53444	198711	-53444	4623712	false
	CPOR 26	23245178	27548288	17720430	21344210	-	#F50241	4303110	3623780	198711	-21492	198711	4501821	false
	CPOR 26	27526796	28417797	16970674	17656069	-	#F50241	891001	685395	-21492	-31430	-21492	869509	false
	CPOR 26	28386367	29276267	13902527	14676401	+	#F50241	889900	773874	-31430	50178	-31430	858470	false
	CPOR 26	29326445	30625448	56048200	57427492	+	#F50241	1299003	1379292	50178	9117331	50178	1349181	false
	CPOR 31	30575610	30668862	35778170	35896369	+	#FF00FF	93252	118199		4712			
	CPOR 31	30673574	39106687	36081062	43919796	+	#FF00FF	8433113	7838734	4712	3979	4712	8437825	false
	CPOR 31	39110666	39285629	44325471	44452134	+	#FF00FF	174963	126663	3979	63607	3979	178942	false
	CPOR 31	39349236	39442620	44221224	44325008	+	#FF00FF	93384	103784	63607	3338	63607	156991	false
	CPOR 31	39445958	39523831	44014065	44131826	+	#FF00FF	77873	117761	3338	6851	3338	81211	false
	CPOR 31	39530682	39587751	43984130	44081918	-	#FF00FF	57069	97788	6851	-8130	6851	63920	false
	CPOR 31	39579621	39730204	44695787	44811400	+	#FF00FF	150583	115613	-8130	159415	-8130	142453	false
	CPOR 31	39889619	41452572	44840872	46407294	+	#FF00FF	1562953	1566422	159415	19540	159415	1722368	false
	CPOR 31	41472112	42606951	46465626	47574554	+	#FF00FF	1134839	1108928	19540	9908	19540	1154379	false

CPOR 31	42616859	42802021	47625155	47800885	+	#FF00FF	185162	175730	9908		9908	195070	false
CPOR 2	46544751	46623790	86462867	86585684	+	#766A7A	79039	122817		73084			
CPOR 2	46696874	46770344	86448284	86558073	-	#766A7A	73470	109789	73084		73084	146554	false
CPOR 10	47257053	57010486	2106505	11065470	+	#6A78D4	9753433	8958965		6587			
CPOR 10	57017073	57081916	11065724	11141375	-	#6A78D4	64843	75651	6587	0	6587	71430	false
CPOR 10	57081916	57139701	11147021	11227594	+	#6A78D4	57785	80573	0	309485	0	57785	false
CPOR 10	57449186	58286520	11231533	12013201	+	#6A78D4	837334	781668	309485	3105	309485	1146819	false
CPOR 10	58289625	61790204	12172206	15713048	+	#6A78D4	3500579	3540842	3105		3105	3503684	false
CPOR 12	61852639	62083202	38592607	38849083	-	#00007F	230563	256476		31958			
CPOR 12	62115160	72199638	38253342	48057762	+	#00007F	10084478	9804420	31958	14	31958	10116436	false
CPOR 12	72199652	72286323	47902225	47977691	+	#00007F	86671	75466	14	22941	14	86685	false
CPOR 12	72309264	85062424	48037195	59175095	+	#00007F	12753160	11137900	22941	-44961	22941	12776101	false
CPOR 12	85017463	85076912	59193636	59287883	+	#00007F	59449	94247	-44961	9646	-44961	14488	false
CPOR 12	85086558	85577901	59657138	60091089	+	#00007F	491343	433951	9646	5551	9646	500989	false
CPOR 12	85583452	85636994	60092787	60149111	-	#00007F	53542	56324	5551	192889	5551	59093	false
CPOR 12	85829883	98121265	60149111	70750575	+	#00007F	12291382	10601464	192889	1360	192889	12484271	false
CPOR 12	98122625	101317806	70824178	73451067	+	#00007F	3195181	2626889	1360	755	1360	3196541	false
CPOR 12	101318561	102440209	83213546	84031735	+	#00007F	1121648	818189	755	88036	755	1122403	false
CPOR 12	102528245	102661410	84034095	84105131	+	#00007F	133165	71036	88036	82814	88036	221201	false
CPOR 12	102744224	107563690	84106648	87220528	+	#00007F	4819466	3113880	82814	52393	82814	4902280	false
CPOR 12	107616083	108899524	87220993	88235300	+	#00007F	1283441	1014307	52393	10082	52393	1335834	false
CPOR 12	108909606	113470570	73465408	77312531	+	#00007F	4560964	3847123	10082	55492	10082	4571046	false
CPOR 12	113526062	120051454	77320861	82565021	+	#00007F	6525392	5244160	55492	70854	55492	6580884	false
CPOR 12	120122308	121014928	82565663	83211612	+	#00007F	892620	645949	70854		70854	963474	false
CPOR 9	120986762	121365251	61995756	62334418	-	#C2FB8F	378489	338662		10239			
CPOR 9	121375490	123815951	59933984	61943433	-	#C2FB8F	2440461	2009449	10239	6098	10239	2450700	false
CPOR 9	123822049	129802564	54440901	59761724	-	#C2FB8F	5980515	5320823	6098	-54779	6098	5986613	false
CPOR 9	129747785	131368712	53040456	54410295	-	#C2FB8F	1620927	1369839	-54779	92549	-54779	1566148	false
CPOR 9	131461261	133930031	50874816	53040333	-	#C2FB8F	2468770	2165517	92549	65945	92549	2561319	false
CPOR 9	133995976	142843437	42789355	50818502	-	#C2FB8F	8847461	8029147	65945	30408	65945	8913406	false
CPOR 9	142873845	143045361	63055127	63187365	-	#C2FB8F	171516	132238	30408	-58825	30408	201924	false
CPOR 9	142986536	143886984	63286465	64016915	+	#C2FB8F	900448	730450	-58825	27255	-58825	841623	false

	CPOR 9	143914239	144756069	64087410	64870801	+	#C2FB8F	841830	783391	27255	14635	27255	869085	false
	CPOR 9	144770704	144845817	64915163	64971865	-	#C2FB8F	75113	56702	14635	28965	14635	89748	false
	CPOR 9	144874782	144953666	64983847	65051409	+	#C2FB8F	78884	67562	28965		28965	107849	false
**HSA 9**	CPOR 1	69631	1132296	61582683	62503021	+	#C18B50	1062665	920338		26901			
	CPOR 1	1159197	6286637	62583233	67461608	+	#C18B50	5127440	4878375	26901	4348	26901	5154341	false
	CPOR 1	6290985	6667938	67513872	67865120	+	#C18B50	376953	351248	4348	71444	4348	381301	false
	CPOR 1	6739382	12052627	67937593	72693230	+	#C18B50	5313245	4755637	71444	257	71444	5384689	false
	CPOR 1	12052884	14966243	72745797	75449146	+	#C18B50	2913359	2703349	257	112685	257	2913616	false
	CPOR 1	15078928	21111432	75461035	81169396	+	#C18B50	6032504	5708361	112685	25995	112685	6145189	false
	CPOR 1	21137427	21351893	81772308	81832557	-	#C18B50	214466	60249	25995	50236	25995	240461	false
	CPOR 1	21402129	27299344	81815846	87149263	+	#C18B50	5897215	5333417	50236	-20981	50236	5947451	false
	CPOR 1	27278363	30538920	87176888	90298596	+	#C18B50	3260557	3121708	-20981	65495	-20981	3239576	false
	CPOR 1	30604415	32383067	90348594	91980048	+	#C18B50	1778652	1631454	65495	-41346	65495	1844147	false
	CPOR 1	32341721	33474466	179408106	180593228	-	#C18B50	1132745	1185122	-41346	338863	-41346	1091399	false
	CPOR 1	33813329	36005743	177404328	179406188	-	#C18B50	2192414	2001860	338863	-1120813	338863	2531277	false
	CPOR 1	34884930	34958637	178497419	178599029	-	#C18B50	73707	101610	-1120813	1048258	-1120813	-1047106	true
	CPOR 1	36006895	36173464	177196187	177347851	-	#C18B50	166569	151664	1048258	-4148	1048258	1214827	false
	CPOR 1	36169316	37503920	175882824	177142140	-	#C18B50	1334604	1259316	-4148	6788	-4148	1330456	false
	CPOR 1	37510708	38472134	174841140	175763553	-	#C18B50	961426	922413	6788	1892932	6788	968214	false
	CPOR 11	38648544	39367397	52662726	53171308	-	#6E0025	718853	508582		2439208			
	CPOR 13	39879026	40100914	48952506	49131043	-	#7F00FF	221888	178537		1263683			
	CPOR 8	40225990	40280497	96155091	96229875	+	#60FA36	54507	74784		2574779			
	CPOR 24	40226122	40278825	46464945	46541231	+	#00FFFF	52703	76286		2576583			
	CPOR 1	40365066	40478571	10494726	10591409	+	#C18B50	113505	96683	1892932	536765	1892932	2006437	false
	CPOR 1	41015336	41196458	61533449	61699559	+	#C18B50	181122	166110	536765	1794985	536765	717887	false
	CPOR 13	41364597	41643581	48952506	49149332	+	#7F00FF	278984	196826	1263683	20620351	1263683	1542667	false
	CPOR 11	41806605	42195350	52662726	52900052	-	#6E0025	388745	237326	2439208	18647511	2439208	2827953	false
	CPOR 8	42855276	42909868	96155091	96229903	+	#60FA36	54592	74812	2574779	21164607	2574779	2629371	false
	CPOR 24	42855408	42908162	46464945	46541231	+	#00FFFF	52754	76286	2576583	21166436	2576583	2629337	false
	CPOR 1	42991443	43106904	10494726	10591409	+	#C18B50	115461	96683	1794985	20039486	1794985	1910446	false
	CPOR 11	60842861	60926114	52662726	52719946	-	#6E0025	83253	57220	18647511	192304	18647511	18730764	false
	CPOR 11	61118418	61201738	52662726	52719946	-	#6E0025	83320	57220	192304	86755	192304	275624	false

CPOR 11	61288493	61468904	52682487	52813664	+	#6E0025	180411	131177	86755	546046	86755	267166	false
CPOR 11	62014950	62148001	52927174	53008079	-	#6E0025	133051	80905	546046	4832838	546046	679097	false
CPOR 13	62263932	62371698	49046669	49131043	+	#7F00FF	107766	84374	20620351	1185456	20620351	20728117	false
CPOR 1	63146390	63202891	10531185	10591409	-	#C18B50	56501	60224	20039486	49930	20039486	20095987	false
CPOR 1	63252821	63314107	10526559	10591409	+	#C18B50	61286	64850	49930	1191033	49930	111216	false
CPOR 13	63557154	63698186	48952506	49079110	-	#7F00FF	141032	126604	1185456	2577550	1185456	1326488	false
CPOR 8	64074475	64135048	96155091	96231340	+	#60FA36	60573	76249	21164607	237217	21164607	21225180	false
CPOR 24	64074598	64131786	46464945	46541231	+	#00FFFF	57188	76286	21166436	240611	21166436	21223624	false
CPOR 8	64372265	64426746	96155091	96229903	+	#60FA36	54481	74812	237217	1669062	237217	291698	false
CPOR 24	64372397	64425039	46464945	46541231	+	#00FFFF	52642	76286	240611	1672441	240611	293253	false
CPOR 1	64505140	64618587	10494726	10591409	+	#C18B50	113447	96683	1191033	1050192	1191033	1304480	false
CPOR 1	65668779	65738803	61632676	61699559	-	#C18B50	70024	66883	1050192	158945	1050192	1120216	false
CPOR 1	65897748	66011255	10494726	10591409	-	#C18B50	113507	96683	158945	2223581	158945	272452	false
CPOR 8	66095808	66150128	101819770	101906324	+	#60FA36	54320	86554	1669062	-54320	1669062	1723382	false
CPOR 8	66095808	66150260	96155091	96229875	-	#60FA36	54452	74784	-54320	1711759	-54320	132	false
CPOR 24	66097480	66150128	46464945	46541231	-	#00FFFF	52648	76286	1672441	1712023	1672441	1725089	false
CPOR 13	66275736	66390515	48952506	49057668	+	#7F00FF	114779	105162	2577550	1336597	2577550	2692329	false
CPOR 13	67727112	67831306	49088618	49149332	-	#7F00FF	104194	60714	1336597		1336597	1440791	false
CPOR 8	67862019	67919291	96155091	96232463	+	#60FA36	57272	77372	1711759		1711759	1769031	false
CPOR 24	67862151	67914818	46464945	46541231	+	#00FFFF	52667	76286	1712023		1712023	1764690	false
CPOR 1	68234836	68566842	61367983	61699559	-	#C18B50	332006	331576	2223581	55542	2223581	2555587	false
CPOR 1	68622384	72260346	57932700	61341799	-	#C18B50	3637962	3409099	55542	-39099	55542	3693504	false
CPOR 1	72221247	72955690	57246162	57913431	-	#C18B50	734443	667269	-39099	-84911	-39099	695344	false
CPOR 1	72870779	83195588	47695388	56949970	-	#C18B50	10324809	9254582	-84911	-10311393	-84911	10239898	false
CPOR 1	72884195	73103937	56953107	57100032	-	#C18B50	219742	146925	-10311393	24171366	-10311393	-10091651	true
CPOR 2	83210354	85748005	73361209	76065515	-	#766A7A	2537651	2704306		109436			
CPOR 2	85857441	86102895	73169539	73359348	-	#766A7A	245454	189809	109436	102066	109436	354890	false
CPOR 2	86204961	87409495	71945913	73140010	-	#766A7A	1204534	1194097	102066	6688	102066	1306600	false
CPOR 2	87416183	87488185	71866422	71945913	+	#766A7A	72002	79491	6688	21	6688	78690	false
CPOR 2	87488206	87719406	71654297	71865989	-	#766A7A	231200	211692	21		21	231221	false
CPOR 11	88161679	90185649	54864685	56370556	+	#6E0025	2023970	1505871	20587918	52141	20587918	22611888	false
CPOR 11	90237790	91464196	56378807	57192412	+	#6E0025	1226406	813605	52141	10322	52141	1278547	false

	CPOR 11	91474518	91547027	57203552	57291557	-	#6E0025	72509	88005	10322	7383	10322	82831	false
	CPOR 11	91554410	92122135	57291640	57845428	+	#6E0025	567725	553788	7383	85636	7383	575108	false
	CPOR 11	92207771	92775086	53276584	53770154	+	#6E0025	567315	493570	85636	169504	85636	652951	false
	CPOR 11	92944590	93321320	53784964	54062072	+	#6E0025	376730	277108	169504	101902	169504	546234	false
	CPOR 11	93423222	94011011	54126833	54536288	+	#6E0025	587789	409455	101902	2287	101902	689691	false
	CPOR 11	94013298	94068858	54719966	54776351	+	#6E0025	55560	56385	2287	-4374	2287	57847	false
	CPOR 11	94064484	94214148	54587551	54698862	-	#6E0025	149664	111311	-4374	128186	-4374	145290	false
	CPOR 11	94342334	94461857	52476346	52556986	-	#6E0025	119523	80640	128186	95567	128186	247709	false
	CPOR 11	94557424	96302402	50128455	51761268	+	#6E0025	1744978	1632813	95567	11430	95567	1840545	false
	CPOR 11	96313832	96670546	51923467	52161450	-	#6E0025	356714	237983	11430	89227	11430	368144	false
	CPOR 11	96759773	96939673	52340165	52412030	-	#6E0025	179900	71865	89227	88928	89227	269127	false
	CPOR 11	97028601	97087976	52230030	52293601	-	#6E0025	59375	63571	88928		88928	148303	false
	CPOR 1	97275303	101383202	171218736	174841140	-	#C18B50	4107899	3622404	24171366	-20851	24171366	28279265	false
	CPOR 1	101362351	112936934	160883212	171186395	-	#C18B50	11574583	10303183	-20851	1901	-20851	11553732	false
	CPOR 1	112938835	113049970	160328728	160400329	+	#C18B50	111135	71601	1901	-111135	1901	113036	false
	CPOR 1	112938835	113115345	160417546	160521229	-	#C18B50	176510	103683	-111135	-18031	-111135	65375	false
	CPOR 1	113097314	115976644	157646615	160304160	-	#C18B50	2879330	2657545	-18031	45	-18031	2861299	false
	CPOR 1	115976689	116037751	157591983	157646615	+	#C18B50	61062	54632	45	59	45	61107	false
	CPOR 1	116037810	121066447	152661015	157591982	-	#C18B50	5028637	4930967	59	-50198	59	5028696	false
	CPOR 1	121016249	121447760	152250899	152660957	-	#C18B50	431511	410058	-50198	-8519	-50198	381313	false
	CPOR 1	121439241	122454578	181062731	182054627	+	#C18B50	1015337	991896	-8519	18347	-8519	1006818	false
	CPOR 1	122472925	122764184	182283332	182632311	+	#C18B50	291259	348979	18347	12323	18347	309606	false
	CPOR 1	122776507	128154332	182697232	187216994	+	#C18B50	5377825	4519762	12323	1775	12323	5390148	false
	CPOR 1	128156107	133174549	189737344	192940213	+	#C18B50	5018442	3202869	1775	156756	1775	5020217	false
	CPOR 1	133331305	135530996	193105721	194471092	-	#C18B50	2199691	1365371	156756	8553	156756	2356447	false
	CPOR 1	135539549	136724262	194514568	195211388	-	#C18B50	1184713	696820	8553	14515	8553	1193266	false
	CPOR 1	136738777	138124581	195218257	196226901	+	#C18B50	1385804	1008644	14515		14515	1400319	false
**HSA X**	CPOR X	4099681	4396770	152033819	152088404	-	#00FF00	297089	54585		168441			
	CPOR X	4565211	4806941	151914803	152016189	-	#00FF00	241730	101386	168441	50943	168441	410171	false
	CPOR X	4857884	5143813	151762886	151908918	-	#00FF00	285929	146032	50943	2442	50943	336872	false
	CPOR X	5146255	8033013	148609380	151348521	-	#00FF00	2886758	2739141	2442	-135092	2442	2889200	false
	CPOR 8	7040245	7353347	103366942	103616692	+	#60FA36	313102	249750		544049			

	CPOR 8	7897396	7996791	103656343	103782101	+	#60FA36	99395	125758	544049	122198922	544049	643444	false
	CPOR X	7897921	8869597	146940549	147925241	+	#00FF00	971676	984692	-135092	31005	-135092	836584	false
	CPOR X	8900602	8960151	145477377	145553111	+	#00FF00	59549	75734	31005	74709	31005	90554	false
	CPOR X	9034860	9962184	145585016	146416169	+	#00FF00	927324	831153	74709	2301	74709	1002033	false
	CPOR X	9964485	12903481	140301806	143151368	-	#00FF00	2938996	2849562	2301	1100	2301	2941297	false
	CPOR X	12904581	13344820	139853224	140242217	-	#00FF00	440239	388993	1100	52425	1100	441339	false
	CPOR X	13397245	15932396	137027223	139802390	-	#00FF00	2535151	2775167	52425	-1794526	52425	2587576	false
	CPOR X	14137870	14233753	144798187	144888665	-	#00FF00	95883	90478	-1794526	1467602	-1794526	-1698643	true
	CPOR X	15701355	15782144	137241654	137318097	-	#00FF00	80789	76443	1467602	150271	1467602	1548391	false
	CPOR X	15932415	16006522	136917081	137027223	+	#00FF00	74107	110142	150271	225	150271	224378	false
	CPOR X	16006747	16279695	136602684	136917044	-	#00FF00	272948	314360	225	-42837	225	273173	false
	CPOR X	16236858	16321408	136529392	136645452	+	#00FF00	84550	116060	-42837	6419	-42837	41713	false
	CPOR X	16327827	18374505	134426905	136529392	-	#00FF00	2046678	2102487	6419	5292	6419	2053097	false
	CPOR X	18379797	23569176	129349089	134376022	-	#00FF00	5189379	5026933	5292	870	5292	5194671	false
	CPOR X	23570046	23635722	129159794	129222472	-	#00FF00	65676	62678	870	-28611	870	66546	false
	CPOR X	23607111	26224427	126618667	129114337	-	#00FF00	2617316	2495670	-28611	-75596	-28611	2588705	false
	CPOR X	26148831	26224037	125800091	125928140	-	#00FF00	75206	128049	-75596	-75206	-75596	-390	true
	CPOR X	26148831	28124919	123575862	125654855	-	#00FF00	1976088	2078993	-75206	-1966380	-75206	1900882	false
	CPOR X	26158539	26224427	126461341	126520622	-	#00FF00	65888	59281	-1966380	-65413	-1966380	-1900492	true
	CPOR X	26159014	26225698	126141282	126206653	+	#00FF00	66684	65371	-65413	-59666	-65413	1271	false
	CPOR X	26166032	26250533	126057583	126122317	+	#00FF00	84501	64734	-59666	1875932	-59666	24835	false
	CPOR X	28126465	30266263	121385827	123523856	-	#00FF00	2139798	2138029	1875932	-27673	1875932	4015730	false
	CPOR X	30238590	34116023	116947801	121094408	-	#00FF00	3877433	4146607	-27673	55767	-27673	3849760	false
	CPOR X	34171790	34348331	116513806	116928915	-	#00FF00	176541	415109	55767	4	55767	232308	false
	CPOR X	34348335	34536520	115464477	115640479	-	#00FF00	188185	176002	4	25206	4	188189	false
	CPOR X	34561726	35803120	113794369	115411228	-	#00FF00	1241394	1616859	25206	-911640	25206	1266600	false
	CPOR X	34891480	35026830	112747458	112862798	+	#00FF00	135350	115340	-911640	359723	-911640	-776290	true
	CPOR X	35386553	35579774	113234007	113412984	+	#00FF00	193221	178977	359723	58212	359723	552944	false
	CPOR X	35637986	35710449	113418970	113499203	+	#00FF00	72463	80233	58212	88033	58212	130675	false
	CPOR X	35798482	36517125	113124752	113769347	-	#00FF00	718643	644595	88033	-38924	88033	806676	false
	CPOR X	36478201	36678475	114407199	114677234	+	#00FF00	200274	270035	-38924	-35218	-38924	161350	false
	CPOR X	36643257	36721544	112876833	112946535	-	#00FF00	78287	69702	-35218	61725	-35218	43069	false

	CPOR X	36783269	36908657	112656514	112865510	-	#00FF00	125388	208996	61725	-30854	61725	187113	false
	CPOR X	36877803	36966884	112429977	112625857	-	#00FF00	89081	195880	-30854	1446	-30854	58227	false
	CPOR X	36968330	37078961	111862572	111934079	+	#00FF00	110631	71507	1446	212232	1446	112077	false
	CPOR X	37291193	37507034	112167104	112429811	+	#00FF00	215841	262707	212232	-33497	212232	428073	false
	CPOR X	37473537	45364699	103678722	111974210	-	#00FF00	7891162	8295488	-33497	60459	-33497	7857665	false
	CPOR X	45425158	45566813	103430946	103662806	-	#00FF00	141655	231860	60459	437	60459	202114	false
	CPOR X	45567250	45624303	103222438	103378649	-	#00FF00	57053	156211	437	16212	437	57490	false
	CPOR X	45640515	47094212	101513321	103167190	-	#00FF00	1453697	1653869	16212	-26925	16212	1469909	false
	CPOR X	47067287	47315549	101350352	101510633	-	#00FF00	248262	160281	-26925	48248	-26925	221337	false
	CPOR X	47363797	47663945	100887629	101251819	-	#00FF00	300148	364190	48248	113420	48248	348396	false
	CPOR X	47777365	48011740	100536156	100874313	-	#00FF00	234375	338157	113420	-35536	113420	347795	false
	CPOR X	47976204	48166579	100386953	100535801	-	#00FF00	190375	148848	-35536	225179	-35536	154839	false
	CPOR X	48391758	49346447	99579592	100388554	-	#00FF00	954689	808962	225179	186869	225179	1179868	false
	CPOR X	49533316	50146582	98968651	99582204	-	#00FF00	613266	613553	186869	133707	186869	800135	false
	CPOR X	50280289	50528151	98445214	98892645	-	#00FF00	247862	447431	133707	7476	133707	381569	false
	CPOR X	50535627	50927717	97765637	98334512	-	#00FF00	392090	568875	7476	59621	7476	399566	false
	CPOR X	50987338	51047161	97489618	97563554	-	#00FF00	59823	73936	59621	331302	59621	119444	false
	CPOR X	51378463	51619037	97160087	97357685	-	#00FF00	240574	197598	331302	91305	331302	571876	false
	CPOR X	51710342	51936073	96804919	97106503	-	#00FF00	225731	301584	91305	178258	91305	317036	false
	CPOR X	52114331	52212257	96540609	96667105	-	#00FF00	97926	126496	178258	675313	178258	276184	false
	CPOR X	52887570	52941093	96221075	96366388	+	#00FF00	53523	145313	675313	-26373	675313	728836	false
	CPOR X	52914720	53716174	95542283	96366388	-	#00FF00	801454	824105	-26373	50363	-26373	775081	false
	CPOR X	53766537	53822766	95880346	95933313	-	#00FF00	56229	52967	50363	107052	50363	106592	false
	CPOR X	53929818	54073350	94881281	95089781	-	#00FF00	143532	208500	107052	19382	107052	250584	false
	CPOR X	54092732	54403431	94347978	94800656	-	#00FF00	310699	452678	19382	36408	19382	330081	false
	CPOR X	54439839	54563139	94041397	94221189	-	#00FF00	123300	179792	36408	160331	36408	159708	false
	CPOR X	54723470	54790593	93760236	93815099	+	#00FF00	67123	54863	160331	-17111	160331	227454	false
	CPOR X	54773482	54844186	93798031	93919284	-	#00FF00	70704	121253	-17111	68759	-17111	53593	false
	CPOR X	54912945	55036803	93429991	93591962	-	#00FF00	123858	161971	68759	677323	68759	192617	false
	CPOR X	55714126	55795399	93159337	93239264	-	#00FF00	81273	79927	677323	428810	677323	758596	false
	CPOR X	56224209	56302272	92942407	93081792	-	#00FF00	78063	139385	428810	1594584	428810	506873	false
	CPOR X	57896856	57962355	92046084	92117598	+	#00FF00	65499	71514	1594584	5669806	1594584	1660083	false

	CPOR X	63632161	63838642	86418855	86668146	+	#00FF00	206481	249291	5669806	339946	5669806	5876287	false
	CPOR X	64178588	64274194	86842110	86943123	-	#00FF00	95606	101013	339946	639745	339946	435552	false
	CPOR X	64913939	65114429	87105731	87312227	+	#00FF00	200490	206496	639745	380813	639745	840235	false
	CPOR X	65495242	65747493	87725580	88050081	+	#00FF00	252251	324501	380813	214312	380813	633064	false
	CPOR X	65961805	66288762	88115209	88524167	+	#00FF00	326957	408958	214312	90716	214312	541269	false
	CPOR X	66379478	66449636	88537203	88628302	+	#00FF00	70158	91099	90716	140262	90716	160874	false
	CPOR X	66589898	66816140	88890135	89078101	+	#00FF00	226242	187966	140262	50598	140262	366504	false
	CPOR X	66866738	66921495	89138406	89264363	+	#00FF00	54757	125957	50598	95915	50598	105355	false
	CPOR X	67017410	67143607	89338748	89459537	+	#00FF00	126197	120789	95915	114090	95915	222112	false
	CPOR X	67257697	67351785	89593801	89701067	+	#00FF00	94088	107266	114090	168347	114090	208178	false
	CPOR X	67520132	67790186	89960081	90301968	+	#00FF00	270054	341887	168347	143320	168347	438401	false
	CPOR X	67933506	68583798	90574754	91278735	+	#00FF00	650292	703981	143320	206749	143320	793612	false
	CPOR X	68790547	69474125	85370595	86208413	-	#00FF00	683578	837818	206749	14037	206749	890327	false
	CPOR X	69488162	70563129	83869601	85314901	-	#00FF00	1074967	1445300	14037	238649	14037	1089004	false
	CPOR X	70801778	70956310	83483798	83657362	-	#00FF00	154532	173564	238649	80771	238649	393181	false
	CPOR X	71037081	71661441	82298781	82960600	-	#00FF00	624360	661819	80771	196235	80771	705131	false
	CPOR X	71857676	72282034	81807018	82322069	-	#00FF00	424358	515051	196235	12458	196235	620593	false
	CPOR X	72294492	72810369	81212347	81750235	-	#00FF00	515877	537888	12458	915900	12458	528335	false
	CPOR X	73726269	73792534	80326563	80393851	-	#00FF00	66265	67288	915900	148283	915900	982165	false
	CPOR X	73940817	73997827	80007817	80080368	-	#00FF00	57010	72551	148283	235356	148283	205293	false
	CPOR X	74233183	74313066	79698092	79842985	-	#00FF00	79883	144893	235356	97525	235356	315239	false
	CPOR X	74410591	74468384	79334579	79421607	+	#00FF00	57793	87028	97525	101895	97525	155318	false
	CPOR X	74570279	74632799	79072100	79145273	-	#00FF00	62520	73173	101895	95796	101895	164415	false
	CPOR X	74728595	75161693	78224415	78854327	-	#00FF00	433098	629912	95796	66797	95796	528894	false
	CPOR X	75228490	75517138	77667311	78031015	-	#00FF00	288648	363704	66797	1337141	66797	355445	false
	CPOR X	76854279	77098669	76494813	76667075	-	#00FF00	244390	172262	1337141	243172	1337141	1581531	false
	CPOR X	77341841	77419055	76176622	76271408	-	#00FF00	77214	94786	243172	306	243172	320386	false
	CPOR X	77419361	77723998	75637421	76008668	-	#00FF00	304637	371247	306	50228	306	304943	false
	CPOR X	77774226	78150623	75102848	75622119	-	#00FF00	376397	519271	50228	117942	50228	426625	false
	CPOR X	78268565	78334313	74969321	75070328	-	#00FF00	65748	101007	117942	735673	117942	183690	false
	CPOR X	79069986	79137228	74453709	74556082	-	#00FF00	67242	102373	735673	2535	735673	802915	false
	CPOR X	79139763	79272541	74209885	74392133	-	#00FF00	132778	182248	2535	984690	2535	135313	false

	CPOR X	80257231	80346135	74080242	74152793	-	#00FF00	88904	72551	984690	304133	984690	1073594	false
	CPOR X	80650268	80811091	73652329	73883874	-	#00FF00	160823	231545	304133	383288	304133	464956	false
	CPOR X	81194379	81298283	73331280	73399827	-	#00FF00	103904	68547	383288	2117268	383288	487192	false
	CPOR X	83415551	83576920	72156850	72412692	-	#00FF00	161369	255842	2117268	458116	2117268	2278637	false
	CPOR X	84035036	84190349	71502417	71762132	-	#00FF00	155313	259715	458116	79073	458116	613429	false
	CPOR X	84269422	84526348	71225202	71461182	-	#00FF00	256926	235980	79073	470752	79073	335999	false
	CPOR X	84997100	85549412	70418578	71113700	-	#00FF00	552312	695122	470752	310427	470752	1023064	false
	CPOR X	85859839	86057124	70077469	70257336	-	#00FF00	197285	179867	310427	1244366	310427	507712	false
	CPOR X	87301490	87432348	69725654	69861348	-	#00FF00	130858	135694	1244366	81351	1244366	1375224	false
	CPOR X	87513699	87670164	69529965	69723820	-	#00FF00	156465	193855	81351	4038567	81351	237816	false
	CPOR X	91708731	91844949	68459561	68726536	+	#00FF00	136218	266975	4038567	299631	4038567	4174785	false
	CPOR X	92144580	92267480	68933406	69144265	+	#00FF00	122900	210859	299631	2955806	299631	422531	false
	CPOR X	95223286	95422145	67667365	67826386	-	#00FF00	198859	159021	2955806	197553	2955806	3154665	false
	CPOR X	95619698	95914868	67181125	67663277	-	#00FF00	295170	482152	197553	36230	197553	492723	false
	CPOR X	95951098	96428545	66211564	67045056	-	#00FF00	477447	833492	36230	3349	36230	513677	false
	CPOR X	96431894	96503640	65987183	66129503	-	#00FF00	71746	142320	3349	67925	3349	75095	false
	CPOR X	96571565	97713976	64611651	65817965	-	#00FF00	1142411	1206314	67925	111152	67925	1210336	false
	CPOR X	97825128	98534243	63449029	64355894	-	#00FF00	709115	906865	111152	17085	111152	820267	false
	CPOR X	98551328	98829222	62994174	63392851	-	#00FF00	277894	398677	17085	119026	17085	294979	false
	CPOR X	98948248	99196196	62295218	62781925	-	#00FF00	247948	486707	119026	52769	119026	366974	false
	CPOR X	99248965	99531181	61974846	62290725	-	#00FF00	282216	315879	52769	54762	52769	334985	false
	CPOR X	99585943	99986126	61415708	61924404	-	#00FF00	400183	508696	54762	16365	54762	454945	false
	CPOR X	100002491	101293459	59737239	61348961	-	#00FF00	1290968	1611722	16365	50072	16365	1307333	false
	CPOR X	101343531	101563221	59563779	59721411	-	#00FF00	219690	157632	50072	32228	50072	269762	false
	CPOR X	101595449	101994222	59080714	59511725	-	#00FF00	398773	431011	32228	91517	32228	431001	false
	CPOR X	102085739	102148840	58954019	59011398	-	#00FF00	63101	57379	91517	121509	91517	154618	false
	CPOR X	102270349	102368216	58803401	58861697	+	#00FF00	97867	58296	121509	-49318	121509	219376	false
	CPOR X	102318898	102416771	58803401	58861697	-	#00FF00	97873	58296	-49318	151306	-49318	48555	false
	CPOR X	102568077	102682056	58695088	58769220	-	#00FF00	113979	74132	151306	11354	151306	265285	false
	CPOR X	102693410	102759409	58548780	58642305	-	#00FF00	65999	93525	11354	333160	11354	77353	false
	CPOR X	103092569	103189210	59011412	59073463	+	#00FF00	96641	62051	333160	29958	333160	429801	false
	CPOR X	103219168	103452590	58323475	58540762	-	#00FF00	233422	217287	29958	72208	29958	263380	false

	CPOR X	103524798	103847035	58000160	58314082	-	#00FF00	322237	313922	72208	158063	72208	394445	false
	CPOR X	104005098	106044838	55924430	57980868	-	#00FF00	2039740	2056438	158063	57762	158063	2197803	false
	CPOR X	106102600	106349889	55750603	55928020	-	#00FF00	247289	177417	57762	-137141	57762	305051	false
	CPOR X	106212748	106350308	55747243	55857705	+	#00FF00	137560	110462	-137141	17749	-137141	419	false
	CPOR X	106368057	106479623	55622013	55686920	-	#00FF00	111566	64907	17749	-42439	17749	129315	false
	CPOR X	106437184	106511965	55266776	55322902	+	#00FF00	74781	56126	-42439	-52054	-42439	32342	false
	CPOR X	106459911	106591071	55380587	55529864	+	#00FF00	131160	149277	-52054	340	-52054	79106	false
	CPOR X	106591411	107019072	54664230	55159994	-	#00FF00	427661	495764	340	42678	340	428001	false
	CPOR X	107061750	108751471	52469593	54472729	-	#00FF00	1689721	2003136	42678	208708	42678	1732399	false
	CPOR X	108960179	109052423	52112975	52213424	-	#00FF00	92244	100449	208708	168941	208708	300952	false
	CPOR X	109221364	110546662	50354363	51931832	-	#00FF00	1325298	1577469	168941	5169	168941	1494239	false
	CPOR X	110551831	110639969	50272562	50354363	+	#00FF00	88138	81801	5169	6204	5169	93307	false
	CPOR X	110646173	113130467	47344388	50272562	-	#00FF00	2484294	2928174	6204	29150	6204	2490498	false
	CPOR X	113159617	113231101	47094399	47156624	+	#00FF00	71484	62225	29150	43637	29150	100634	false
	CPOR X	113274738	113445342	46855702	47061181	-	#00FF00	170604	205479	43637	6378	43637	214241	false
	CPOR X	113451720	114279885	45632355	46785711	-	#00FF00	828165	1153356	6378	52659	6378	834543	false
	CPOR X	114332544	115727199	44105397	45616589	-	#00FF00	1394655	1511192	52659	408409	52659	1447314	false
	CPOR X	116135608	116212362	43680748	43796512	-	#00FF00	76754	115764	408409	8444	408409	485163	false
	CPOR X	116220806	116658594	43211941	43614736	-	#00FF00	437788	402795	8444	166603	8444	446232	false
	CPOR X	116825197	117069499	42754559	43024376	-	#00FF00	244302	269817	166603	22498	166603	410905	false
	CPOR X	117091997	119728432	40036375	42696717	-	#00FF00	2636435	2660342	22498	241272	22498	2658933	false
	CPOR X	119969704	120058164	39901559	40033604	-	#00FF00	88460	132045	241272	143418	241272	329732	false
	CPOR X	120201582	120876436	38836768	39550463	-	#00FF00	674854	713695	143418	66827	143418	818272	false
	CPOR X	120943263	124135424	35543079	38839999	-	#00FF00	3192161	3296920	66827	53517	66827	3258988	false
	CPOR X	124188941	124247808	35349209	35543079	-	#00FF00	58867	193870	53517	0	53517	112384	false
	CPOR X	124247808	126894449	32740795	35294125	-	#00FF00	2646641	2553330	0	58632	0	2646641	false
	CPOR X	126953081	127335159	32167078	32658785	-	#00FF00	382078	491707	58632	58562	58632	440710	false
	CPOR X	127393721	127471782	32005161	32097177	+	#00FF00	78061	92016	58562	225612	58562	136623	false
	CPOR X	127697394	127830330	31641380	31795702	+	#00FF00	132936	154322	225612	-100	225612	358548	false
	CPOR X	127830230	127950808	31347173	31453648	-	#00FF00	120578	106475	-100	8	-100	120478	false
	CPOR X	127950816	128052587	31170632	31290248	-	#00FF00	101771	119616	8	73112	8	101779	false
	CPOR X	128125699	128213218	31047246	31172328	-	#00FF00	87519	125082	73112	41222	73112	160631	false

	CPOR X	128254440	128656805	30546350	30984007	-	#00FF00	402365	437657	41222	2716	41222	443587	false
	CPOR X	128659521	129896798	28872819	30175806	-	#00FF00	1237277	1302987	2716	587	2716	1239993	false
	CPOR X	129897385	131678741	26585083	28502208	-	#00FF00	1781356	1917125	587	125045	587	1781943	false
	CPOR X	131803786	135001616	23286793	26384463	-	#00FF00	3197830	3097670	125045	3845	125045	3322875	false
	CPOR X	135005461	135105927	20824723	20975895	-	#00FF00	100466	151172	3845	24476	3845	104311	false
	CPOR X	135130403	135216343	20360798	20427605	-	#00FF00	85940	66807	24476	-55317	24476	110416	false
	CPOR X	135161026	135295098	20365617	20453805	+	#00FF00	134072	88188	-55317	-47726	-55317	78755	false
	CPOR X	135247372	135386109	20964132	21120873	-	#00FF00	138737	156741	-47726	-132057	-47726	91011	false
	CPOR X	135254052	135400967	21064829	21134377	-	#00FF00	146915	69548	-132057	-79343	-132057	14858	false
	CPOR X	135321624	135449316	20186930	20284696	-	#00FF00	127692	97766	-79343	-28389	-79343	48349	false
	CPOR X	135420927	135583159	19874630	20033327	-	#00FF00	162232	158697	-28389	6904	-28389	133843	false
	CPOR X	135590063	135687485	19788175	19884409	+	#00FF00	97422	96234	6904	22847	6904	104326	false
	CPOR X	135710332	135886920	19785030	19871009	+	#00FF00	176588	85979	22847	42623	22847	199435	false
	CPOR X	135929543	140879318	14850292	19822646	-	#00FF00	4949775	4972354	42623	8464	42623	4992398	false
	CPOR X	140887782	140995024	14663856	14791246	-	#00FF00	107242	127390	8464	40380	8464	115706	false
	CPOR X	141035404	141472695	14332953	14663500	+	#00FF00	437291	330547	40380	1291	40380	477671	false
	CPOR X	141473986	141860993	14087826	14408163	-	#00FF00	387007	320337	1291	-3178	1291	388298	false
	CPOR X	141857815	142148667	13875439	14086627	+	#00FF00	290852	211188	-3178	-4527	-3178	287674	false
	CPOR X	142144140	142697994	13409157	13906428	-	#00FF00	553854	497271	-4527	7206	-4527	549327	false
	CPOR X	142705200	142787261	13344508	13405159	+	#00FF00	82061	60651	7206	9289	7206	89267	false
	CPOR X	142796550	144423729	11826462	13338927	-	#00FF00	1627179	1512465	9289	59219	9289	1636468	false
	CPOR X	144482948	144861727	11547842	11824961	-	#00FF00	378779	277119	59219	42	59219	437998	false
	CPOR X	144861769	145002404	11453333	11547151	+	#00FF00	140635	93818	42	0	42	140677	false
	CPOR X	145002404	145279696	11149155	11450446	-	#00FF00	277292	301291	0	1516	0	277292	false
	CPOR X	145281212	145361122	10796204	10913144	-	#00FF00	79910	116940	1516	0	1516	81426	false
	CPOR X	145361122	146764131	9423581	10739052	-	#00FF00	1403009	1315471	0	2068	0	1403009	false
	CPOR X	146766199	146931830	9245113	9423581	+	#00FF00	165631	178468	2068	37185	2068	167699	false
	CPOR X	146969015	147219203	8973265	9198883	+	#00FF00	250188	225618	37185	-42589	37185	287373	false
	CPOR X	147176614	147279092	8863120	8983753	-	#00FF00	102478	120633	-42589	-29459	-42589	59889	false
	CPOR X	147249633	149552179	6853909	8863554	-	#00FF00	2302546	2009645	-29459	-20803	-29459	2273087	false
	CPOR X	149531376	149636879	5233487	5329616	+	#00FF00	105503	96129	-20803	1297	-20803	84700	false
	CPOR X	149638176	149704233	6561841	6621334	+	#00FF00	66057	59493	1297	223717	1297	67354	false

	CPOR X	149927950	151001748	4360367	5253184	-	#00FF00	1073798	892817	223717	149839	223717	1297515	false
	CPOR X	151151587	151495336	4093811	4338297	-	#00FF00	343749	244486	149839	2405	149839	493588	false
	CPOR X	151497741	151932937	3634839	4009305	-	#00FF00	435196	374466	2405	-6531	2405	437601	false
	CPOR X	151926406	152129275	3456359	3668307	+	#00FF00	202869	211948	-6531	-25053	-6531	196338	false
	CPOR X	152104222	152670628	2918199	3474360	-	#00FF00	566406	556161	-25053	137241	-25053	541353	false
	CPOR X	152807869	153043973	2724532	2907762	-	#00FF00	236104	183230	137241	216161	137241	373345	false
	CPOR X	153260134	154553010	1394366	2329554	-	#00FF00	1292876	935188	216161	103164	216161	1509037	false
	CPOR X	154656174	155383049	457420	1191528	-	#00FF00	726875	734108	103164	79313	103164	830039	false
	CPOR X	155462362	155942085	0	455033	-	#00FF00	479723	455033	79313		79313	559036	false

The column names are similar to the column names in the Supplementary Table S1, but the color code of the chromosome corresponds to the block in the query genome in the Supplementary Figure S5.

Supplementary Table S4 (part 1). Syntenic blocks with a minimum cut-off length of 50 kb in the reference guinea pig genome without regard to block orientation.

scaffold	query	start	end	color	target_len
**CPOR 1**	HSA 2	36451	6006258	#036A9C	5969807
	HSA 14	7349946	35112351	#FFFF00	27762405
	HSA 15	35144936	47077202	#EC83BA	11932266
	HSA 9	47695388	91980048	#819EF7	44284660
	HSA 1	92030842	152094638	#00FF7F	60063796
	HSA 9	152250899	187216994	#819EF7	34966095
	HSA 9	189737344	196226901	#819EF7	6489557
	HSA 2	196814789	233513972	#036A9C	36699183
**CPOR 10**	HSA 8	2106505	15713048	#80797D	13606543
	HSA 5	16063387	65076099	#F33071	49012712
	HSA 5	64751686	66018366	#F33071	1266680
	HSA 5	65683898	82402609	#F33071	16718711
**CPOR 11**	HSA 19	2301696	7869002	#64E09A	5567306
	HSA 19	8789573	13225261	#64E09A	4435688
	HSA 11	13888532	24684321	#0000FF	10795789
	HSA 19	25088125	28028633	#64E09A	2940508
	HSA 11	28345172	50098413	#0000FF	21753241
	HSA 9	50128455	57845428	#819EF7	7716973
	HSA 3	57927822	72247757	#02BCB7	14319935
**CPOR 12**	HSA 15	9489	2017955	#EC83BA	2008466
	HSA 6	2121928	6382783	#EBE47D	4260855
	HSA 3	7379674	38114432	#02BCB7	30734758
	HSA 8	38253342	88235300	#80797D	49981958
**CPOR 13**	HSA 3	8586	17993936	#02BCB7	17985350
	HSA 4	18039503	59431073	#7FFFFF	41391570
	HSA 4	59406966	76050087	#7FFFFF	16643121
**CPOR 14**	HSA 3	111898	51454185	#02BCB7	51342287
	HSA 21	51404034	73461731	#007FFF	22057697
**CPOR 15**	HSA 6	400660	60470173	#EBE47D	60069513
**CPOR 16**	HSA 4	63	15633177	#7FFFFF	15633114
	HSA 2	15879417	62564633	#036A9C	46685216
**CPOR 17**	HSA 6	797	14958805	#EBE47D	14958008
	HSA 16	15146175	38568089	#0A803B	23421914
	HSA 16	38591961	41444288	#0A803B	2852327
	HSA 22	41559149	53327959	#F400E1	11768810
	HSA 12	53374380	55190923	#8D3B0C	1816543
	HSA 12	55409237	73322090	#8D3B0C	17912853
**CPOR 18**	HSA 2	259315	78198288	#036A9C	77938973
	HSA 17	78351017	81963280	#FF0000	3612263
**CPOR 19**	HSA 5	677957	21918254	#F33071	21240297
	HSA 13	22670890	61080017	#00FFFF	38409127
**CPOR 2**	HSA 5	157227	9141098	#F33071	8983871
	HSA 5	10269182	28424237	#F33071	18155055

	HSA 1	28652766	32294701	#00FF7F	3641935
	HSA 4	32767307	34122097	#7FFFFF	1354790
	HSA 10	34241568	71347568	#82C90F	37106000
	HSA 9	71654297	76065515	#819EF7	4411218
	HSA 7	76437412	86579440	#7F00FF	10142028
	HSA 7	86670603	145753126	#7F00FF	59082523
**CPOR 20**	HSA 6	2157949	27262690	#EBE47D	25104741
	HSA 1	27470819	35694920	#00FF7F	8224101
	HSA 10	35729009	45256559	#82C90F	9527550
	HSA 3	45624174	68960963	#02BCB7	23336789
**CPOR 21**	HSA 17	375322	10540324	#FF0000	10165002
	HSA 17	11601010	20889258	#FF0000	9288248
	HSA 17	21619340	31242615	#FF0000	9623275
**CPOR 22**	HSA 12	8126	4213966	#8D3B0C	4205840
	HSA 3	4810356	7914244	#02BCB7	3103888
	HSA 10	8309841	17533468	#82C90F	9223627
	HSA 3	17566294	20017799	#02BCB7	2451505
	HSA 10	20222246	24913243	#82C90F	4690997
	HSA 5	25153681	26335048	#F33071	1181367
	HSA 12	28239016	47753260	#8D3B0C	19514244
	HSA 22	47792101	59845734	#F400E1	12053633
**CPOR 23**	HSA 18	241827	68194102	#4E1A96	67952275
**CPOR 24**	HSA 7	32230	31319026	#7F00FF	31286796
	HSA 7	30618654	33550207	#7F00FF	2931553
	HSA 7	33489896	42029816	#7F00FF	8539920
	HSA 10	46572247	48718556	#82C90F	2146309
	HSA 10	48942143	56542663	#82C90F	7600520
**CPOR 25**	HSA 1	524448	52276457	#00FF7F	51752009
**CPOR 26**	HSA 6	229598	9037991	#EBE47D	8808393
	HSA 8	9071624	25530991	#80797D	16459367
	HSA 4	25544846	49428813	#7FFFFF	23883967
	HSA 8	49543790	57427492	#80797D	7883702
**CPOR 27**	HSA 2	576	28080366	#036A9C	28079790
	HSA 14	28438372	34467368	#FFFF00	6028996
	HSA 1	34722178	51422768	#00FF7F	16700590
**CPOR 28**	HSA 6	95584	15196135	#EBE47D	15100551
	HSA 10	15331373	44676762	#82C90F	29345389
	HSA 10	45817809	48398397	#82C90F	2580588
**CPOR 29**	HSA 14	29255	45851339	#FFFF00	45822084
**CPOR 3**	HSA 15	33042	3015569	#EC83BA	2982527
	HSA 13	3254123	48011603	#00FFFF	44757480
	HSA 16	48349662	54860603	#0A803B	6510941
	HSA 4	54886720	74301681	#7FFFFF	19414961
	HSA 16	74441537	112335960	#0A803B	37894423
**CPOR 30**	HSA 4	64	45052184	#7FFFFF	45052120
**CPOR 31**	HSA 1	7	35772809	#00FF7F	35772802

	HSA 8	35778170	47800885	#80797D	12022715
CPOR 4	HSA 15	274027	37596007	#EC83BA	37321980
	HSA 1	38004956	41202299	#00FF7F	3197343
	HSA 7	41492551	61882269	#7F00FF	20389718
	HSA 17	62285036	70960076	#FF0000	8675040
	HSA 17	72057731	95382766	#FF0000	23325035
	HSA 11	95610087	104764596	#0000FF	9154509
CPOR 5	HSA 11	49297	33643567	#0000FF	33594270
	HSA 11	35042807	46760380	#0000FF	11717573
	HSA 11	46722833	91323700	#0000FF	44600867
CPOR 6	HSA 4	315557	5297415	#7FFFFF	4981858
	HSA 16	5302785	7089514	#0A803B	1786729
	HSA 3	7620056	47631742	#02BCB7	40011686
	HSA 20	48426408	98614970	#EF8701	50188562
CPOR 7	HSA 6	348	9739800	#EBE47D	9739452
	HSA 6	14166857	25493079	#EBE47D	11326222
	HSA 10	25747432	37023384	#82C90F	11275952
	HSA 1	37081223	49648672	#00FF7F	12567449
	HSA 19	51755272	62043783	#64E09A	10288511
	HSA 19	63239756	80882754	#64E09A	17642998
	HSA 21	80964311	88883894	#007FFF	7919583
CPOR 8	HSA 22	305237	2085917	#F400E1	1780680
	HSA 12	2086503	72401448	#8D3B0C	70314945
	HSA 2	79284937	85157727	#036A9C	5872790
	HSA 2	85263285	88288224	#036A9C	3024939
	HSA 15	88889117	90906168	#EC83BA	2017051
	HSA 15	91417833	96004299	#EC83BA	4586466
	HSA 15	96251203	97412952	#EC83BA	1161749
	HSA 15	98737743	101761934	#EC83BA	3024191
	HSA 15	101984750	103118061	#EC83BA	1133311
CPOR 9	HSA 5	668075	42377458	#F33071	41709383
	HSA 8	42789355	65051409	#80797D	22262054
	HSA 12	65105595	66826945	#8D3B0C	1721350
CPOR X	HSA X	0	5329616	#00FF00	5329616
	HSA X	6341873	21127672	#00FF00	14785799
	HSA X	23286793	76667075	#00FF00	53380282
	HSA X	77667311	152088404	#00FF00	74421093

Supplementary Table S4 (part 2). Syntenic blocks with a minimum cut-off length of 50 kb in the reference guinea pig genome without regard to block orientation.

scaffold	query	start	end	color	target_len
CPOR 1	HGL 24	36451	6006258	#FF0000	5969807
	HGL 2	6332332	47078620	#1CB707	40746288
	HGL 14	47553527	91980048	#7F00FF	44426521
	HGL 20	91991552	152093744	#00FFFF	60102192
	HGL 14	152257938	180593228	#7F00FF	28335290

	HGL 14	181052434	188126515	#7F00FF	7074081
	HGL 14	189167270	196230313	#7F00FF	7063043
	HGL 5	196814789	233464054	#093456	36649265
**CPOR 10**	HGL 12	44221	9838731	#F33071	9794510
	HGL 17	10076120	15642792	#82C90F	5566672
	HGL 10	16063387	82407688	#02BCB7	66344301
**CPOR 11**	HGL 29	114270	7884794	#F400E1	7770524
	HGL 29	8764323	13643723	#F400E1	4879400
	HGL 22	15897111	22931292	#EC83BA	7034181
	HGL 29	25088078	28041137	#F400E1	2953059
	HGL 22	28157521	52576619	#EC83BA	24419098
	HGL 24	53086454	57845444	#FF0000	4758990
	HGL 6	57927822	72247757	#980157	14319935
**CPOR 12**	HGL 26	9279	2019349	#64E09A	2010070
	HGL 22	2221341	6391459	#EC83BA	4170118
	HGL 4	7377223	38089967	#5654E8	30712744
	HGL 17	38252720	88218628	#82C90F	49965908
**CPOR 13**	HGL 6	8821	17742328	#980157	17733507
	HGL 1	18086471	59370030	#49F643	41283559
	HGL 1	59348248	64969609	#49F643	5621361
	HGL 26	65085019	76100318	#64E09A	11015299
**CPOR 14**	HGL 2	111724	73461729	#1CB707	73350005
**CPOR 15**	HGL 3	400660	60319526	#C3A652	59918866
**CPOR 16**	HGL 12	0	15717188	#F33071	15717188
	HGL 5	15879413	62564633	#093456	46685220
**CPOR 17**	HGL 22	1053	15002797	#EC83BA	15001744
	HGL 19	15004171	73314007	#8D3B0C	58309836
**CPOR 18**	HGL 1	259140	78198288	#49F643	77939148
	HGL 18	78351016	82009440	#0000FF	3658424
**CPOR 19**	HGL 11	93448	21843561	#7FFFFF	21750113
	HGL 15	22666971	61080017	#80797D	38413046
**CPOR 2**	HGL 11	157227	9465414	#7FFFFF	9308187
	HGL 11	10269182	29011108	#7FFFFF	18741926
	HGL 6	32680360	34122398	#980157	1442038
	HGL 13	34235967	71353990	#EBE47D	37118023
	HGL 21	71654297	145850575	#FFFF00	74196278
**CPOR 20**	HGL 4	250588	68959858	#5654E8	68709270
**CPOR 21**	HGL 18	309071	10544768	#0000FF	10235697
	HGL 18	11592353	20890131	#0000FF	9297778
	HGL 18	21619340	31242615	#0000FF	9623275
**CPOR 22**	HGL 8	56954	4214281	#00FF7F	4157327
	HGL 6	4698813	25120606	#980157	20421793
	HGL 11	25153681	26335048	#7FFFFF	1181367
	HGL 8	27445936	59846039	#00FF7F	32400103
**CPOR 23**	HGL 23	141132	68194136	#0A803B	68053004
**CPOR 24**	HGL 3	32107	38447014	#C3A652	38414907

	HGL 9	38905373	41260883	#036A9C	2355510
	HGL 10	46502443	56608146	#02BCB7	10105703
**CPOR 25**	HGL 7	524448	52303456	#B542E4	51779008
**CPOR 26**	HGL 3	229598	9037161	#C3A652	8807563
	HGL 11	9097786	57427499	#7FFFFF	48329713
**CPOR 27**	HGL 24	382	14633358	#FF0000	14632976
	HGL 24	15310813	28068761	#FF0000	12757948
	HGL 2	28278626	34605879	#1CB707	6327253
	HGL 25	34695399	51431783	#4E1A96	16736384
**CPOR 28**	HGL 13	150643	2120833	#EBE47D	1970190
	HGL 13	3646223	44676762	#EBE47D	41030539
	HGL 13	45808878	48398397	#EBE47D	2589519
**CPOR 29**	HGL 28	29255	45857281	#007FFF	45828026
**CPOR 3**	HGL 26	37652	3015569	#64E09A	2977917
	HGL 15	3251537	48015453	#80797D	44763916
	HGL 7	48218368	112335634	#B542E4	64117266
**CPOR 30**	HGL 12	100	45014456	#F33071	45014356
**CPOR 31**	HGL 25	7	35772809	#4E1A96	35772802
	HGL 11	35778650	47813161	#7FFFFF	12034511
**CPOR 4**	HGL 9	724175	25641953	#036A9C	24917778
	HGL 2	25691209	37796299	#1CB707	12105090
	HGL 20	38010038	41204935	#00FFFF	3194897
	HGL 24	41491042	48991320	#FF0000	7500278
	HGL 22	49093723	59735858	#EC83BA	10642135
	HGL 22	59838227	61820492	#EC83BA	1982265
	HGL 18	62283975	70960129	#0000FF	8676154
	HGL 18	72084768	95350692	#0000FF	23265924
	HGL 8	95587122	104768829	#00FF7F	9181707
**CPOR 5**	HGL 8	49797	17157488	#00FF7F	17107691
	HGL 8	17777196	91323700	#00FF7F	73546504
**CPOR 6**	HGL 6	315556	7089514	#980157	6773958
	HGL 6	7582570	47763402	#980157	40180832
	HGL 9	48423401	98618828	#036A9C	50195427
**CPOR 7**	HGL 3	0	9730460	#C3A652	9730460
	HGL 3	14206613	25480651	#C3A652	11274038
	HGL 27	25747432	49454735	#EF8701	23707303
	HGL 27	51087751	59470273	#EF8701	8382522
	HGL 27	60765261	62164263	#EF8701	1399002
	HGL 27	63239925	80882754	#EF8701	17642829
	HGL 2	80964311	88894802	#1CB707	7930491
**CPOR 8**	HGL 16	43733	72370111	#819EF7	72326378
	HGL 26	79284237	103349682	#64E09A	24065445
**CPOR 9**	HGL 5	622879	42377458	#093456	41754579
	HGL 12	42789355	62321552	#F33071	19532197
	HGL 12	63036879	66943108	#F33071	3906229
**CPOR X**	HGL X	0	4113201	#00FF00	4113201

	HGL X	5233522	21319139	#00FF00	16085617
	HGL X	22359753	91225543	#00FF00	68865790
	HGL X	92026123	143181214	#00FF00	51155091
	HGL 19	143212856	144431980	#8D3B0C	1219124
	HGL X	144798200	152291774	#00FF00	7493574

The column names and the color code of the chromosome are the same as in the Supplementary Table S1. In first column green color indicates the complete agreement with FISH (L > 5Mbp), blue color markers new synteny blocks (1 Mbp < L < 5 Mbp), yellow color shows complete agreement with previous alignment, but some disagreement with FISH.

Supplementary Table S5 (part 1). Syntenic blocks with a minimum cut-off length of 50 kilobases in the reference naked mole-rat genome without regard to block orientation.

scaffold	query	start	end	color	target_len
HGL 1	HSA 2	45687	85931005	#4C0851	85885318
	HSA 4	86000603	113212490	#11559D	27211887
	HSA 4	113212556	133260703	#11559D	20048147
HGL 10	HSA 5	17737	70100096	#FDB568	70082359
	HSA 10	70777945	83208221	#7AB506	12430276
HGL 11	HSA 8	109262	16875710	#02B4A9	16766448
	HSA 4	16920006	43066400	#11559D	26146394
	HSA 8	43161889	65899910	#02B4A9	22738021
	HSA 1	66482208	69115829	#CBE5A7	2633621
	HSA 5	70372771	122742221	#FDB568	52369450
	HSA 1	123168261	125182313	#CBE5A7	2014052
HGL 12	HSA 4	201356	66330555	#11559D	66129199
	HSA 8	66457344	88145568	#02B4A9	21688224
	HSA 12	88187549	89966389	#7444DF	1778840
	HSA 22	89967493	90990455	#DB00F9	1022962
	HSA 8	91953133	100074019	#02B4A9	8120886
HGL 13	HSA 6	10928	12826731	#564208	12815803
	HSA 10	12897815	87301593	#7AB506	74403778
HGL 14	HSA 9	58790	91878552	#FA2D80	91819762
HGL 15	HSA 13	901231	80143448	#007F00	79242217
	HSA 13	80260497	93260380	#007F00	12999883
HGL 16	HSA 22	247663	2062714	#DB00F9	1815051
	HSA 12	2063325	53766260	#7444DF	51702935
	HSA 12	55046004	76344281	#7444DF	21298277
HGL 17	HSA 8	86508	60190451	#02B4A9	60103943
HGL 18	HSA 17	36474	64827220	#C9FE31	64790746
HGL 19	HSA 16	62344	14508330	#49FEF6	14445986
	HSA 16	15079807	21157891	#49FEF6	6078084
	HSA 16	21259241	25964115	#49FEF6	4704874
	HSA 22	25987289	37461277	#DB00F9	11473988
	HSA 12	37492442	39459681	#7444DF	1967239
	HSA 12	39673369	55484456	#7444DF	15811087

**HGL 2**	HSA 21	15283	29116325	#FF7F00	29101042
	HSA 3	29128843	74548275	#9B0496	45419432
	HSA 3	74845857	82581126	#9B0496	7735269
	HSA 15	82903262	91443037	#AF0926	8539775
	HSA 14	91484515	118135951	#E181CE	26651436
	HSA 14	118144029	125018939	#E181CE	6874910
	HSA 15	127767291	139843863	#AF0926	12076572
**HGL 20**	HSA 1	14433	63505227	#CBE5A7	63490794
**HGL 21**	HSA 7	645465	64757386	#81A8F8	64111921
	HSA 7	64757498	69198002	#81A8F8	4440504
	HSA 7	69222011	75320343	#81A8F8	6098332
	HSA 9	76760503	80564350	#FA2D80	3803847
**HGL 22**	HSA 11	3031473	33245877	#69E283	30214404
	HSA 9	33329101	35599562	#FA2D80	2270461
	HSA 6	35611514	54236282	#564208	18624768
	HSA 7	55044974	67144732	#81A8F8	12099758
**HGL 23**	HSA 18	18919	69470893	#0800AC	69451974
**HGL 24**	HSA 2	42028	35824115	#4C0851	35782087
	HSA 7	35936360	43583762	#81A8F8	7647402
	HSA 9	43585302	49654100	#FA2D80	6068798
**HGL 25**	HSA 1	39669	54742351	#CBE5A7	54702682
**HGL 26**	HSA 4	16224	10408517	#11559D	10392293
	HSA 15	10557474	28779863	#AF0926	18222389
	HSA 2	31128447	41239835	#4C0851	10111388
**HGL 27**	HSA 10	179490	10257159	#7AB506	10077669
	HSA 1	10341494	22798482	#CBE5A7	12456988
	HSA 19	22869462	44037548	#60765F	21168086
**HGL 28**	HSA 14	25874	46254903	#E181CE	46229029
**HGL 29**	HSA 19	401166	13456390	#60765F	13055224
**HGL 3**	HSA 7	2975	7993814	#81A8F8	7990839
	HSA 7	7283543	10311044	#81A8F8	3027501
	HSA 7	10246408	32700556	#81A8F8	22454148
	HSA 6	32820649	40499136	#564208	7678487
	HSA 11	41996200	46089844	#69E283	4093644
	HSA 6	46198222	135280965	#564208	89082743
**HGL 4**	HSA 6	706478	24877504	#564208	24171026
	HSA 1	25520401	34547257	#CBE5A7	9026856
	HSA 10	34595050	45263418	#7AB506	10668368
	HSA 3	45522830	102034657	#9B0496	56511827
**HGL 5**	HSA 5	211197	43403360	#FDB568	43192163
	HSA 2	43484472	57777354	#4C0851	14292882
	HSA 2	58311499	127547228	#4C0851	69235729
**HGL 6**	HSA 3	50282	76549912	#9B0496	76499630
	HSA 4	77063869	84540723	#11559D	7476854
	HSA 16	84590879	86929461	#49FEF6	2338582
	HSA 3	87408673	92999287	#9B0496	5590614

	HSA 10	93237868	105311489	#7AB506	12073621
HGL 7	HSA 16	25963	37355642	#49FEF6	37329679
	HSA 4	37381410	56765431	#11559D	19384021
	HSA 1	56816501	106798643	#CBE5A7	49982142
HGL 8	HSA 22	7256	12117541	#DB00F9	12110285
	HSA 12	12161006	33033506	#7444DF	20872500
	HSA 11	33693586	124921250	#69E283	91227664
HGL 9	HSA 20	23444	19574076	#007FFF	19550632
	HSA 1	20617413	22733827	#CBE5A7	2116414
	HSA 20	33606723	66255657	#007FFF	32648934
	HSA 15	66346946	91885732	#AF0926	25538786
	HSA 7	91902934	93871691	#81A8F8	1968757
HGL X	HSA X	265040	76837150	#01FC33	76572110
	HSA X	77984595	153264935	#01FC33	75280340

Supplementary Table S5 (part 2). Syntenic blocks with a minimum cut-off length of 50 kilobases in the reference naked mole-rat genome without regard to block orientation.

scaffold	query	start	end	color	target_len
HGL 1	CPOR 18	45687	85929393	#FA2D80	85883706
	CPOR 13	86000603	133337723	#11559D	47337120
HGL 10	CPOR 10	25004	70097658	#CBE5A7	70072654
	CPOR 24	70905498	83284483	#AF0926	12378985
HGL 11	CPOR 26	113062	52031369	#C9FE31	51918307
	CPOR 31	51985295	65289233	#DB00F9	13303938
	CPOR 2	67027071	69208880	#7FFF00	2181809
	CPOR 19	70375290	92965901	#7AB506	22590611
	CPOR 2	92965901	96936093	#7FFF00	3970192
	CPOR 22	96841577	98202672	#007F00	1361095
	CPOR 2	98200986	124601580	#7FFF00	26400594
HGL 12	CPOR 16	31887	15272556	#81A8F8	15240669
	CPOR 30	15278582	60624440	#FF7F00	45345858
	CPOR 30	61687160	66302879	#FF7F00	4615719
	CPOR 9	66457375	90068718	#BF726D	23611343
	CPOR 10	90058565	100074019	#CBE5A7	10015454
HGL 13	CPOR 28	8512	48833388	#60765F	48824876
	CPOR 2	48998029	87311750	#7FFF00	38313721
HGL 14	CPOR 1	202252	91881877	#A6F1FC	91679625
HGL 15	CPOR 19	895150	44844868	#7AB506	43949718
	CPOR 3	44827341	93261865	#D9C001	48434524
HGL 16	CPOR 8	44814	53766260	#1036F2	53721446
	CPOR 8	55047831	76344281	#1036F2	21296450
HGL 17	CPOR 12	86334	53624308	#9B0496	53537974
	CPOR 10	54092104	60190451	#CBE5A7	6098347
HGL 18	CPOR 4	33805	30934925	#26B63B	30901120

	CPOR 21	30948727	52519128	#7444DF	21570401
	CPOR 18	52555940	56312545	#FA2D80	3756605
	CPOR 21	56066725	64828114	#7444DF	8761389
**HGL 19**	CPOR 17	62344	14661624	#02B4A9	14599280
	CPOR 17	15042150	45711028	#02B4A9	30668878
	CPOR 17	45577737	55477892	#02B4A9	9900155
**HGL 2**	CPOR 14	15181	10025026	#FDB568	10009845
	CPOR 7	10009588	18278125	#FF0000	8268537
	CPOR 14	18266192	74548275	#FDB568	56282083
	CPOR 14	74841434	82581128	#FDB568	7739694
	CPOR 1	82895219	120810205	#A6F1FC	37914986
	CPOR 27	120806014	125023848	#0800AC	4217834
	CPOR 27	126577043	127720101	#0800AC	1143058
	CPOR 4	127745768	139847614	#26B63B	12101846
**HGL 20**	CPOR 1	14433	60680689	#A6F1FC	60666256
	CPOR 4	60811726	63510366	#26B63B	2698640
**HGL 21**	CPOR 2	469778	80564350	#7FFF00	80094572
**HGL 22**	CPOR 11	2975071	8233378	#4C0851	5258307
	CPOR 11	11132146	20287544	#4C0851	9155398
	CPOR 11	20293478	35600079	#4C0851	15306601
	CPOR 12	35627141	39487995	#9B0496	3860854
	CPOR 17	39501248	44799704	#02B4A9	5298456
	CPOR 17	44800166	54244450	#02B4A9	9444284
	CPOR 4	54696951	67196853	#26B63B	12499902
**HGL 23**	CPOR 23	19468	69472106	#E181CE	69452638
**HGL 24**	CPOR 27	41738	12669605	#0800AC	12627867
	CPOR 1	12691544	18602371	#A6F1FC	5910827
	CPOR 27	18570028	35824115	#0800AC	17254087
	CPOR 4	35939705	43487719	#26B63B	7548014
	CPOR 11	43585302	49654116	#4C0851	6068814
**HGL 25**	CPOR 27	39669	15214610	#0800AC	15174941
	CPOR 31	15216091	17051645	#DB00F9	1835554
	CPOR 31	17131960	52487474	#DB00F9	35355514
	CPOR 27	52487474	53948068	#0800AC	1460594
**HGL 26**	CPOR 13	16515	10427360	#11559D	10410845
	CPOR 8	10537485	17239062	#1036F2	6701577
	CPOR 8	18198410	19789350	#1036F2	1590940
	CPOR 12	19789350	21824042	#9B0496	2034692
	CPOR 3	21824917	24383970	#D9C001	2559053
	CPOR 8	24431464	29341316	#1036F2	4909852
	CPOR 8	31127765	41216681	#1036F2	10088916
**HGL 27**	CPOR 7	184520	44037548	#FF0000	43853028
**HGL 28**	CPOR 29	16484	46252118	#007FFF	46235634
**HGL 29**	CPOR 11	80884	14744336	#4C0851	14663452
**HGL 3**	CPOR 24	1211	30083724	#AF0926	30082513
	CPOR 24	29915028	32702238	#AF0926	2787210

	CPOR 7	32820649	40690468	#FF0000	7869819
	CPOR 11	41887168	42971248	#4C0851	1084080
	CPOR 11	44078266	45718237	#4C0851	1639971
	CPOR 7	45553926	57174241	#FF0000	11620315
	CPOR 7	57639608	63384581	#FF0000	5744973
	CPOR 15	63398607	126799605	#564208	63400998
	CPOR 26	126666438	133922172	#C9FE31	7255734
	CPOR 26	133904861	135280462	#C9FE31	1375601
**HGL 4**	CPOR 20	106400	62352894	#69E283	62246494
	CPOR 20	62370495	70846679	#69E283	8476184
	CPOR 12	70863440	102034657	#9B0496	31171217
**HGL 5**	CPOR 9	160511	42805753	#BF726D	42645242
	CPOR 16	43484719	57289924	#81A8F8	13805205
	CPOR 16	58479292	92829925	#81A8F8	34350633
	CPOR 1	92830201	127447574	#A6F1FC	34617373
**HGL 6**	CPOR 13	53270	18375906	#11559D	18322636
	CPOR 11	18375945	33304229	#4C0851	14928284
	CPOR 6	33780949	76732894	#7F00FF	42951945
	CPOR 2	76936750	78593218	#7FFF00	1656468
	CPOR 6	78599078	86929475	#7F00FF	8330397
	CPOR 22	87282749	105228490	#007F00	17945741
**HGL 7**	CPOR 3	21547	56749025	#D9C001	56727478
	CPOR 25	56820696	106804146	#49FEF6	49983450
**HGL 8**	CPOR 22	10448	33035695	#007F00	33025247
	CPOR 5	33798859	104078146	#09FC8D	70279287
	CPOR 4	104078146	112413322	#26B63B	8335176
	CPOR 5	112414290	123857878	#09FC8D	11443588
	CPOR 4	123843312	124925466	#26B63B	1082154
**HGL 9**	CPOR 6	23557	19578468	#7F00FF	19554911
	CPOR 2	20452633	24000542	#7FFF00	3547909
	CPOR 2	24527022	25661943	#7FFF00	1134921
	CPOR 6	33605216	66266865	#7F00FF	32661649
	CPOR 4	66342245	91888718	#26B63B	25546473
	CPOR 24	91905920	93742470	#AF0926	1836550
**HGL X**	CPOR X	1125418	5620862	#01FC33	4495444
	CPOR X	5493381	76902657	#01FC33	71409276
	CPOR X	77958170	85598447	#01FC33	7640277
	CPOR X	88307189	153250973	#01FC33	64943784

The column names and the color code of the chromosome are the same as in the Supplementary Table S2. In first column green color indicates the complete agreement with FISH (L > 5Mbp), blue color markers new synteny blocks (1 Mbp < L < 5 Mbp), yellow color shows complete agreement with previous alignment, but some disagreement with FISH.

Supplementary Table S6 (part 1). Syntenic blocks with a minimum cut-off length of 50 kilobases in the reference human genome without regard to block orientation.

scaffold	query	start	end	color	target_len
**HSA 1**	HGL 7	903444	59927569	#C2FB8F	59024125
	HGL 20	59945679	121122313	#F7FE2D	61176634
	HGL 25	143938007	145128659	#682292	1190652
	HGL 20	145600531	157496542	#F7FE2D	11896011
	HGL 27	157489663	170930989	#FF7F00	13441326
	HGL 25	171038789	227463930	#682292	56425141
	HGL 4	228661429	239931861	#0C404C	11270432
	HGL 25	240013784	246962717	#682292	6948933
**HSA 10**	HGL 4	88592	3186999	#0C404C	3098407
	HGL 6	3181979	5407032	#60FA36	2225053
	HGL 4	5407677	12093571	#0C404C	6685894
	HGL 27	12058685	15414481	#FF7F00	3355796
	HGL 6	15411024	27242291	#60FA36	11831267
	HGL 27	27486990	36879500	#FF7F00	9392510
	HGL 10	42784023	50141381	#00007F	7357358
	HGL 13	50194148	79794251	#097F8D	29600103
	HGL 10	80258809	87216735	#00007F	6957926
	HGL 13	87237441	133561533	#097F8D	46324092
**HSA 11**	HGL 8	192132	1577388	#6A78D4	1385256
	HGL 22	1620561	3239511	#00FFFF	1618950
	HGL 8	3605106	32796426	#6A78D4	29191320
	HGL 22	32809111	48497313	#00FFFF	15688202
	HGL 22	56696639	67678162	#00FFFF	10981523
	HGL 22	67992983	71513052	#00FFFF	3520069
	HGL 8	71915623	134973894	#6A78D4	63058271
**HSA 12**	HGL 12	12052	2745935	#DD528A	2733883
	HGL 8	2788219	8157259	#6A78D4	5369040
	HGL 8	8452104	27040704	#6A78D4	18588600
	HGL 16	27063978	30791643	#80E0F6	3727665
	HGL 16	31249288	33999965	#80E0F6	2750677
	HGL 16	38213447	63430883	#80E0F6	25217436
	HGL 16	63779114	107786213	#80E0F6	44007099
	HGL 19	107870465	133200058	#0000FF	25329593
**HSA 13**	HGL 15	19348575	114328985	#FAB28C	94980410
**HSA 14**	HGL 2	19709591	55209328	#C008AD	35499737
	HGL 28	55266489	105531522	#007FFF	50265033
**HSA 15**	HGL 9	22493501	32261428	#6E0025	9767927
	HGL 2	32609272	56254727	#C008AD	23645455
	HGL 26	56357668	78813026	#7FBF7F	22455358
	HGL 9	78864613	101732197	#6E0025	22867584
**HSA 16**	HGL 19	28451	31531343	#0000FF	31502892
	HGL 6	46578990	48615880	#60FA36	2036890

	HGL 7	48639697	90049507	#C2FB8F	41409810
HSA 17	HGL 18	142967	16654105	#00FF7F	16511138
	HGL 18	16852450	18364610	#00FF7F	1512160
	HGL 18	18691947	20319402	#00FF7F	1627455
	HGL 18	27231006	83231424	#00FF7F	56000418
HSA 18	HGL 23	112548	13919461	#D284F9	13806913
	HGL 23	20951411	80254828	#D284F9	59303417
HSA 19	HGL 29	278727	11561477	#FF00FF	11282750
	HGL 29	12645087	14658686	#FF00FF	2013599
	HGL 29	14931265	19664846	#FF00FF	4733581
	HGL 27	28098099	58576795	#FF7F00	30478696
HSA 2	HGL 26	37055	16770796	#7FBF7F	16733741
	HGL 5	17223333	87695749	#2DCDC1	70472416
	HGL 5	88113731	90235559	#2DCDC1	2121828
	HGL 5	94979408	113392708	#2DCDC1	18413300
	HGL 1	113672181	129920729	#13C840	16248548
	HGL 1	132366508	201785979	#13C840	69419471
	HGL 24	201778160	241889761	#F50241	40111601
HSA 20	HGL 9	90257	25623076	#6E0025	25532819
	HGL 9	31288770	64276314	#6E0025	32987544
HSA 21	HGL 2	5011613	6076907	#C008AD	1065294
	HGL 2	13156152	46666244	#C008AD	33510092
HSA 22	HGL 12	17064707	18178377	#DD528A	1113670
	HGL 19	19021794	32111148	#0000FF	13089354
	HGL 16	32387502	35658503	#80E0F6	3271001
	HGL 8	35648762	50785612	#6A78D4	15136850
HSA 3	HGL 4	12066	8442826	#0C404C	8430760
	HGL 2	8436423	10753965	#C008AD	2317542
	HGL 6	10753986	15102688	#60FA36	4348702
	HGL 4	15200927	38144059	#0C404C	22943132
	HGL 2	38325030	39381900	#C008AD	1056870
	HGL 6	39378656	52313051	#60FA36	12934395
	HGL 4	52315137	75243253	#0C404C	22928116
	HGL 2	75810436	90262808	#C008AD	14452372
	HGL 2	93808606	125524976	#C008AD	31716370
	HGL 6	126691286	198044771	#60FA36	71353485
HSA 4	HGL 12	554457	3863252	#DD528A	3308795
	HGL 12	4197110	8913117	#DD528A	4716007
	HGL 12	9745116	49083563	#DD528A	39338447
	HGL 12	51814471	73660001	#DD528A	21845530
	HGL 6	73671859	75904702	#60FA36	2232843
	HGL 7	75905334	82923099	#C2FB8F	7017765
	HGL 6	82924502	88078355	#60FA36	5153853
	HGL 7	88023735	99119403	#C2FB8F	11095668
	HGL 26	99221303	113396998	#7FBF7F	14175695
	HGL 1	113477832	125290328	#13C840	11812496

	HGL 1	125302632	162125322	#13C840	36822690
	HGL 11	162576717	189906408	#7F00FF	27329691
**HSA 5**	HGL 10	167066	45911317	#00007F	45744251
	HGL 10	50275558	80086509	#00007F	29810951
	HGL 11	80116042	112940839	#7F00FF	32824797
	HGL 5	112940839	121499344	#2DCDC1	8558505
	HGL 11	121927050	131038347	#7F00FF	9111297
	HGL 5	131137458	137889254	#2DCDC1	6751796
	HGL 11	137889288	150986473	#7F00FF	13097185
	HGL 5	151000851	180669574	#2DCDC1	29668723
**HSA 6**	HGL 4	181356	28593341	#0C404C	28411985
	HGL 13	29569284	49049403	#097F8D	19480119
	HGL 3	49553417	56344605	#1240C3	6791188
	HGL 22	56423621	58042502	#00FFFF	1618881
	HGL 22	60240863	80148526	#00FFFF	19907663
	HGL 3	80093178	170601377	#1240C3	90508199
**HSA 7**	HGL 3	31731	6731631	#1240C3	6699900
	HGL 21	7000233	29648663	#0C8017	22648430
	HGL 21	29744111	32480205	#0C8017	2736094
	HGL 9	32475965	35073302	#6E0025	2597337
	HGL 3	35051265	56226019	#1240C3	21174754
	HGL 3	65726831	76516704	#1240C3	10789873
	HGL 21	77069779	97873787	#0C8017	20804008
	HGL 3	97974383	102526158	#1240C3	4551775
	HGL 21	103289100	134631335	#0C8017	31342235
	HGL 24	134503076	142297012	#F50241	7793936
	HGL 22	142312535	159175861	#00FFFF	16863326
**HSA 8**	HGL 11	362093	7060477	#7F00FF	6698384
	HGL 11	8243874	12405183	#7F00FF	4161309
	HGL 11	12721538	43205136	#7F00FF	30483598
	HGL 12	47257053	55551938	#DD528A	8294885
	HGL 17	55837965	121018038	#8C5319	65180073
	HGL 12	121015853	144952043	#DD528A	23936190
**HSA 9**	HGL 14	69631	38472134	#98C405	38402503
	HGL 14	68234836	83146021	#98C405	14911185
	HGL 21	83242058	87758330	#0C8017	4516272
	HGL 24	88085921	94214148	#F50241	6128227
	HGL 22	94342341	97088795	#00FFFF	2746454
	HGL 14	97275303	138124581	#98C405	40849278
**HSA X**	HGL X	283173	57434098	#00FF00	57150925
	HGL X	63632161	75669739	#00FF00	12037578
	HGL X	76689781	81298283	#00FF00	4608502
	HGL X	82695994	87670280	#00FF00	4974286
	HGL X	91425916	149531957	#00FF00	58106041
	HGL X	151501615	155944777	#00FF00	4443162

Supplementary Table S6 (part 2). Syntenic blocks with a minimum cut-off length of 50 kilobases in the reference human genome without regard to block orientation.

scaffold	query	start	end	color	target_len
**HSA 1**	CPOR 25	903444	59881074	#D284F9	58977630
	CPOR 1	59944564	121098587	#C18B50	61154023
	CPOR 31	143938351	145128659	#FF00FF	1190308
	CPOR 1	145600531	153414439	#C18B50	7813908
	CPOR 4	153503215	157192434	#C008AD	3689219
	CPOR 7	157489667	170930989	#2DCDC1	13441322
	CPOR 31	171038789	209684464	#FF00FF	38645675
	CPOR 27	209684464	225881306	#682292	16196842
	CPOR 20	228661429	239931861	#00FF7F	11270432
	CPOR 31	240013784	241450246	#FF00FF	1436462
	CPOR 27	241459285	246962717	#682292	5503432
	CPOR 2	247370354	248918969	#766A7A	1548615
**HSA 10**	CPOR 20	88592	3194890	#00FF7F	3106298
	CPOR 22	3213662	5382295	#F7FE2D	2168633
	CPOR 20	5407677	12093571	#00FF7F	6685894
	CPOR 7	12058685	15411017	#2DCDC1	3352332
	CPOR 22	15411024	26872931	#F7FE2D	11461907
	CPOR 7	27448494	36885576	#2DCDC1	9437082
	CPOR 24	42403746	50141381	#00FFFF	7737635
	CPOR 28	50194392	80211745	#7FBF7F	30017353
	CPOR 24	80258809	87216735	#00FFFF	6957926
	CPOR 28	87237441	94612562	#7FBF7F	7375121
	CPOR 2	94680127	133423664	#766A7A	38743537
**HSA 11**	CPOR 4	192132	1577388	#C008AD	1385256
	CPOR 11	1595851	3239511	#6E0025	1643660
	CPOR 5	3605106	9275140	#1240C3	5670034
	CPOR 4	9277181	18118624	#C008AD	8841443
	CPOR 5	18236654	32796688	#1240C3	14560034
	CPOR 11	32802442	48245061	#6E0025	15442619
	CPOR 11	56774931	67678162	#6E0025	10903231
	CPOR 11	67992983	71504575	#6E0025	3511592
	CPOR 5	71915623	134885434	#1240C3	62969811
**HSA 12**	CPOR 9	952073	2745935	#C2FB8F	1793862
	CPOR 22	2788219	8157259	#F7FE2D	5369040
	CPOR 22	8452104	27040704	#F7FE2D	18588600
	CPOR 8	27063978	30791643	#60FA36	3727665
	CPOR 8	31249288	33997133	#60FA36	2747845
	CPOR 8	38209113	63551845	#60FA36	25342732
	CPOR 8	63731511	107786213	#60FA36	44054702
	CPOR 17	107913428	133204347	#FAB28C	25290919
**HSA 13**	CPOR 19	19409424	61958664	#8C5319	42549240
	CPOR 3	61952113	114328985	#13C840	52376872

**HSA 14**	CPOR 27	21620992	24636404	#682292	3015412
	CPOR 1	24624505	55209328	#C18B50	30584823
	CPOR 29	55269884	105531522	#FF7F00	50261638
**HSA 15**	CPOR 4	21604056	43657409	#C008AD	22053353
	CPOR 4	43658491	44748401	#C008AD	1089910
	CPOR 1	44955976	56254727	#C18B50	11298751
	CPOR 8	56357668	63823906	#60FA36	7466238
	CPOR 12	63900391	64931589	#00007F	1031198
	CPOR 8	64958289	66572101	#60FA36	1613812
	CPOR 12	66572451	69292682	#00007F	2720231
	CPOR 3	69293230	72600528	#13C840	3307298
	CPOR 8	72723550	78828929	#60FA36	6105379
	CPOR 4	78853185	101950224	#C008AD	23097039
**HSA 16**	CPOR 17	28451	31531343	#FAB28C	31502892
	CPOR 6	46581690	48615880	#0C404C	2034190
	CPOR 3	48639697	90051543	#13C840	41411846
**HSA 17**	CPOR 4	142967	4628870	#C008AD	4485903
	CPOR 4	5514149	6845307	#C008AD	1331158
	CPOR 21	6871735	16654105	#0000FF	9782370
	CPOR 21	16852450	18364610	#0000FF	1512160
	CPOR 21	18691947	20319402	#0000FF	1627455
	CPOR 4	27197291	45499992	#C008AD	18302701
	CPOR 21	45632770	47335354	#0000FF	1702584
	CPOR 4	47480742	62228764	#C008AD	14748022
	CPOR 21	62366674	64758892	#0000FF	2392218
	CPOR 21	64969422	69146693	#0000FF	4177271
	CPOR 18	69240889	73209171	#80E0F6	3968282
	CPOR 21	72972437	83231424	#0000FF	10258987
**HSA 18**	CPOR 23	112548	13834038	#0C8017	13721490
	CPOR 23	20947738	80254828	#0C8017	59307090
**HSA 19**	CPOR 11	474906	11561477	#6E0025	11086571
	CPOR 11	12645065	19551453	#6E0025	6906388
	CPOR 7	27999787	58574293	#2DCDC1	30574506
**HSA 2**	CPOR 8	37055	16741051	#60FA36	16703996
	CPOR 1	17215029	53634302	#C18B50	36419273
	CPOR 16	53634559	90235559	#98C405	36601000
	CPOR 16	95641982	97387534	#98C405	1745552
	CPOR 16	97643499	113384792	#98C405	15741293
	CPOR 18	113672181	129920729	#80E0F6	16248548
	CPOR 18	132366508	201783809	#80E0F6	69417301
	CPOR 27	201778160	218475084	#682292	16696924
	CPOR 1	218435809	224710822	#C18B50	6275013
	CPOR 27	224746425	241892126	#682292	17145701
**HSA 20**	CPOR 6	85809	25624405	#0C404C	25538596
	CPOR 6	31295126	64276314	#0C404C	32981188
**HSA 21**	CPOR 14	5011613	6076907	#DD528A	1065294

	CPOR 14	14105860	25391714	#DD528A	11285854
	CPOR 7	25371677	33480516	#2DCDC1	8108839
	CPOR 14	33473440	46666244	#DD528A	13192804
**HSA 22**	CPOR 10	17141817	18178377	#6A78D4	1036560
	CPOR 17	19021794	32111152	#FAB28C	13089358
	CPOR 8	32387502	35625560	#60FA36	3238058
	CPOR 22	35656240	50783890	#F7FE2D	15127650
**HSA 3**	CPOR 20	12066	8442826	#00FF7F	8430760
	CPOR 14	8427346	10727172	#DD528A	2299826
	CPOR 22	10753986	15102688	#F7FE2D	4348702
	CPOR 12	15200927	32650039	#00007F	17449112
	CPOR 20	32684946	38138616	#00FF7F	5453670
	CPOR 14	38122246	39381900	#DD528A	1259654
	CPOR 13	39383014	48199660	#7F00FF	8816646
	CPOR 6	48348173	52313051	#0C404C	3964878
	CPOR 12	52315137	66141471	#00007F	13826334
	CPOR 20	66213651	75243253	#00FF7F	9029602
	CPOR 14	75810436	90261169	#DD528A	14450733
	CPOR 14	93808606	125524976	#DD528A	31716370
	CPOR 22	126567248	128816833	#F7FE2D	2249585
	CPOR 6	130212776	167172132	#0C404C	36959356
	CPOR 11	167638488	182728884	#6E0025	15090396
	CPOR 13	182729166	198044771	#7F00FF	15315605
**HSA 4**	CPOR 16	498744	3863252	#98C405	3364508
	CPOR 16	4197110	8913117	#98C405	4716007
	CPOR 16	9763159	20745871	#98C405	10982712
	CPOR 30	20748954	49083563	#007FFF	28334609
	CPOR 30	51814471	73650509	#007FFF	21836038
	CPOR 2	73671859	75567434	#766A7A	1895575
	CPOR 3	75897187	82923099	#13C840	7025912
	CPOR 6	82923215	88078355	#0C404C	5155140
	CPOR 3	88023735	98811188	#13C840	10787453
	CPOR 13	99221303	162184448	#7F00FF	62963145
	CPOR 26	162585130	189915207	#F50241	27330077
**HSA 5**	CPOR 10	189445	45911317	#6A78D4	45721872
	CPOR 10	50275558	80084130	#6A78D4	29808572
	CPOR 2	80116042	107849966	#766A7A	27733924
	CPOR 22	107846821	109228131	#F7FE2D	1381310
	CPOR 2	109144667	112940839	#766A7A	3796172
	CPOR 9	112940839	120921505	#C2FB8F	7980666
	CPOR 19	121849424	131070339	#8C5319	9220915
	CPOR 9	131137458	137889254	#C2FB8F	6751796
	CPOR 19	138188172	150986473	#8C5319	12798301
	CPOR 9	151000851	180669574	#C2FB8F	29668723
**HSA 6**	CPOR 20	181356	28593341	#00FF7F	28411985
	CPOR 28	29548395	49050869	#7FBF7F	19502474

	CPOR 15	49596597	52933893	#097F8D	3337296
	CPOR 7	52942853	56352609	#2DCDC1	3409756
	CPOR 12	56355395	58315846	#00007F	1960451
	CPOR 12	60240863	63857271	#00007F	3616408
	CPOR 17	63872374	80148526	#FAB28C	16276152
	CPOR 15	80101386	99870796	#097F8D	19769410
	CPOR 7	99874430	116593592	#2DCDC1	16719162
	CPOR 26	116614588	127403458	#F50241	10788870
	CPOR 15	127269648	168060722	#097F8D	40791074
	CPOR 7	168150961	170581093	#2DCDC1	2430132
**HSA 7**	CPOR 24	31731	6730960	#00FFFF	6699229
	CPOR 2	7021513	29648663	#766A7A	22627150
	CPOR 2	29744111	32480205	#766A7A	2736094
	CPOR 24	32475945	51358487	#00FFFF	18882542
	CPOR 24	51449148	56226019	#00FFFF	4776871
	CPOR 24	65726831	76516704	#00FFFF	10789873
	CPOR 2	77069779	97873787	#766A7A	20804008
	CPOR 24	97974383	102526158	#00FFFF	4551775
	CPOR 2	103289099	134631312	#766A7A	31342213
	CPOR 4	134509504	159331441	#C008AD	24821937
**HSA 8**	CPOR 26	352195	7060496	#F50241	6708301
	CPOR 26	8243874	12349078	#F50241	4105204
	CPOR 26	12721538	30625448	#F50241	17903910
	CPOR 31	30575610	42802021	#FF00FF	12226411
	CPOR 10	47257053	61790204	#6A78D4	14533151
	CPOR 12	61852639	121014928	#00007F	59162289
	CPOR 9	120986762	144953666	#C2FB8F	23966904
**HSA 9**	CPOR 1	69631	38472134	#C18B50	38402503
	CPOR 11	60842861	62148001	#6E0025	1305140
	CPOR 1	68234836	83195588	#C18B50	14960752
	CPOR 2	83210354	87719406	#766A7A	4509052
	CPOR 11	88161679	97087976	#6E0025	8926297
	CPOR 1	97275303	138124581	#C18B50	40849278
**HSA X**	CPOR X	4099681	8033013	#00FF00	3933332
	CPOR X	7897921	56302272	#00FF00	48404351
	CPOR X	63632161	75517138	#00FF00	11884977
	CPOR X	76854279	81298283	#00FF00	4444004
	CPOR X	83415551	86057124	#00FF00	2641573
	CPOR X	95223286	155942085	#00FF00	60718799

The column names and the color code of the chromosome are the same as in the Supplementary Table S3. In first column green color indicates the complete agreement with FISH (L > 5Mbp), blue color markers new synteny blocks (1 Mbp < L < 5 Mbp), yellow color shows complete agreement with previous alignment, but some disagreement with FISH.

Supplementary Table S7. Number of conserved autosomal evolutionary segments identified in *Cavia porcellus* (CPOR) and *Heterocephalus glaber* (HGL) genomes when comparing with *Heterocephalus glaber* and *Homo sapiens* (HSA).

	HGL			HSA		
	Chr. paint.	5 Mbp	1Mbp	Chr. paint.	5 Mbp	1Mbp
CPOR	73 (84)	73 (79)	73 (433)	90 (102)	90 (100)	90 (536)
	CPOR			HSA		
HGL	76 (91)	76 (93)	77 (448)	70 (80)	70 (80)	70 (524)

Chr. paint. – comparative chromosome painting data, 5 Mbp – pairwise comparison with the 5 Mbp threshold, 1 Mbp – pairwise comparison with the 1 Mbp threshold. First number is a number of syntenic chromosomal fragments without consideration of intrachromosomal rearrangement. In parentheses is a number of syntenic chromosomal fragments, including fragments formed by intrachromosomal rearrangements.

Supplementary Table S8. Correspondence of *Heterocephalus glaber* (HGL) superscaffolds identified in the work of Zhou et al.[43] and chromosome numbers used in present work established based on identified associations of human chromosomes. In the case of the cited work, we were guided by the color scheme shown in the Supplementary Figure S2 (Supplement to Zhou et al.[43]).

HGL chromosome, this work	Synteny, this work	HGL superscaffold number[43]	Synteny[43]
HGL 3	HSA 7/6/11/6	18	HSA 6
HGL 2	HSA 15/14/15/3/21	2	HSA 15/14/15/3/21
HGL 8	HSA 22/12/11	7	HSA 22/12/11
HGL 19	HSA 16/22/12	28	HSA 12/22/16

HGL 20?	HSA 1	26	HSA 1
HGL 25?	HSA 1	27	HSA 1
HGL X	HSA X	3 and 13	HSA X
HGL 11	HSA 1/5/1/8/4/8	4	HSA 8/4/8/5/1*/7*
HGL 23	HSA 18	22	HSA 18
HGL 18 + HGL 27	HSA 17 + HSA 19/1/10	8	HSA 10/1**/19/17
HGL 15	HSA 13	1***	HSA 13
HGL 17	HSA 8	24	HSA 8
HGL 9	HSA 20/1/20/15/20	17	HSA 15/20

* This chromosome fragments were not noted by the authors in the description, but they were presented in the color scheme[43].

** According to the human color key[43], this is a fragment of HSA 1, but the description indicates HSA 11.

*** The superscaffold number may be incorrect.
